# Supplementary material for: Metabolomic atlas of dengue virus infection reveals distinct circulating bioactive lipid signatures
Source: PLoS Negl Trop Dis. 2026 May 12;20(5):e0014327. doi: 10.1371/journal.pntd.0014327 (PMC13189415; doi:10.1371/journal.pntd.0014327)
Supplement: S1 Fig — PCA analysis score plots showing the distribution of metabolomic profiles among healthy (green), primary dengue (blue), and secondary dengue (orange). Each point represents an individual sample. The plot demonstrates distinct clustering of healthy controls and partial separation between primary and secondary dengue. (DOCX) [file pntd.0014327.s001.docx]

**
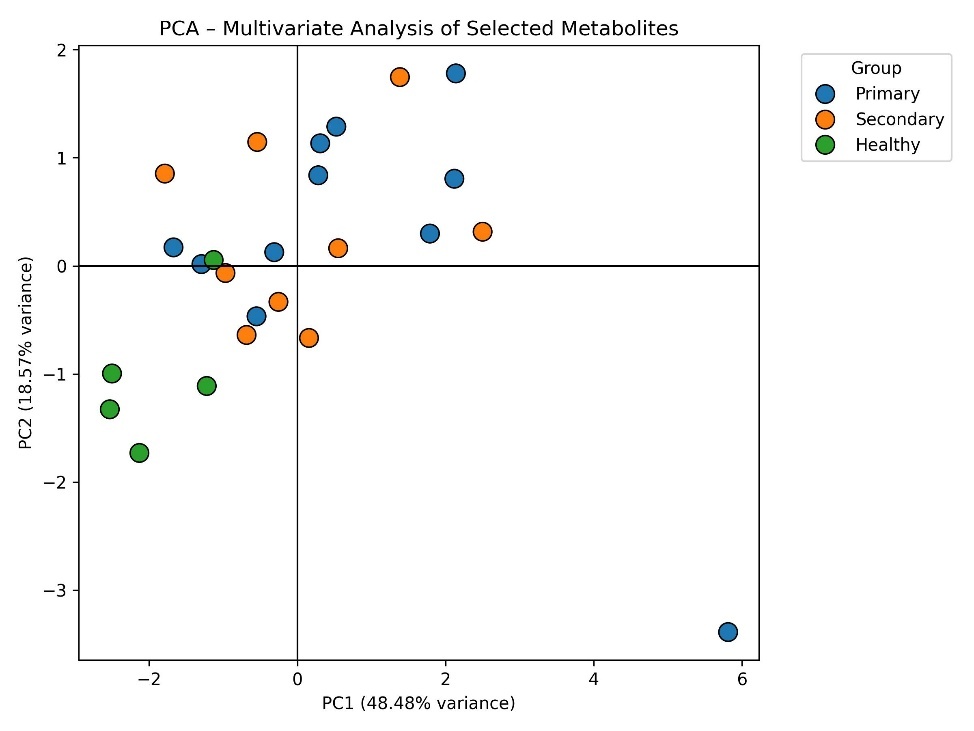
**

**S1 Fig. Principal component analysis of metabolomic profiles in dengue.** PCA score plots showing the distribution of metabolomic profiles among healthy (green), primary dengue (blue), and secondary dengue (orange). Each point represents an individual sample. The plot demonstrates distinct clustering of healthy controls and partial separation between primary and secondary dengue.
