# Supplementary material for: Metabolomic atlas of dengue virus infection reveals distinct circulating bioactive lipid signatures
Source: PLoS Negl Trop Dis. 2026 May 12;20(5):e0014327. doi: 10.1371/journal.pntd.0014327 (PMC13189415; doi:10.1371/journal.pntd.0014327)
Supplement: S2 Fig — ROC curves comparing the predictive performance of primary and secondary dengue in the lipidomic and clinical model: the clinical parameters (blue, AUC: 0.67), lipidomic feature (orange, AUC: 1.00) and the combined model (green, AUC:0.67) represented in the graph. The red dotted line represents the random classification (AUC = 0.5). (DOCX) [file pntd.0014327.s002.docx]

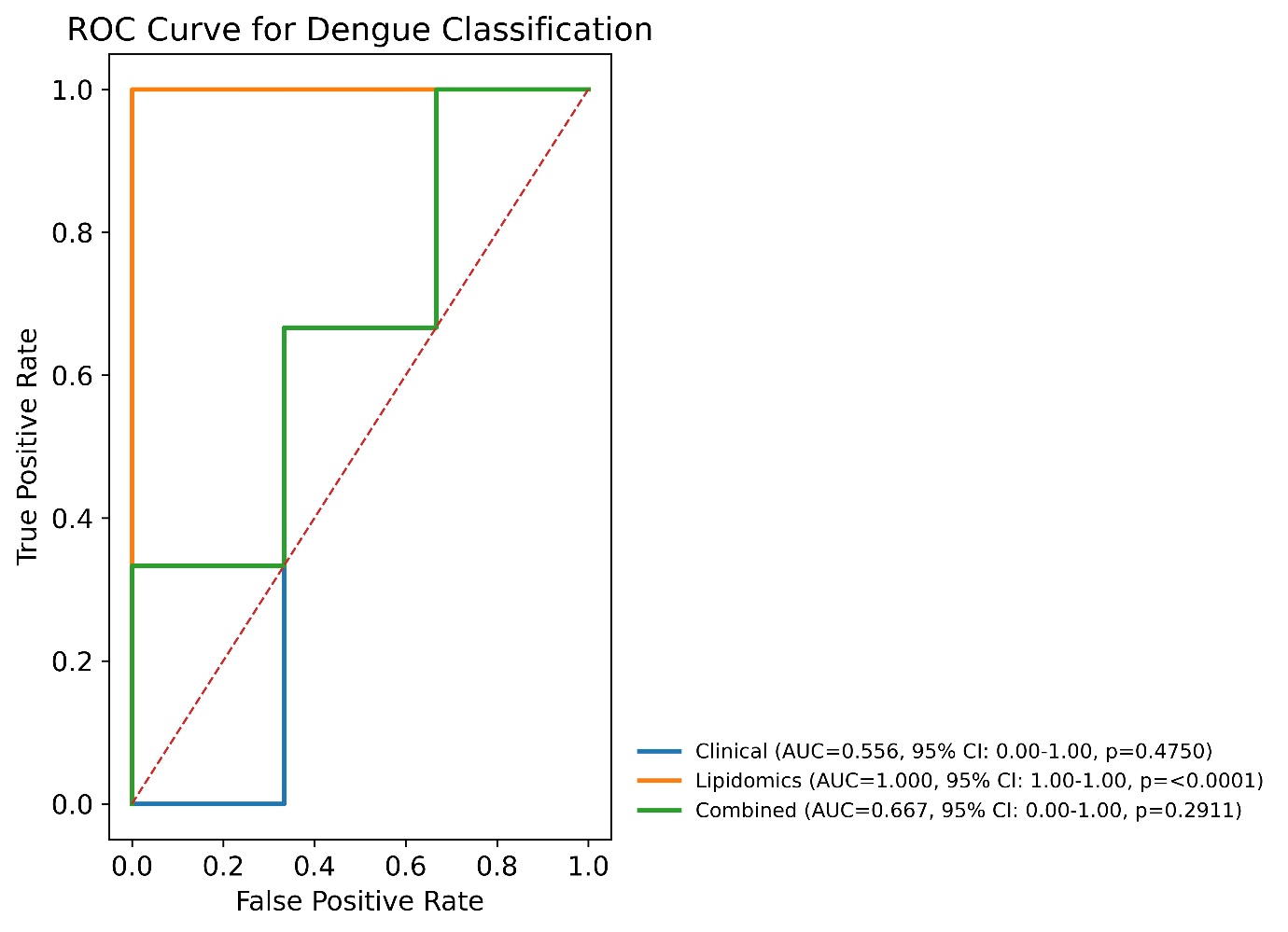


**S2 Fig. ROC curve for the dengue classification model**: ROC curves comparing the predictive performance of primary and secondary dengue in the lipidomic and clinical model: the clinical parameters (blue, AUC: 0.67), lipidomic feature (orange, AUC: 1.00) and the combined model (green, AUC:0.67) represented in the graph. The red dotted line represents the random classification (AUC=0.5).
