# Supplementary figures and images for: Metabolomic atlas of dengue virus infection reveals distinct circulating bioactive lipid signatures (part 2 of 2)
Source: PLoS Negl Trop Dis. 2026 May 12;20(5):e0014327. doi: 10.1371/journal.pntd.0014327 (PMC13189415; doi:10.1371/journal.pntd.0014327)

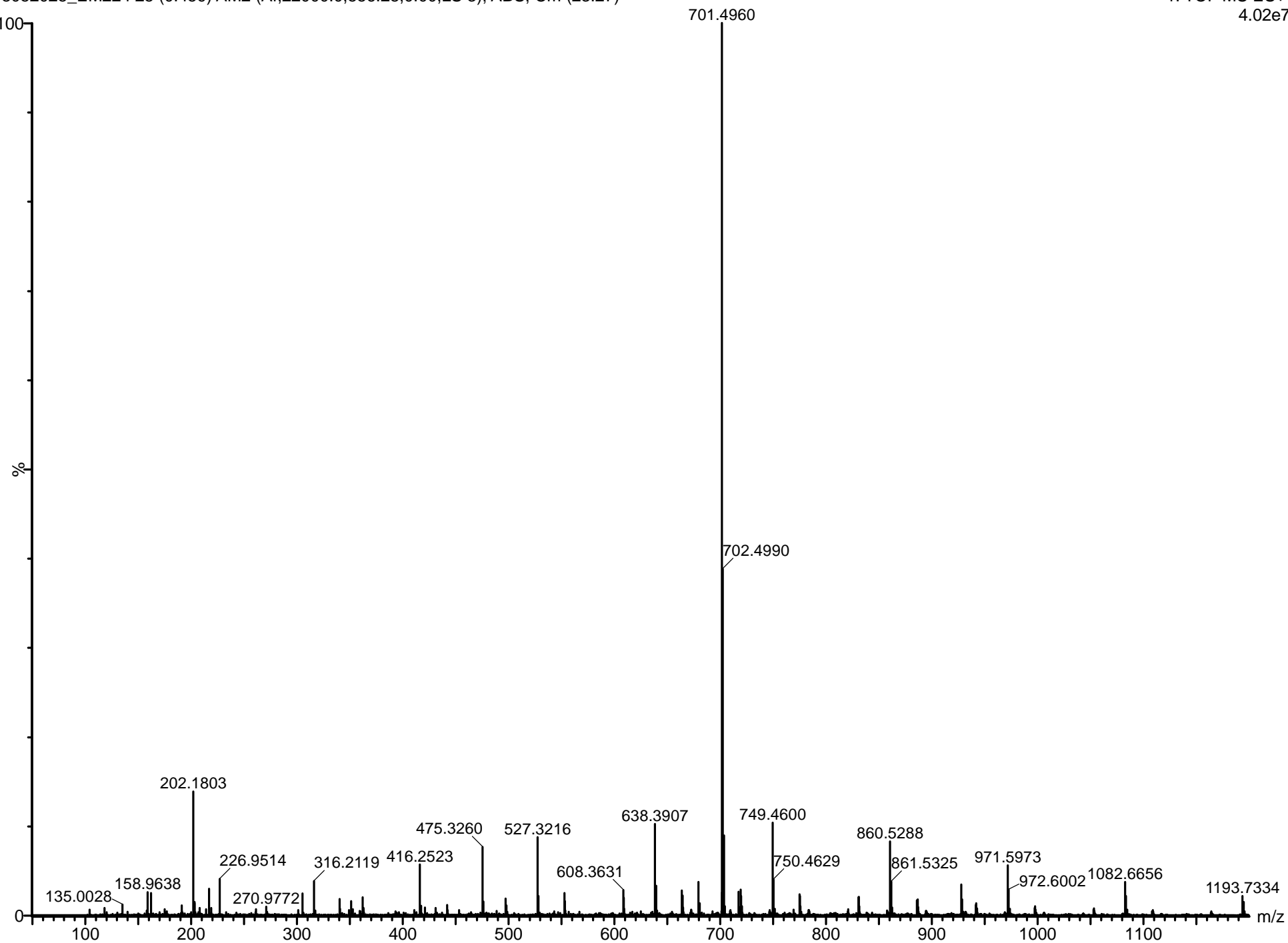

Supplement: S1 Data — Electrospray ionisation time of flight mass spectrometry (ESI-TOF MS, positive mode) spectra of the dengue cohort and ESI-TOF at different retention times. The spectra display the relative abundance (%) of detected ions across the m/z range. Prominent peaks corresponding to major ionised species are indicated. Variation in spectral profiles between retention times reflects the differences in compound composition and ionisation patterns within the sample. Data were acquired under identical instrumental conditions and are presented as representative scans. (ZIP) [file pntd.0014327.s003.zip › EM COMPLETE SAMPLES SPECTRUM/EM224 SPECTRUM RT 0.459.pdf]

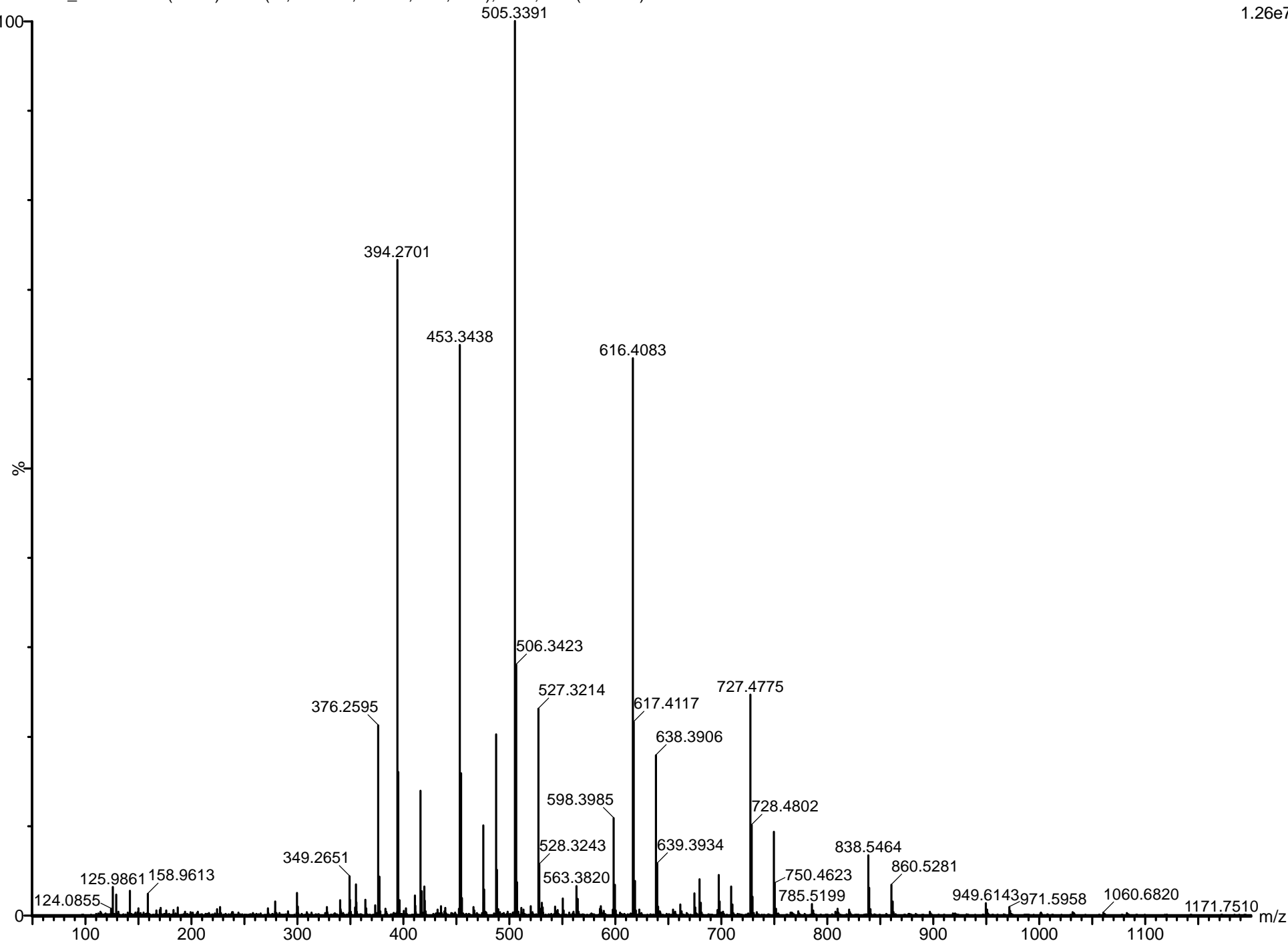

Supplement: S1 Data — Electrospray ionisation time of flight mass spectrometry (ESI-TOF MS, positive mode) spectra of the dengue cohort and ESI-TOF at different retention times. The spectra display the relative abundance (%) of detected ions across the m/z range. Prominent peaks corresponding to major ionised species are indicated. Variation in spectral profiles between retention times reflects the differences in compound composition and ionisation patterns within the sample. Data were acquired under identical instrumental conditions and are presented as representative scans. (ZIP) [file pntd.0014327.s003.zip › EM COMPLETE SAMPLES SPECTRUM/EM224 SPECTRUM RT 2.058.pdf]

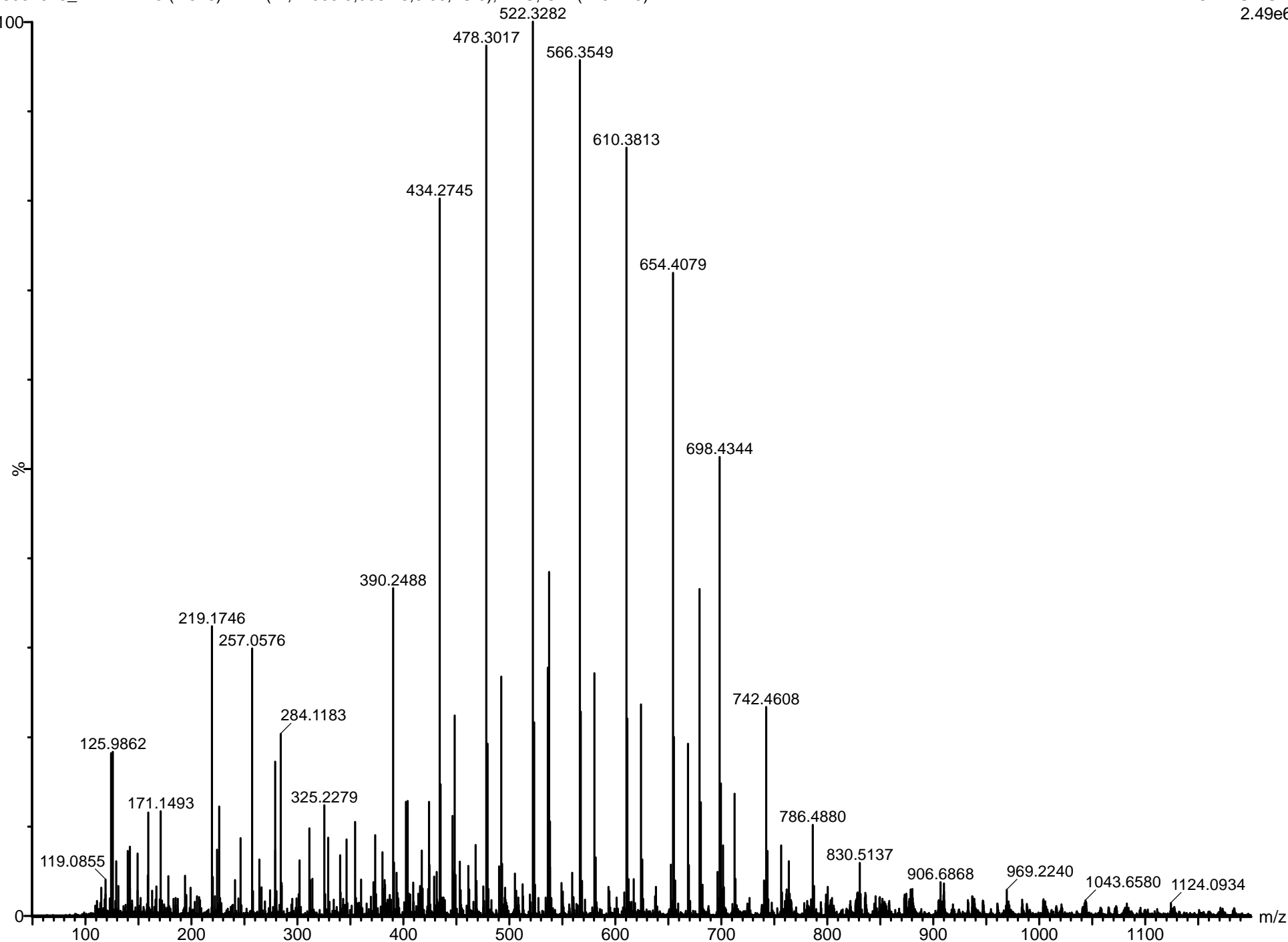

Supplement: S1 Data — Electrospray ionisation time of flight mass spectrometry (ESI-TOF MS, positive mode) spectra of the dengue cohort and ESI-TOF at different retention times. The spectra display the relative abundance (%) of detected ions across the m/z range. Prominent peaks corresponding to major ionised species are indicated. Variation in spectral profiles between retention times reflects the differences in compound composition and ionisation patterns within the sample. Data were acquired under identical instrumental conditions and are presented as representative scans. (ZIP) [file pntd.0014327.s003.zip › EM COMPLETE SAMPLES SPECTRUM/EM224 SPECTRUM RT 2.548.pdf]

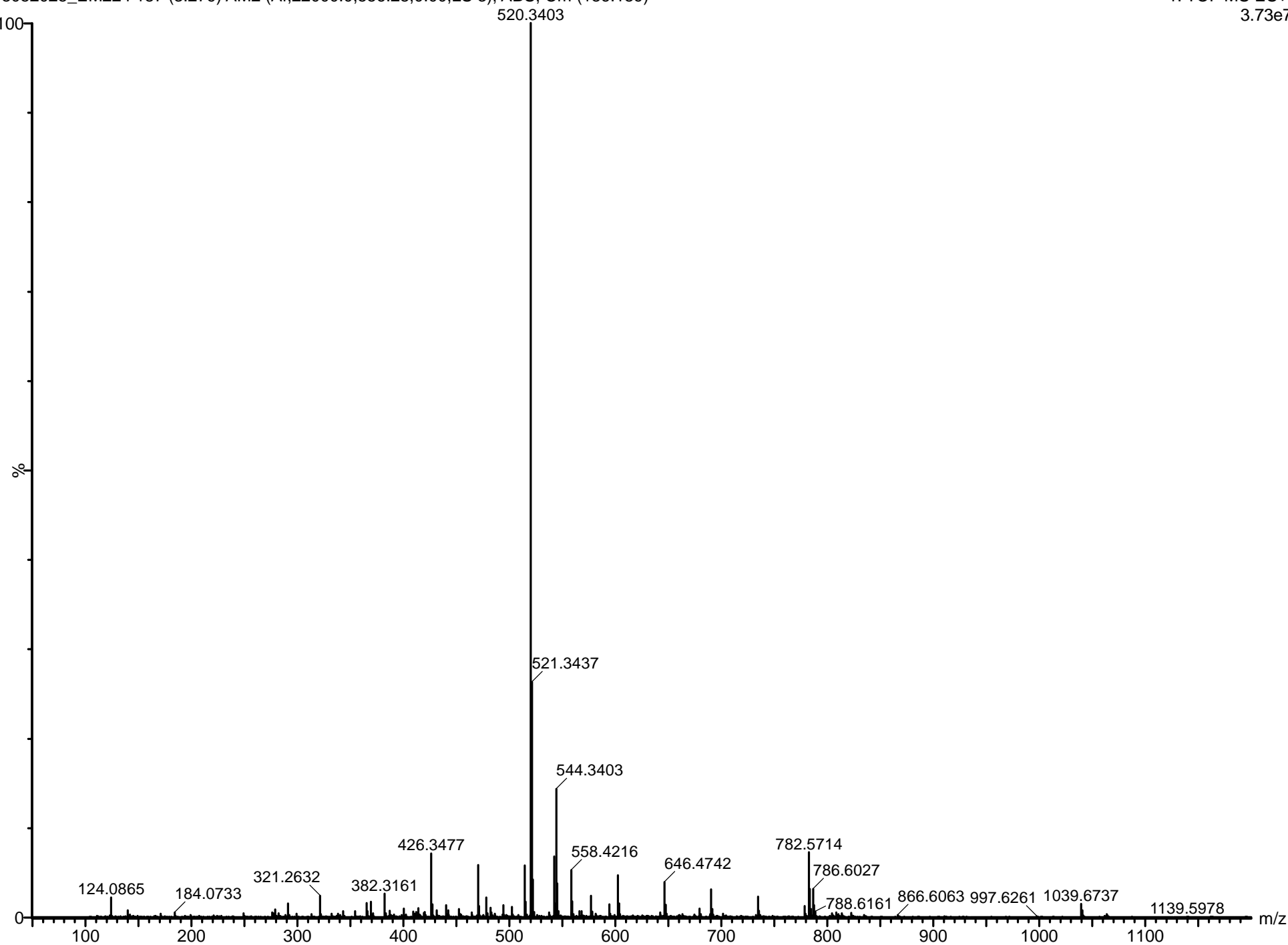

Supplement: S1 Data — Electrospray ionisation time of flight mass spectrometry (ESI-TOF MS, positive mode) spectra of the dengue cohort and ESI-TOF at different retention times. The spectra display the relative abundance (%) of detected ions across the m/z range. Prominent peaks corresponding to major ionised species are indicated. Variation in spectral profiles between retention times reflects the differences in compound composition and ionisation patterns within the sample. Data were acquired under identical instrumental conditions and are presented as representative scans. (ZIP) [file pntd.0014327.s003.zip › EM COMPLETE SAMPLES SPECTRUM/EM224 SPECTRUM RT 3.279.pdf]

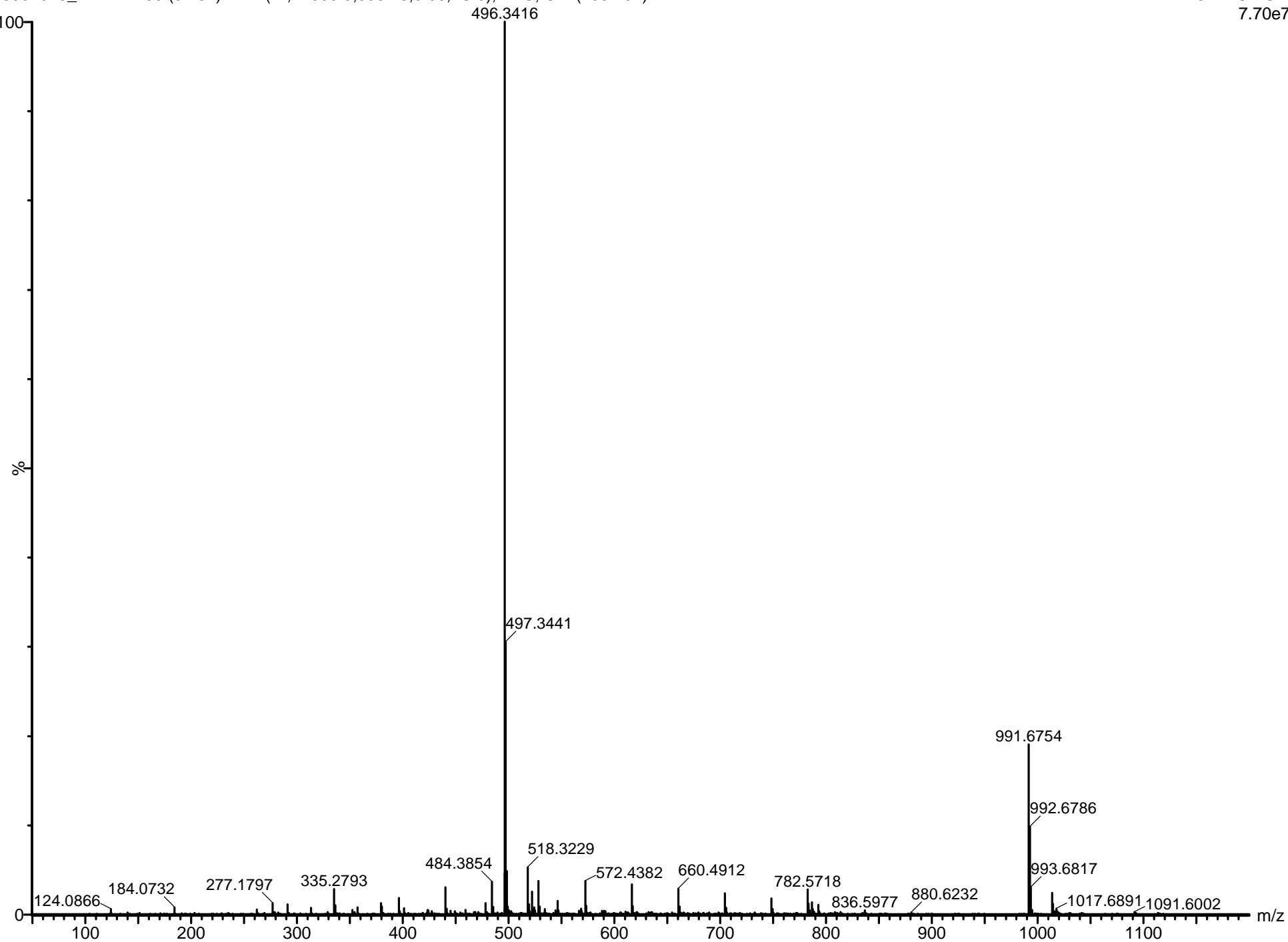

Supplement: S1 Data — Electrospray ionisation time of flight mass spectrometry (ESI-TOF MS, positive mode) spectra of the dengue cohort and ESI-TOF at different retention times. The spectra display the relative abundance (%) of detected ions across the m/z range. Prominent peaks corresponding to major ionised species are indicated. Variation in spectral profiles between retention times reflects the differences in compound composition and ionisation patterns within the sample. Data were acquired under identical instrumental conditions and are presented as representative scans. (ZIP) [file pntd.0014327.s003.zip › EM COMPLETE SAMPLES SPECTRUM/EM224 SPECTRUM RT 3.434.pdf]

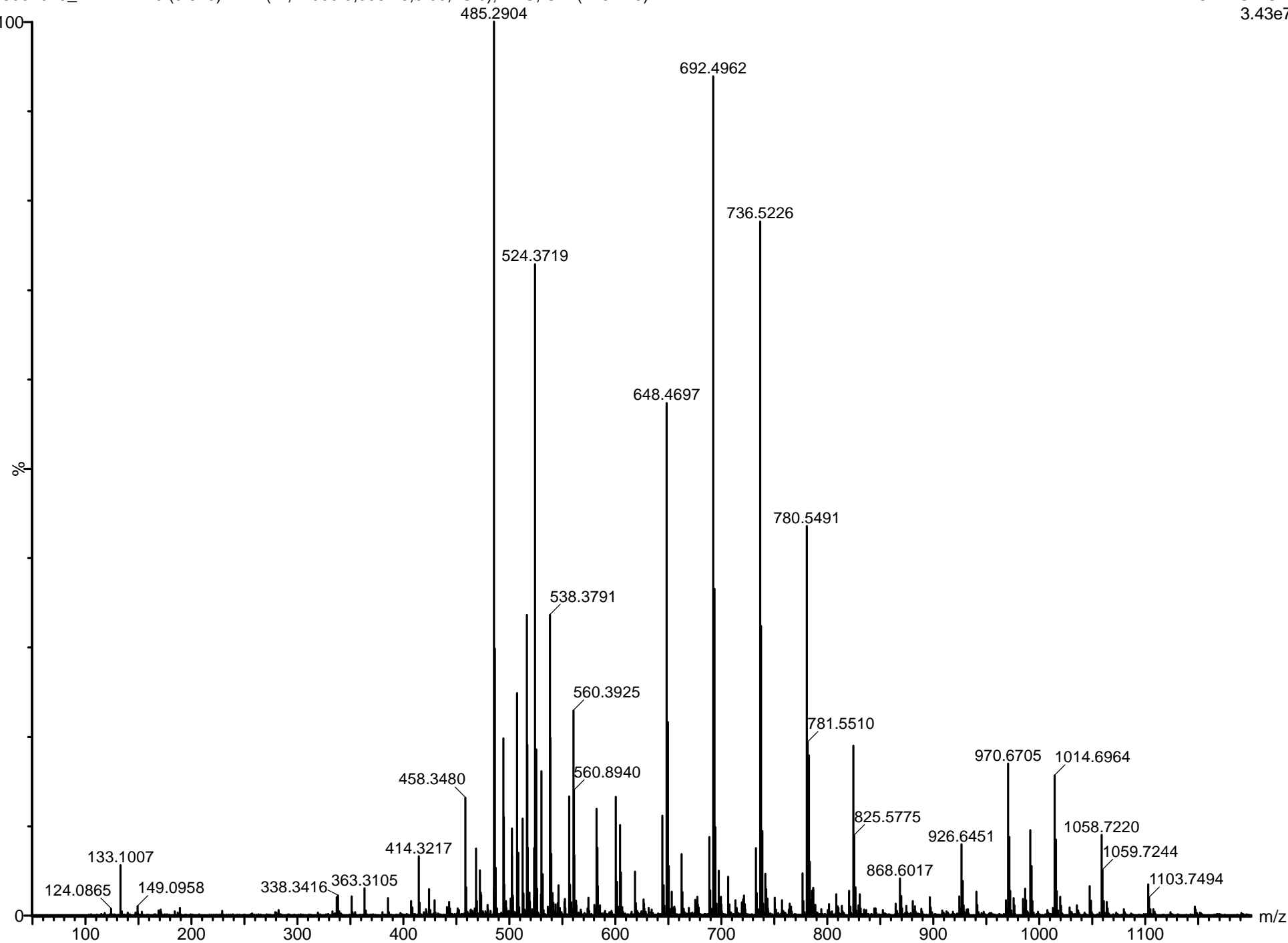

Supplement: S1 Data — Electrospray ionisation time of flight mass spectrometry (ESI-TOF MS, positive mode) spectra of the dengue cohort and ESI-TOF at different retention times. The spectra display the relative abundance (%) of detected ions across the m/z range. Prominent peaks corresponding to major ionised species are indicated. Variation in spectral profiles between retention times reflects the differences in compound composition and ionisation patterns within the sample. Data were acquired under identical instrumental conditions and are presented as representative scans. (ZIP) [file pntd.0014327.s003.zip › EM COMPLETE SAMPLES SPECTRUM/EM224 SPECTRUM RT 3.823.pdf]

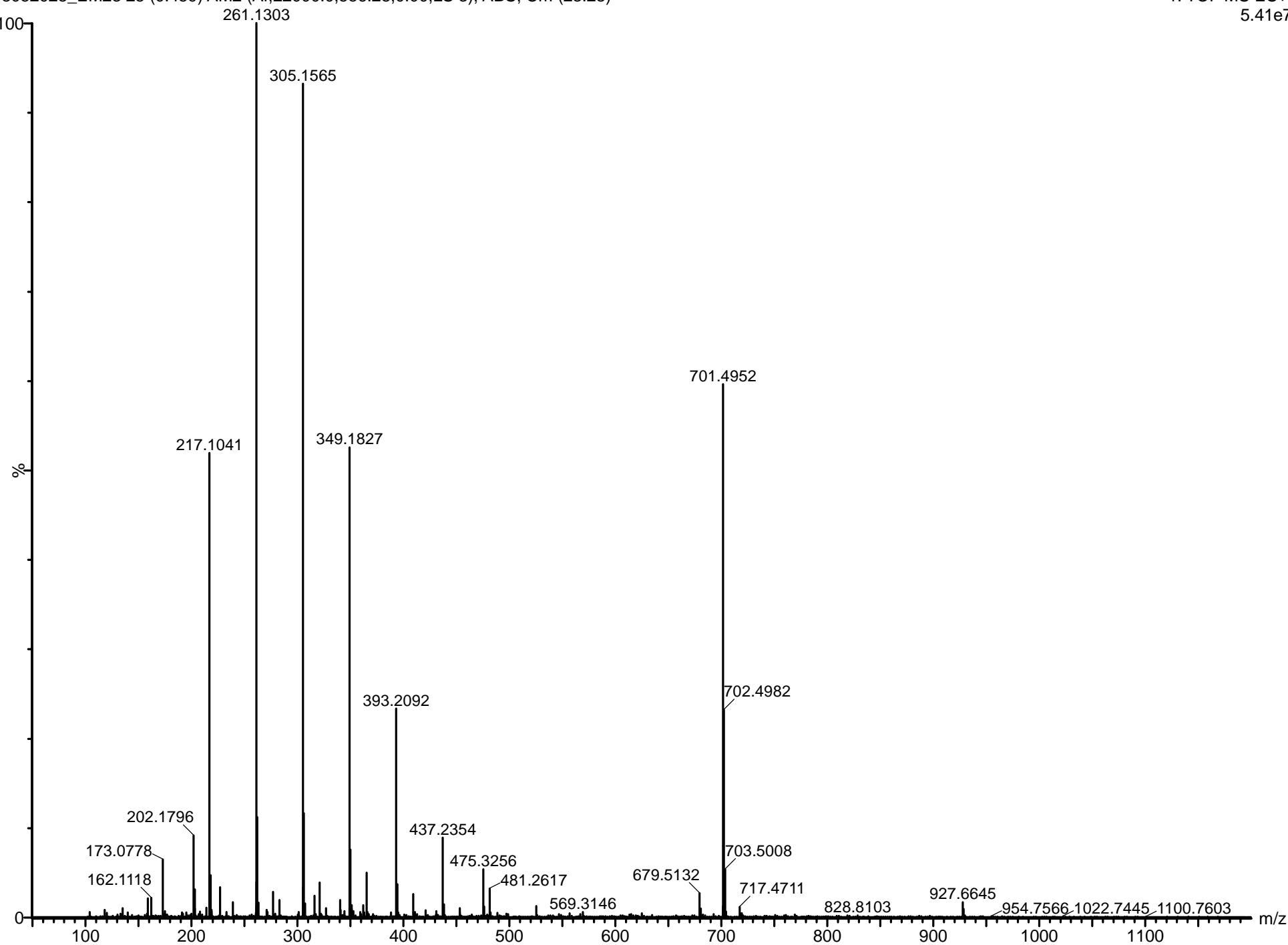

Supplement: S1 Data — Electrospray ionisation time of flight mass spectrometry (ESI-TOF MS, positive mode) spectra of the dengue cohort and ESI-TOF at different retention times. The spectra display the relative abundance (%) of detected ions across the m/z range. Prominent peaks corresponding to major ionised species are indicated. Variation in spectral profiles between retention times reflects the differences in compound composition and ionisation patterns within the sample. Data were acquired under identical instrumental conditions and are presented as representative scans. (ZIP) [file pntd.0014327.s003.zip › EM COMPLETE SAMPLES SPECTRUM/EM23 SPECTRUM RT 0.459.pdf]

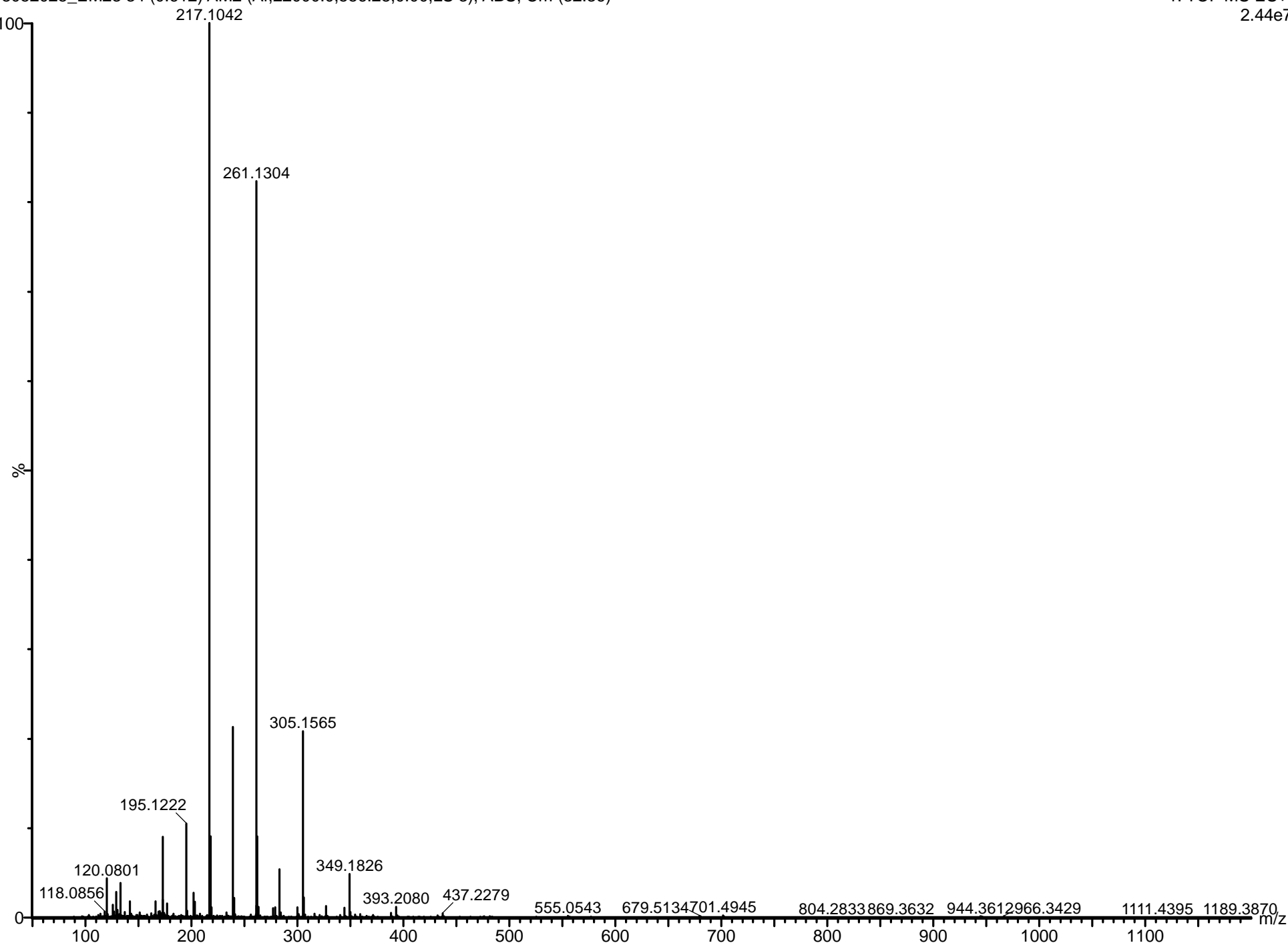

Supplement: S1 Data — Electrospray ionisation time of flight mass spectrometry (ESI-TOF MS, positive mode) spectra of the dengue cohort and ESI-TOF at different retention times. The spectra display the relative abundance (%) of detected ions across the m/z range. Prominent peaks corresponding to major ionised species are indicated. Variation in spectral profiles between retention times reflects the differences in compound composition and ionisation patterns within the sample. Data were acquired under identical instrumental conditions and are presented as representative scans. (ZIP) [file pntd.0014327.s003.zip › EM COMPLETE SAMPLES SPECTRUM/EM23 SPECTRUM RT 0.612.pdf]

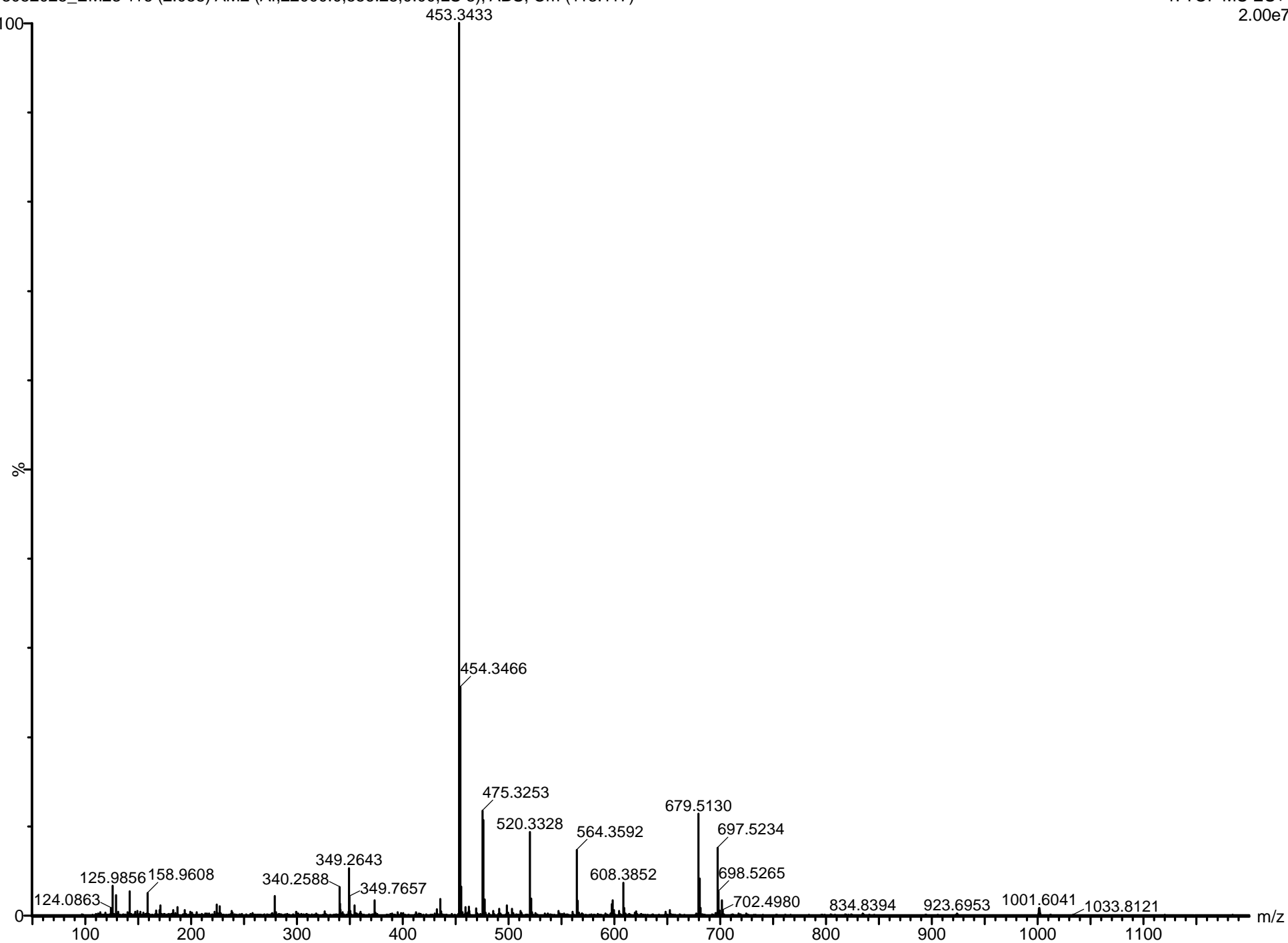

Supplement: S1 Data — Electrospray ionisation time of flight mass spectrometry (ESI-TOF MS, positive mode) spectra of the dengue cohort and ESI-TOF at different retention times. The spectra display the relative abundance (%) of detected ions across the m/z range. Prominent peaks corresponding to major ionised species are indicated. Variation in spectral profiles between retention times reflects the differences in compound composition and ionisation patterns within the sample. Data were acquired under identical instrumental conditions and are presented as representative scans. (ZIP) [file pntd.0014327.s003.zip › EM COMPLETE SAMPLES SPECTRUM/EM23 SPECTRUM RT 2.058.pdf]

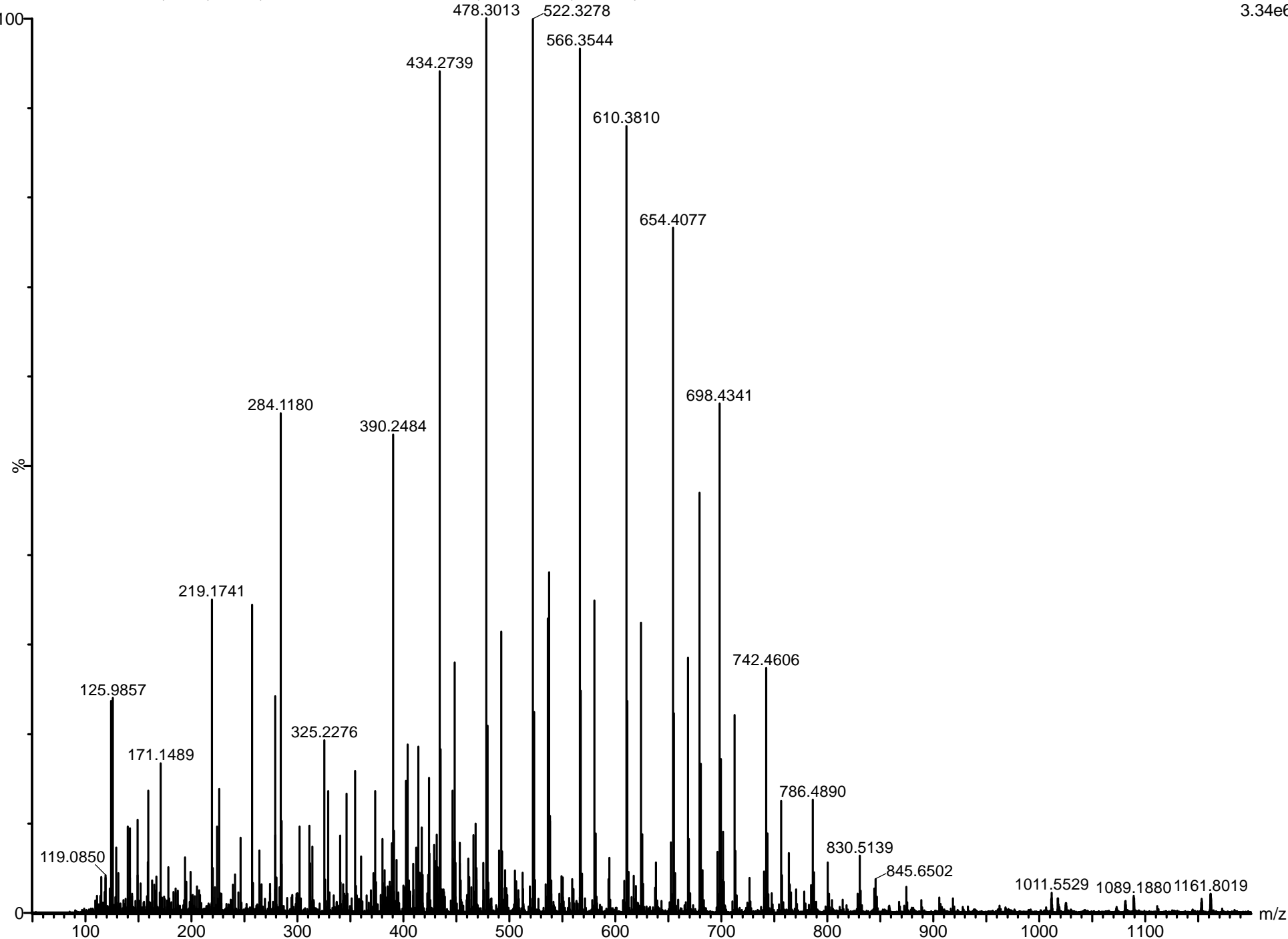

Supplement: S1 Data — Electrospray ionisation time of flight mass spectrometry (ESI-TOF MS, positive mode) spectra of the dengue cohort and ESI-TOF at different retention times. The spectra display the relative abundance (%) of detected ions across the m/z range. Prominent peaks corresponding to major ionised species are indicated. Variation in spectral profiles between retention times reflects the differences in compound composition and ionisation patterns within the sample. Data were acquired under identical instrumental conditions and are presented as representative scans. (ZIP) [file pntd.0014327.s003.zip › EM COMPLETE SAMPLES SPECTRUM/EM23 SPECTRUM RT 2.582.pdf]

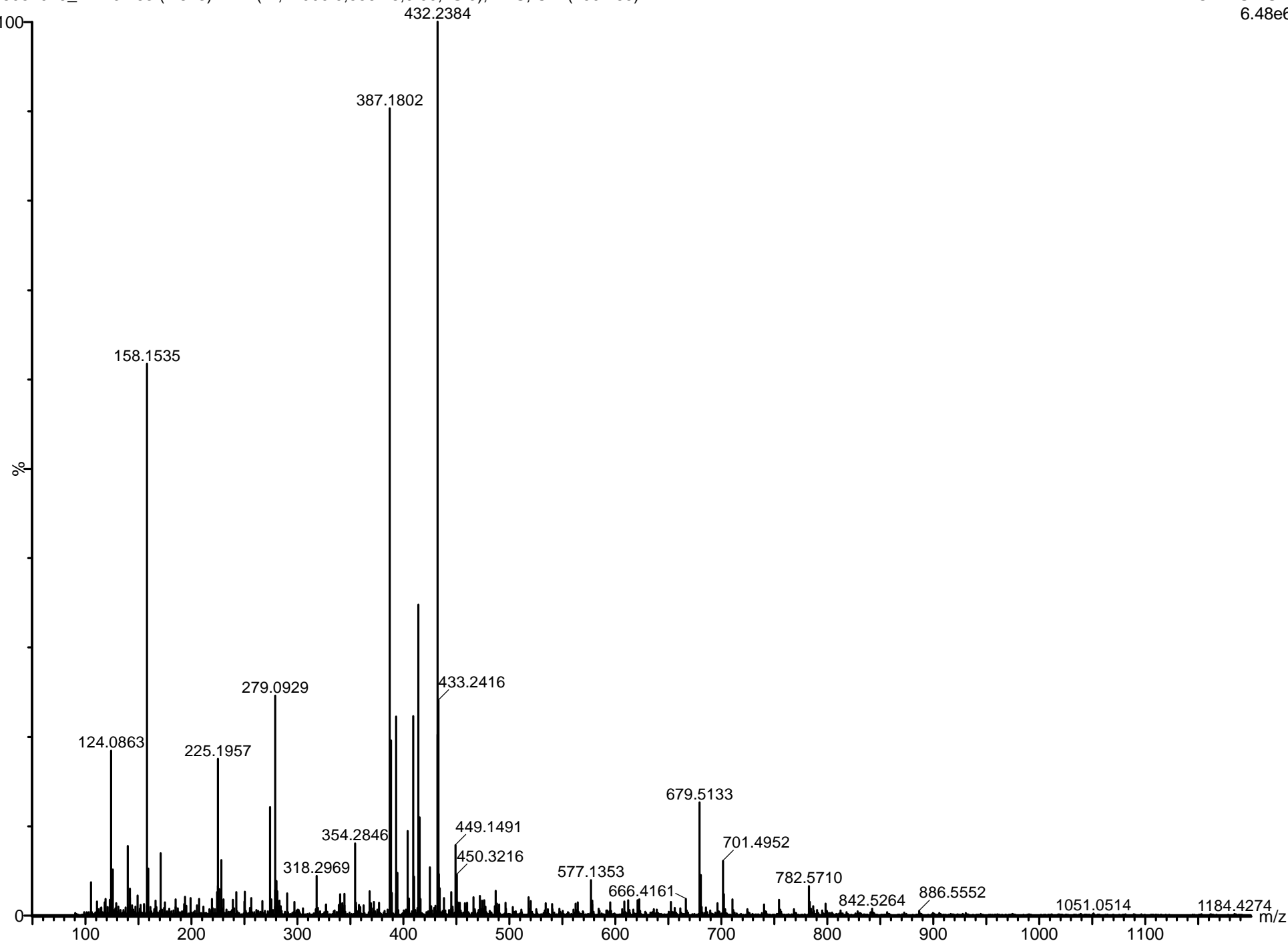

Supplement: S1 Data — Electrospray ionisation time of flight mass spectrometry (ESI-TOF MS, positive mode) spectra of the dengue cohort and ESI-TOF at different retention times. The spectra display the relative abundance (%) of detected ions across the m/z range. Prominent peaks corresponding to major ionised species are indicated. Variation in spectral profiles between retention times reflects the differences in compound composition and ionisation patterns within the sample. Data were acquired under identical instrumental conditions and are presented as representative scans. (ZIP) [file pntd.0014327.s003.zip › EM COMPLETE SAMPLES SPECTRUM/EM23 SPECTRUM RT 2.873.pdf]

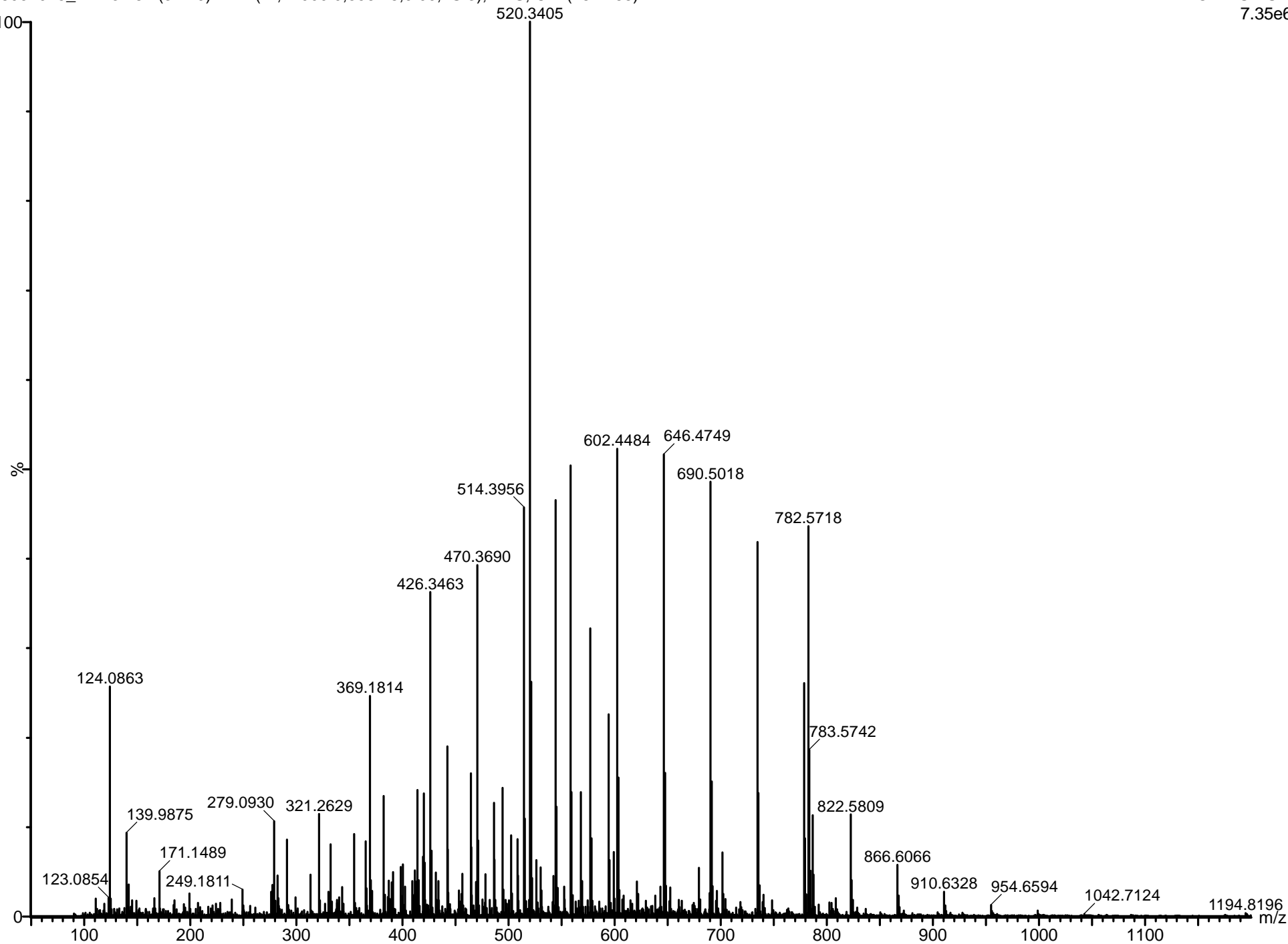

Supplement: S1 Data — Electrospray ionisation time of flight mass spectrometry (ESI-TOF MS, positive mode) spectra of the dengue cohort and ESI-TOF at different retention times. The spectra display the relative abundance (%) of detected ions across the m/z range. Prominent peaks corresponding to major ionised species are indicated. Variation in spectral profiles between retention times reflects the differences in compound composition and ionisation patterns within the sample. Data were acquired under identical instrumental conditions and are presented as representative scans. (ZIP) [file pntd.0014327.s003.zip › EM COMPLETE SAMPLES SPECTRUM/EM23 SPECTRUM RT 3.279.pdf]

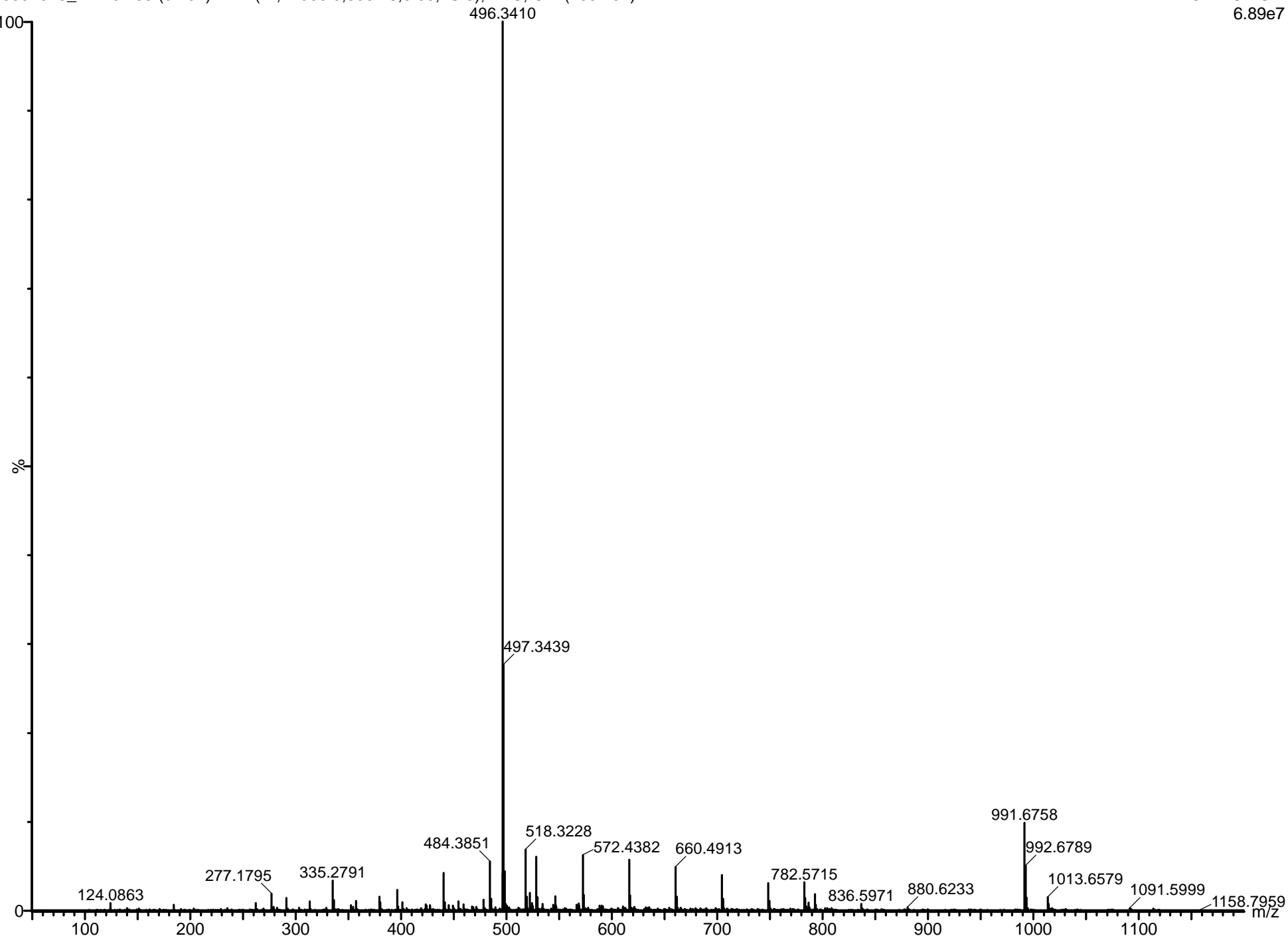

Supplement: S1 Data — Electrospray ionisation time of flight mass spectrometry (ESI-TOF MS, positive mode) spectra of the dengue cohort and ESI-TOF at different retention times. The spectra display the relative abundance (%) of detected ions across the m/z range. Prominent peaks corresponding to major ionised species are indicated. Variation in spectral profiles between retention times reflects the differences in compound composition and ionisation patterns within the sample. Data were acquired under identical instrumental conditions and are presented as representative scans. (ZIP) [file pntd.0014327.s003.zip › EM COMPLETE SAMPLES SPECTRUM/EM23 SPECTRUM RT 3.434.pdf]

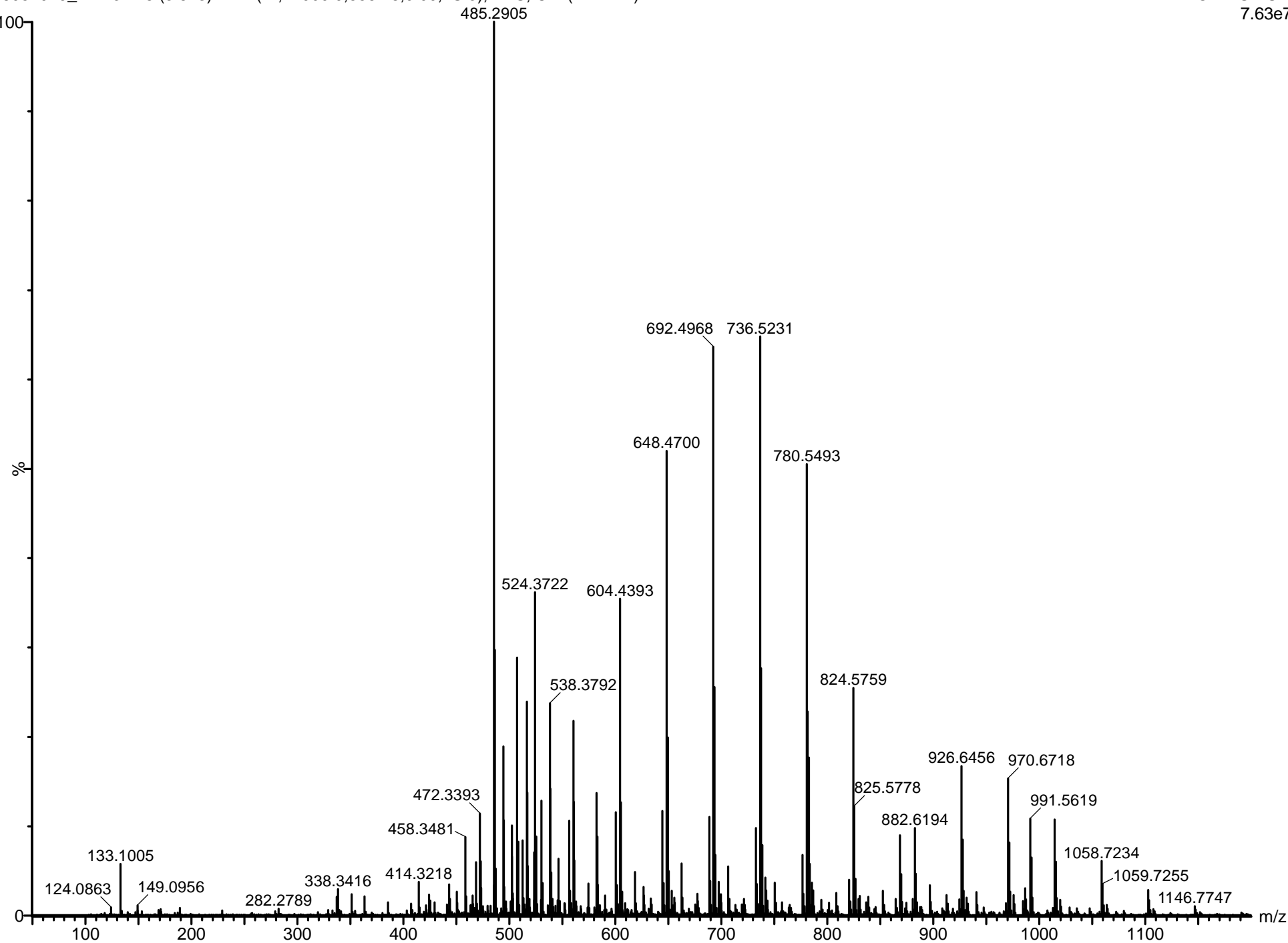

Supplement: S1 Data — Electrospray ionisation time of flight mass spectrometry (ESI-TOF MS, positive mode) spectra of the dengue cohort and ESI-TOF at different retention times. The spectra display the relative abundance (%) of detected ions across the m/z range. Prominent peaks corresponding to major ionised species are indicated. Variation in spectral profiles between retention times reflects the differences in compound composition and ionisation patterns within the sample. Data were acquired under identical instrumental conditions and are presented as representative scans. (ZIP) [file pntd.0014327.s003.zip › EM COMPLETE SAMPLES SPECTRUM/EM23 SPECTRUM RT 3.823.pdf]

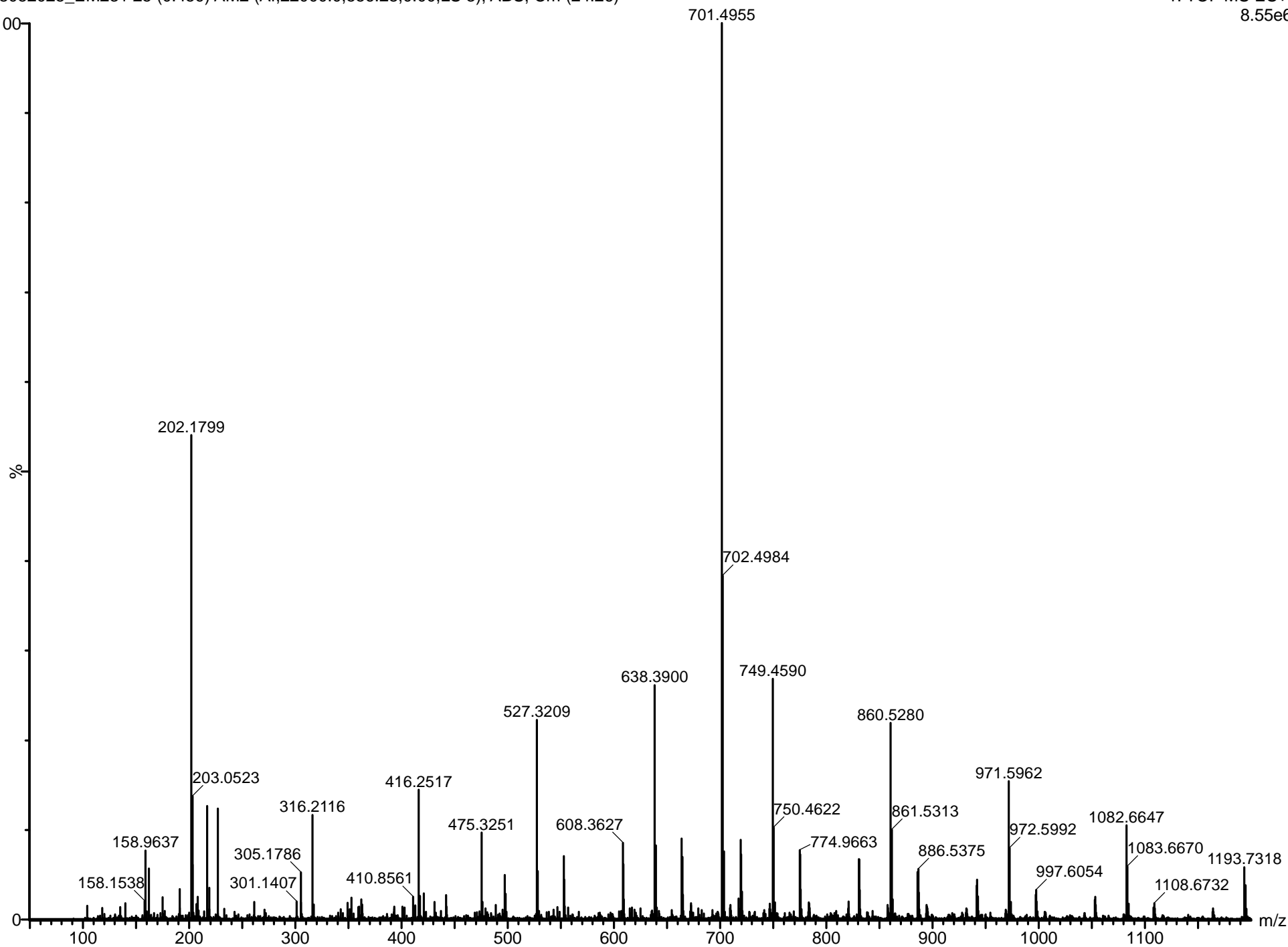

Supplement: S1 Data — Electrospray ionisation time of flight mass spectrometry (ESI-TOF MS, positive mode) spectra of the dengue cohort and ESI-TOF at different retention times. The spectra display the relative abundance (%) of detected ions across the m/z range. Prominent peaks corresponding to major ionised species are indicated. Variation in spectral profiles between retention times reflects the differences in compound composition and ionisation patterns within the sample. Data were acquired under identical instrumental conditions and are presented as representative scans. (ZIP) [file pntd.0014327.s003.zip › EM COMPLETE SAMPLES SPECTRUM/EM231 SPECTRUM RT 0.459.pdf]

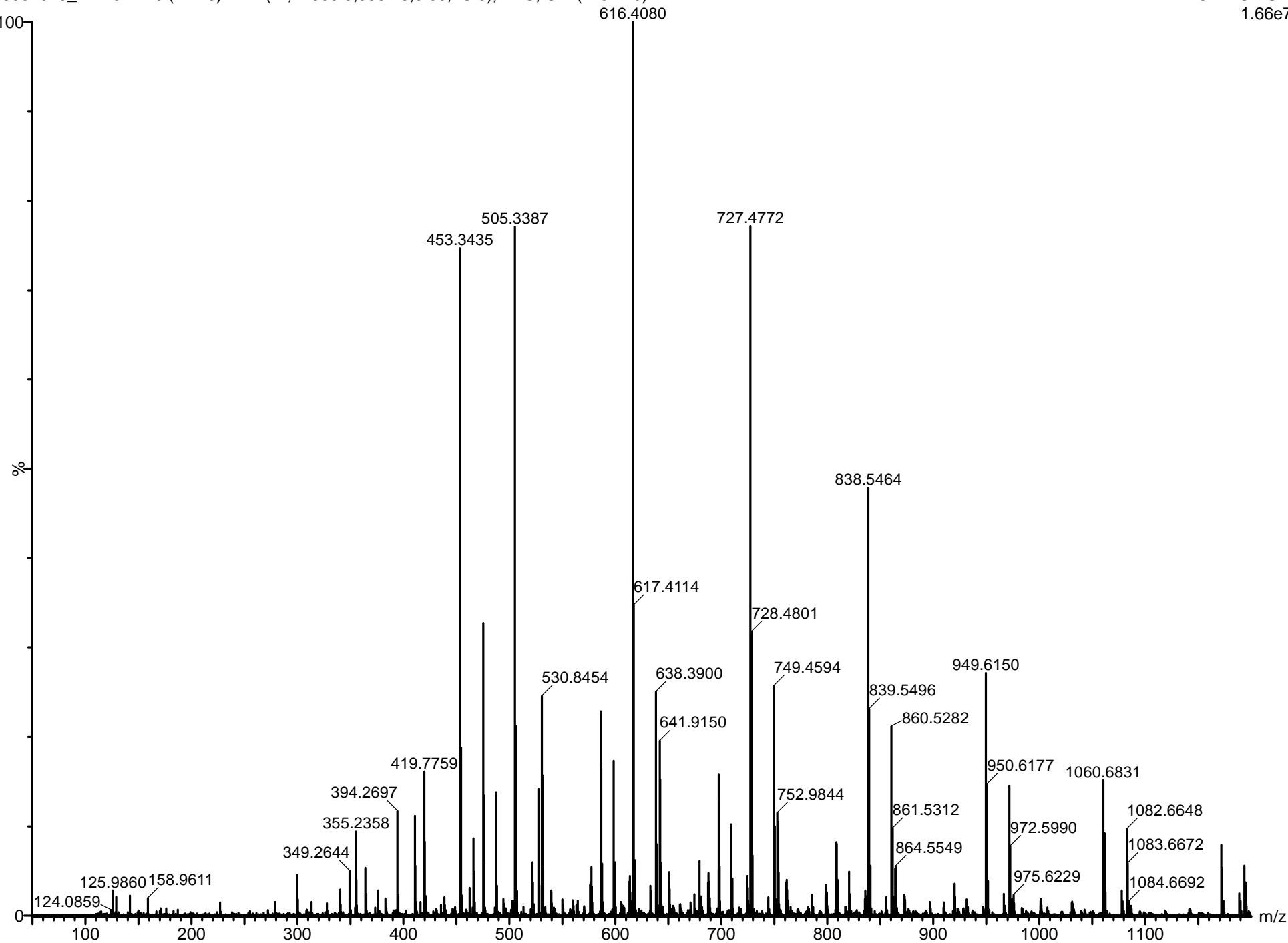

Supplement: S1 Data — Electrospray ionisation time of flight mass spectrometry (ESI-TOF MS, positive mode) spectra of the dengue cohort and ESI-TOF at different retention times. The spectra display the relative abundance (%) of detected ions across the m/z range. Prominent peaks corresponding to major ionised species are indicated. Variation in spectral profiles between retention times reflects the differences in compound composition and ionisation patterns within the sample. Data were acquired under identical instrumental conditions and are presented as representative scans. (ZIP) [file pntd.0014327.s003.zip › EM COMPLETE SAMPLES SPECTRUM/EM231 SPECTRUM RT 2.126.pdf]

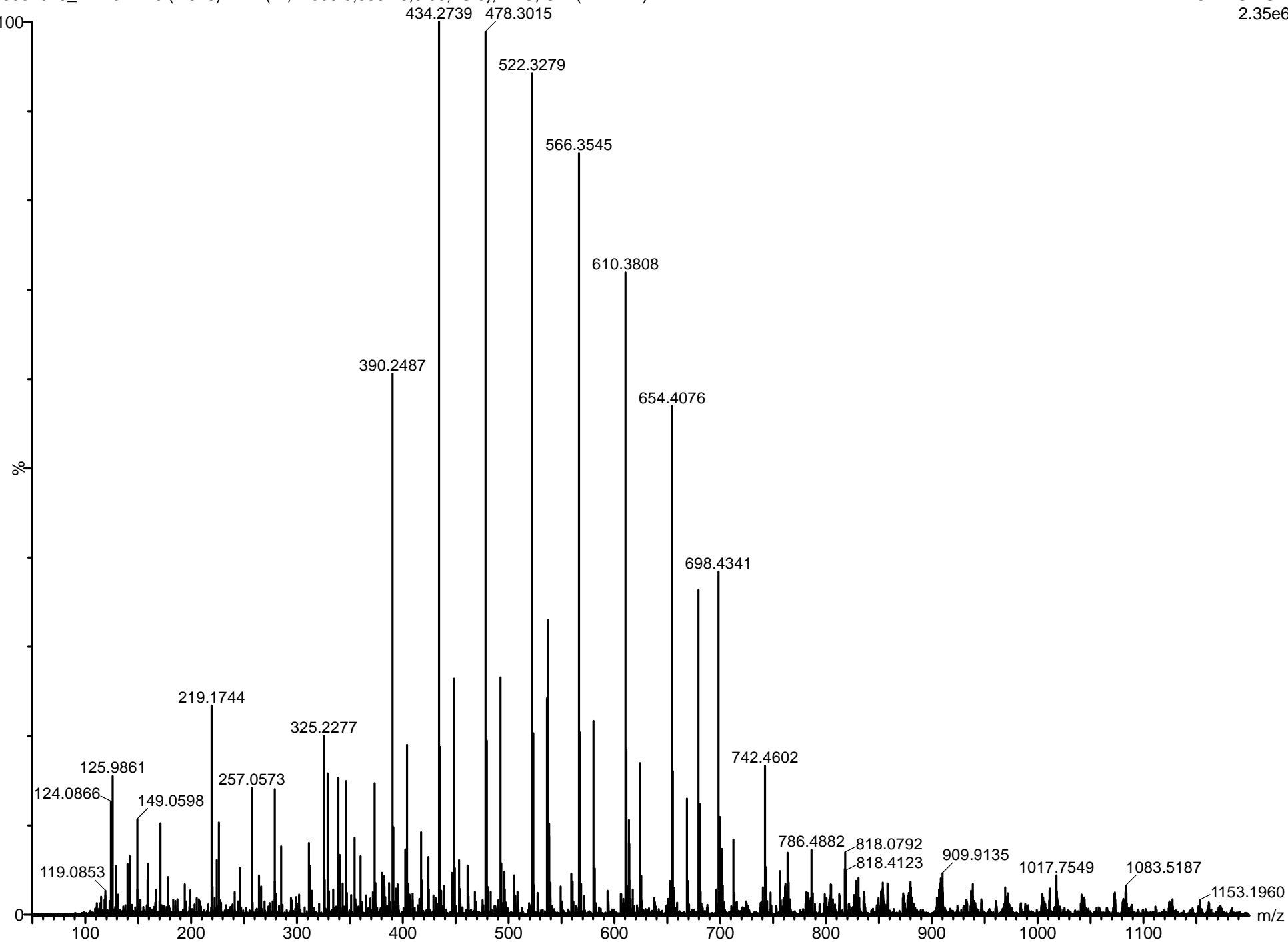

Supplement: S1 Data — Electrospray ionisation time of flight mass spectrometry (ESI-TOF MS, positive mode) spectra of the dengue cohort and ESI-TOF at different retention times. The spectra display the relative abundance (%) of detected ions across the m/z range. Prominent peaks corresponding to major ionised species are indicated. Variation in spectral profiles between retention times reflects the differences in compound composition and ionisation patterns within the sample. Data were acquired under identical instrumental conditions and are presented as representative scans. (ZIP) [file pntd.0014327.s003.zip › EM COMPLETE SAMPLES SPECTRUM/EM231 SPECTRUM RT 2.548.pdf]

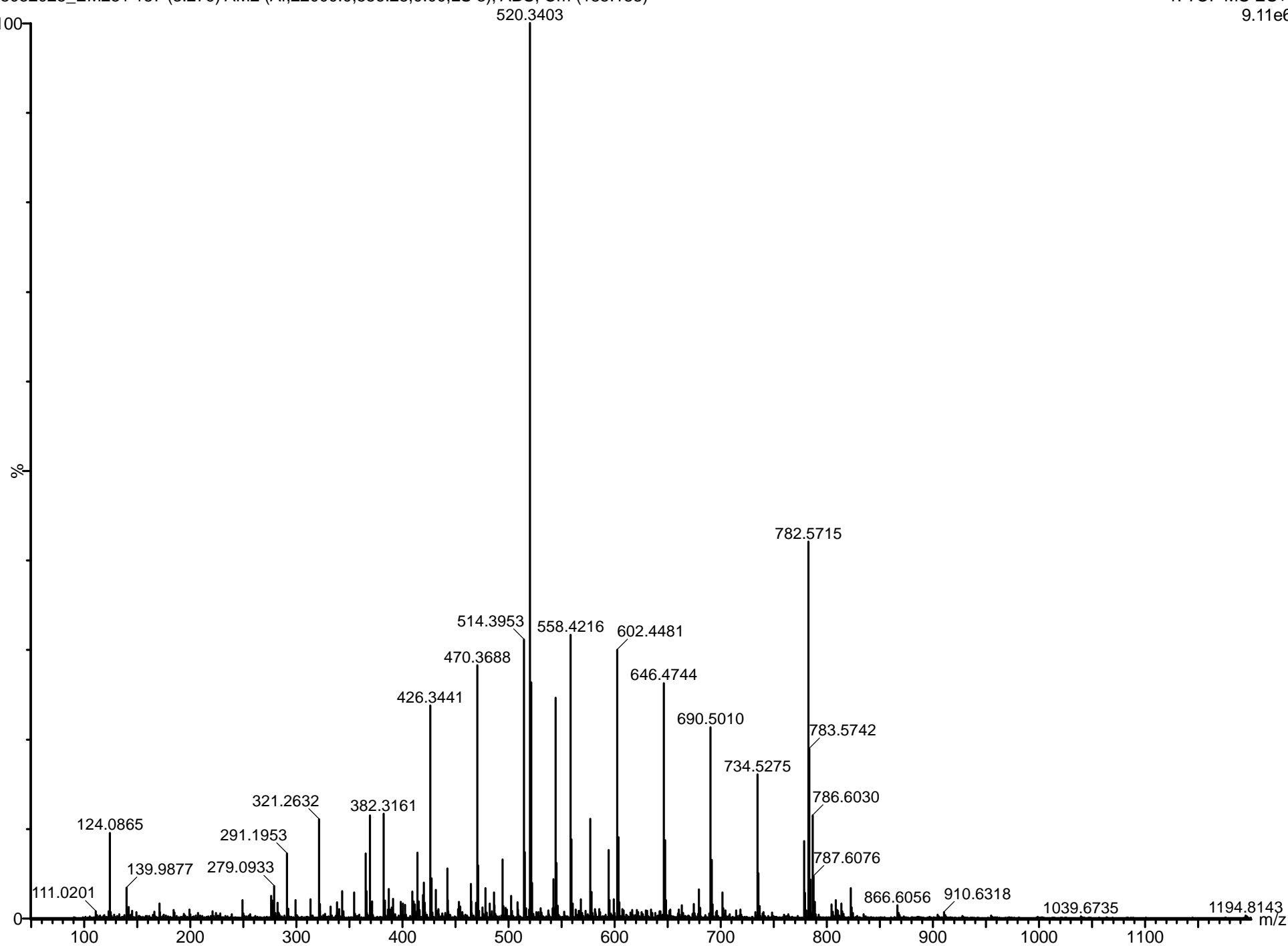

Supplement: S1 Data — Electrospray ionisation time of flight mass spectrometry (ESI-TOF MS, positive mode) spectra of the dengue cohort and ESI-TOF at different retention times. The spectra display the relative abundance (%) of detected ions across the m/z range. Prominent peaks corresponding to major ionised species are indicated. Variation in spectral profiles between retention times reflects the differences in compound composition and ionisation patterns within the sample. Data were acquired under identical instrumental conditions and are presented as representative scans. (ZIP) [file pntd.0014327.s003.zip › EM COMPLETE SAMPLES SPECTRUM/EM231 SPECTRUM RT 3.279.pdf]

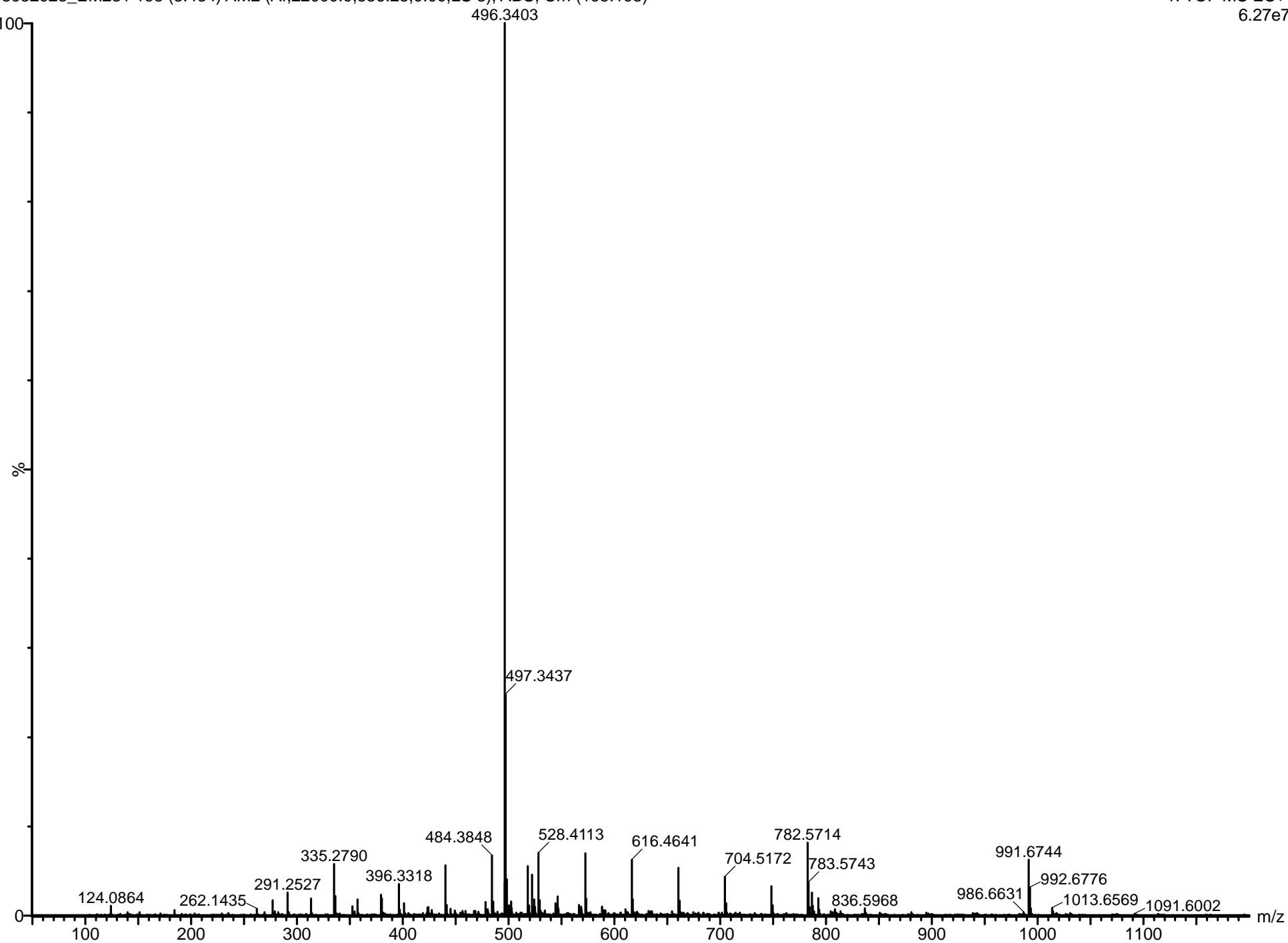

Supplement: S1 Data — Electrospray ionisation time of flight mass spectrometry (ESI-TOF MS, positive mode) spectra of the dengue cohort and ESI-TOF at different retention times. The spectra display the relative abundance (%) of detected ions across the m/z range. Prominent peaks corresponding to major ionised species are indicated. Variation in spectral profiles between retention times reflects the differences in compound composition and ionisation patterns within the sample. Data were acquired under identical instrumental conditions and are presented as representative scans. (ZIP) [file pntd.0014327.s003.zip › EM COMPLETE SAMPLES SPECTRUM/EM231 SPECTRUM RT 3.434.pdf]

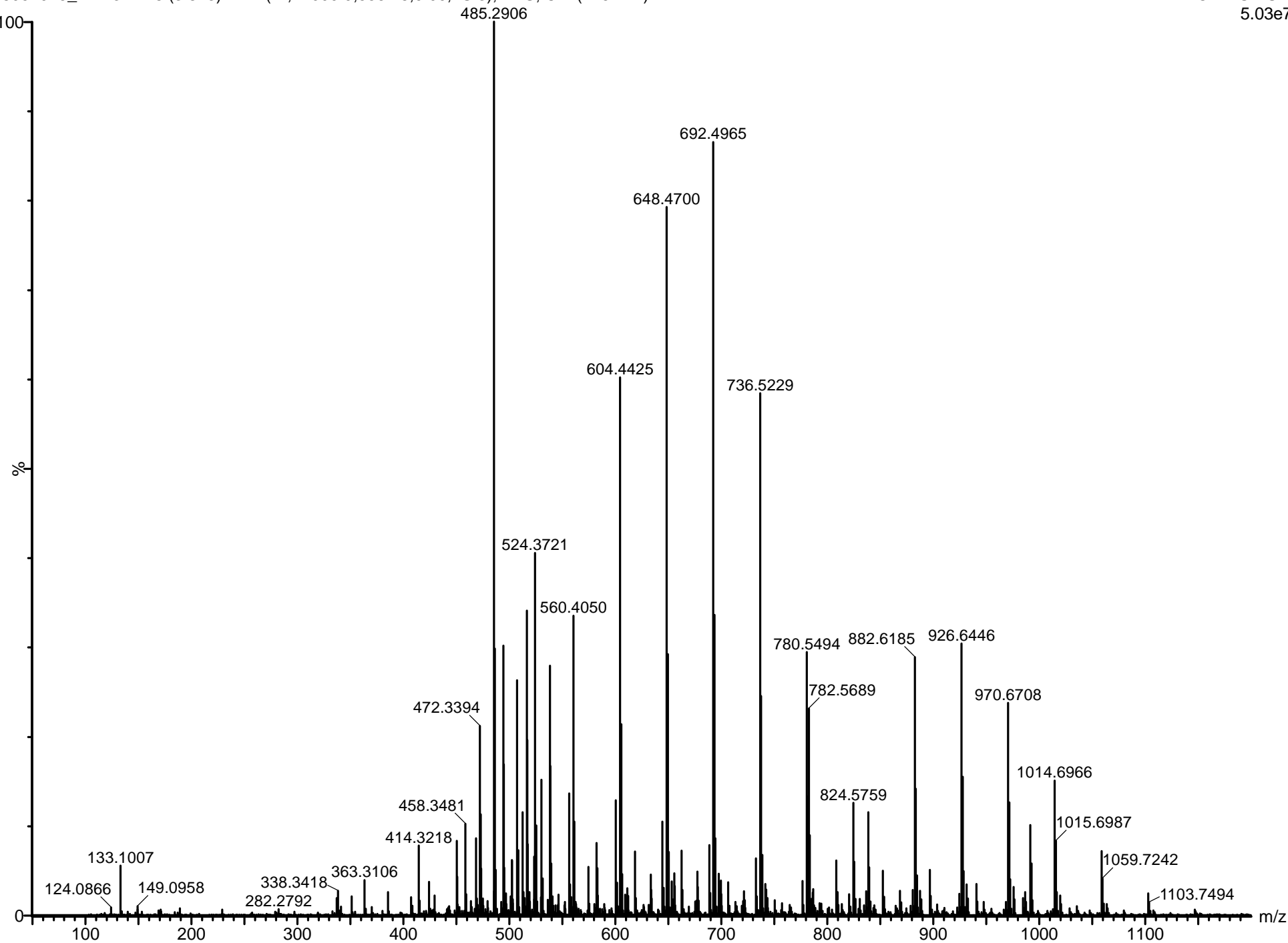

Supplement: S1 Data — Electrospray ionisation time of flight mass spectrometry (ESI-TOF MS, positive mode) spectra of the dengue cohort and ESI-TOF at different retention times. The spectra display the relative abundance (%) of detected ions across the m/z range. Prominent peaks corresponding to major ionised species are indicated. Variation in spectral profiles between retention times reflects the differences in compound composition and ionisation patterns within the sample. Data were acquired under identical instrumental conditions and are presented as representative scans. (ZIP) [file pntd.0014327.s003.zip › EM COMPLETE SAMPLES SPECTRUM/EM231 SPECTRUM RT 3.823.pdf]

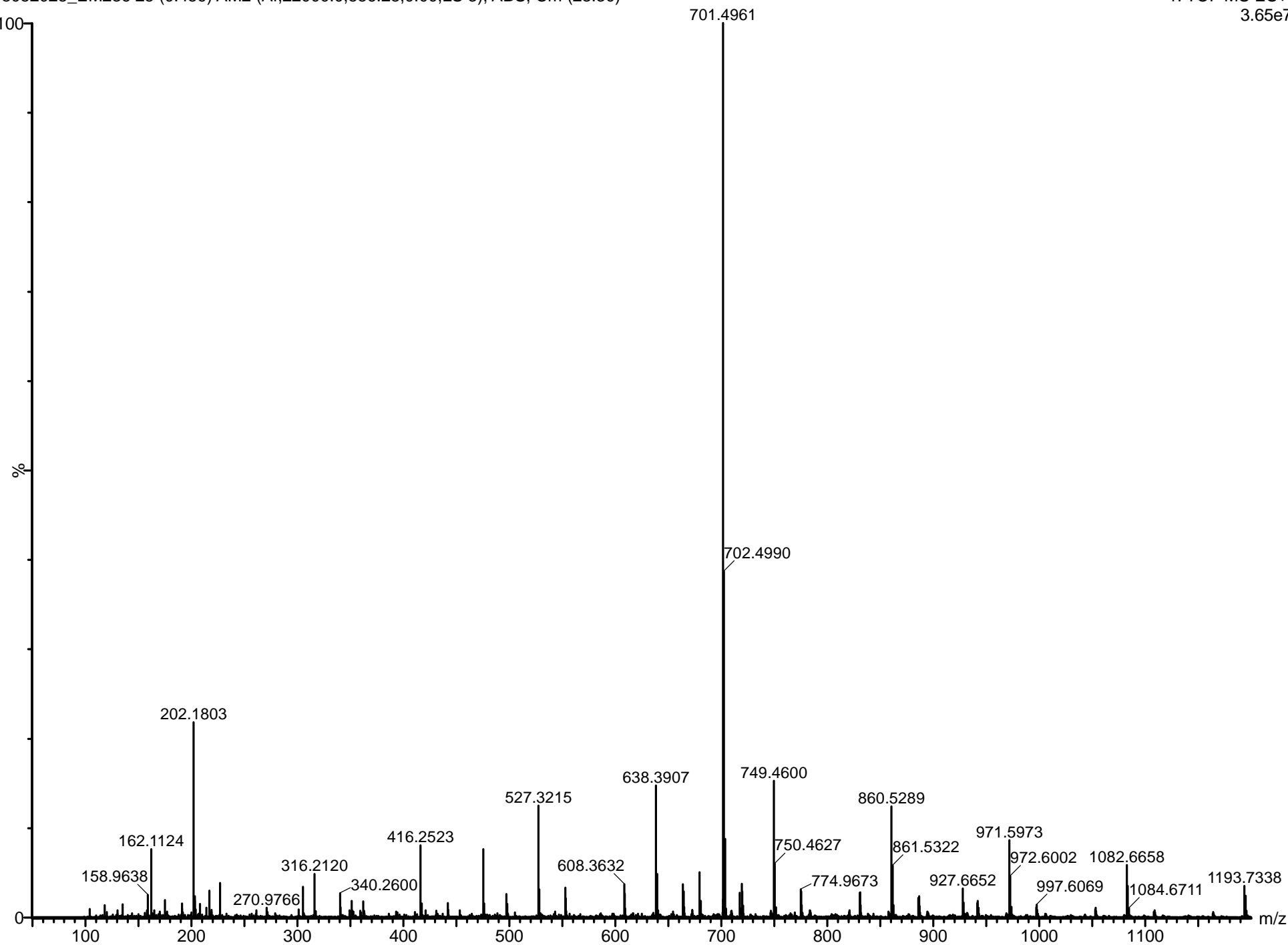

Supplement: S1 Data — Electrospray ionisation time of flight mass spectrometry (ESI-TOF MS, positive mode) spectra of the dengue cohort and ESI-TOF at different retention times. The spectra display the relative abundance (%) of detected ions across the m/z range. Prominent peaks corresponding to major ionised species are indicated. Variation in spectral profiles between retention times reflects the differences in compound composition and ionisation patterns within the sample. Data were acquired under identical instrumental conditions and are presented as representative scans. (ZIP) [file pntd.0014327.s003.zip › EM COMPLETE SAMPLES SPECTRUM/EM236 SPECTRUM RT 0.459.pdf]

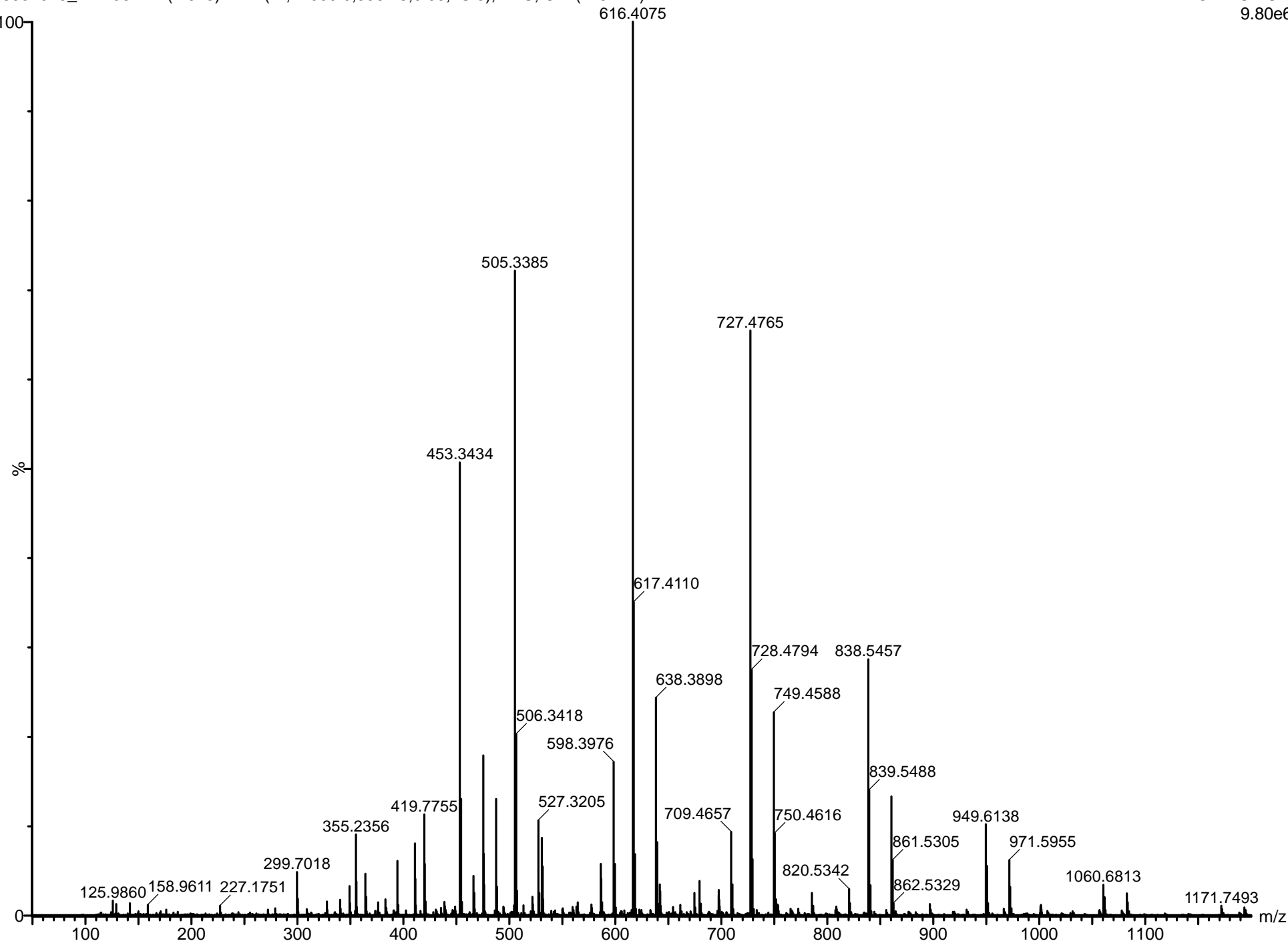

Supplement: S1 Data — Electrospray ionisation time of flight mass spectrometry (ESI-TOF MS, positive mode) spectra of the dengue cohort and ESI-TOF at different retention times. The spectra display the relative abundance (%) of detected ions across the m/z range. Prominent peaks corresponding to major ionised species are indicated. Variation in spectral profiles between retention times reflects the differences in compound composition and ionisation patterns within the sample. Data were acquired under identical instrumental conditions and are presented as representative scans. (ZIP) [file pntd.0014327.s003.zip › EM COMPLETE SAMPLES SPECTRUM/EM236 SPECTRUM RT 2.075.pdf]

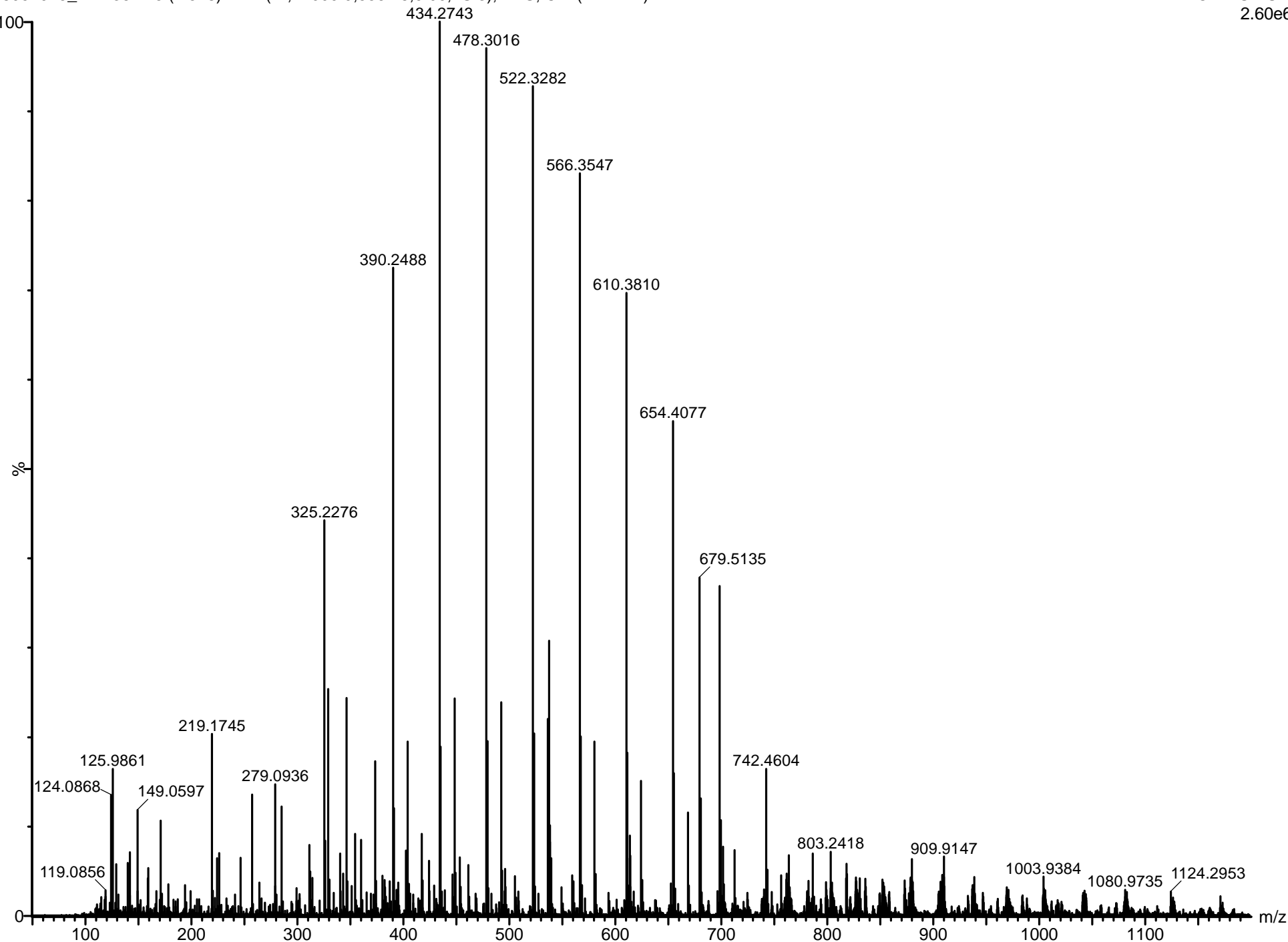

Supplement: S1 Data — Electrospray ionisation time of flight mass spectrometry (ESI-TOF MS, positive mode) spectra of the dengue cohort and ESI-TOF at different retention times. The spectra display the relative abundance (%) of detected ions across the m/z range. Prominent peaks corresponding to major ionised species are indicated. Variation in spectral profiles between retention times reflects the differences in compound composition and ionisation patterns within the sample. Data were acquired under identical instrumental conditions and are presented as representative scans. (ZIP) [file pntd.0014327.s003.zip › EM COMPLETE SAMPLES SPECTRUM/EM236 SPECTRUM RT 2.548.pdf]

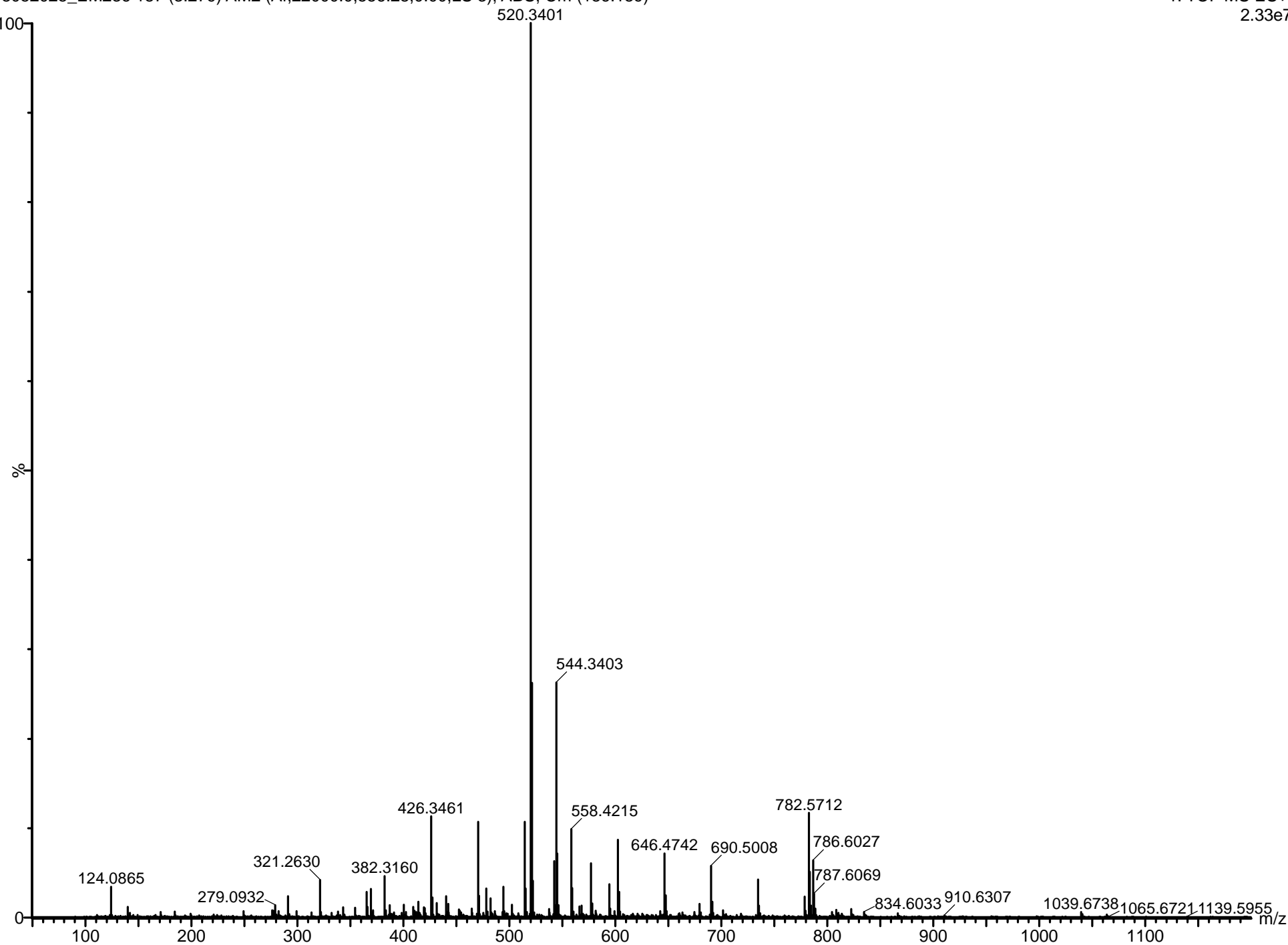

Supplement: S1 Data — Electrospray ionisation time of flight mass spectrometry (ESI-TOF MS, positive mode) spectra of the dengue cohort and ESI-TOF at different retention times. The spectra display the relative abundance (%) of detected ions across the m/z range. Prominent peaks corresponding to major ionised species are indicated. Variation in spectral profiles between retention times reflects the differences in compound composition and ionisation patterns within the sample. Data were acquired under identical instrumental conditions and are presented as representative scans. (ZIP) [file pntd.0014327.s003.zip › EM COMPLETE SAMPLES SPECTRUM/EM236 SPECTRUM RT 3.279.pdf]

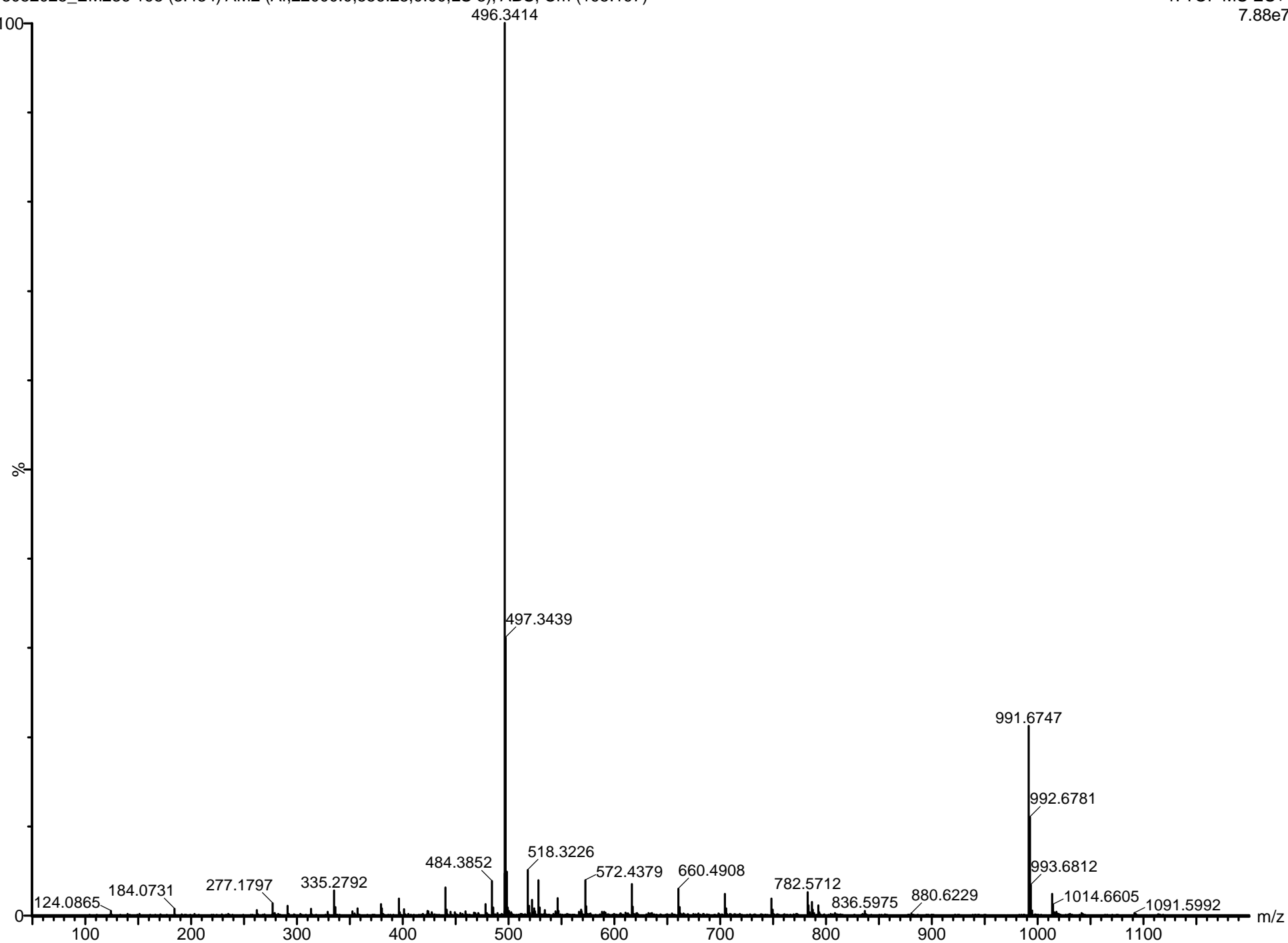

Supplement: S1 Data — Electrospray ionisation time of flight mass spectrometry (ESI-TOF MS, positive mode) spectra of the dengue cohort and ESI-TOF at different retention times. The spectra display the relative abundance (%) of detected ions across the m/z range. Prominent peaks corresponding to major ionised species are indicated. Variation in spectral profiles between retention times reflects the differences in compound composition and ionisation patterns within the sample. Data were acquired under identical instrumental conditions and are presented as representative scans. (ZIP) [file pntd.0014327.s003.zip › EM COMPLETE SAMPLES SPECTRUM/EM236 SPECTRUM RT 3.434.pdf]

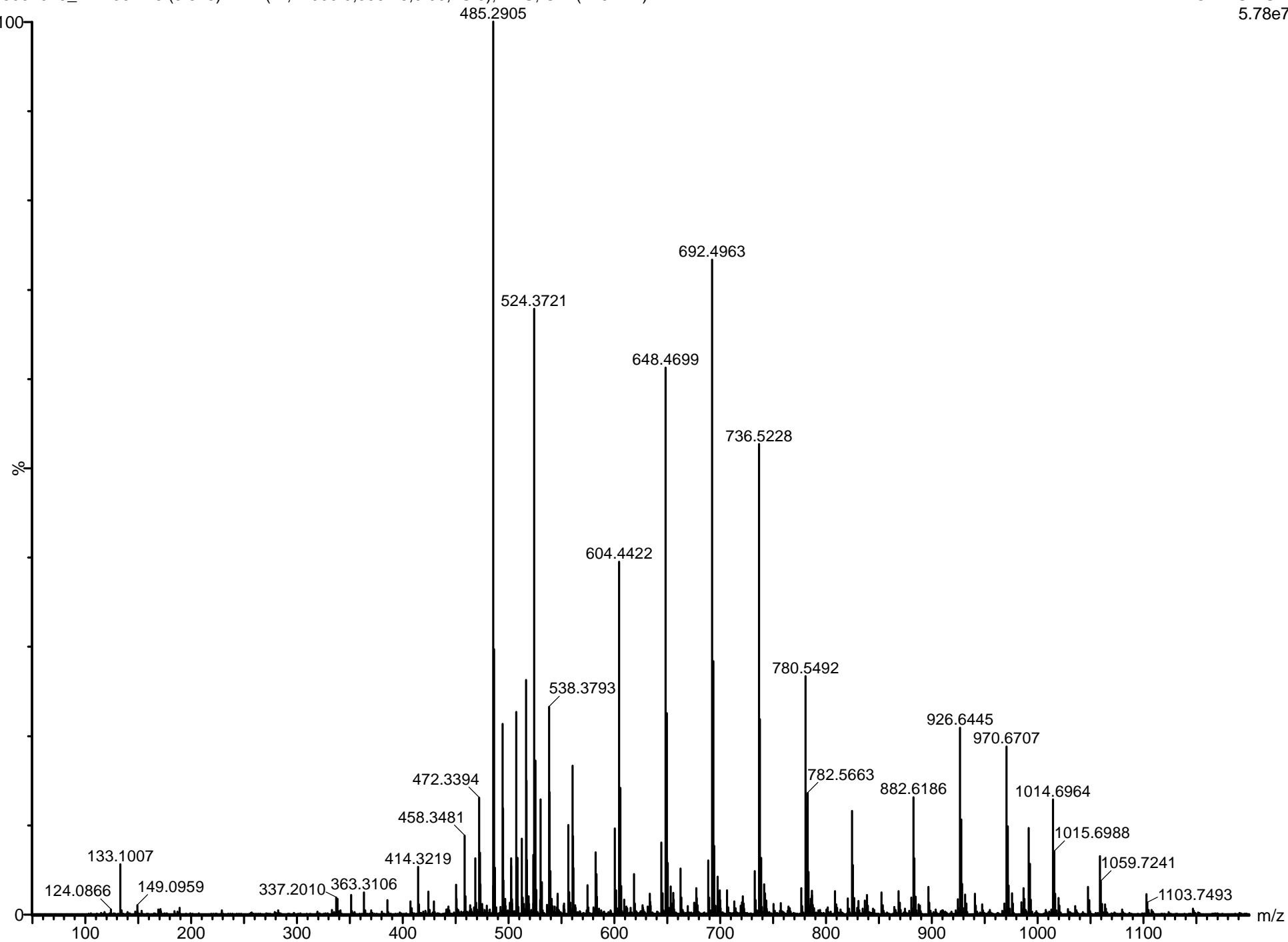

Supplement: S1 Data — Electrospray ionisation time of flight mass spectrometry (ESI-TOF MS, positive mode) spectra of the dengue cohort and ESI-TOF at different retention times. The spectra display the relative abundance (%) of detected ions across the m/z range. Prominent peaks corresponding to major ionised species are indicated. Variation in spectral profiles between retention times reflects the differences in compound composition and ionisation patterns within the sample. Data were acquired under identical instrumental conditions and are presented as representative scans. (ZIP) [file pntd.0014327.s003.zip › EM COMPLETE SAMPLES SPECTRUM/EM236 SPECTRUM RT 3.823.pdf]

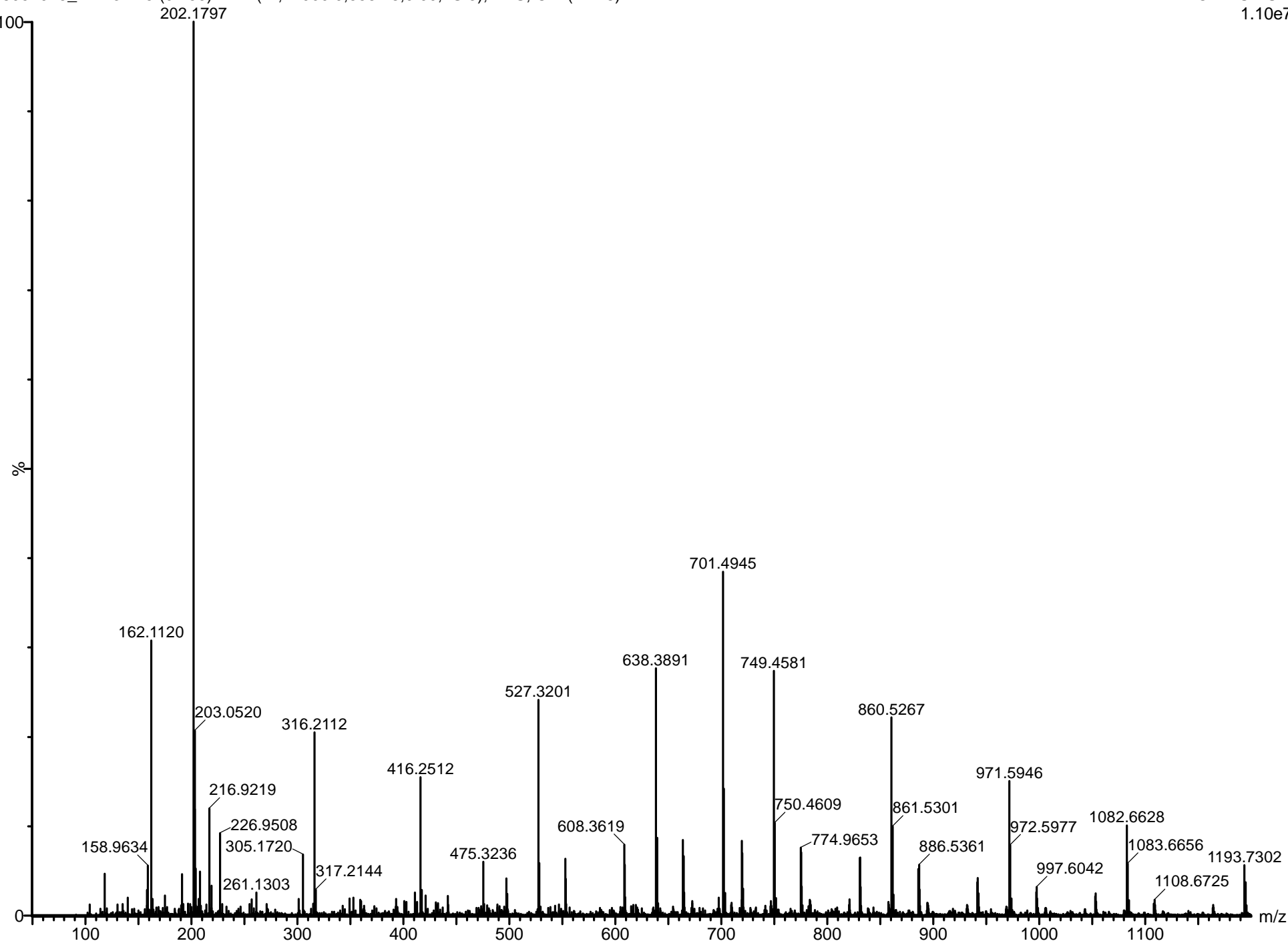

Supplement: S1 Data — Electrospray ionisation time of flight mass spectrometry (ESI-TOF MS, positive mode) spectra of the dengue cohort and ESI-TOF at different retention times. The spectra display the relative abundance (%) of detected ions across the m/z range. Prominent peaks corresponding to major ionised species are indicated. Variation in spectral profiles between retention times reflects the differences in compound composition and ionisation patterns within the sample. Data were acquired under identical instrumental conditions and are presented as representative scans. (ZIP) [file pntd.0014327.s003.zip › EM COMPLETE SAMPLES SPECTRUM/EM237 SPECTRUM RT 0.459.pdf]

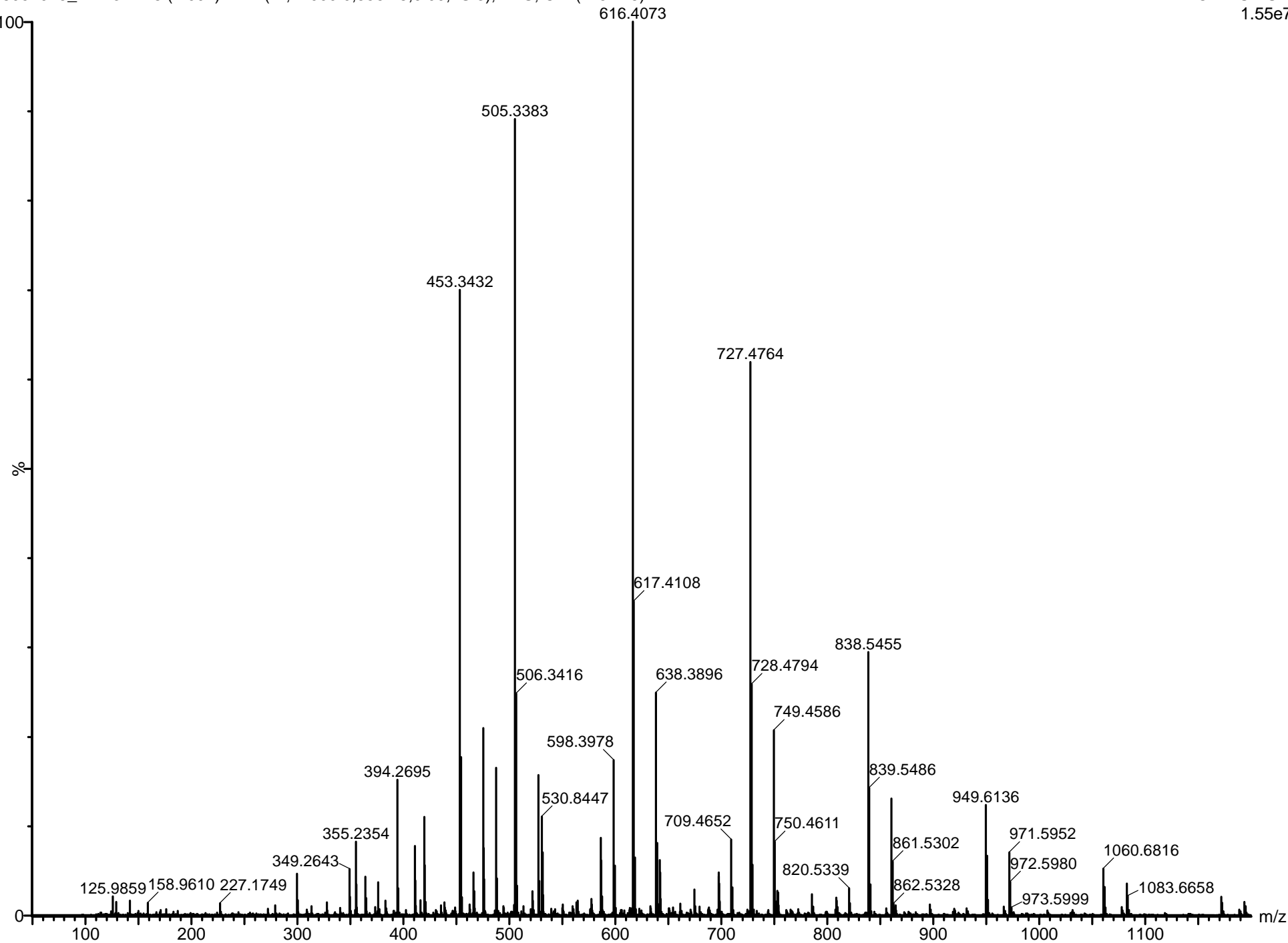

Supplement: S1 Data — Electrospray ionisation time of flight mass spectrometry (ESI-TOF MS, positive mode) spectra of the dengue cohort and ESI-TOF at different retention times. The spectra display the relative abundance (%) of detected ions across the m/z range. Prominent peaks corresponding to major ionised species are indicated. Variation in spectral profiles between retention times reflects the differences in compound composition and ionisation patterns within the sample. Data were acquired under identical instrumental conditions and are presented as representative scans. (ZIP) [file pntd.0014327.s003.zip › EM COMPLETE SAMPLES SPECTRUM/EM237 SPECTRUM RT 2.092.pdf]

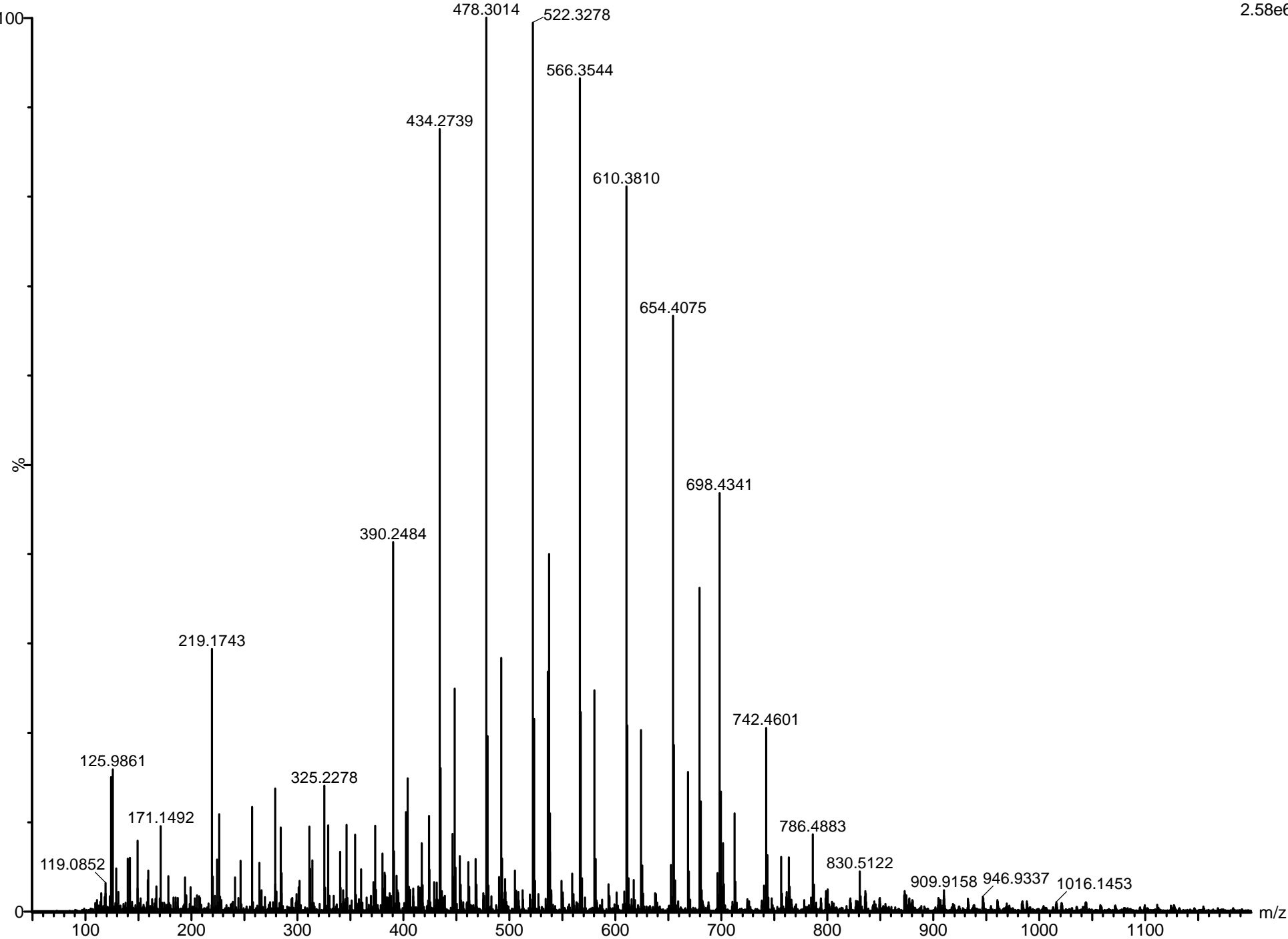

Supplement: S1 Data — Electrospray ionisation time of flight mass spectrometry (ESI-TOF MS, positive mode) spectra of the dengue cohort and ESI-TOF at different retention times. The spectra display the relative abundance (%) of detected ions across the m/z range. Prominent peaks corresponding to major ionised species are indicated. Variation in spectral profiles between retention times reflects the differences in compound composition and ionisation patterns within the sample. Data were acquired under identical instrumental conditions and are presented as representative scans. (ZIP) [file pntd.0014327.s003.zip › EM COMPLETE SAMPLES SPECTRUM/EM237 SPECTRUM RT 2.565.pdf]

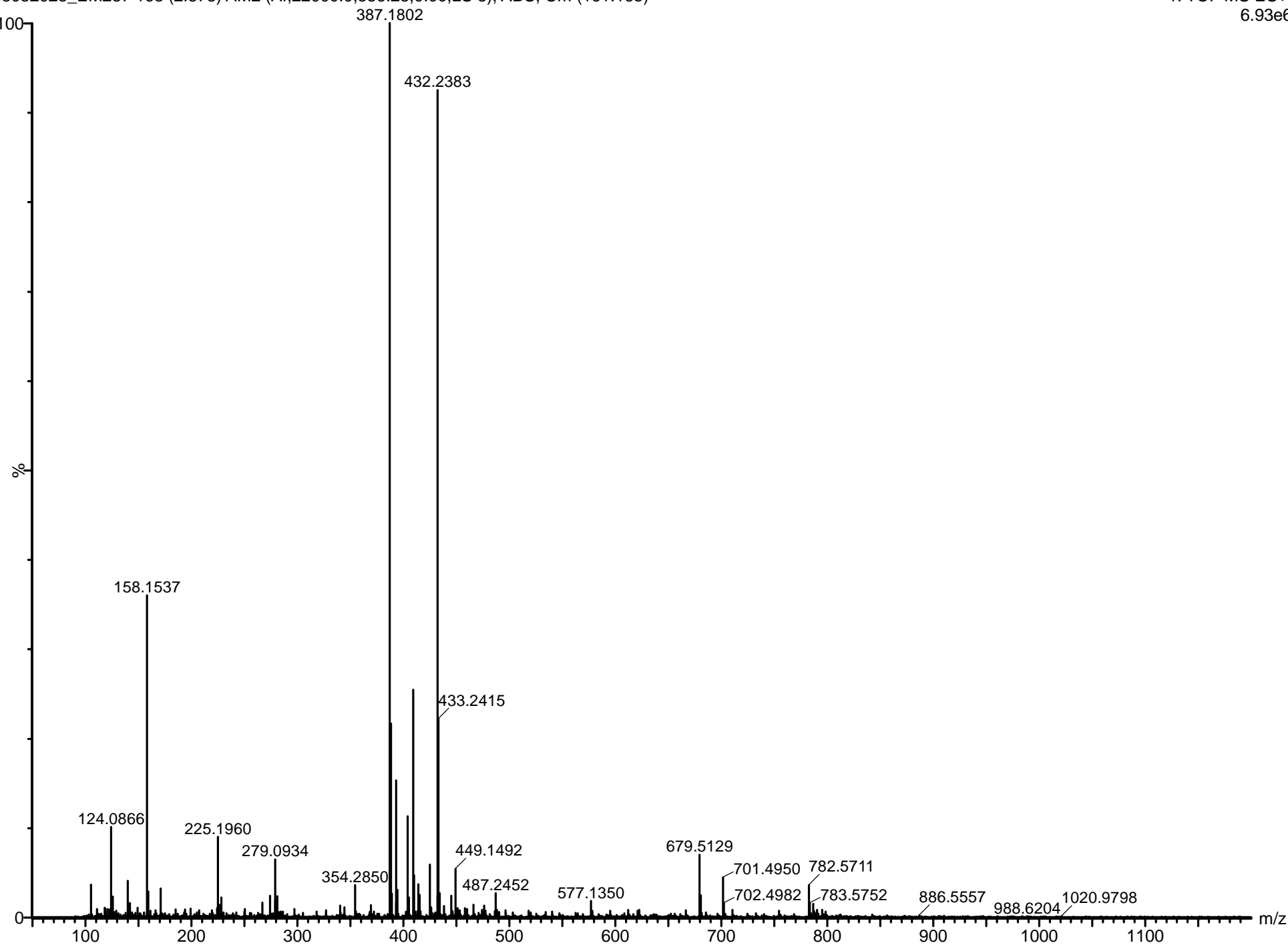

Supplement: S1 Data — Electrospray ionisation time of flight mass spectrometry (ESI-TOF MS, positive mode) spectra of the dengue cohort and ESI-TOF at different retention times. The spectra display the relative abundance (%) of detected ions across the m/z range. Prominent peaks corresponding to major ionised species are indicated. Variation in spectral profiles between retention times reflects the differences in compound composition and ionisation patterns within the sample. Data were acquired under identical instrumental conditions and are presented as representative scans. (ZIP) [file pntd.0014327.s003.zip › EM COMPLETE SAMPLES SPECTRUM/EM237 SPECTRUM RT 2.873.pdf]

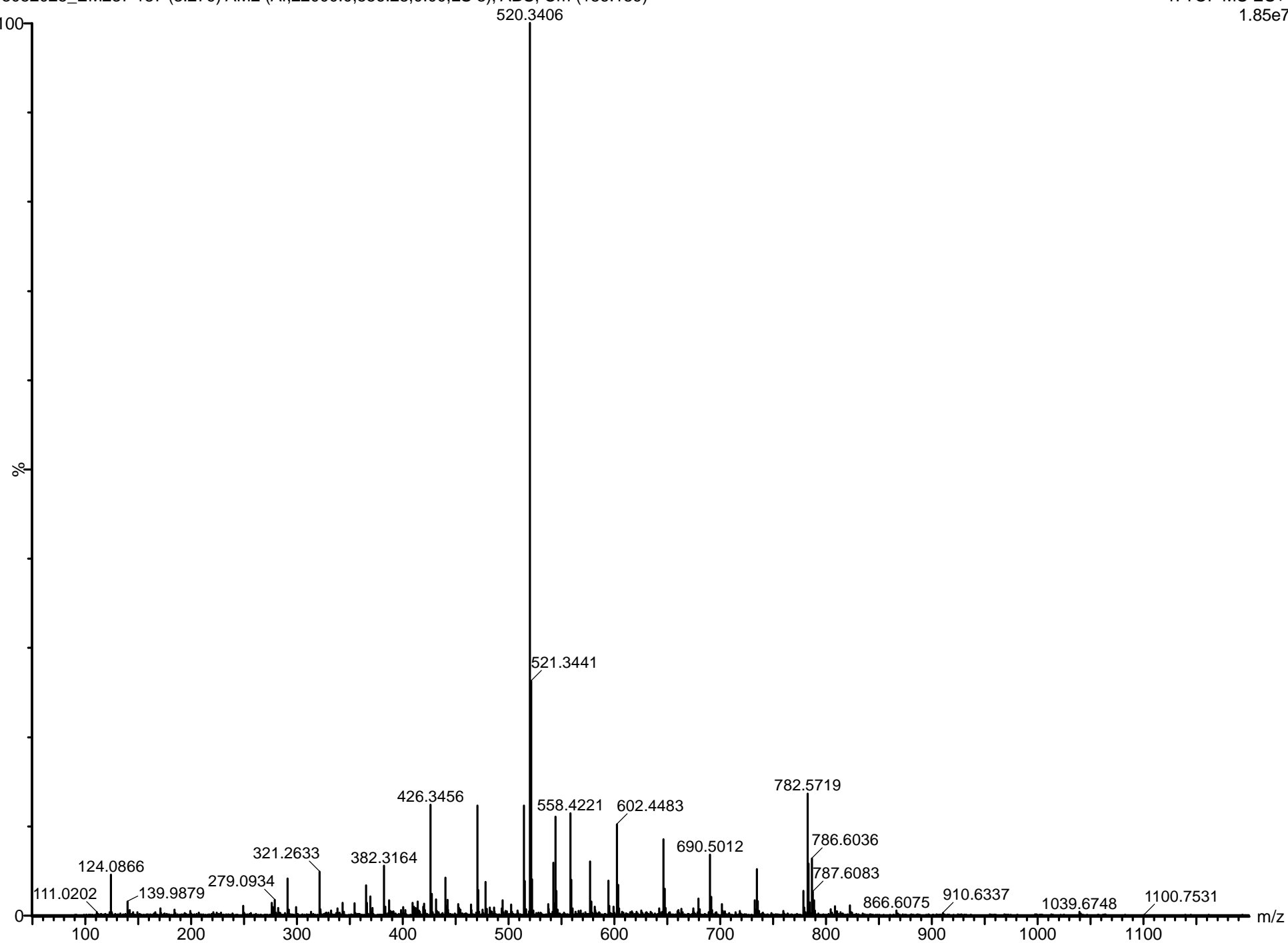

Supplement: S1 Data — Electrospray ionisation time of flight mass spectrometry (ESI-TOF MS, positive mode) spectra of the dengue cohort and ESI-TOF at different retention times. The spectra display the relative abundance (%) of detected ions across the m/z range. Prominent peaks corresponding to major ionised species are indicated. Variation in spectral profiles between retention times reflects the differences in compound composition and ionisation patterns within the sample. Data were acquired under identical instrumental conditions and are presented as representative scans. (ZIP) [file pntd.0014327.s003.zip › EM COMPLETE SAMPLES SPECTRUM/EM237 SPECTRUM RT 3.279.pdf]

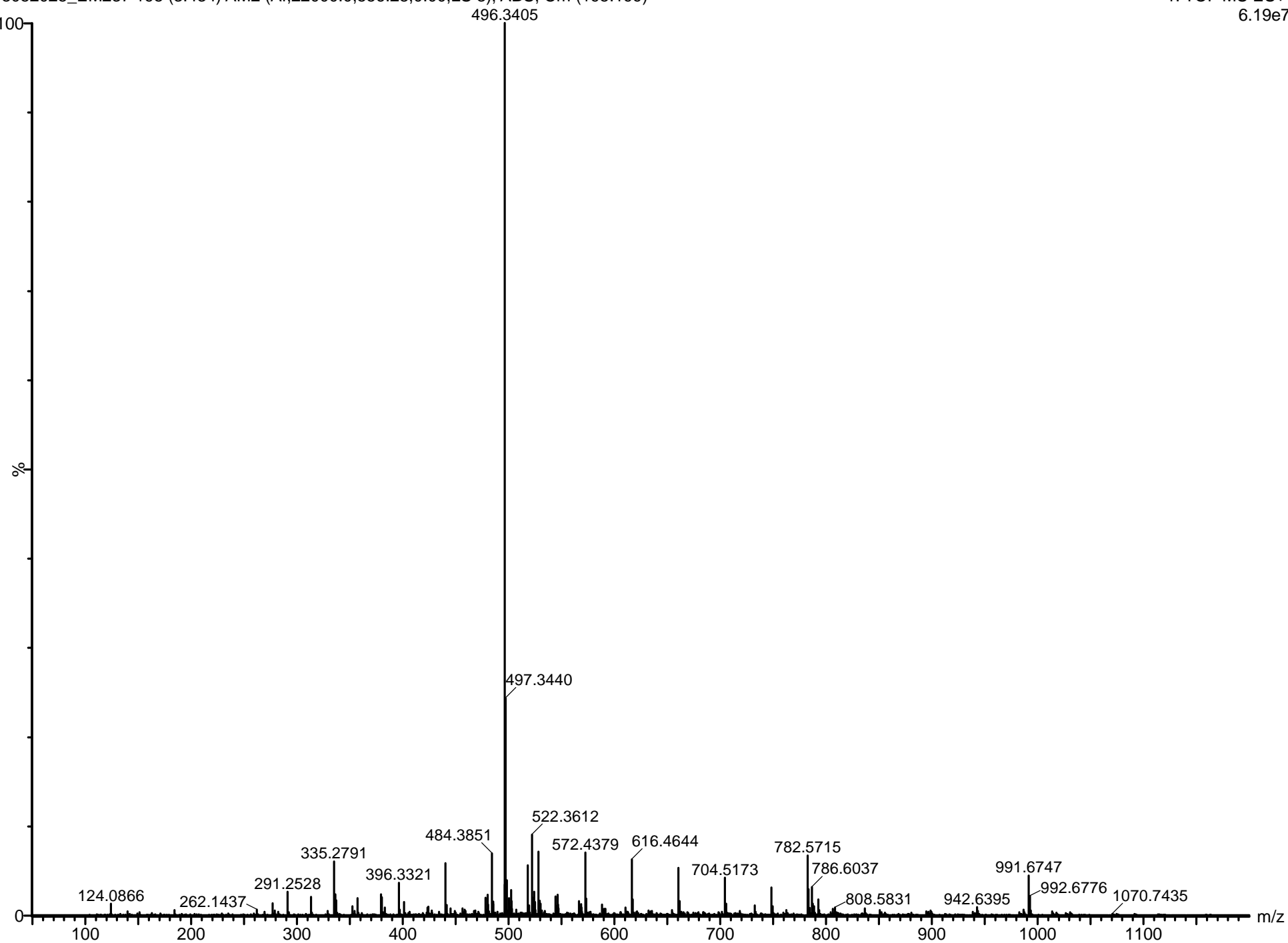

Supplement: S1 Data — Electrospray ionisation time of flight mass spectrometry (ESI-TOF MS, positive mode) spectra of the dengue cohort and ESI-TOF at different retention times. The spectra display the relative abundance (%) of detected ions across the m/z range. Prominent peaks corresponding to major ionised species are indicated. Variation in spectral profiles between retention times reflects the differences in compound composition and ionisation patterns within the sample. Data were acquired under identical instrumental conditions and are presented as representative scans. (ZIP) [file pntd.0014327.s003.zip › EM COMPLETE SAMPLES SPECTRUM/EM237 SPECTRUM RT 3.434.pdf]

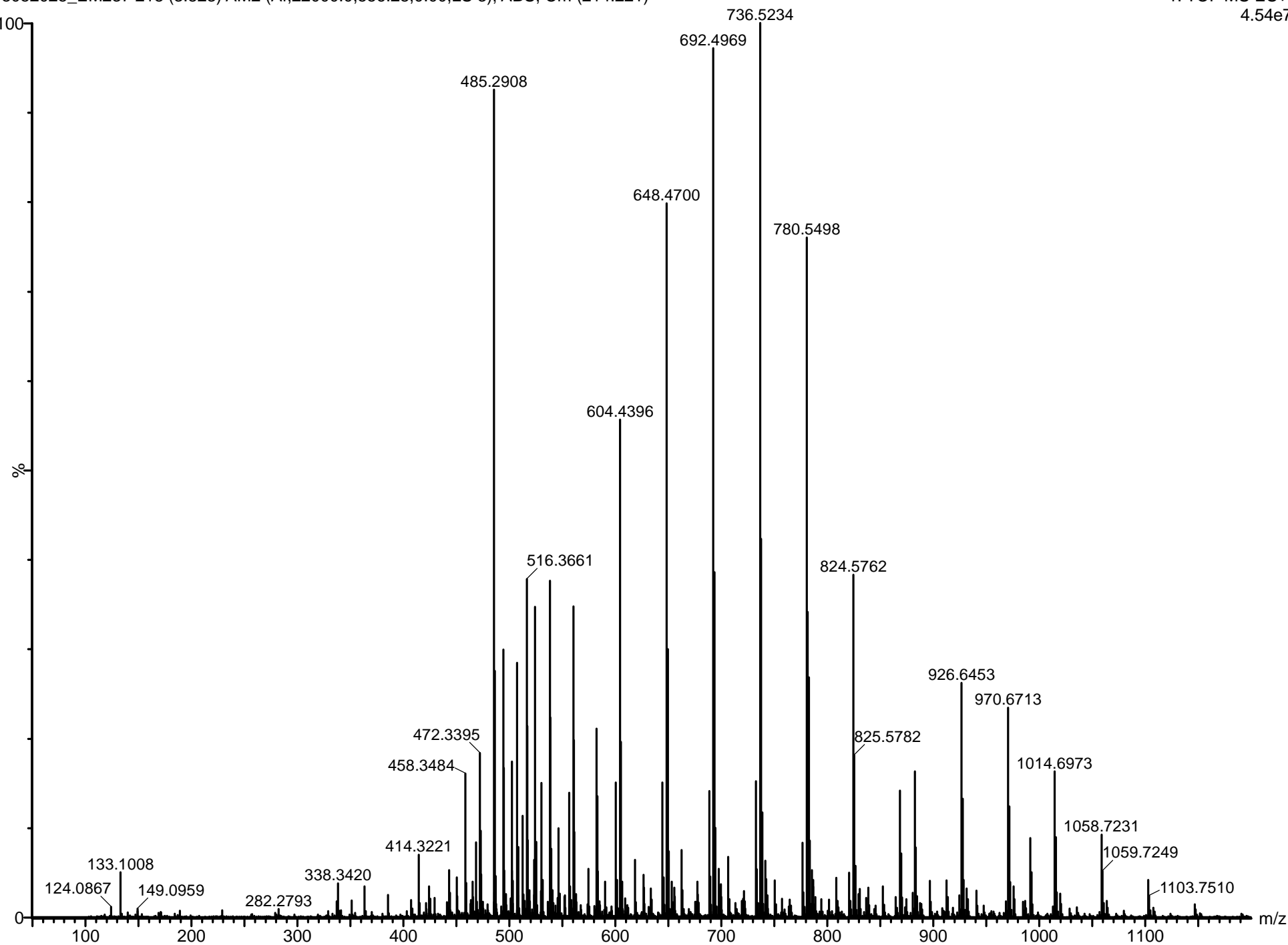

Supplement: S1 Data — Electrospray ionisation time of flight mass spectrometry (ESI-TOF MS, positive mode) spectra of the dengue cohort and ESI-TOF at different retention times. The spectra display the relative abundance (%) of detected ions across the m/z range. Prominent peaks corresponding to major ionised species are indicated. Variation in spectral profiles between retention times reflects the differences in compound composition and ionisation patterns within the sample. Data were acquired under identical instrumental conditions and are presented as representative scans. (ZIP) [file pntd.0014327.s003.zip › EM COMPLETE SAMPLES SPECTRUM/EM237 SPECTRUM RT 3.823.pdf]

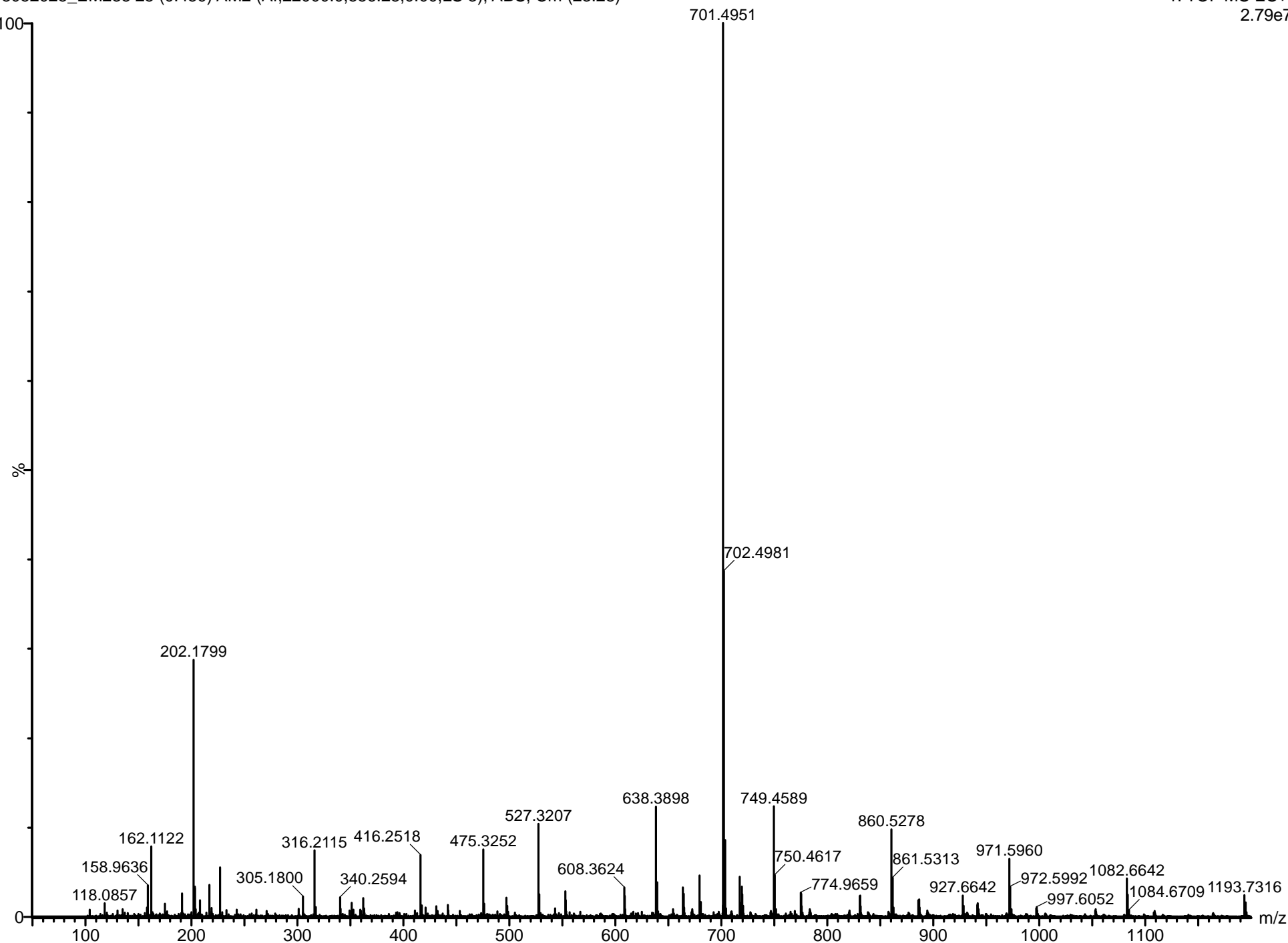

Supplement: S1 Data — Electrospray ionisation time of flight mass spectrometry (ESI-TOF MS, positive mode) spectra of the dengue cohort and ESI-TOF at different retention times. The spectra display the relative abundance (%) of detected ions across the m/z range. Prominent peaks corresponding to major ionised species are indicated. Variation in spectral profiles between retention times reflects the differences in compound composition and ionisation patterns within the sample. Data were acquired under identical instrumental conditions and are presented as representative scans. (ZIP) [file pntd.0014327.s003.zip › EM COMPLETE SAMPLES SPECTRUM/EM238 SPECTRUM RT 0.459.pdf]

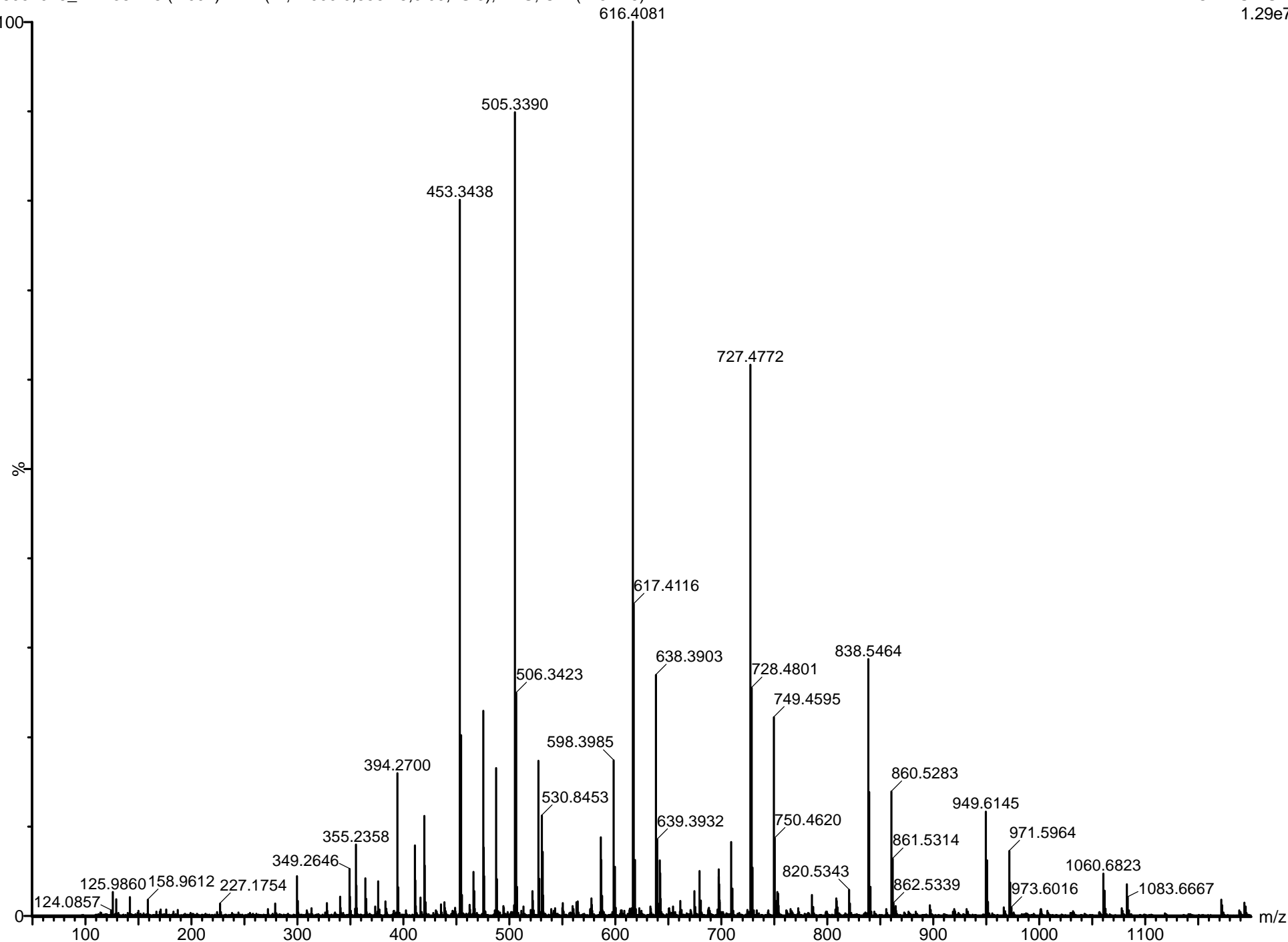

Supplement: S1 Data — Electrospray ionisation time of flight mass spectrometry (ESI-TOF MS, positive mode) spectra of the dengue cohort and ESI-TOF at different retention times. The spectra display the relative abundance (%) of detected ions across the m/z range. Prominent peaks corresponding to major ionised species are indicated. Variation in spectral profiles between retention times reflects the differences in compound composition and ionisation patterns within the sample. Data were acquired under identical instrumental conditions and are presented as representative scans. (ZIP) [file pntd.0014327.s003.zip › EM COMPLETE SAMPLES SPECTRUM/EM238 SPECTRUM RT 2.092.pdf]

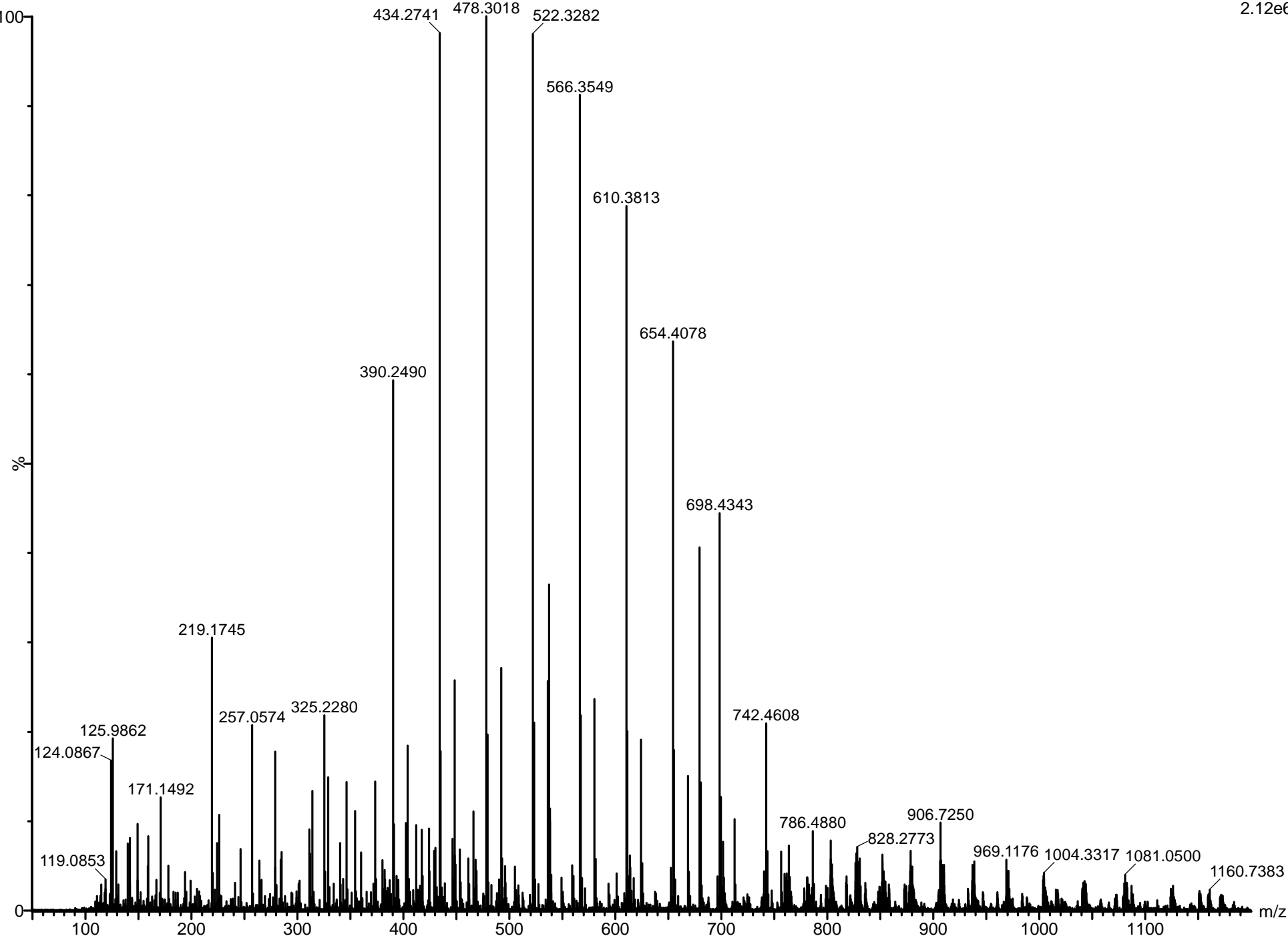

Supplement: S1 Data — Electrospray ionisation time of flight mass spectrometry (ESI-TOF MS, positive mode) spectra of the dengue cohort and ESI-TOF at different retention times. The spectra display the relative abundance (%) of detected ions across the m/z range. Prominent peaks corresponding to major ionised species are indicated. Variation in spectral profiles between retention times reflects the differences in compound composition and ionisation patterns within the sample. Data were acquired under identical instrumental conditions and are presented as representative scans. (ZIP) [file pntd.0014327.s003.zip › EM COMPLETE SAMPLES SPECTRUM/EM238 SPECTRUM RT 2.548.pdf]

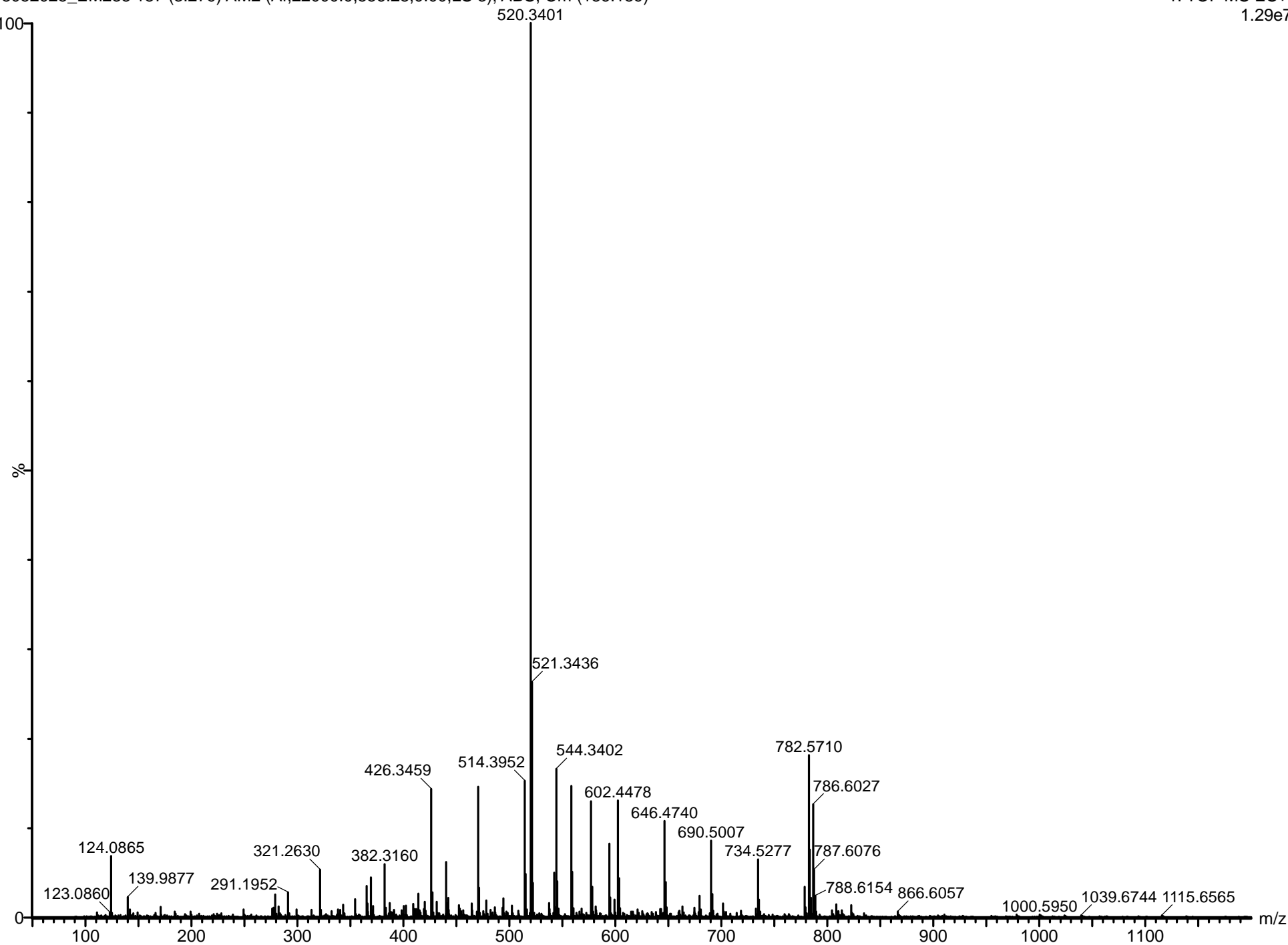

Supplement: S1 Data — Electrospray ionisation time of flight mass spectrometry (ESI-TOF MS, positive mode) spectra of the dengue cohort and ESI-TOF at different retention times. The spectra display the relative abundance (%) of detected ions across the m/z range. Prominent peaks corresponding to major ionised species are indicated. Variation in spectral profiles between retention times reflects the differences in compound composition and ionisation patterns within the sample. Data were acquired under identical instrumental conditions and are presented as representative scans. (ZIP) [file pntd.0014327.s003.zip › EM COMPLETE SAMPLES SPECTRUM/EM238 SPECTRUM RT 3.279.pdf]

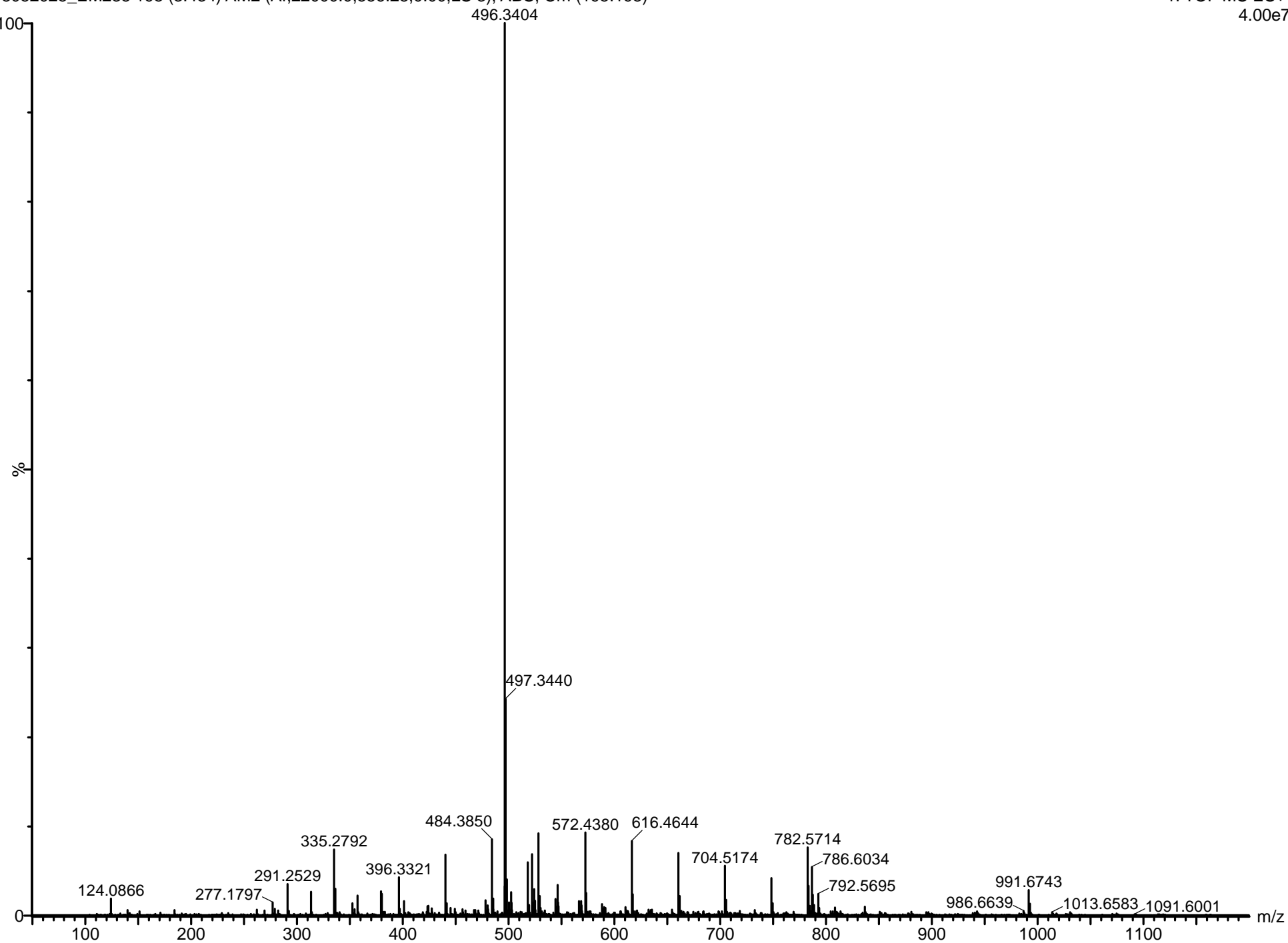

Supplement: S1 Data — Electrospray ionisation time of flight mass spectrometry (ESI-TOF MS, positive mode) spectra of the dengue cohort and ESI-TOF at different retention times. The spectra display the relative abundance (%) of detected ions across the m/z range. Prominent peaks corresponding to major ionised species are indicated. Variation in spectral profiles between retention times reflects the differences in compound composition and ionisation patterns within the sample. Data were acquired under identical instrumental conditions and are presented as representative scans. (ZIP) [file pntd.0014327.s003.zip › EM COMPLETE SAMPLES SPECTRUM/EM238 SPECTRUM RT 3.434.pdf]

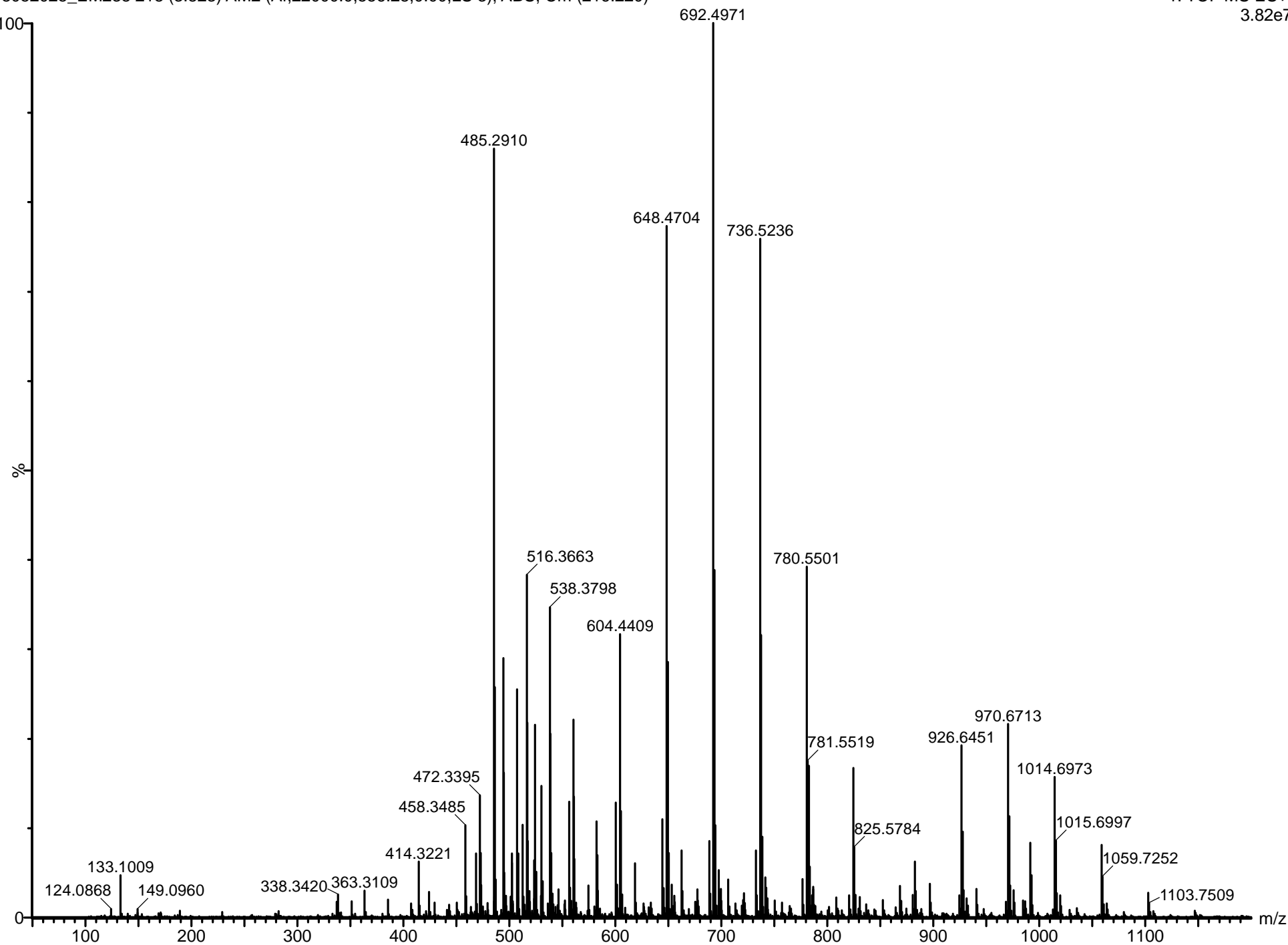

Supplement: S1 Data — Electrospray ionisation time of flight mass spectrometry (ESI-TOF MS, positive mode) spectra of the dengue cohort and ESI-TOF at different retention times. The spectra display the relative abundance (%) of detected ions across the m/z range. Prominent peaks corresponding to major ionised species are indicated. Variation in spectral profiles between retention times reflects the differences in compound composition and ionisation patterns within the sample. Data were acquired under identical instrumental conditions and are presented as representative scans. (ZIP) [file pntd.0014327.s003.zip › EM COMPLETE SAMPLES SPECTRUM/EM238 SPECTRUM RT 3.823.pdf]

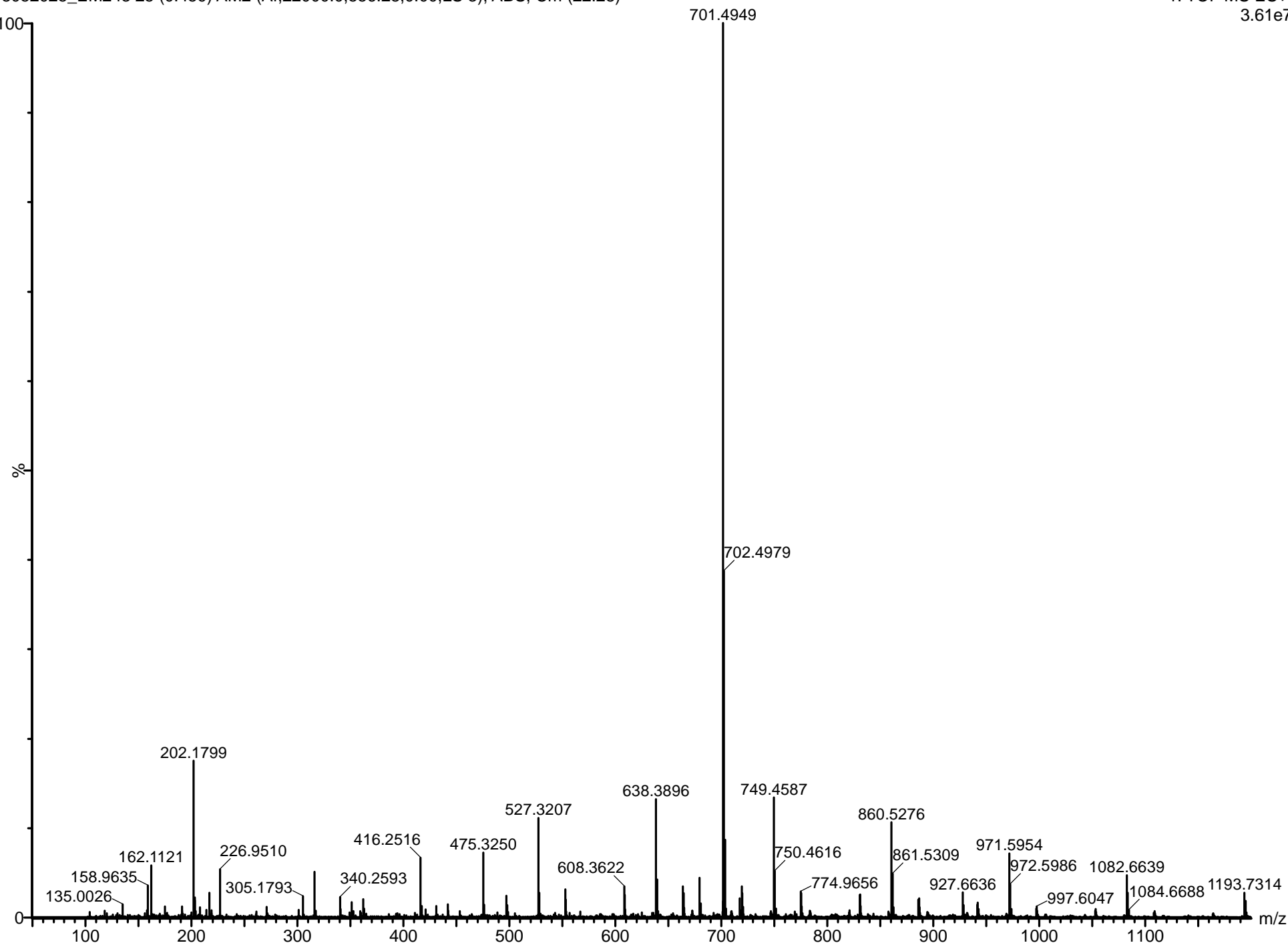

Supplement: S1 Data — Electrospray ionisation time of flight mass spectrometry (ESI-TOF MS, positive mode) spectra of the dengue cohort and ESI-TOF at different retention times. The spectra display the relative abundance (%) of detected ions across the m/z range. Prominent peaks corresponding to major ionised species are indicated. Variation in spectral profiles between retention times reflects the differences in compound composition and ionisation patterns within the sample. Data were acquired under identical instrumental conditions and are presented as representative scans. (ZIP) [file pntd.0014327.s003.zip › EM COMPLETE SAMPLES SPECTRUM/EM243 SPECTRUM RT 0.459.pdf]

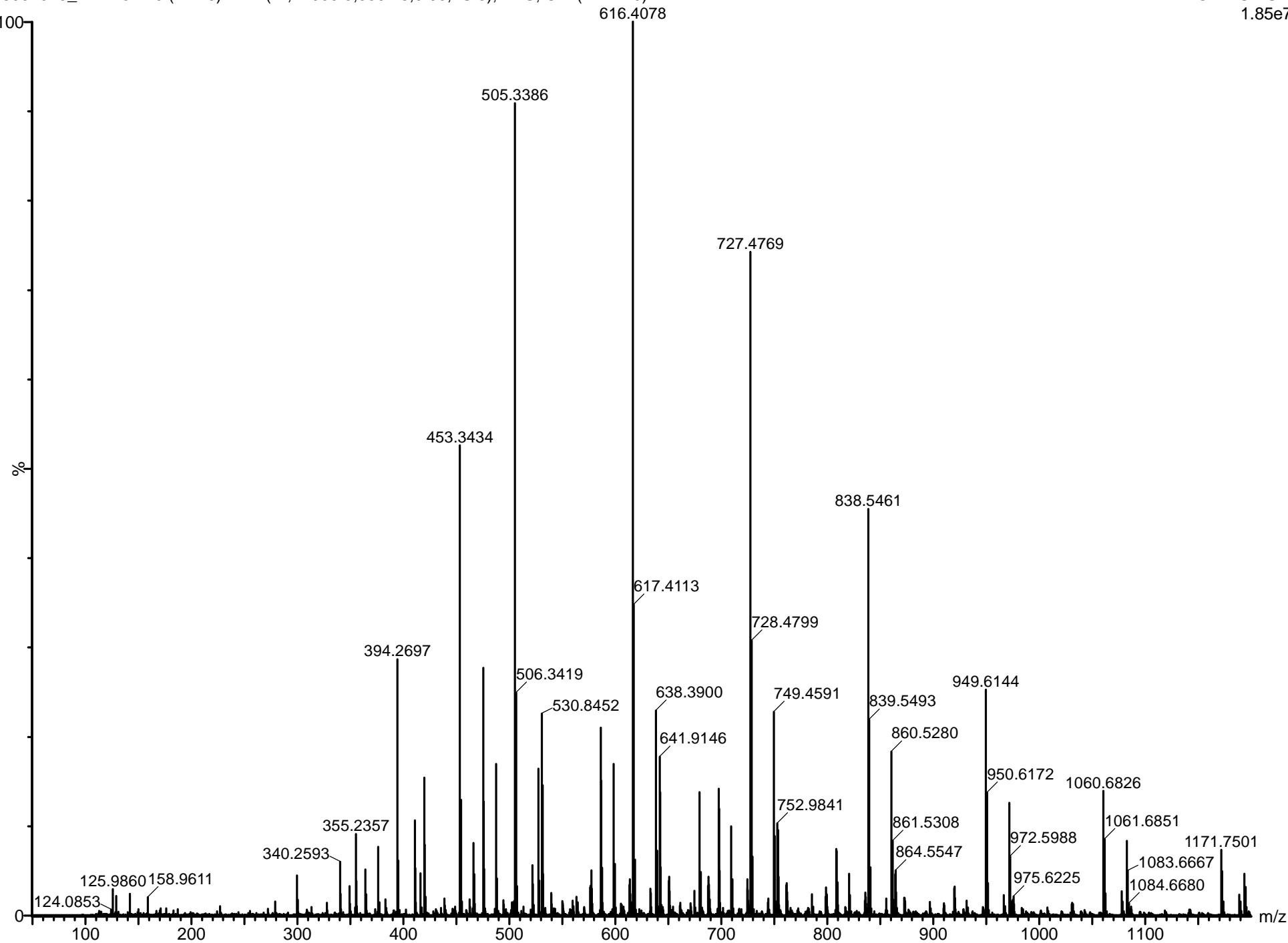

Supplement: S1 Data — Electrospray ionisation time of flight mass spectrometry (ESI-TOF MS, positive mode) spectra of the dengue cohort and ESI-TOF at different retention times. The spectra display the relative abundance (%) of detected ions across the m/z range. Prominent peaks corresponding to major ionised species are indicated. Variation in spectral profiles between retention times reflects the differences in compound composition and ionisation patterns within the sample. Data were acquired under identical instrumental conditions and are presented as representative scans. (ZIP) [file pntd.0014327.s003.zip › EM COMPLETE SAMPLES SPECTRUM/EM243 SPECTRUM RT 2.126.pdf]

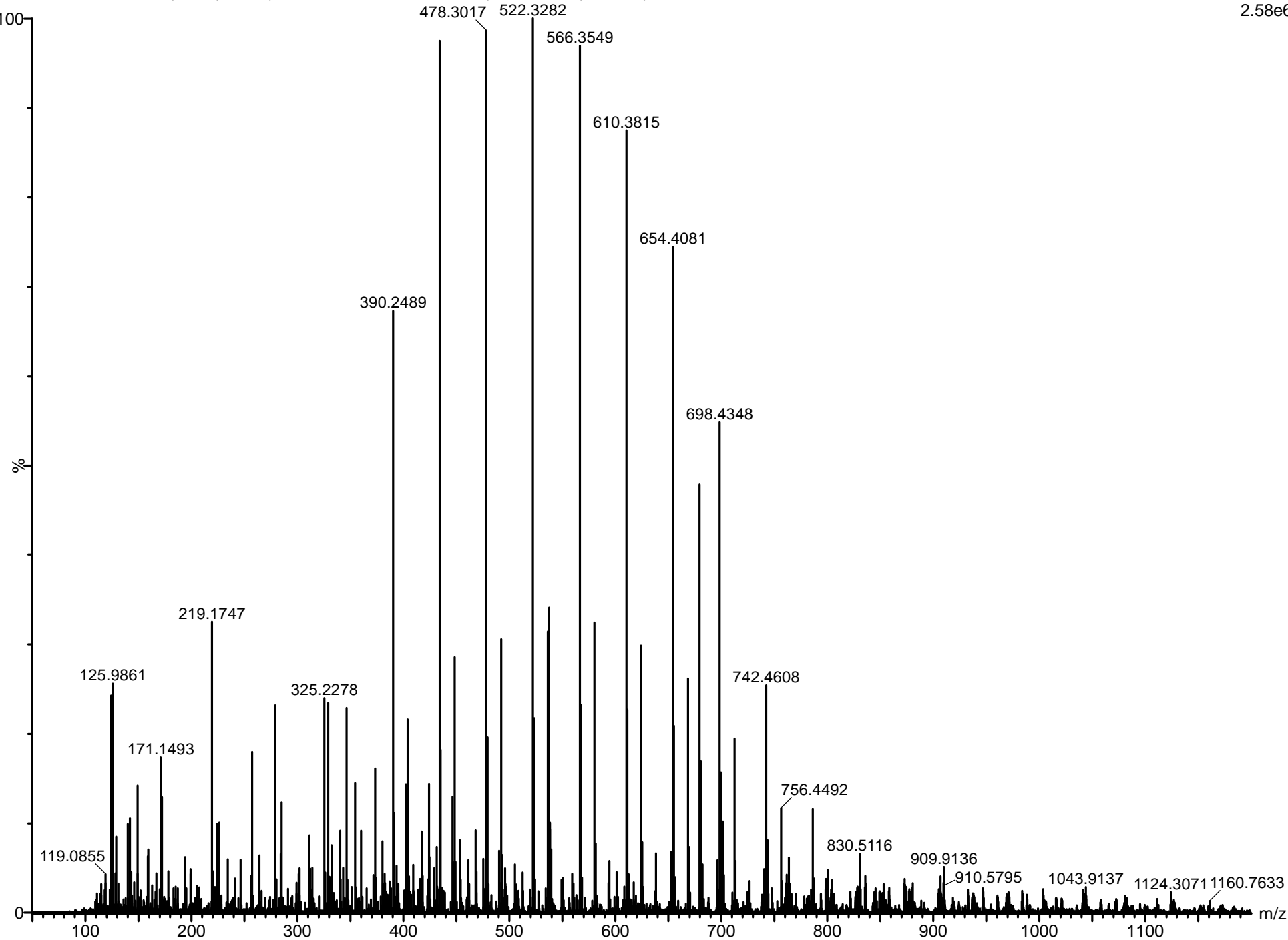

Supplement: S1 Data — Electrospray ionisation time of flight mass spectrometry (ESI-TOF MS, positive mode) spectra of the dengue cohort and ESI-TOF at different retention times. The spectra display the relative abundance (%) of detected ions across the m/z range. Prominent peaks corresponding to major ionised species are indicated. Variation in spectral profiles between retention times reflects the differences in compound composition and ionisation patterns within the sample. Data were acquired under identical instrumental conditions and are presented as representative scans. (ZIP) [file pntd.0014327.s003.zip › EM COMPLETE SAMPLES SPECTRUM/EM243 SPECTRUM RT 2.548.pdf]

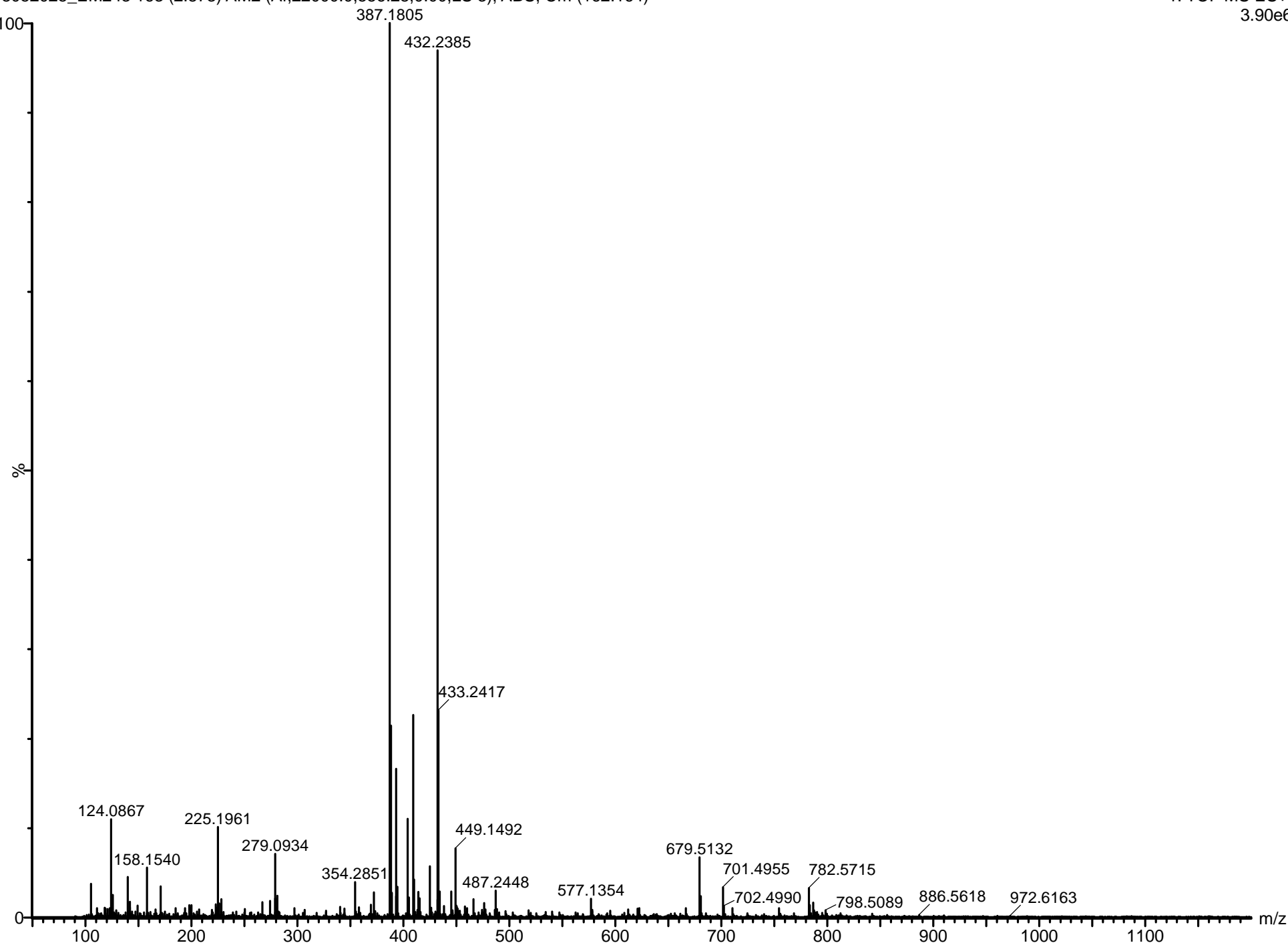

Supplement: S1 Data — Electrospray ionisation time of flight mass spectrometry (ESI-TOF MS, positive mode) spectra of the dengue cohort and ESI-TOF at different retention times. The spectra display the relative abundance (%) of detected ions across the m/z range. Prominent peaks corresponding to major ionised species are indicated. Variation in spectral profiles between retention times reflects the differences in compound composition and ionisation patterns within the sample. Data were acquired under identical instrumental conditions and are presented as representative scans. (ZIP) [file pntd.0014327.s003.zip › EM COMPLETE SAMPLES SPECTRUM/EM243 SPECTRUM RT 2.873.pdf]

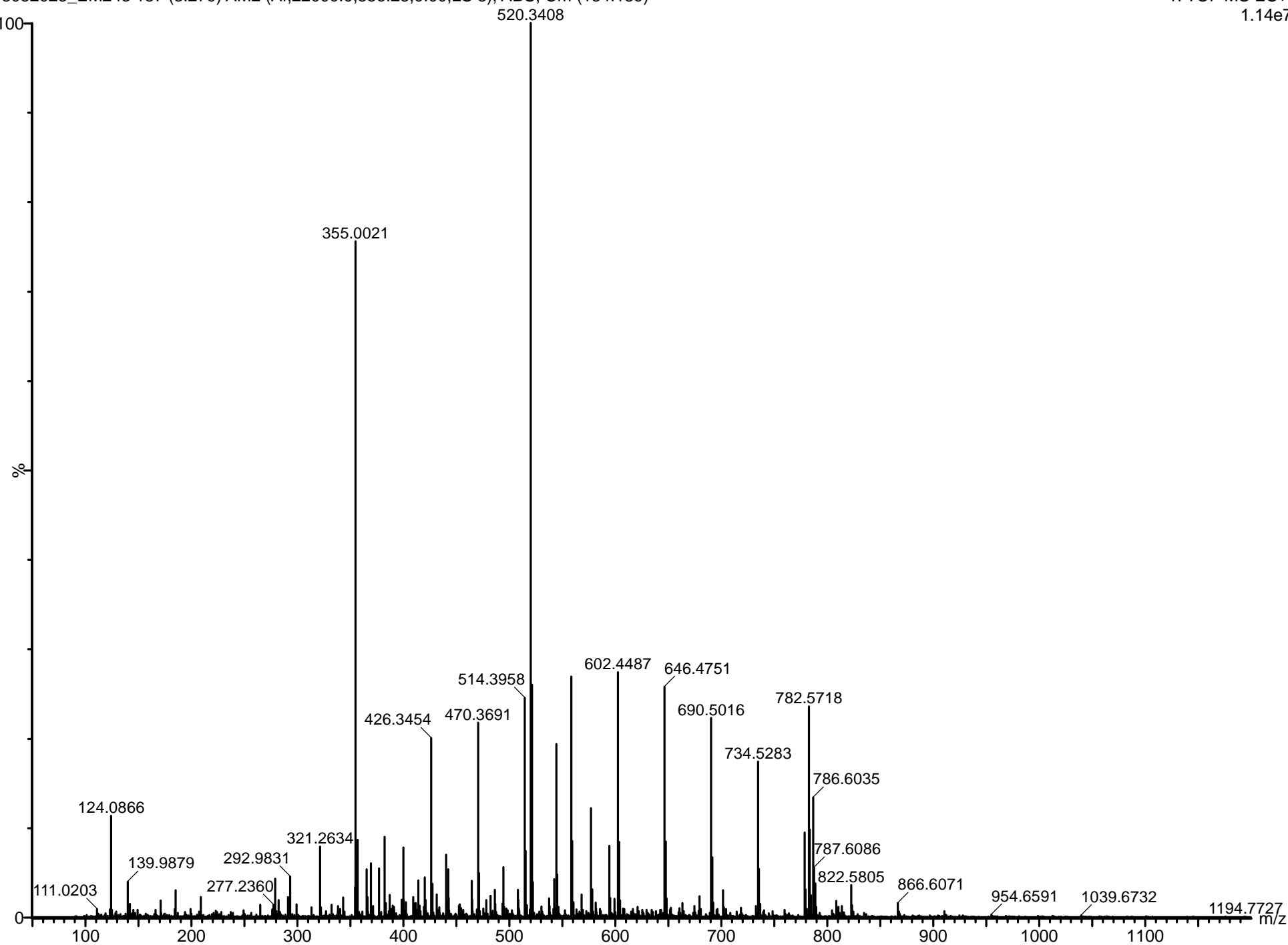

Supplement: S1 Data — Electrospray ionisation time of flight mass spectrometry (ESI-TOF MS, positive mode) spectra of the dengue cohort and ESI-TOF at different retention times. The spectra display the relative abundance (%) of detected ions across the m/z range. Prominent peaks corresponding to major ionised species are indicated. Variation in spectral profiles between retention times reflects the differences in compound composition and ionisation patterns within the sample. Data were acquired under identical instrumental conditions and are presented as representative scans. (ZIP) [file pntd.0014327.s003.zip › EM COMPLETE SAMPLES SPECTRUM/EM243 SPECTRUM RT 3.279.pdf]

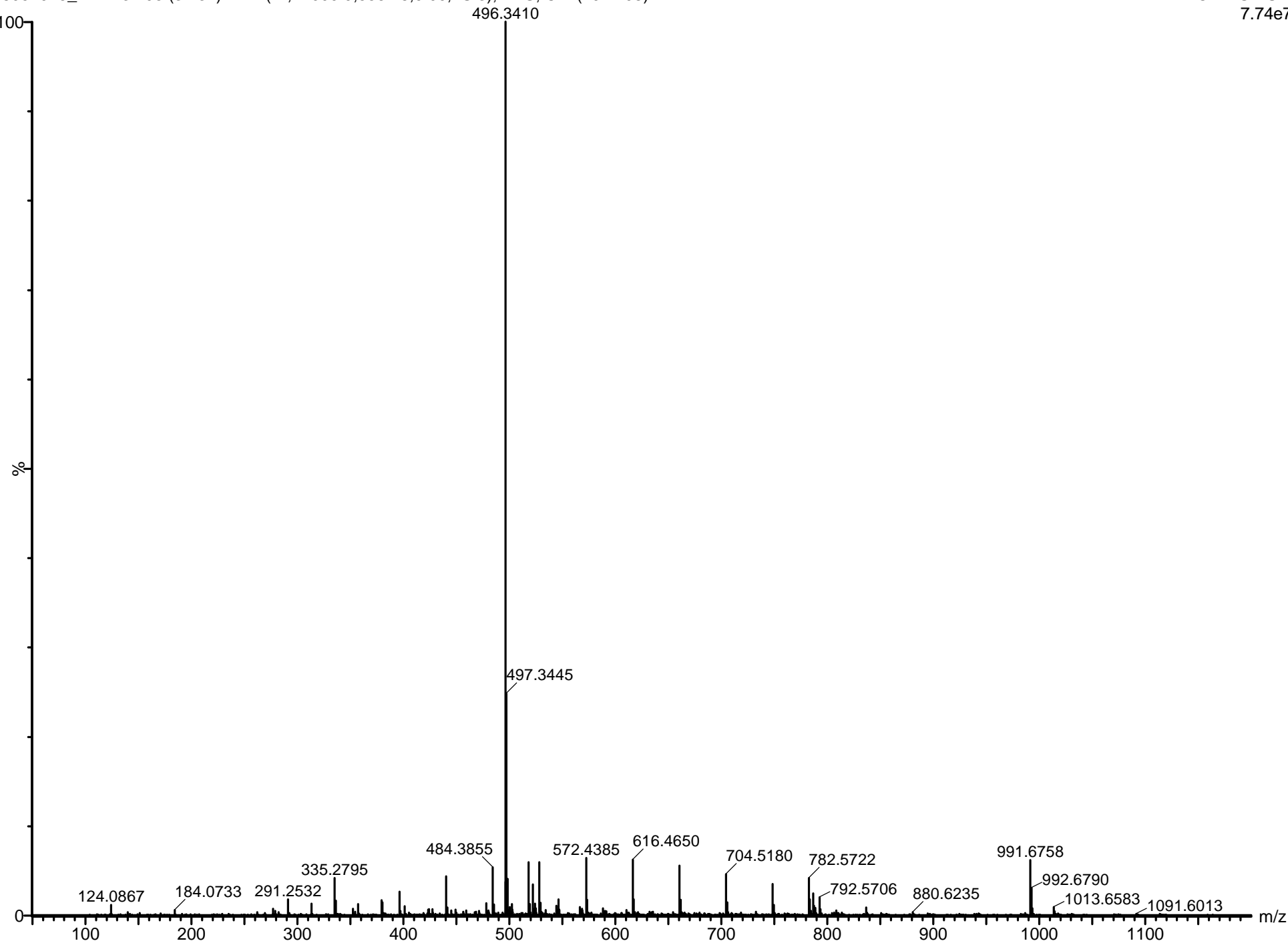

Supplement: S1 Data — Electrospray ionisation time of flight mass spectrometry (ESI-TOF MS, positive mode) spectra of the dengue cohort and ESI-TOF at different retention times. The spectra display the relative abundance (%) of detected ions across the m/z range. Prominent peaks corresponding to major ionised species are indicated. Variation in spectral profiles between retention times reflects the differences in compound composition and ionisation patterns within the sample. Data were acquired under identical instrumental conditions and are presented as representative scans. (ZIP) [file pntd.0014327.s003.zip › EM COMPLETE SAMPLES SPECTRUM/EM243 SPECTRUM RT 3.434.pdf]

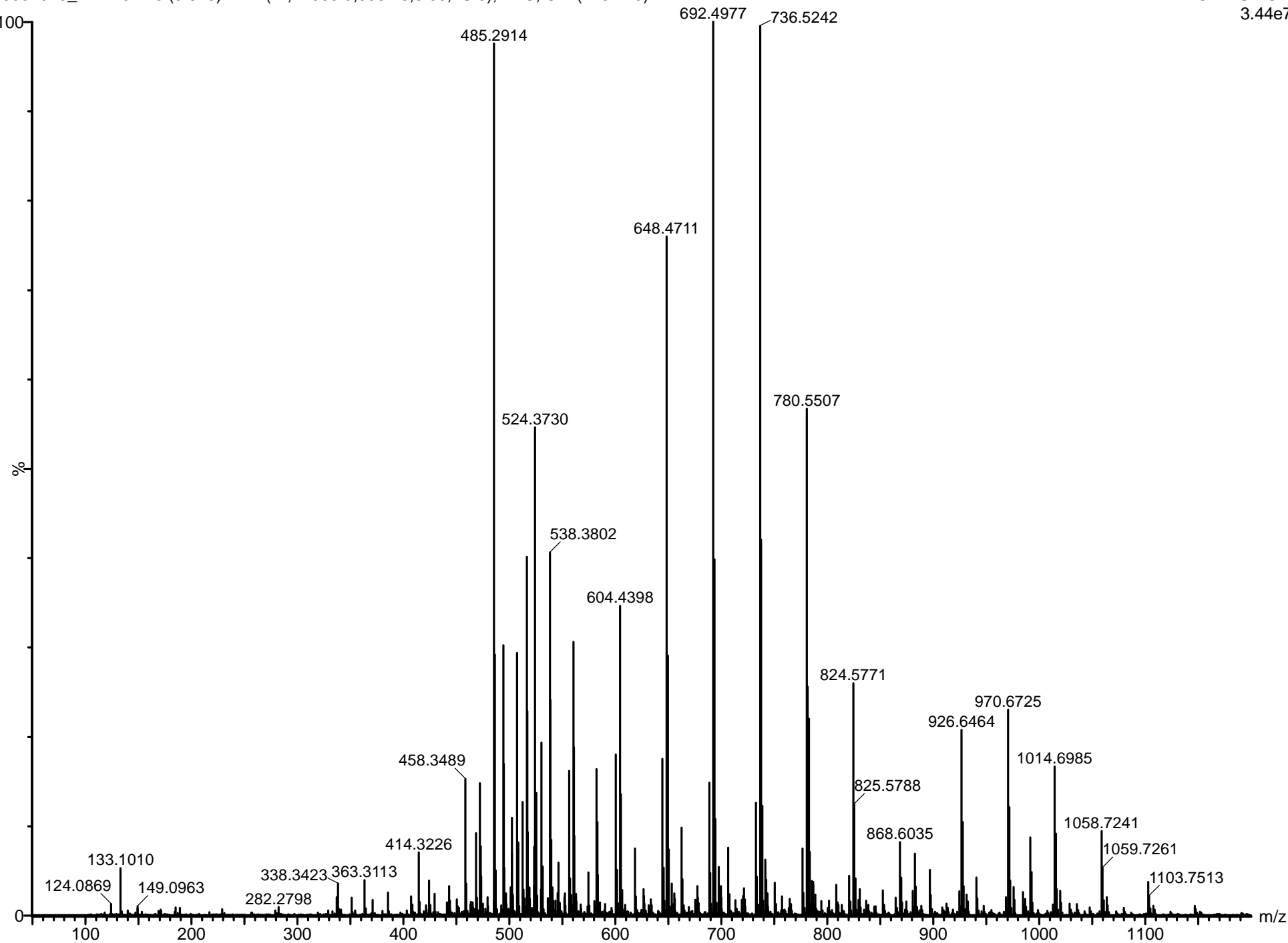

Supplement: S1 Data — Electrospray ionisation time of flight mass spectrometry (ESI-TOF MS, positive mode) spectra of the dengue cohort and ESI-TOF at different retention times. The spectra display the relative abundance (%) of detected ions across the m/z range. Prominent peaks corresponding to major ionised species are indicated. Variation in spectral profiles between retention times reflects the differences in compound composition and ionisation patterns within the sample. Data were acquired under identical instrumental conditions and are presented as representative scans. (ZIP) [file pntd.0014327.s003.zip › EM COMPLETE SAMPLES SPECTRUM/EM243 SPECTRUM RT 3.823.pdf]

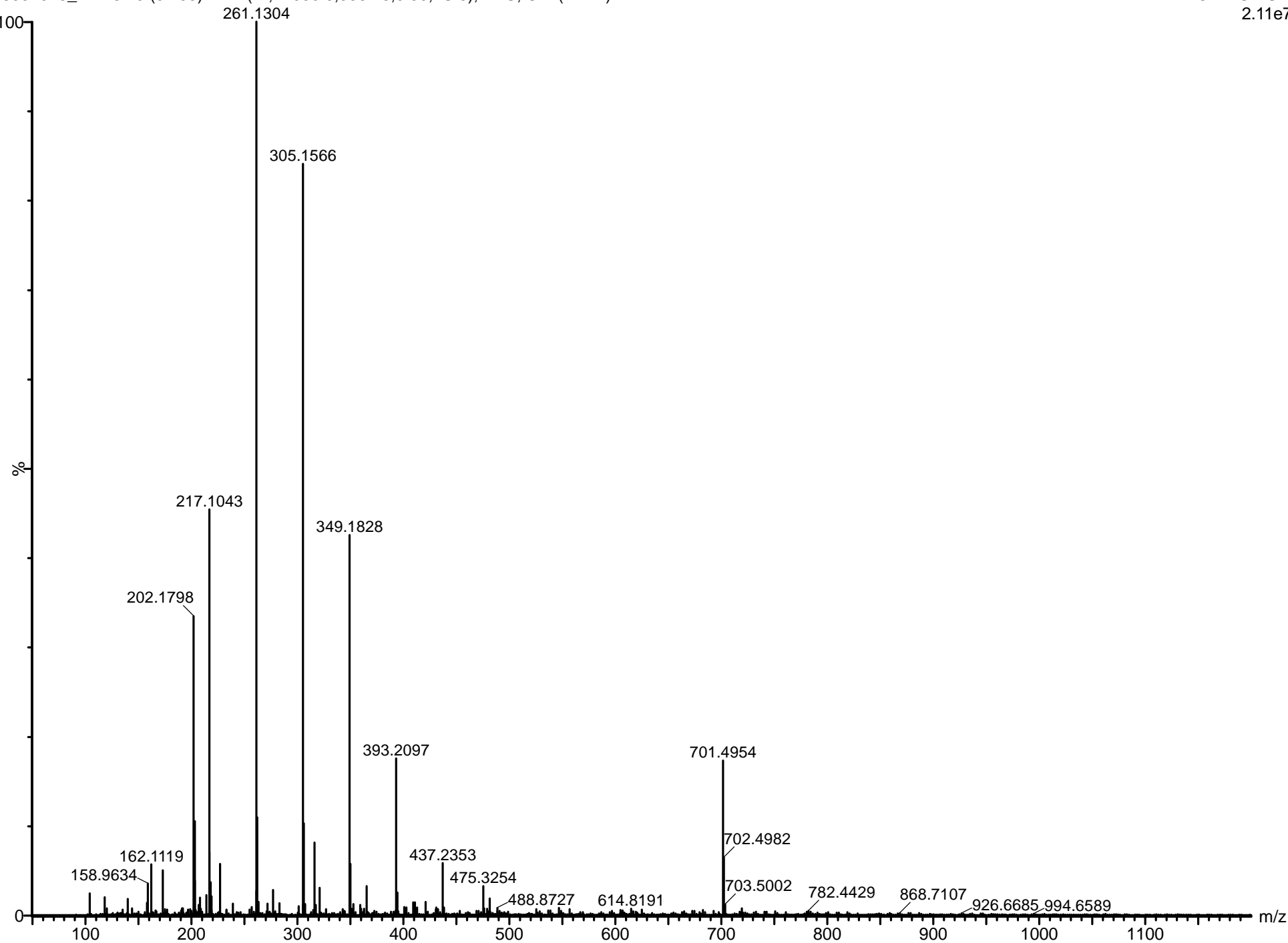

Supplement: S1 Data — Electrospray ionisation time of flight mass spectrometry (ESI-TOF MS, positive mode) spectra of the dengue cohort and ESI-TOF at different retention times. The spectra display the relative abundance (%) of detected ions across the m/z range. Prominent peaks corresponding to major ionised species are indicated. Variation in spectral profiles between retention times reflects the differences in compound composition and ionisation patterns within the sample. Data were acquired under identical instrumental conditions and are presented as representative scans. (ZIP) [file pntd.0014327.s003.zip › EM COMPLETE SAMPLES SPECTRUM/EM26 SPECTRUM RT 0.459.pdf]

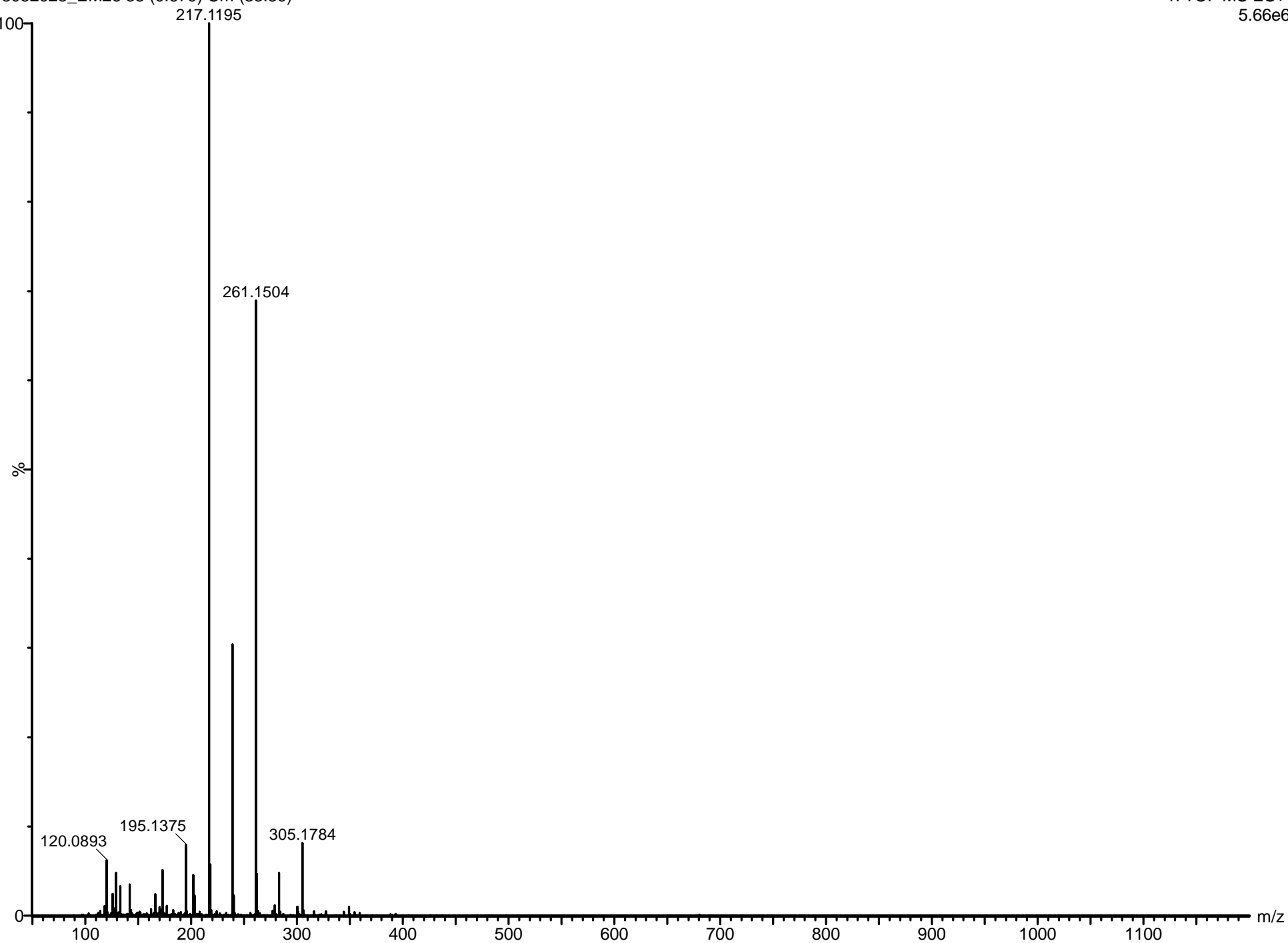

Supplement: S1 Data — Electrospray ionisation time of flight mass spectrometry (ESI-TOF MS, positive mode) spectra of the dengue cohort and ESI-TOF at different retention times. The spectra display the relative abundance (%) of detected ions across the m/z range. Prominent peaks corresponding to major ionised species are indicated. Variation in spectral profiles between retention times reflects the differences in compound composition and ionisation patterns within the sample. Data were acquired under identical instrumental conditions and are presented as representative scans. (ZIP) [file pntd.0014327.s003.zip › EM COMPLETE SAMPLES SPECTRUM/EM26 SPECTRUM RT 0.679.pdf]

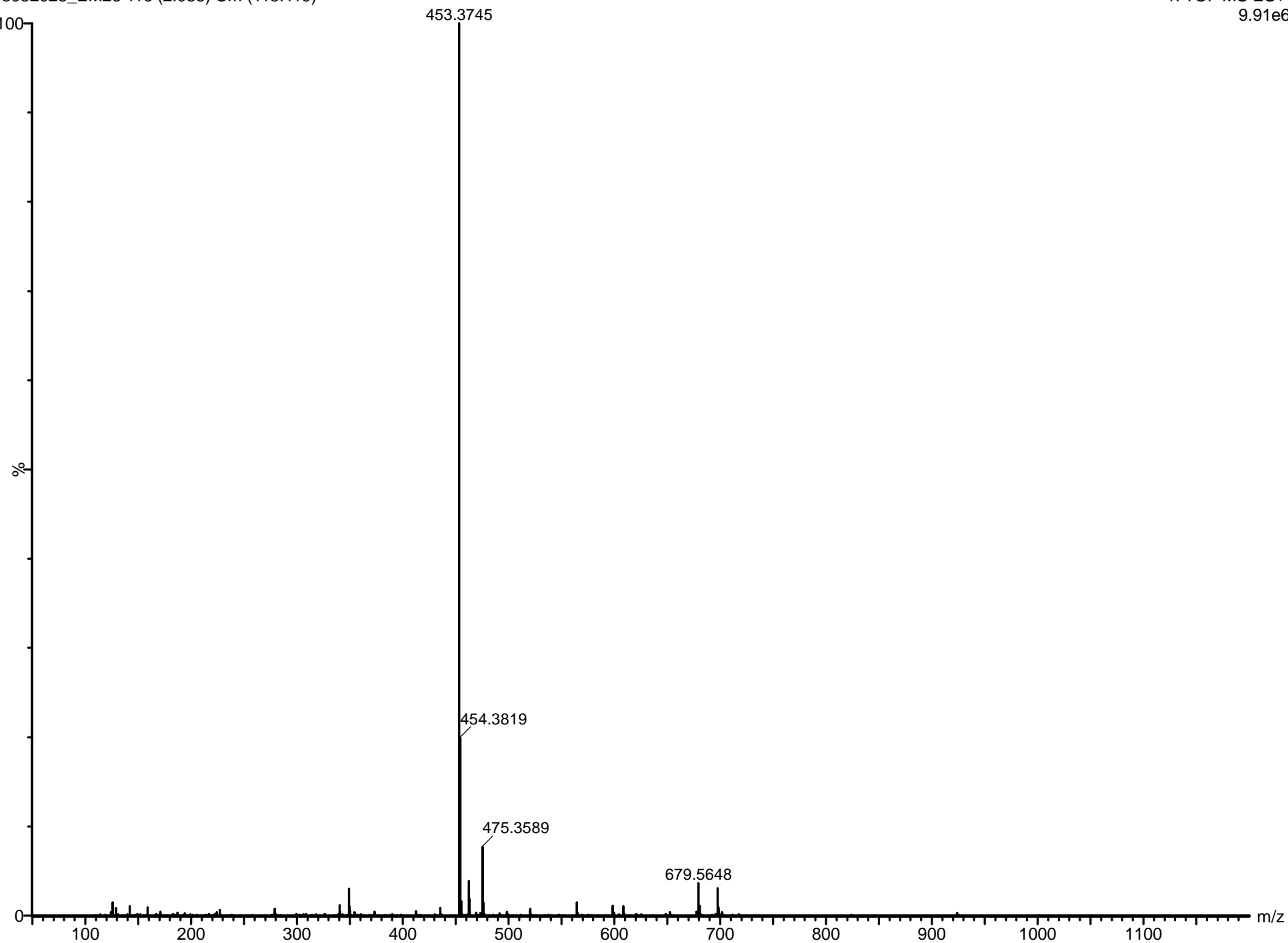

Supplement: S1 Data — Electrospray ionisation time of flight mass spectrometry (ESI-TOF MS, positive mode) spectra of the dengue cohort and ESI-TOF at different retention times. The spectra display the relative abundance (%) of detected ions across the m/z range. Prominent peaks corresponding to major ionised species are indicated. Variation in spectral profiles between retention times reflects the differences in compound composition and ionisation patterns within the sample. Data were acquired under identical instrumental conditions and are presented as representative scans. (ZIP) [file pntd.0014327.s003.zip › EM COMPLETE SAMPLES SPECTRUM/EM26 SPECTRUM RT 2.058.pdf]

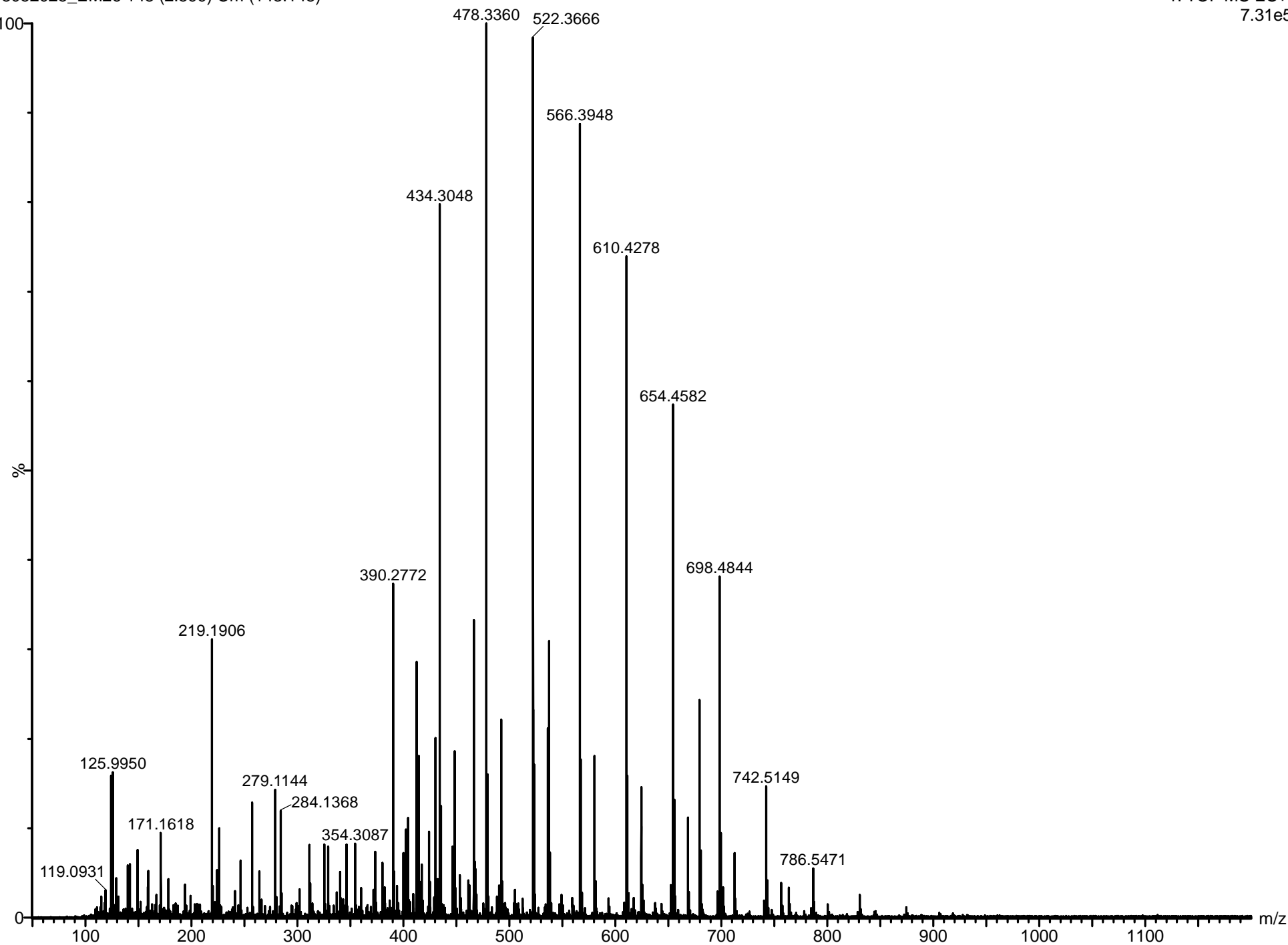

Supplement: S1 Data — Electrospray ionisation time of flight mass spectrometry (ESI-TOF MS, positive mode) spectra of the dengue cohort and ESI-TOF at different retention times. The spectra display the relative abundance (%) of detected ions across the m/z range. Prominent peaks corresponding to major ionised species are indicated. Variation in spectral profiles between retention times reflects the differences in compound composition and ionisation patterns within the sample. Data were acquired under identical instrumental conditions and are presented as representative scans. (ZIP) [file pntd.0014327.s003.zip › EM COMPLETE SAMPLES SPECTRUM/EM26 SPECTRUM RT 2.559.pdf]

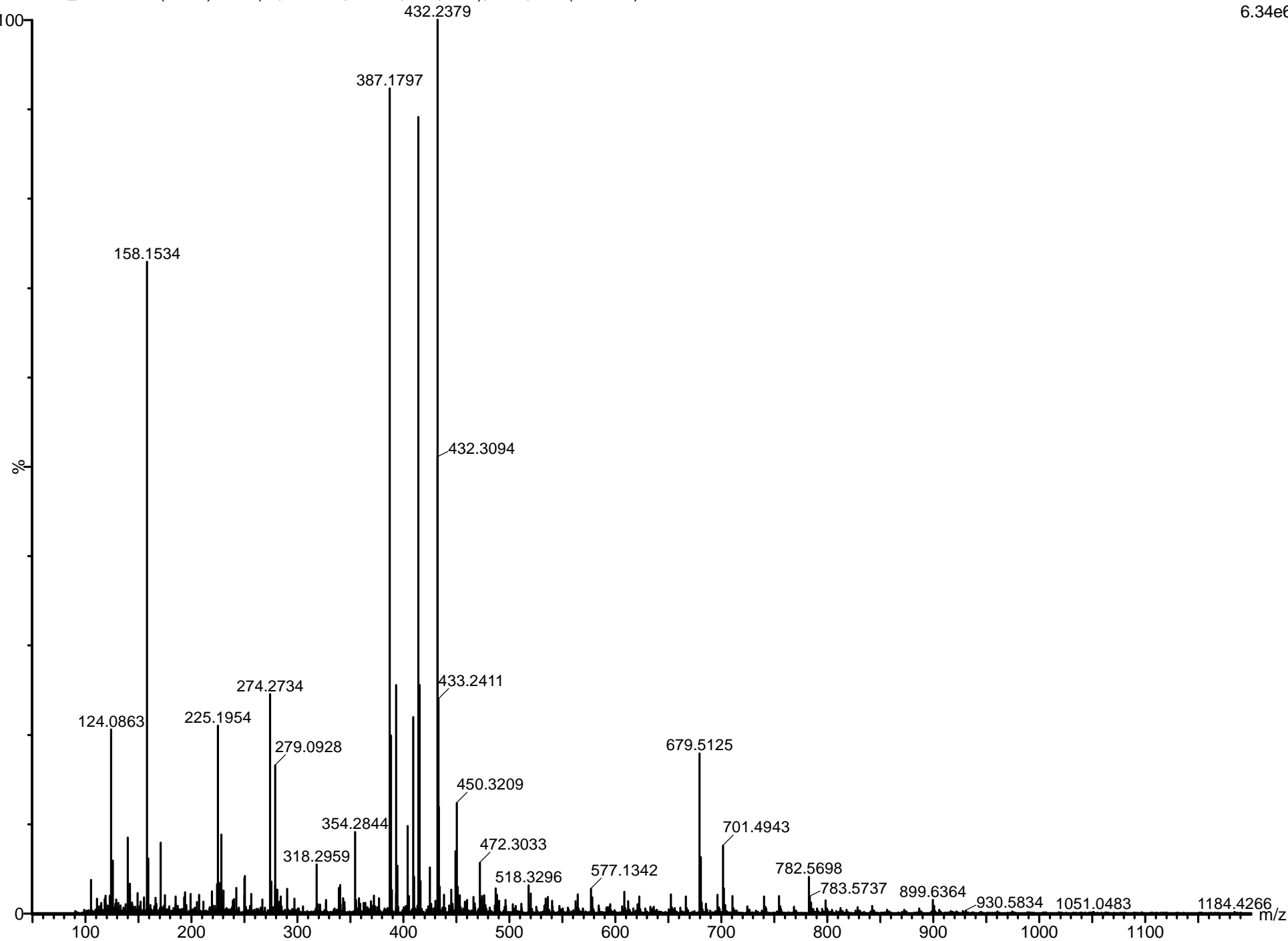

Supplement: S1 Data — Electrospray ionisation time of flight mass spectrometry (ESI-TOF MS, positive mode) spectra of the dengue cohort and ESI-TOF at different retention times. The spectra display the relative abundance (%) of detected ions across the m/z range. Prominent peaks corresponding to major ionised species are indicated. Variation in spectral profiles between retention times reflects the differences in compound composition and ionisation patterns within the sample. Data were acquired under identical instrumental conditions and are presented as representative scans. (ZIP) [file pntd.0014327.s003.zip › EM COMPLETE SAMPLES SPECTRUM/EM26 SPECTRUM RT 2.873.pdf]

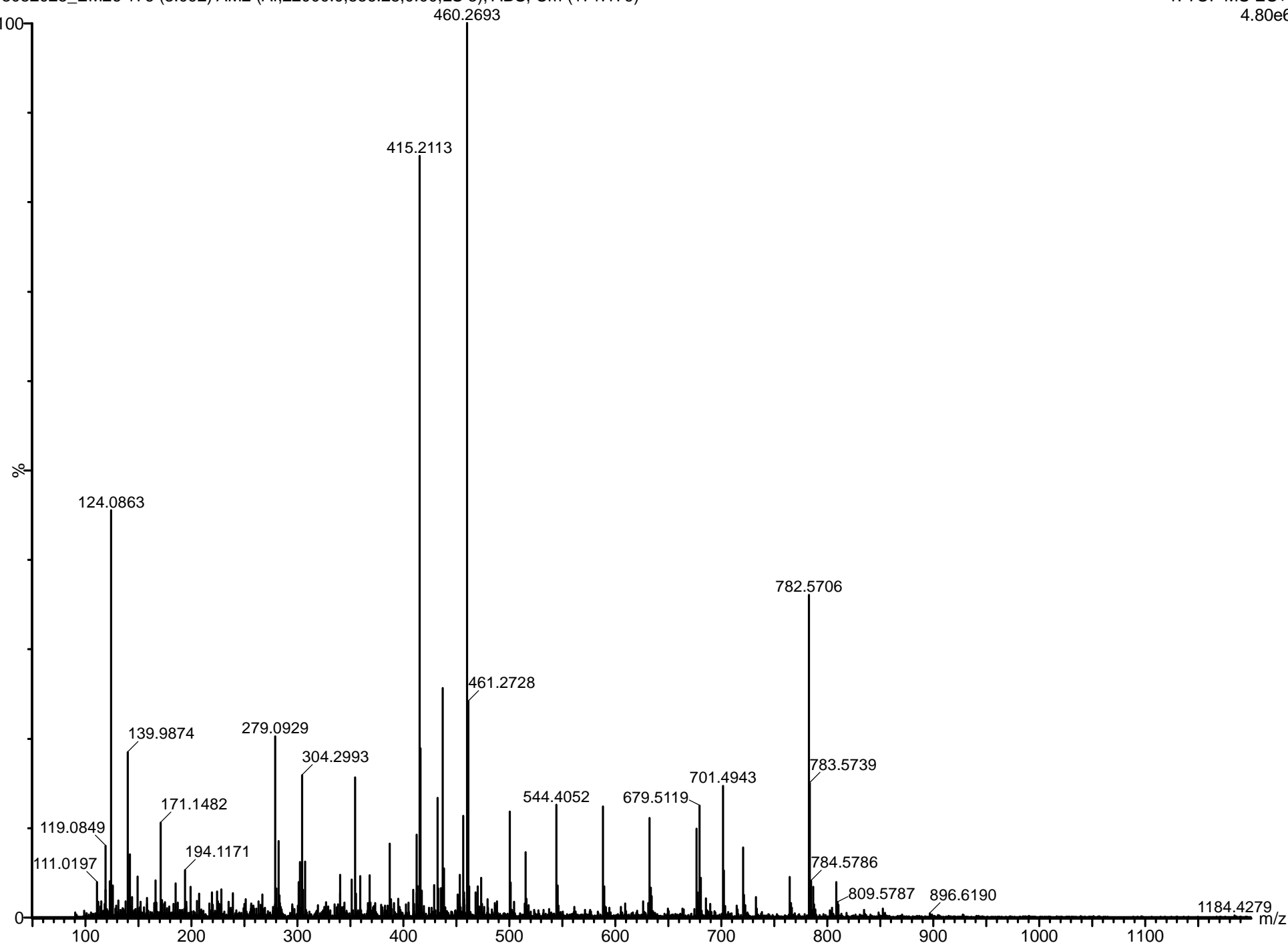

Supplement: S1 Data — Electrospray ionisation time of flight mass spectrometry (ESI-TOF MS, positive mode) spectra of the dengue cohort and ESI-TOF at different retention times. The spectra display the relative abundance (%) of detected ions across the m/z range. Prominent peaks corresponding to major ionised species are indicated. Variation in spectral profiles between retention times reflects the differences in compound composition and ionisation patterns within the sample. Data were acquired under identical instrumental conditions and are presented as representative scans. (ZIP) [file pntd.0014327.s003.zip › EM COMPLETE SAMPLES SPECTRUM/EM26 SPECTRUM RT 3.092.pdf]

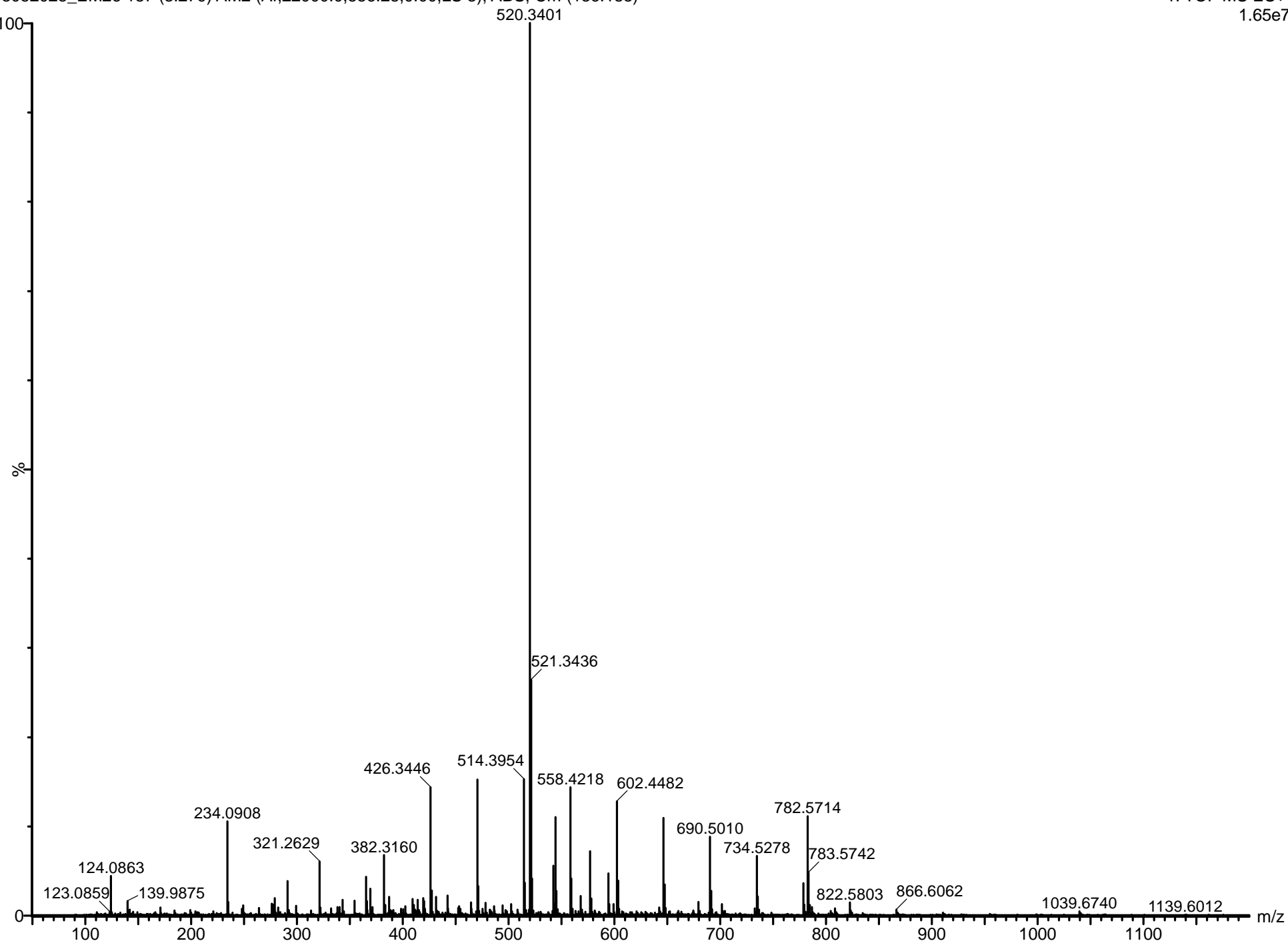

Supplement: S1 Data — Electrospray ionisation time of flight mass spectrometry (ESI-TOF MS, positive mode) spectra of the dengue cohort and ESI-TOF at different retention times. The spectra display the relative abundance (%) of detected ions across the m/z range. Prominent peaks corresponding to major ionised species are indicated. Variation in spectral profiles between retention times reflects the differences in compound composition and ionisation patterns within the sample. Data were acquired under identical instrumental conditions and are presented as representative scans. (ZIP) [file pntd.0014327.s003.zip › EM COMPLETE SAMPLES SPECTRUM/EM26 SPECTRUM RT 3.279.pdf]

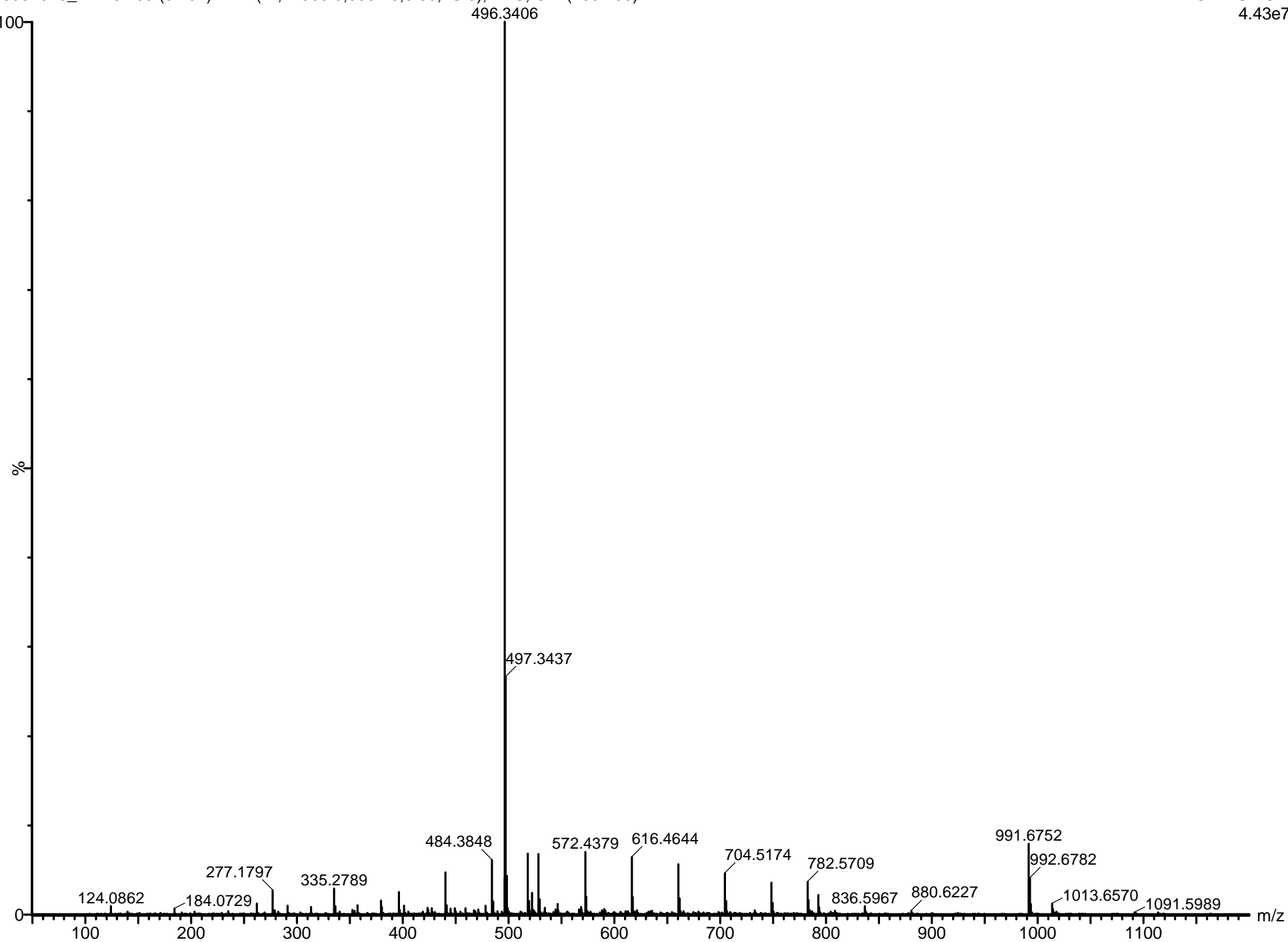

Supplement: S1 Data — Electrospray ionisation time of flight mass spectrometry (ESI-TOF MS, positive mode) spectra of the dengue cohort and ESI-TOF at different retention times. The spectra display the relative abundance (%) of detected ions across the m/z range. Prominent peaks corresponding to major ionised species are indicated. Variation in spectral profiles between retention times reflects the differences in compound composition and ionisation patterns within the sample. Data were acquired under identical instrumental conditions and are presented as representative scans. (ZIP) [file pntd.0014327.s003.zip › EM COMPLETE SAMPLES SPECTRUM/EM26 SPECTRUM RT 3.434.pdf]

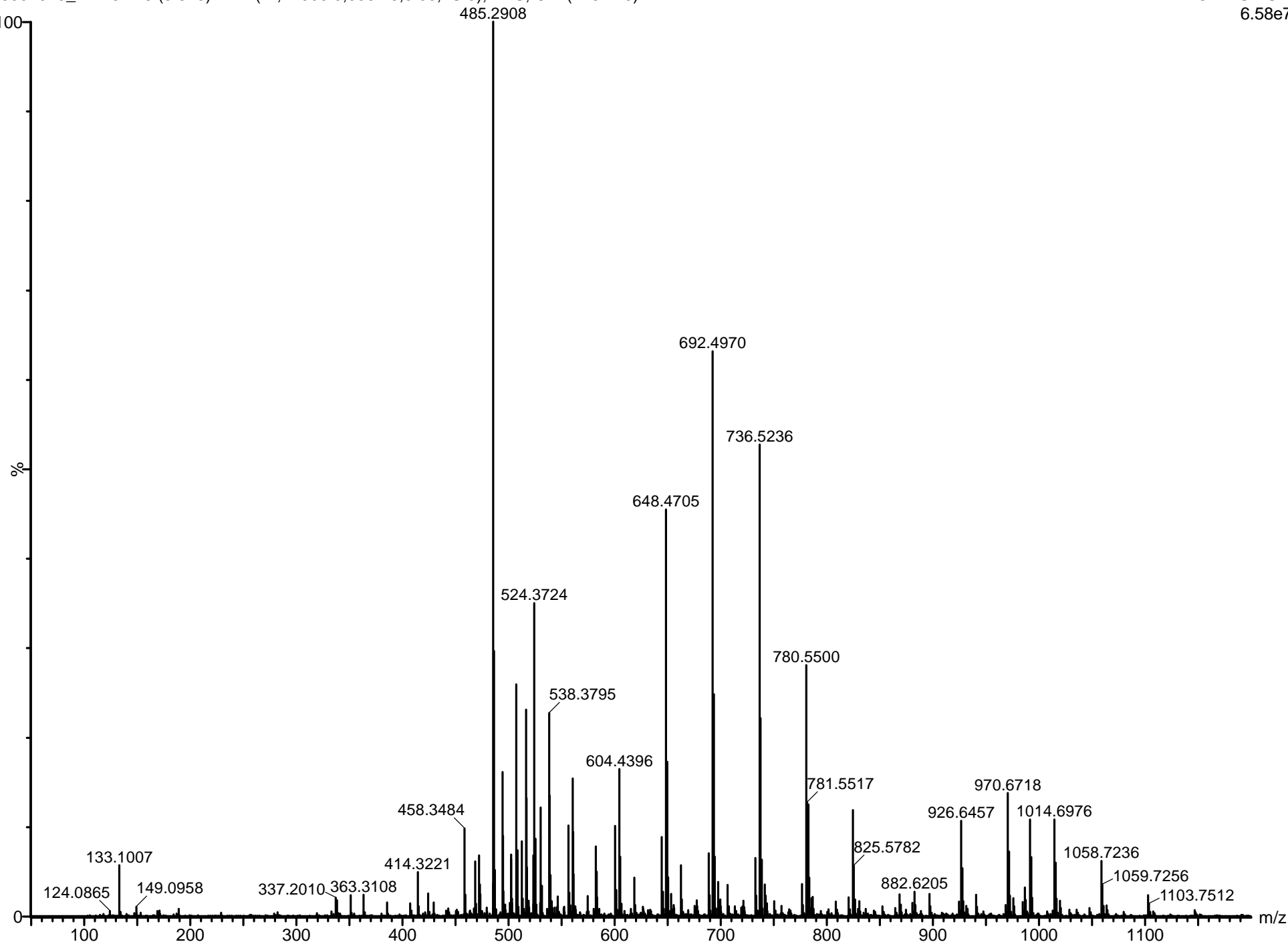

Supplement: S1 Data — Electrospray ionisation time of flight mass spectrometry (ESI-TOF MS, positive mode) spectra of the dengue cohort and ESI-TOF at different retention times. The spectra display the relative abundance (%) of detected ions across the m/z range. Prominent peaks corresponding to major ionised species are indicated. Variation in spectral profiles between retention times reflects the differences in compound composition and ionisation patterns within the sample. Data were acquired under identical instrumental conditions and are presented as representative scans. (ZIP) [file pntd.0014327.s003.zip › EM COMPLETE SAMPLES SPECTRUM/EM26 SPECTRUM RT 3.823.pdf]

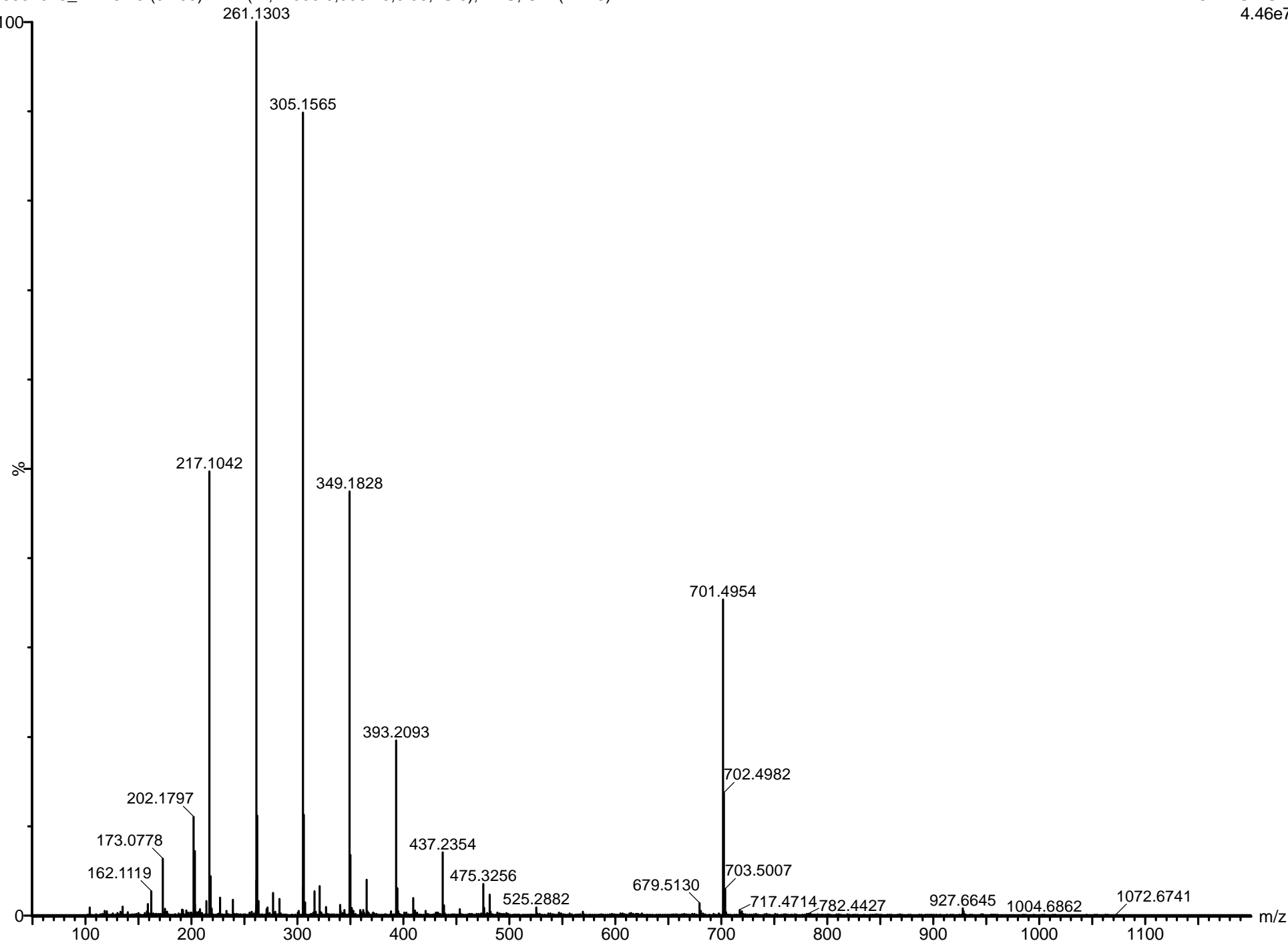

Supplement: S1 Data — Electrospray ionisation time of flight mass spectrometry (ESI-TOF MS, positive mode) spectra of the dengue cohort and ESI-TOF at different retention times. The spectra display the relative abundance (%) of detected ions across the m/z range. Prominent peaks corresponding to major ionised species are indicated. Variation in spectral profiles between retention times reflects the differences in compound composition and ionisation patterns within the sample. Data were acquired under identical instrumental conditions and are presented as representative scans. (ZIP) [file pntd.0014327.s003.zip › EM COMPLETE SAMPLES SPECTRUM/EM28 SPECTRUM RT 0.459.pdf]

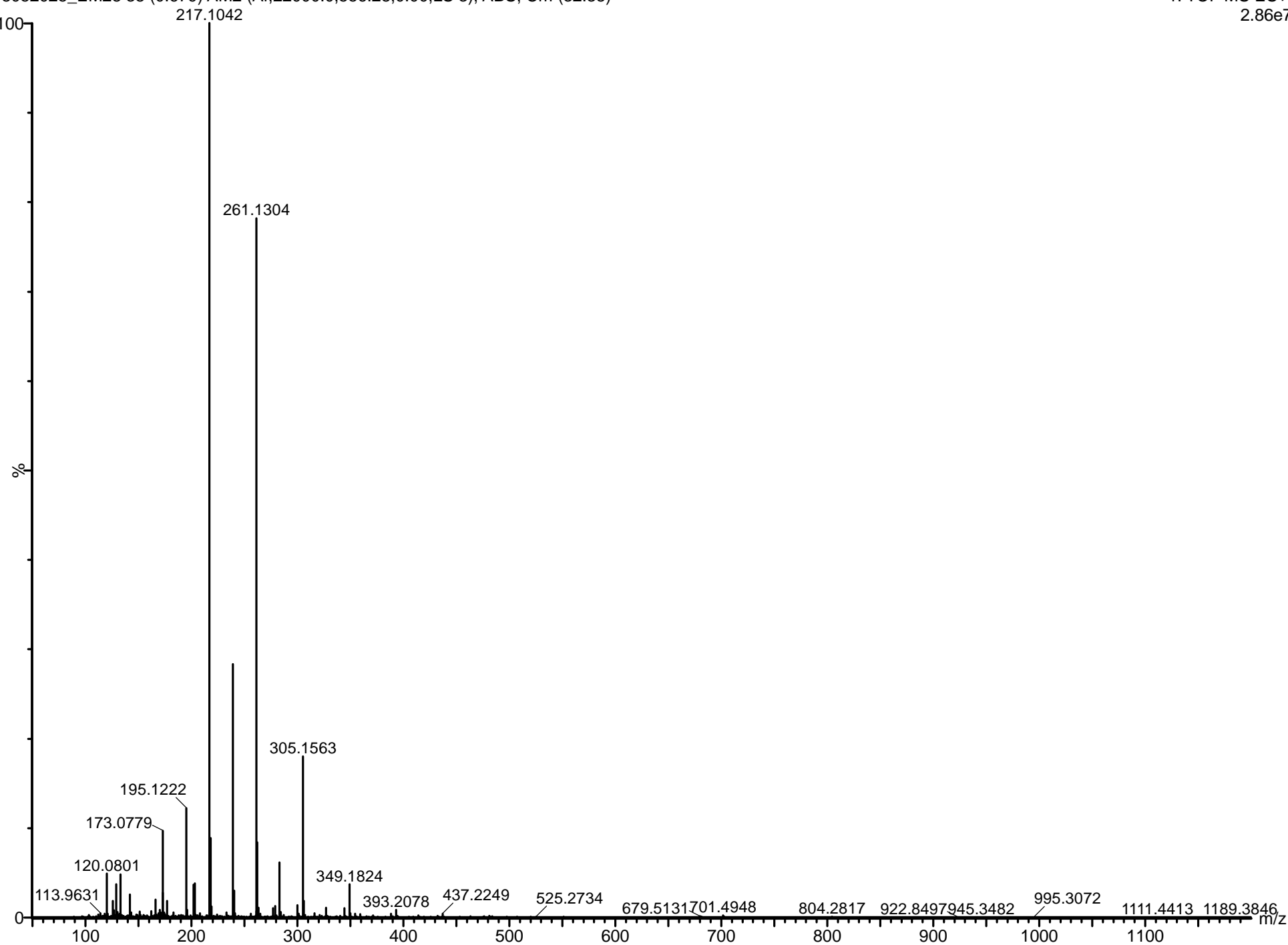

Supplement: S1 Data — Electrospray ionisation time of flight mass spectrometry (ESI-TOF MS, positive mode) spectra of the dengue cohort and ESI-TOF at different retention times. The spectra display the relative abundance (%) of detected ions across the m/z range. Prominent peaks corresponding to major ionised species are indicated. Variation in spectral profiles between retention times reflects the differences in compound composition and ionisation patterns within the sample. Data were acquired under identical instrumental conditions and are presented as representative scans. (ZIP) [file pntd.0014327.s003.zip › EM COMPLETE SAMPLES SPECTRUM/EM28 SPECTRUM RT 0.679.pdf]

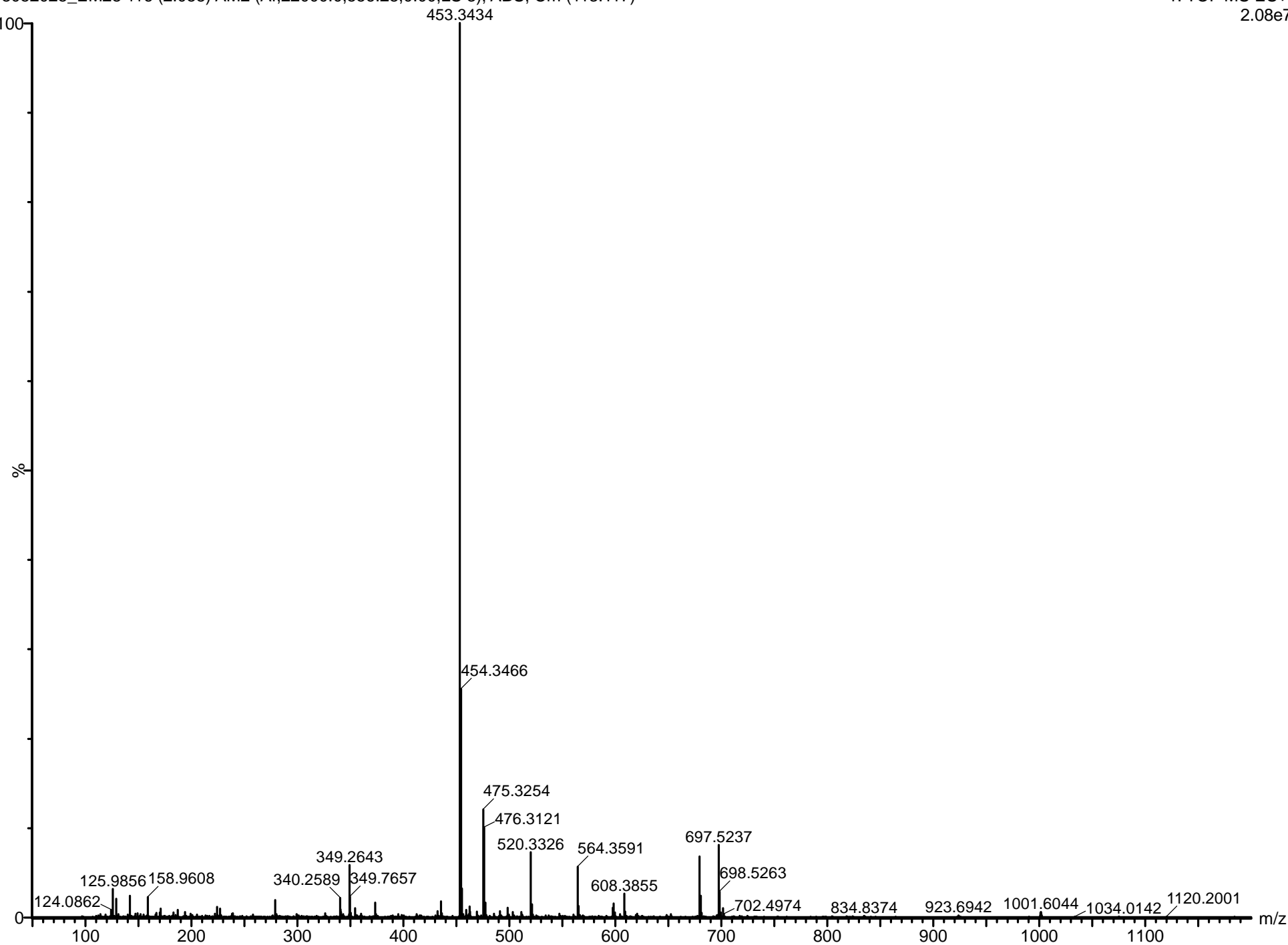

Supplement: S1 Data — Electrospray ionisation time of flight mass spectrometry (ESI-TOF MS, positive mode) spectra of the dengue cohort and ESI-TOF at different retention times. The spectra display the relative abundance (%) of detected ions across the m/z range. Prominent peaks corresponding to major ionised species are indicated. Variation in spectral profiles between retention times reflects the differences in compound composition and ionisation patterns within the sample. Data were acquired under identical instrumental conditions and are presented as representative scans. (ZIP) [file pntd.0014327.s003.zip › EM COMPLETE SAMPLES SPECTRUM/EM28 SPECTRUM RT 2.058.pdf]

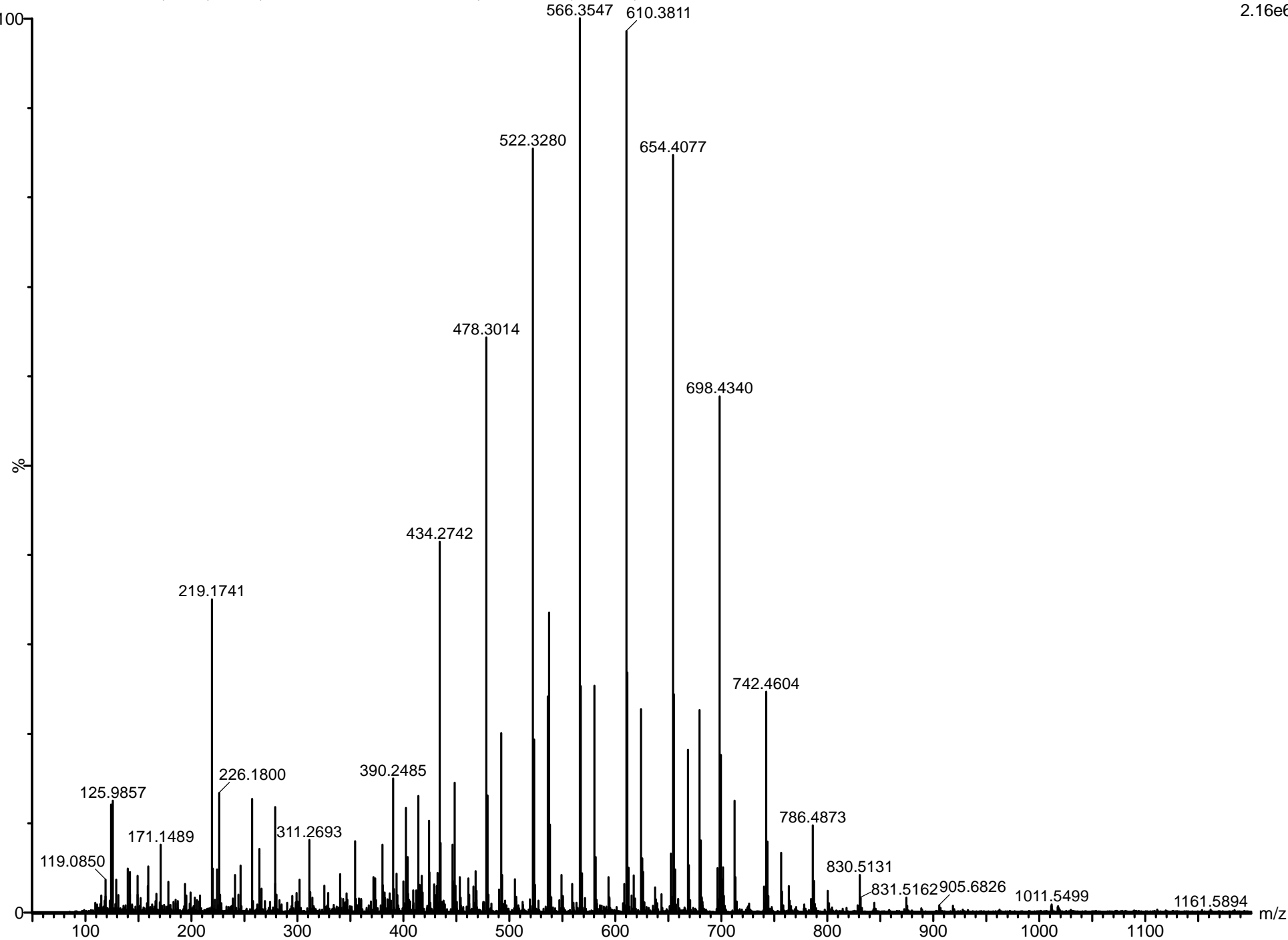

Supplement: S1 Data — Electrospray ionisation time of flight mass spectrometry (ESI-TOF MS, positive mode) spectra of the dengue cohort and ESI-TOF at different retention times. The spectra display the relative abundance (%) of detected ions across the m/z range. Prominent peaks corresponding to major ionised species are indicated. Variation in spectral profiles between retention times reflects the differences in compound composition and ionisation patterns within the sample. Data were acquired under identical instrumental conditions and are presented as representative scans. (ZIP) [file pntd.0014327.s003.zip › EM COMPLETE SAMPLES SPECTRUM/EM28 SPECTRUM RT 2.565.pdf]

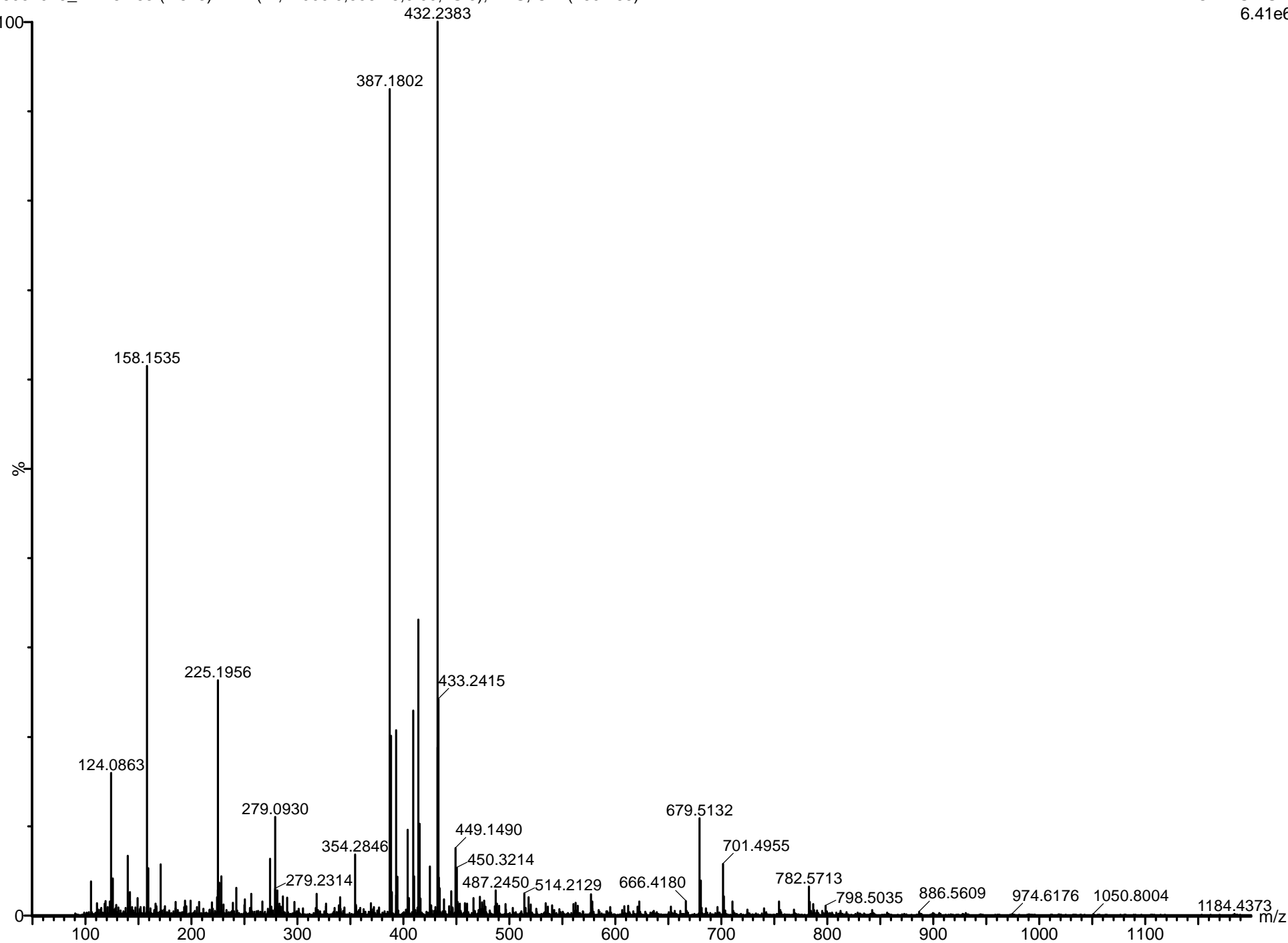

Supplement: S1 Data — Electrospray ionisation time of flight mass spectrometry (ESI-TOF MS, positive mode) spectra of the dengue cohort and ESI-TOF at different retention times. The spectra display the relative abundance (%) of detected ions across the m/z range. Prominent peaks corresponding to major ionised species are indicated. Variation in spectral profiles between retention times reflects the differences in compound composition and ionisation patterns within the sample. Data were acquired under identical instrumental conditions and are presented as representative scans. (ZIP) [file pntd.0014327.s003.zip › EM COMPLETE SAMPLES SPECTRUM/EM28 SPECTRUM RT 2.873.pdf]

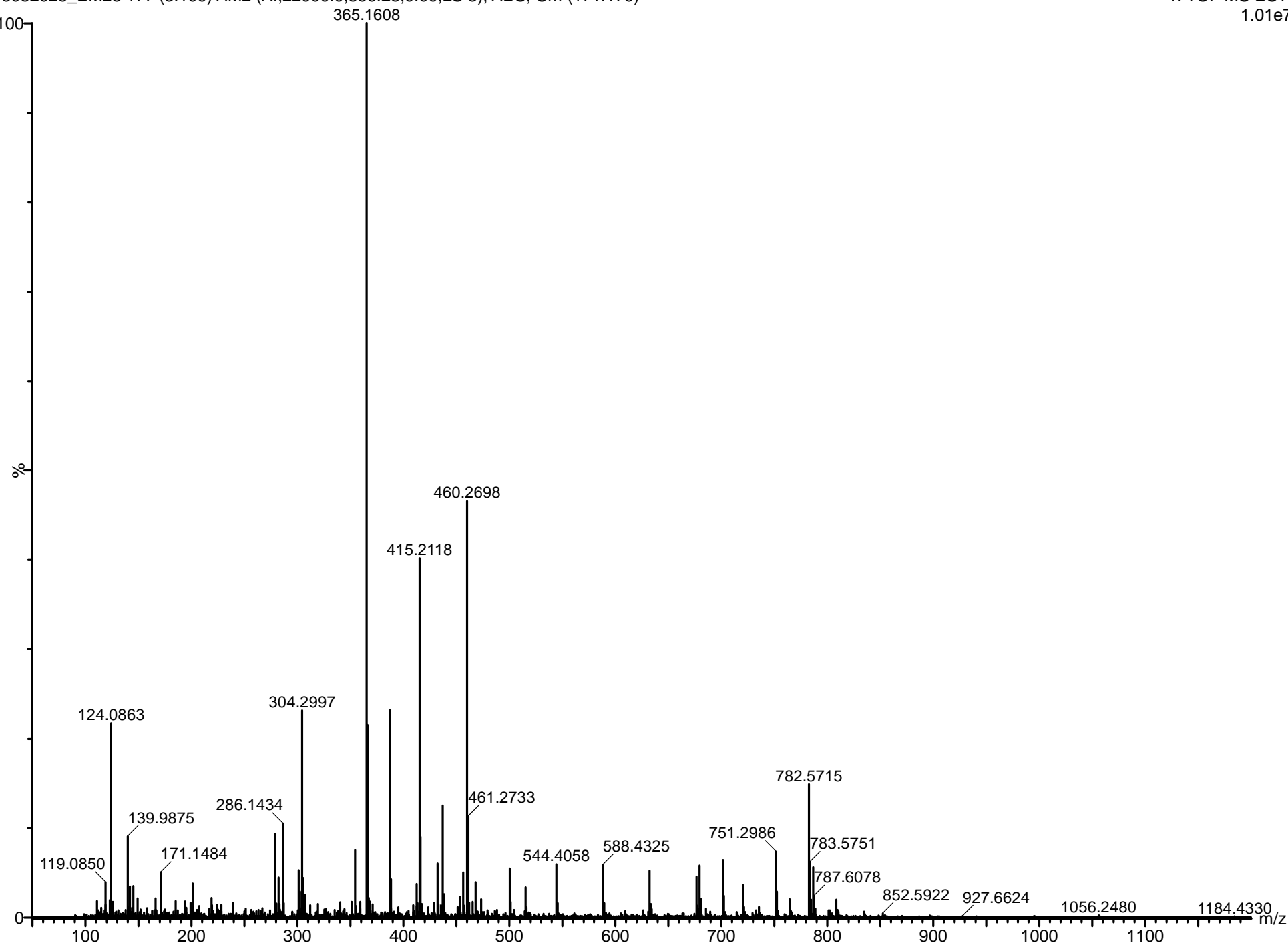

Supplement: S1 Data — Electrospray ionisation time of flight mass spectrometry (ESI-TOF MS, positive mode) spectra of the dengue cohort and ESI-TOF at different retention times. The spectra display the relative abundance (%) of detected ions across the m/z range. Prominent peaks corresponding to major ionised species are indicated. Variation in spectral profiles between retention times reflects the differences in compound composition and ionisation patterns within the sample. Data were acquired under identical instrumental conditions and are presented as representative scans. (ZIP) [file pntd.0014327.s003.zip › EM COMPLETE SAMPLES SPECTRUM/EM28 SPECTRUM RT 3.109.pdf]

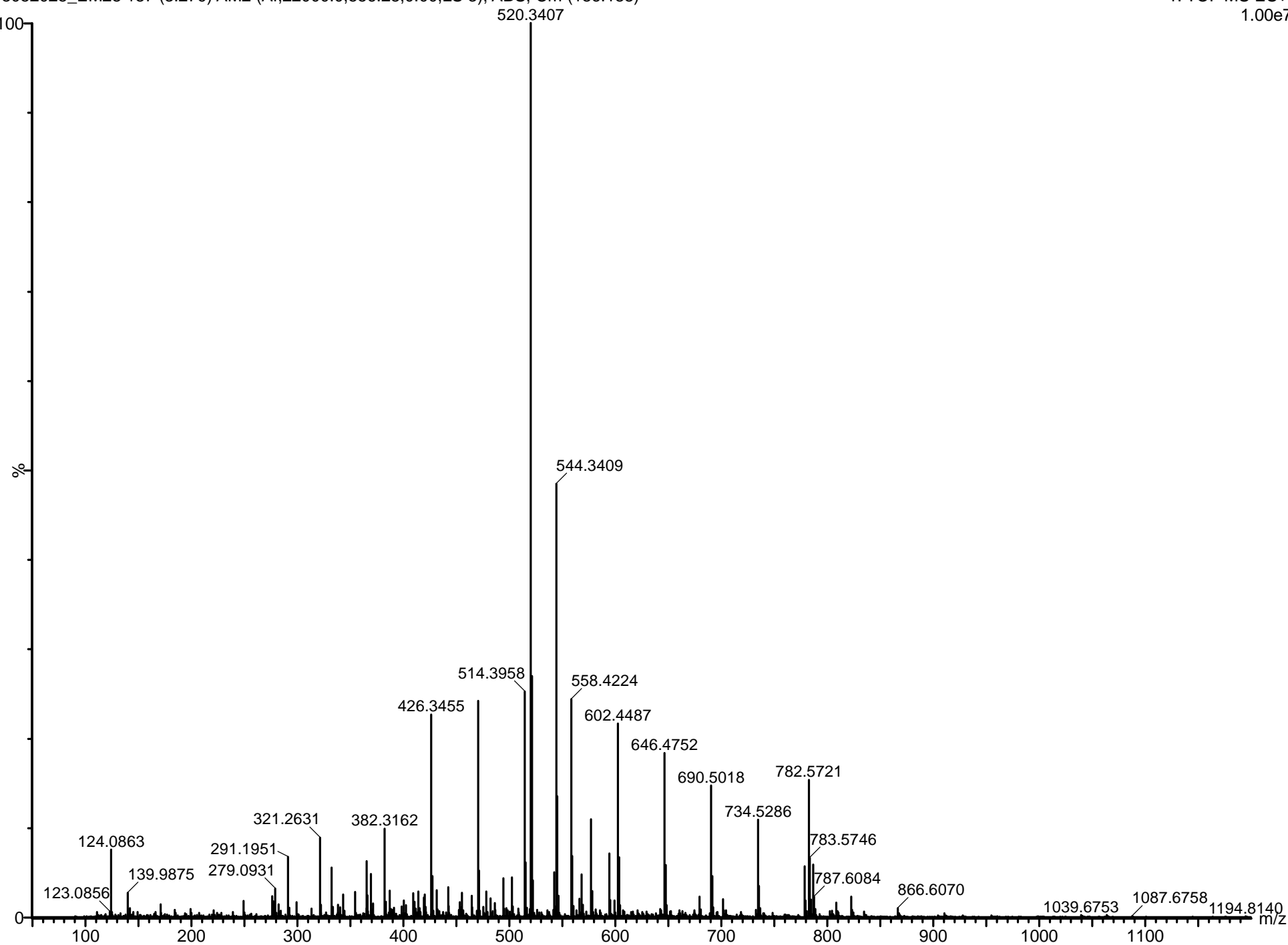

Supplement: S1 Data — Electrospray ionisation time of flight mass spectrometry (ESI-TOF MS, positive mode) spectra of the dengue cohort and ESI-TOF at different retention times. The spectra display the relative abundance (%) of detected ions across the m/z range. Prominent peaks corresponding to major ionised species are indicated. Variation in spectral profiles between retention times reflects the differences in compound composition and ionisation patterns within the sample. Data were acquired under identical instrumental conditions and are presented as representative scans. (ZIP) [file pntd.0014327.s003.zip › EM COMPLETE SAMPLES SPECTRUM/EM28 SPECTRUM RT 3.279.pdf]

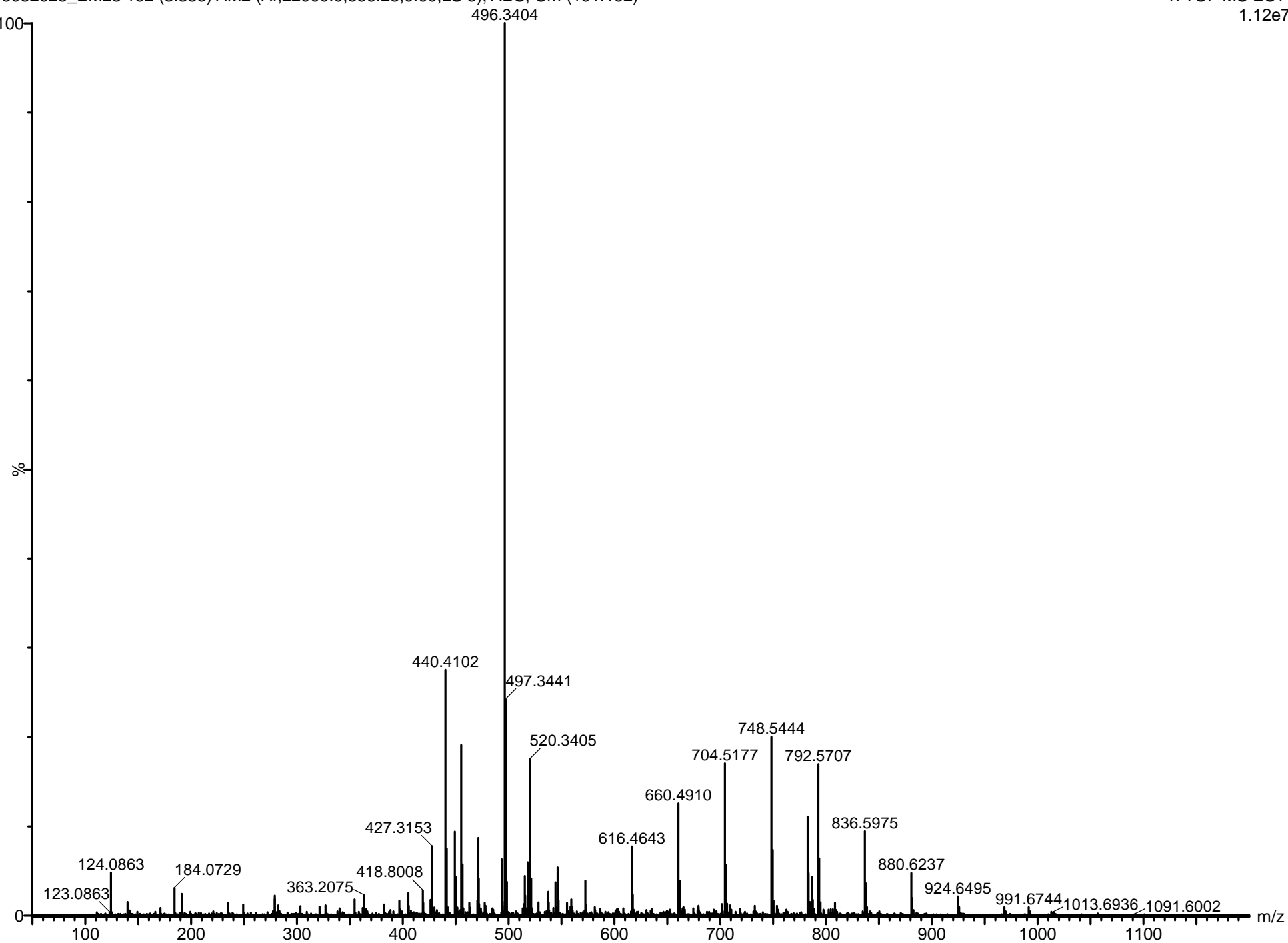

Supplement: S1 Data — Electrospray ionisation time of flight mass spectrometry (ESI-TOF MS, positive mode) spectra of the dengue cohort and ESI-TOF at different retention times. The spectra display the relative abundance (%) of detected ions across the m/z range. Prominent peaks corresponding to major ionised species are indicated. Variation in spectral profiles between retention times reflects the differences in compound composition and ionisation patterns within the sample. Data were acquired under identical instrumental conditions and are presented as representative scans. (ZIP) [file pntd.0014327.s003.zip › EM COMPLETE SAMPLES SPECTRUM/EM28 SPECTRUM RT 3.383.pdf]

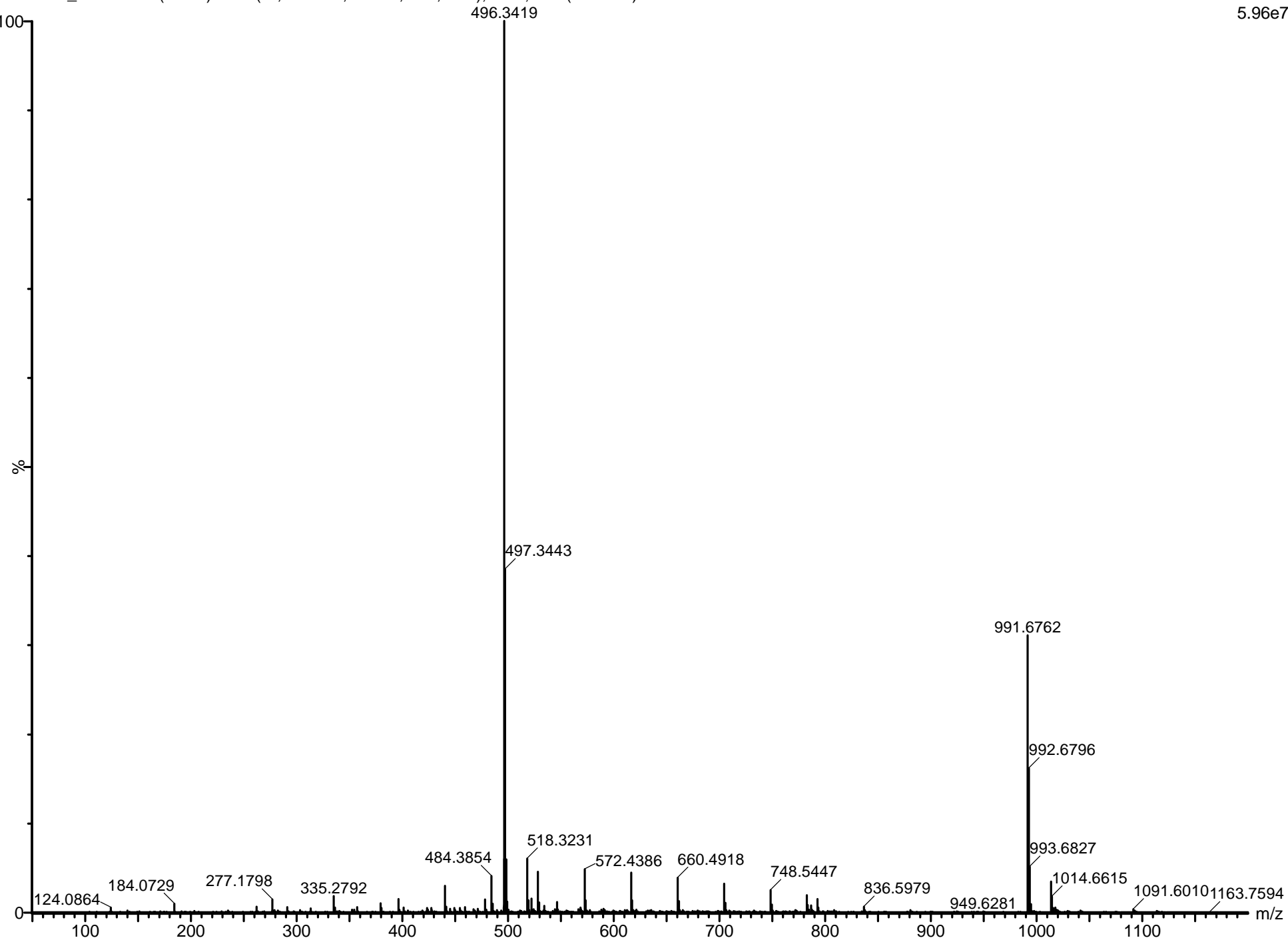

Supplement: S1 Data — Electrospray ionisation time of flight mass spectrometry (ESI-TOF MS, positive mode) spectra of the dengue cohort and ESI-TOF at different retention times. The spectra display the relative abundance (%) of detected ions across the m/z range. Prominent peaks corresponding to major ionised species are indicated. Variation in spectral profiles between retention times reflects the differences in compound composition and ionisation patterns within the sample. Data were acquired under identical instrumental conditions and are presented as representative scans. (ZIP) [file pntd.0014327.s003.zip › EM COMPLETE SAMPLES SPECTRUM/EM28 SPECTRUM RT 3.434.pdf]

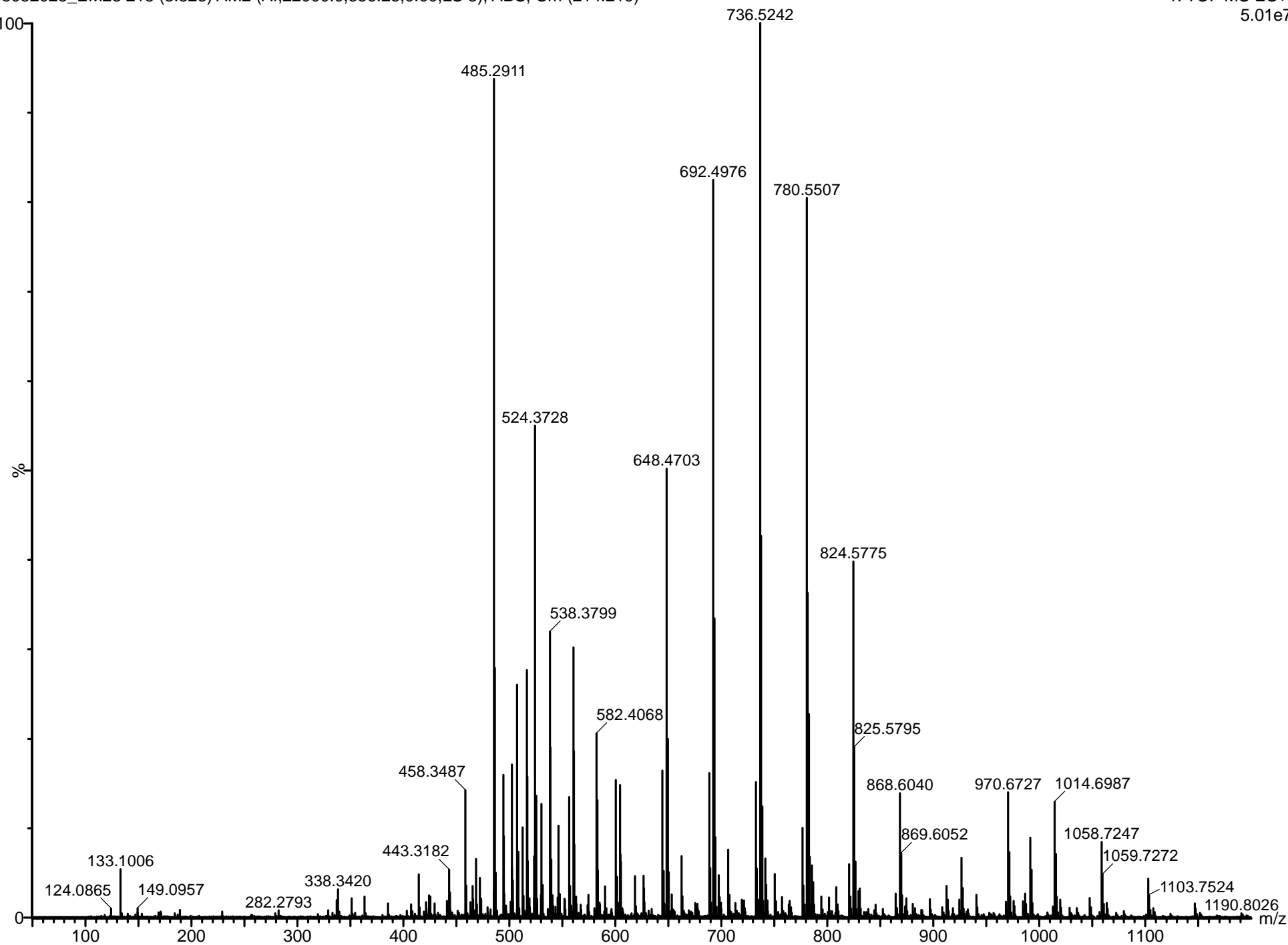

Supplement: S1 Data — Electrospray ionisation time of flight mass spectrometry (ESI-TOF MS, positive mode) spectra of the dengue cohort and ESI-TOF at different retention times. The spectra display the relative abundance (%) of detected ions across the m/z range. Prominent peaks corresponding to major ionised species are indicated. Variation in spectral profiles between retention times reflects the differences in compound composition and ionisation patterns within the sample. Data were acquired under identical instrumental conditions and are presented as representative scans. (ZIP) [file pntd.0014327.s003.zip › EM COMPLETE SAMPLES SPECTRUM/EM28 SPECTRUM RT 3.823.pdf]

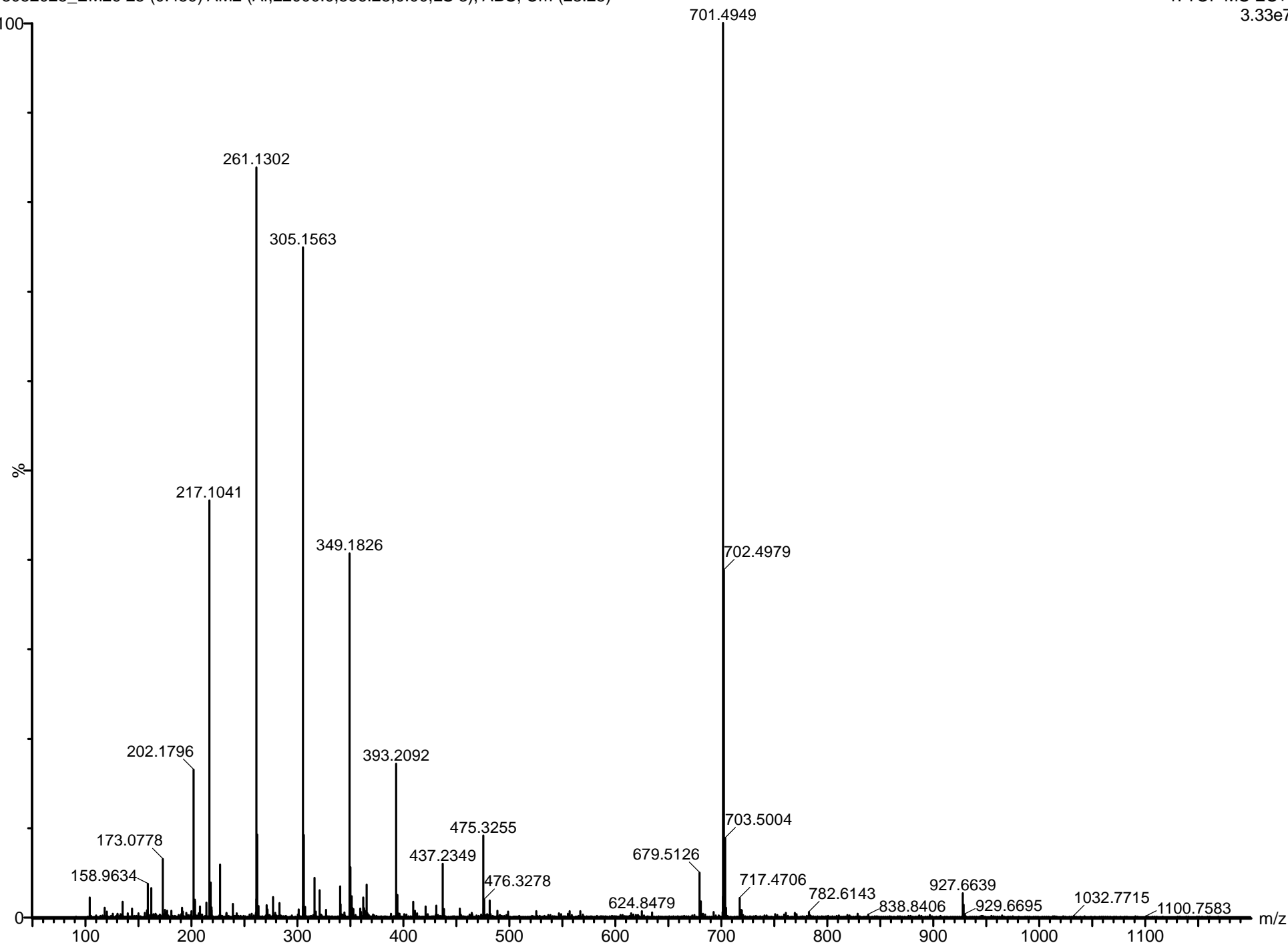

Supplement: S1 Data — Electrospray ionisation time of flight mass spectrometry (ESI-TOF MS, positive mode) spectra of the dengue cohort and ESI-TOF at different retention times. The spectra display the relative abundance (%) of detected ions across the m/z range. Prominent peaks corresponding to major ionised species are indicated. Variation in spectral profiles between retention times reflects the differences in compound composition and ionisation patterns within the sample. Data were acquired under identical instrumental conditions and are presented as representative scans. (ZIP) [file pntd.0014327.s003.zip › EM COMPLETE SAMPLES SPECTRUM/EM29 SPECTRUM RT 0.459.pdf]

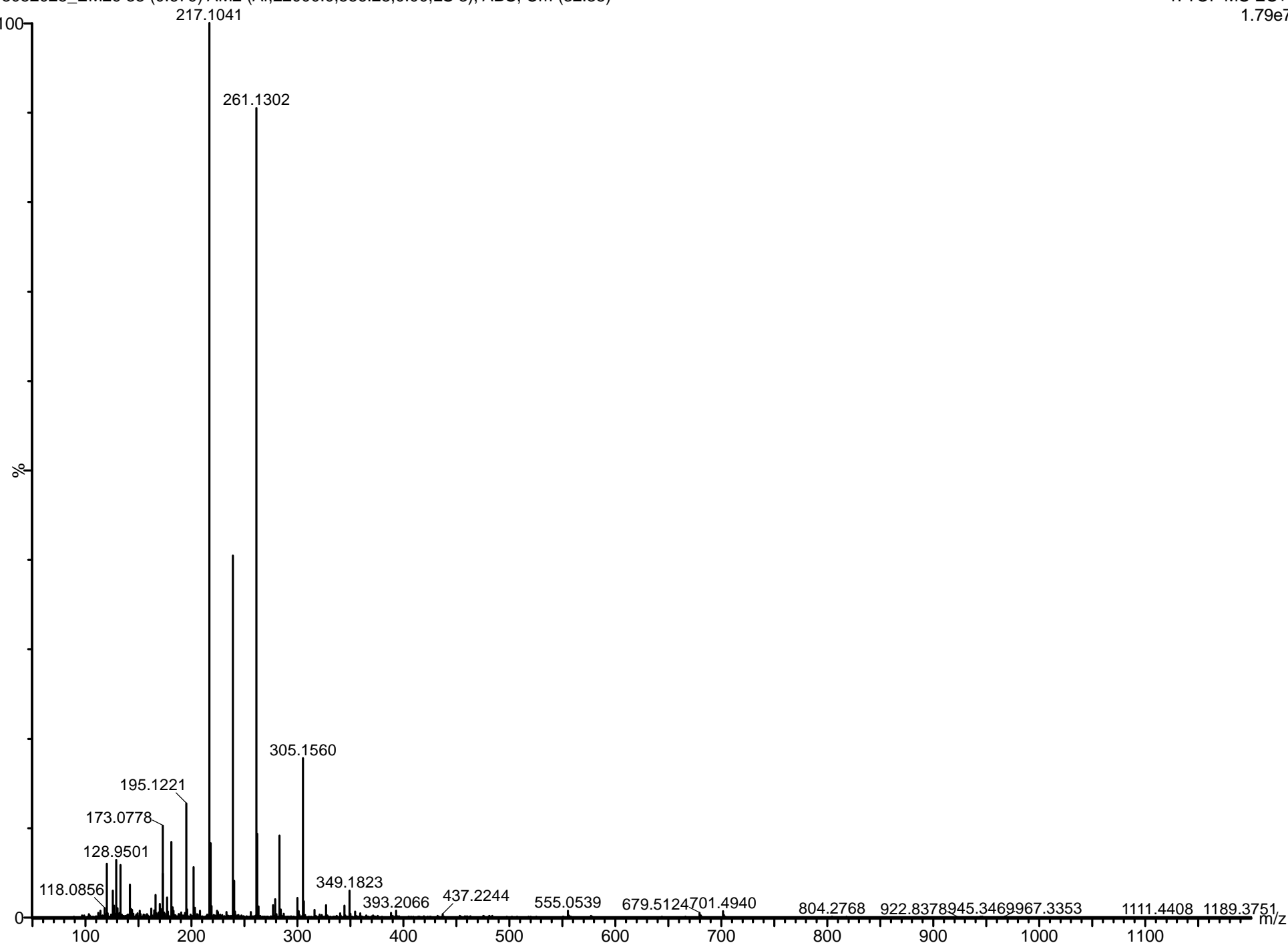

Supplement: S1 Data — Electrospray ionisation time of flight mass spectrometry (ESI-TOF MS, positive mode) spectra of the dengue cohort and ESI-TOF at different retention times. The spectra display the relative abundance (%) of detected ions across the m/z range. Prominent peaks corresponding to major ionised species are indicated. Variation in spectral profiles between retention times reflects the differences in compound composition and ionisation patterns within the sample. Data were acquired under identical instrumental conditions and are presented as representative scans. (ZIP) [file pntd.0014327.s003.zip › EM COMPLETE SAMPLES SPECTRUM/EM29 SPECTRUM RT 0.679.pdf]

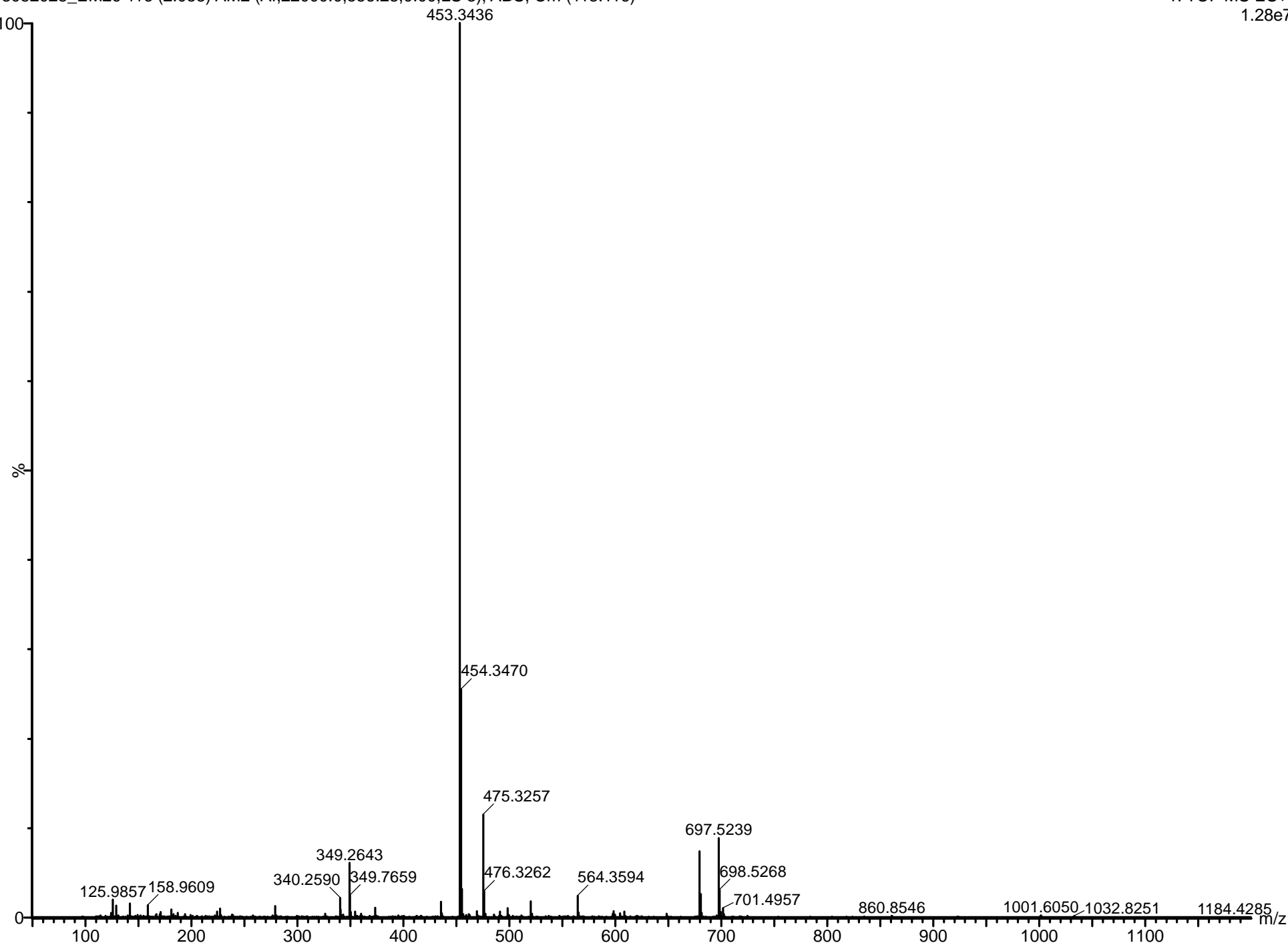

Supplement: S1 Data — Electrospray ionisation time of flight mass spectrometry (ESI-TOF MS, positive mode) spectra of the dengue cohort and ESI-TOF at different retention times. The spectra display the relative abundance (%) of detected ions across the m/z range. Prominent peaks corresponding to major ionised species are indicated. Variation in spectral profiles between retention times reflects the differences in compound composition and ionisation patterns within the sample. Data were acquired under identical instrumental conditions and are presented as representative scans. (ZIP) [file pntd.0014327.s003.zip › EM COMPLETE SAMPLES SPECTRUM/EM29 SPECTRUM RT 2.058.pdf]

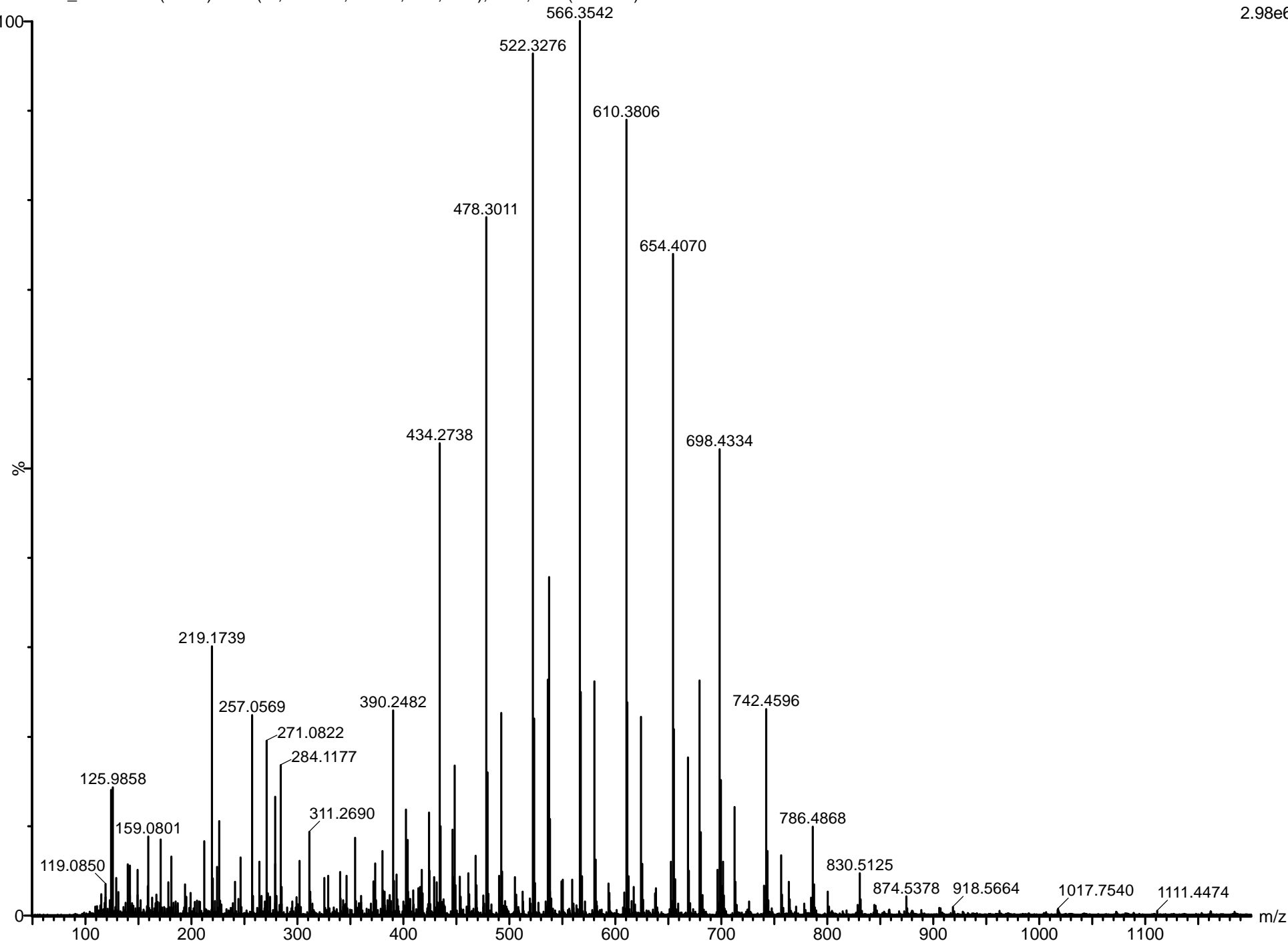

Supplement: S1 Data — Electrospray ionisation time of flight mass spectrometry (ESI-TOF MS, positive mode) spectra of the dengue cohort and ESI-TOF at different retention times. The spectra display the relative abundance (%) of detected ions across the m/z range. Prominent peaks corresponding to major ionised species are indicated. Variation in spectral profiles between retention times reflects the differences in compound composition and ionisation patterns within the sample. Data were acquired under identical instrumental conditions and are presented as representative scans. (ZIP) [file pntd.0014327.s003.zip › EM COMPLETE SAMPLES SPECTRUM/EM29 SPECTRUM RT 2.565.pdf]

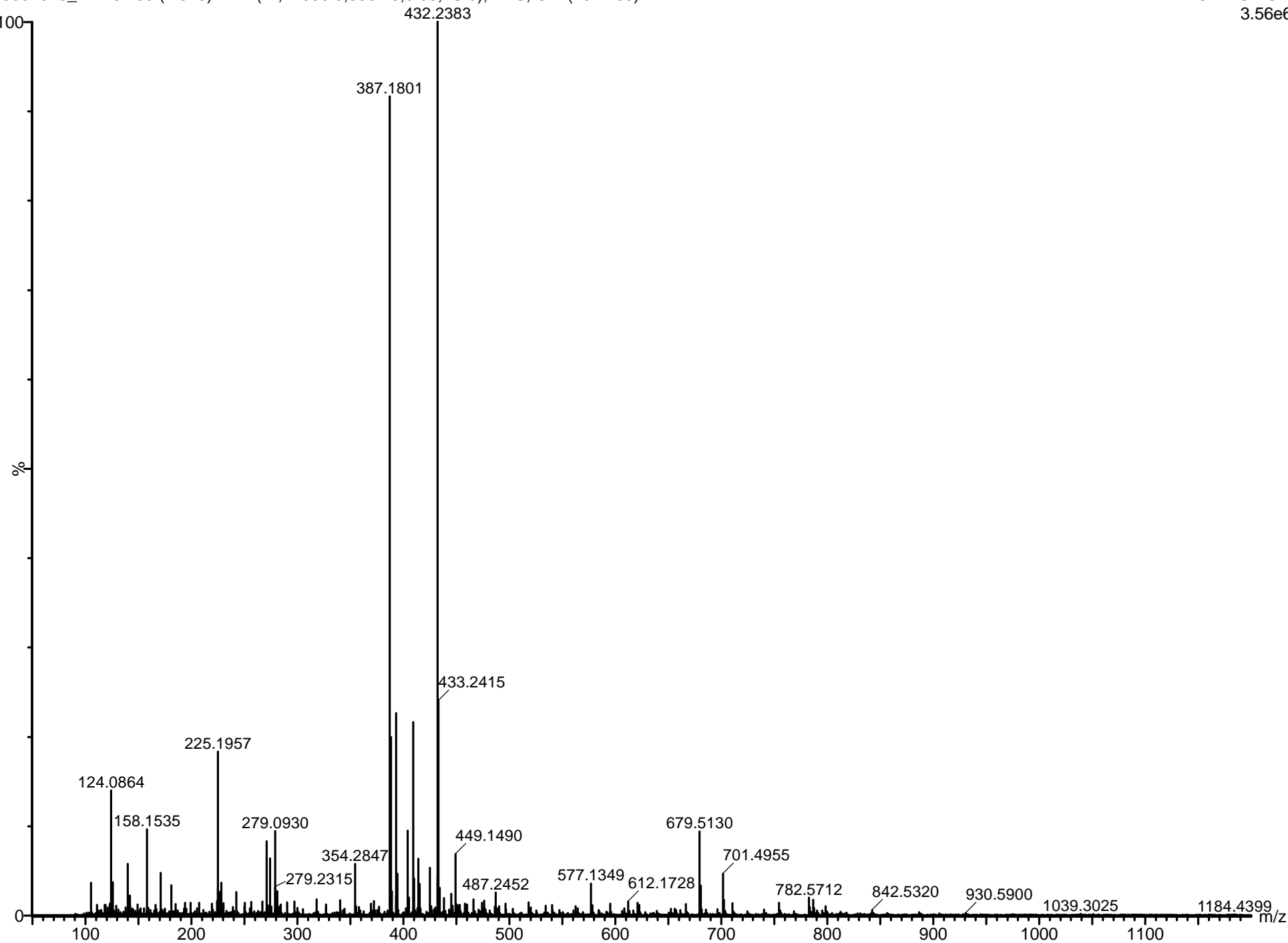

Supplement: S1 Data — Electrospray ionisation time of flight mass spectrometry (ESI-TOF MS, positive mode) spectra of the dengue cohort and ESI-TOF at different retention times. The spectra display the relative abundance (%) of detected ions across the m/z range. Prominent peaks corresponding to major ionised species are indicated. Variation in spectral profiles between retention times reflects the differences in compound composition and ionisation patterns within the sample. Data were acquired under identical instrumental conditions and are presented as representative scans. (ZIP) [file pntd.0014327.s003.zip › EM COMPLETE SAMPLES SPECTRUM/EM29 SPECTRUM RT 2.873.pdf]

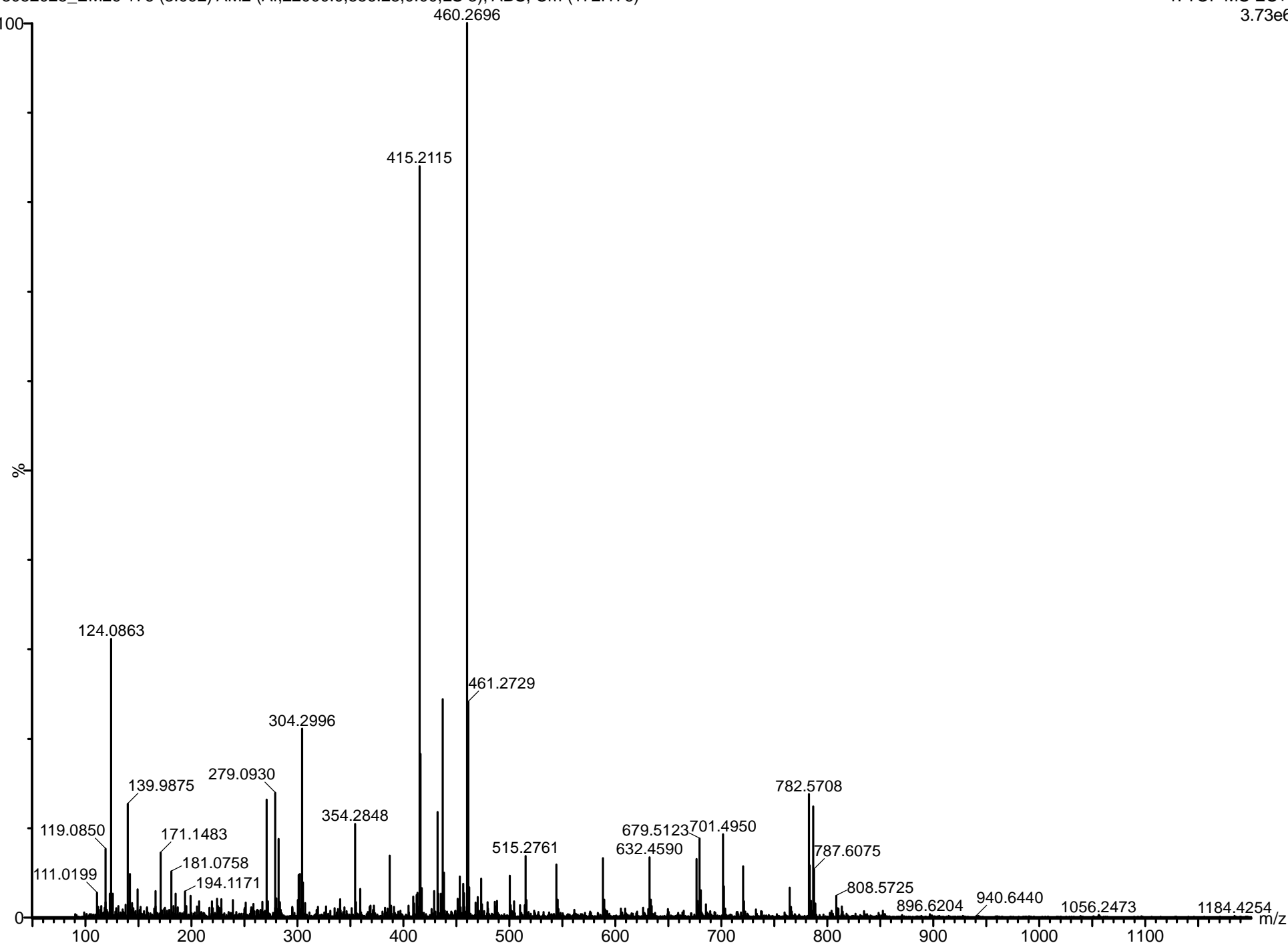

Supplement: S1 Data — Electrospray ionisation time of flight mass spectrometry (ESI-TOF MS, positive mode) spectra of the dengue cohort and ESI-TOF at different retention times. The spectra display the relative abundance (%) of detected ions across the m/z range. Prominent peaks corresponding to major ionised species are indicated. Variation in spectral profiles between retention times reflects the differences in compound composition and ionisation patterns within the sample. Data were acquired under identical instrumental conditions and are presented as representative scans. (ZIP) [file pntd.0014327.s003.zip › EM COMPLETE SAMPLES SPECTRUM/EM29 SPECTRUM RT 3.092.pdf]

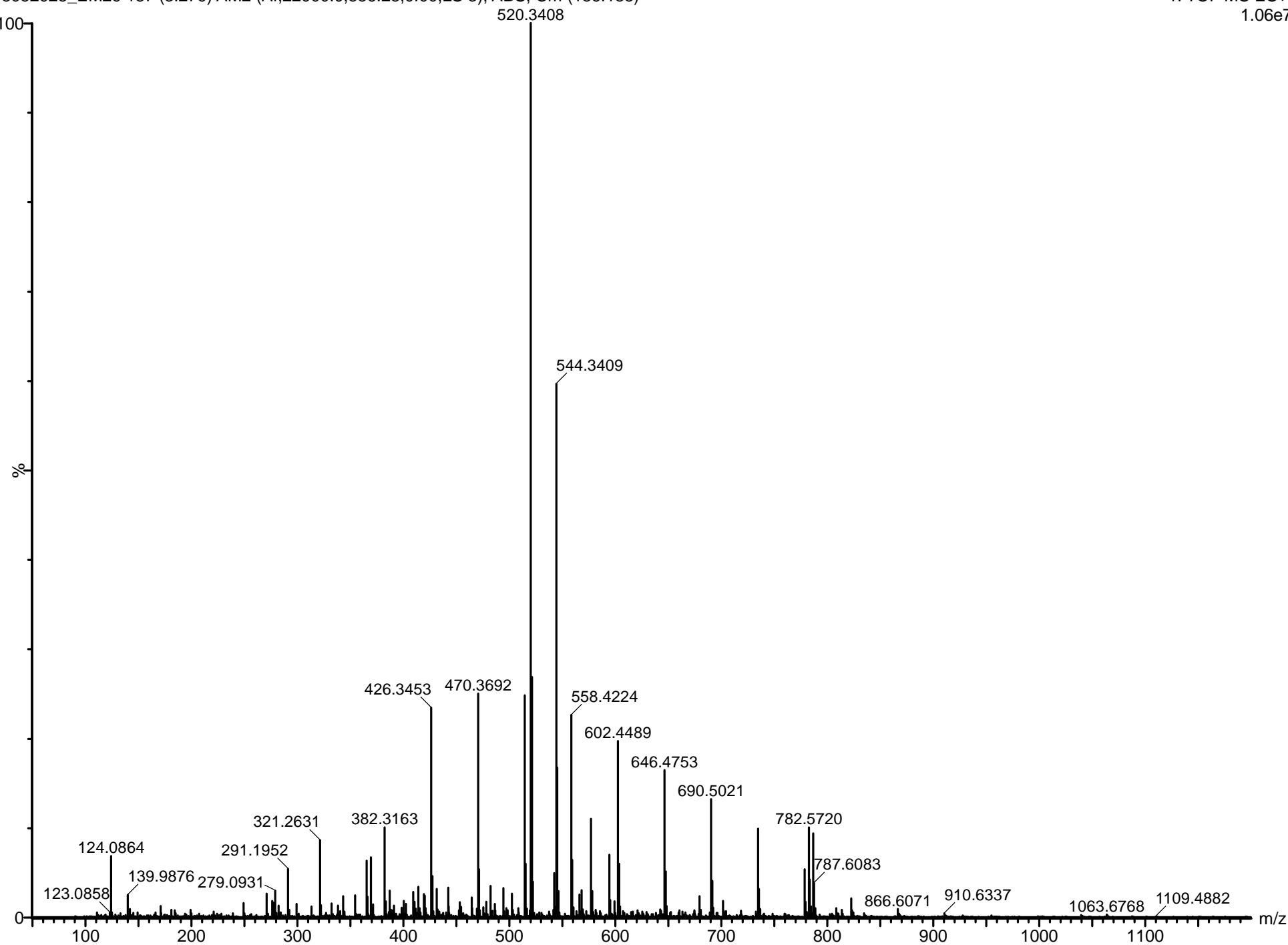

Supplement: S1 Data — Electrospray ionisation time of flight mass spectrometry (ESI-TOF MS, positive mode) spectra of the dengue cohort and ESI-TOF at different retention times. The spectra display the relative abundance (%) of detected ions across the m/z range. Prominent peaks corresponding to major ionised species are indicated. Variation in spectral profiles between retention times reflects the differences in compound composition and ionisation patterns within the sample. Data were acquired under identical instrumental conditions and are presented as representative scans. (ZIP) [file pntd.0014327.s003.zip › EM COMPLETE SAMPLES SPECTRUM/EM29 SPECTRUM RT 3.279.pdf]

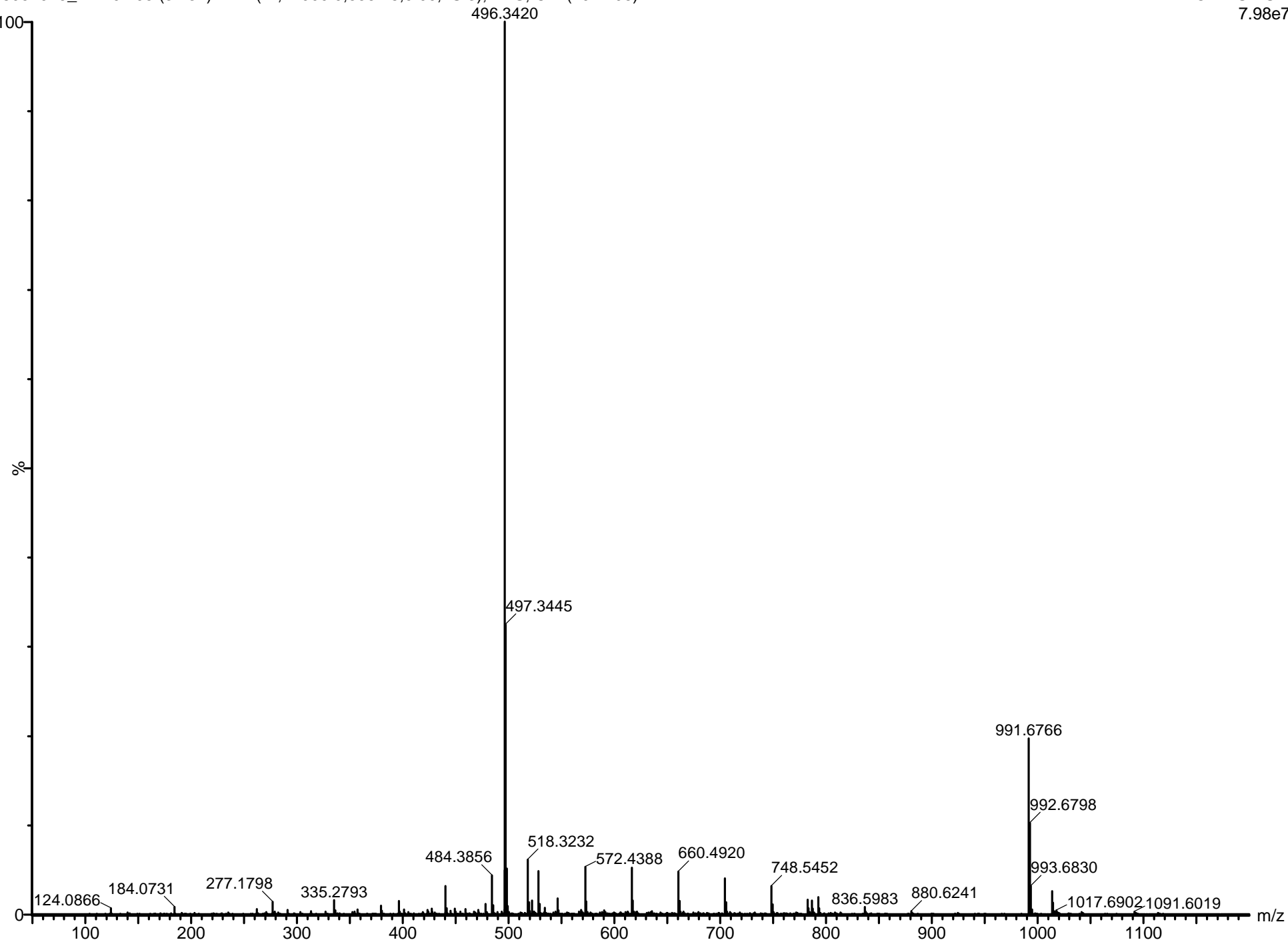

Supplement: S1 Data — Electrospray ionisation time of flight mass spectrometry (ESI-TOF MS, positive mode) spectra of the dengue cohort and ESI-TOF at different retention times. The spectra display the relative abundance (%) of detected ions across the m/z range. Prominent peaks corresponding to major ionised species are indicated. Variation in spectral profiles between retention times reflects the differences in compound composition and ionisation patterns within the sample. Data were acquired under identical instrumental conditions and are presented as representative scans. (ZIP) [file pntd.0014327.s003.zip › EM COMPLETE SAMPLES SPECTRUM/EM29 SPECTRUM RT 3.434.pdf]

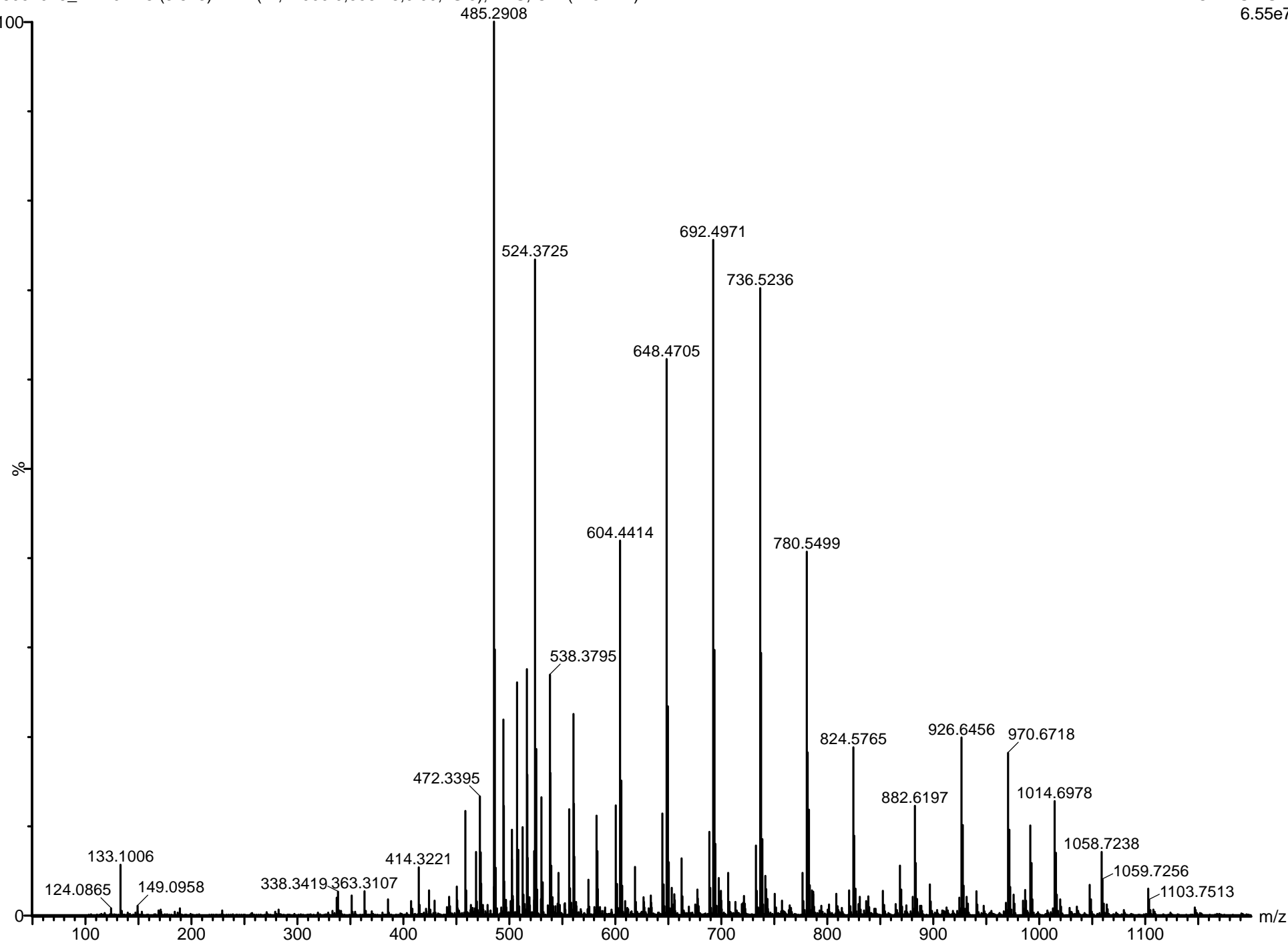

Supplement: S1 Data — Electrospray ionisation time of flight mass spectrometry (ESI-TOF MS, positive mode) spectra of the dengue cohort and ESI-TOF at different retention times. The spectra display the relative abundance (%) of detected ions across the m/z range. Prominent peaks corresponding to major ionised species are indicated. Variation in spectral profiles between retention times reflects the differences in compound composition and ionisation patterns within the sample. Data were acquired under identical instrumental conditions and are presented as representative scans. (ZIP) [file pntd.0014327.s003.zip › EM COMPLETE SAMPLES SPECTRUM/EM29 SPECTRUM RT 3.823.pdf]

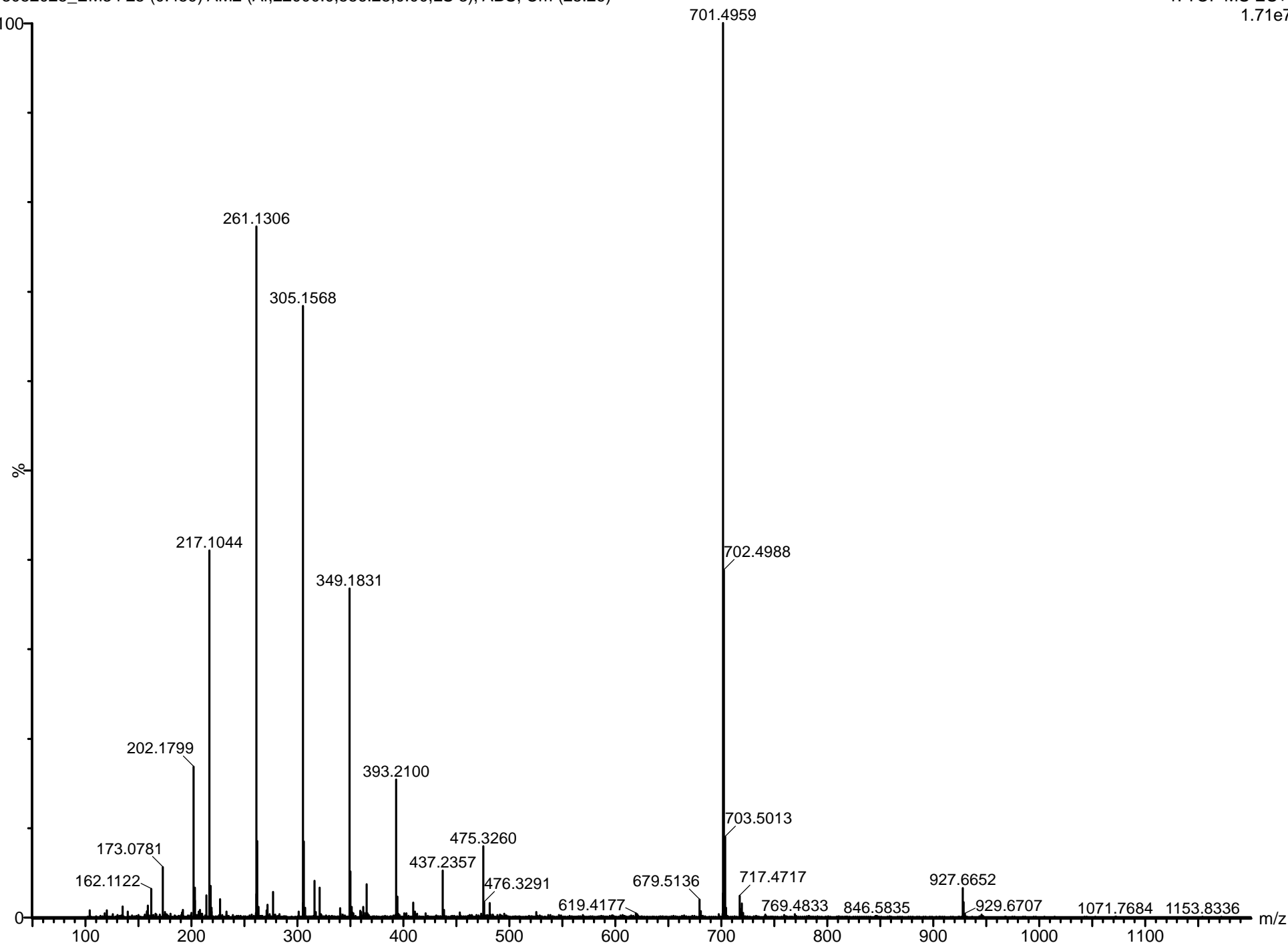

Supplement: S1 Data — Electrospray ionisation time of flight mass spectrometry (ESI-TOF MS, positive mode) spectra of the dengue cohort and ESI-TOF at different retention times. The spectra display the relative abundance (%) of detected ions across the m/z range. Prominent peaks corresponding to major ionised species are indicated. Variation in spectral profiles between retention times reflects the differences in compound composition and ionisation patterns within the sample. Data were acquired under identical instrumental conditions and are presented as representative scans. (ZIP) [file pntd.0014327.s003.zip › EM COMPLETE SAMPLES SPECTRUM/EM34 SPECTRUM RT 0.459.pdf]

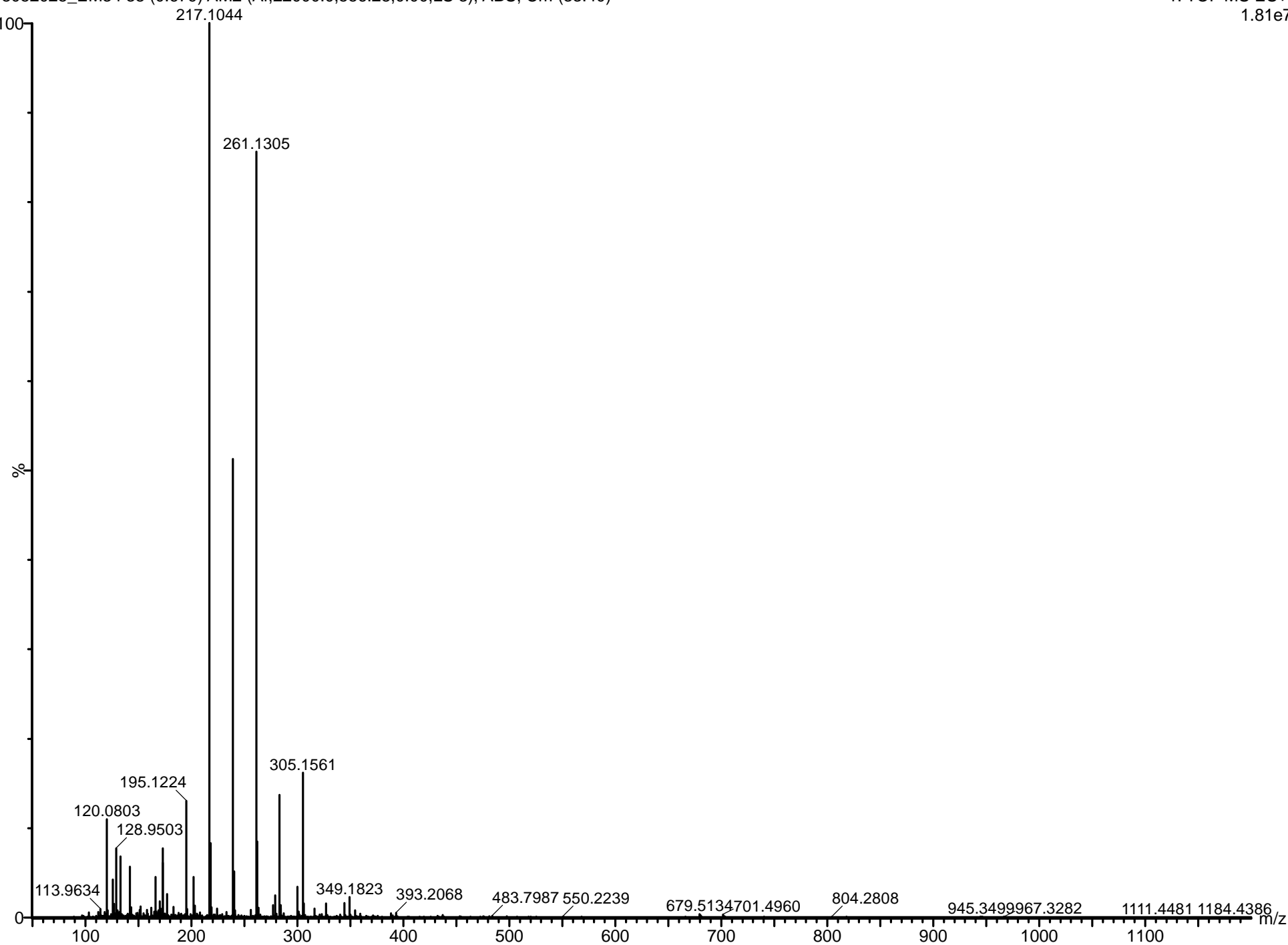

Supplement: S1 Data — Electrospray ionisation time of flight mass spectrometry (ESI-TOF MS, positive mode) spectra of the dengue cohort and ESI-TOF at different retention times. The spectra display the relative abundance (%) of detected ions across the m/z range. Prominent peaks corresponding to major ionised species are indicated. Variation in spectral profiles between retention times reflects the differences in compound composition and ionisation patterns within the sample. Data were acquired under identical instrumental conditions and are presented as representative scans. (ZIP) [file pntd.0014327.s003.zip › EM COMPLETE SAMPLES SPECTRUM/EM34 SPECTRUM RT 0.679.pdf]

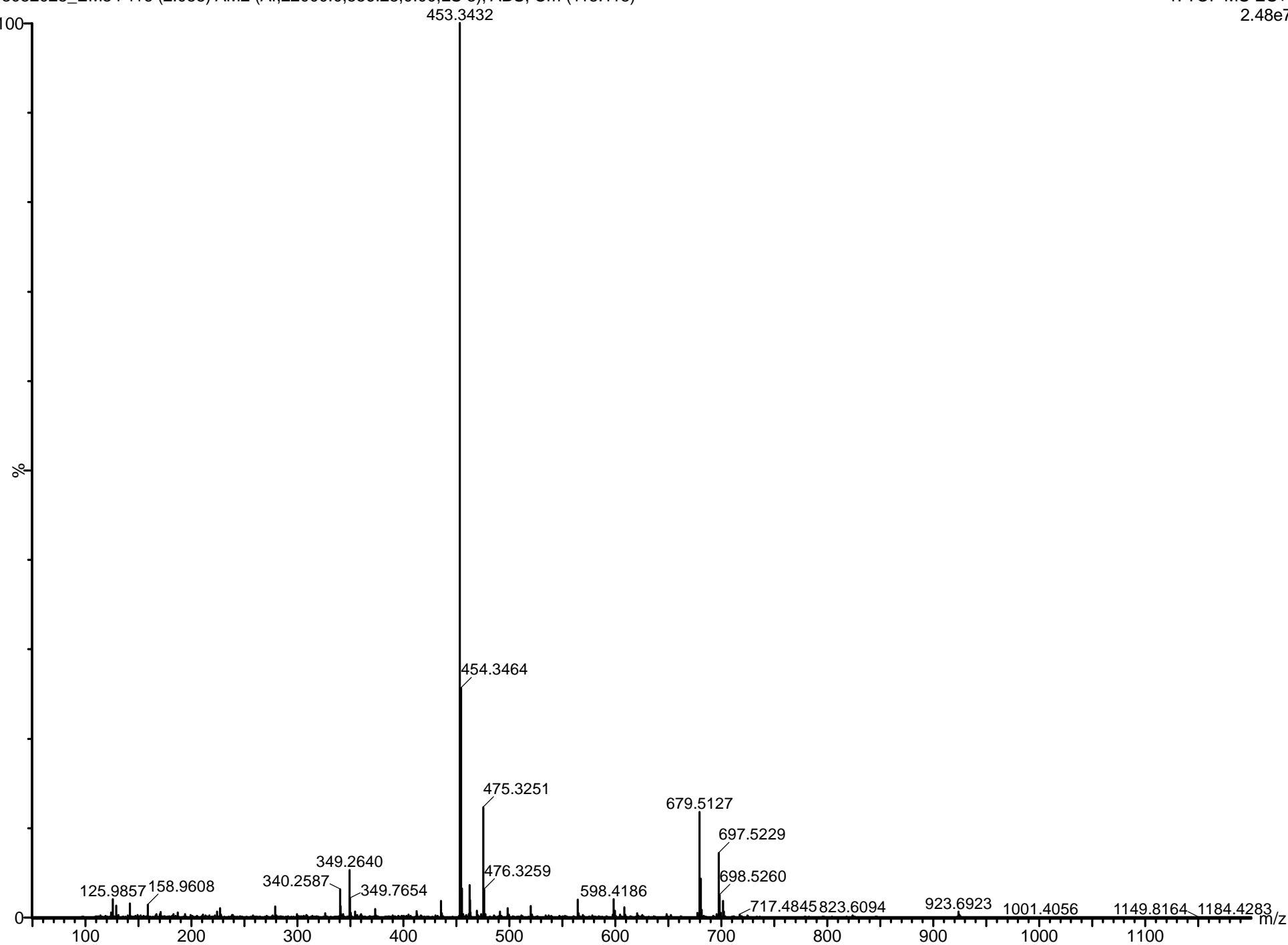

Supplement: S1 Data — Electrospray ionisation time of flight mass spectrometry (ESI-TOF MS, positive mode) spectra of the dengue cohort and ESI-TOF at different retention times. The spectra display the relative abundance (%) of detected ions across the m/z range. Prominent peaks corresponding to major ionised species are indicated. Variation in spectral profiles between retention times reflects the differences in compound composition and ionisation patterns within the sample. Data were acquired under identical instrumental conditions and are presented as representative scans. (ZIP) [file pntd.0014327.s003.zip › EM COMPLETE SAMPLES SPECTRUM/EM34 SPECTRUM RT 2.058.pdf]

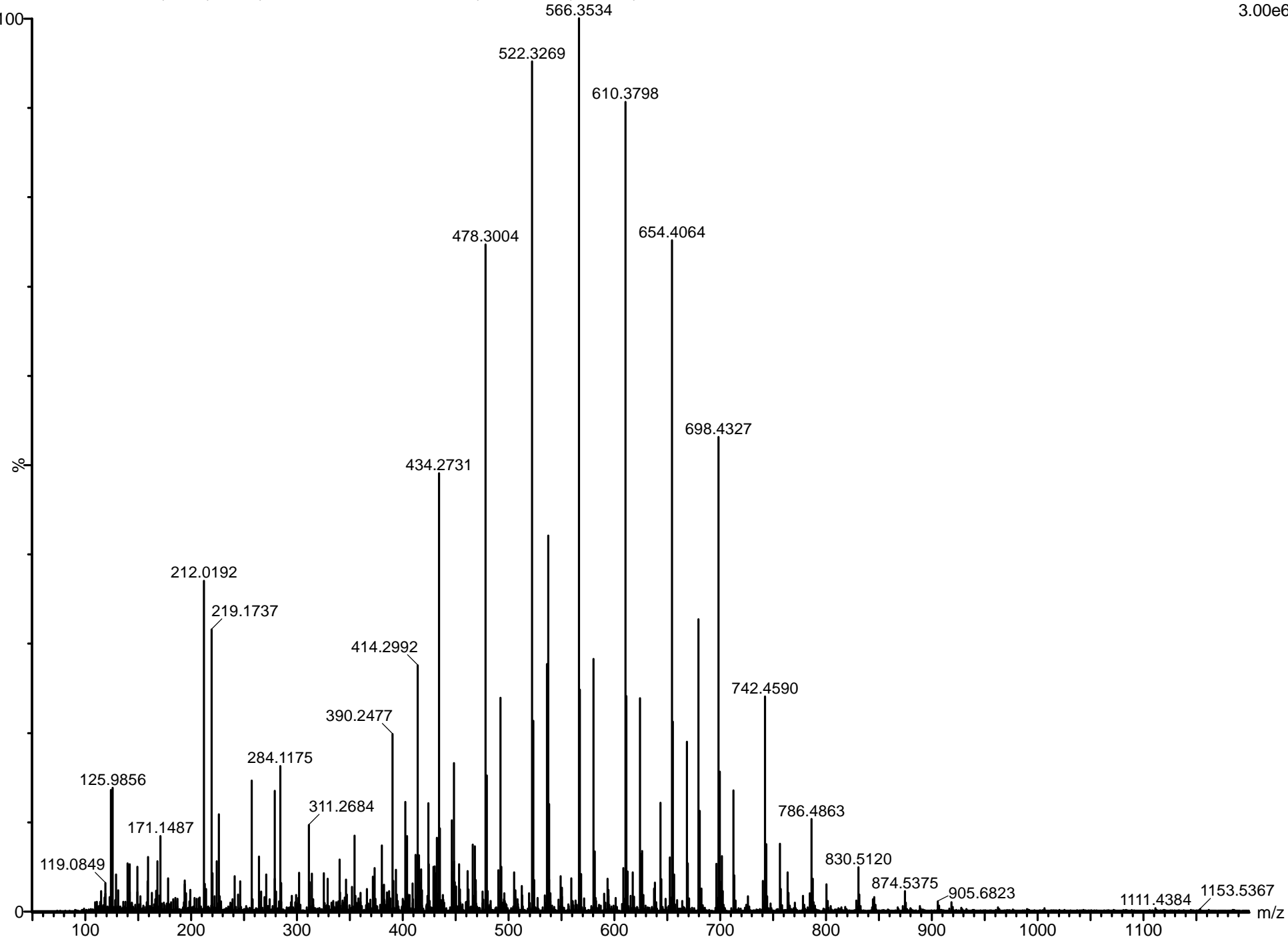

Supplement: S1 Data — Electrospray ionisation time of flight mass spectrometry (ESI-TOF MS, positive mode) spectra of the dengue cohort and ESI-TOF at different retention times. The spectra display the relative abundance (%) of detected ions across the m/z range. Prominent peaks corresponding to major ionised species are indicated. Variation in spectral profiles between retention times reflects the differences in compound composition and ionisation patterns within the sample. Data were acquired under identical instrumental conditions and are presented as representative scans. (ZIP) [file pntd.0014327.s003.zip › EM COMPLETE SAMPLES SPECTRUM/EM34 SPECTRUM RT 2.582.pdf]

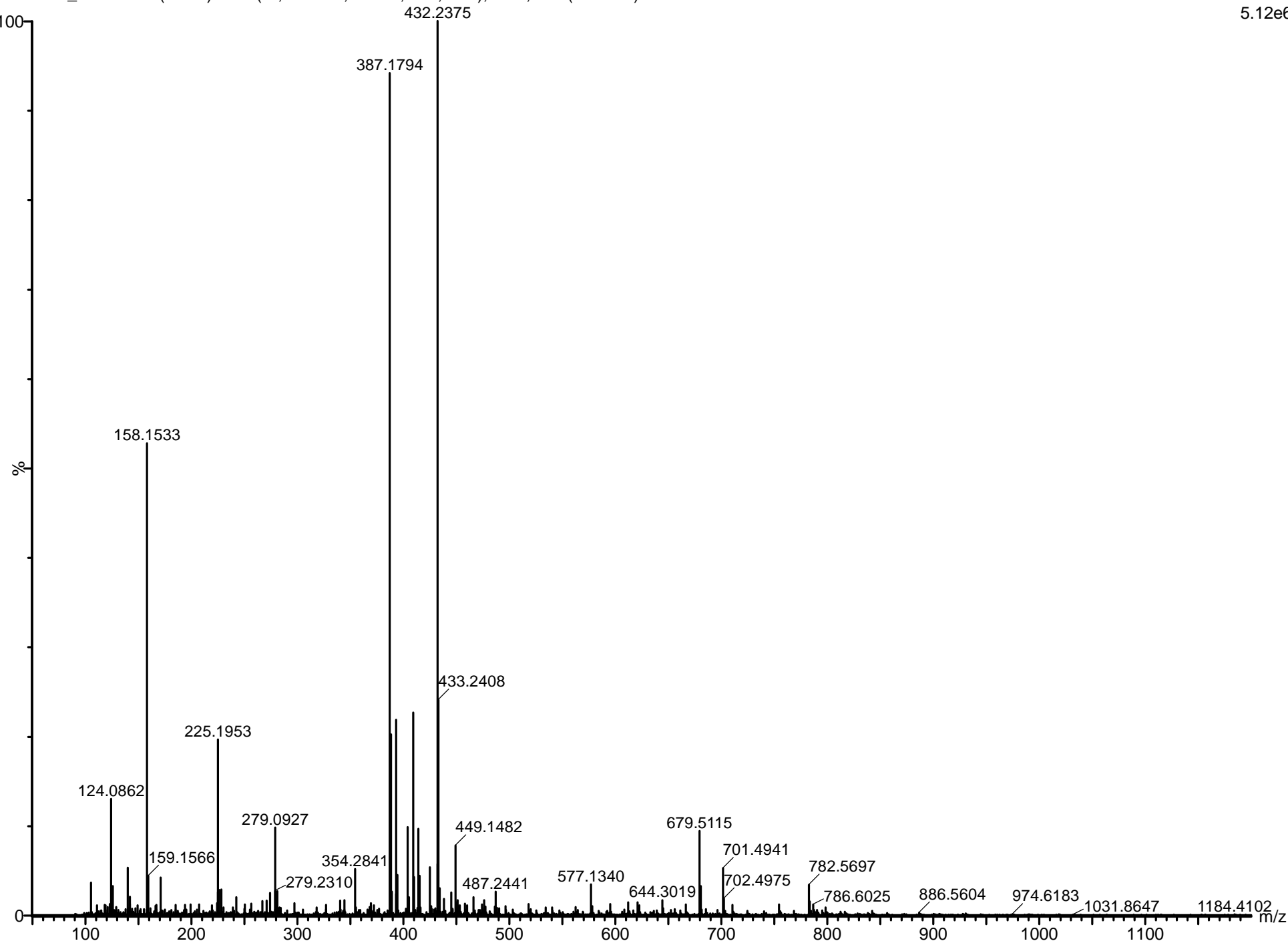

Supplement: S1 Data — Electrospray ionisation time of flight mass spectrometry (ESI-TOF MS, positive mode) spectra of the dengue cohort and ESI-TOF at different retention times. The spectra display the relative abundance (%) of detected ions across the m/z range. Prominent peaks corresponding to major ionised species are indicated. Variation in spectral profiles between retention times reflects the differences in compound composition and ionisation patterns within the sample. Data were acquired under identical instrumental conditions and are presented as representative scans. (ZIP) [file pntd.0014327.s003.zip › EM COMPLETE SAMPLES SPECTRUM/EM34 SPECTRUM RT 2.873.pdf]

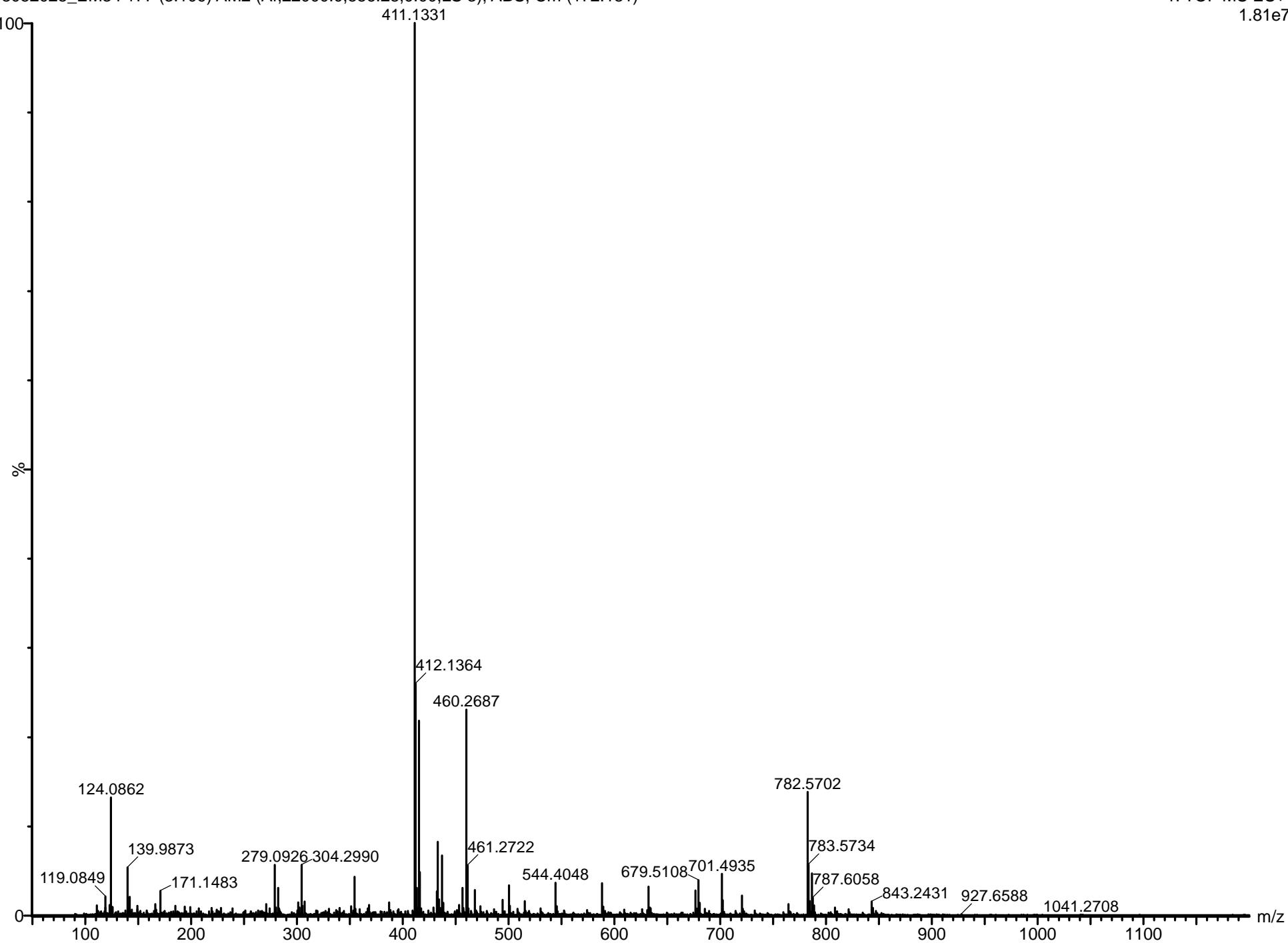

Supplement: S1 Data — Electrospray ionisation time of flight mass spectrometry (ESI-TOF MS, positive mode) spectra of the dengue cohort and ESI-TOF at different retention times. The spectra display the relative abundance (%) of detected ions across the m/z range. Prominent peaks corresponding to major ionised species are indicated. Variation in spectral profiles between retention times reflects the differences in compound composition and ionisation patterns within the sample. Data were acquired under identical instrumental conditions and are presented as representative scans. (ZIP) [file pntd.0014327.s003.zip › EM COMPLETE SAMPLES SPECTRUM/EM34 SPECTRUM RT 3.109.pdf]

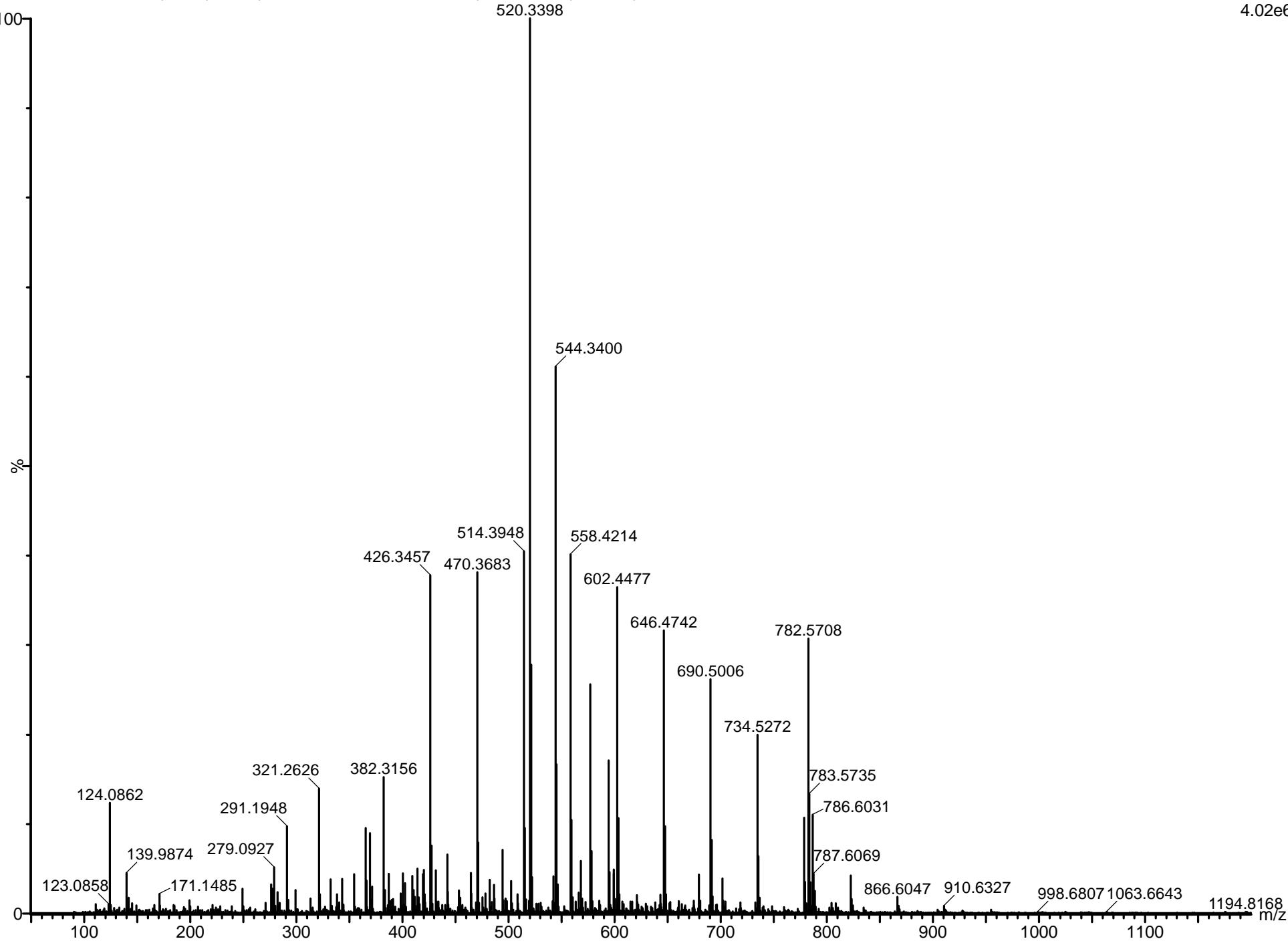

Supplement: S1 Data — Electrospray ionisation time of flight mass spectrometry (ESI-TOF MS, positive mode) spectra of the dengue cohort and ESI-TOF at different retention times. The spectra display the relative abundance (%) of detected ions across the m/z range. Prominent peaks corresponding to major ionised species are indicated. Variation in spectral profiles between retention times reflects the differences in compound composition and ionisation patterns within the sample. Data were acquired under identical instrumental conditions and are presented as representative scans. (ZIP) [file pntd.0014327.s003.zip › EM COMPLETE SAMPLES SPECTRUM/EM34 SPECTRUM RT 3.279.pdf]

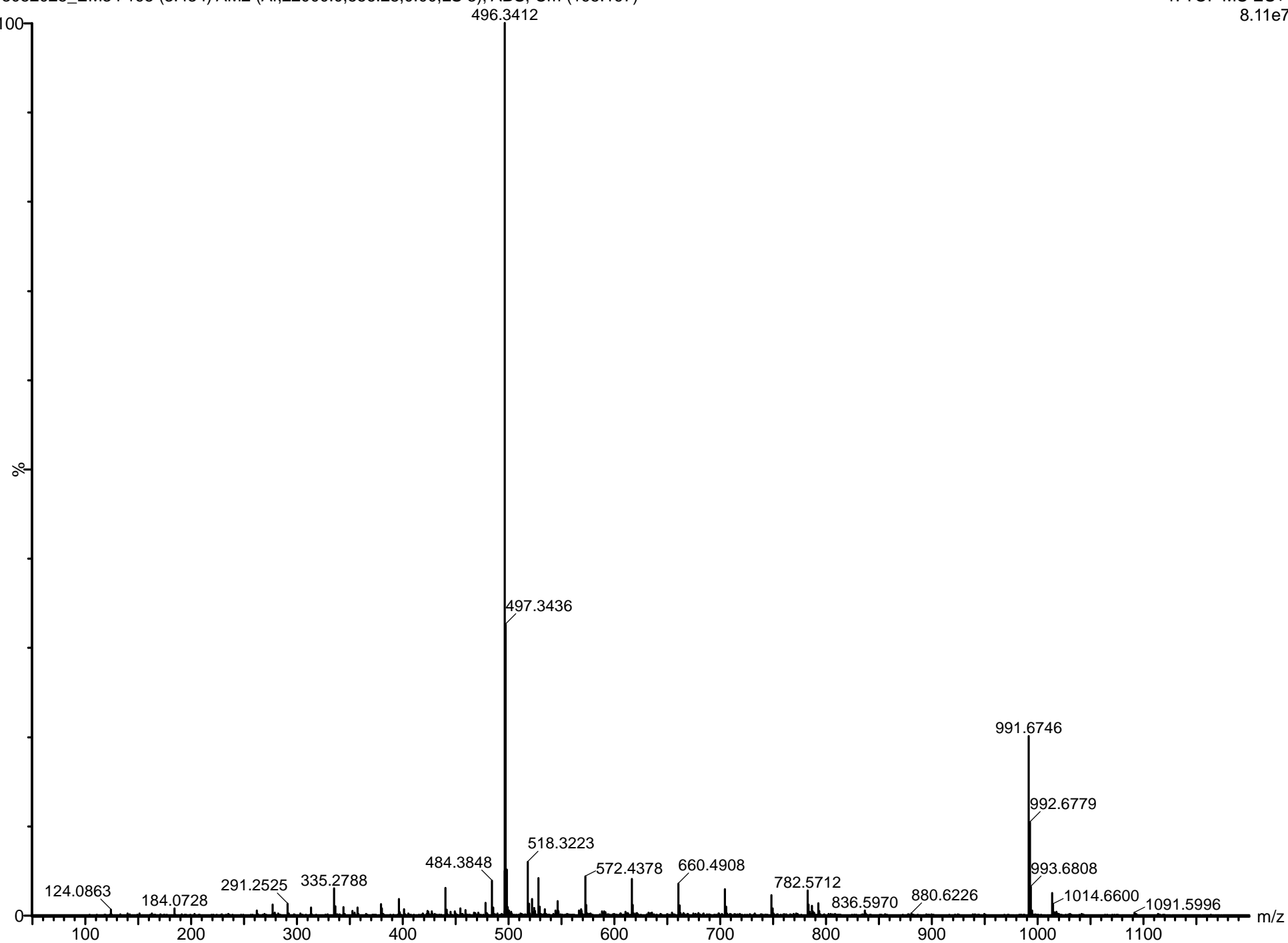

Supplement: S1 Data — Electrospray ionisation time of flight mass spectrometry (ESI-TOF MS, positive mode) spectra of the dengue cohort and ESI-TOF at different retention times. The spectra display the relative abundance (%) of detected ions across the m/z range. Prominent peaks corresponding to major ionised species are indicated. Variation in spectral profiles between retention times reflects the differences in compound composition and ionisation patterns within the sample. Data were acquired under identical instrumental conditions and are presented as representative scans. (ZIP) [file pntd.0014327.s003.zip › EM COMPLETE SAMPLES SPECTRUM/EM34 SPECTRUM RT 3.434.pdf]

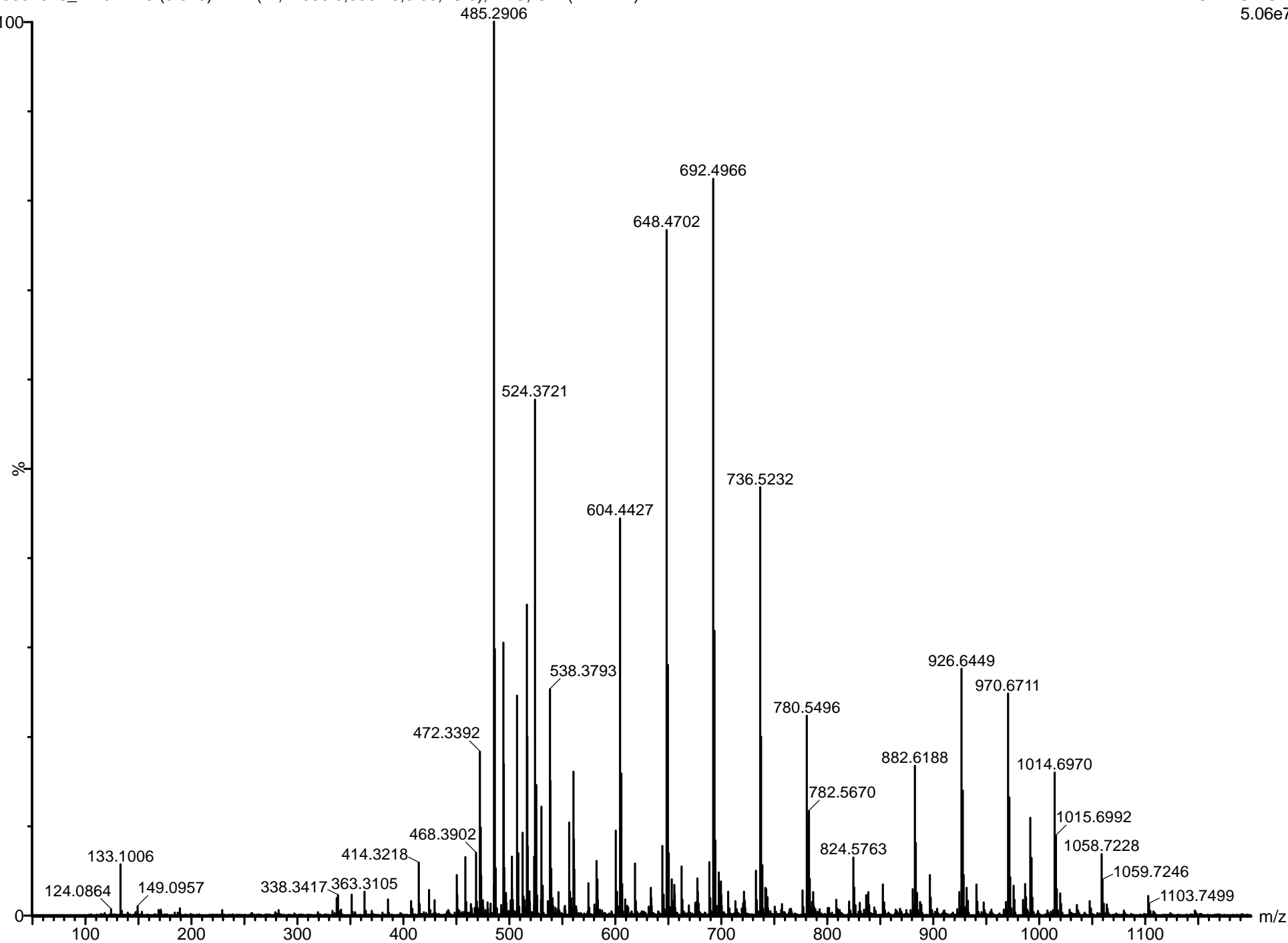

Supplement: S1 Data — Electrospray ionisation time of flight mass spectrometry (ESI-TOF MS, positive mode) spectra of the dengue cohort and ESI-TOF at different retention times. The spectra display the relative abundance (%) of detected ions across the m/z range. Prominent peaks corresponding to major ionised species are indicated. Variation in spectral profiles between retention times reflects the differences in compound composition and ionisation patterns within the sample. Data were acquired under identical instrumental conditions and are presented as representative scans. (ZIP) [file pntd.0014327.s003.zip › EM COMPLETE SAMPLES SPECTRUM/EM34 SPECTRUM RT 3.823.pdf]

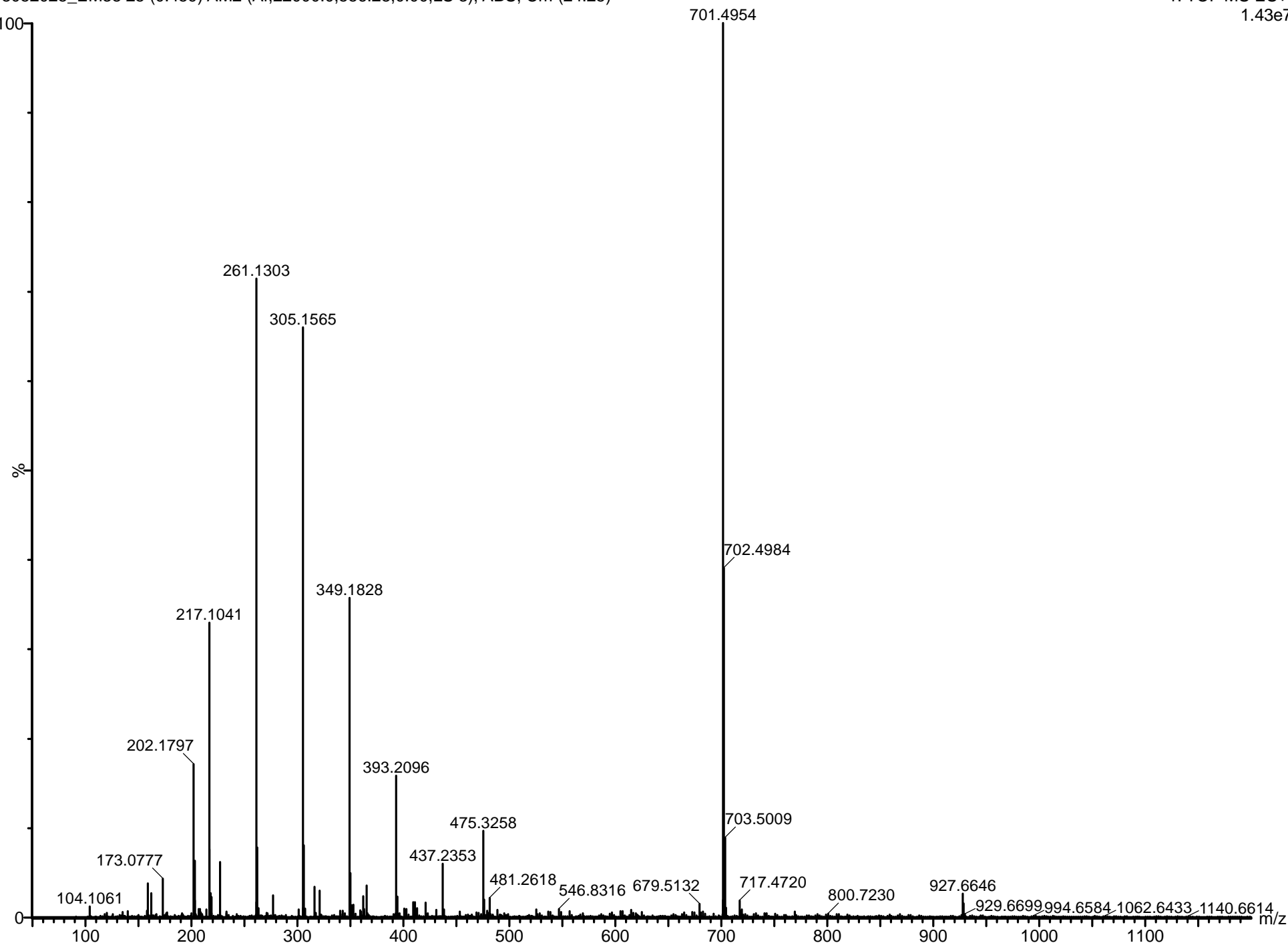

Supplement: S1 Data — Electrospray ionisation time of flight mass spectrometry (ESI-TOF MS, positive mode) spectra of the dengue cohort and ESI-TOF at different retention times. The spectra display the relative abundance (%) of detected ions across the m/z range. Prominent peaks corresponding to major ionised species are indicated. Variation in spectral profiles between retention times reflects the differences in compound composition and ionisation patterns within the sample. Data were acquired under identical instrumental conditions and are presented as representative scans. (ZIP) [file pntd.0014327.s003.zip › EM COMPLETE SAMPLES SPECTRUM/EM38 SPECTRUM RT 0.459.pdf]

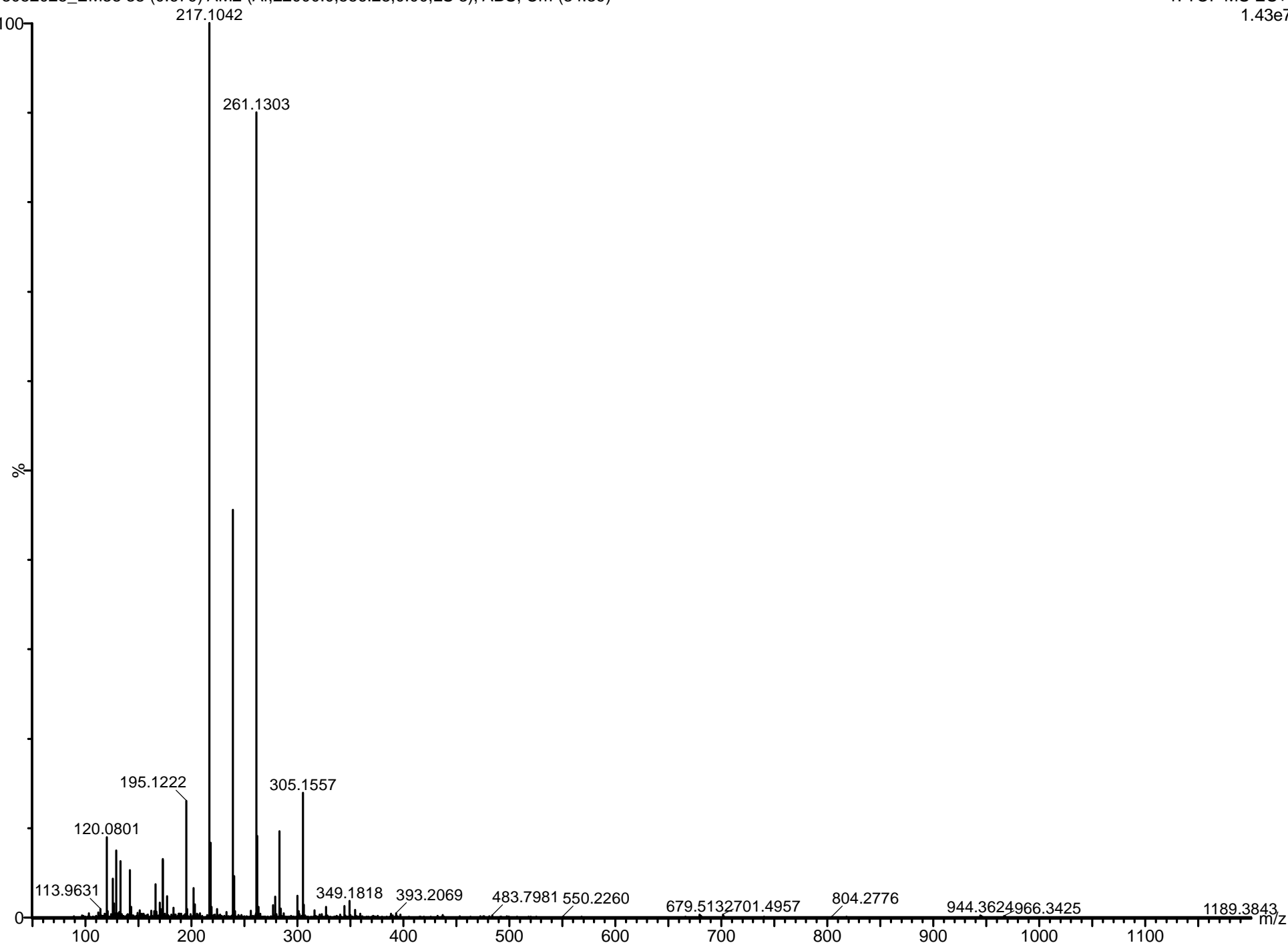

Supplement: S1 Data — Electrospray ionisation time of flight mass spectrometry (ESI-TOF MS, positive mode) spectra of the dengue cohort and ESI-TOF at different retention times. The spectra display the relative abundance (%) of detected ions across the m/z range. Prominent peaks corresponding to major ionised species are indicated. Variation in spectral profiles between retention times reflects the differences in compound composition and ionisation patterns within the sample. Data were acquired under identical instrumental conditions and are presented as representative scans. (ZIP) [file pntd.0014327.s003.zip › EM COMPLETE SAMPLES SPECTRUM/EM38 SPECTRUM RT 0.679.pdf]

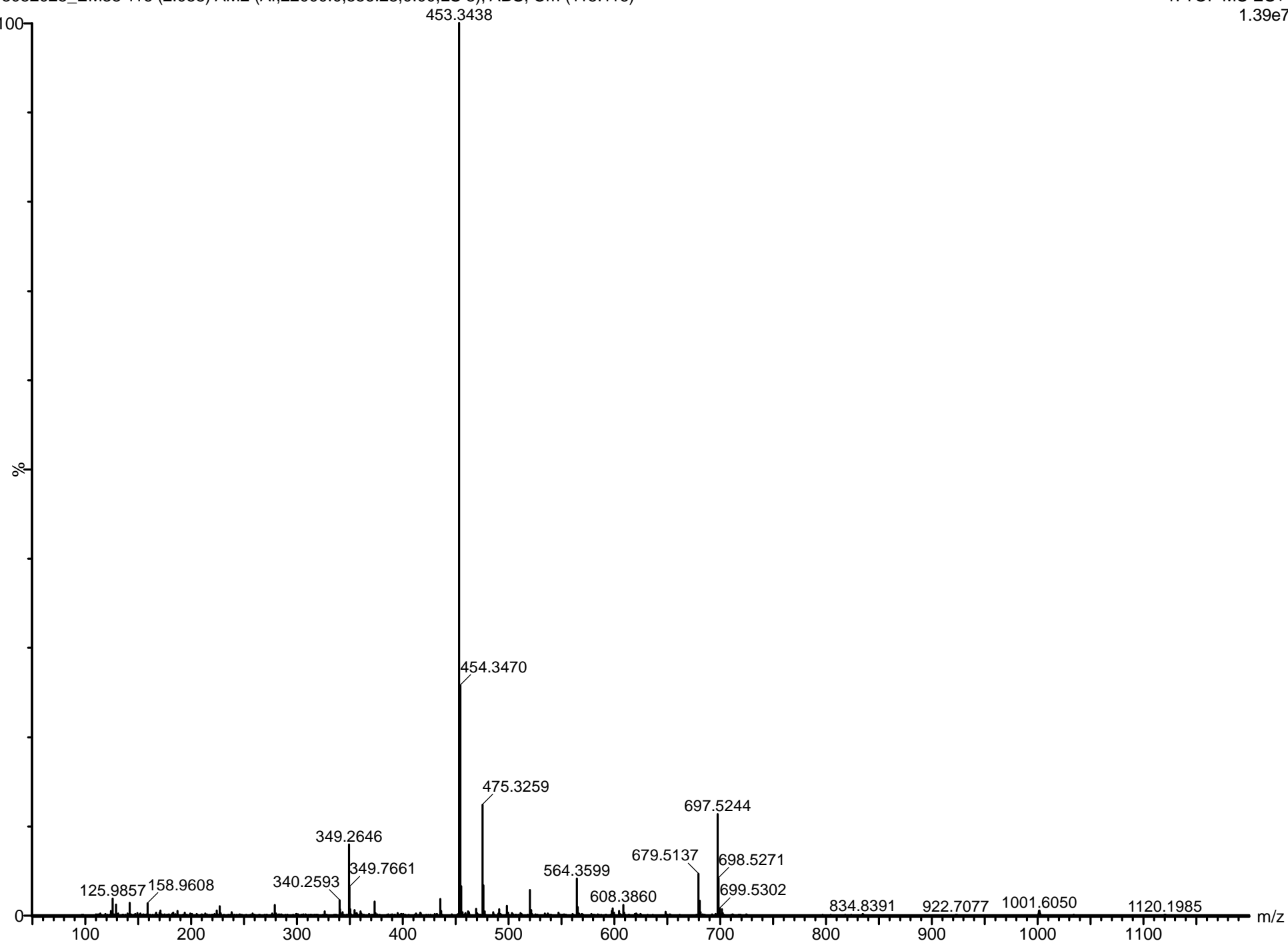

Supplement: S1 Data — Electrospray ionisation time of flight mass spectrometry (ESI-TOF MS, positive mode) spectra of the dengue cohort and ESI-TOF at different retention times. The spectra display the relative abundance (%) of detected ions across the m/z range. Prominent peaks corresponding to major ionised species are indicated. Variation in spectral profiles between retention times reflects the differences in compound composition and ionisation patterns within the sample. Data were acquired under identical instrumental conditions and are presented as representative scans. (ZIP) [file pntd.0014327.s003.zip › EM COMPLETE SAMPLES SPECTRUM/EM38 SPECTRUM RT 2.058.pdf]

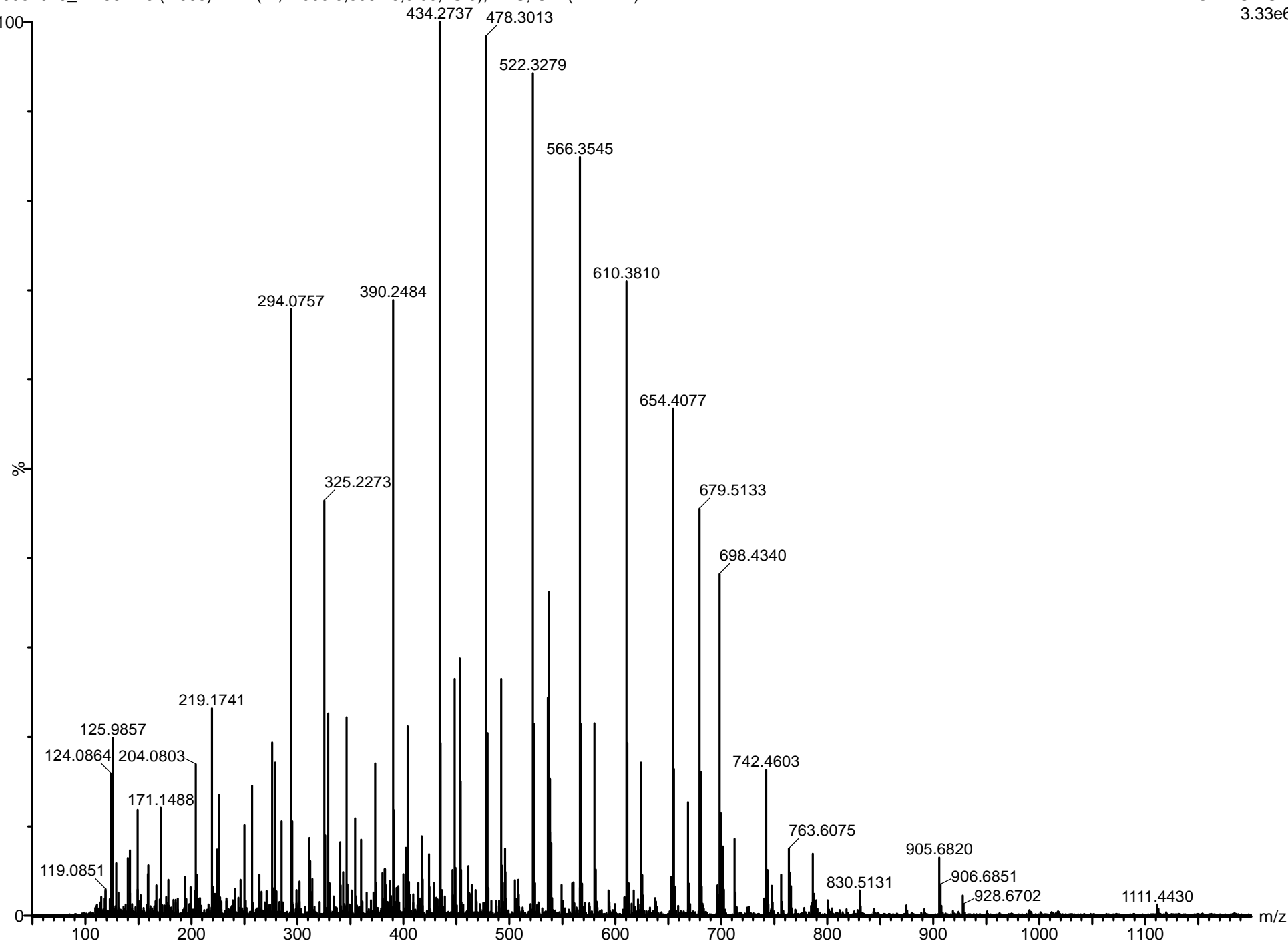

Supplement: S1 Data — Electrospray ionisation time of flight mass spectrometry (ESI-TOF MS, positive mode) spectra of the dengue cohort and ESI-TOF at different retention times. The spectra display the relative abundance (%) of detected ions across the m/z range. Prominent peaks corresponding to major ionised species are indicated. Variation in spectral profiles between retention times reflects the differences in compound composition and ionisation patterns within the sample. Data were acquired under identical instrumental conditions and are presented as representative scans. (ZIP) [file pntd.0014327.s003.zip › EM COMPLETE SAMPLES SPECTRUM/EM38 SPECTRUM RT 2.565.pdf]

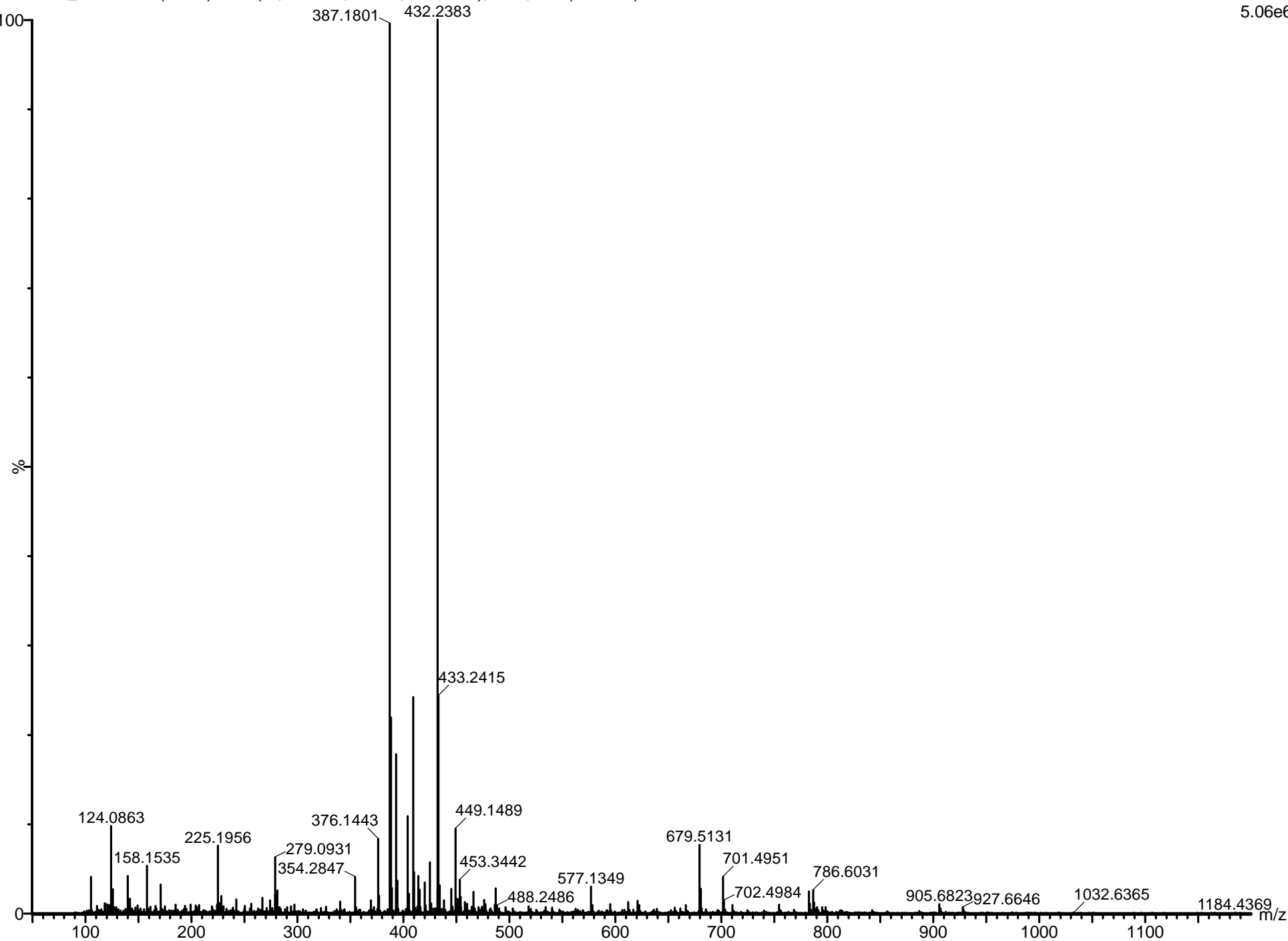

Supplement: S1 Data — Electrospray ionisation time of flight mass spectrometry (ESI-TOF MS, positive mode) spectra of the dengue cohort and ESI-TOF at different retention times. The spectra display the relative abundance (%) of detected ions across the m/z range. Prominent peaks corresponding to major ionised species are indicated. Variation in spectral profiles between retention times reflects the differences in compound composition and ionisation patterns within the sample. Data were acquired under identical instrumental conditions and are presented as representative scans. (ZIP) [file pntd.0014327.s003.zip › EM COMPLETE SAMPLES SPECTRUM/EM38 SPECTRUM RT 2.873.pdf]

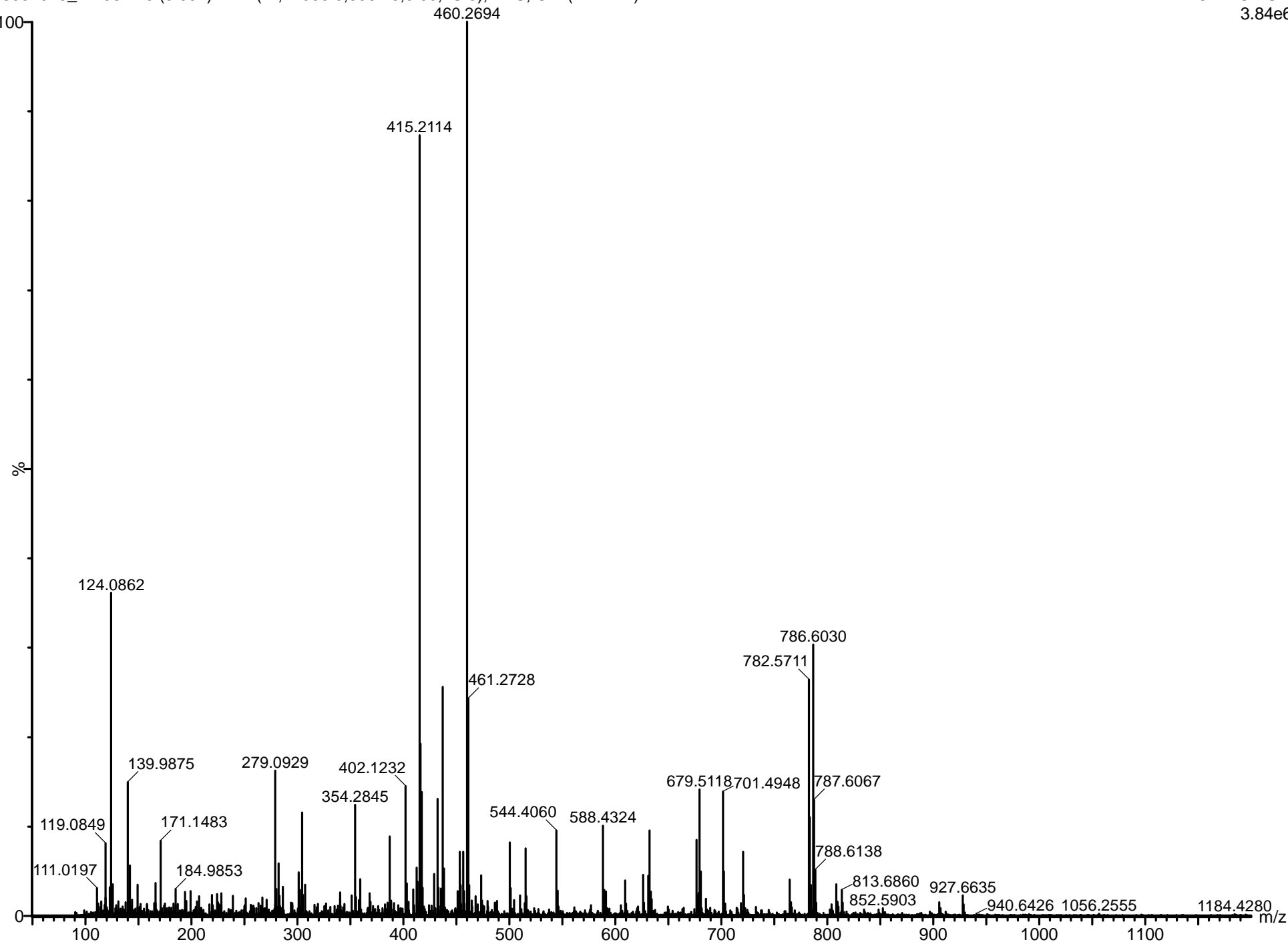

Supplement: S1 Data — Electrospray ionisation time of flight mass spectrometry (ESI-TOF MS, positive mode) spectra of the dengue cohort and ESI-TOF at different retention times. The spectra display the relative abundance (%) of detected ions across the m/z range. Prominent peaks corresponding to major ionised species are indicated. Variation in spectral profiles between retention times reflects the differences in compound composition and ionisation patterns within the sample. Data were acquired under identical instrumental conditions and are presented as representative scans. (ZIP) [file pntd.0014327.s003.zip › EM COMPLETE SAMPLES SPECTRUM/EM38 SPECTRUM RT 3.092.pdf]

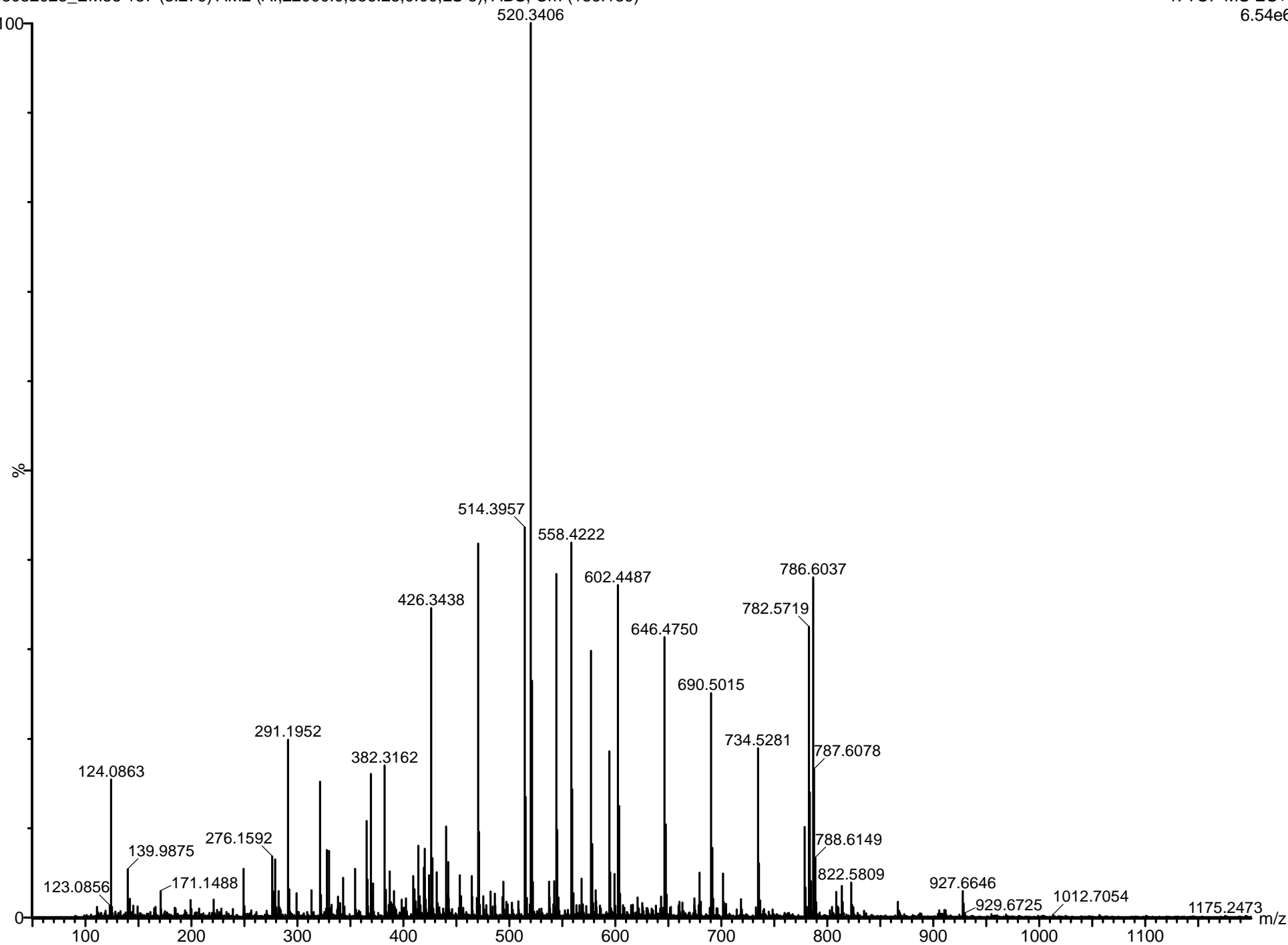

Supplement: S1 Data — Electrospray ionisation time of flight mass spectrometry (ESI-TOF MS, positive mode) spectra of the dengue cohort and ESI-TOF at different retention times. The spectra display the relative abundance (%) of detected ions across the m/z range. Prominent peaks corresponding to major ionised species are indicated. Variation in spectral profiles between retention times reflects the differences in compound composition and ionisation patterns within the sample. Data were acquired under identical instrumental conditions and are presented as representative scans. (ZIP) [file pntd.0014327.s003.zip › EM COMPLETE SAMPLES SPECTRUM/EM38 SPECTRUM RT 3.279.pdf]

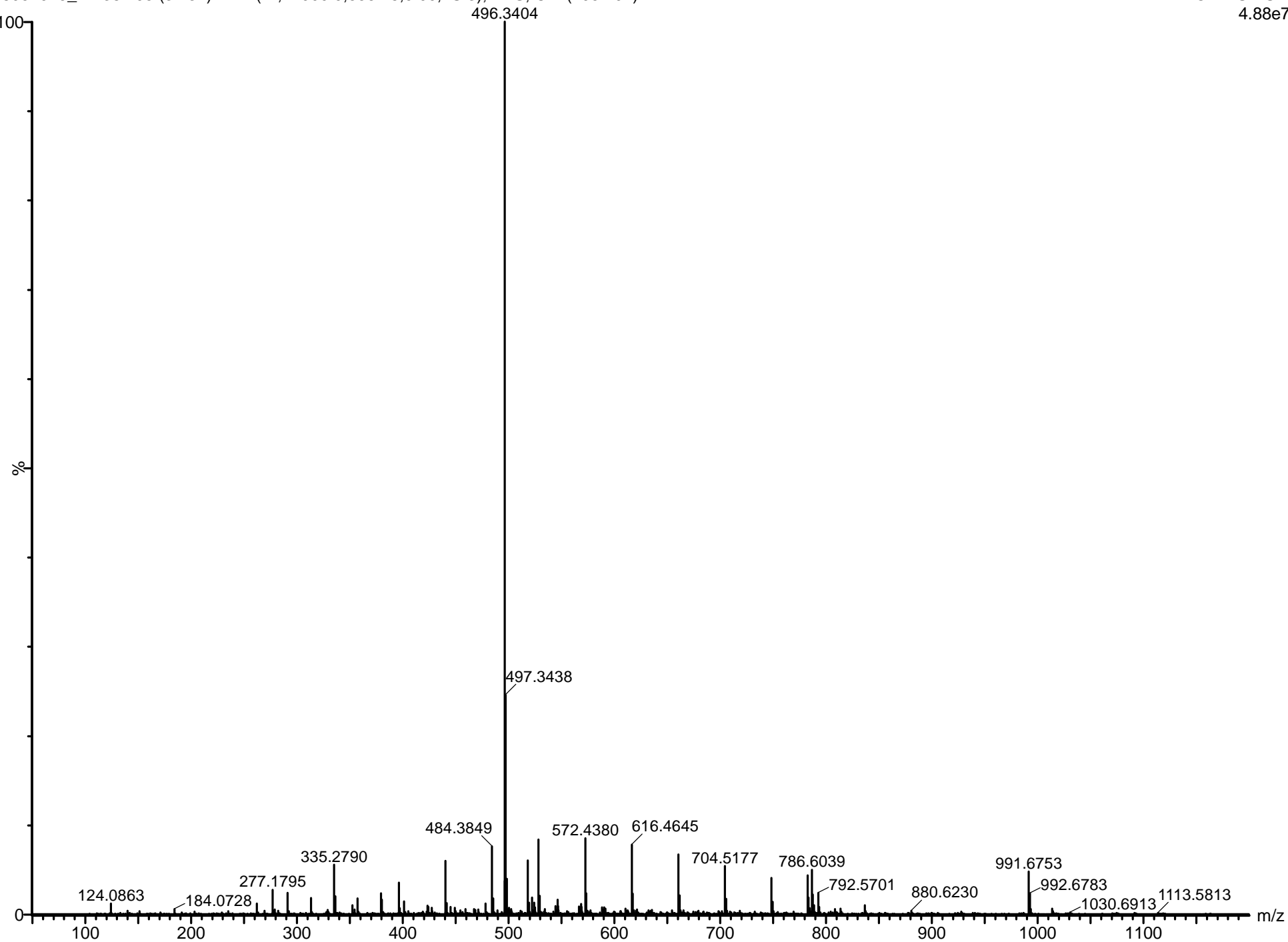

Supplement: S1 Data — Electrospray ionisation time of flight mass spectrometry (ESI-TOF MS, positive mode) spectra of the dengue cohort and ESI-TOF at different retention times. The spectra display the relative abundance (%) of detected ions across the m/z range. Prominent peaks corresponding to major ionised species are indicated. Variation in spectral profiles between retention times reflects the differences in compound composition and ionisation patterns within the sample. Data were acquired under identical instrumental conditions and are presented as representative scans. (ZIP) [file pntd.0014327.s003.zip › EM COMPLETE SAMPLES SPECTRUM/EM38 SPECTRUM RT 3.434.pdf]

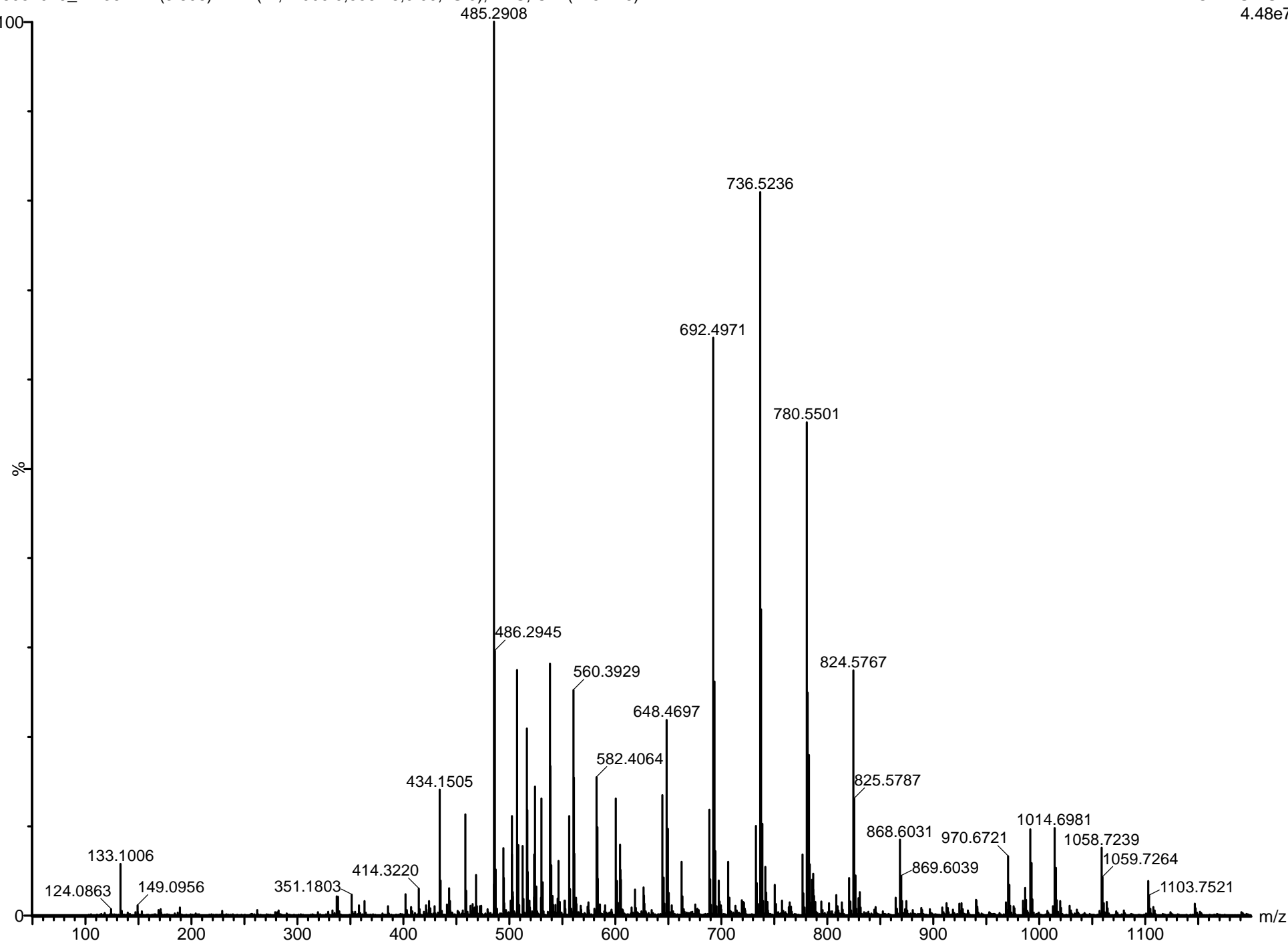

Supplement: S1 Data — Electrospray ionisation time of flight mass spectrometry (ESI-TOF MS, positive mode) spectra of the dengue cohort and ESI-TOF at different retention times. The spectra display the relative abundance (%) of detected ions across the m/z range. Prominent peaks corresponding to major ionised species are indicated. Variation in spectral profiles between retention times reflects the differences in compound composition and ionisation patterns within the sample. Data were acquired under identical instrumental conditions and are presented as representative scans. (ZIP) [file pntd.0014327.s003.zip › EM COMPLETE SAMPLES SPECTRUM/EM38 SPECTRUM RT 3.806.pdf]

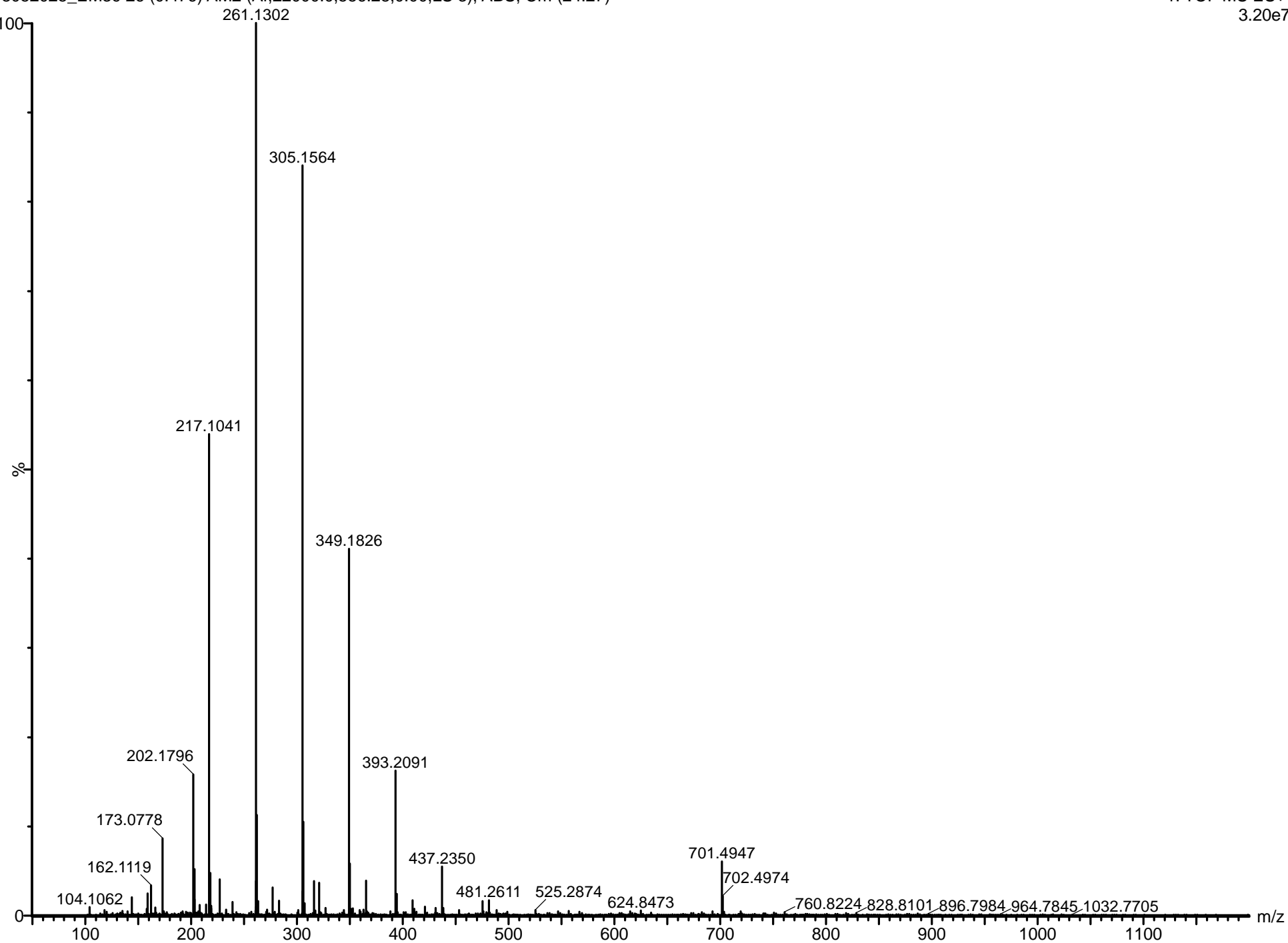

Supplement: S1 Data — Electrospray ionisation time of flight mass spectrometry (ESI-TOF MS, positive mode) spectra of the dengue cohort and ESI-TOF at different retention times. The spectra display the relative abundance (%) of detected ions across the m/z range. Prominent peaks corresponding to major ionised species are indicated. Variation in spectral profiles between retention times reflects the differences in compound composition and ionisation patterns within the sample. Data were acquired under identical instrumental conditions and are presented as representative scans. (ZIP) [file pntd.0014327.s003.zip › EM COMPLETE SAMPLES SPECTRUM/EM39 SPECTRUM RT 0.476.pdf]

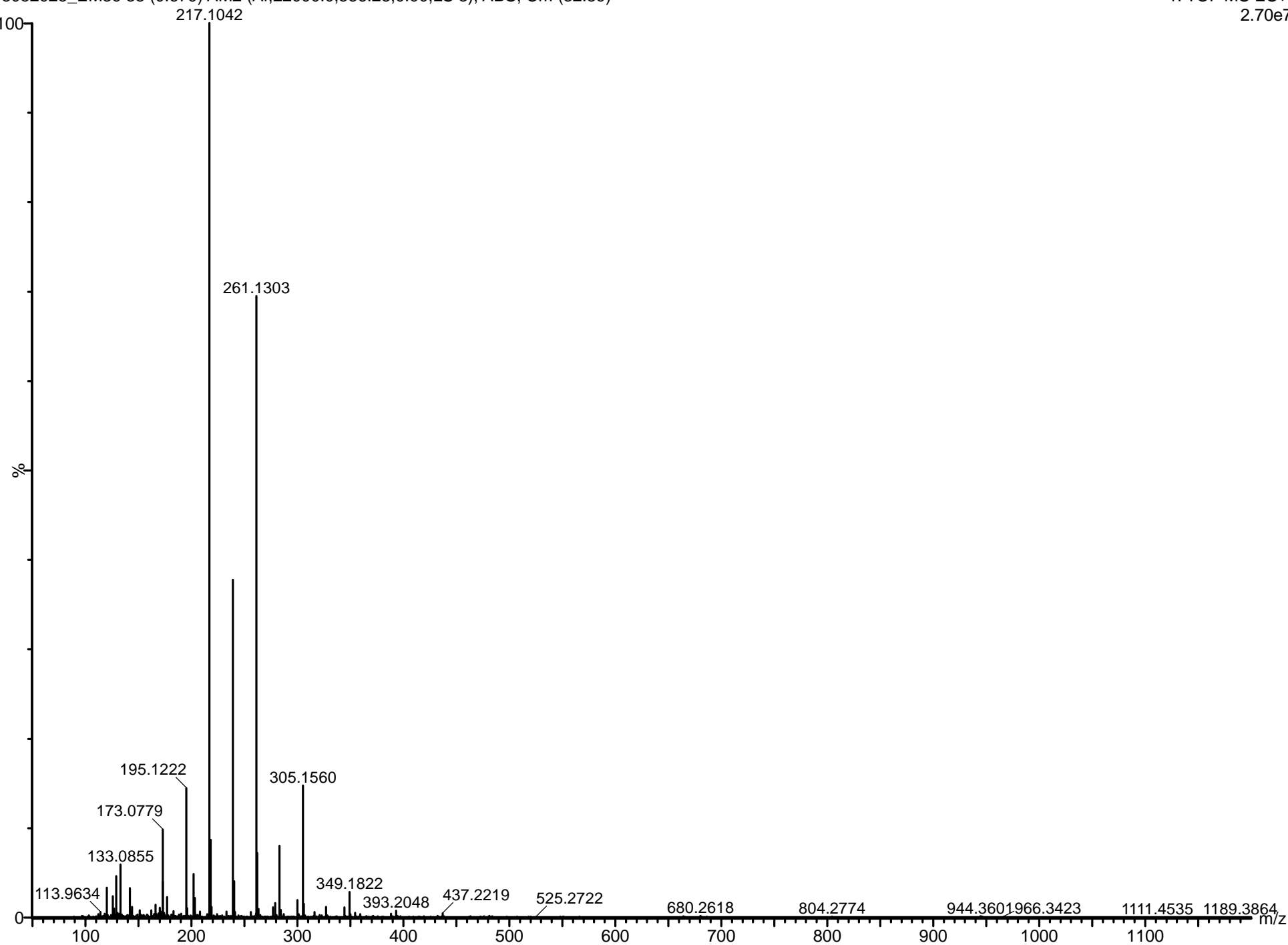

Supplement: S1 Data — Electrospray ionisation time of flight mass spectrometry (ESI-TOF MS, positive mode) spectra of the dengue cohort and ESI-TOF at different retention times. The spectra display the relative abundance (%) of detected ions across the m/z range. Prominent peaks corresponding to major ionised species are indicated. Variation in spectral profiles between retention times reflects the differences in compound composition and ionisation patterns within the sample. Data were acquired under identical instrumental conditions and are presented as representative scans. (ZIP) [file pntd.0014327.s003.zip › EM COMPLETE SAMPLES SPECTRUM/EM39 SPECTRUM RT 0.679.pdf]

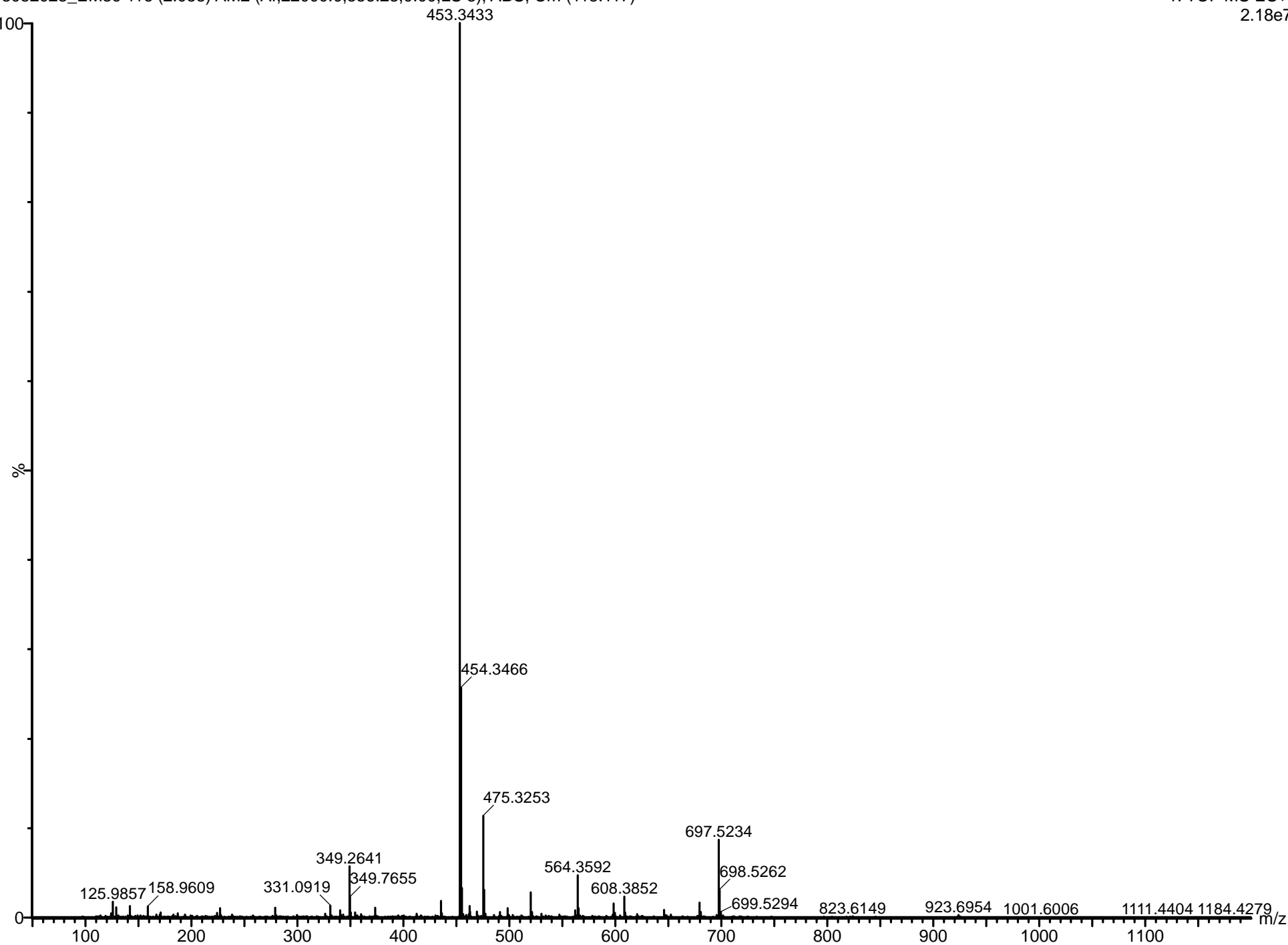

Supplement: S1 Data — Electrospray ionisation time of flight mass spectrometry (ESI-TOF MS, positive mode) spectra of the dengue cohort and ESI-TOF at different retention times. The spectra display the relative abundance (%) of detected ions across the m/z range. Prominent peaks corresponding to major ionised species are indicated. Variation in spectral profiles between retention times reflects the differences in compound composition and ionisation patterns within the sample. Data were acquired under identical instrumental conditions and are presented as representative scans. (ZIP) [file pntd.0014327.s003.zip › EM COMPLETE SAMPLES SPECTRUM/EM39 SPECTRUM RT 2.058.pdf]

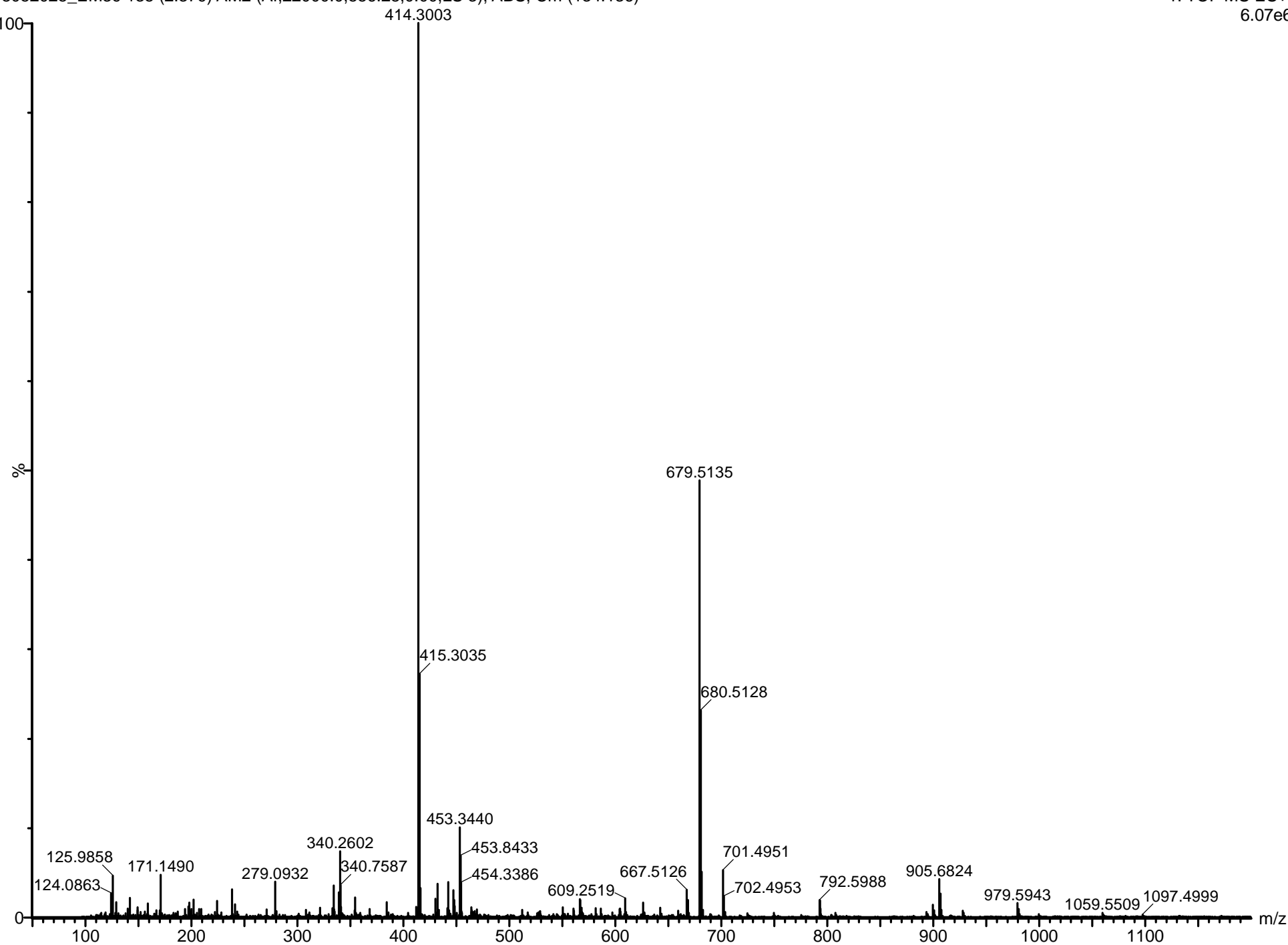

Supplement: S1 Data — Electrospray ionisation time of flight mass spectrometry (ESI-TOF MS, positive mode) spectra of the dengue cohort and ESI-TOF at different retention times. The spectra display the relative abundance (%) of detected ions across the m/z range. Prominent peaks corresponding to major ionised species are indicated. Variation in spectral profiles between retention times reflects the differences in compound composition and ionisation patterns within the sample. Data were acquired under identical instrumental conditions and are presented as representative scans. (ZIP) [file pntd.0014327.s003.zip › EM COMPLETE SAMPLES SPECTRUM/EM39 SPECTRUM RT 2.379.pdf]

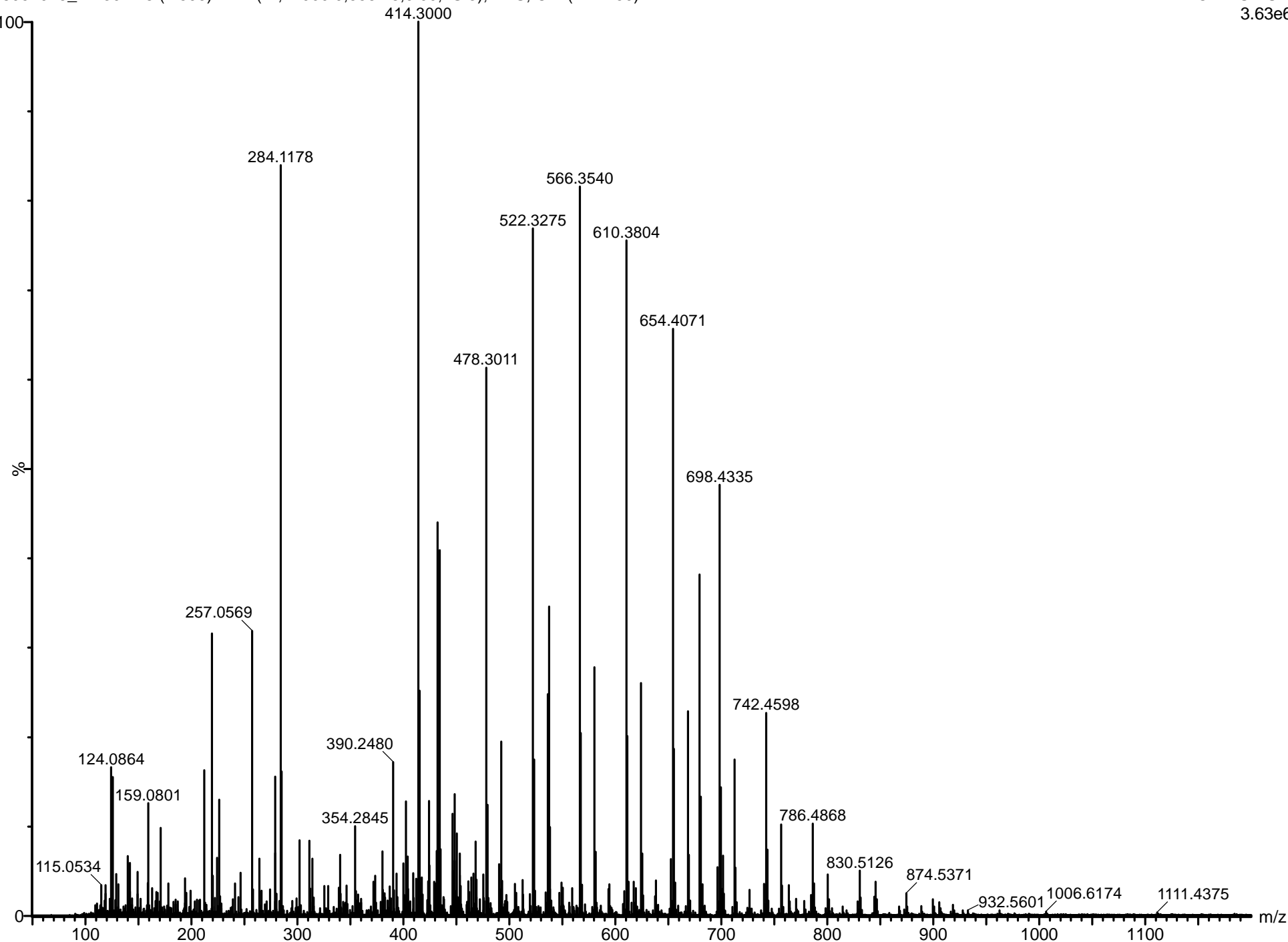

Supplement: S1 Data — Electrospray ionisation time of flight mass spectrometry (ESI-TOF MS, positive mode) spectra of the dengue cohort and ESI-TOF at different retention times. The spectra display the relative abundance (%) of detected ions across the m/z range. Prominent peaks corresponding to major ionised species are indicated. Variation in spectral profiles between retention times reflects the differences in compound composition and ionisation patterns within the sample. Data were acquired under identical instrumental conditions and are presented as representative scans. (ZIP) [file pntd.0014327.s003.zip › EM COMPLETE SAMPLES SPECTRUM/EM39 SPECTRUM RT 2.599.pdf]

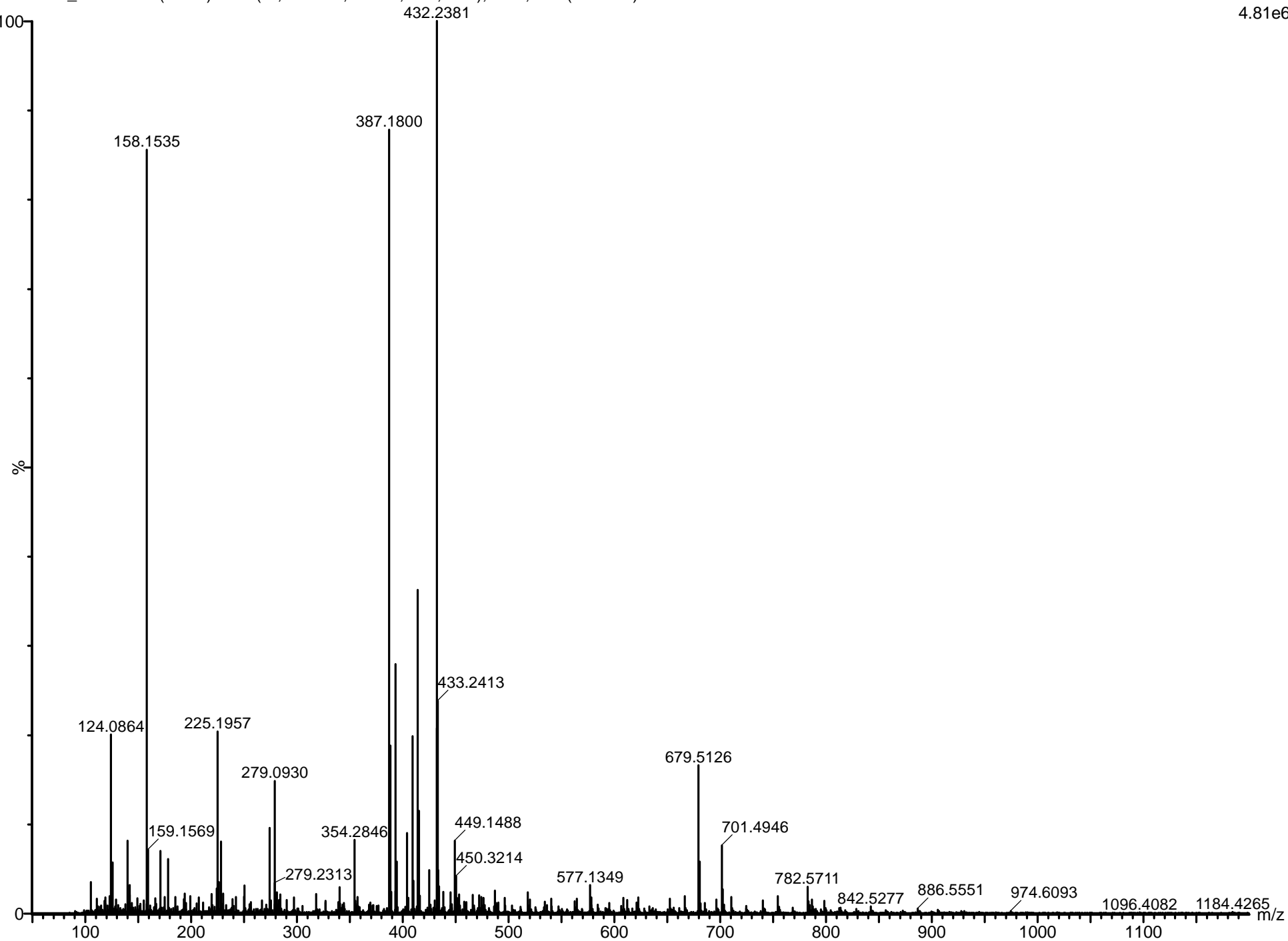

Supplement: S1 Data — Electrospray ionisation time of flight mass spectrometry (ESI-TOF MS, positive mode) spectra of the dengue cohort and ESI-TOF at different retention times. The spectra display the relative abundance (%) of detected ions across the m/z range. Prominent peaks corresponding to major ionised species are indicated. Variation in spectral profiles between retention times reflects the differences in compound composition and ionisation patterns within the sample. Data were acquired under identical instrumental conditions and are presented as representative scans. (ZIP) [file pntd.0014327.s003.zip › EM COMPLETE SAMPLES SPECTRUM/EM39 SPECTRUM RT 2.873.pdf]

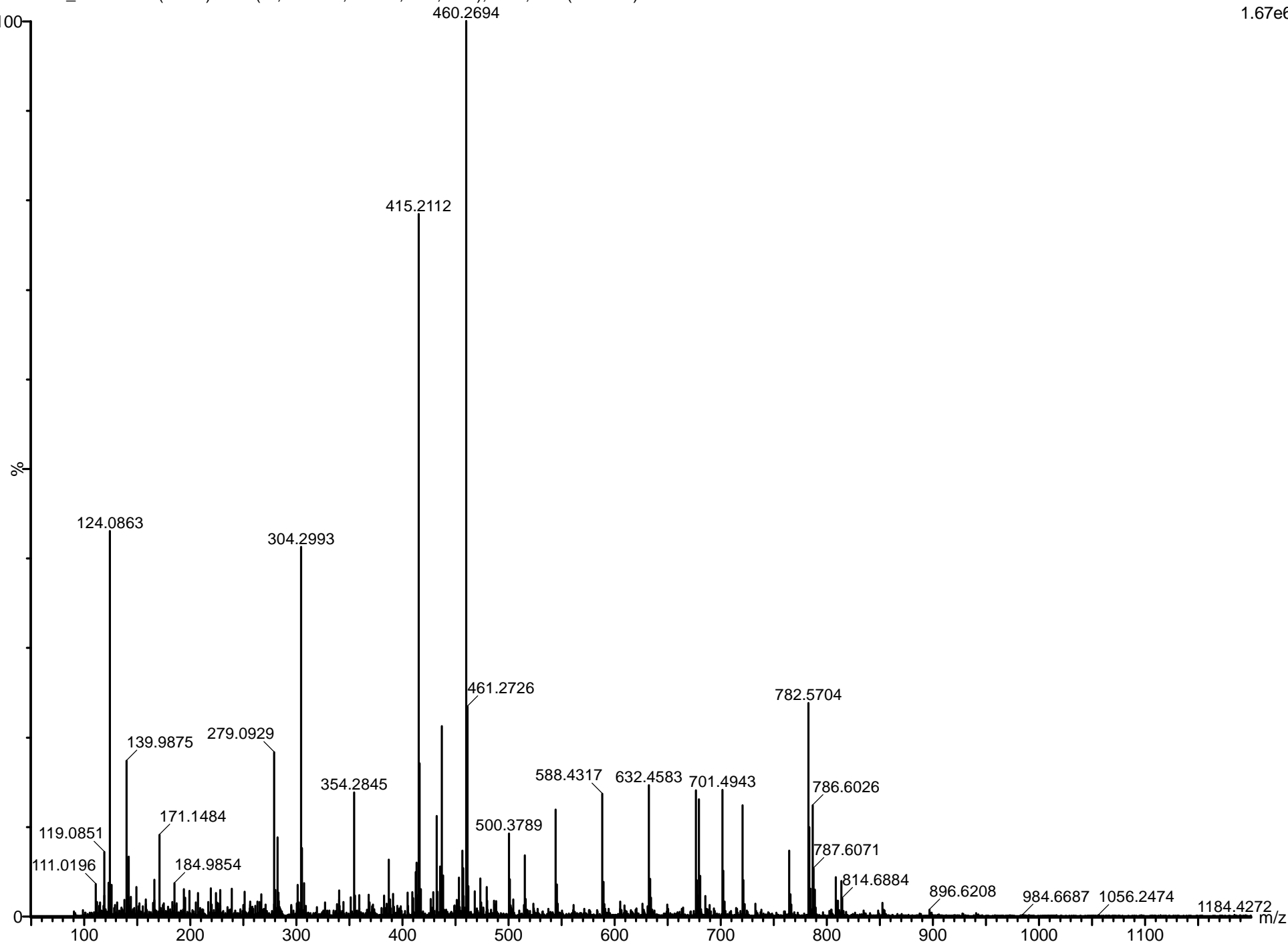

Supplement: S1 Data — Electrospray ionisation time of flight mass spectrometry (ESI-TOF MS, positive mode) spectra of the dengue cohort and ESI-TOF at different retention times. The spectra display the relative abundance (%) of detected ions across the m/z range. Prominent peaks corresponding to major ionised species are indicated. Variation in spectral profiles between retention times reflects the differences in compound composition and ionisation patterns within the sample. Data were acquired under identical instrumental conditions and are presented as representative scans. (ZIP) [file pntd.0014327.s003.zip › EM COMPLETE SAMPLES SPECTRUM/EM39 SPECTRUM RT 3.092.pdf]

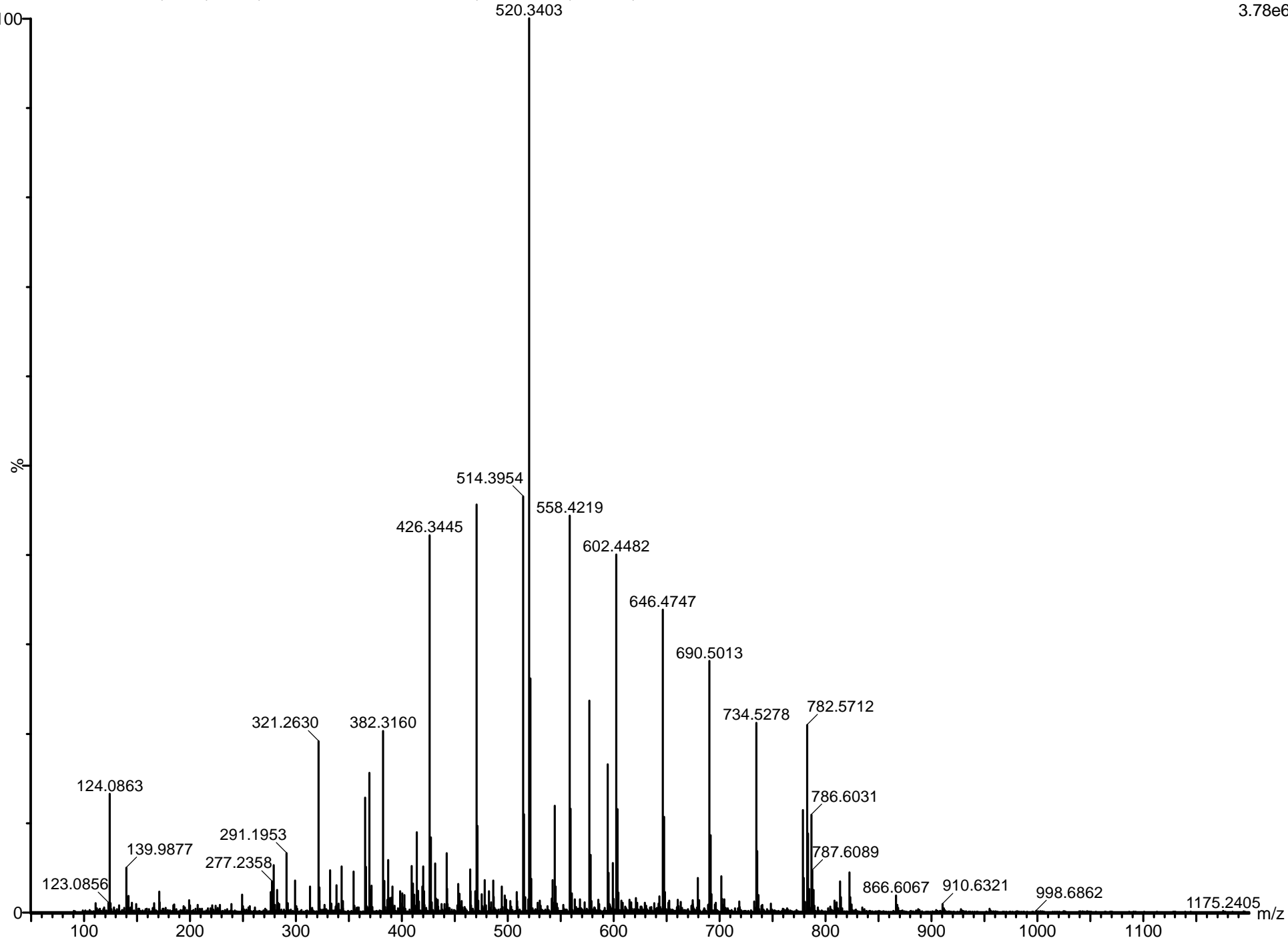

Supplement: S1 Data — Electrospray ionisation time of flight mass spectrometry (ESI-TOF MS, positive mode) spectra of the dengue cohort and ESI-TOF at different retention times. The spectra display the relative abundance (%) of detected ions across the m/z range. Prominent peaks corresponding to major ionised species are indicated. Variation in spectral profiles between retention times reflects the differences in compound composition and ionisation patterns within the sample. Data were acquired under identical instrumental conditions and are presented as representative scans. (ZIP) [file pntd.0014327.s003.zip › EM COMPLETE SAMPLES SPECTRUM/EM39 SPECTRUM RT 3.279.pdf]
