# Supplementary figures and images for: Metabolomic atlas of dengue virus infection reveals distinct circulating bioactive lipid signatures (part 1 of 2)
Source: PLoS Negl Trop Dis. 2026 May 12;20(5):e0014327. doi: 10.1371/journal.pntd.0014327 (PMC13189415; doi:10.1371/journal.pntd.0014327)

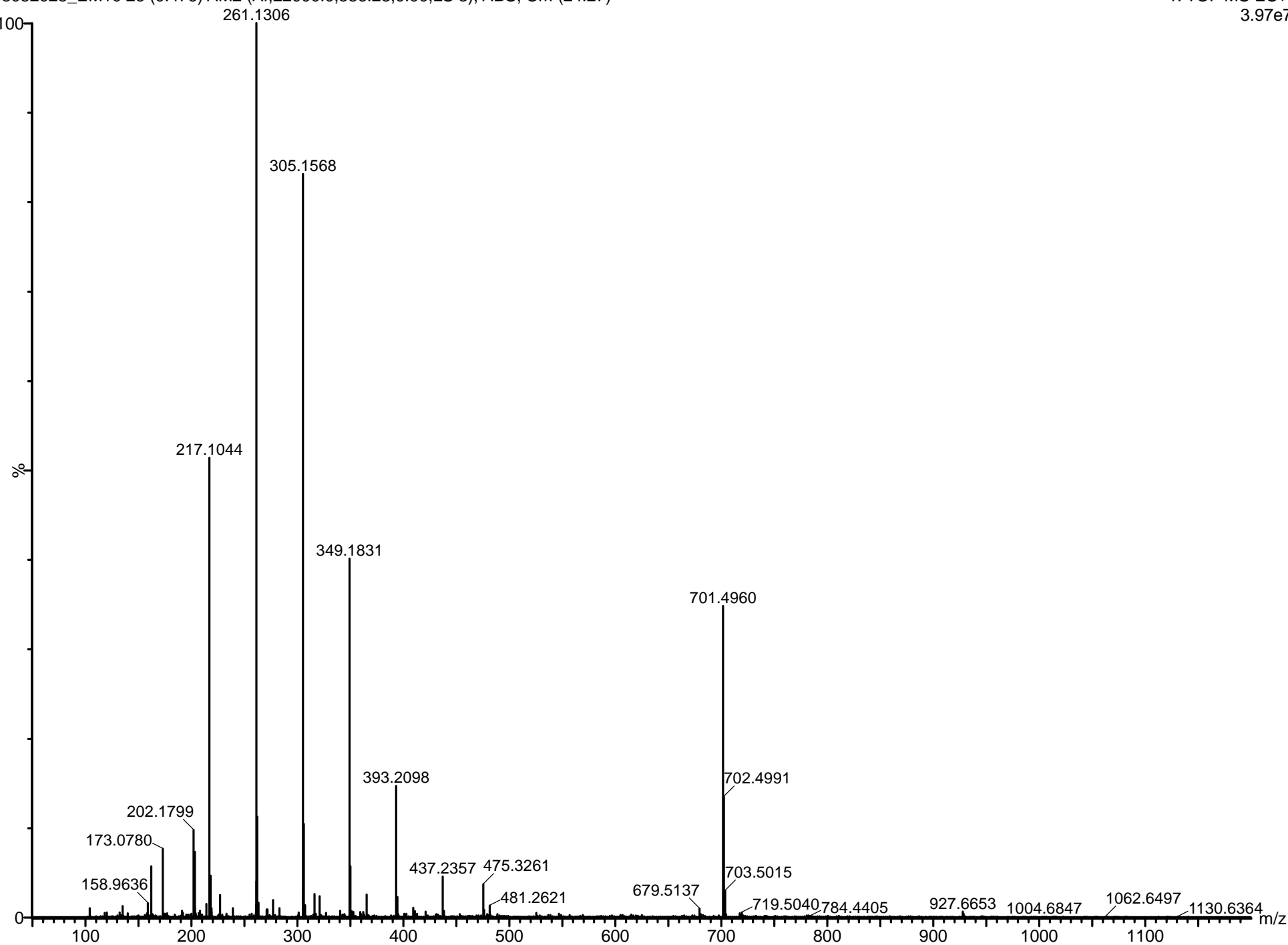

Supplement: S1 Data — Electrospray ionisation time of flight mass spectrometry (ESI-TOF MS, positive mode) spectra of the dengue cohort and ESI-TOF at different retention times. The spectra display the relative abundance (%) of detected ions across the m/z range. Prominent peaks corresponding to major ionised species are indicated. Variation in spectral profiles between retention times reflects the differences in compound composition and ionisation patterns within the sample. Data were acquired under identical instrumental conditions and are presented as representative scans. (ZIP) [file pntd.0014327.s003.zip › EM COMPLETE SAMPLES SPECTRUM/EM10 SPECTRUM RT 0.476.pdf]

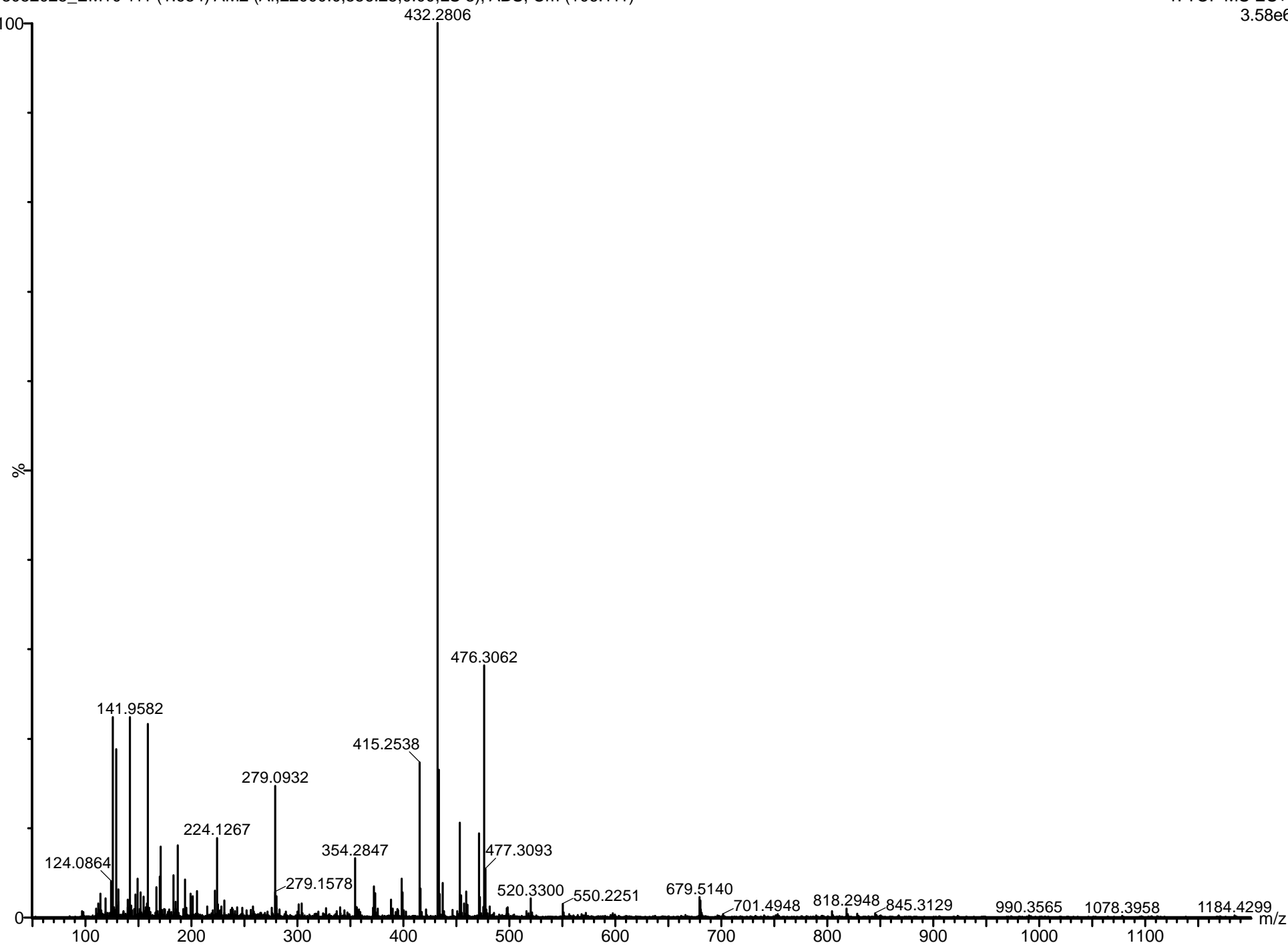

Supplement: S1 Data — Electrospray ionisation time of flight mass spectrometry (ESI-TOF MS, positive mode) spectra of the dengue cohort and ESI-TOF at different retention times. The spectra display the relative abundance (%) of detected ions across the m/z range. Prominent peaks corresponding to major ionised species are indicated. Variation in spectral profiles between retention times reflects the differences in compound composition and ionisation patterns within the sample. Data were acquired under identical instrumental conditions and are presented as representative scans. (ZIP) [file pntd.0014327.s003.zip › EM COMPLETE SAMPLES SPECTRUM/EM10 SPECTRUM RT 1.954.pdf]

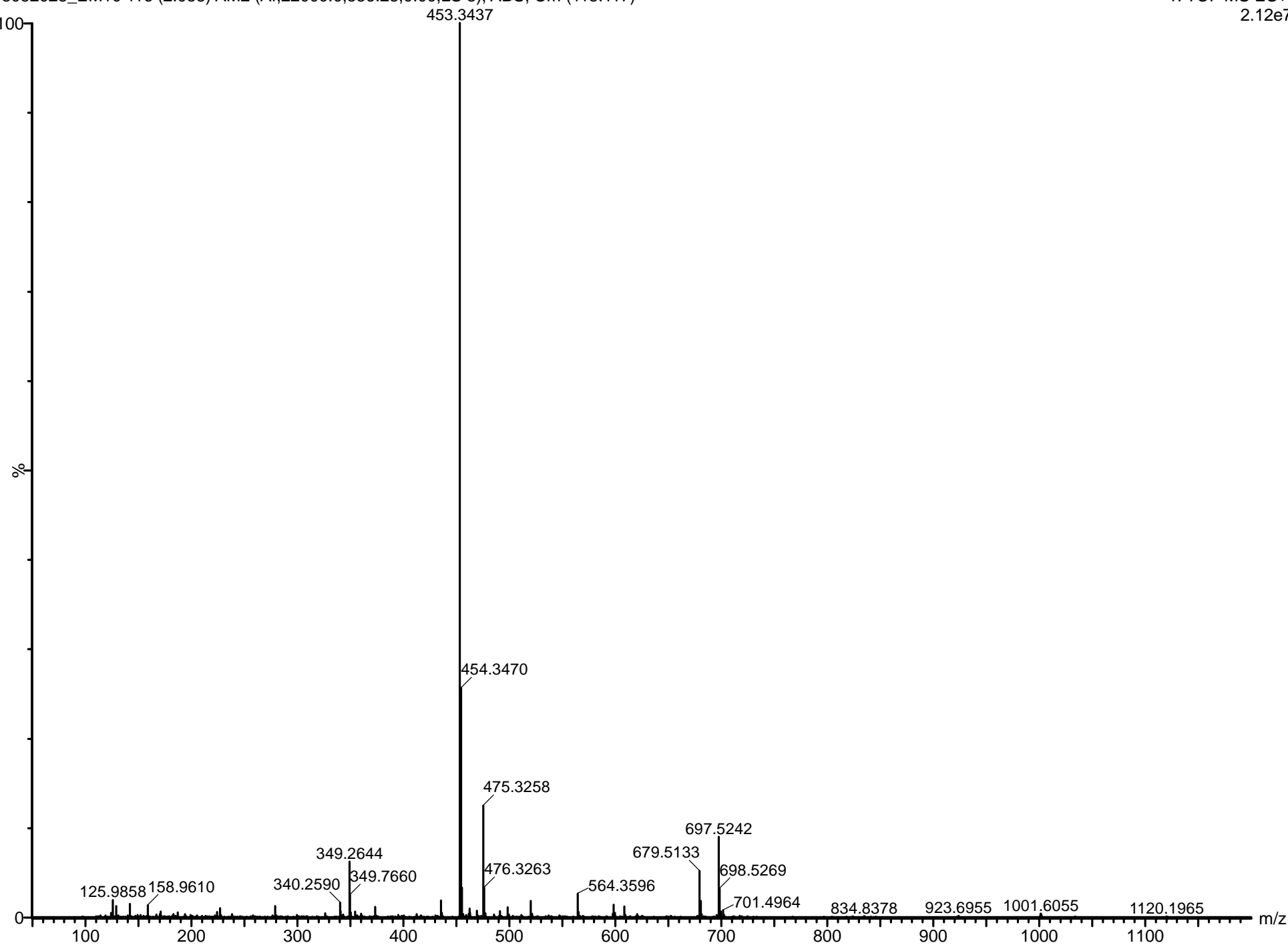

Supplement: S1 Data — Electrospray ionisation time of flight mass spectrometry (ESI-TOF MS, positive mode) spectra of the dengue cohort and ESI-TOF at different retention times. The spectra display the relative abundance (%) of detected ions across the m/z range. Prominent peaks corresponding to major ionised species are indicated. Variation in spectral profiles between retention times reflects the differences in compound composition and ionisation patterns within the sample. Data were acquired under identical instrumental conditions and are presented as representative scans. (ZIP) [file pntd.0014327.s003.zip › EM COMPLETE SAMPLES SPECTRUM/EM10 SPECTRUM RT 2.058.pdf]

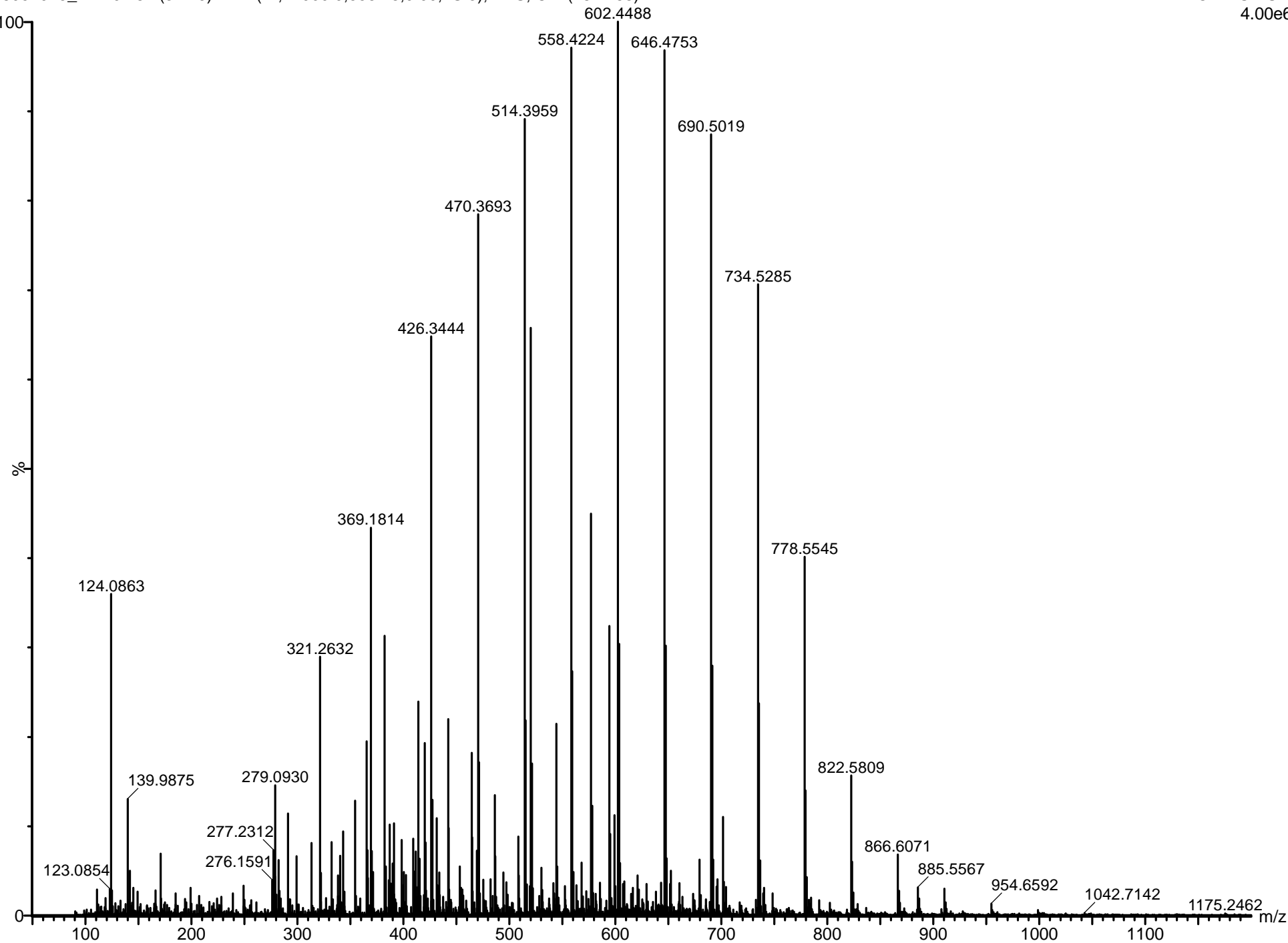

Supplement: S1 Data — Electrospray ionisation time of flight mass spectrometry (ESI-TOF MS, positive mode) spectra of the dengue cohort and ESI-TOF at different retention times. The spectra display the relative abundance (%) of detected ions across the m/z range. Prominent peaks corresponding to major ionised species are indicated. Variation in spectral profiles between retention times reflects the differences in compound composition and ionisation patterns within the sample. Data were acquired under identical instrumental conditions and are presented as representative scans. (ZIP) [file pntd.0014327.s003.zip › EM COMPLETE SAMPLES SPECTRUM/EM10 SPECTRUM RT 2.279.pdf]

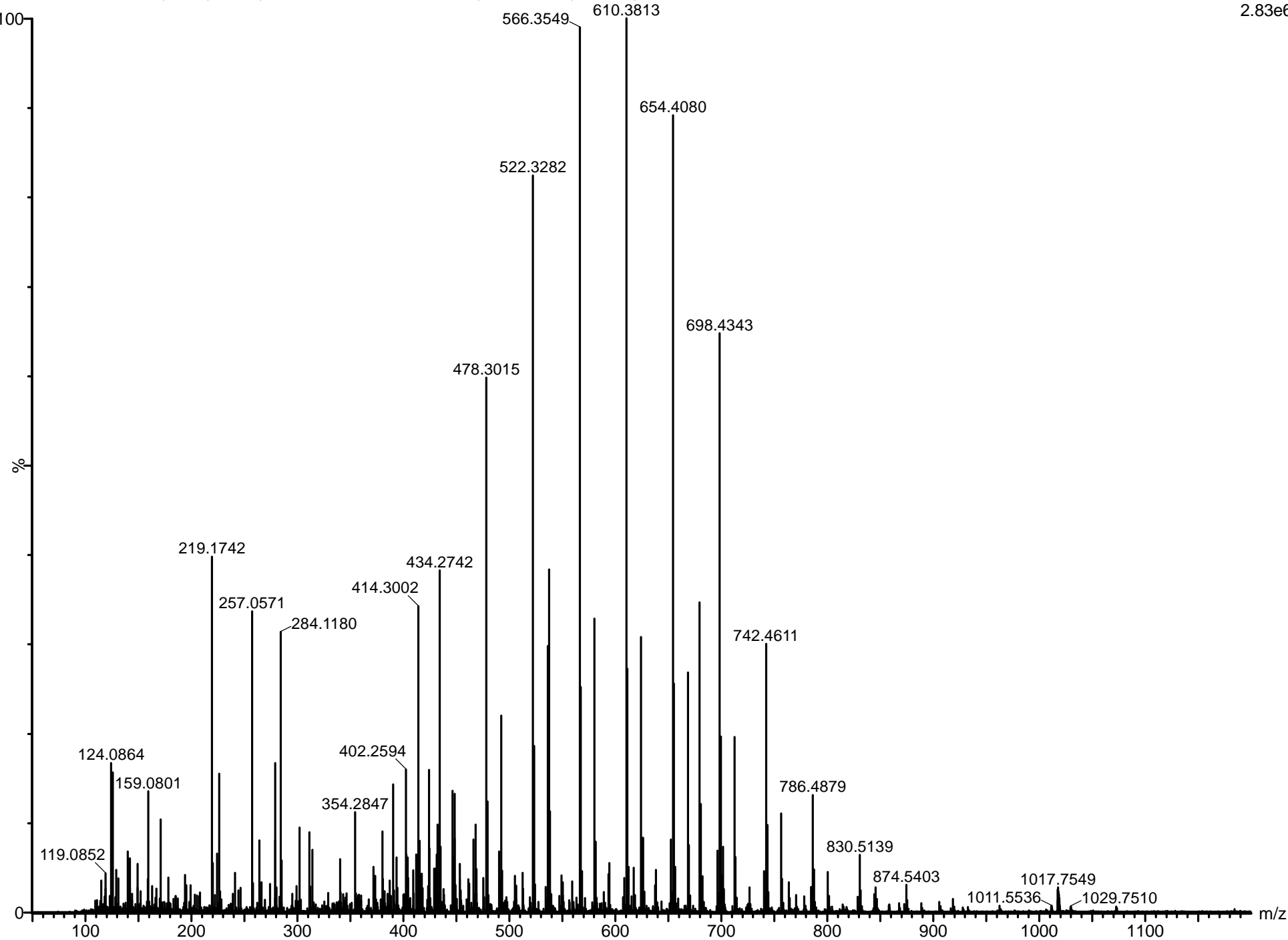

Supplement: S1 Data — Electrospray ionisation time of flight mass spectrometry (ESI-TOF MS, positive mode) spectra of the dengue cohort and ESI-TOF at different retention times. The spectra display the relative abundance (%) of detected ions across the m/z range. Prominent peaks corresponding to major ionised species are indicated. Variation in spectral profiles between retention times reflects the differences in compound composition and ionisation patterns within the sample. Data were acquired under identical instrumental conditions and are presented as representative scans. (ZIP) [file pntd.0014327.s003.zip › EM COMPLETE SAMPLES SPECTRUM/EM10 SPECTRUM RT 2.565.pdf]

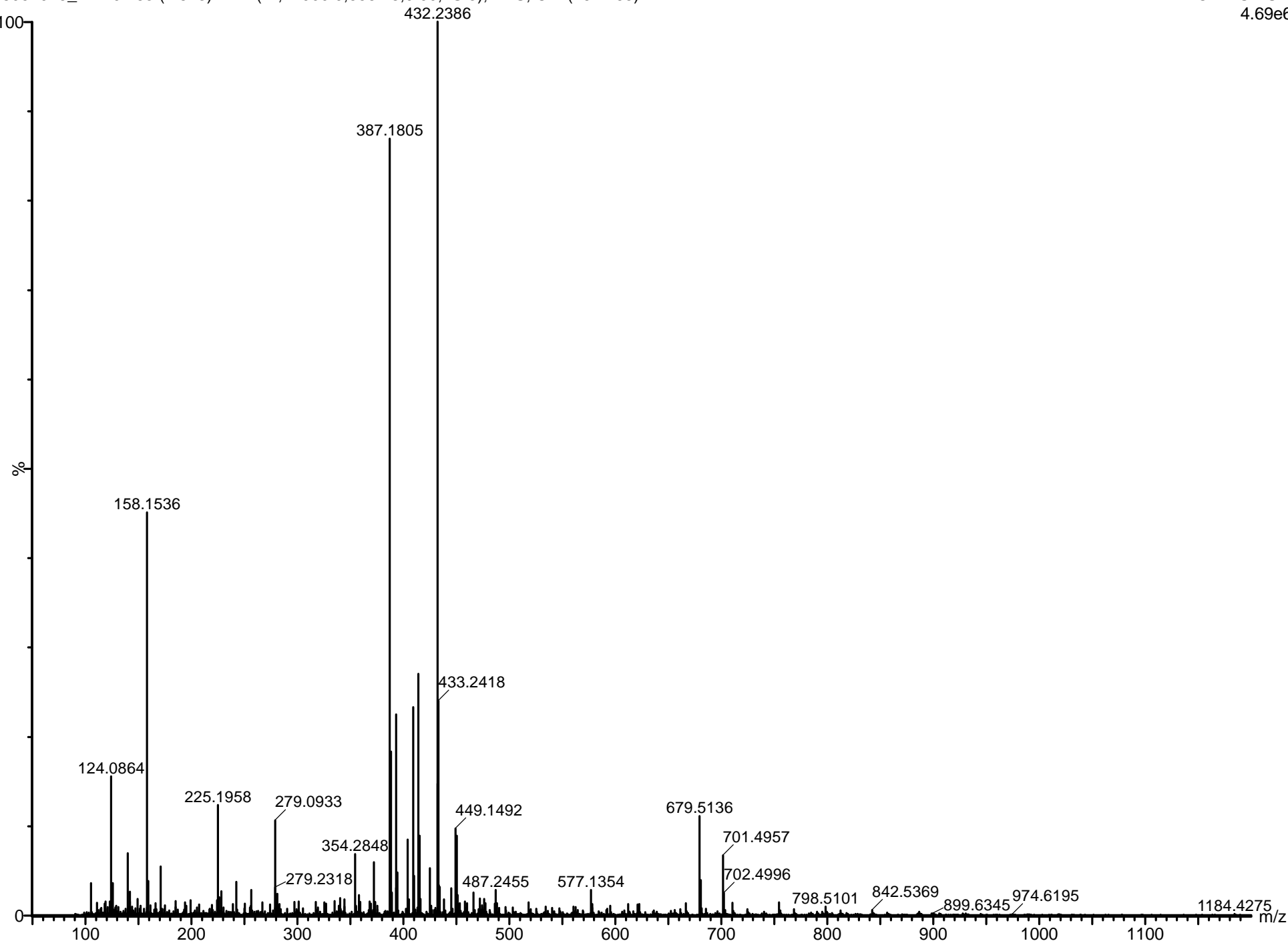

Supplement: S1 Data — Electrospray ionisation time of flight mass spectrometry (ESI-TOF MS, positive mode) spectra of the dengue cohort and ESI-TOF at different retention times. The spectra display the relative abundance (%) of detected ions across the m/z range. Prominent peaks corresponding to major ionised species are indicated. Variation in spectral profiles between retention times reflects the differences in compound composition and ionisation patterns within the sample. Data were acquired under identical instrumental conditions and are presented as representative scans. (ZIP) [file pntd.0014327.s003.zip › EM COMPLETE SAMPLES SPECTRUM/EM10 SPECTRUM RT 2.873.pdf]

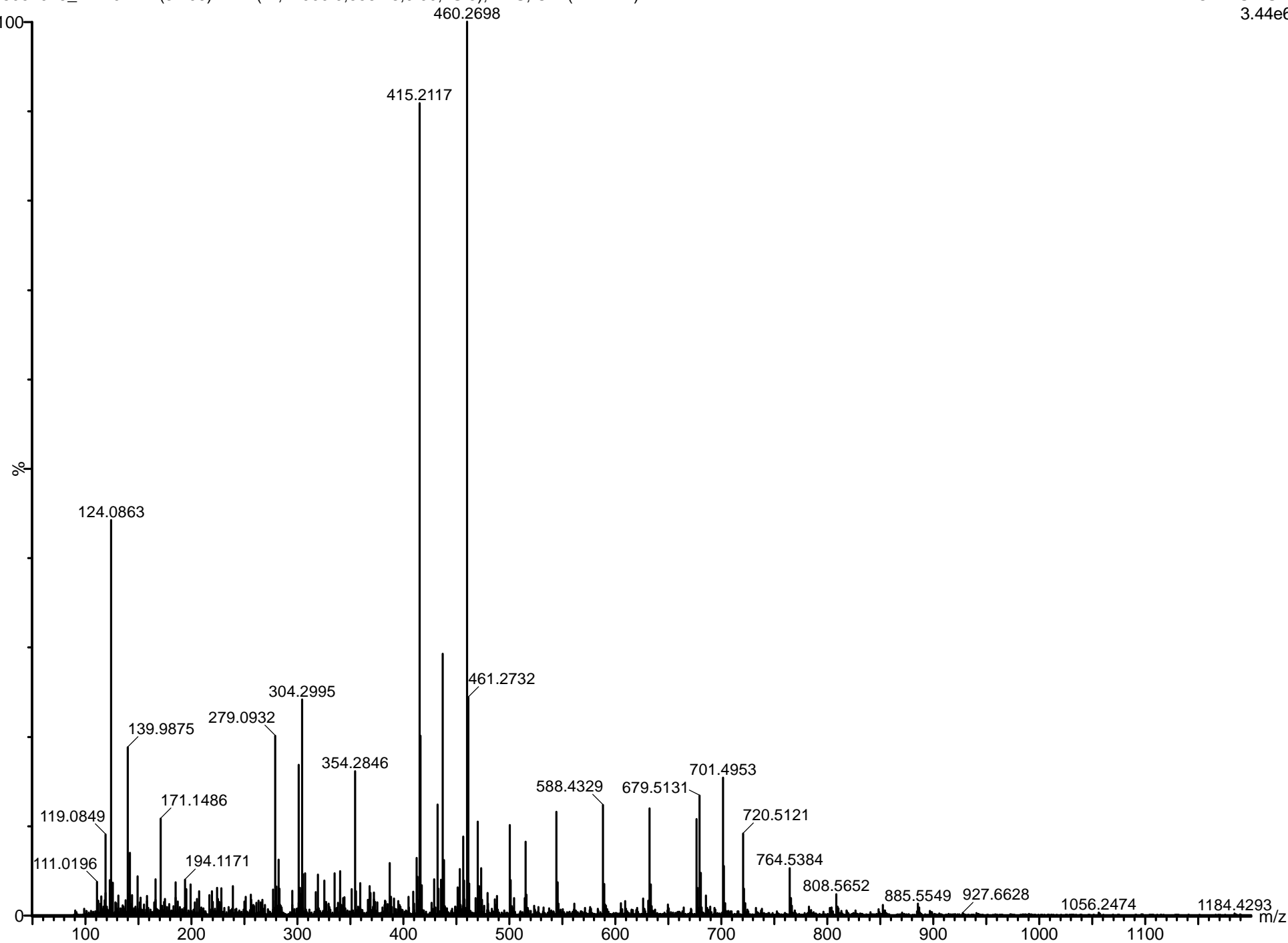

Supplement: S1 Data — Electrospray ionisation time of flight mass spectrometry (ESI-TOF MS, positive mode) spectra of the dengue cohort and ESI-TOF at different retention times. The spectra display the relative abundance (%) of detected ions across the m/z range. Prominent peaks corresponding to major ionised species are indicated. Variation in spectral profiles between retention times reflects the differences in compound composition and ionisation patterns within the sample. Data were acquired under identical instrumental conditions and are presented as representative scans. (ZIP) [file pntd.0014327.s003.zip › EM COMPLETE SAMPLES SPECTRUM/EM10 SPECTRUM RT 3.109.pdf]

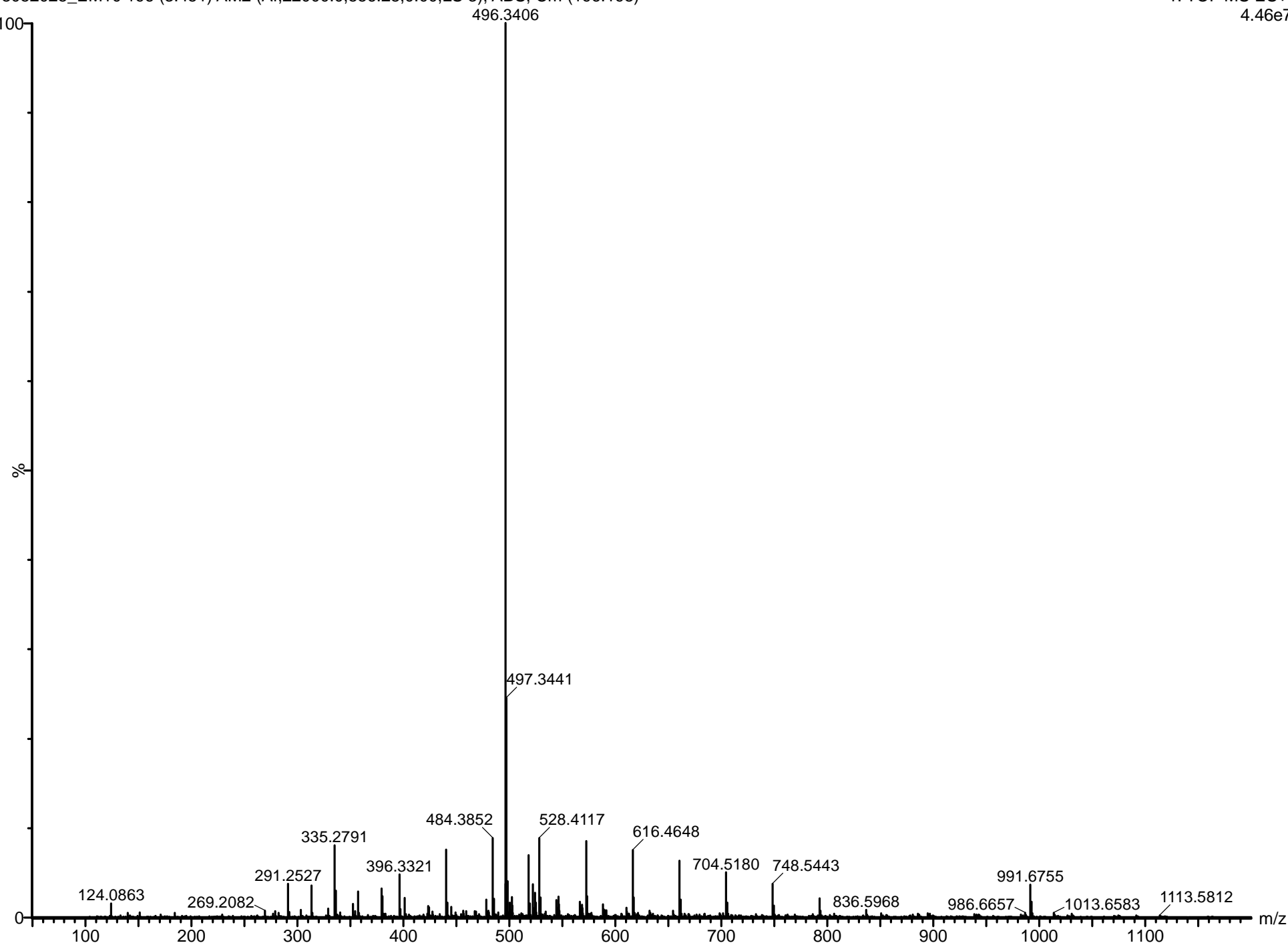

Supplement: S1 Data — Electrospray ionisation time of flight mass spectrometry (ESI-TOF MS, positive mode) spectra of the dengue cohort and ESI-TOF at different retention times. The spectra display the relative abundance (%) of detected ions across the m/z range. Prominent peaks corresponding to major ionised species are indicated. Variation in spectral profiles between retention times reflects the differences in compound composition and ionisation patterns within the sample. Data were acquired under identical instrumental conditions and are presented as representative scans. (ZIP) [file pntd.0014327.s003.zip › EM COMPLETE SAMPLES SPECTRUM/EM10 SPECTRUM RT 3.451.pdf]

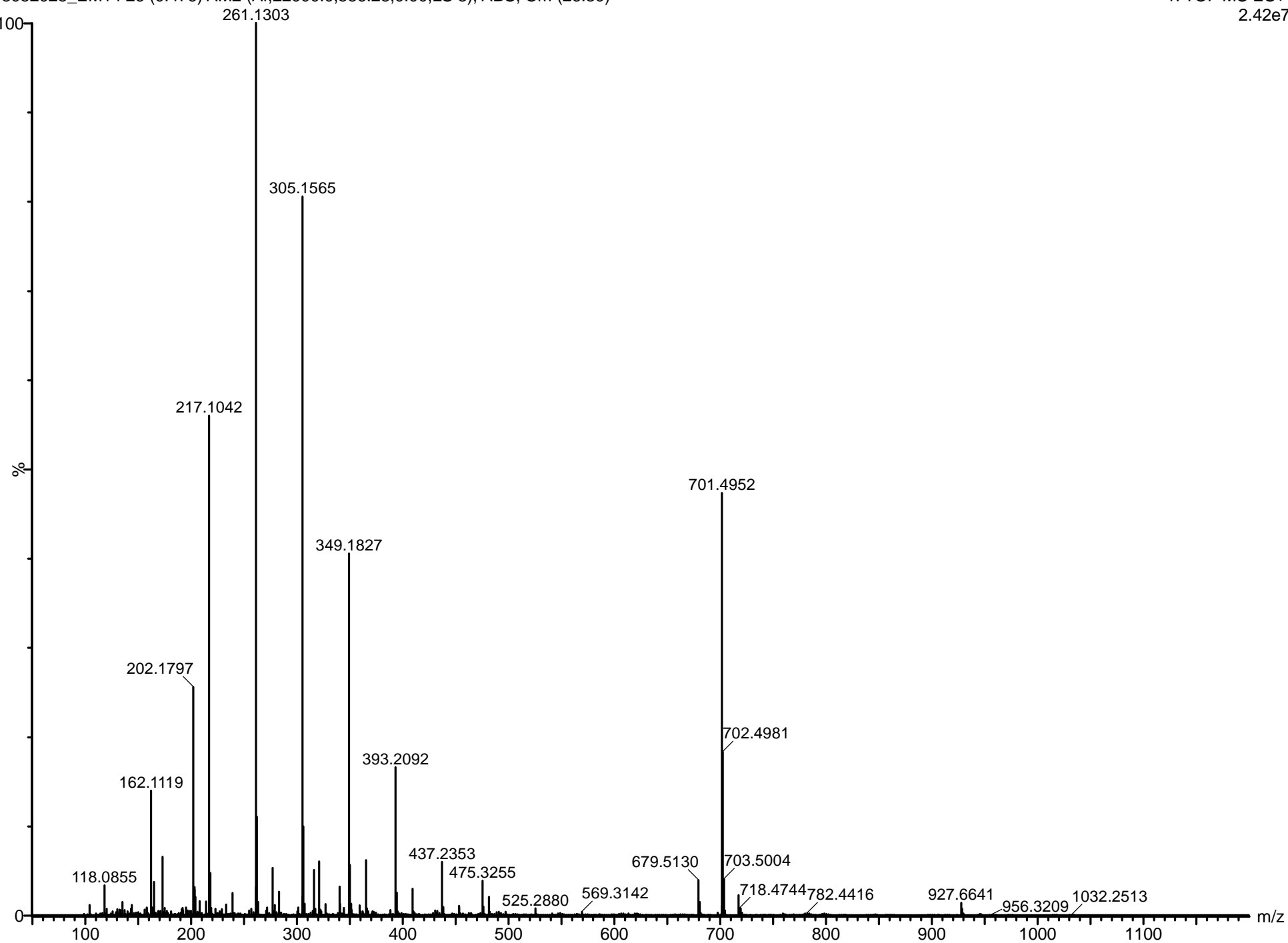

Supplement: S1 Data — Electrospray ionisation time of flight mass spectrometry (ESI-TOF MS, positive mode) spectra of the dengue cohort and ESI-TOF at different retention times. The spectra display the relative abundance (%) of detected ions across the m/z range. Prominent peaks corresponding to major ionised species are indicated. Variation in spectral profiles between retention times reflects the differences in compound composition and ionisation patterns within the sample. Data were acquired under identical instrumental conditions and are presented as representative scans. (ZIP) [file pntd.0014327.s003.zip › EM COMPLETE SAMPLES SPECTRUM/EM14 SPECTRUM RT 0.476.pdf]

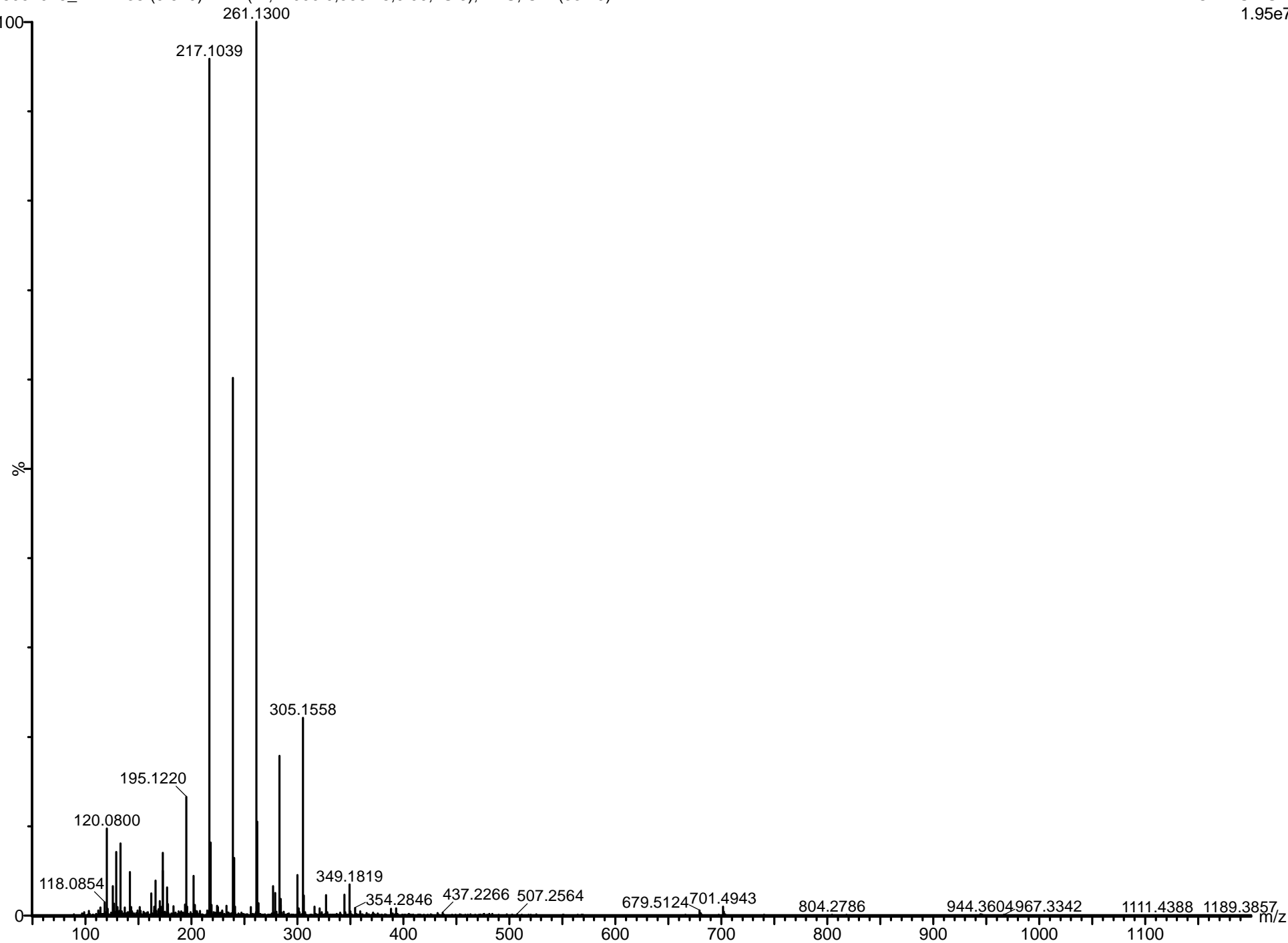

Supplement: S1 Data — Electrospray ionisation time of flight mass spectrometry (ESI-TOF MS, positive mode) spectra of the dengue cohort and ESI-TOF at different retention times. The spectra display the relative abundance (%) of detected ions across the m/z range. Prominent peaks corresponding to major ionised species are indicated. Variation in spectral profiles between retention times reflects the differences in compound composition and ionisation patterns within the sample. Data were acquired under identical instrumental conditions and are presented as representative scans. (ZIP) [file pntd.0014327.s003.zip › EM COMPLETE SAMPLES SPECTRUM/EM14 SPECTRUM RT 0.679.pdf]

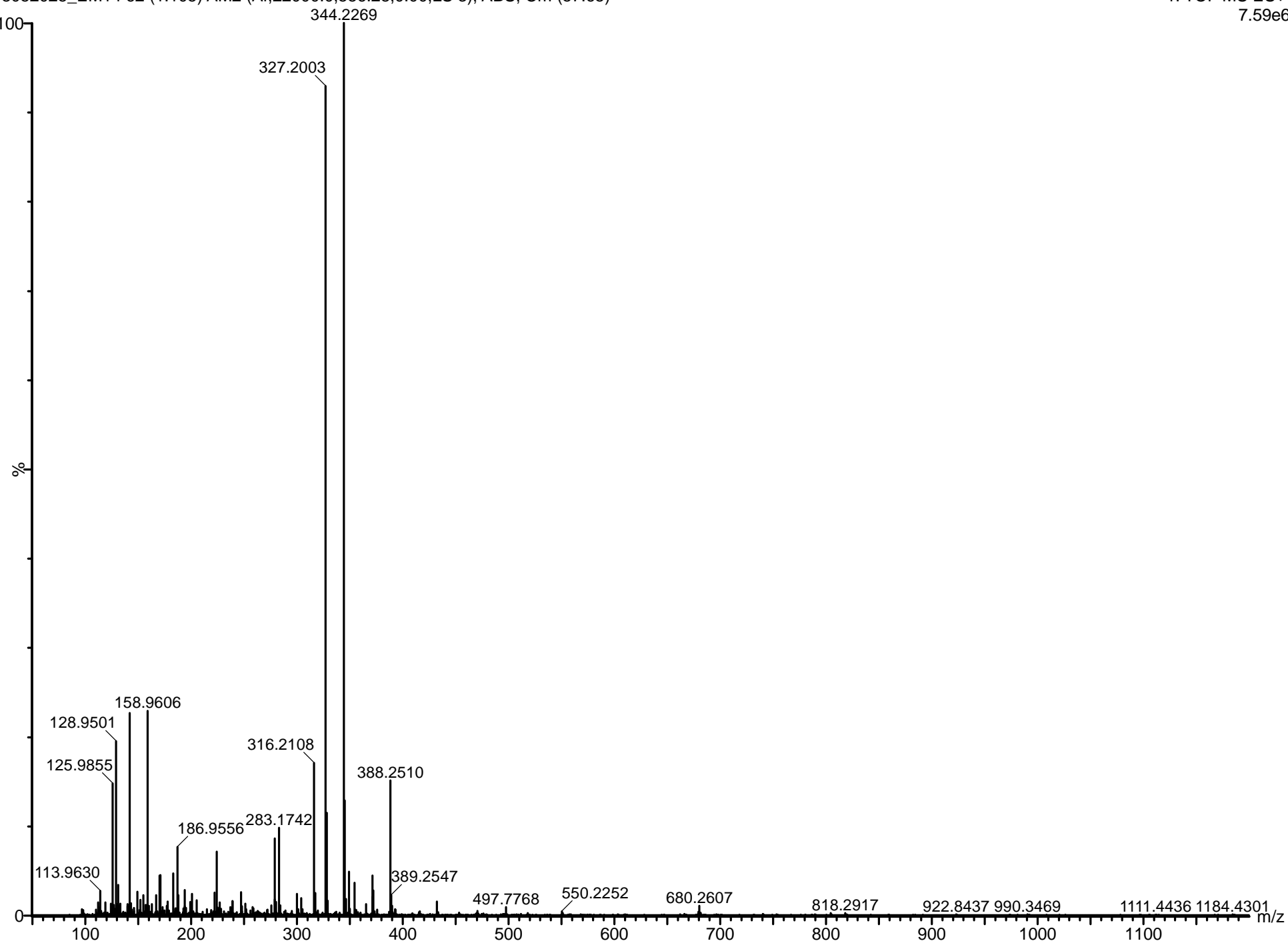

Supplement: S1 Data — Electrospray ionisation time of flight mass spectrometry (ESI-TOF MS, positive mode) spectra of the dengue cohort and ESI-TOF at different retention times. The spectra display the relative abundance (%) of detected ions across the m/z range. Prominent peaks corresponding to major ionised species are indicated. Variation in spectral profiles between retention times reflects the differences in compound composition and ionisation patterns within the sample. Data were acquired under identical instrumental conditions and are presented as representative scans. (ZIP) [file pntd.0014327.s003.zip › EM COMPLETE SAMPLES SPECTRUM/EM14 SPECTRUM RT 1.105.pdf]

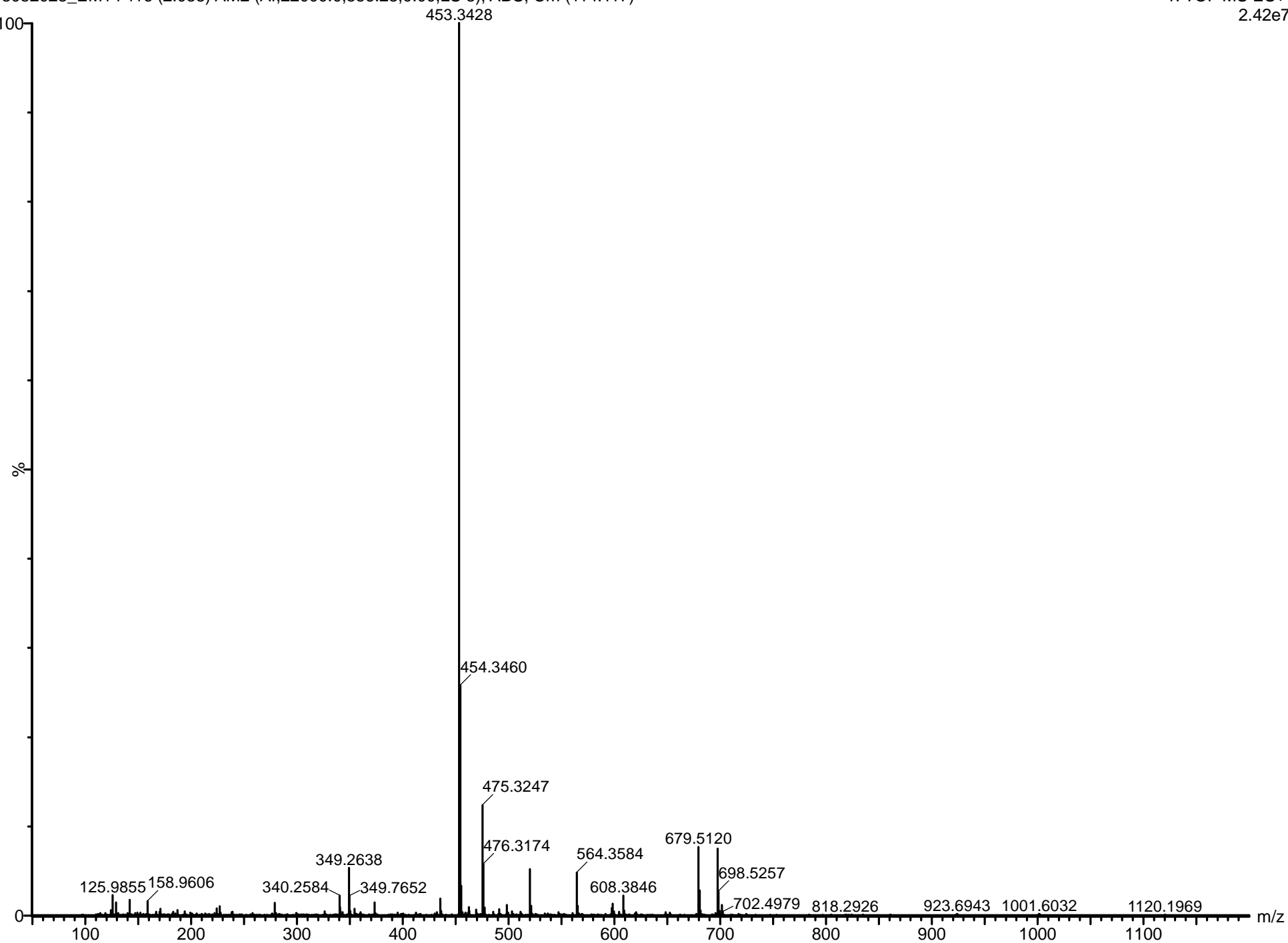

Supplement: S1 Data — Electrospray ionisation time of flight mass spectrometry (ESI-TOF MS, positive mode) spectra of the dengue cohort and ESI-TOF at different retention times. The spectra display the relative abundance (%) of detected ions across the m/z range. Prominent peaks corresponding to major ionised species are indicated. Variation in spectral profiles between retention times reflects the differences in compound composition and ionisation patterns within the sample. Data were acquired under identical instrumental conditions and are presented as representative scans. (ZIP) [file pntd.0014327.s003.zip › EM COMPLETE SAMPLES SPECTRUM/EM14 SPECTRUM RT 2.058.pdf]

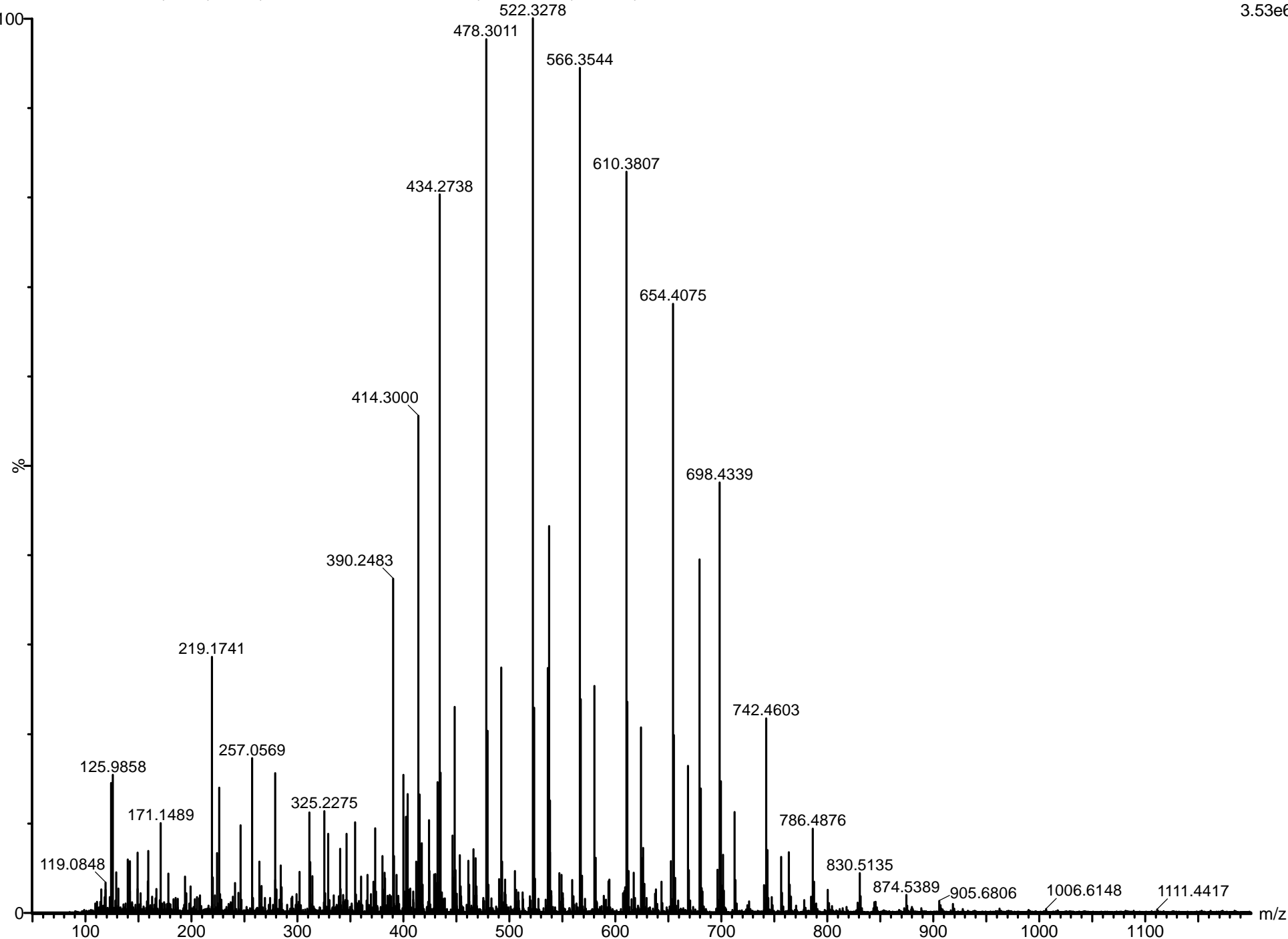

Supplement: S1 Data — Electrospray ionisation time of flight mass spectrometry (ESI-TOF MS, positive mode) spectra of the dengue cohort and ESI-TOF at different retention times. The spectra display the relative abundance (%) of detected ions across the m/z range. Prominent peaks corresponding to major ionised species are indicated. Variation in spectral profiles between retention times reflects the differences in compound composition and ionisation patterns within the sample. Data were acquired under identical instrumental conditions and are presented as representative scans. (ZIP) [file pntd.0014327.s003.zip › EM COMPLETE SAMPLES SPECTRUM/EM14 SPECTRUM RT 2.565.pdf]

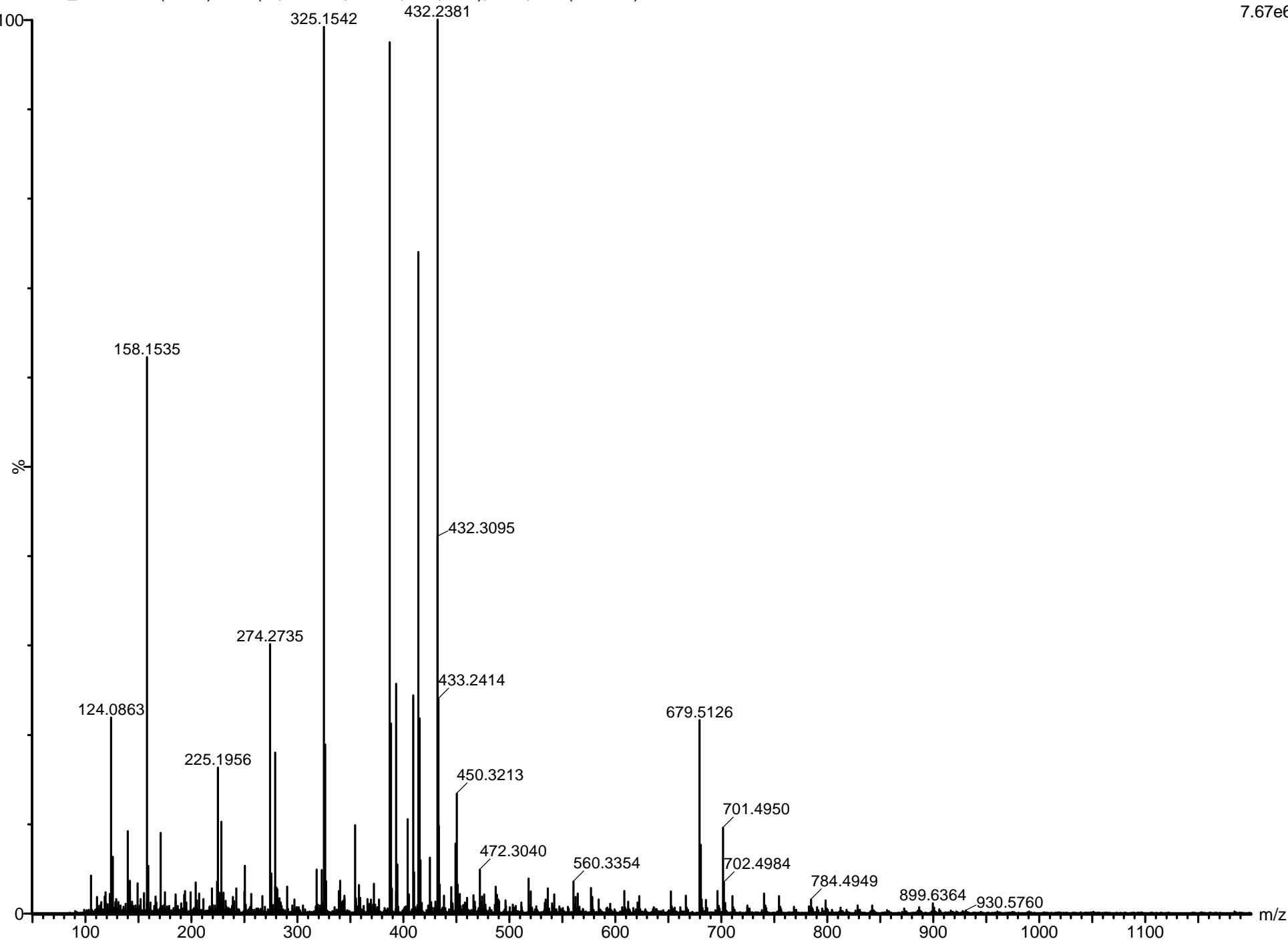

Supplement: S1 Data — Electrospray ionisation time of flight mass spectrometry (ESI-TOF MS, positive mode) spectra of the dengue cohort and ESI-TOF at different retention times. The spectra display the relative abundance (%) of detected ions across the m/z range. Prominent peaks corresponding to major ionised species are indicated. Variation in spectral profiles between retention times reflects the differences in compound composition and ionisation patterns within the sample. Data were acquired under identical instrumental conditions and are presented as representative scans. (ZIP) [file pntd.0014327.s003.zip › EM COMPLETE SAMPLES SPECTRUM/EM14 SPECTRUM RT 2.879.pdf]

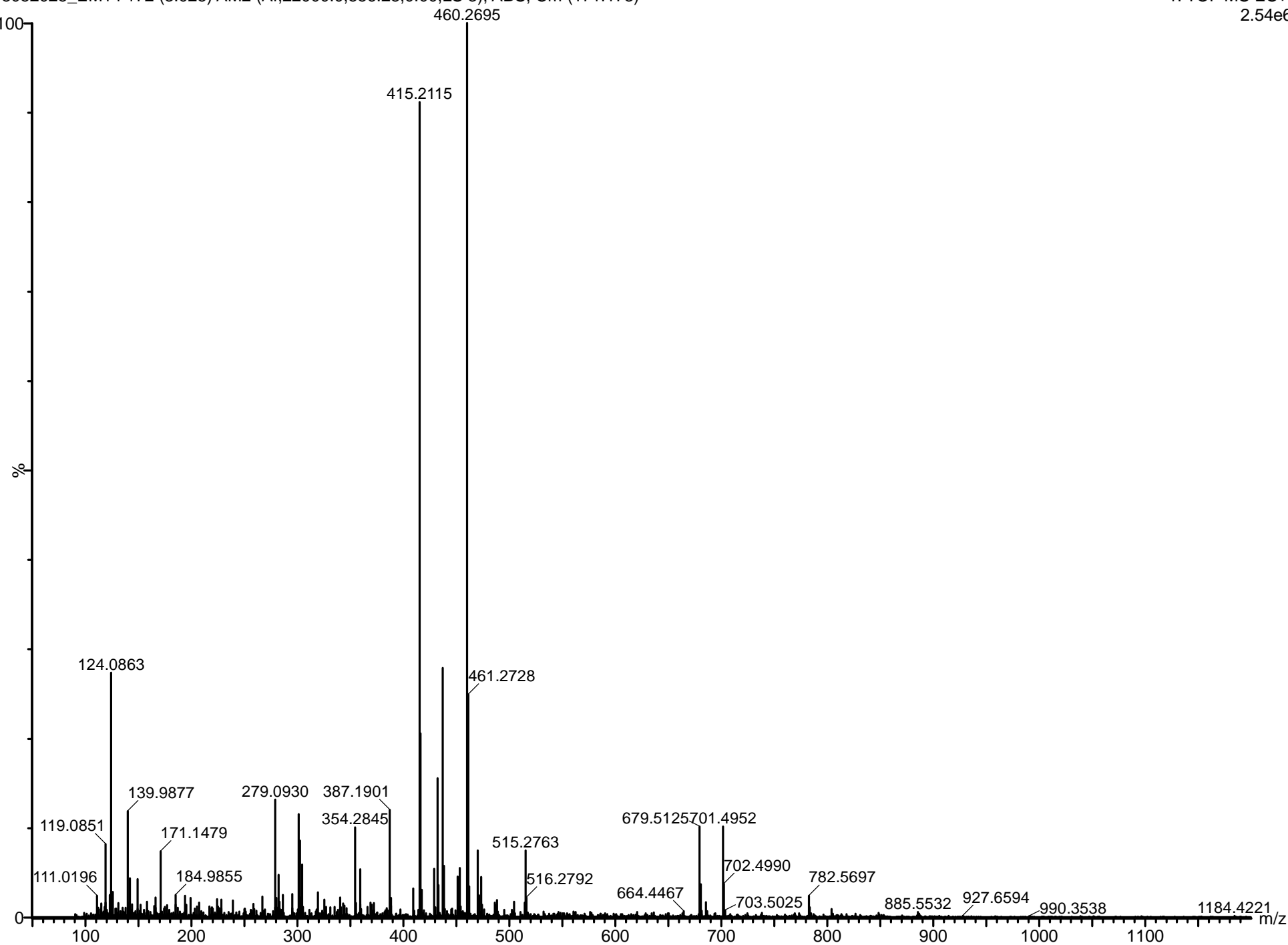

Supplement: S1 Data — Electrospray ionisation time of flight mass spectrometry (ESI-TOF MS, positive mode) spectra of the dengue cohort and ESI-TOF at different retention times. The spectra display the relative abundance (%) of detected ions across the m/z range. Prominent peaks corresponding to major ionised species are indicated. Variation in spectral profiles between retention times reflects the differences in compound composition and ionisation patterns within the sample. Data were acquired under identical instrumental conditions and are presented as representative scans. (ZIP) [file pntd.0014327.s003.zip › EM COMPLETE SAMPLES SPECTRUM/EM14 SPECTRUM RT 3.025.pdf]

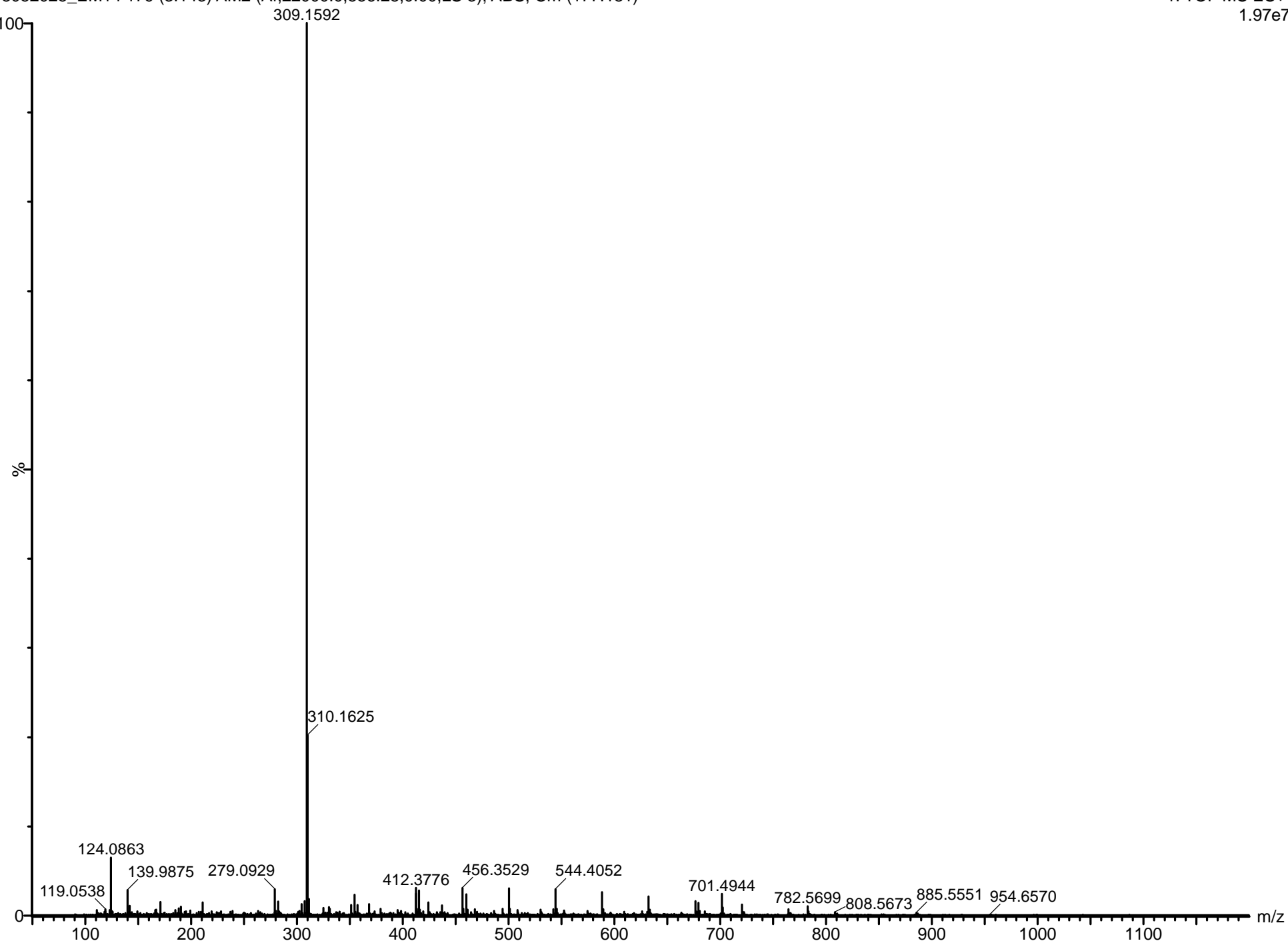

Supplement: S1 Data — Electrospray ionisation time of flight mass spectrometry (ESI-TOF MS, positive mode) spectra of the dengue cohort and ESI-TOF at different retention times. The spectra display the relative abundance (%) of detected ions across the m/z range. Prominent peaks corresponding to major ionised species are indicated. Variation in spectral profiles between retention times reflects the differences in compound composition and ionisation patterns within the sample. Data were acquired under identical instrumental conditions and are presented as representative scans. (ZIP) [file pntd.0014327.s003.zip › EM COMPLETE SAMPLES SPECTRUM/EM14 SPECTRUM RT 3.143.pdf]

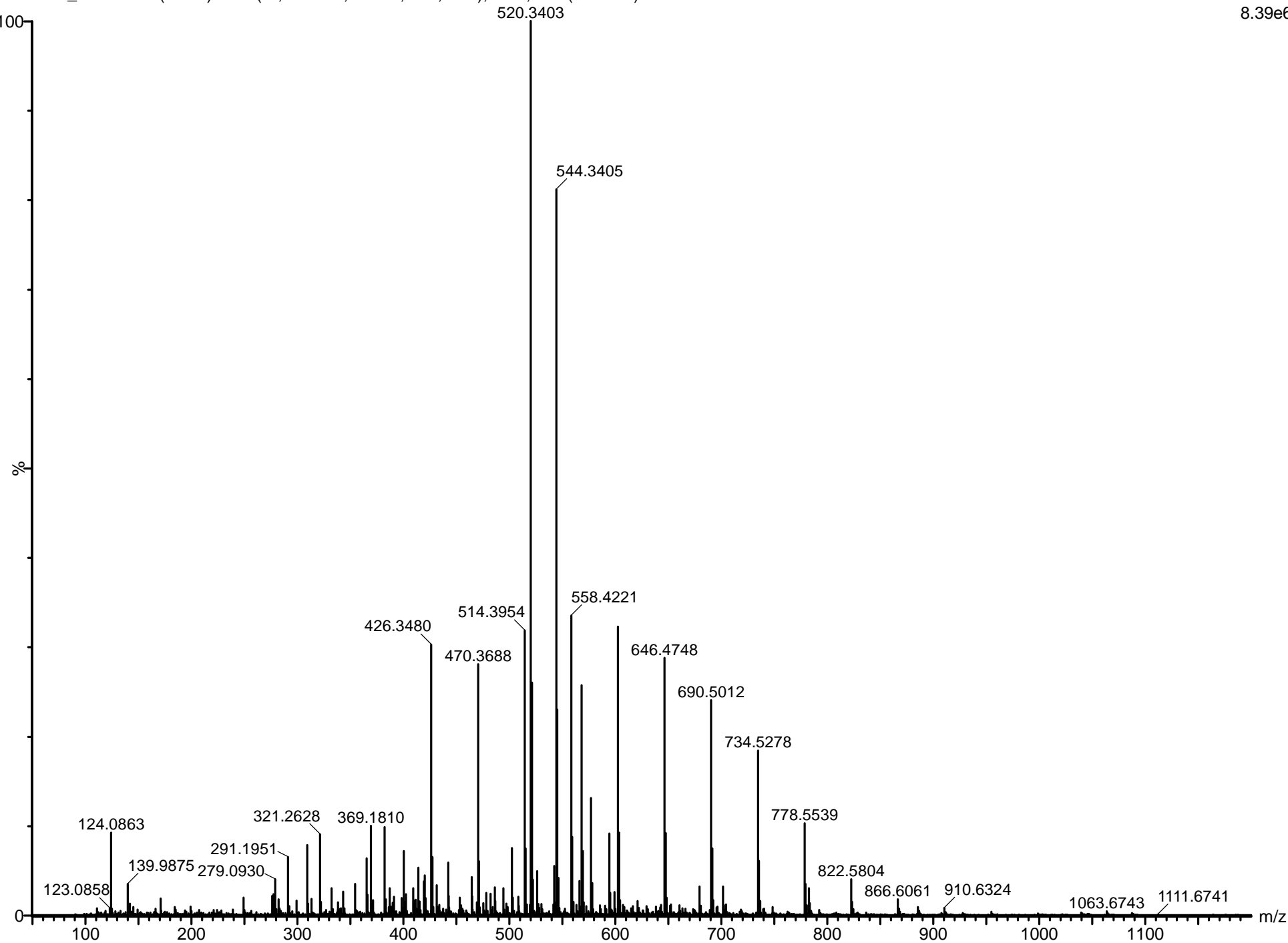

Supplement: S1 Data — Electrospray ionisation time of flight mass spectrometry (ESI-TOF MS, positive mode) spectra of the dengue cohort and ESI-TOF at different retention times. The spectra display the relative abundance (%) of detected ions across the m/z range. Prominent peaks corresponding to major ionised species are indicated. Variation in spectral profiles between retention times reflects the differences in compound composition and ionisation patterns within the sample. Data were acquired under identical instrumental conditions and are presented as representative scans. (ZIP) [file pntd.0014327.s003.zip › EM COMPLETE SAMPLES SPECTRUM/EM14 SPECTRUM RT 3.279.pdf]

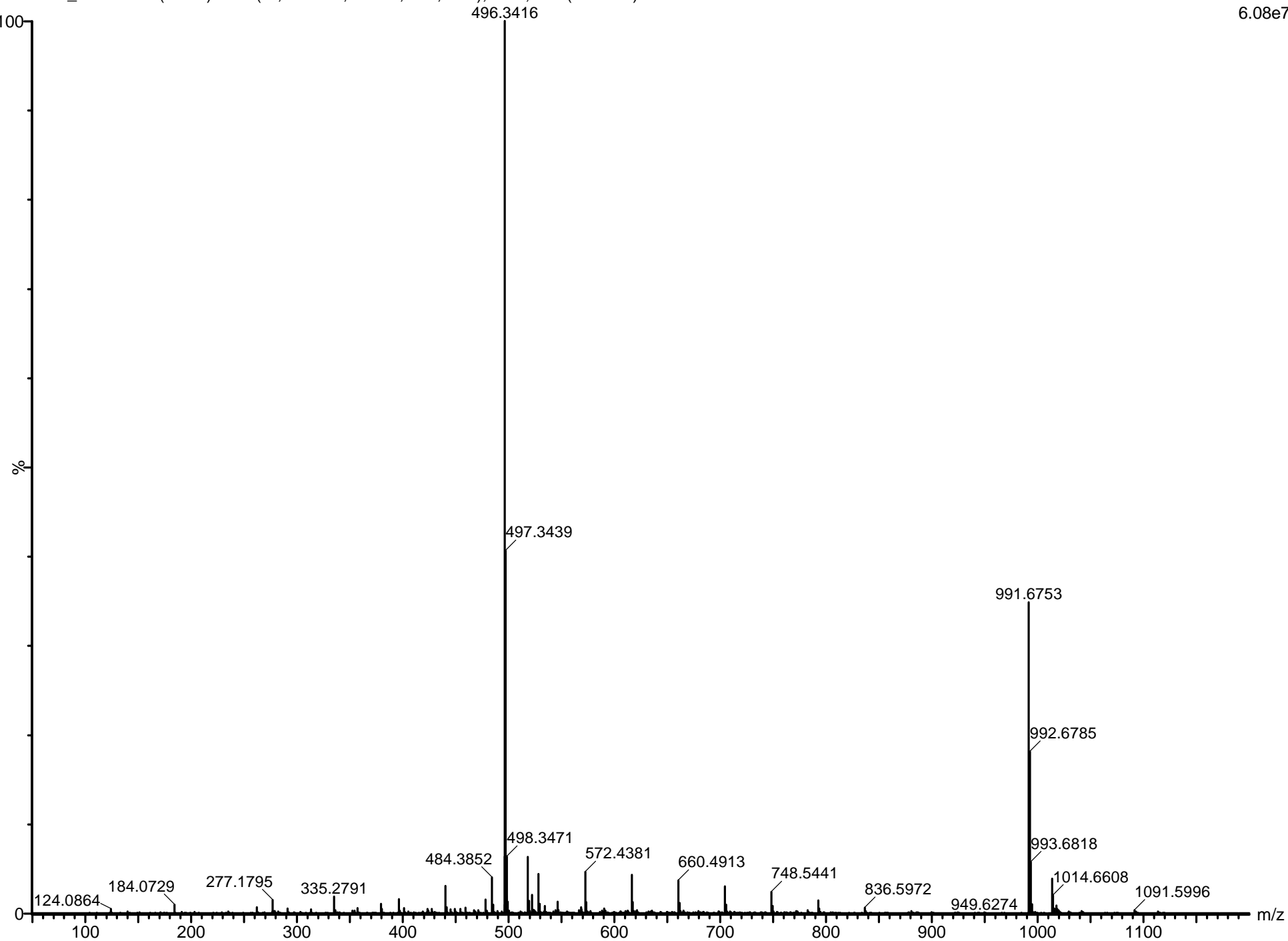

Supplement: S1 Data — Electrospray ionisation time of flight mass spectrometry (ESI-TOF MS, positive mode) spectra of the dengue cohort and ESI-TOF at different retention times. The spectra display the relative abundance (%) of detected ions across the m/z range. Prominent peaks corresponding to major ionised species are indicated. Variation in spectral profiles between retention times reflects the differences in compound composition and ionisation patterns within the sample. Data were acquired under identical instrumental conditions and are presented as representative scans. (ZIP) [file pntd.0014327.s003.zip › EM COMPLETE SAMPLES SPECTRUM/EM14 SPECTRUM RT 3.434.pdf]

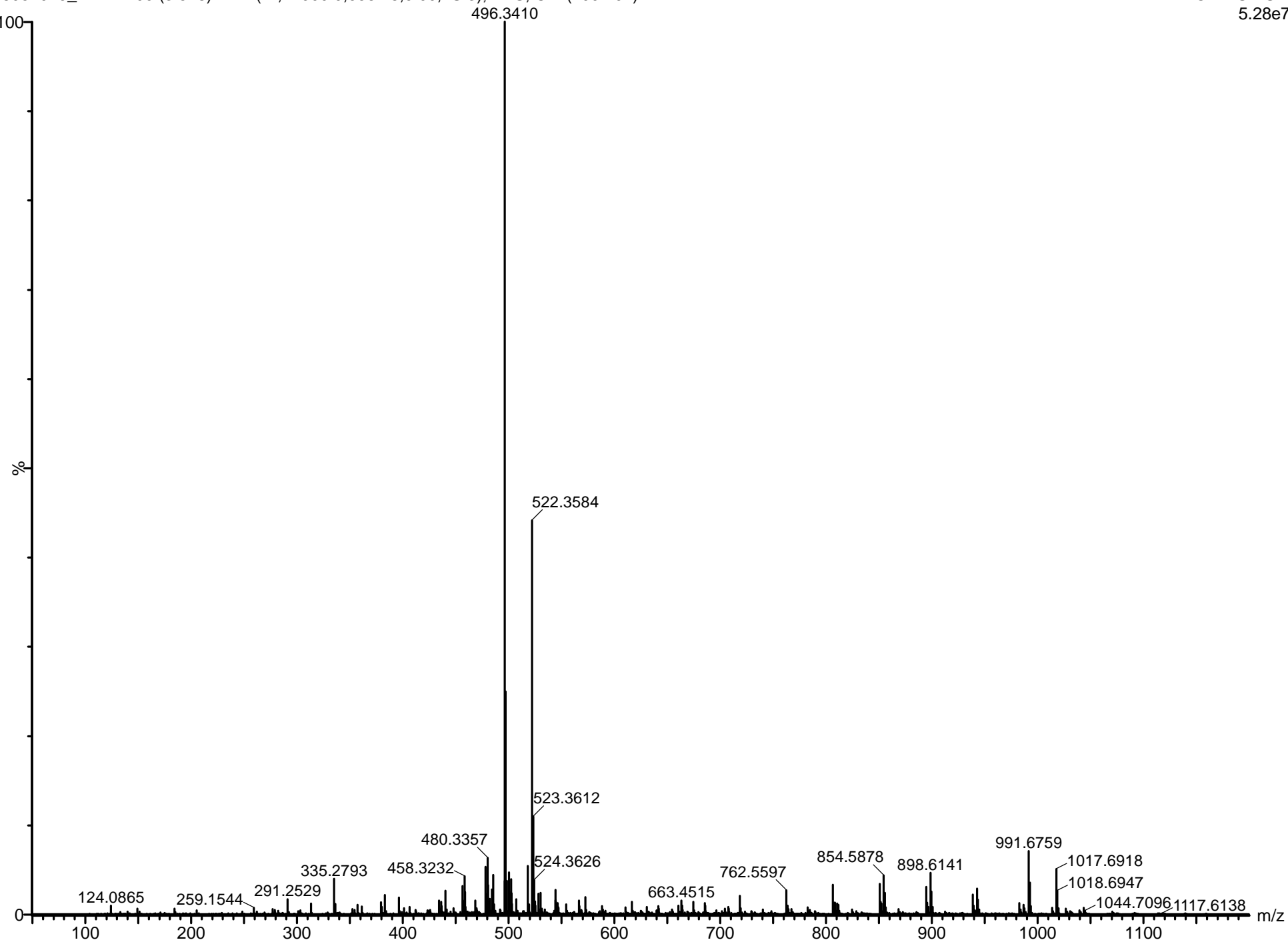

Supplement: S1 Data — Electrospray ionisation time of flight mass spectrometry (ESI-TOF MS, positive mode) spectra of the dengue cohort and ESI-TOF at different retention times. The spectra display the relative abundance (%) of detected ions across the m/z range. Prominent peaks corresponding to major ionised species are indicated. Variation in spectral profiles between retention times reflects the differences in compound composition and ionisation patterns within the sample. Data were acquired under identical instrumental conditions and are presented as representative scans. (ZIP) [file pntd.0014327.s003.zip › EM COMPLETE SAMPLES SPECTRUM/EM14 SPECTRUM RT 3.518.pdf]

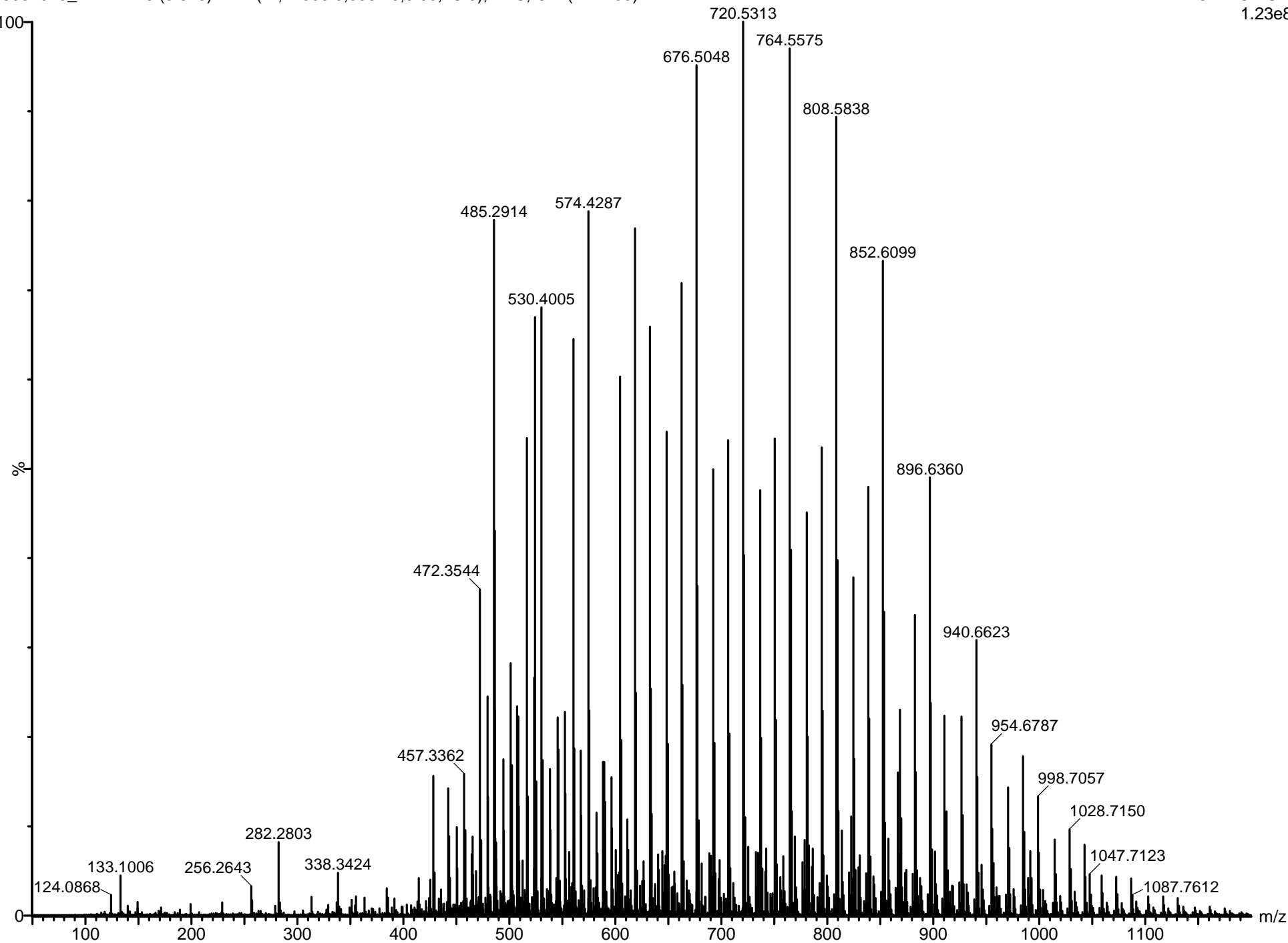

Supplement: S1 Data — Electrospray ionisation time of flight mass spectrometry (ESI-TOF MS, positive mode) spectra of the dengue cohort and ESI-TOF at different retention times. The spectra display the relative abundance (%) of detected ions across the m/z range. Prominent peaks corresponding to major ionised species are indicated. Variation in spectral profiles between retention times reflects the differences in compound composition and ionisation patterns within the sample. Data were acquired under identical instrumental conditions and are presented as representative scans. (ZIP) [file pntd.0014327.s003.zip › EM COMPLETE SAMPLES SPECTRUM/EM14 SPECTRUM RT 3.823.pdf]

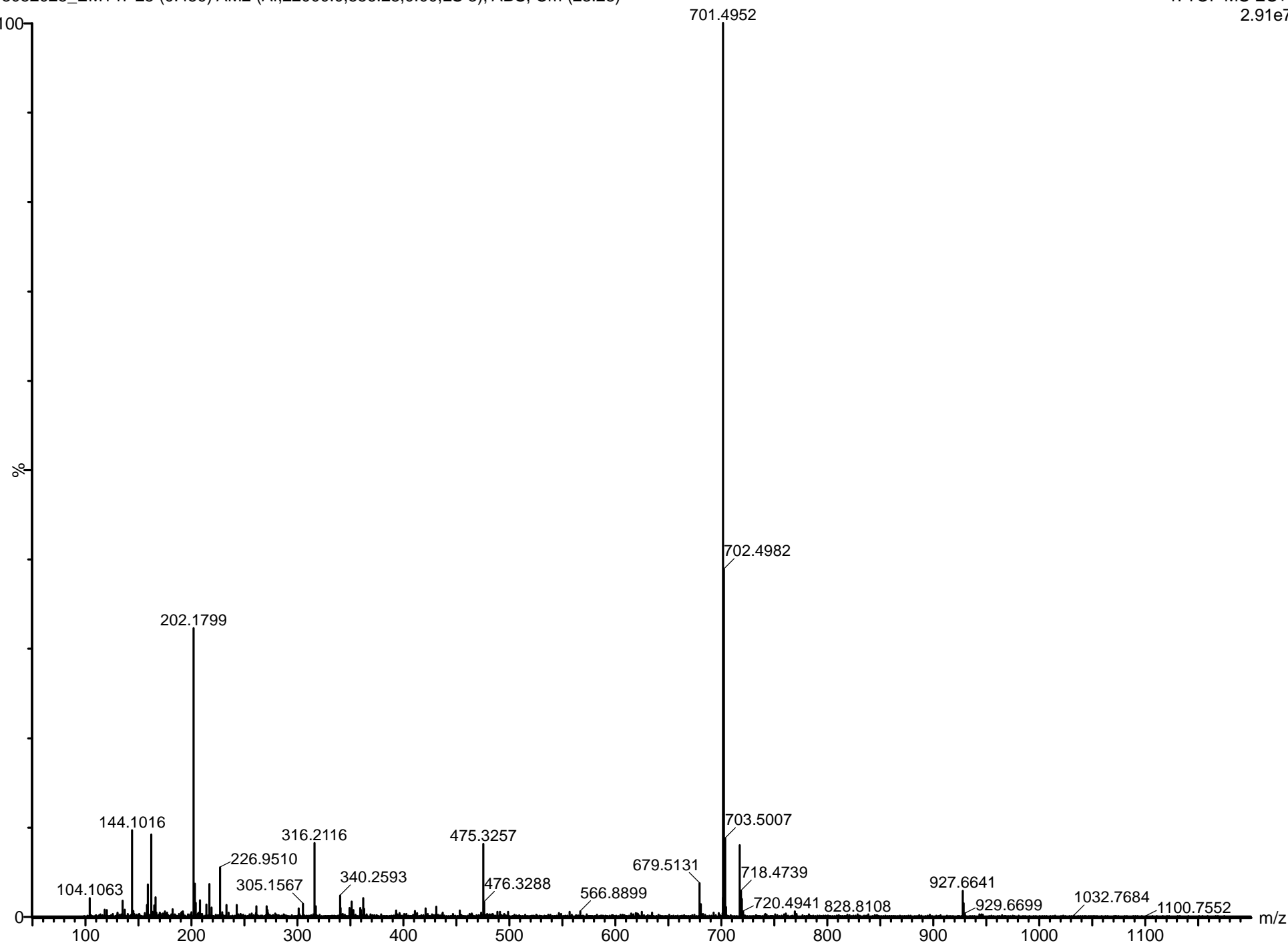

Supplement: S1 Data — Electrospray ionisation time of flight mass spectrometry (ESI-TOF MS, positive mode) spectra of the dengue cohort and ESI-TOF at different retention times. The spectra display the relative abundance (%) of detected ions across the m/z range. Prominent peaks corresponding to major ionised species are indicated. Variation in spectral profiles between retention times reflects the differences in compound composition and ionisation patterns within the sample. Data were acquired under identical instrumental conditions and are presented as representative scans. (ZIP) [file pntd.0014327.s003.zip › EM COMPLETE SAMPLES SPECTRUM/EM147 SPECTRUM RT 0.459.pdf]

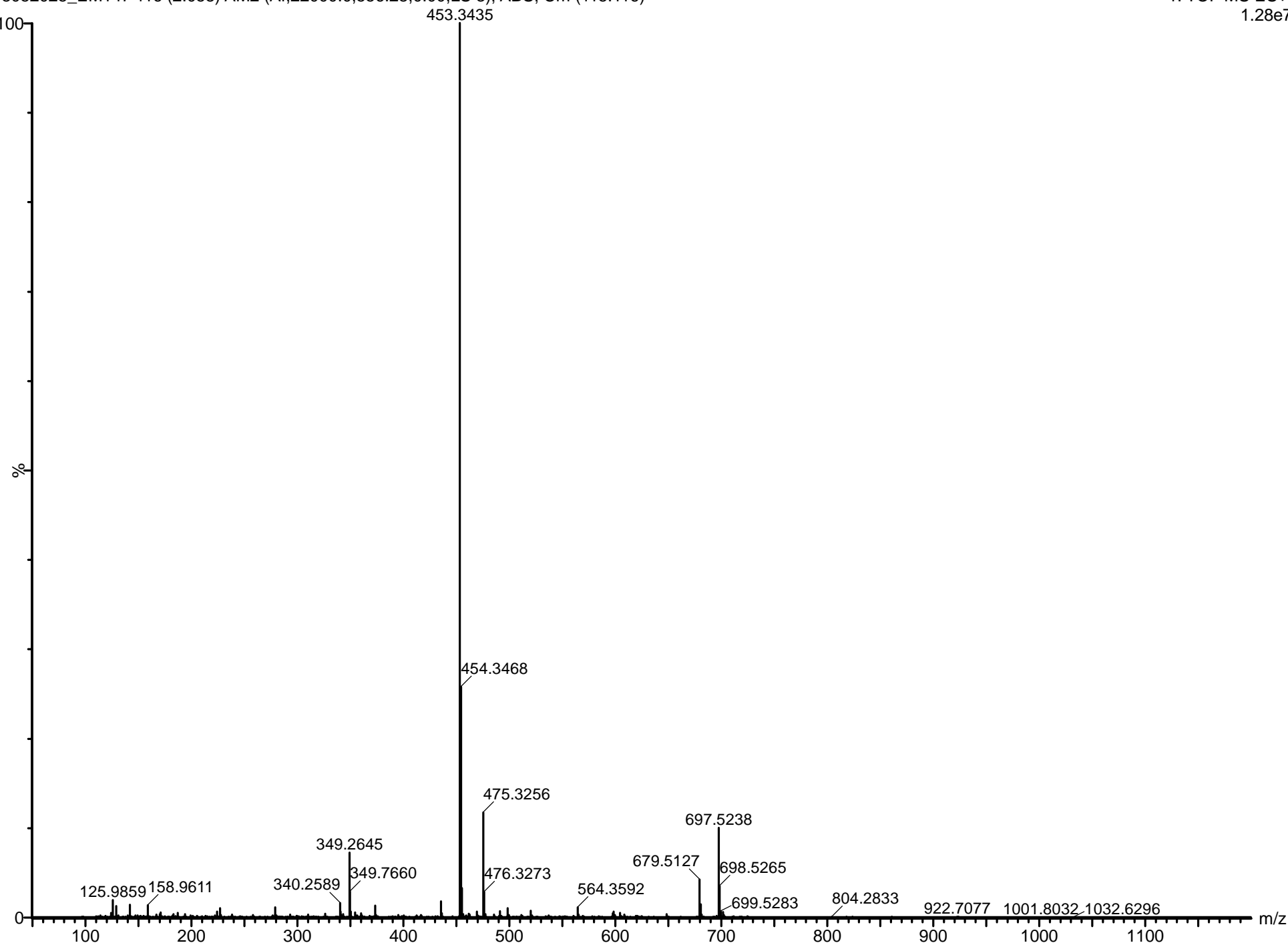

Supplement: S1 Data — Electrospray ionisation time of flight mass spectrometry (ESI-TOF MS, positive mode) spectra of the dengue cohort and ESI-TOF at different retention times. The spectra display the relative abundance (%) of detected ions across the m/z range. Prominent peaks corresponding to major ionised species are indicated. Variation in spectral profiles between retention times reflects the differences in compound composition and ionisation patterns within the sample. Data were acquired under identical instrumental conditions and are presented as representative scans. (ZIP) [file pntd.0014327.s003.zip › EM COMPLETE SAMPLES SPECTRUM/EM147 SPECTRUM RT 2.058.pdf]

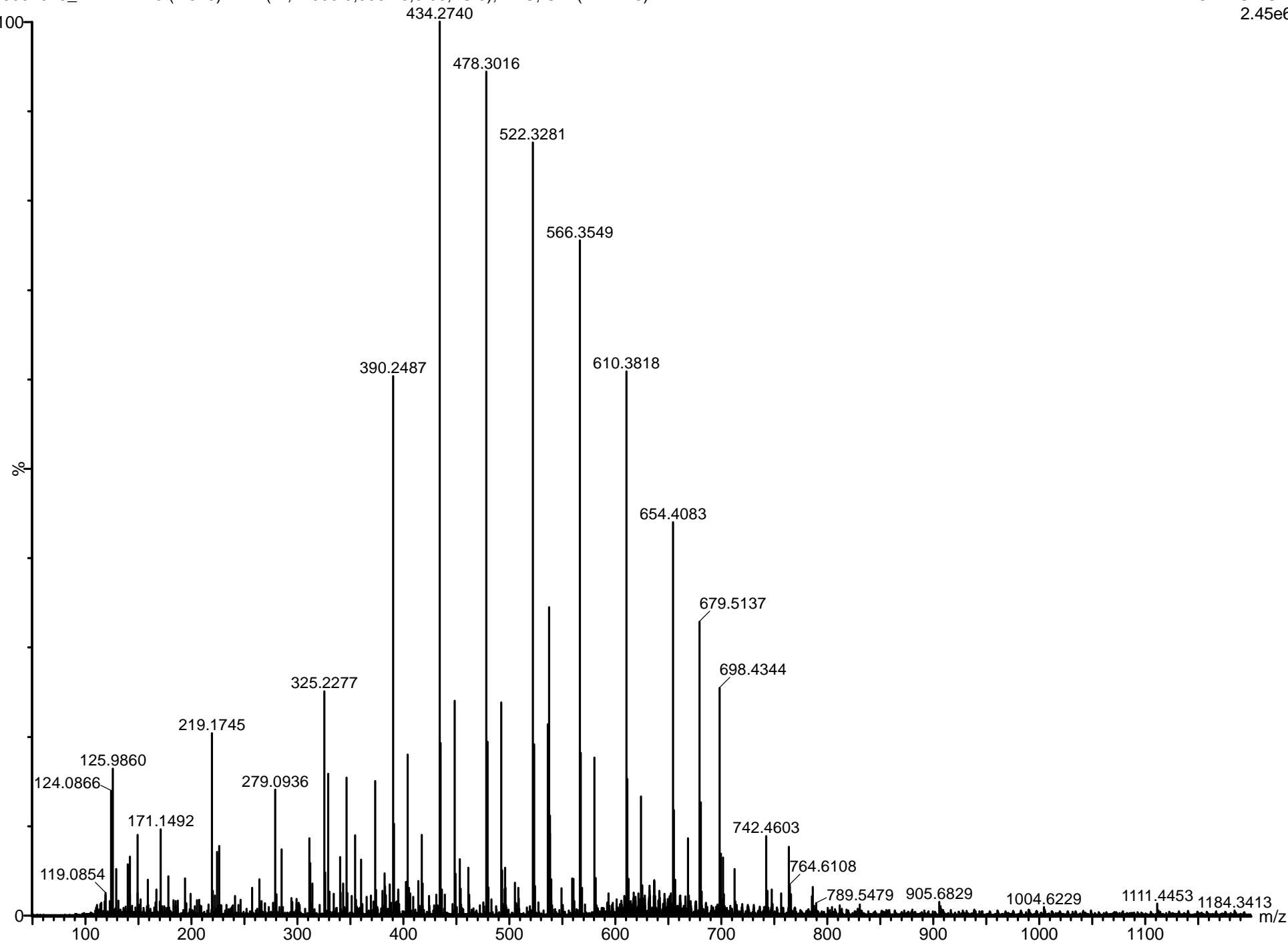

Supplement: S1 Data — Electrospray ionisation time of flight mass spectrometry (ESI-TOF MS, positive mode) spectra of the dengue cohort and ESI-TOF at different retention times. The spectra display the relative abundance (%) of detected ions across the m/z range. Prominent peaks corresponding to major ionised species are indicated. Variation in spectral profiles between retention times reflects the differences in compound composition and ionisation patterns within the sample. Data were acquired under identical instrumental conditions and are presented as representative scans. (ZIP) [file pntd.0014327.s003.zip › EM COMPLETE SAMPLES SPECTRUM/EM147 SPECTRUM RT 2.515.pdf]

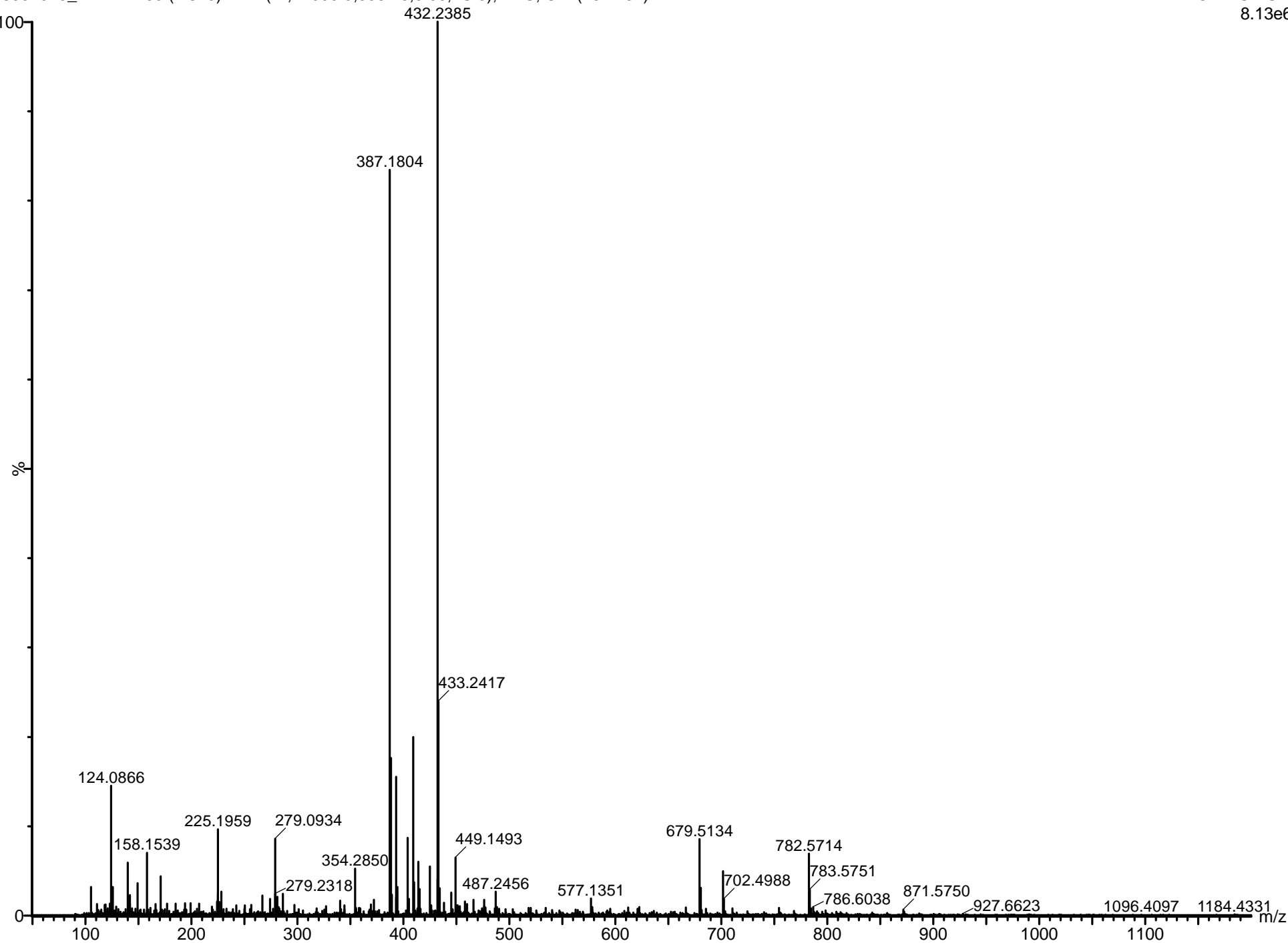

Supplement: S1 Data — Electrospray ionisation time of flight mass spectrometry (ESI-TOF MS, positive mode) spectra of the dengue cohort and ESI-TOF at different retention times. The spectra display the relative abundance (%) of detected ions across the m/z range. Prominent peaks corresponding to major ionised species are indicated. Variation in spectral profiles between retention times reflects the differences in compound composition and ionisation patterns within the sample. Data were acquired under identical instrumental conditions and are presented as representative scans. (ZIP) [file pntd.0014327.s003.zip › EM COMPLETE SAMPLES SPECTRUM/EM147 SPECTRUM RT 2.879.pdf]

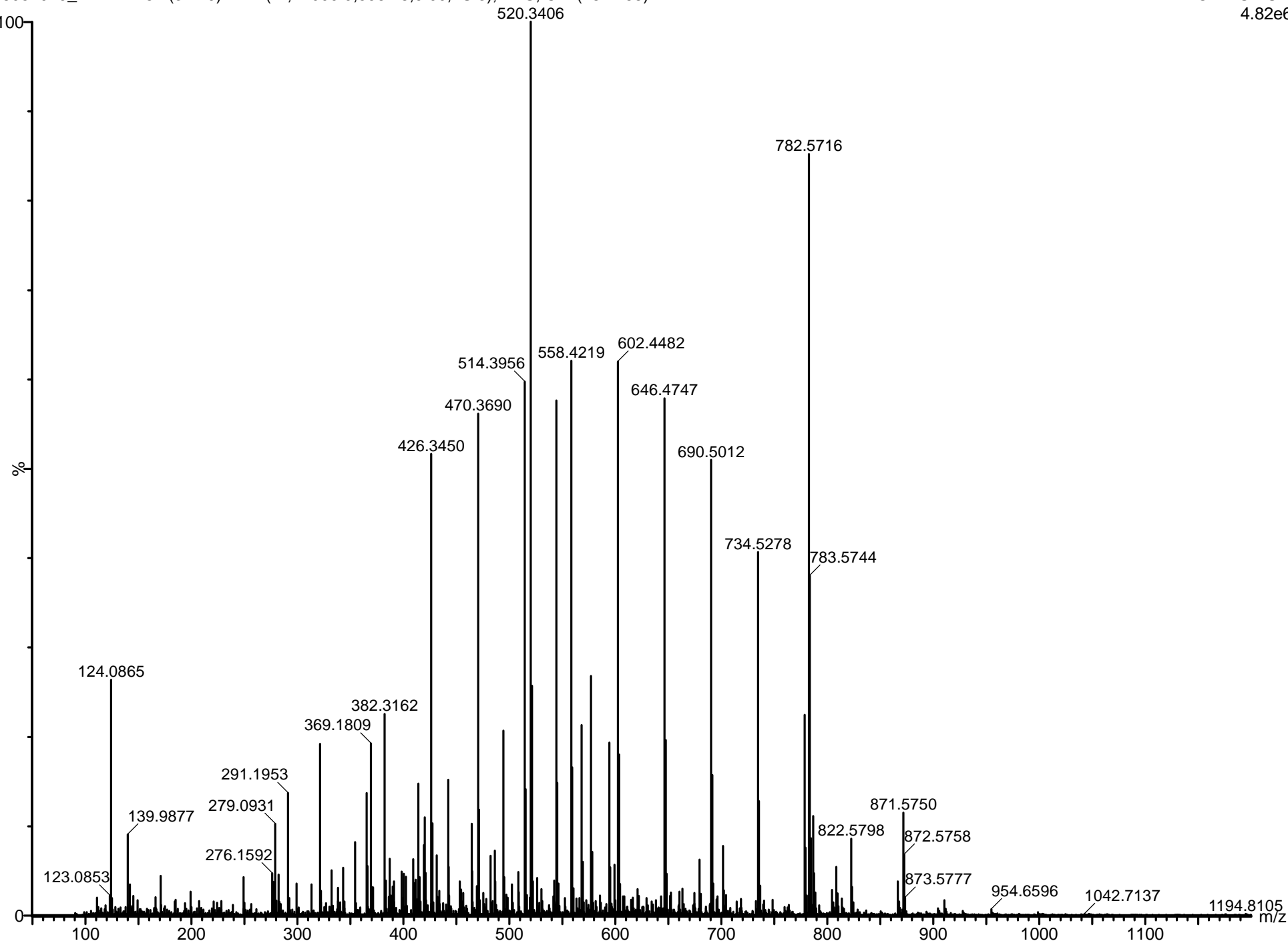

Supplement: S1 Data — Electrospray ionisation time of flight mass spectrometry (ESI-TOF MS, positive mode) spectra of the dengue cohort and ESI-TOF at different retention times. The spectra display the relative abundance (%) of detected ions across the m/z range. Prominent peaks corresponding to major ionised species are indicated. Variation in spectral profiles between retention times reflects the differences in compound composition and ionisation patterns within the sample. Data were acquired under identical instrumental conditions and are presented as representative scans. (ZIP) [file pntd.0014327.s003.zip › EM COMPLETE SAMPLES SPECTRUM/EM147 SPECTRUM RT 3.279.pdf]

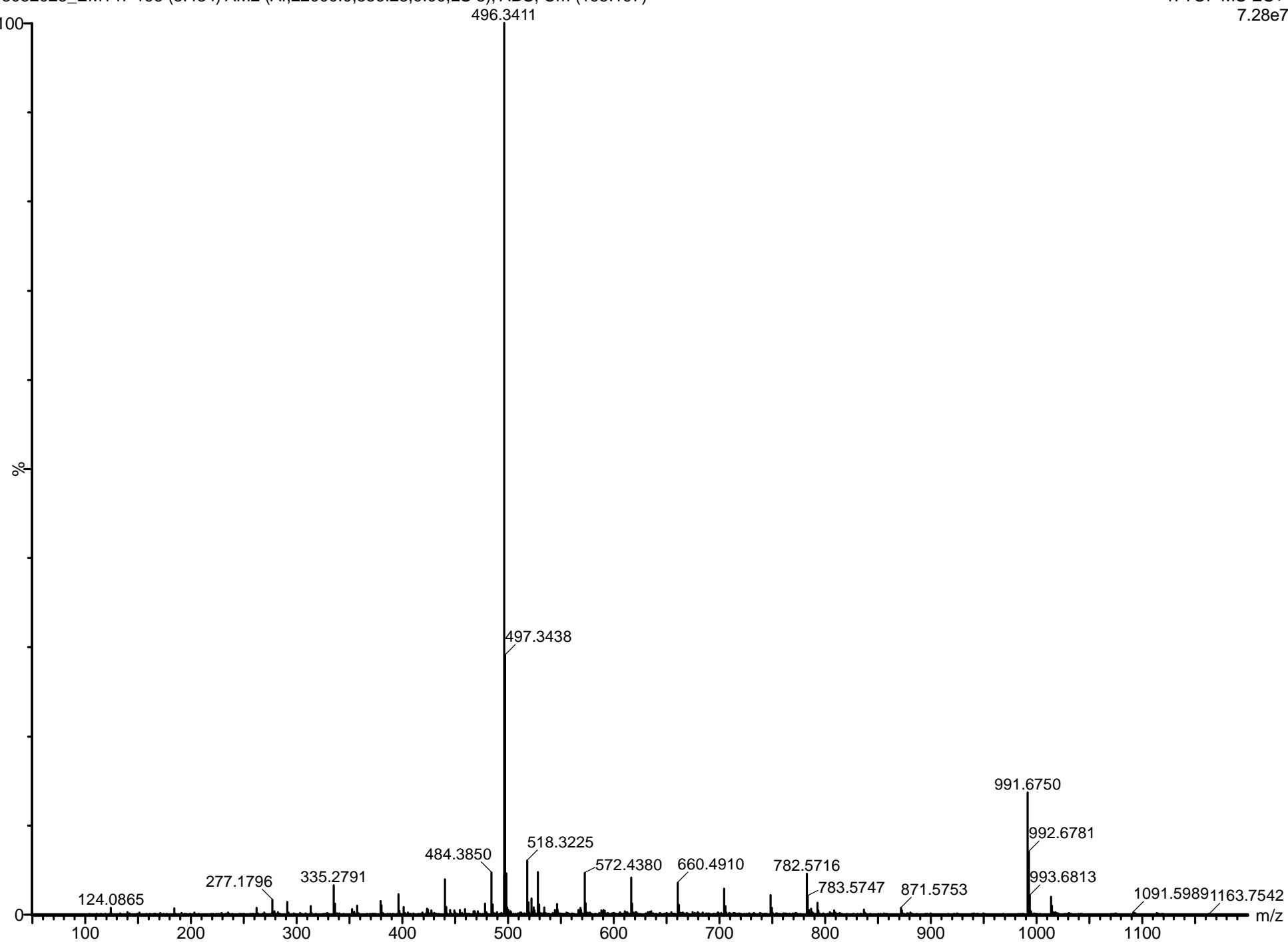

Supplement: S1 Data — Electrospray ionisation time of flight mass spectrometry (ESI-TOF MS, positive mode) spectra of the dengue cohort and ESI-TOF at different retention times. The spectra display the relative abundance (%) of detected ions across the m/z range. Prominent peaks corresponding to major ionised species are indicated. Variation in spectral profiles between retention times reflects the differences in compound composition and ionisation patterns within the sample. Data were acquired under identical instrumental conditions and are presented as representative scans. (ZIP) [file pntd.0014327.s003.zip › EM COMPLETE SAMPLES SPECTRUM/EM147 SPECTRUM RT 3.434.pdf]

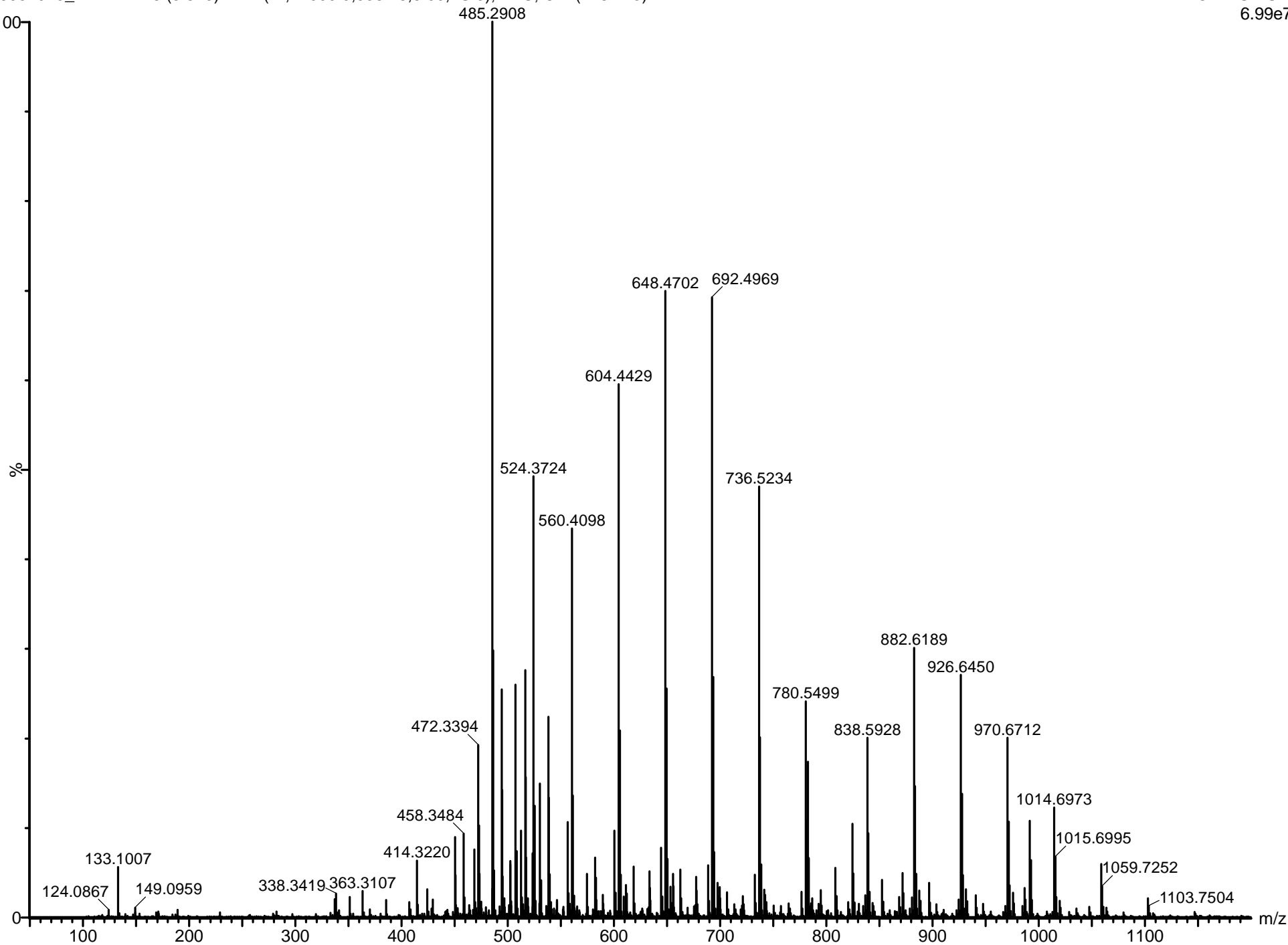

Supplement: S1 Data — Electrospray ionisation time of flight mass spectrometry (ESI-TOF MS, positive mode) spectra of the dengue cohort and ESI-TOF at different retention times. The spectra display the relative abundance (%) of detected ions across the m/z range. Prominent peaks corresponding to major ionised species are indicated. Variation in spectral profiles between retention times reflects the differences in compound composition and ionisation patterns within the sample. Data were acquired under identical instrumental conditions and are presented as representative scans. (ZIP) [file pntd.0014327.s003.zip › EM COMPLETE SAMPLES SPECTRUM/EM147 SPECTRUM RT 3.823.pdf]

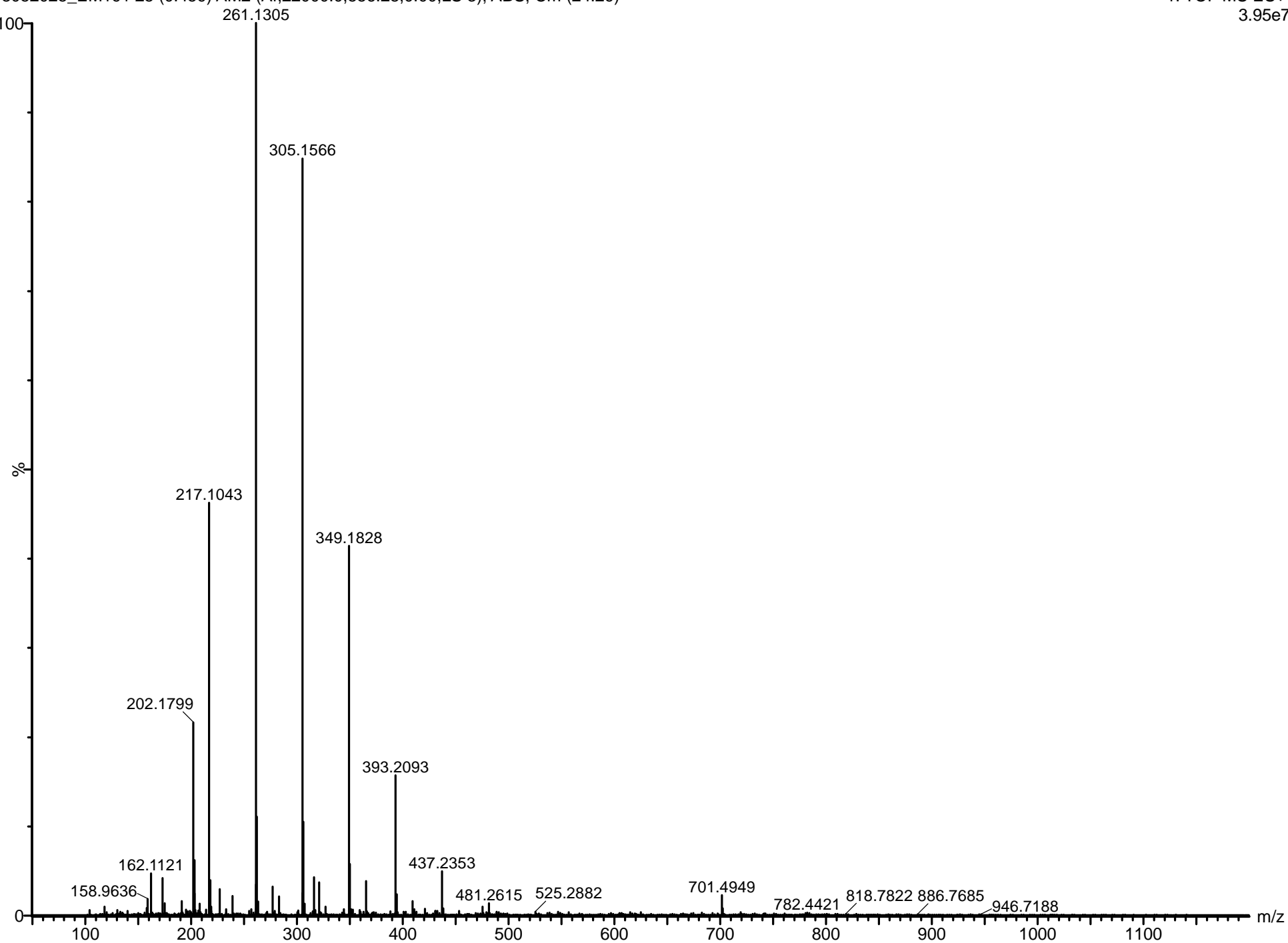

Supplement: S1 Data — Electrospray ionisation time of flight mass spectrometry (ESI-TOF MS, positive mode) spectra of the dengue cohort and ESI-TOF at different retention times. The spectra display the relative abundance (%) of detected ions across the m/z range. Prominent peaks corresponding to major ionised species are indicated. Variation in spectral profiles between retention times reflects the differences in compound composition and ionisation patterns within the sample. Data were acquired under identical instrumental conditions and are presented as representative scans. (ZIP) [file pntd.0014327.s003.zip › EM COMPLETE SAMPLES SPECTRUM/EM191 SPECTRUM RT 0.459.pdf]

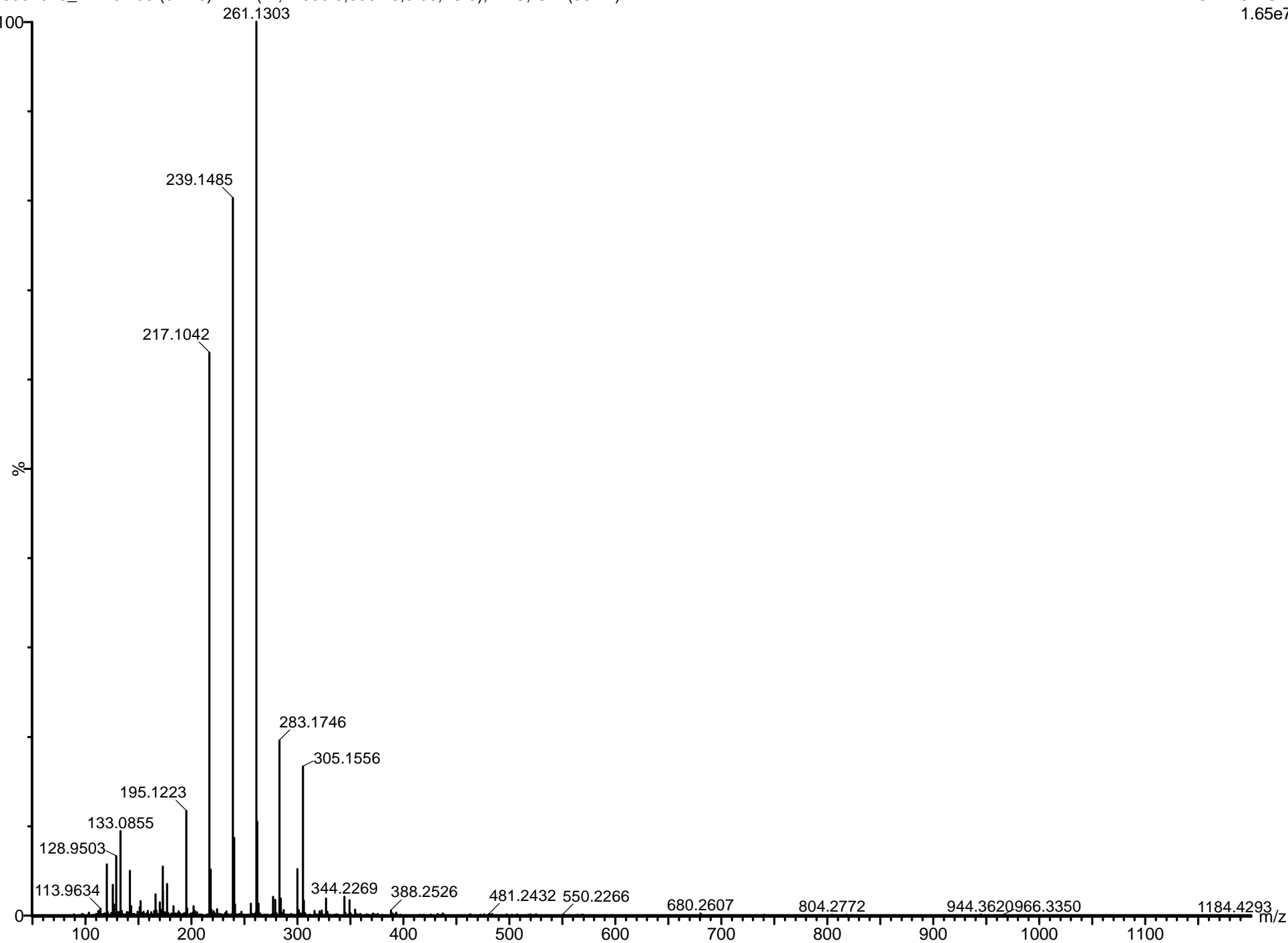

Supplement: S1 Data — Electrospray ionisation time of flight mass spectrometry (ESI-TOF MS, positive mode) spectra of the dengue cohort and ESI-TOF at different retention times. The spectra display the relative abundance (%) of detected ions across the m/z range. Prominent peaks corresponding to major ionised species are indicated. Variation in spectral profiles between retention times reflects the differences in compound composition and ionisation patterns within the sample. Data were acquired under identical instrumental conditions and are presented as representative scans. (ZIP) [file pntd.0014327.s003.zip › EM COMPLETE SAMPLES SPECTRUM/EM191 SPECTRUM RT 0.716.pdf]

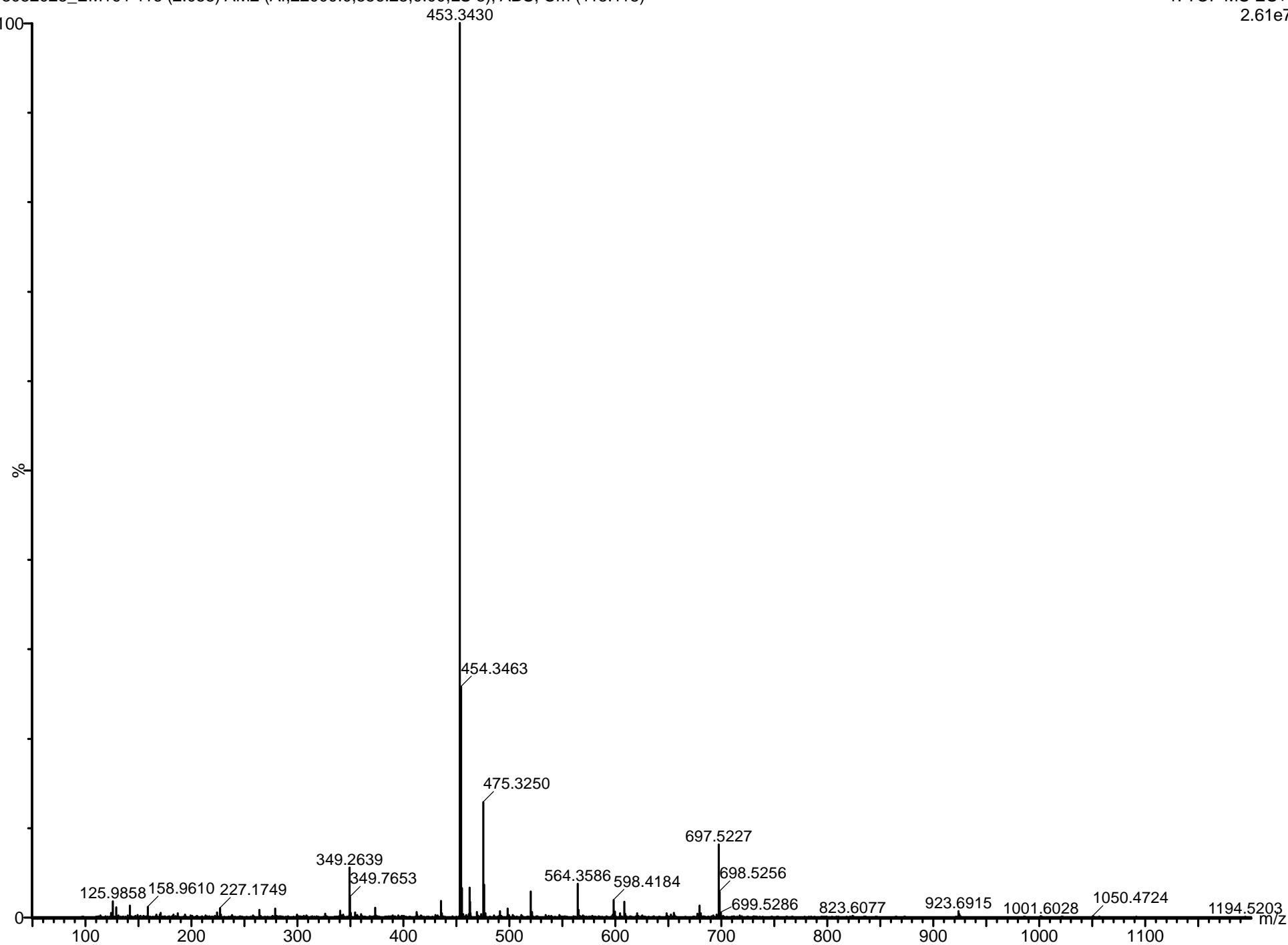

Supplement: S1 Data — Electrospray ionisation time of flight mass spectrometry (ESI-TOF MS, positive mode) spectra of the dengue cohort and ESI-TOF at different retention times. The spectra display the relative abundance (%) of detected ions across the m/z range. Prominent peaks corresponding to major ionised species are indicated. Variation in spectral profiles between retention times reflects the differences in compound composition and ionisation patterns within the sample. Data were acquired under identical instrumental conditions and are presented as representative scans. (ZIP) [file pntd.0014327.s003.zip › EM COMPLETE SAMPLES SPECTRUM/EM191 SPECTRUM RT 2.058.pdf]

18052025\_EM191 146 (2.565) AM2 (Ar,22000.0,556.28,0.00,LS 3); ABS; Cm (142:148)

1: TOF MS ES+  
2.98e6

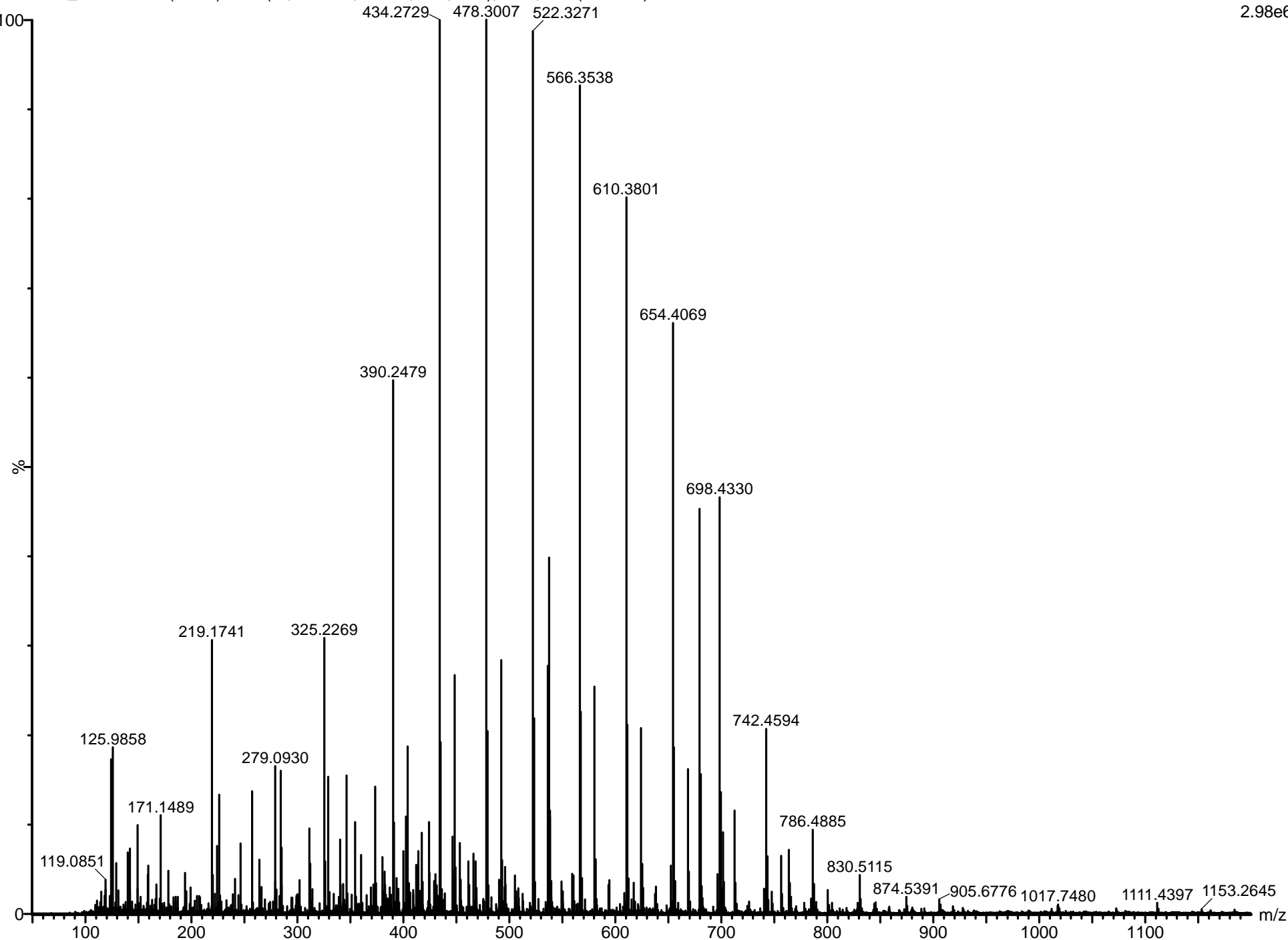

Supplement: S1 Data — Electrospray ionisation time of flight mass spectrometry (ESI-TOF MS, positive mode) spectra of the dengue cohort and ESI-TOF at different retention times. The spectra display the relative abundance (%) of detected ions across the m/z range. Prominent peaks corresponding to major ionised species are indicated. Variation in spectral profiles between retention times reflects the differences in compound composition and ionisation patterns within the sample. Data were acquired under identical instrumental conditions and are presented as representative scans. (ZIP) [file pntd.0014327.s003.zip › EM COMPLETE SAMPLES SPECTRUM/EM191 SPECTRUM RT 2.565.pdf]

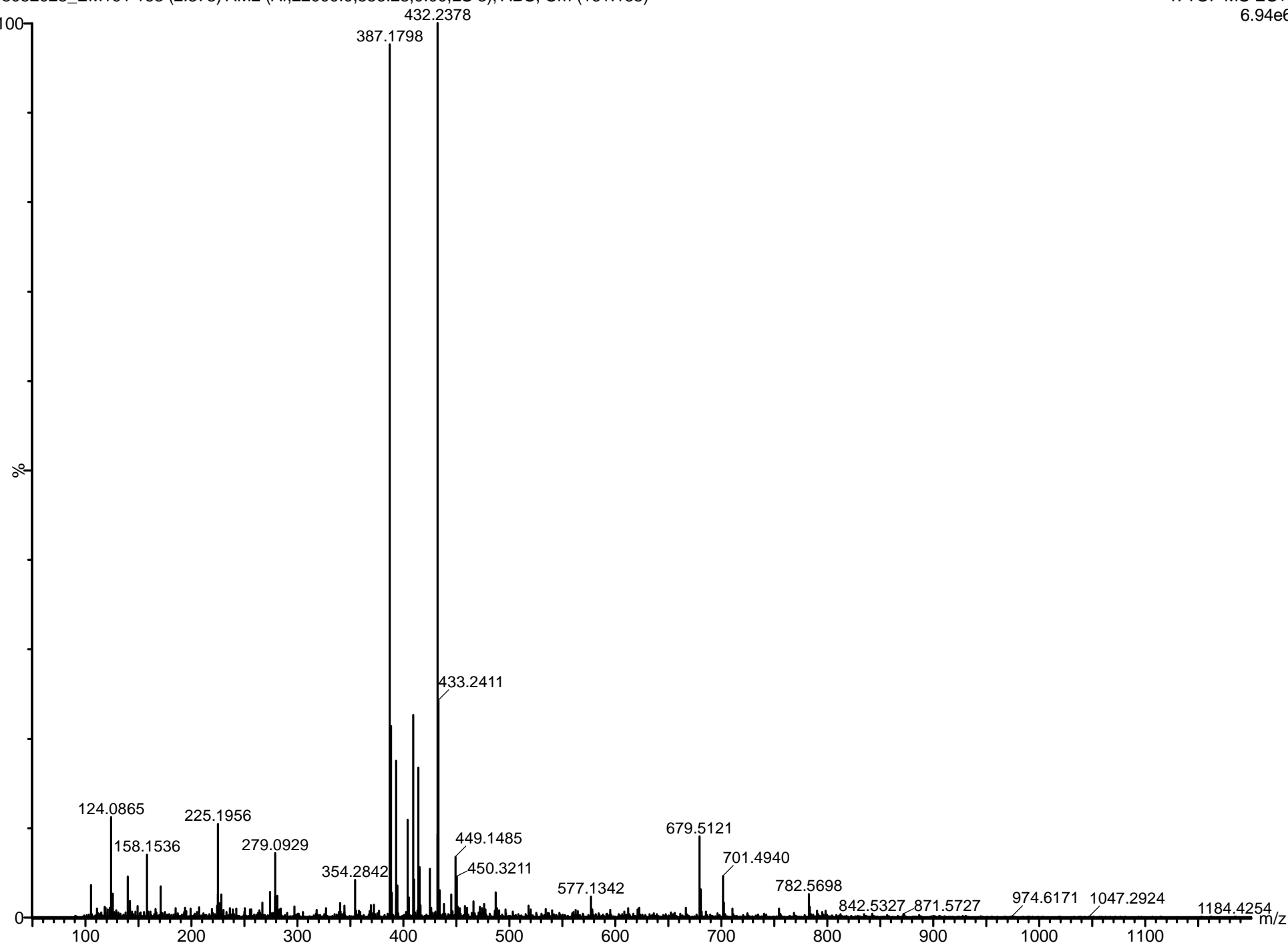

Supplement: S1 Data — Electrospray ionisation time of flight mass spectrometry (ESI-TOF MS, positive mode) spectra of the dengue cohort and ESI-TOF at different retention times. The spectra display the relative abundance (%) of detected ions across the m/z range. Prominent peaks corresponding to major ionised species are indicated. Variation in spectral profiles between retention times reflects the differences in compound composition and ionisation patterns within the sample. Data were acquired under identical instrumental conditions and are presented as representative scans. (ZIP) [file pntd.0014327.s003.zip › EM COMPLETE SAMPLES SPECTRUM/EM191 SPECTRUM RT 2.873.pdf]

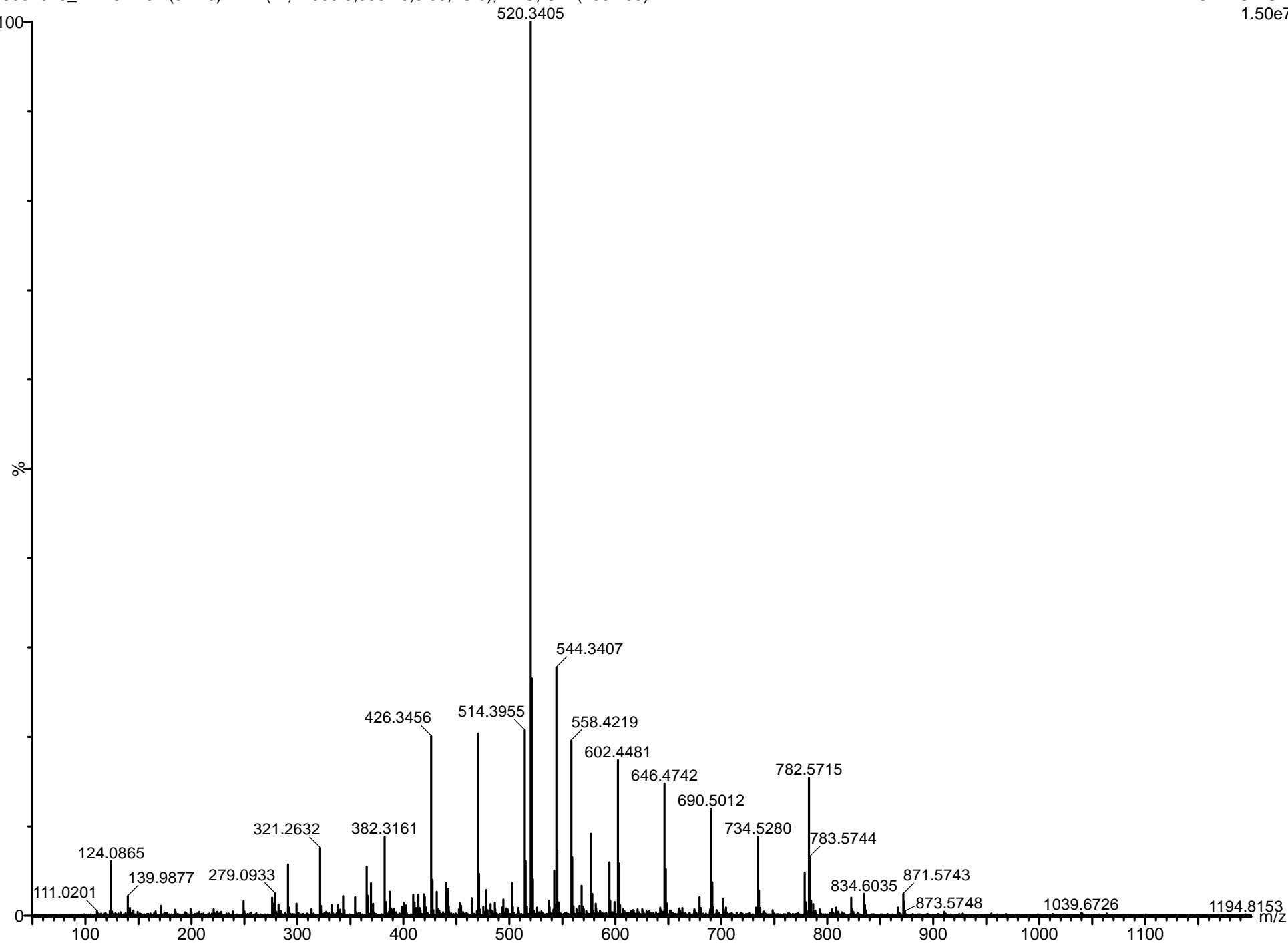

Supplement: S1 Data — Electrospray ionisation time of flight mass spectrometry (ESI-TOF MS, positive mode) spectra of the dengue cohort and ESI-TOF at different retention times. The spectra display the relative abundance (%) of detected ions across the m/z range. Prominent peaks corresponding to major ionised species are indicated. Variation in spectral profiles between retention times reflects the differences in compound composition and ionisation patterns within the sample. Data were acquired under identical instrumental conditions and are presented as representative scans. (ZIP) [file pntd.0014327.s003.zip › EM COMPLETE SAMPLES SPECTRUM/EM191 SPECTRUM RT 3.279.pdf]

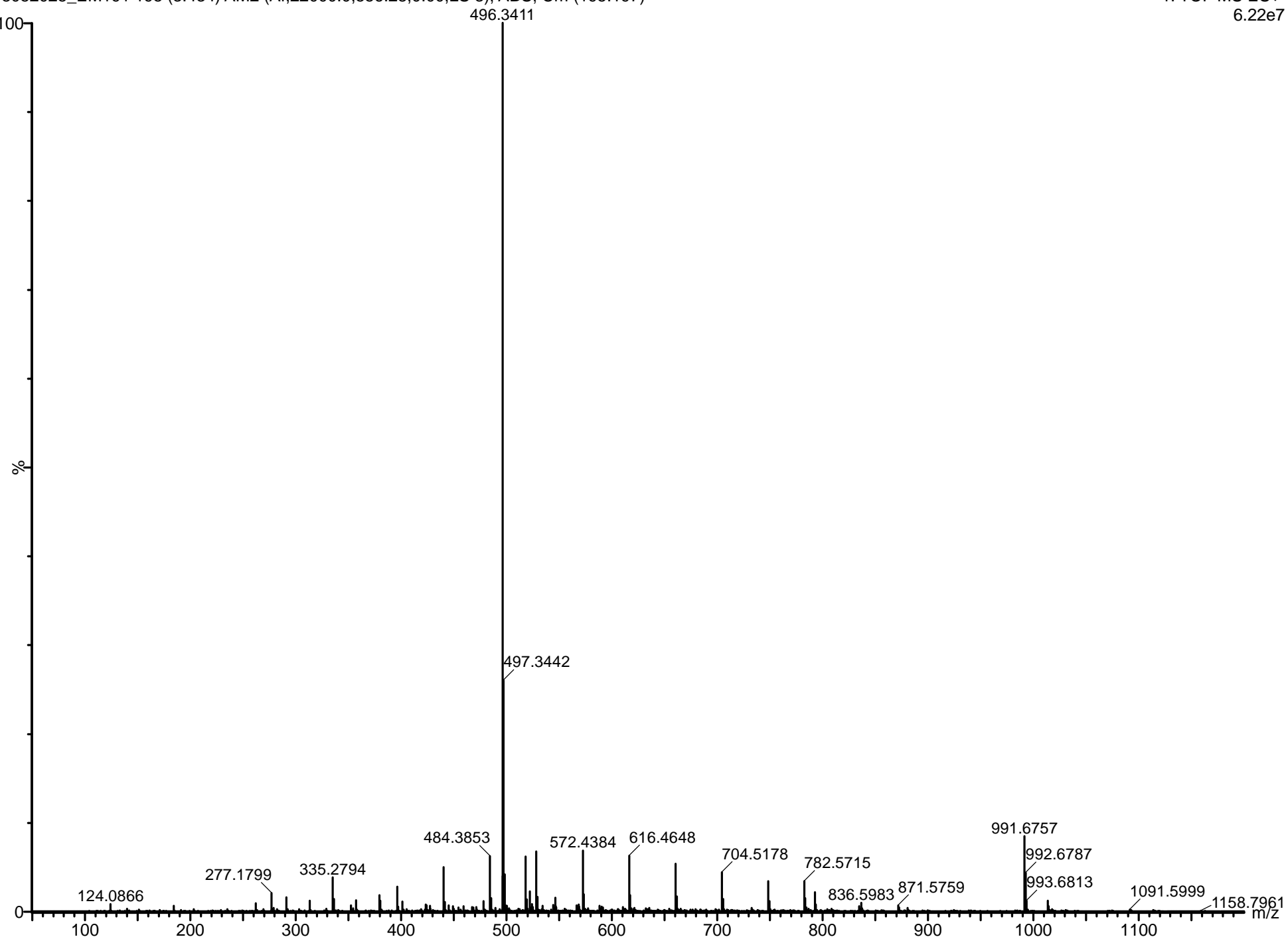

Supplement: S1 Data — Electrospray ionisation time of flight mass spectrometry (ESI-TOF MS, positive mode) spectra of the dengue cohort and ESI-TOF at different retention times. The spectra display the relative abundance (%) of detected ions across the m/z range. Prominent peaks corresponding to major ionised species are indicated. Variation in spectral profiles between retention times reflects the differences in compound composition and ionisation patterns within the sample. Data were acquired under identical instrumental conditions and are presented as representative scans. (ZIP) [file pntd.0014327.s003.zip › EM COMPLETE SAMPLES SPECTRUM/EM191 SPECTRUM RT 3.434.pdf]

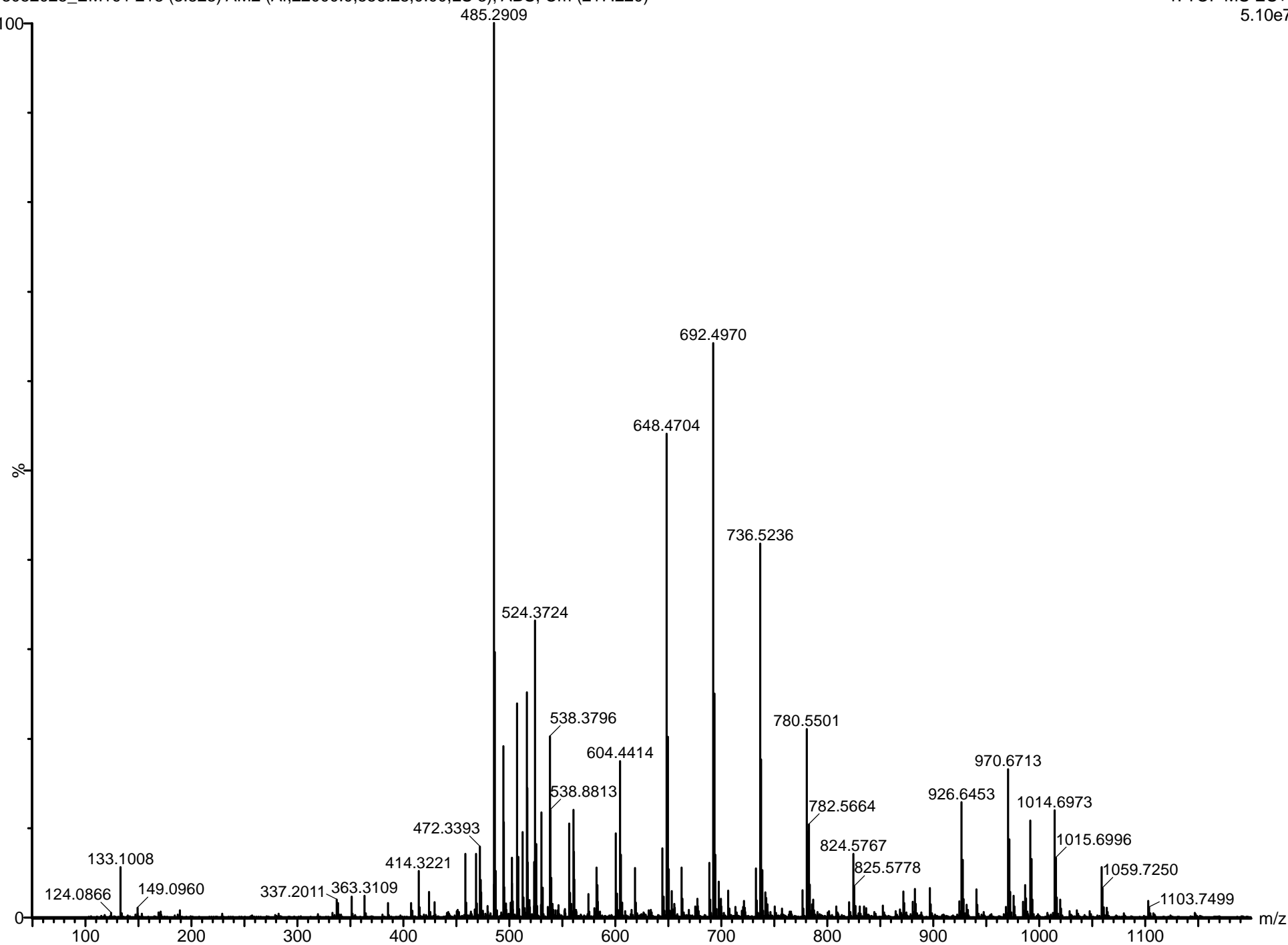

Supplement: S1 Data — Electrospray ionisation time of flight mass spectrometry (ESI-TOF MS, positive mode) spectra of the dengue cohort and ESI-TOF at different retention times. The spectra display the relative abundance (%) of detected ions across the m/z range. Prominent peaks corresponding to major ionised species are indicated. Variation in spectral profiles between retention times reflects the differences in compound composition and ionisation patterns within the sample. Data were acquired under identical instrumental conditions and are presented as representative scans. (ZIP) [file pntd.0014327.s003.zip › EM COMPLETE SAMPLES SPECTRUM/EM191 SPECTRUM RT 3.823.pdf]

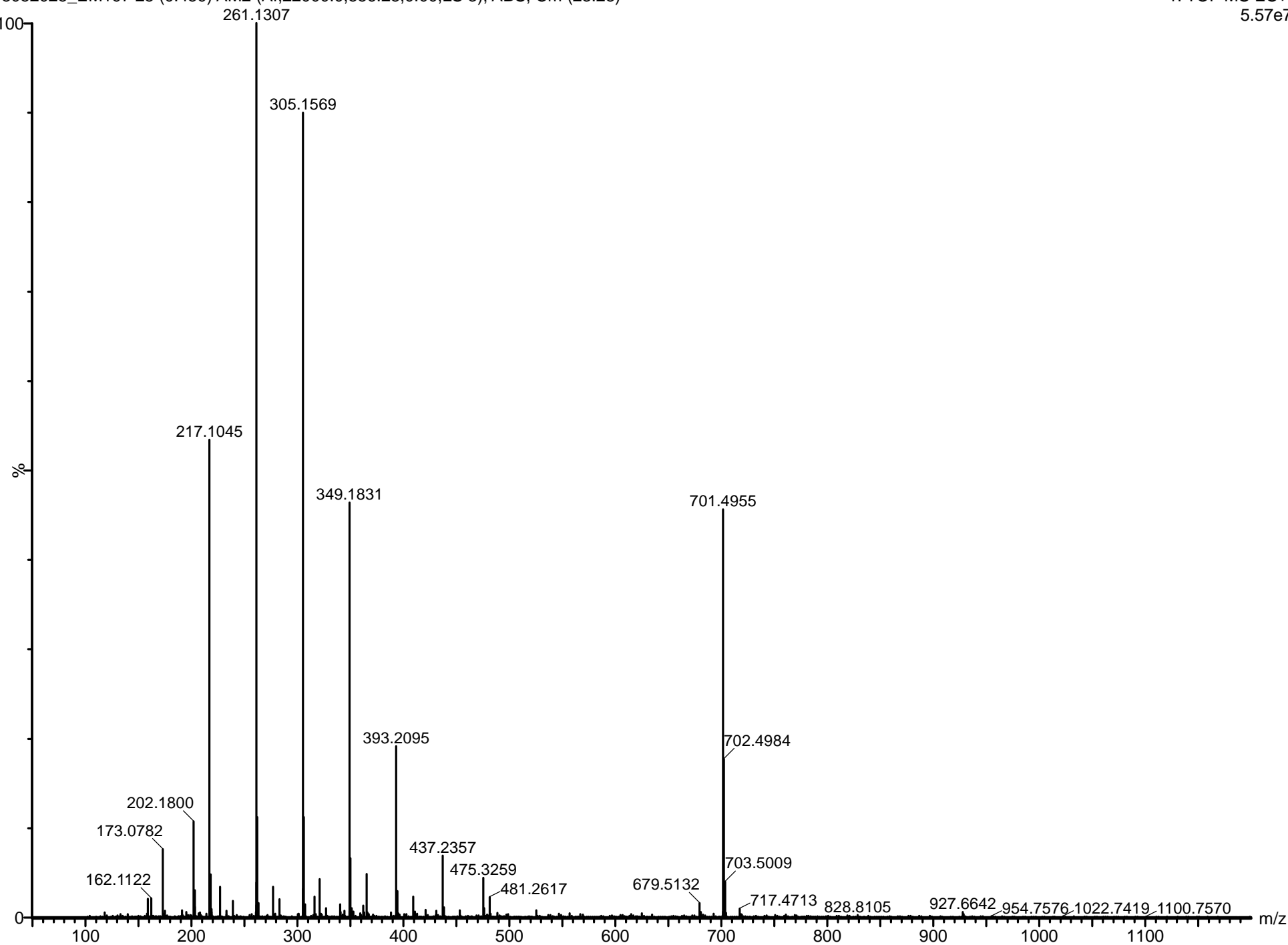

Supplement: S1 Data — Electrospray ionisation time of flight mass spectrometry (ESI-TOF MS, positive mode) spectra of the dengue cohort and ESI-TOF at different retention times. The spectra display the relative abundance (%) of detected ions across the m/z range. Prominent peaks corresponding to major ionised species are indicated. Variation in spectral profiles between retention times reflects the differences in compound composition and ionisation patterns within the sample. Data were acquired under identical instrumental conditions and are presented as representative scans. (ZIP) [file pntd.0014327.s003.zip › EM COMPLETE SAMPLES SPECTRUM/EM197 SPECTRUM RT 0.459.pdf]

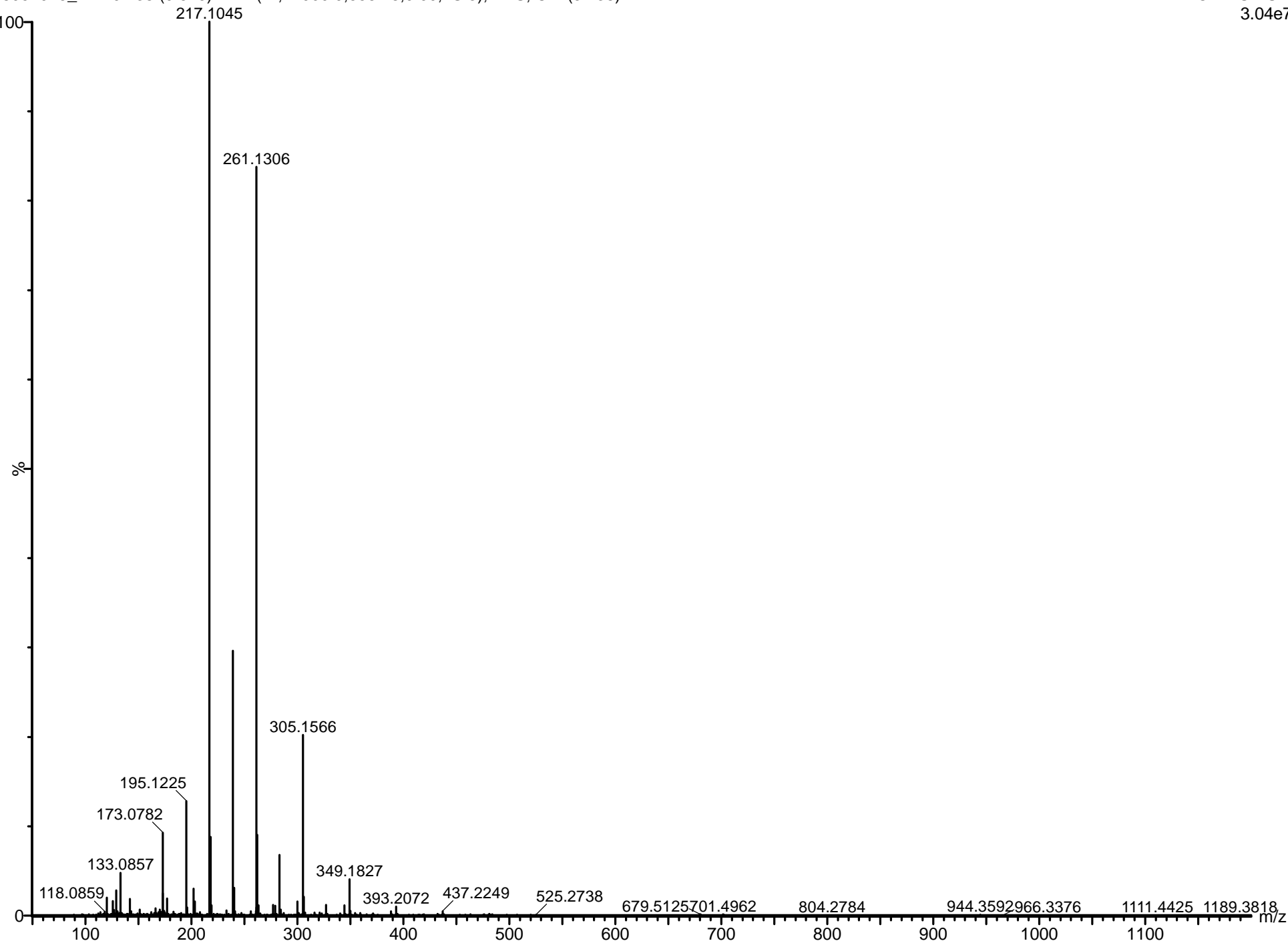

Supplement: S1 Data — Electrospray ionisation time of flight mass spectrometry (ESI-TOF MS, positive mode) spectra of the dengue cohort and ESI-TOF at different retention times. The spectra display the relative abundance (%) of detected ions across the m/z range. Prominent peaks corresponding to major ionised species are indicated. Variation in spectral profiles between retention times reflects the differences in compound composition and ionisation patterns within the sample. Data were acquired under identical instrumental conditions and are presented as representative scans. (ZIP) [file pntd.0014327.s003.zip › EM COMPLETE SAMPLES SPECTRUM/EM197 SPECTRUM RT 0.679.pdf]

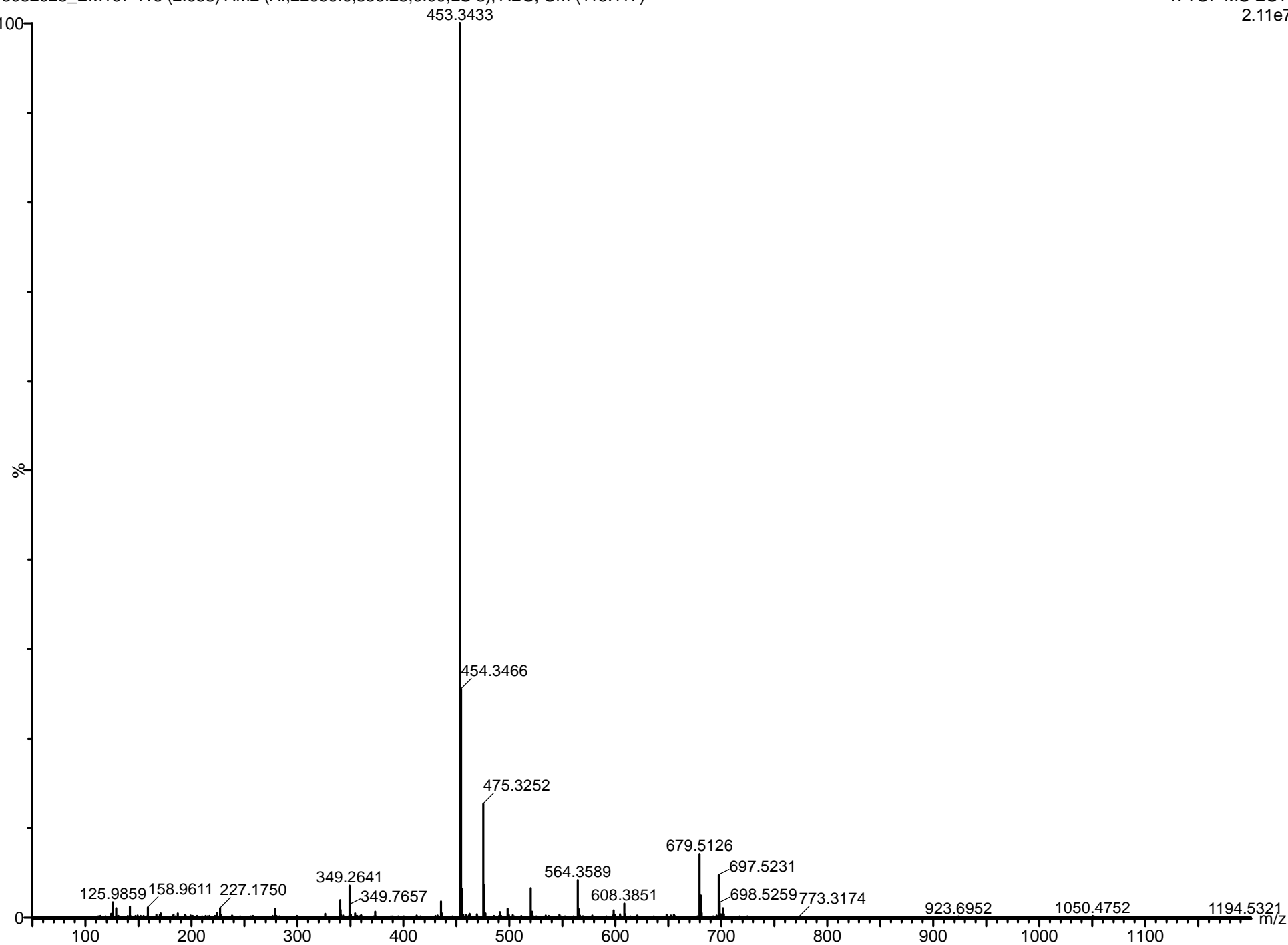

Supplement: S1 Data — Electrospray ionisation time of flight mass spectrometry (ESI-TOF MS, positive mode) spectra of the dengue cohort and ESI-TOF at different retention times. The spectra display the relative abundance (%) of detected ions across the m/z range. Prominent peaks corresponding to major ionised species are indicated. Variation in spectral profiles between retention times reflects the differences in compound composition and ionisation patterns within the sample. Data were acquired under identical instrumental conditions and are presented as representative scans. (ZIP) [file pntd.0014327.s003.zip › EM COMPLETE SAMPLES SPECTRUM/EM197 SPECTRUM RT 2.058.pdf]

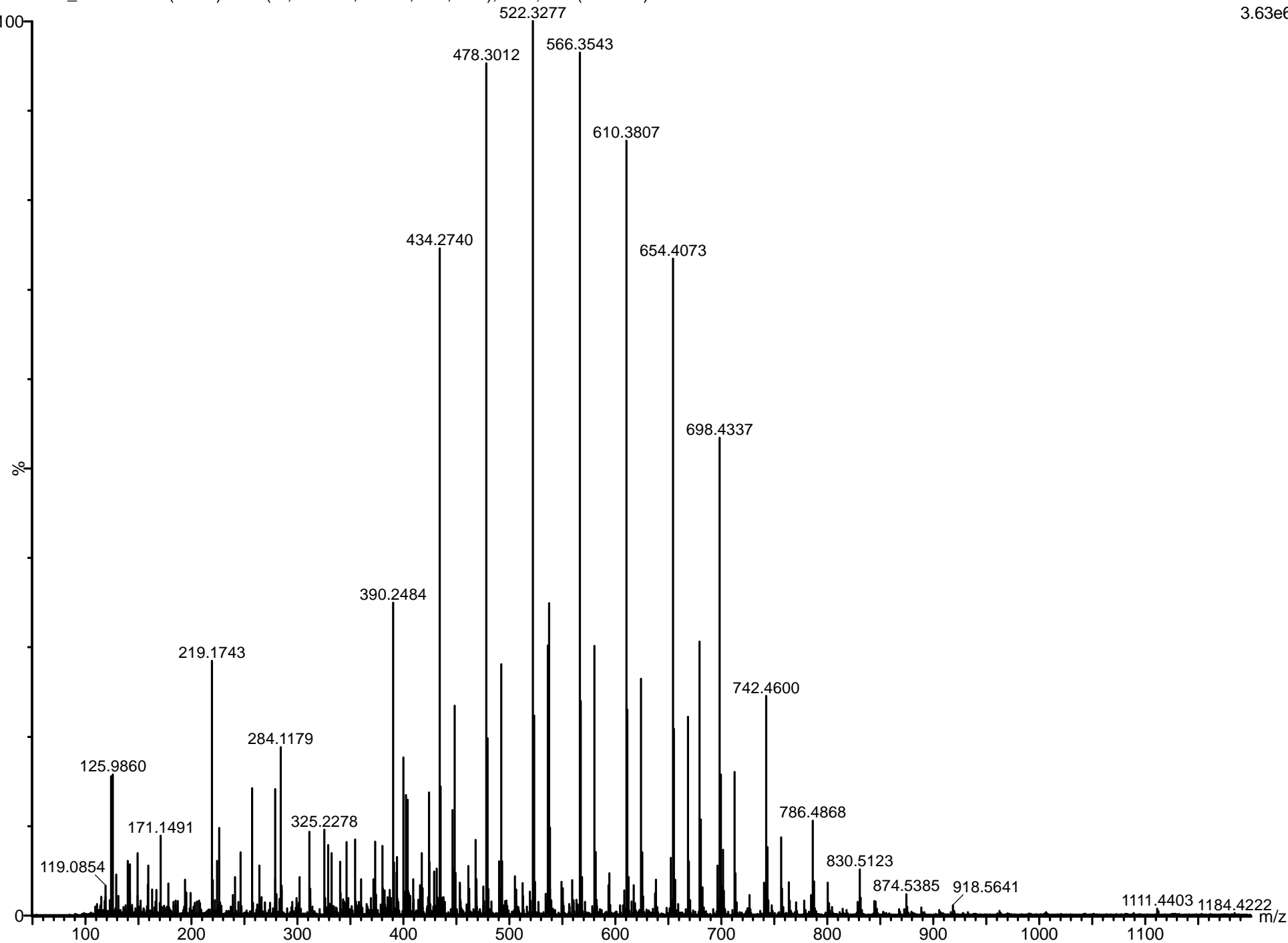

Supplement: S1 Data — Electrospray ionisation time of flight mass spectrometry (ESI-TOF MS, positive mode) spectra of the dengue cohort and ESI-TOF at different retention times. The spectra display the relative abundance (%) of detected ions across the m/z range. Prominent peaks corresponding to major ionised species are indicated. Variation in spectral profiles between retention times reflects the differences in compound composition and ionisation patterns within the sample. Data were acquired under identical instrumental conditions and are presented as representative scans. (ZIP) [file pntd.0014327.s003.zip › EM COMPLETE SAMPLES SPECTRUM/EM197 SPECTRUM RT 2.565.pdf]

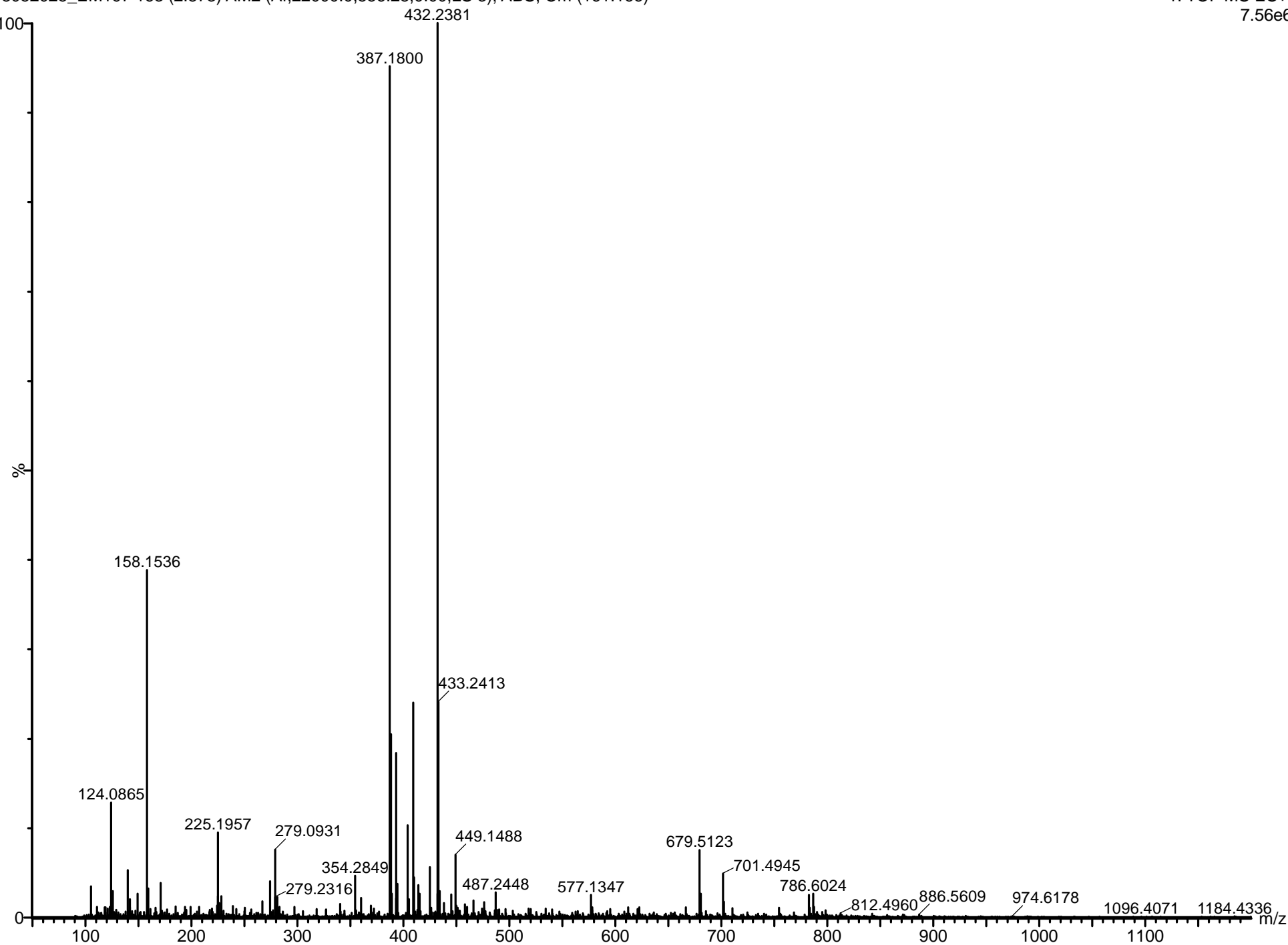

Supplement: S1 Data — Electrospray ionisation time of flight mass spectrometry (ESI-TOF MS, positive mode) spectra of the dengue cohort and ESI-TOF at different retention times. The spectra display the relative abundance (%) of detected ions across the m/z range. Prominent peaks corresponding to major ionised species are indicated. Variation in spectral profiles between retention times reflects the differences in compound composition and ionisation patterns within the sample. Data were acquired under identical instrumental conditions and are presented as representative scans. (ZIP) [file pntd.0014327.s003.zip › EM COMPLETE SAMPLES SPECTRUM/EM197 SPECTRUM RT 2.873.pdf]

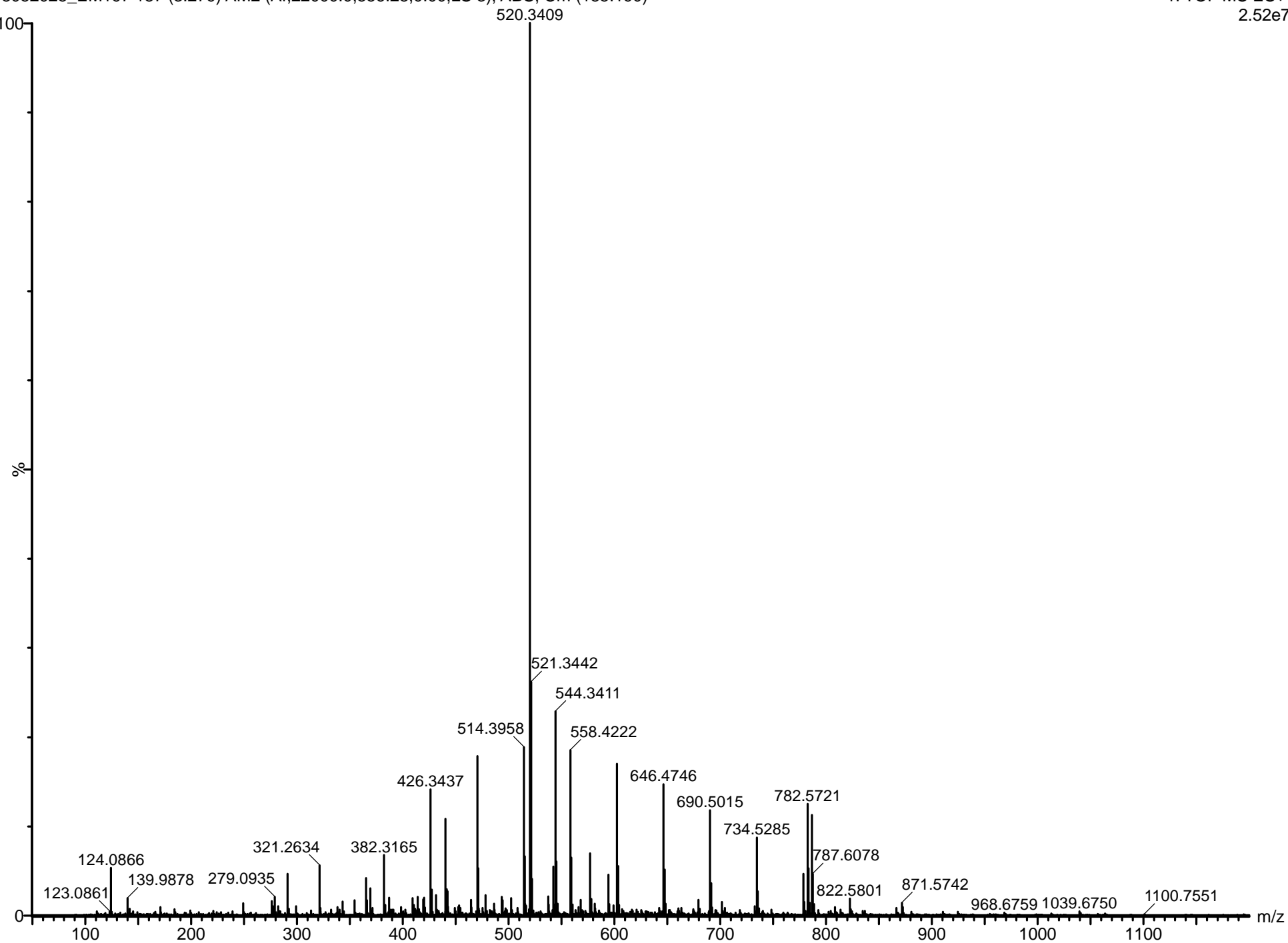

Supplement: S1 Data — Electrospray ionisation time of flight mass spectrometry (ESI-TOF MS, positive mode) spectra of the dengue cohort and ESI-TOF at different retention times. The spectra display the relative abundance (%) of detected ions across the m/z range. Prominent peaks corresponding to major ionised species are indicated. Variation in spectral profiles between retention times reflects the differences in compound composition and ionisation patterns within the sample. Data were acquired under identical instrumental conditions and are presented as representative scans. (ZIP) [file pntd.0014327.s003.zip › EM COMPLETE SAMPLES SPECTRUM/EM197 SPECTRUM RT 3.279.pdf]

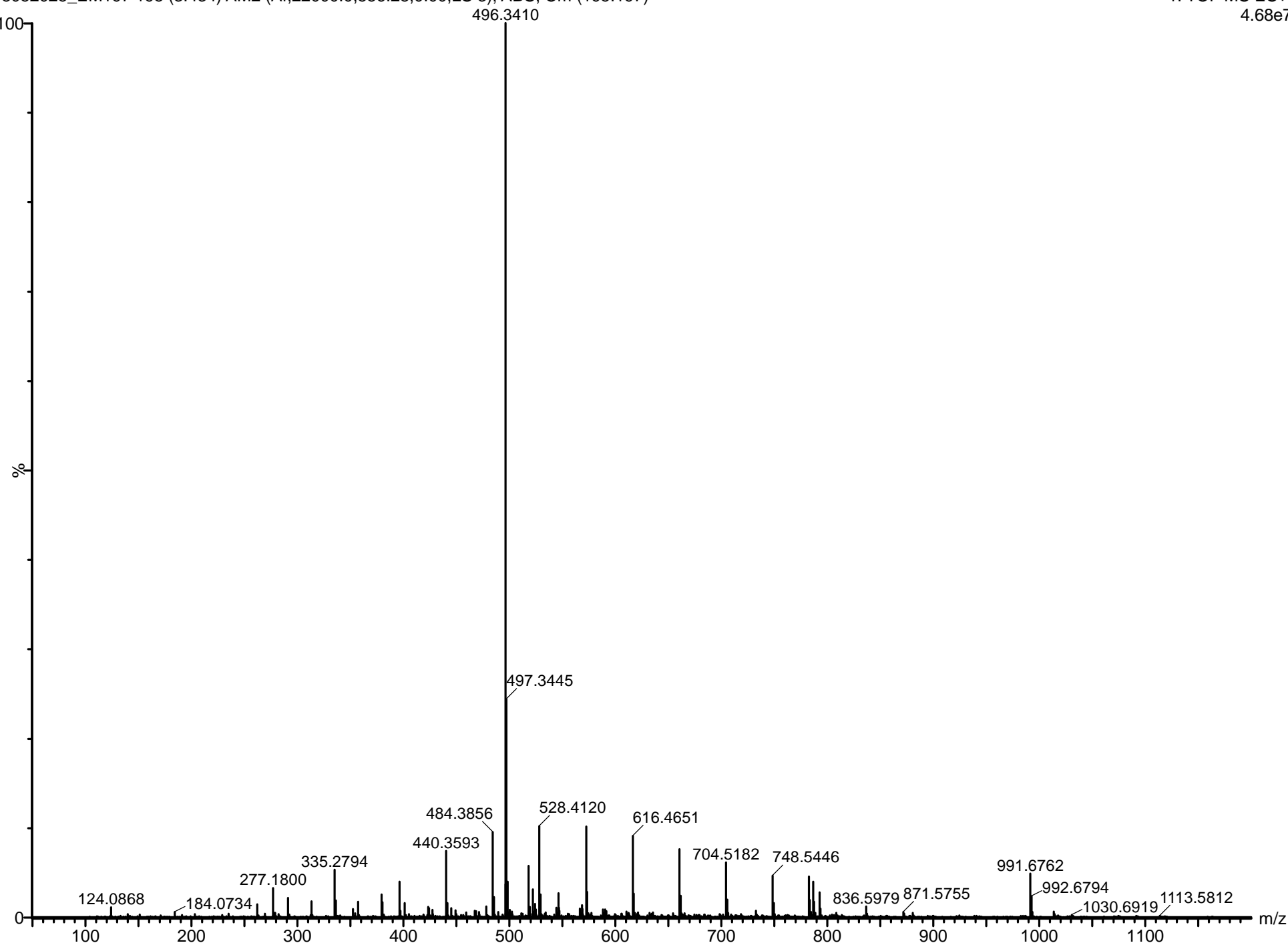

Supplement: S1 Data — Electrospray ionisation time of flight mass spectrometry (ESI-TOF MS, positive mode) spectra of the dengue cohort and ESI-TOF at different retention times. The spectra display the relative abundance (%) of detected ions across the m/z range. Prominent peaks corresponding to major ionised species are indicated. Variation in spectral profiles between retention times reflects the differences in compound composition and ionisation patterns within the sample. Data were acquired under identical instrumental conditions and are presented as representative scans. (ZIP) [file pntd.0014327.s003.zip › EM COMPLETE SAMPLES SPECTRUM/EM197 SPECTRUM RT 3.434.pdf]

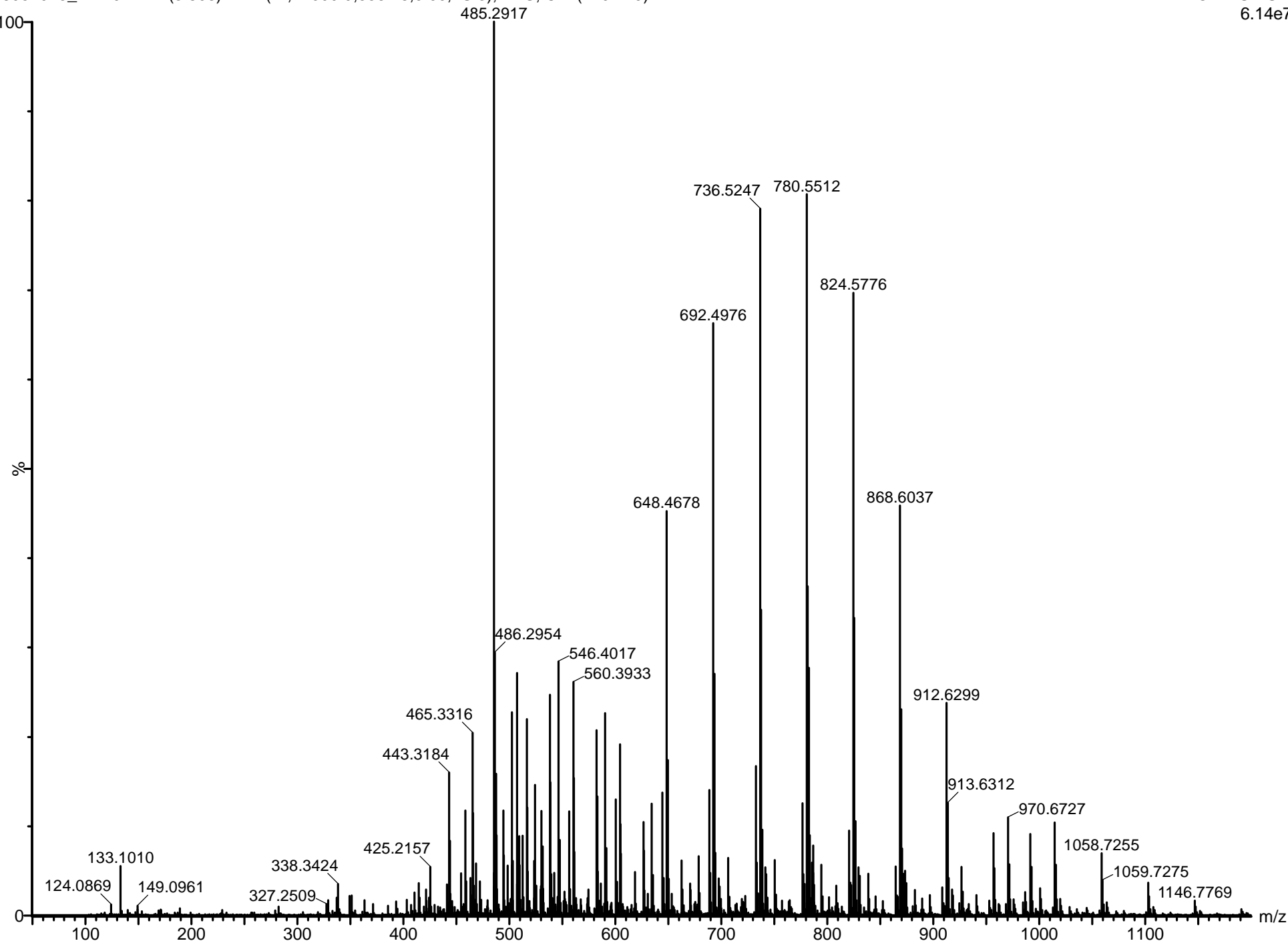

Supplement: S1 Data — Electrospray ionisation time of flight mass spectrometry (ESI-TOF MS, positive mode) spectra of the dengue cohort and ESI-TOF at different retention times. The spectra display the relative abundance (%) of detected ions across the m/z range. Prominent peaks corresponding to major ionised species are indicated. Variation in spectral profiles between retention times reflects the differences in compound composition and ionisation patterns within the sample. Data were acquired under identical instrumental conditions and are presented as representative scans. (ZIP) [file pntd.0014327.s003.zip › EM COMPLETE SAMPLES SPECTRUM/EM197 SPECTRUM RT 3.806.pdf]

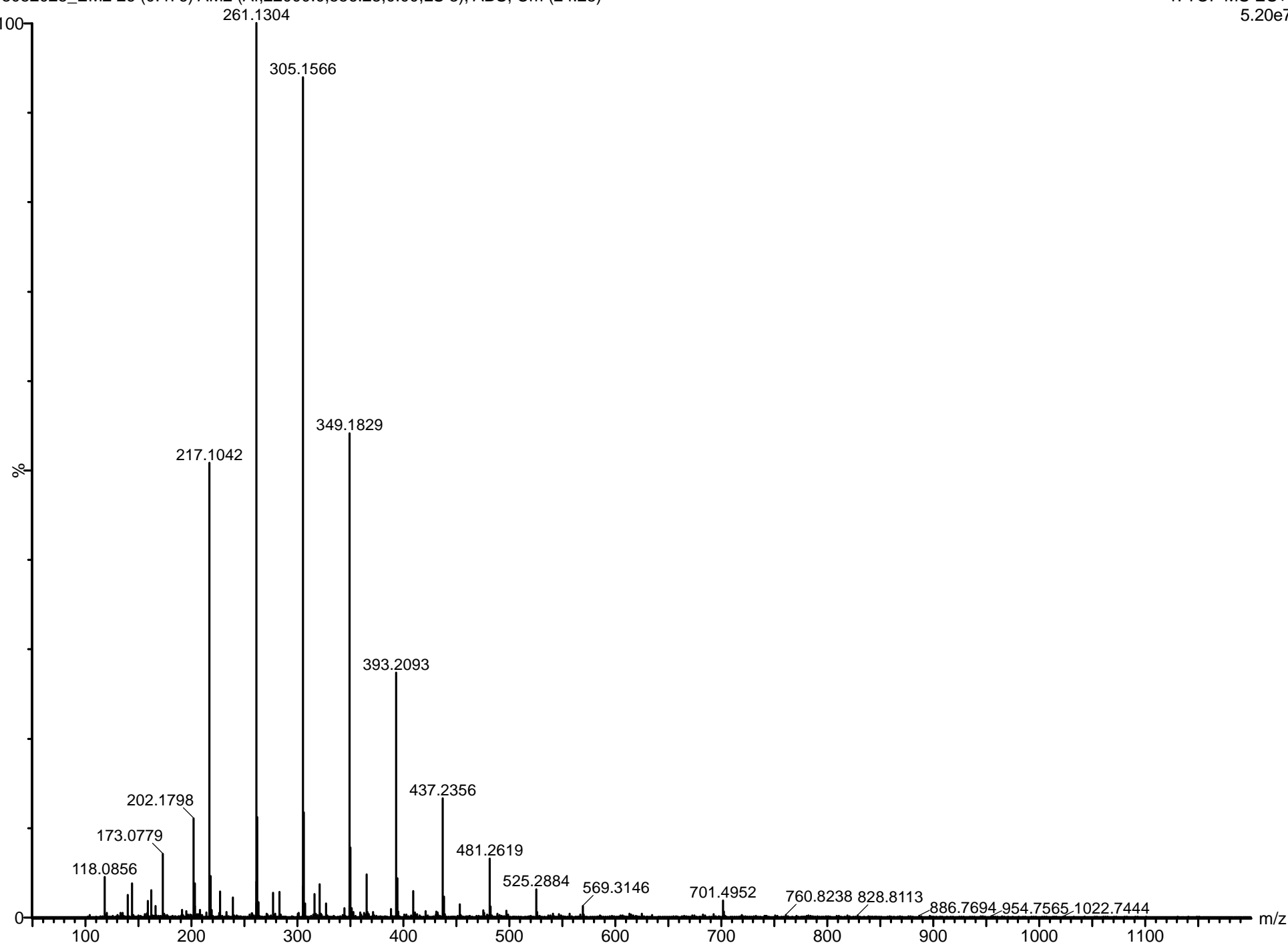

Supplement: S1 Data — Electrospray ionisation time of flight mass spectrometry (ESI-TOF MS, positive mode) spectra of the dengue cohort and ESI-TOF at different retention times. The spectra display the relative abundance (%) of detected ions across the m/z range. Prominent peaks corresponding to major ionised species are indicated. Variation in spectral profiles between retention times reflects the differences in compound composition and ionisation patterns within the sample. Data were acquired under identical instrumental conditions and are presented as representative scans. (ZIP) [file pntd.0014327.s003.zip › EM COMPLETE SAMPLES SPECTRUM/EM2 SPECTRUM RT 0.476.pdf]

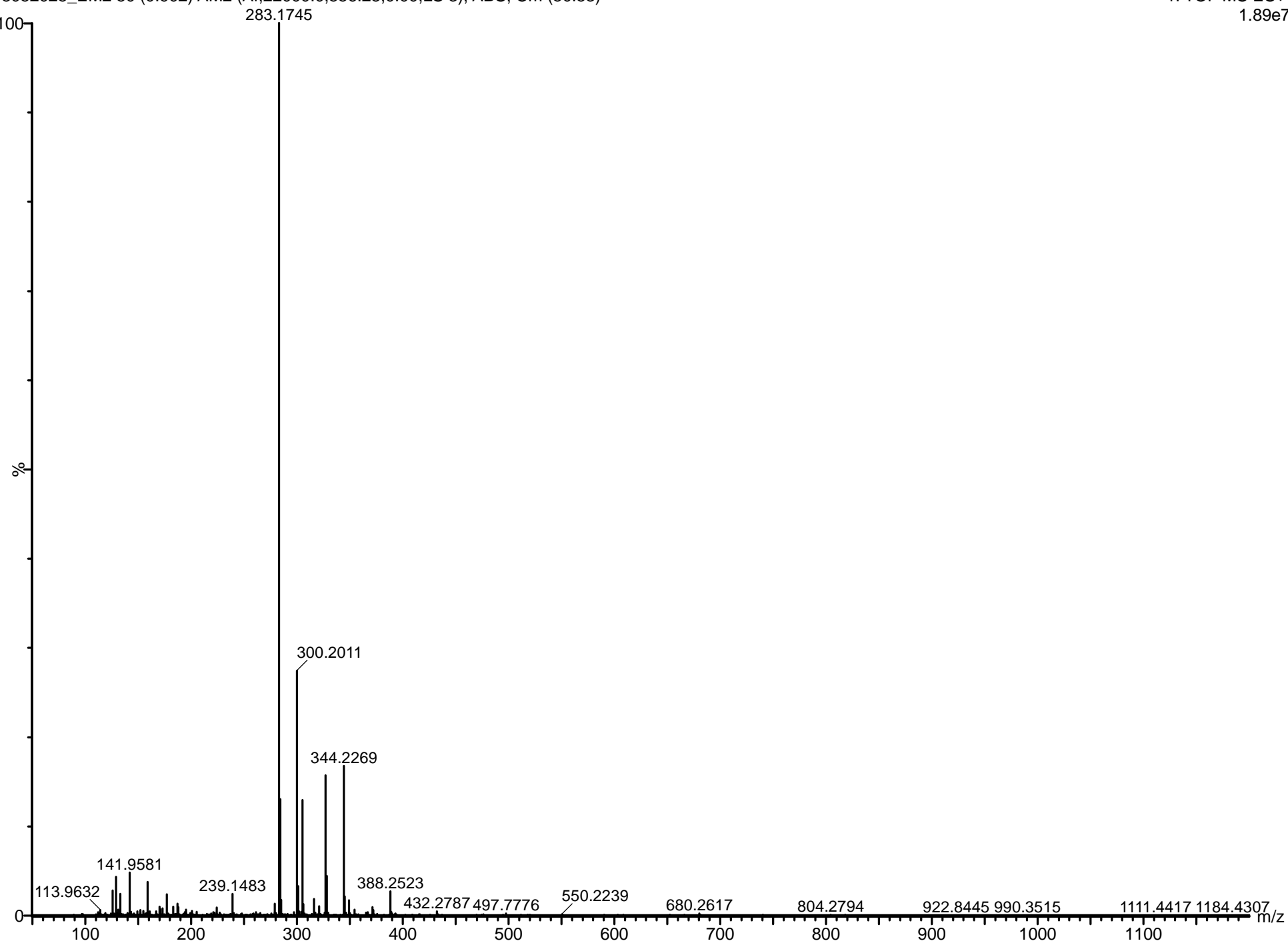

Supplement: S1 Data — Electrospray ionisation time of flight mass spectrometry (ESI-TOF MS, positive mode) spectra of the dengue cohort and ESI-TOF at different retention times. The spectra display the relative abundance (%) of detected ions across the m/z range. Prominent peaks corresponding to major ionised species are indicated. Variation in spectral profiles between retention times reflects the differences in compound composition and ionisation patterns within the sample. Data were acquired under identical instrumental conditions and are presented as representative scans. (ZIP) [file pntd.0014327.s003.zip › EM COMPLETE SAMPLES SPECTRUM/EM2 SPECTRUM RT 0.902.pdf]

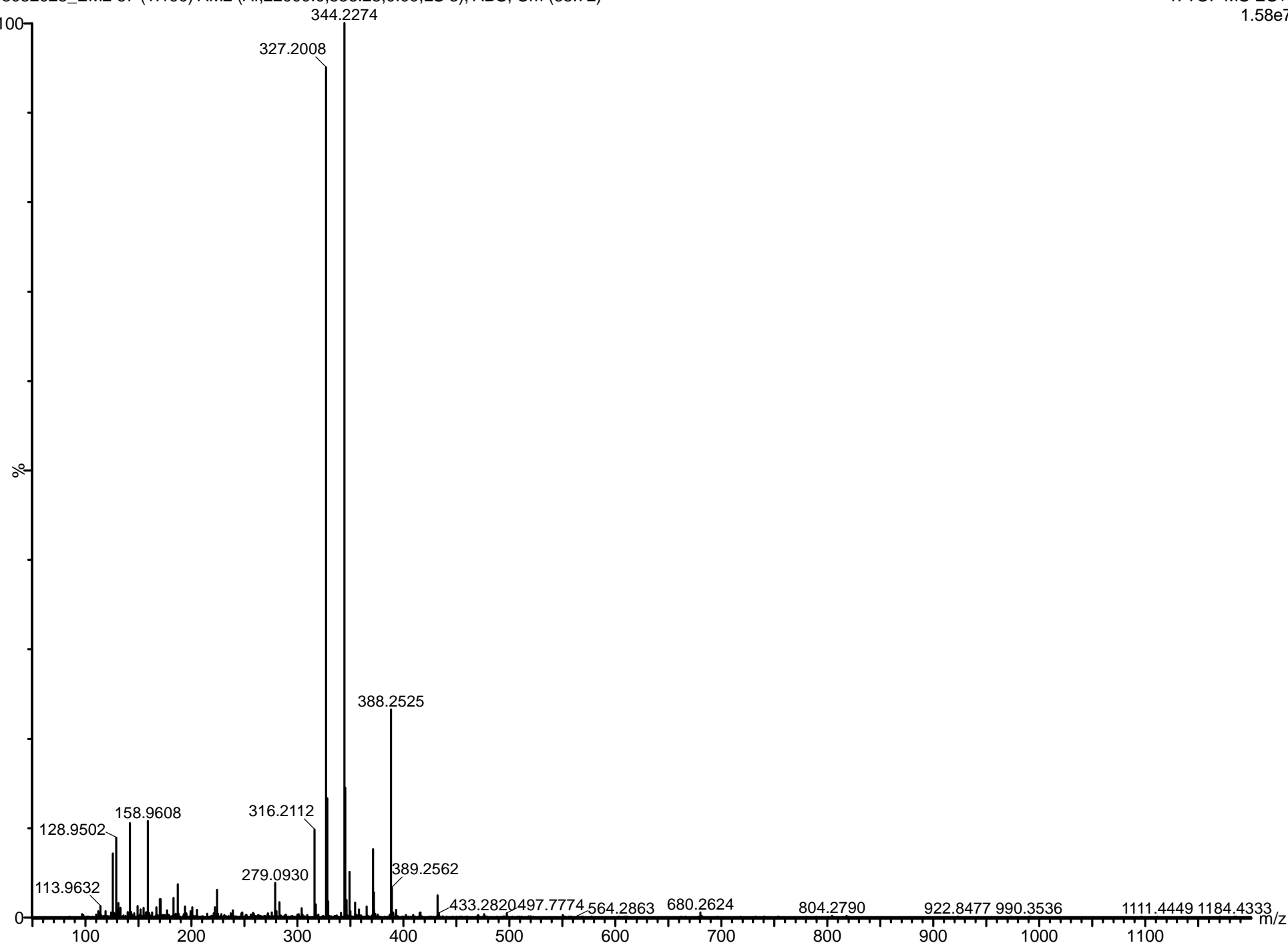

Supplement: S1 Data — Electrospray ionisation time of flight mass spectrometry (ESI-TOF MS, positive mode) spectra of the dengue cohort and ESI-TOF at different retention times. The spectra display the relative abundance (%) of detected ions across the m/z range. Prominent peaks corresponding to major ionised species are indicated. Variation in spectral profiles between retention times reflects the differences in compound composition and ionisation patterns within the sample. Data were acquired under identical instrumental conditions and are presented as representative scans. (ZIP) [file pntd.0014327.s003.zip › EM COMPLETE SAMPLES SPECTRUM/EM2 SPECTRUM RT 1.190.pdf]

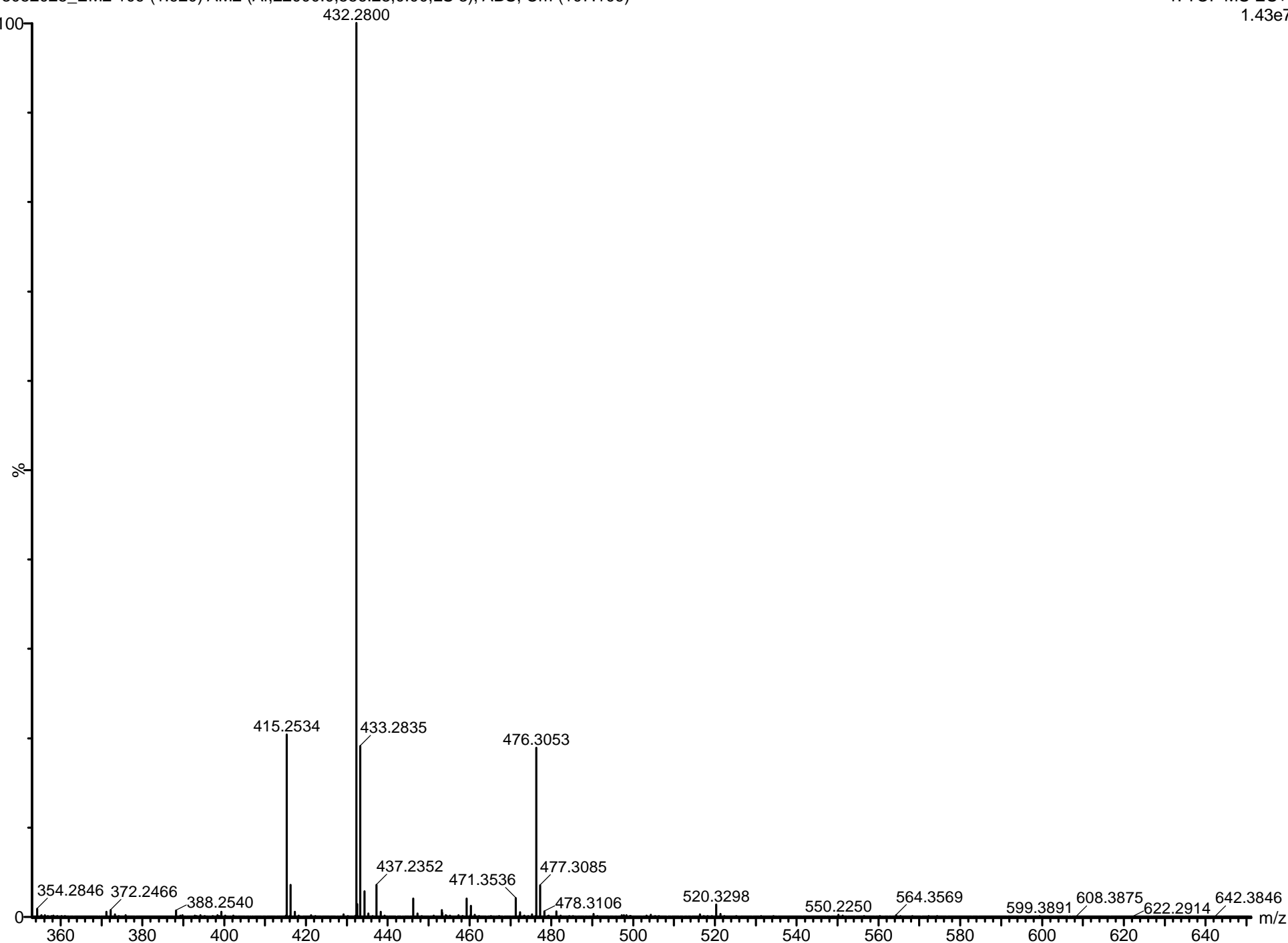

Supplement: S1 Data — Electrospray ionisation time of flight mass spectrometry (ESI-TOF MS, positive mode) spectra of the dengue cohort and ESI-TOF at different retention times. The spectra display the relative abundance (%) of detected ions across the m/z range. Prominent peaks corresponding to major ionised species are indicated. Variation in spectral profiles between retention times reflects the differences in compound composition and ionisation patterns within the sample. Data were acquired under identical instrumental conditions and are presented as representative scans. (ZIP) [file pntd.0014327.s003.zip › EM COMPLETE SAMPLES SPECTRUM/EM2 SPECTRUM RT 1.920 EX.pdf]

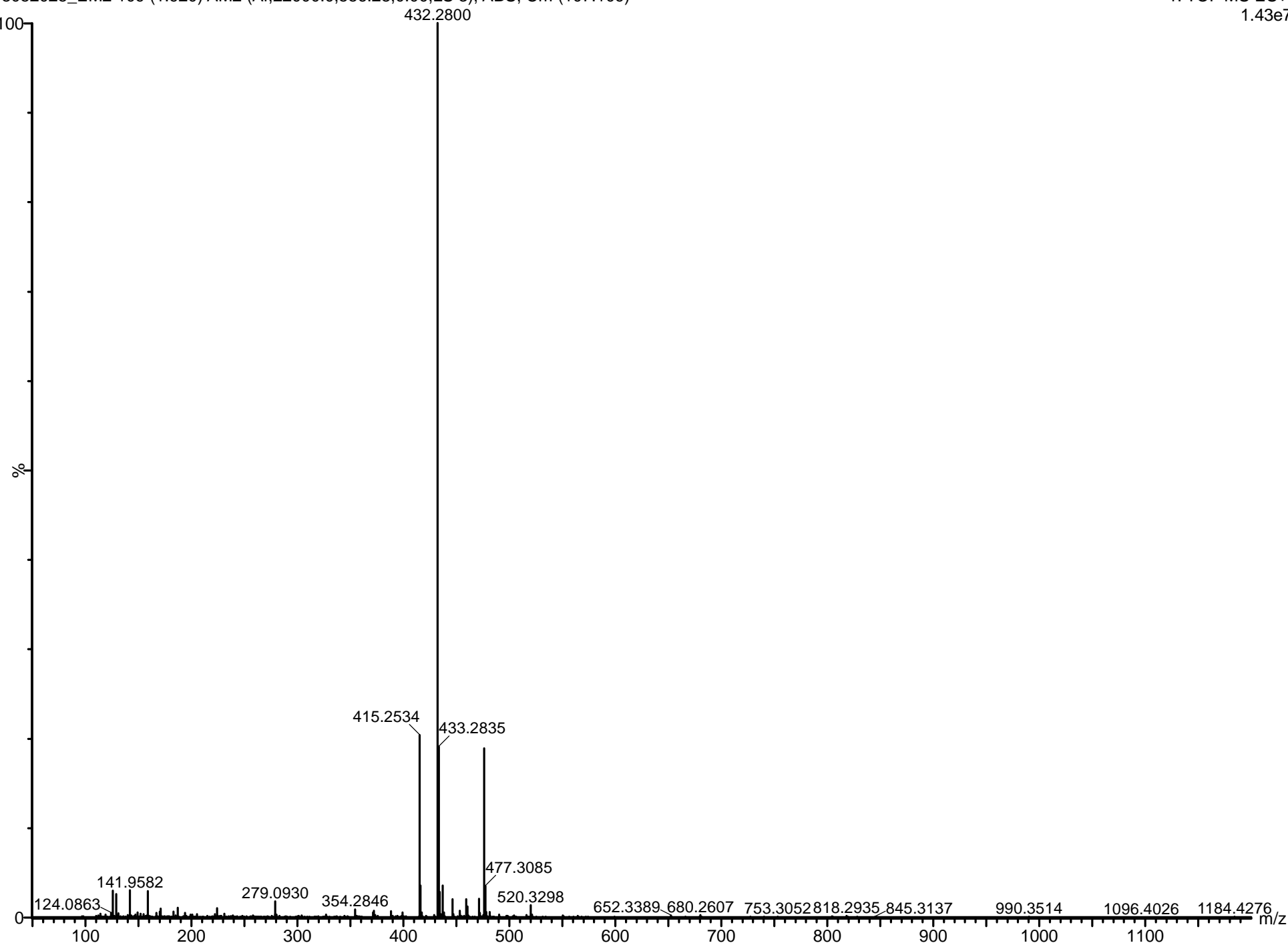

Supplement: S1 Data — Electrospray ionisation time of flight mass spectrometry (ESI-TOF MS, positive mode) spectra of the dengue cohort and ESI-TOF at different retention times. The spectra display the relative abundance (%) of detected ions across the m/z range. Prominent peaks corresponding to major ionised species are indicated. Variation in spectral profiles between retention times reflects the differences in compound composition and ionisation patterns within the sample. Data were acquired under identical instrumental conditions and are presented as representative scans. (ZIP) [file pntd.0014327.s003.zip › EM COMPLETE SAMPLES SPECTRUM/EM2 SPECTRUM RT 1.920.pdf]

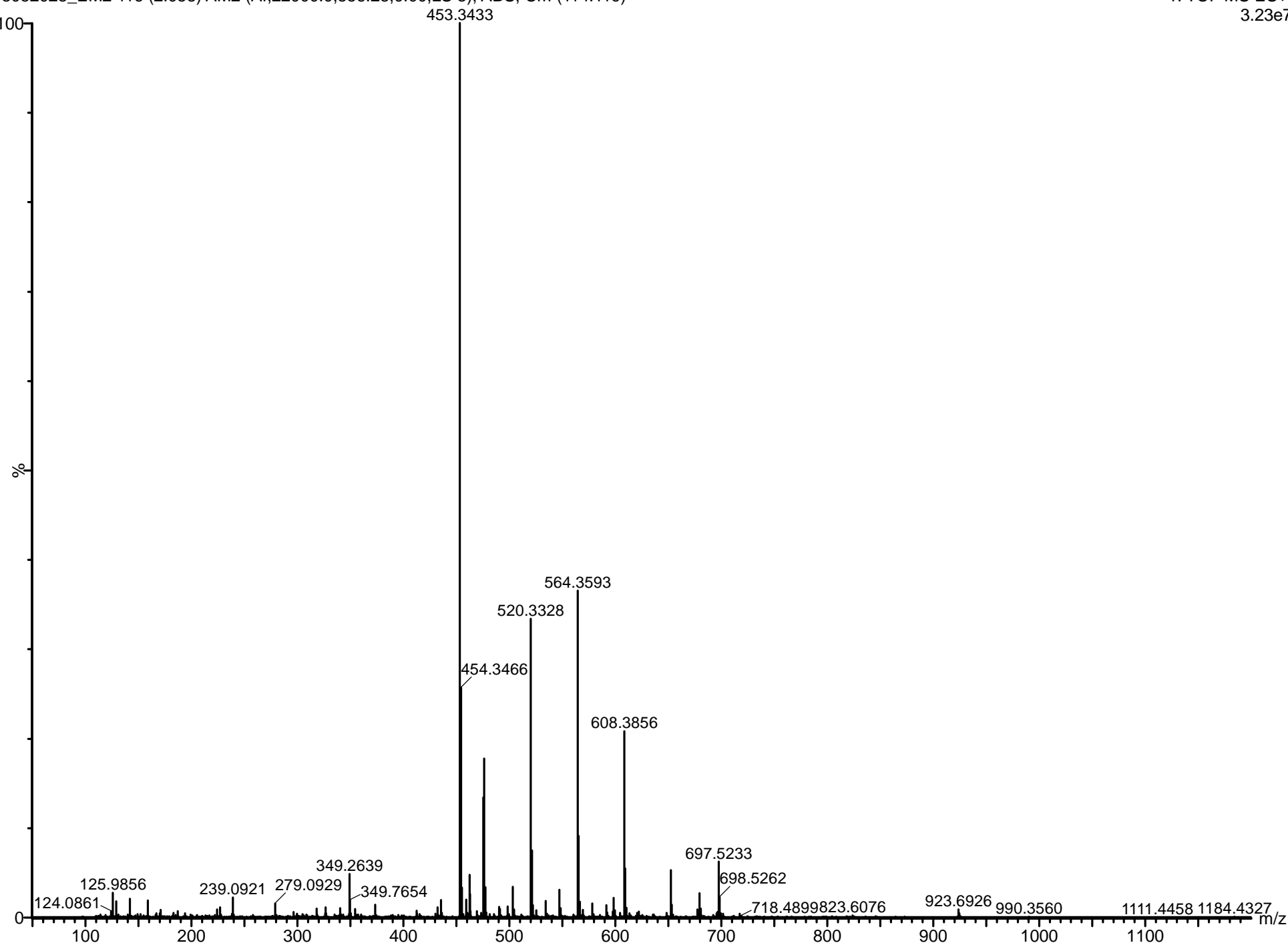

Supplement: S1 Data — Electrospray ionisation time of flight mass spectrometry (ESI-TOF MS, positive mode) spectra of the dengue cohort and ESI-TOF at different retention times. The spectra display the relative abundance (%) of detected ions across the m/z range. Prominent peaks corresponding to major ionised species are indicated. Variation in spectral profiles between retention times reflects the differences in compound composition and ionisation patterns within the sample. Data were acquired under identical instrumental conditions and are presented as representative scans. (ZIP) [file pntd.0014327.s003.zip › EM COMPLETE SAMPLES SPECTRUM/EM2 SPECTRUM RT 2.058.pdf]

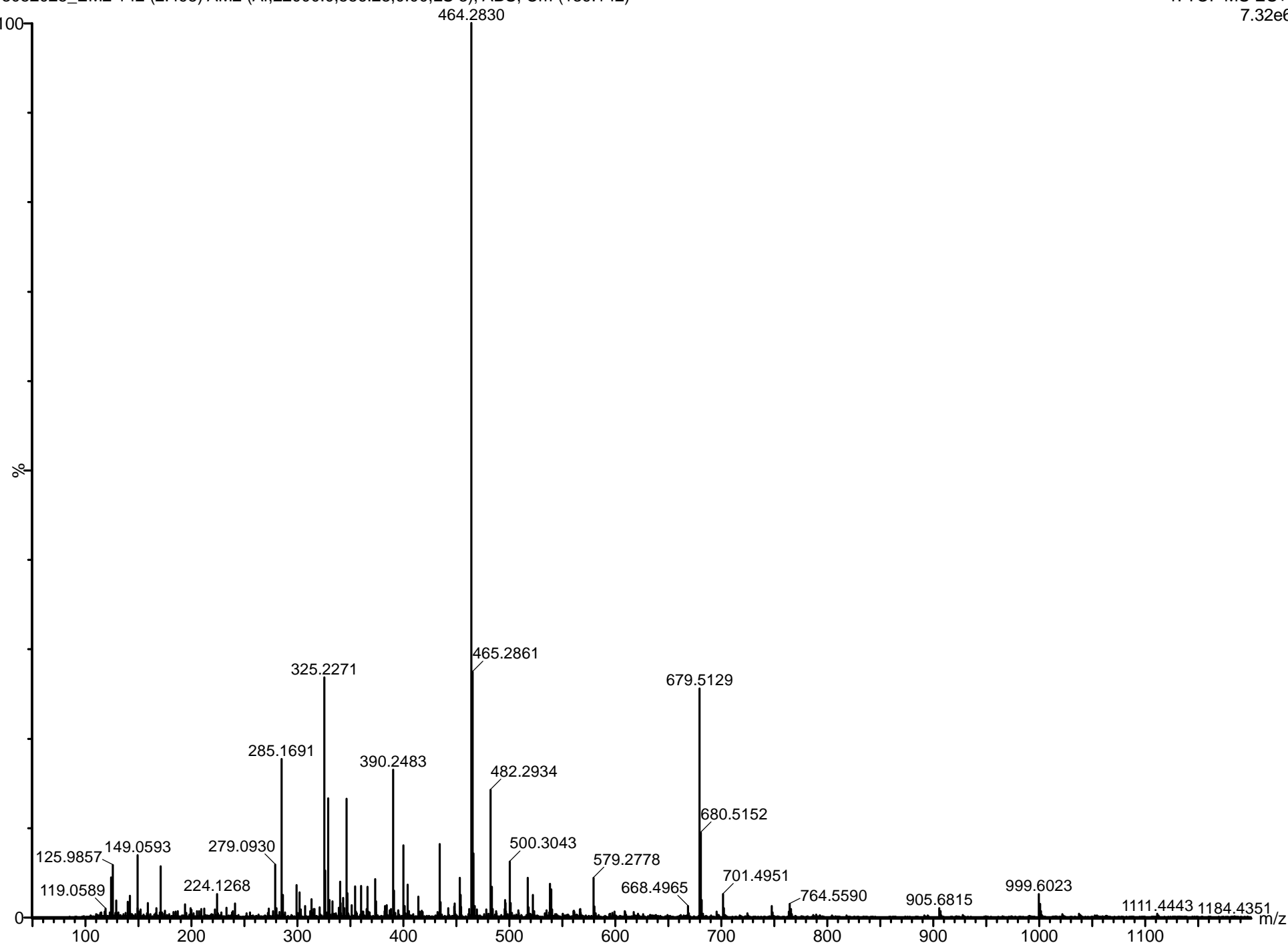

Supplement: S1 Data — Electrospray ionisation time of flight mass spectrometry (ESI-TOF MS, positive mode) spectra of the dengue cohort and ESI-TOF at different retention times. The spectra display the relative abundance (%) of detected ions across the m/z range. Prominent peaks corresponding to major ionised species are indicated. Variation in spectral profiles between retention times reflects the differences in compound composition and ionisation patterns within the sample. Data were acquired under identical instrumental conditions and are presented as representative scans. (ZIP) [file pntd.0014327.s003.zip › EM COMPLETE SAMPLES SPECTRUM/EM2 SPECTRUM RT 2.498.pdf]

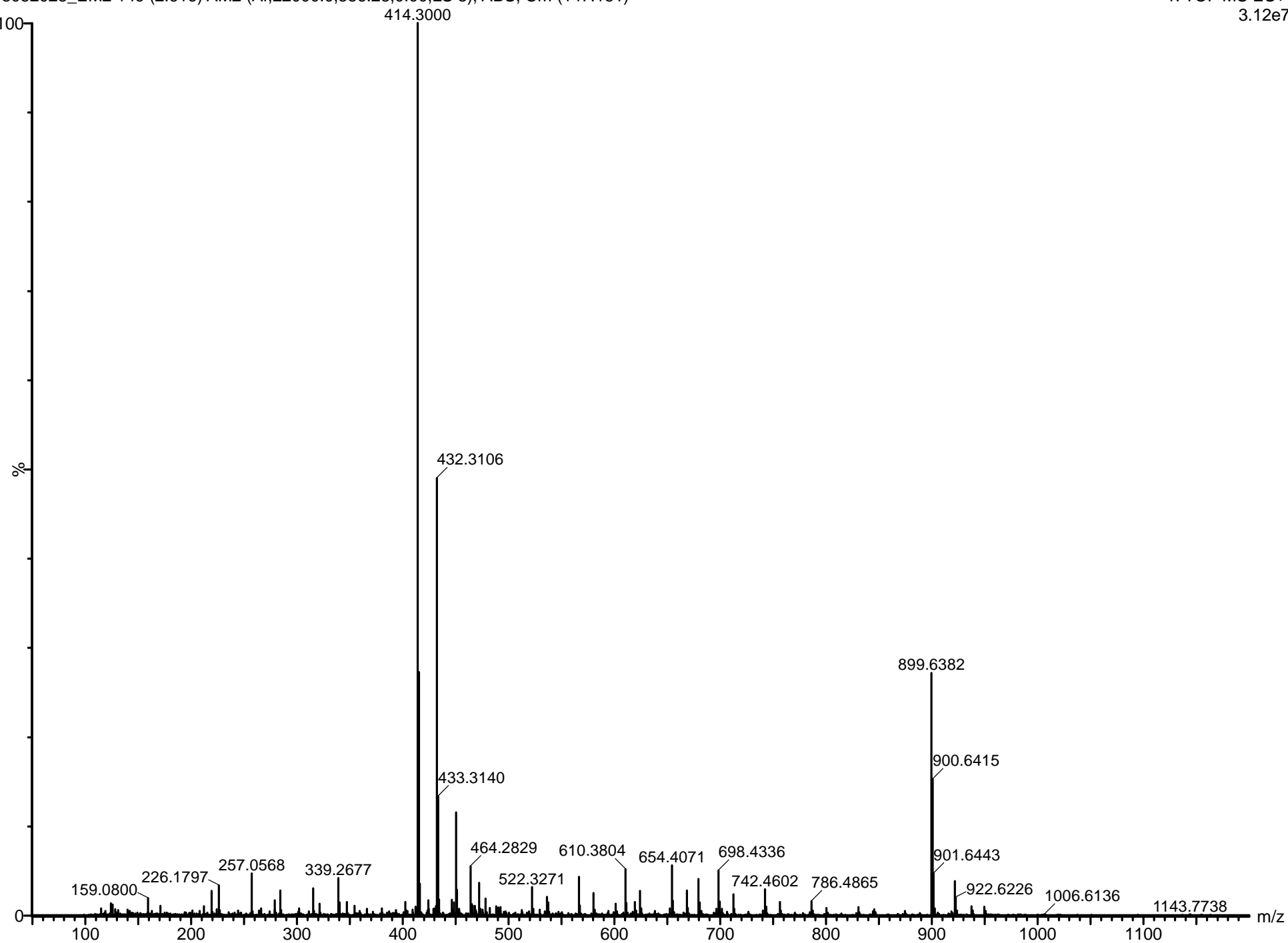

Supplement: S1 Data — Electrospray ionisation time of flight mass spectrometry (ESI-TOF MS, positive mode) spectra of the dengue cohort and ESI-TOF at different retention times. The spectra display the relative abundance (%) of detected ions across the m/z range. Prominent peaks corresponding to major ionised species are indicated. Variation in spectral profiles between retention times reflects the differences in compound composition and ionisation patterns within the sample. Data were acquired under identical instrumental conditions and are presented as representative scans. (ZIP) [file pntd.0014327.s003.zip › EM COMPLETE SAMPLES SPECTRUM/EM2 SPECTRUM RT 2.616.pdf]

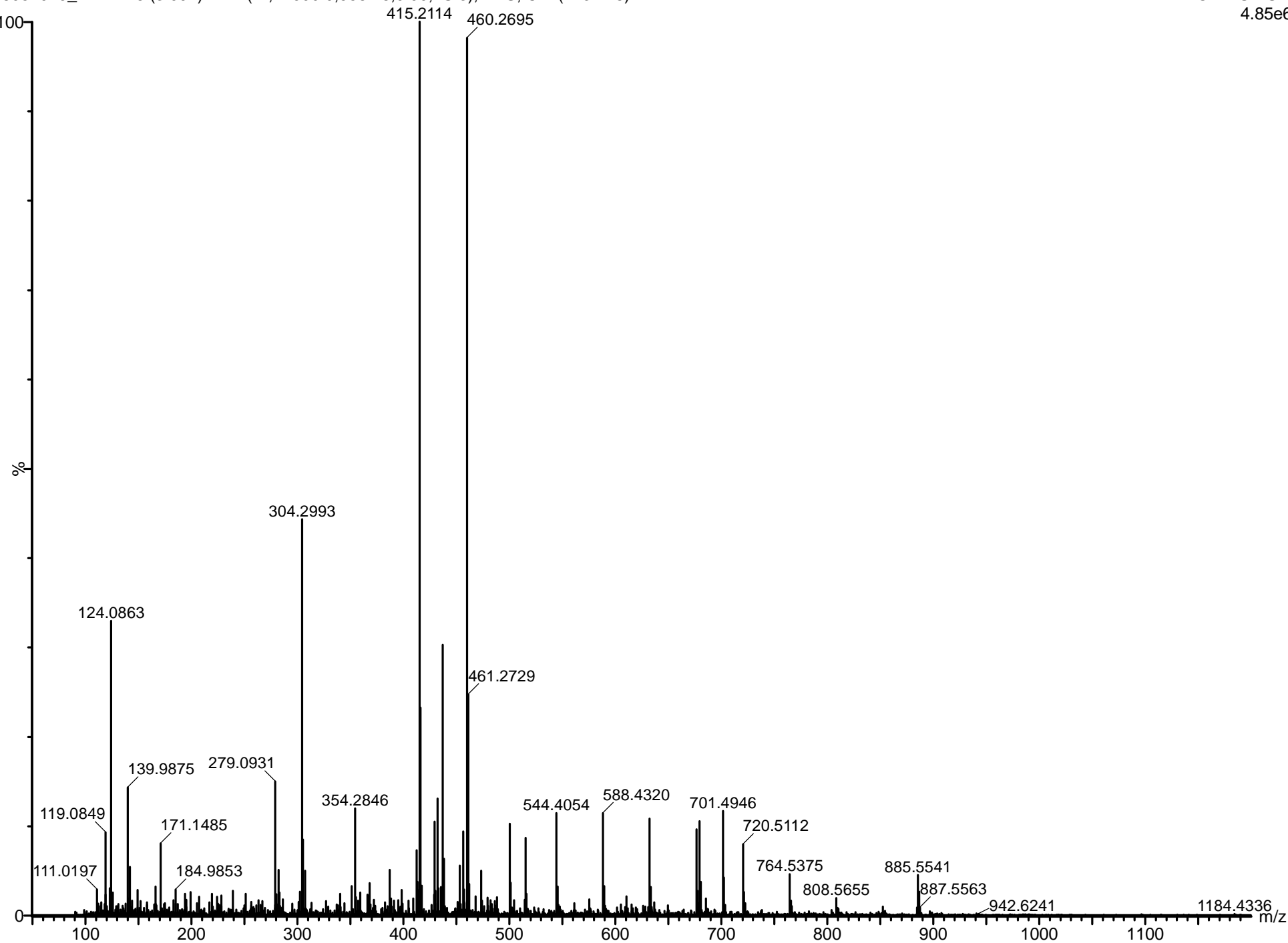

Supplement: S1 Data — Electrospray ionisation time of flight mass spectrometry (ESI-TOF MS, positive mode) spectra of the dengue cohort and ESI-TOF at different retention times. The spectra display the relative abundance (%) of detected ions across the m/z range. Prominent peaks corresponding to major ionised species are indicated. Variation in spectral profiles between retention times reflects the differences in compound composition and ionisation patterns within the sample. Data were acquired under identical instrumental conditions and are presented as representative scans. (ZIP) [file pntd.0014327.s003.zip › EM COMPLETE SAMPLES SPECTRUM/EM2 SPECTRUM RT 3.092.pdf]

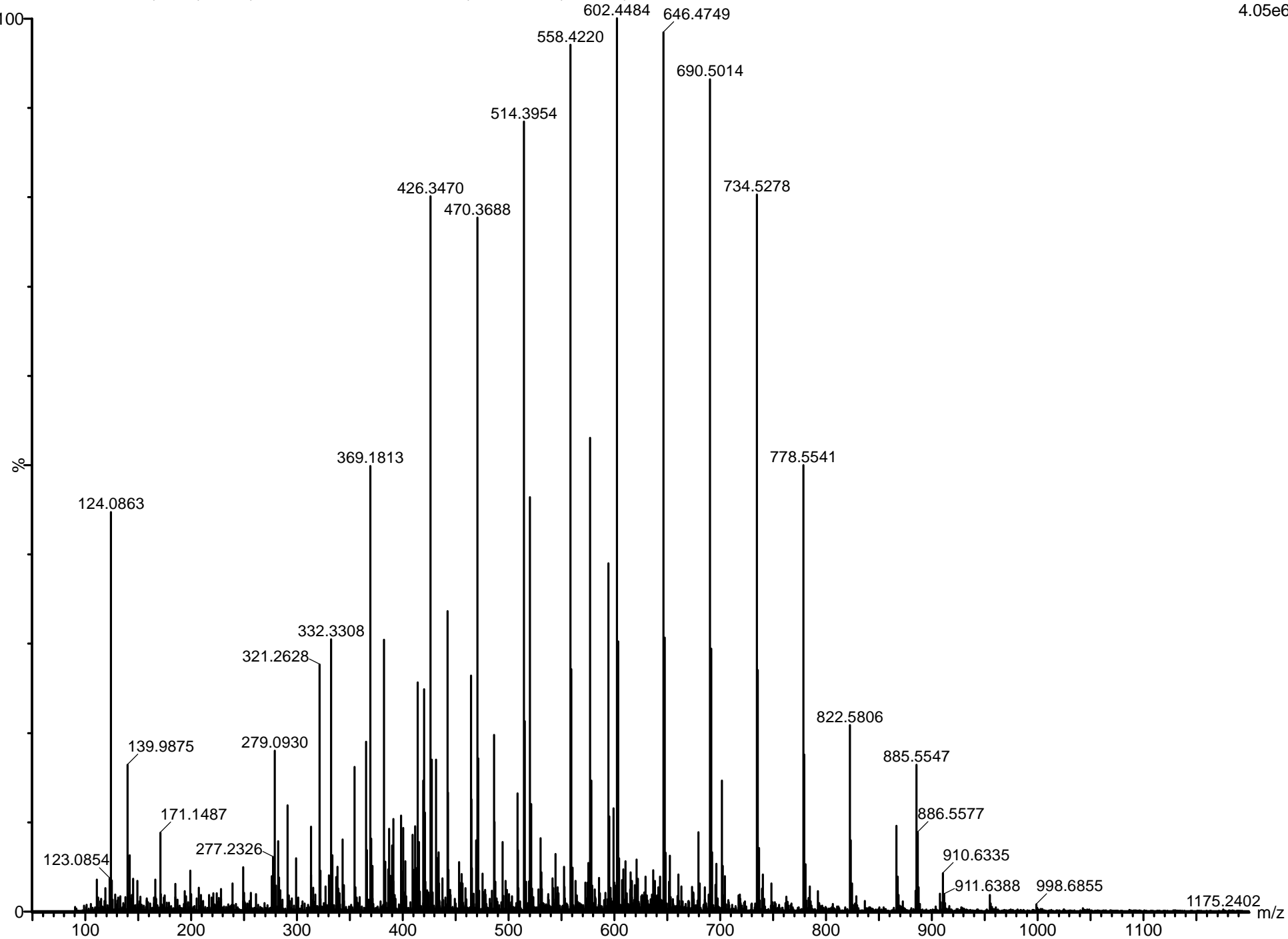

Supplement: S1 Data — Electrospray ionisation time of flight mass spectrometry (ESI-TOF MS, positive mode) spectra of the dengue cohort and ESI-TOF at different retention times. The spectra display the relative abundance (%) of detected ions across the m/z range. Prominent peaks corresponding to major ionised species are indicated. Variation in spectral profiles between retention times reflects the differences in compound composition and ionisation patterns within the sample. Data were acquired under identical instrumental conditions and are presented as representative scans. (ZIP) [file pntd.0014327.s003.zip › EM COMPLETE SAMPLES SPECTRUM/EM2 SPECTRUM RT 3.279.pdf]

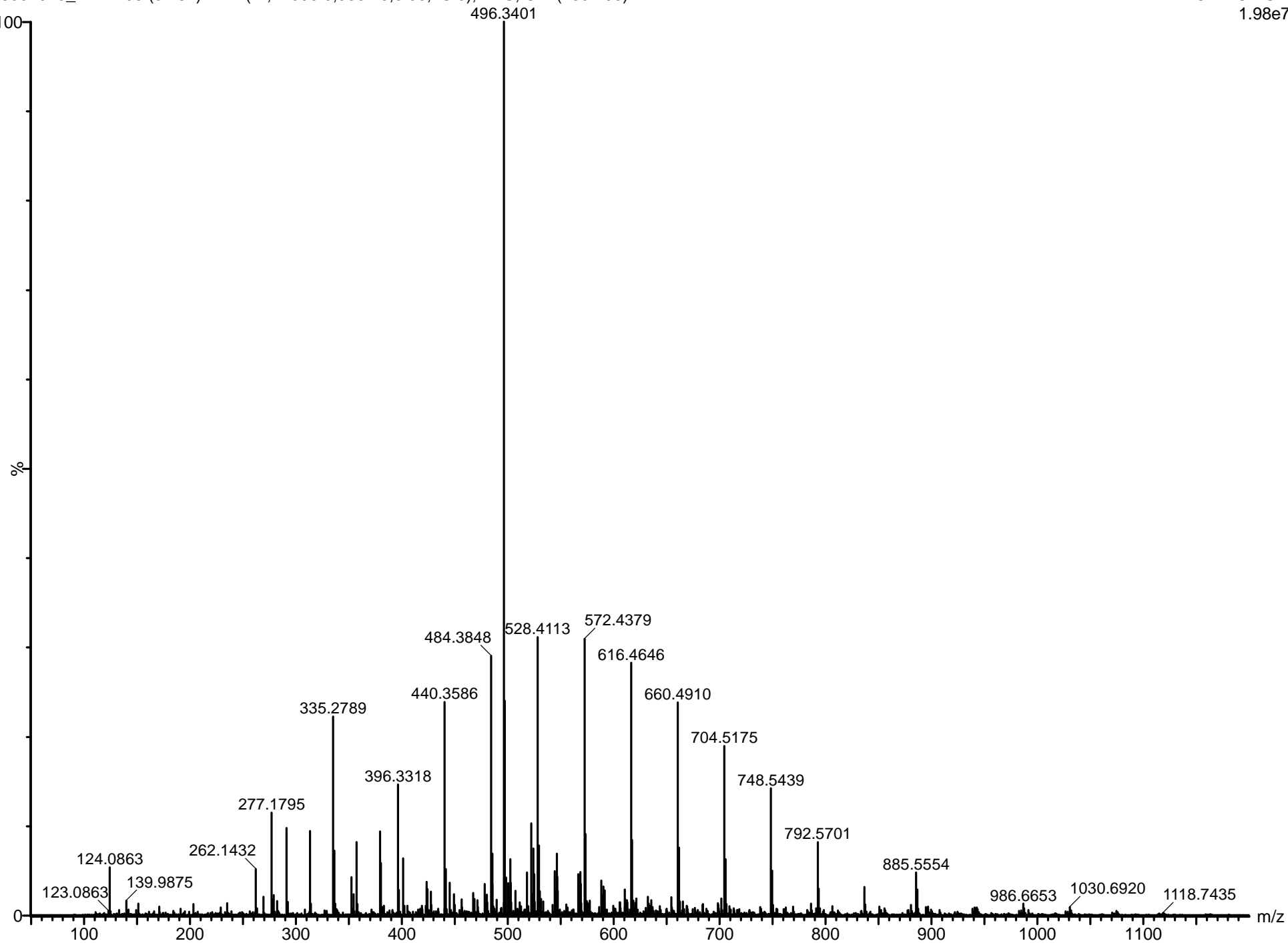

Supplement: S1 Data — Electrospray ionisation time of flight mass spectrometry (ESI-TOF MS, positive mode) spectra of the dengue cohort and ESI-TOF at different retention times. The spectra display the relative abundance (%) of detected ions across the m/z range. Prominent peaks corresponding to major ionised species are indicated. Variation in spectral profiles between retention times reflects the differences in compound composition and ionisation patterns within the sample. Data were acquired under identical instrumental conditions and are presented as representative scans. (ZIP) [file pntd.0014327.s003.zip › EM COMPLETE SAMPLES SPECTRUM/EM2 SPECTRUM RT 3.484.pdf]

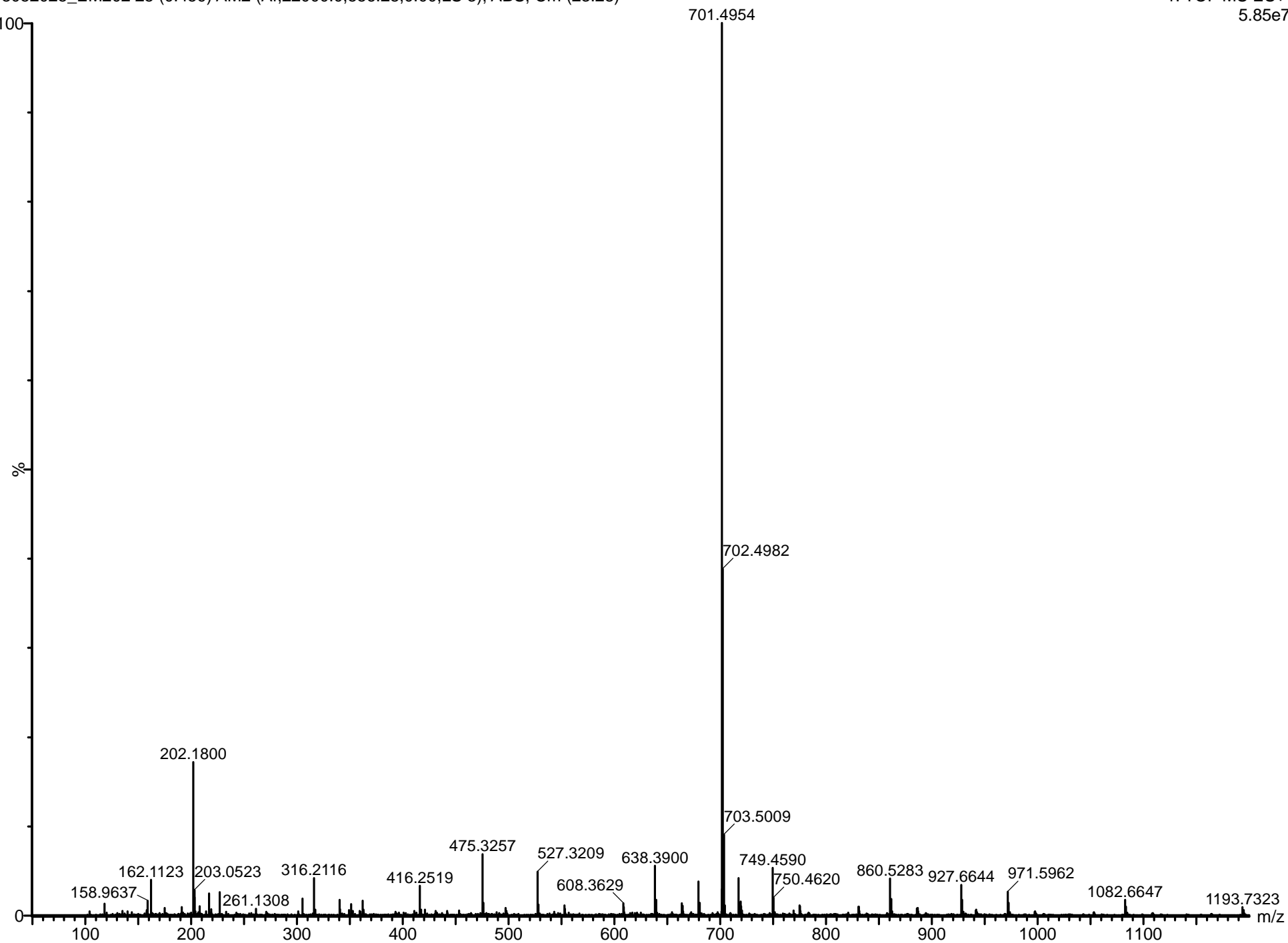

Supplement: S1 Data — Electrospray ionisation time of flight mass spectrometry (ESI-TOF MS, positive mode) spectra of the dengue cohort and ESI-TOF at different retention times. The spectra display the relative abundance (%) of detected ions across the m/z range. Prominent peaks corresponding to major ionised species are indicated. Variation in spectral profiles between retention times reflects the differences in compound composition and ionisation patterns within the sample. Data were acquired under identical instrumental conditions and are presented as representative scans. (ZIP) [file pntd.0014327.s003.zip › EM COMPLETE SAMPLES SPECTRUM/EM202 SPECTRUM RT 0.459.pdf]

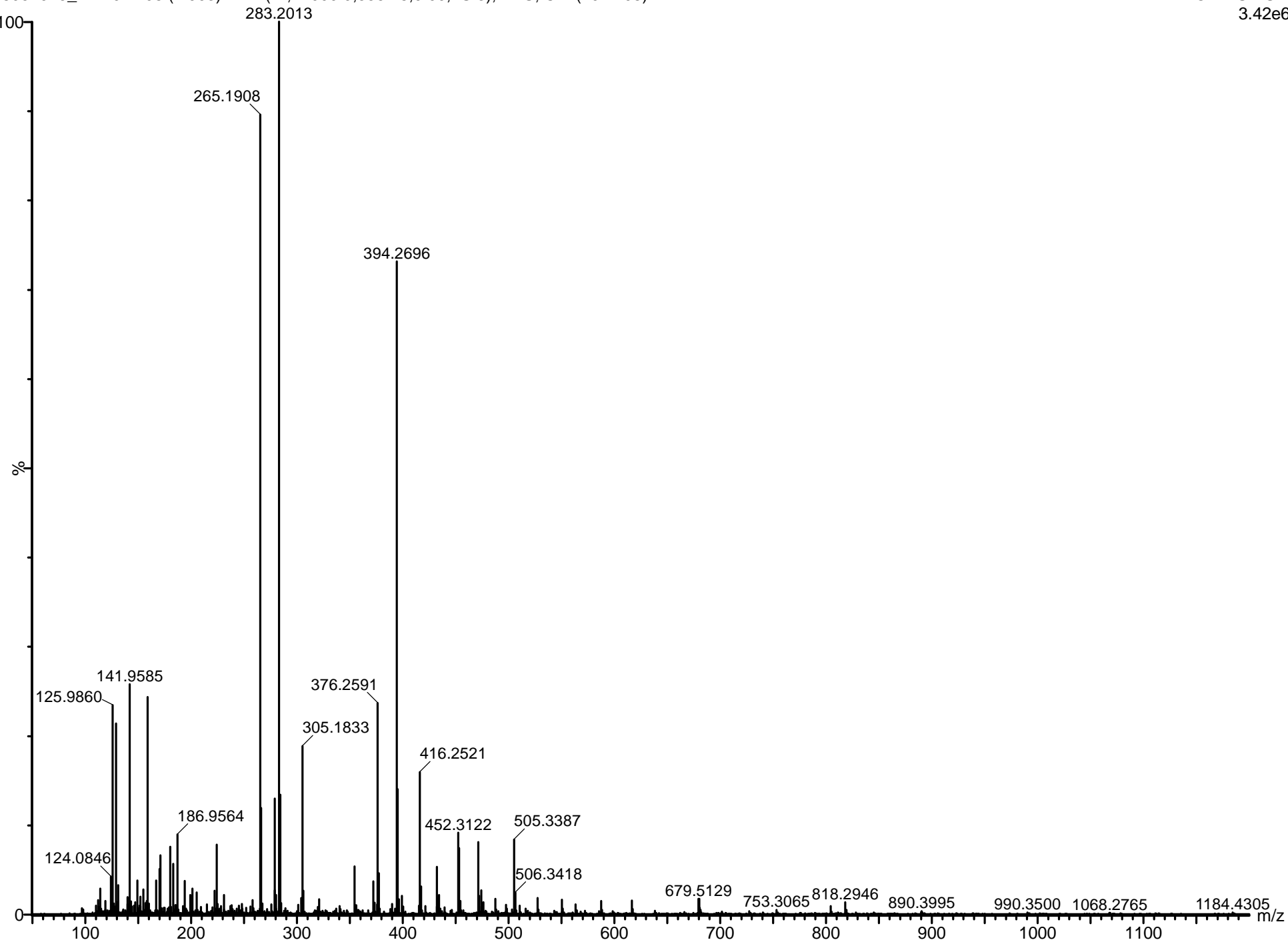

Supplement: S1 Data — Electrospray ionisation time of flight mass spectrometry (ESI-TOF MS, positive mode) spectra of the dengue cohort and ESI-TOF at different retention times. The spectra display the relative abundance (%) of detected ions across the m/z range. Prominent peaks corresponding to major ionised species are indicated. Variation in spectral profiles between retention times reflects the differences in compound composition and ionisation patterns within the sample. Data were acquired under identical instrumental conditions and are presented as representative scans. (ZIP) [file pntd.0014327.s003.zip › EM COMPLETE SAMPLES SPECTRUM/EM202 SPECTRUM RT 1.093.pdf]

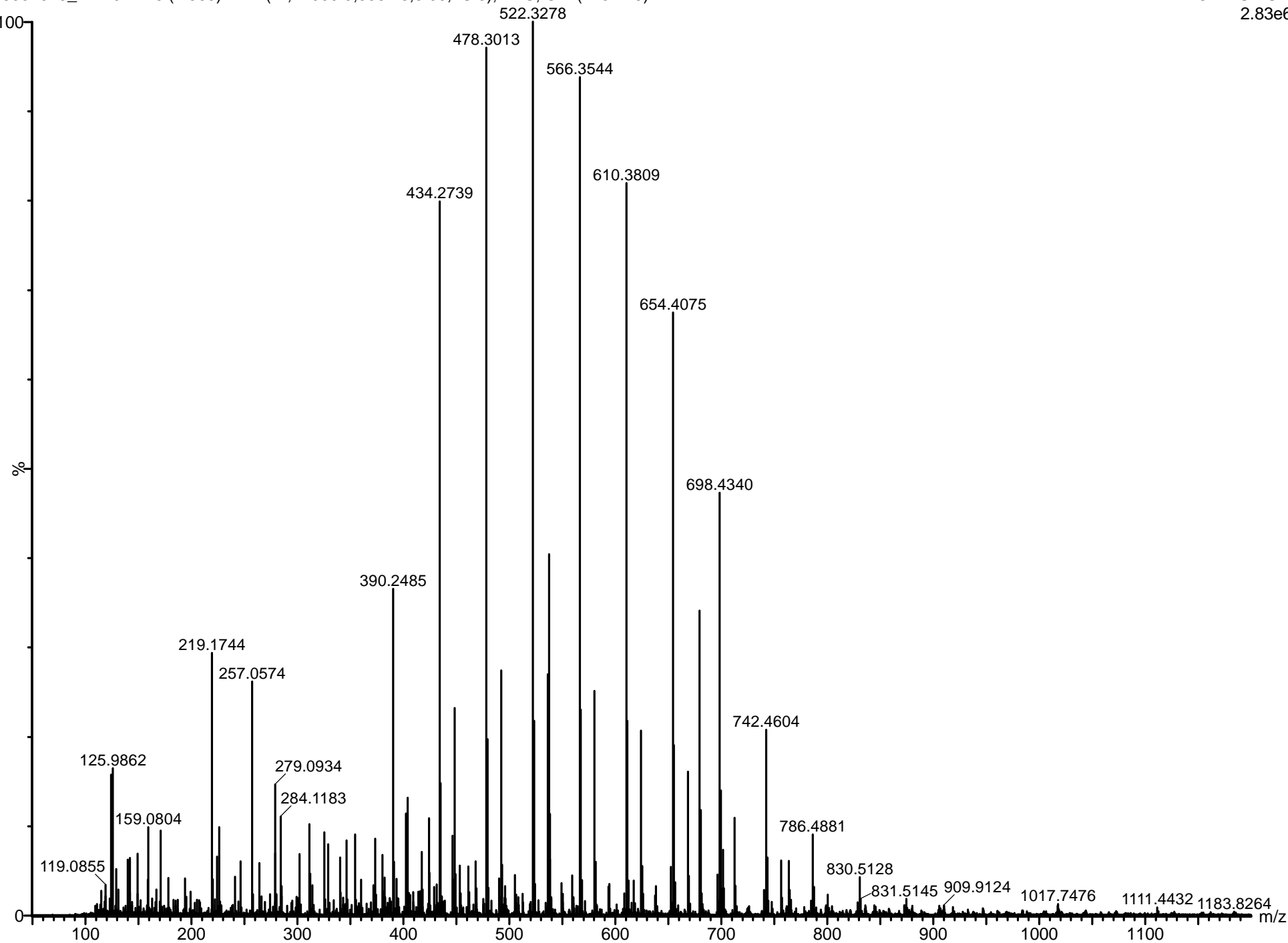

Supplement: S1 Data — Electrospray ionisation time of flight mass spectrometry (ESI-TOF MS, positive mode) spectra of the dengue cohort and ESI-TOF at different retention times. The spectra display the relative abundance (%) of detected ions across the m/z range. Prominent peaks corresponding to major ionised species are indicated. Variation in spectral profiles between retention times reflects the differences in compound composition and ionisation patterns within the sample. Data were acquired under identical instrumental conditions and are presented as representative scans. (ZIP) [file pntd.0014327.s003.zip › EM COMPLETE SAMPLES SPECTRUM/EM202 SPECTRUM RT 2.565.pdf]

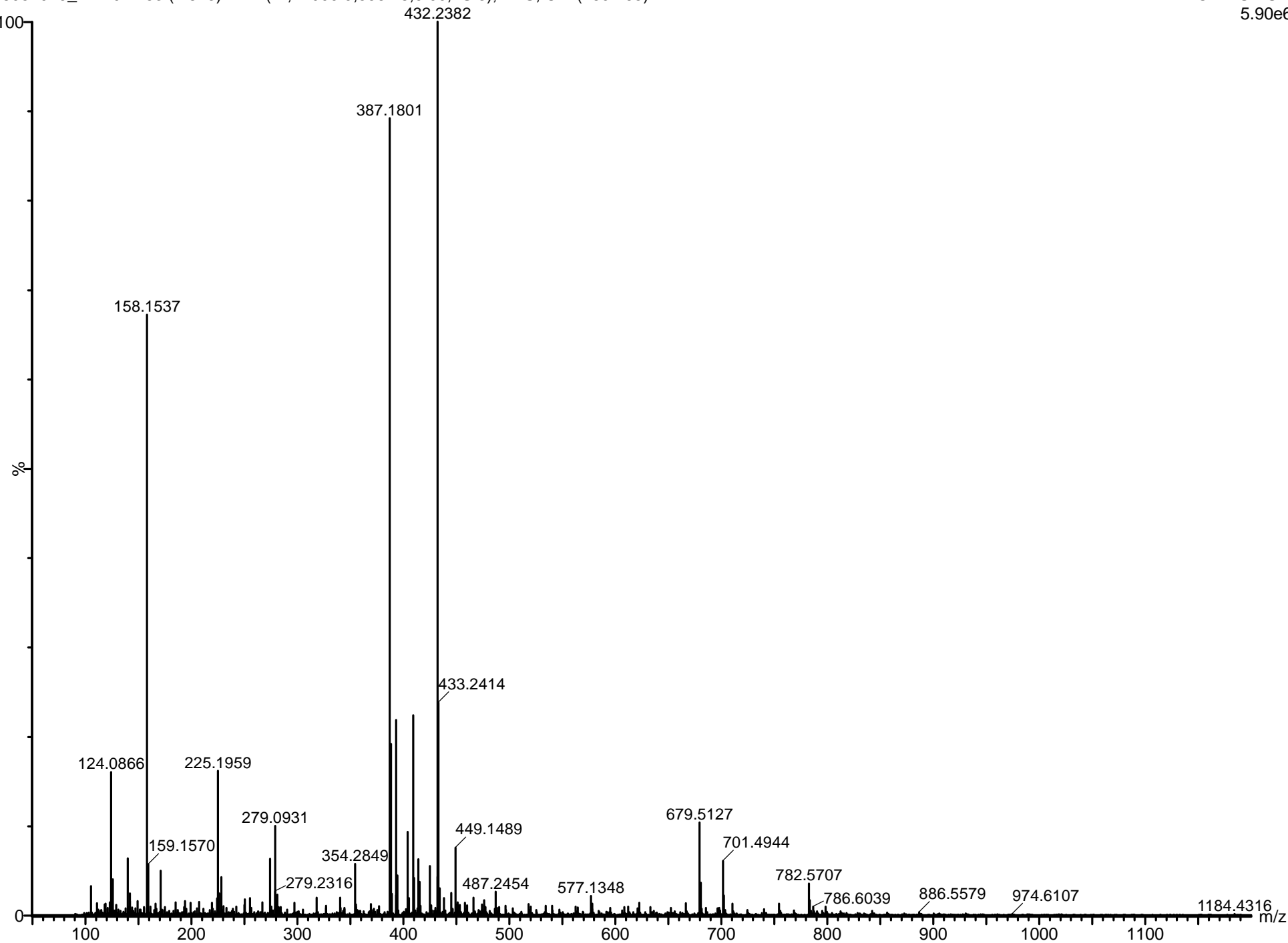

Supplement: S1 Data — Electrospray ionisation time of flight mass spectrometry (ESI-TOF MS, positive mode) spectra of the dengue cohort and ESI-TOF at different retention times. The spectra display the relative abundance (%) of detected ions across the m/z range. Prominent peaks corresponding to major ionised species are indicated. Variation in spectral profiles between retention times reflects the differences in compound composition and ionisation patterns within the sample. Data were acquired under identical instrumental conditions and are presented as representative scans. (ZIP) [file pntd.0014327.s003.zip › EM COMPLETE SAMPLES SPECTRUM/EM202 SPECTRUM RT 2.873.pdf]

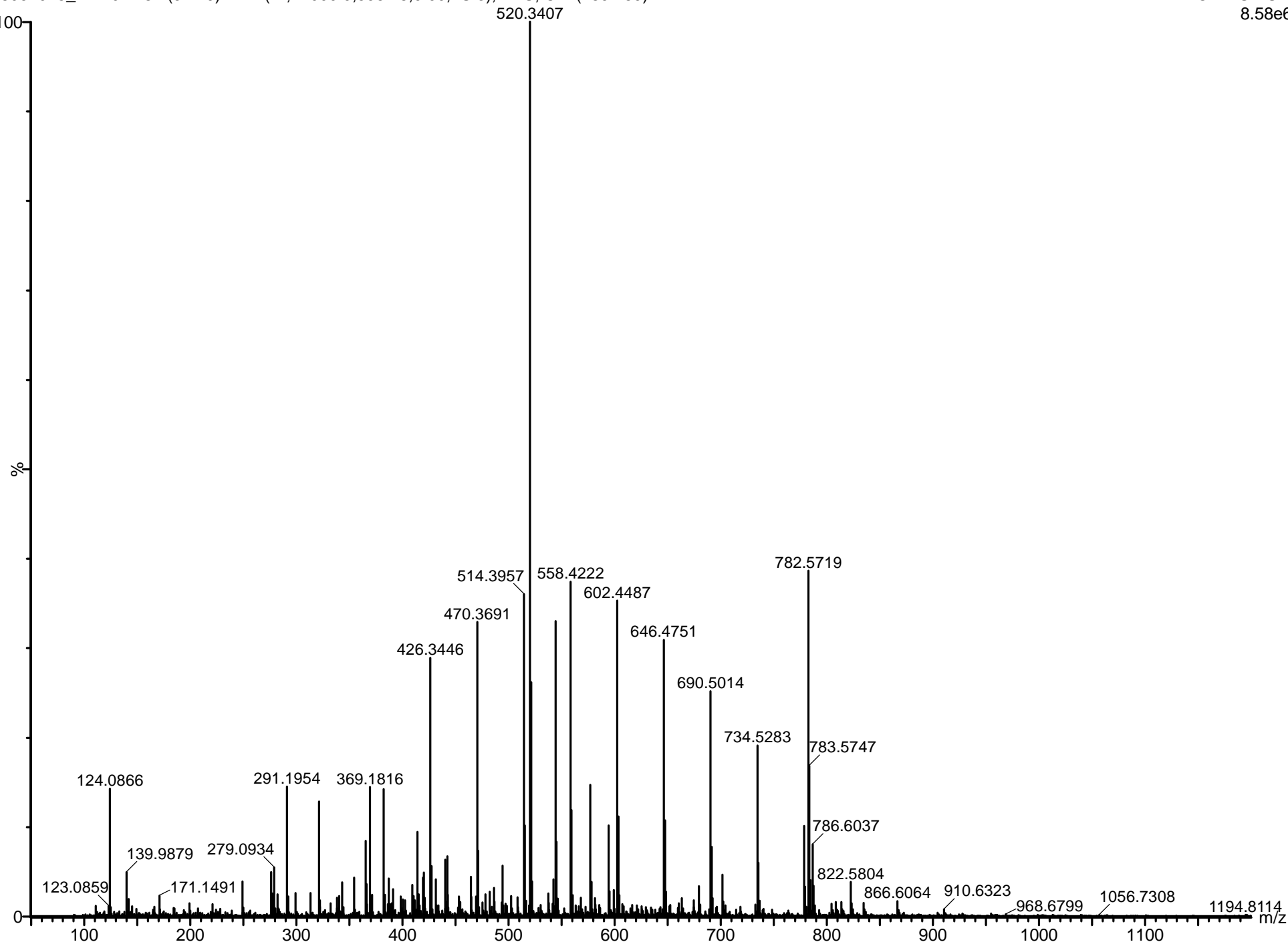

Supplement: S1 Data — Electrospray ionisation time of flight mass spectrometry (ESI-TOF MS, positive mode) spectra of the dengue cohort and ESI-TOF at different retention times. The spectra display the relative abundance (%) of detected ions across the m/z range. Prominent peaks corresponding to major ionised species are indicated. Variation in spectral profiles between retention times reflects the differences in compound composition and ionisation patterns within the sample. Data were acquired under identical instrumental conditions and are presented as representative scans. (ZIP) [file pntd.0014327.s003.zip › EM COMPLETE SAMPLES SPECTRUM/EM202 SPECTRUM RT 3.279.pdf]

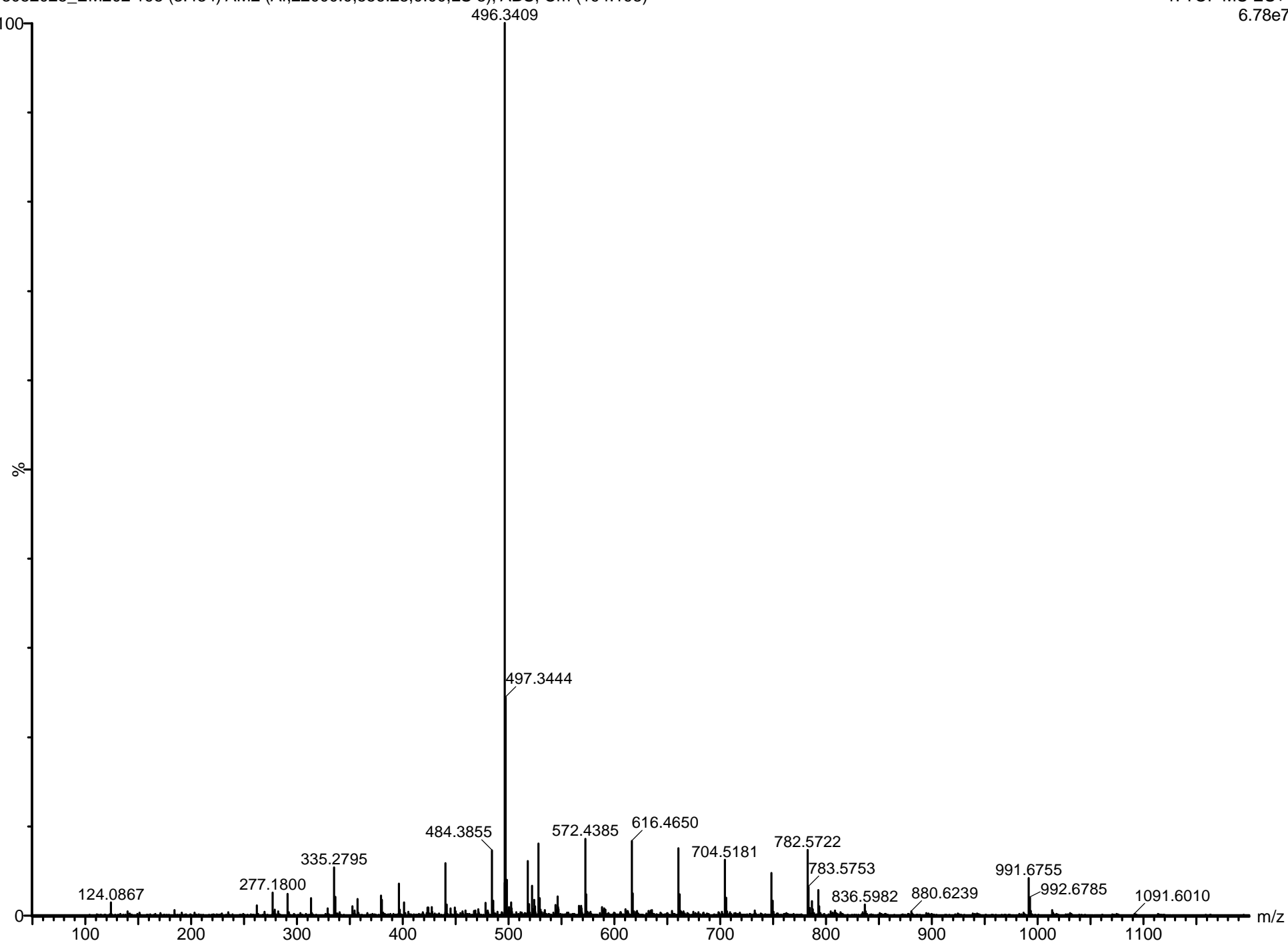

Supplement: S1 Data — Electrospray ionisation time of flight mass spectrometry (ESI-TOF MS, positive mode) spectra of the dengue cohort and ESI-TOF at different retention times. The spectra display the relative abundance (%) of detected ions across the m/z range. Prominent peaks corresponding to major ionised species are indicated. Variation in spectral profiles between retention times reflects the differences in compound composition and ionisation patterns within the sample. Data were acquired under identical instrumental conditions and are presented as representative scans. (ZIP) [file pntd.0014327.s003.zip › EM COMPLETE SAMPLES SPECTRUM/EM202 SPECTRUM RT 3.434.pdf]

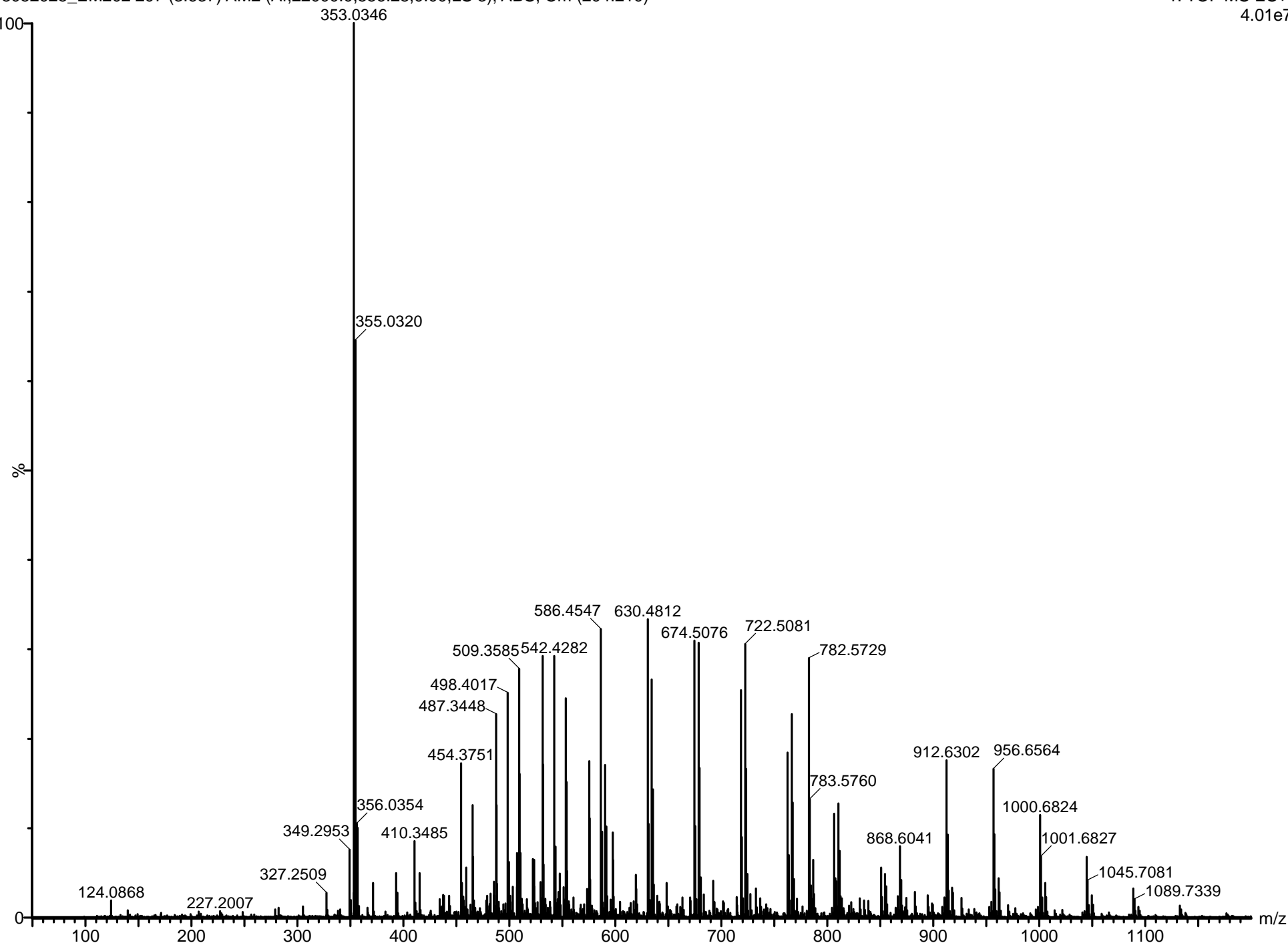

Supplement: S1 Data — Electrospray ionisation time of flight mass spectrometry (ESI-TOF MS, positive mode) spectra of the dengue cohort and ESI-TOF at different retention times. The spectra display the relative abundance (%) of detected ions across the m/z range. Prominent peaks corresponding to major ionised species are indicated. Variation in spectral profiles between retention times reflects the differences in compound composition and ionisation patterns within the sample. Data were acquired under identical instrumental conditions and are presented as representative scans. (ZIP) [file pntd.0014327.s003.zip › EM COMPLETE SAMPLES SPECTRUM/EM202 SPECTRUM RT 3.637.pdf]

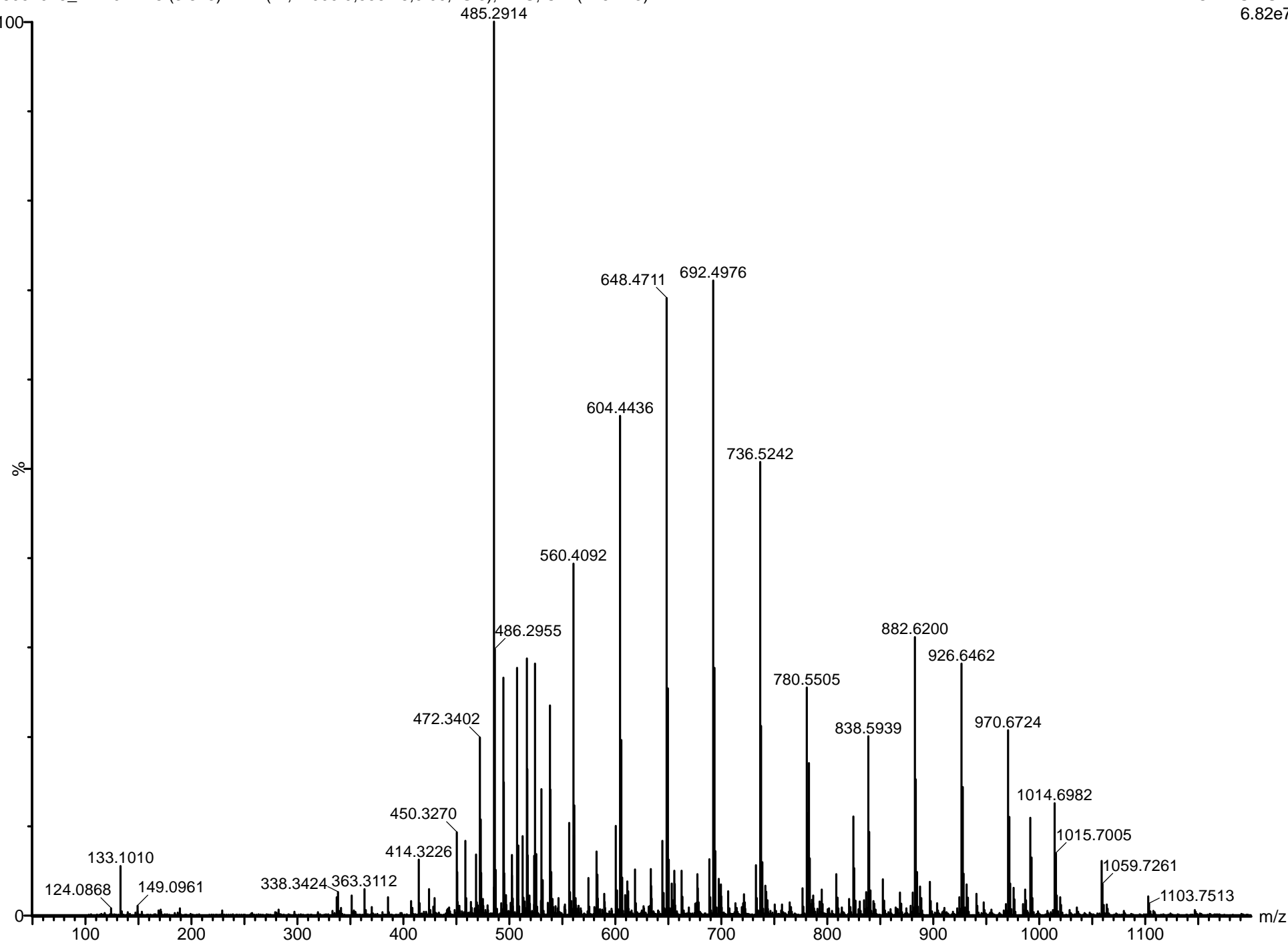

Supplement: S1 Data — Electrospray ionisation time of flight mass spectrometry (ESI-TOF MS, positive mode) spectra of the dengue cohort and ESI-TOF at different retention times. The spectra display the relative abundance (%) of detected ions across the m/z range. Prominent peaks corresponding to major ionised species are indicated. Variation in spectral profiles between retention times reflects the differences in compound composition and ionisation patterns within the sample. Data were acquired under identical instrumental conditions and are presented as representative scans. (ZIP) [file pntd.0014327.s003.zip › EM COMPLETE SAMPLES SPECTRUM/EM202 SPECTRUM RT 3.823.pdf]

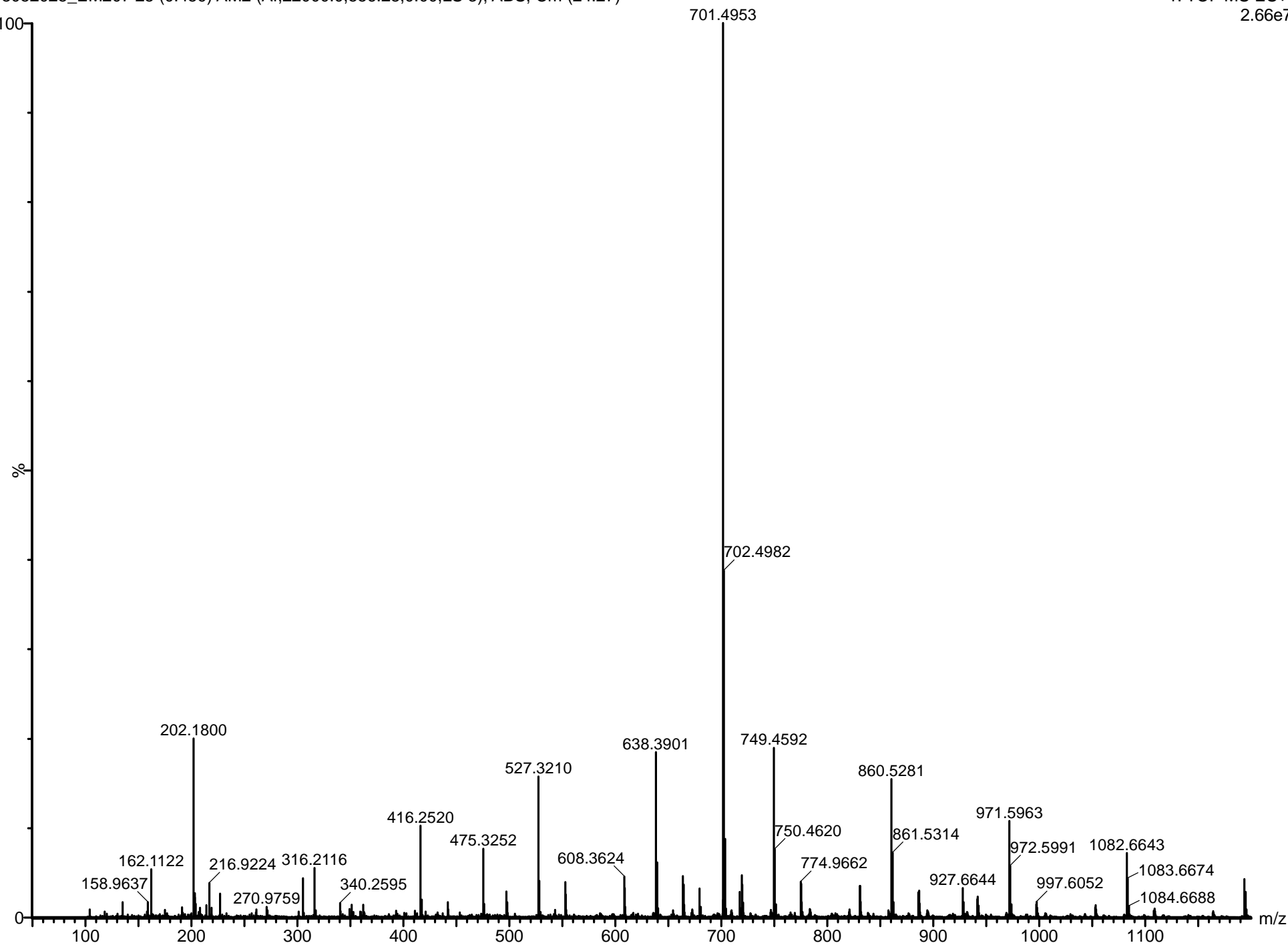

Supplement: S1 Data — Electrospray ionisation time of flight mass spectrometry (ESI-TOF MS, positive mode) spectra of the dengue cohort and ESI-TOF at different retention times. The spectra display the relative abundance (%) of detected ions across the m/z range. Prominent peaks corresponding to major ionised species are indicated. Variation in spectral profiles between retention times reflects the differences in compound composition and ionisation patterns within the sample. Data were acquired under identical instrumental conditions and are presented as representative scans. (ZIP) [file pntd.0014327.s003.zip › EM COMPLETE SAMPLES SPECTRUM/EM207 SPECTRUM RT 0.459.pdf]

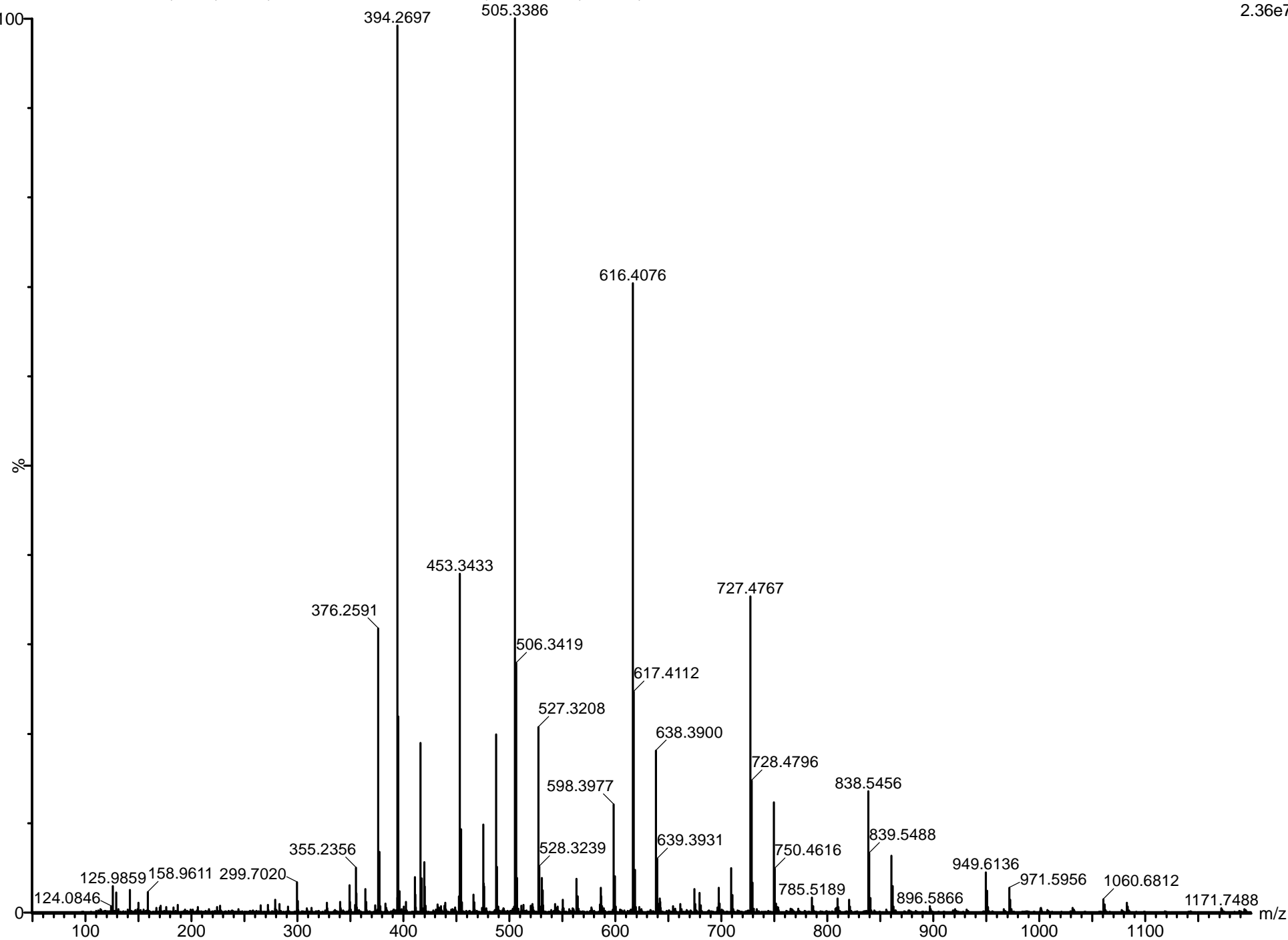

Supplement: S1 Data — Electrospray ionisation time of flight mass spectrometry (ESI-TOF MS, positive mode) spectra of the dengue cohort and ESI-TOF at different retention times. The spectra display the relative abundance (%) of detected ions across the m/z range. Prominent peaks corresponding to major ionised species are indicated. Variation in spectral profiles between retention times reflects the differences in compound composition and ionisation patterns within the sample. Data were acquired under identical instrumental conditions and are presented as representative scans. (ZIP) [file pntd.0014327.s003.zip › EM COMPLETE SAMPLES SPECTRUM/EM207 SPECTRUM RT 2.075.pdf]

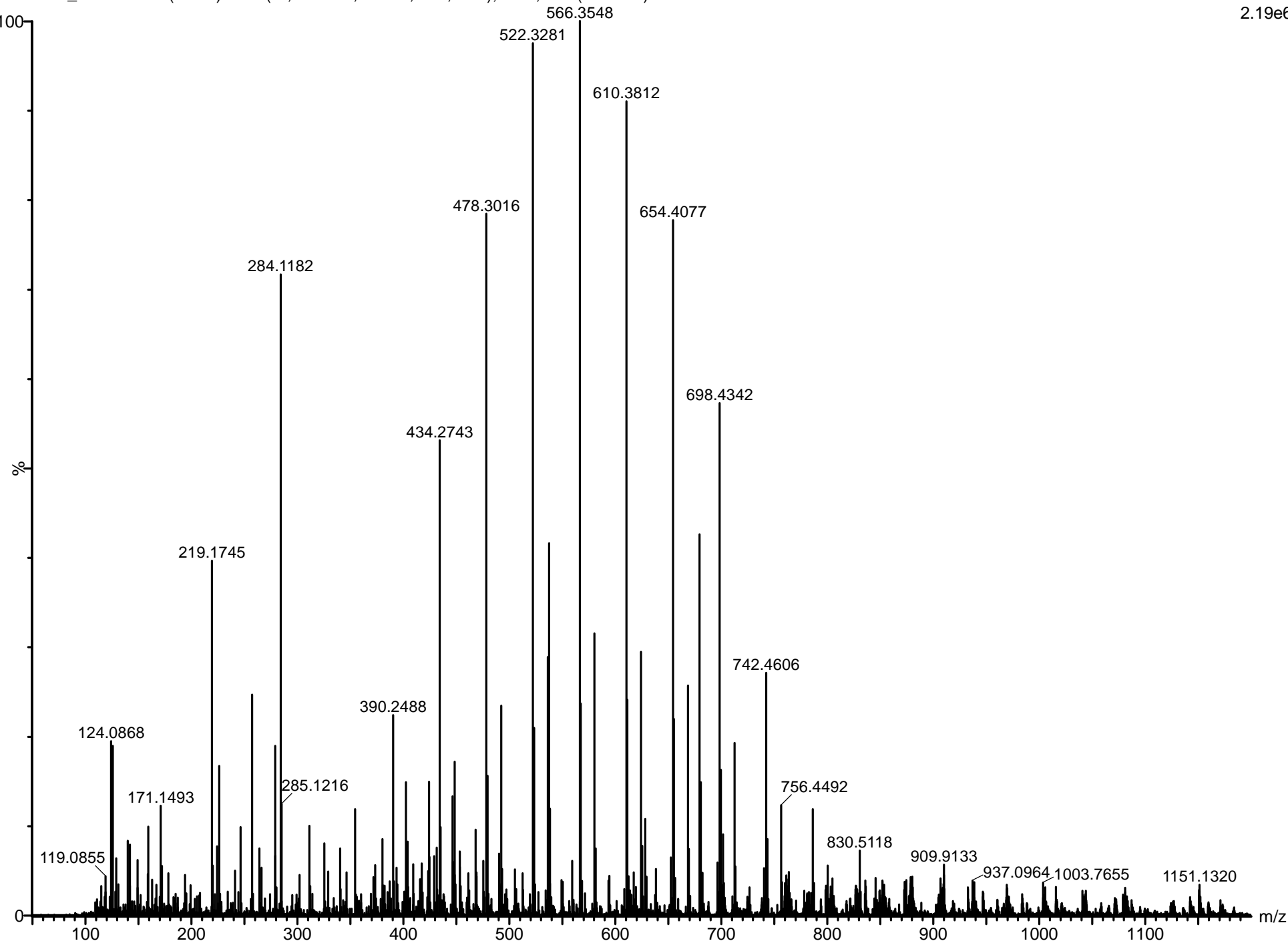

Supplement: S1 Data — Electrospray ionisation time of flight mass spectrometry (ESI-TOF MS, positive mode) spectra of the dengue cohort and ESI-TOF at different retention times. The spectra display the relative abundance (%) of detected ions across the m/z range. Prominent peaks corresponding to major ionised species are indicated. Variation in spectral profiles between retention times reflects the differences in compound composition and ionisation patterns within the sample. Data were acquired under identical instrumental conditions and are presented as representative scans. (ZIP) [file pntd.0014327.s003.zip › EM COMPLETE SAMPLES SPECTRUM/EM207 SPECTRUM RT 2.548.pdf]

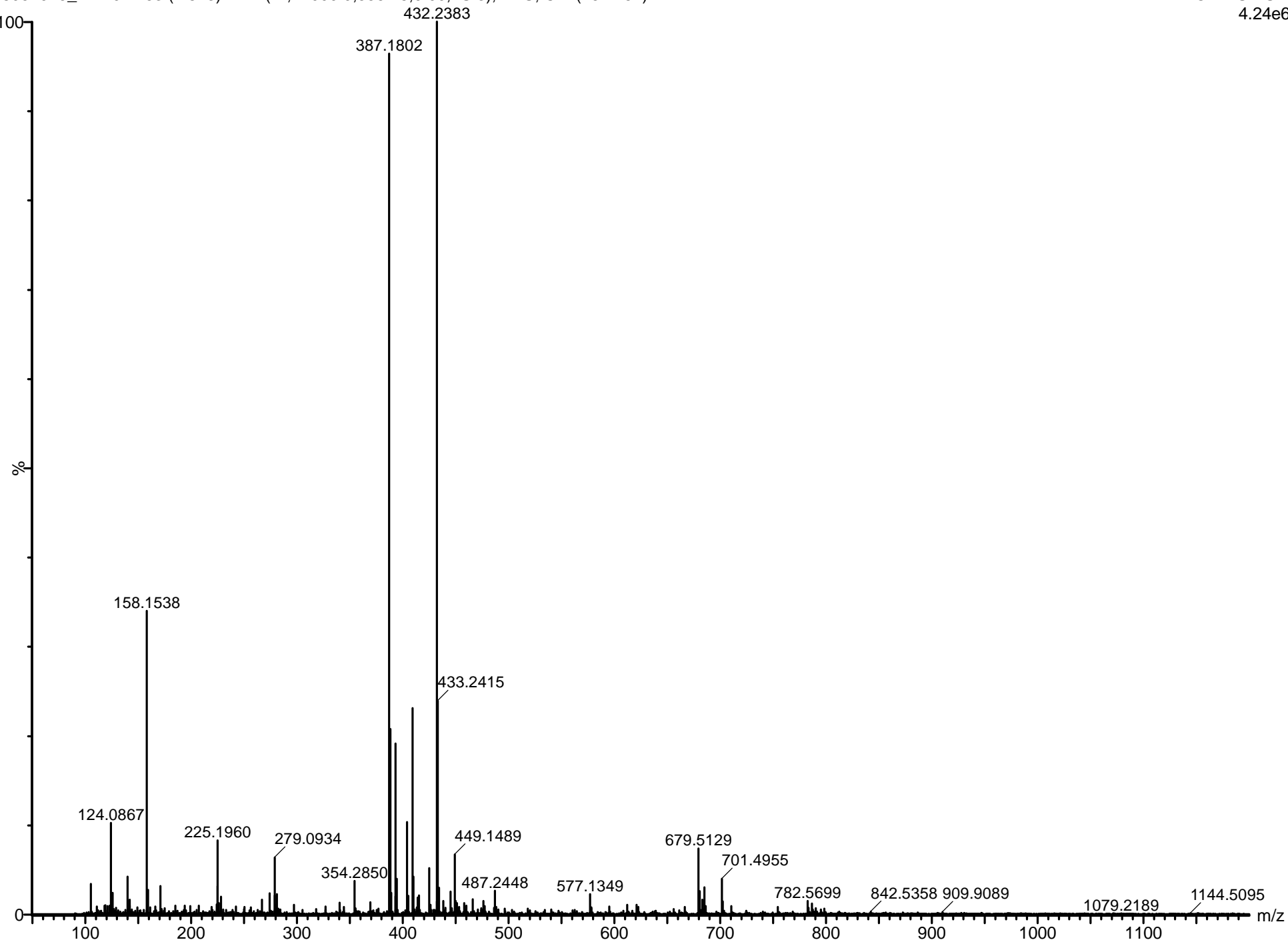

Supplement: S1 Data — Electrospray ionisation time of flight mass spectrometry (ESI-TOF MS, positive mode) spectra of the dengue cohort and ESI-TOF at different retention times. The spectra display the relative abundance (%) of detected ions across the m/z range. Prominent peaks corresponding to major ionised species are indicated. Variation in spectral profiles between retention times reflects the differences in compound composition and ionisation patterns within the sample. Data were acquired under identical instrumental conditions and are presented as representative scans. (ZIP) [file pntd.0014327.s003.zip › EM COMPLETE SAMPLES SPECTRUM/EM207 SPECTRUM RT 2.873.pdf]

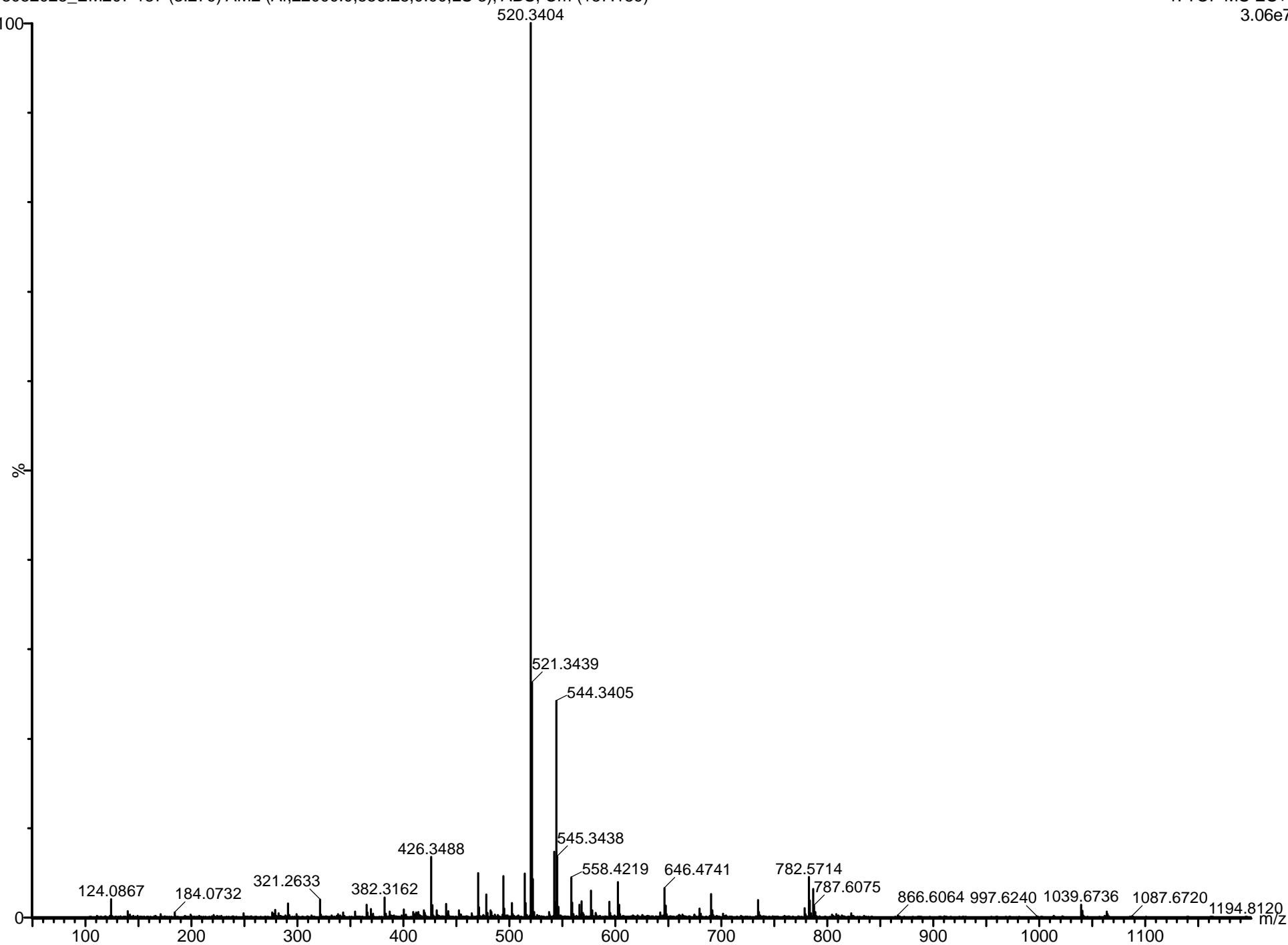

Supplement: S1 Data — Electrospray ionisation time of flight mass spectrometry (ESI-TOF MS, positive mode) spectra of the dengue cohort and ESI-TOF at different retention times. The spectra display the relative abundance (%) of detected ions across the m/z range. Prominent peaks corresponding to major ionised species are indicated. Variation in spectral profiles between retention times reflects the differences in compound composition and ionisation patterns within the sample. Data were acquired under identical instrumental conditions and are presented as representative scans. (ZIP) [file pntd.0014327.s003.zip › EM COMPLETE SAMPLES SPECTRUM/EM207 SPECTRUM RT 3.279.pdf]

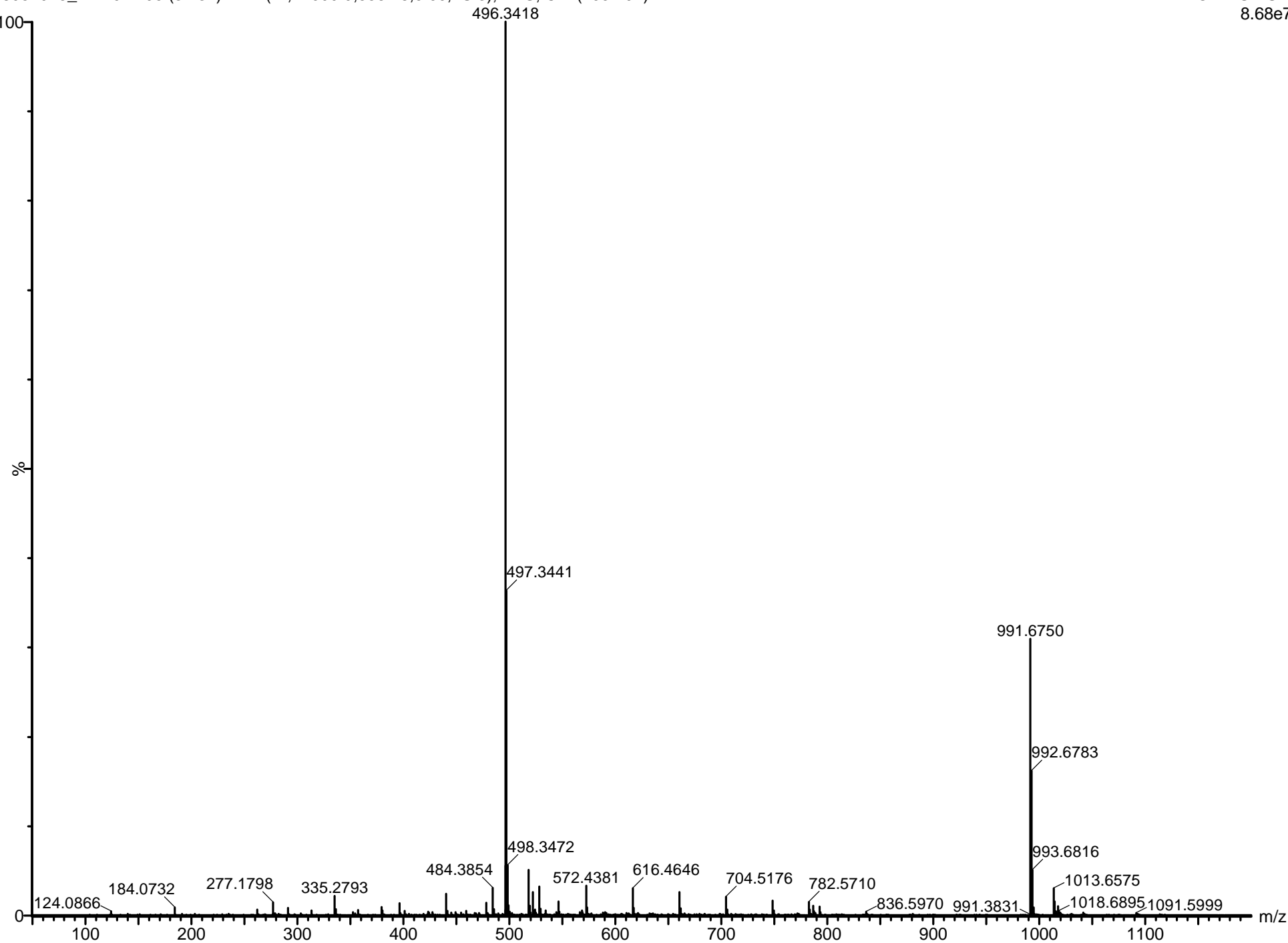

Supplement: S1 Data — Electrospray ionisation time of flight mass spectrometry (ESI-TOF MS, positive mode) spectra of the dengue cohort and ESI-TOF at different retention times. The spectra display the relative abundance (%) of detected ions across the m/z range. Prominent peaks corresponding to major ionised species are indicated. Variation in spectral profiles between retention times reflects the differences in compound composition and ionisation patterns within the sample. Data were acquired under identical instrumental conditions and are presented as representative scans. (ZIP) [file pntd.0014327.s003.zip › EM COMPLETE SAMPLES SPECTRUM/EM207 SPECTRUM RT 3.434.pdf]

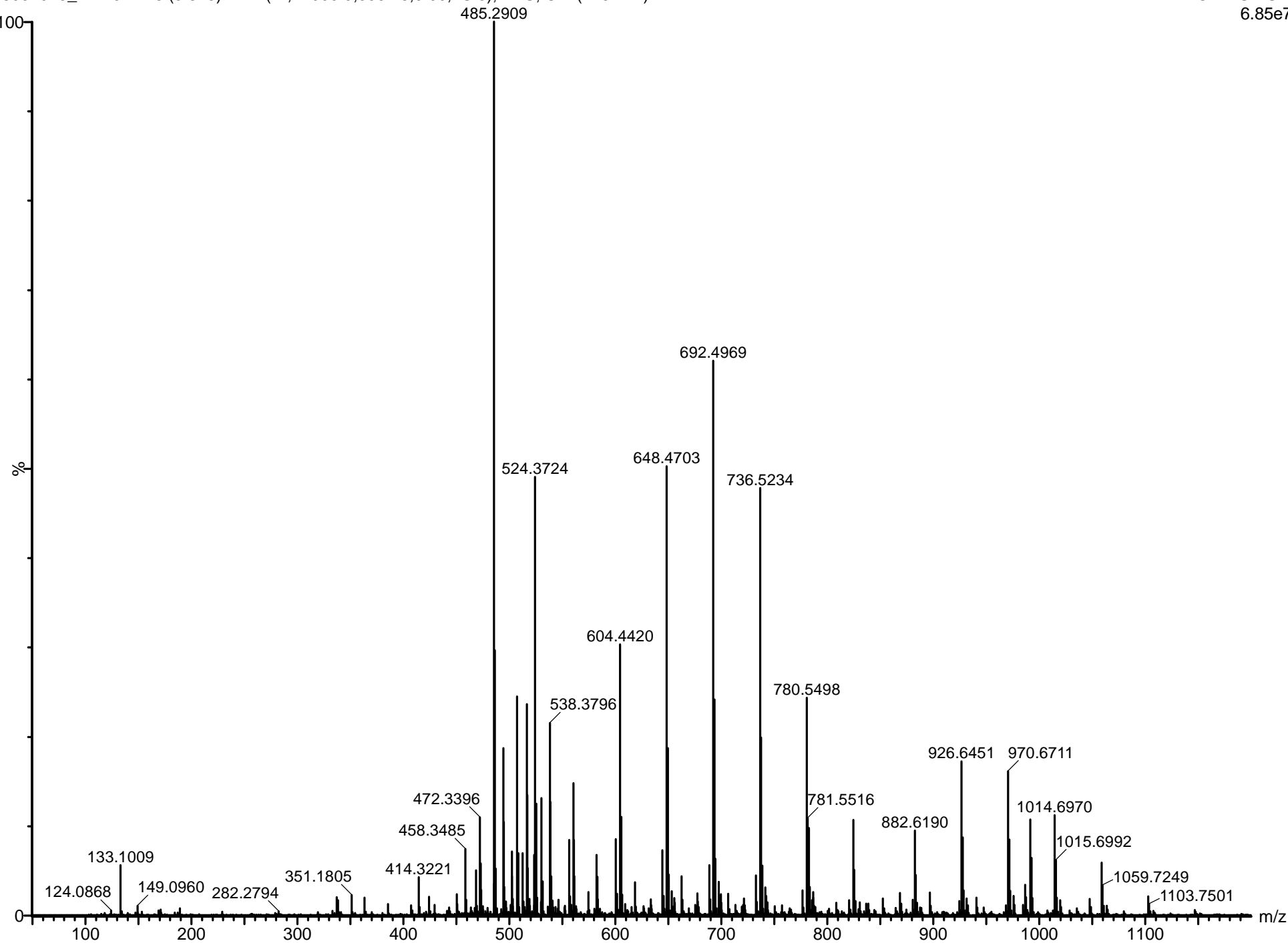

Supplement: S1 Data — Electrospray ionisation time of flight mass spectrometry (ESI-TOF MS, positive mode) spectra of the dengue cohort and ESI-TOF at different retention times. The spectra display the relative abundance (%) of detected ions across the m/z range. Prominent peaks corresponding to major ionised species are indicated. Variation in spectral profiles between retention times reflects the differences in compound composition and ionisation patterns within the sample. Data were acquired under identical instrumental conditions and are presented as representative scans. (ZIP) [file pntd.0014327.s003.zip › EM COMPLETE SAMPLES SPECTRUM/EM207 SPECTRUM RT 3.823.pdf]

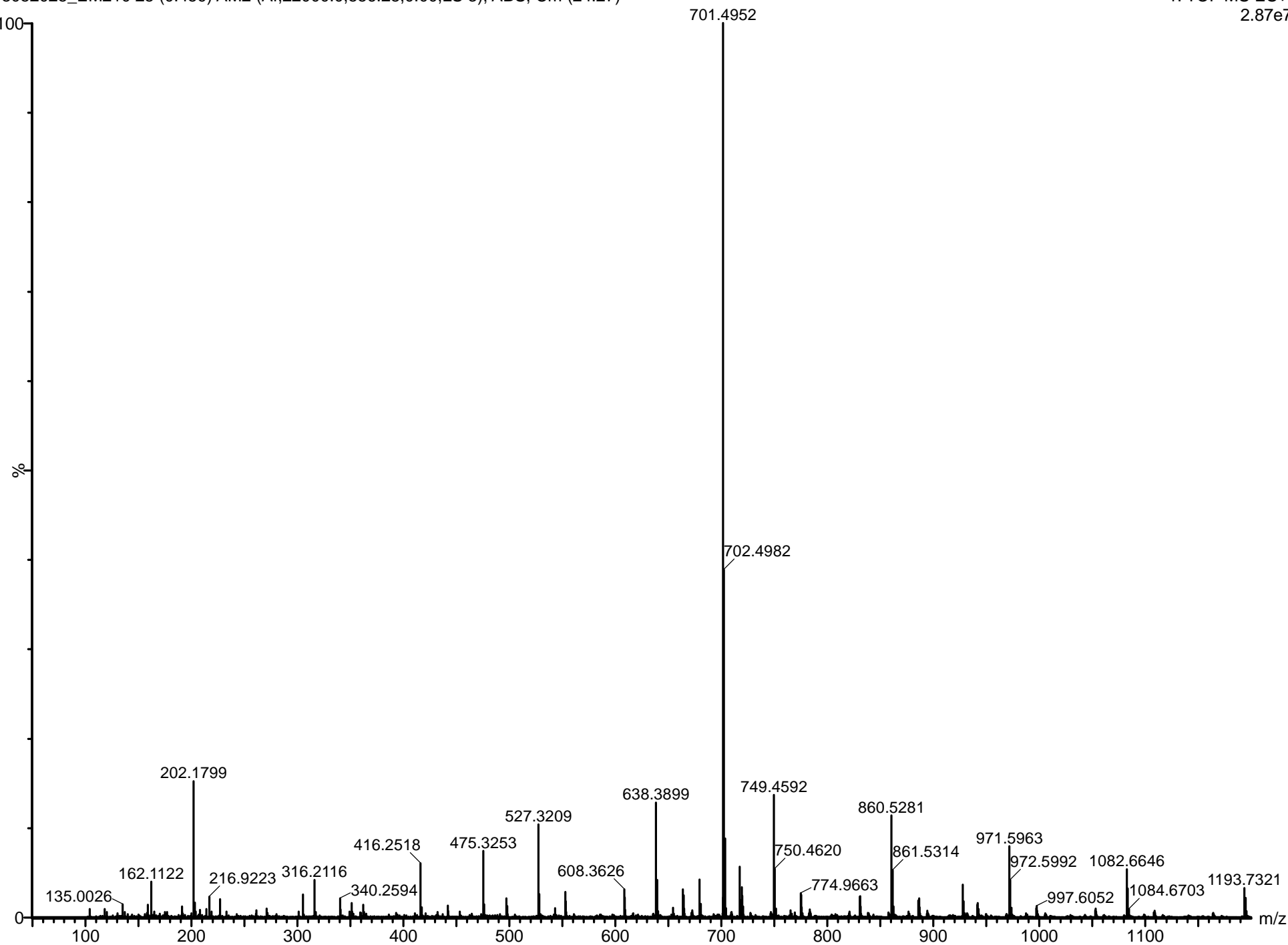

Supplement: S1 Data — Electrospray ionisation time of flight mass spectrometry (ESI-TOF MS, positive mode) spectra of the dengue cohort and ESI-TOF at different retention times. The spectra display the relative abundance (%) of detected ions across the m/z range. Prominent peaks corresponding to major ionised species are indicated. Variation in spectral profiles between retention times reflects the differences in compound composition and ionisation patterns within the sample. Data were acquired under identical instrumental conditions and are presented as representative scans. (ZIP) [file pntd.0014327.s003.zip › EM COMPLETE SAMPLES SPECTRUM/EM210 SPECTRUM RT 0.459.pdf]

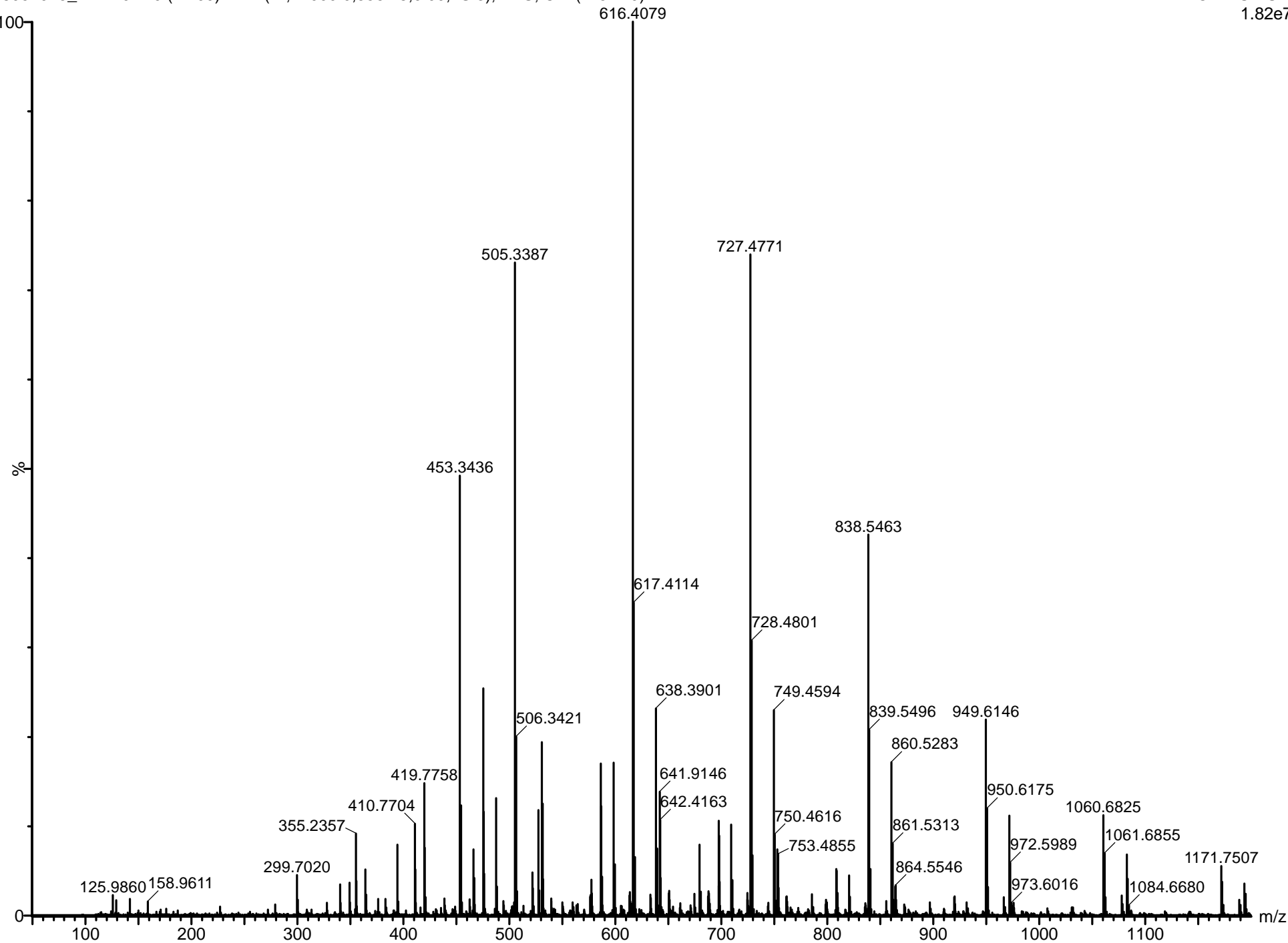

Supplement: S1 Data — Electrospray ionisation time of flight mass spectrometry (ESI-TOF MS, positive mode) spectra of the dengue cohort and ESI-TOF at different retention times. The spectra display the relative abundance (%) of detected ions across the m/z range. Prominent peaks corresponding to major ionised species are indicated. Variation in spectral profiles between retention times reflects the differences in compound composition and ionisation patterns within the sample. Data were acquired under identical instrumental conditions and are presented as representative scans. (ZIP) [file pntd.0014327.s003.zip › EM COMPLETE SAMPLES SPECTRUM/EM210 SPECTRUM RT 2.109.pdf]

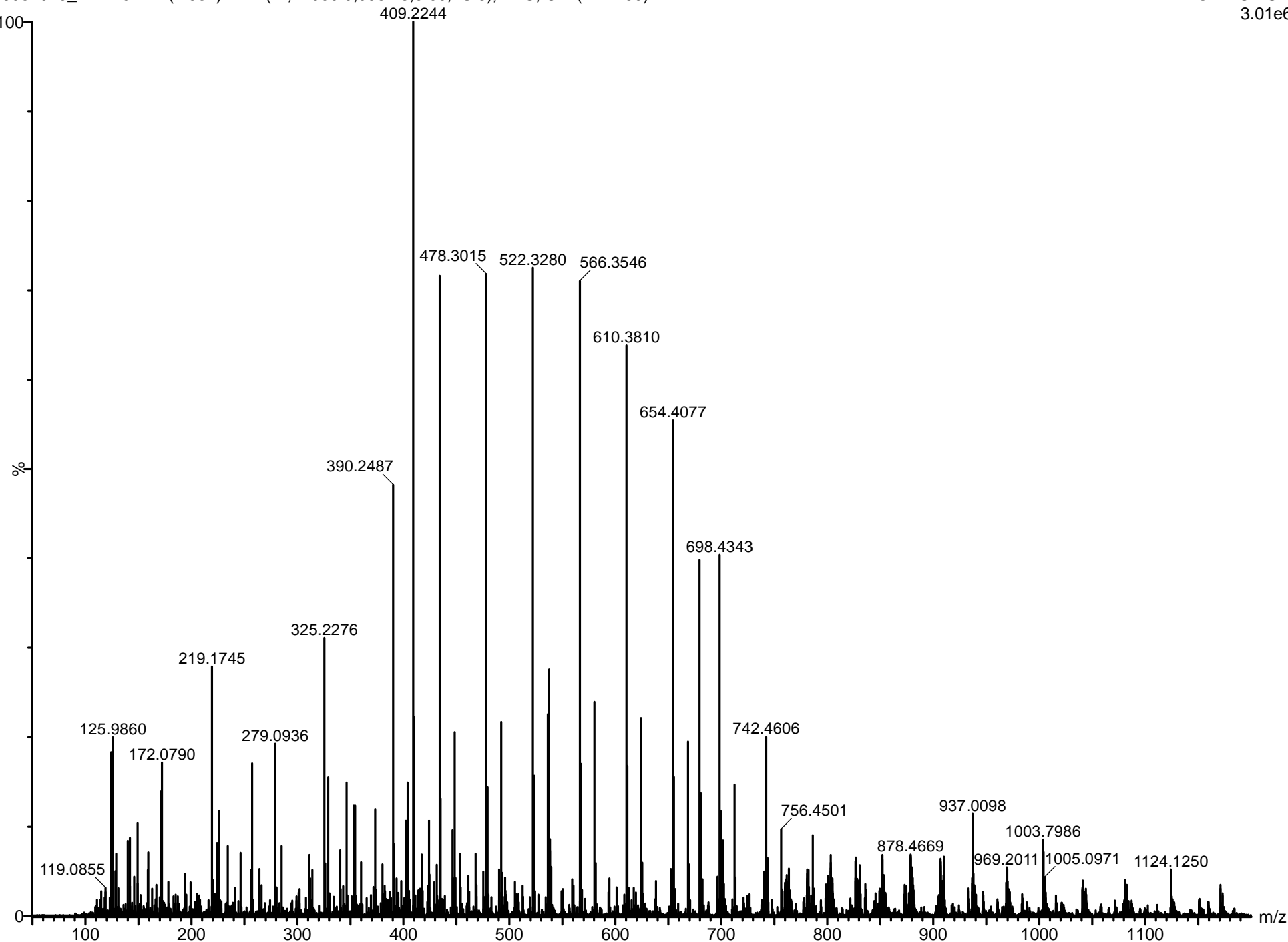

Supplement: S1 Data — Electrospray ionisation time of flight mass spectrometry (ESI-TOF MS, positive mode) spectra of the dengue cohort and ESI-TOF at different retention times. The spectra display the relative abundance (%) of detected ions across the m/z range. Prominent peaks corresponding to major ionised species are indicated. Variation in spectral profiles between retention times reflects the differences in compound composition and ionisation patterns within the sample. Data were acquired under identical instrumental conditions and are presented as representative scans. (ZIP) [file pntd.0014327.s003.zip › EM COMPLETE SAMPLES SPECTRUM/EM210 SPECTRUM RT 2.531.pdf]

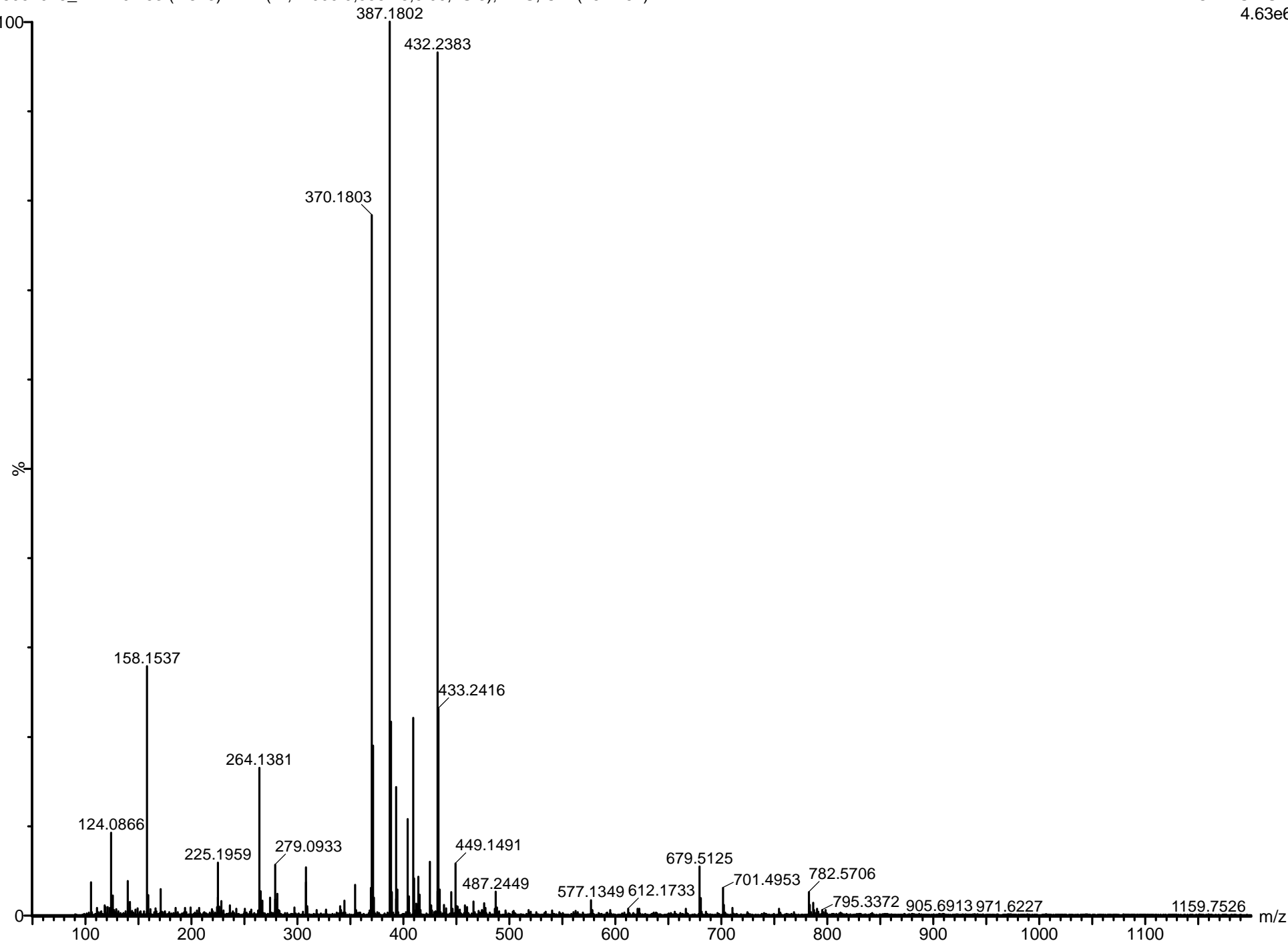

Supplement: S1 Data — Electrospray ionisation time of flight mass spectrometry (ESI-TOF MS, positive mode) spectra of the dengue cohort and ESI-TOF at different retention times. The spectra display the relative abundance (%) of detected ions across the m/z range. Prominent peaks corresponding to major ionised species are indicated. Variation in spectral profiles between retention times reflects the differences in compound composition and ionisation patterns within the sample. Data were acquired under identical instrumental conditions and are presented as representative scans. (ZIP) [file pntd.0014327.s003.zip › EM COMPLETE SAMPLES SPECTRUM/EM210 SPECTRUM RT 2.873.pdf]

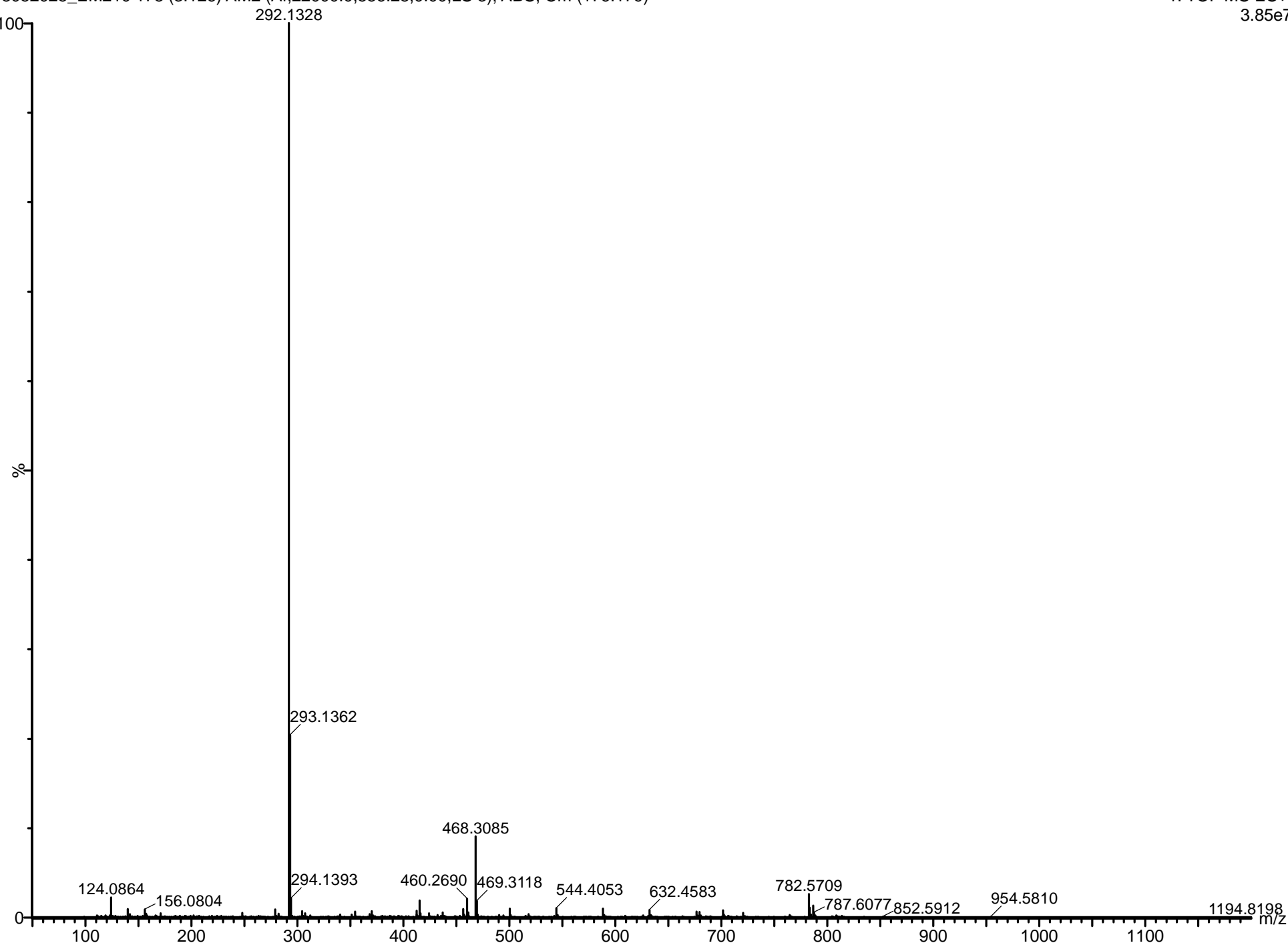

Supplement: S1 Data — Electrospray ionisation time of flight mass spectrometry (ESI-TOF MS, positive mode) spectra of the dengue cohort and ESI-TOF at different retention times. The spectra display the relative abundance (%) of detected ions across the m/z range. Prominent peaks corresponding to major ionised species are indicated. Variation in spectral profiles between retention times reflects the differences in compound composition and ionisation patterns within the sample. Data were acquired under identical instrumental conditions and are presented as representative scans. (ZIP) [file pntd.0014327.s003.zip › EM COMPLETE SAMPLES SPECTRUM/EM210 SPECTRUM RT 3.126.pdf]

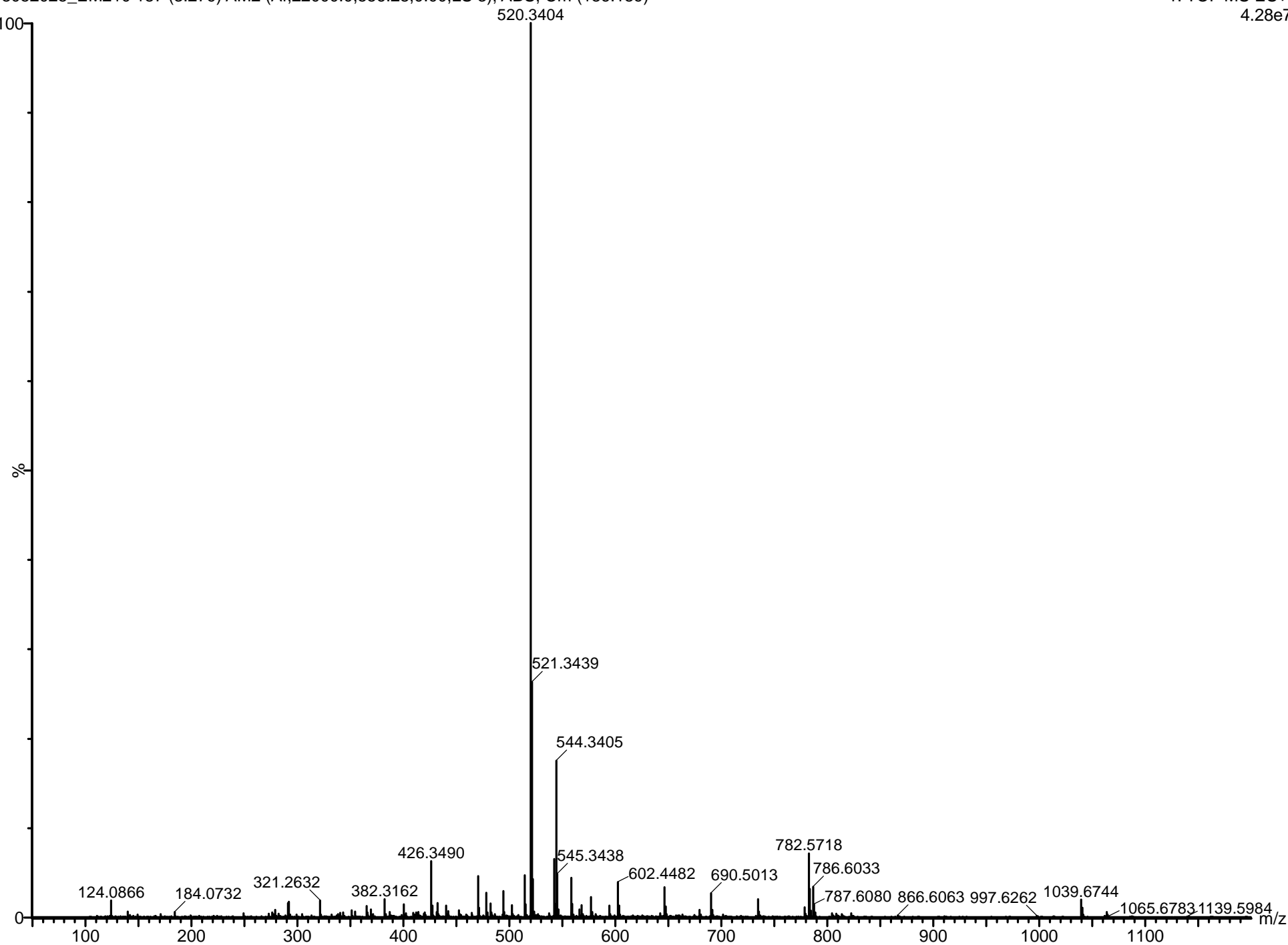

Supplement: S1 Data — Electrospray ionisation time of flight mass spectrometry (ESI-TOF MS, positive mode) spectra of the dengue cohort and ESI-TOF at different retention times. The spectra display the relative abundance (%) of detected ions across the m/z range. Prominent peaks corresponding to major ionised species are indicated. Variation in spectral profiles between retention times reflects the differences in compound composition and ionisation patterns within the sample. Data were acquired under identical instrumental conditions and are presented as representative scans. (ZIP) [file pntd.0014327.s003.zip › EM COMPLETE SAMPLES SPECTRUM/EM210 SPECTRUM RT 3.279.pdf]

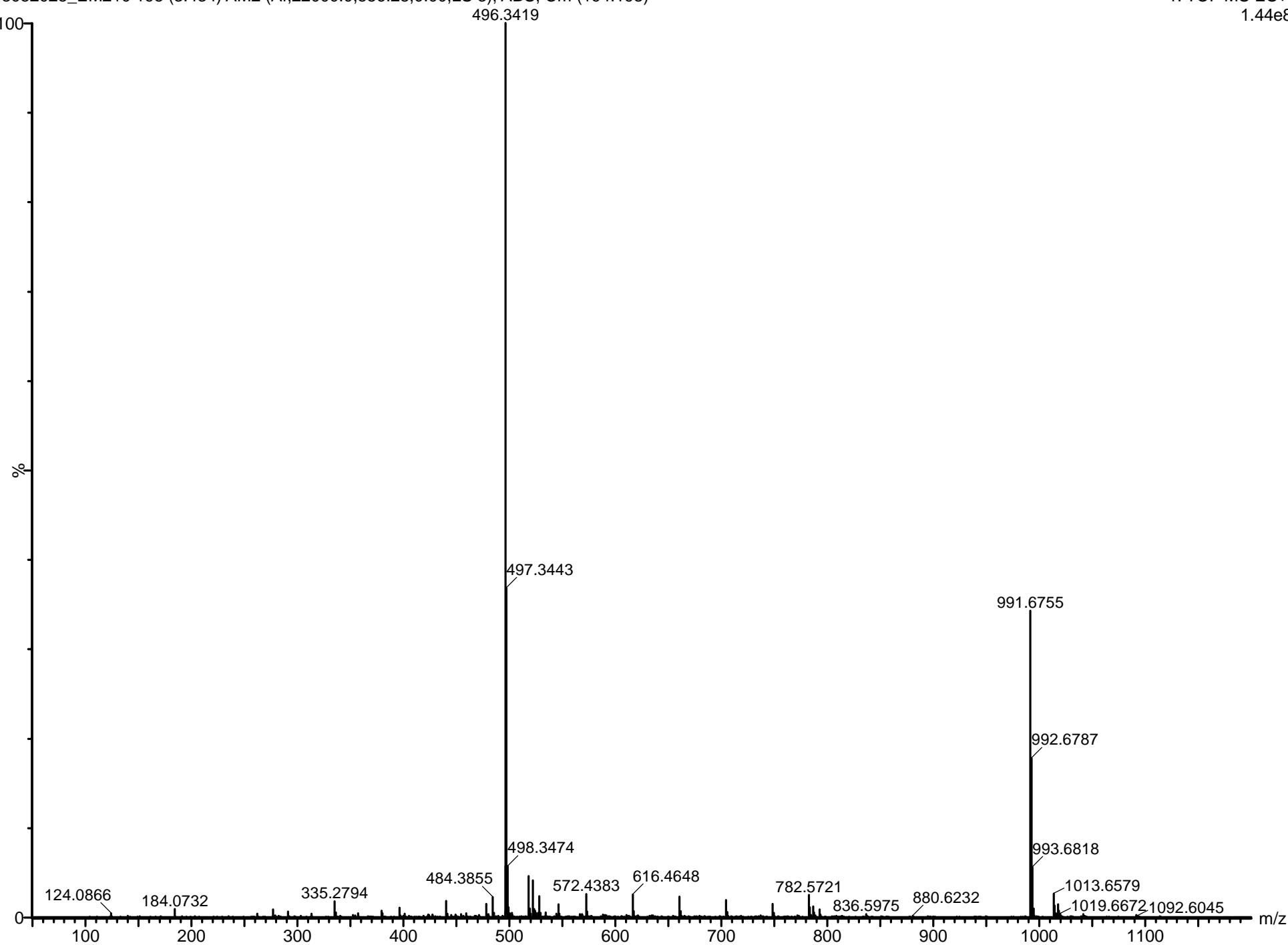

Supplement: S1 Data — Electrospray ionisation time of flight mass spectrometry (ESI-TOF MS, positive mode) spectra of the dengue cohort and ESI-TOF at different retention times. The spectra display the relative abundance (%) of detected ions across the m/z range. Prominent peaks corresponding to major ionised species are indicated. Variation in spectral profiles between retention times reflects the differences in compound composition and ionisation patterns within the sample. Data were acquired under identical instrumental conditions and are presented as representative scans. (ZIP) [file pntd.0014327.s003.zip › EM COMPLETE SAMPLES SPECTRUM/EM210 SPECTRUM RT 3.434.pdf]

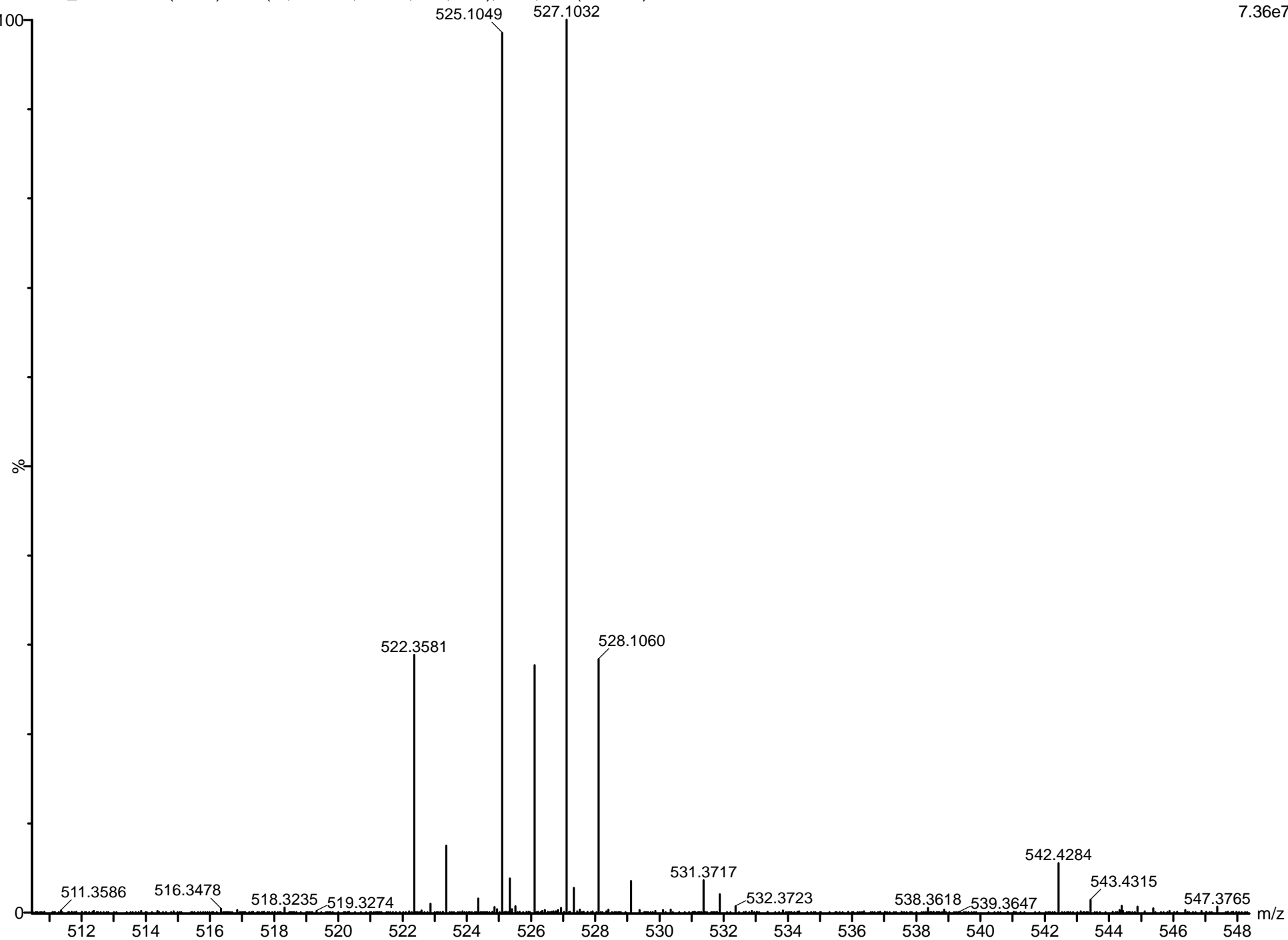

Supplement: S1 Data — Electrospray ionisation time of flight mass spectrometry (ESI-TOF MS, positive mode) spectra of the dengue cohort and ESI-TOF at different retention times. The spectra display the relative abundance (%) of detected ions across the m/z range. Prominent peaks corresponding to major ionised species are indicated. Variation in spectral profiles between retention times reflects the differences in compound composition and ionisation patterns within the sample. Data were acquired under identical instrumental conditions and are presented as representative scans. (ZIP) [file pntd.0014327.s003.zip › EM COMPLETE SAMPLES SPECTRUM/EM210 SPECTRUM RT 3.586 EX.pdf]

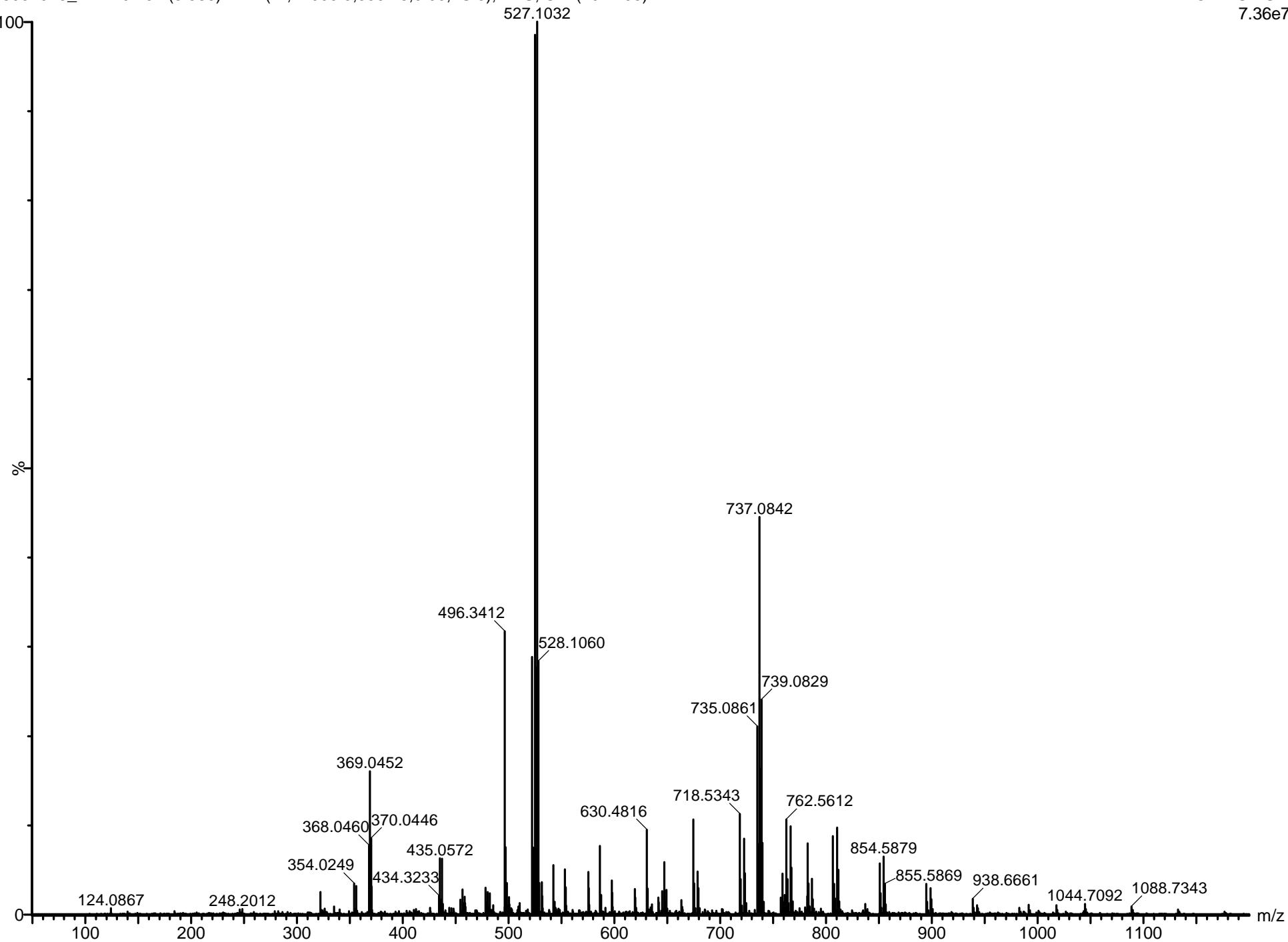

Supplement: S1 Data — Electrospray ionisation time of flight mass spectrometry (ESI-TOF MS, positive mode) spectra of the dengue cohort and ESI-TOF at different retention times. The spectra display the relative abundance (%) of detected ions across the m/z range. Prominent peaks corresponding to major ionised species are indicated. Variation in spectral profiles between retention times reflects the differences in compound composition and ionisation patterns within the sample. Data were acquired under identical instrumental conditions and are presented as representative scans. (ZIP) [file pntd.0014327.s003.zip › EM COMPLETE SAMPLES SPECTRUM/EM210 SPECTRUM RT 3.586.pdf]

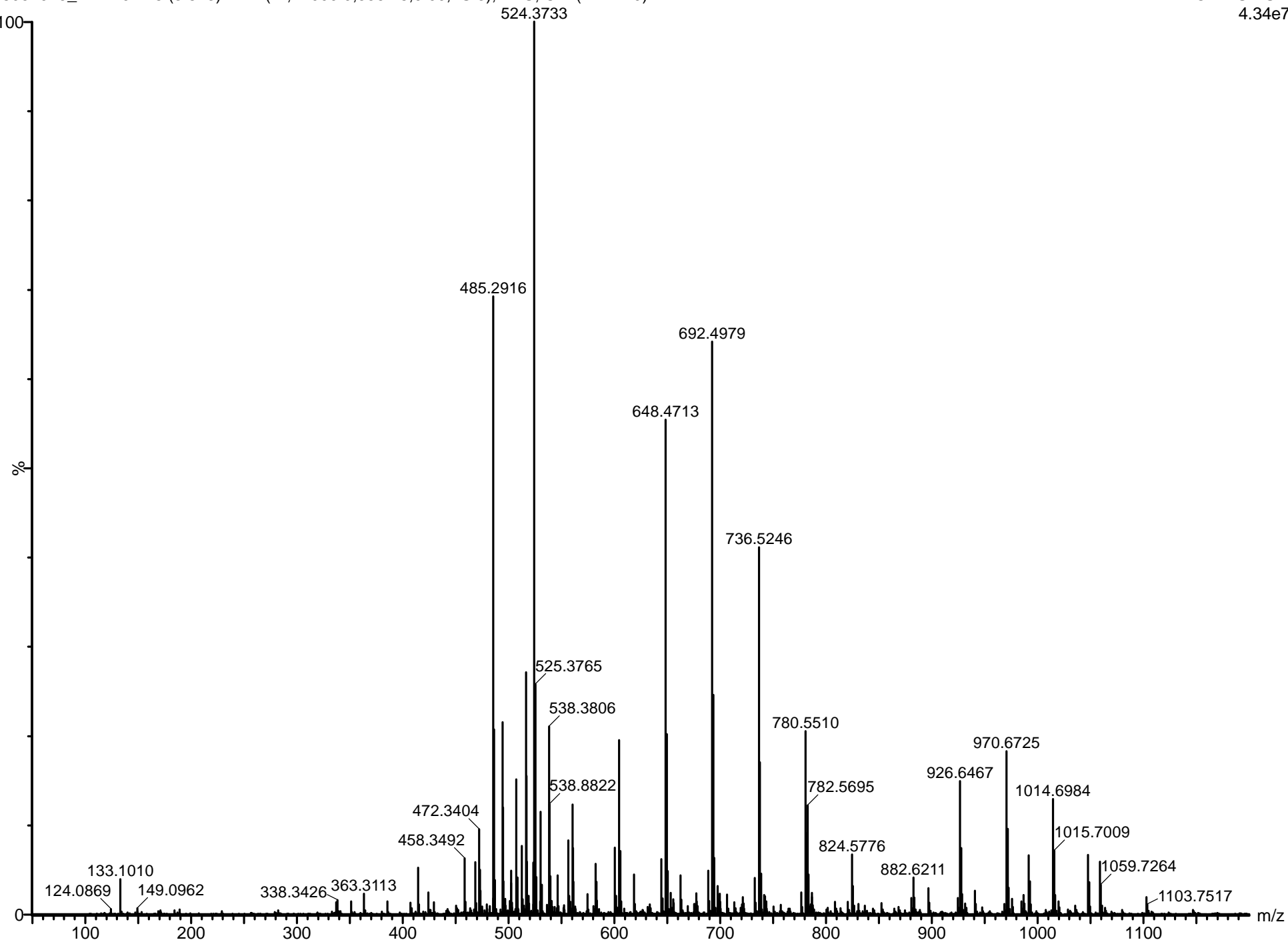

Supplement: S1 Data — Electrospray ionisation time of flight mass spectrometry (ESI-TOF MS, positive mode) spectra of the dengue cohort and ESI-TOF at different retention times. The spectra display the relative abundance (%) of detected ions across the m/z range. Prominent peaks corresponding to major ionised species are indicated. Variation in spectral profiles between retention times reflects the differences in compound composition and ionisation patterns within the sample. Data were acquired under identical instrumental conditions and are presented as representative scans. (ZIP) [file pntd.0014327.s003.zip › EM COMPLETE SAMPLES SPECTRUM/EM210 SPECTRUM RT 3.823.pdf]

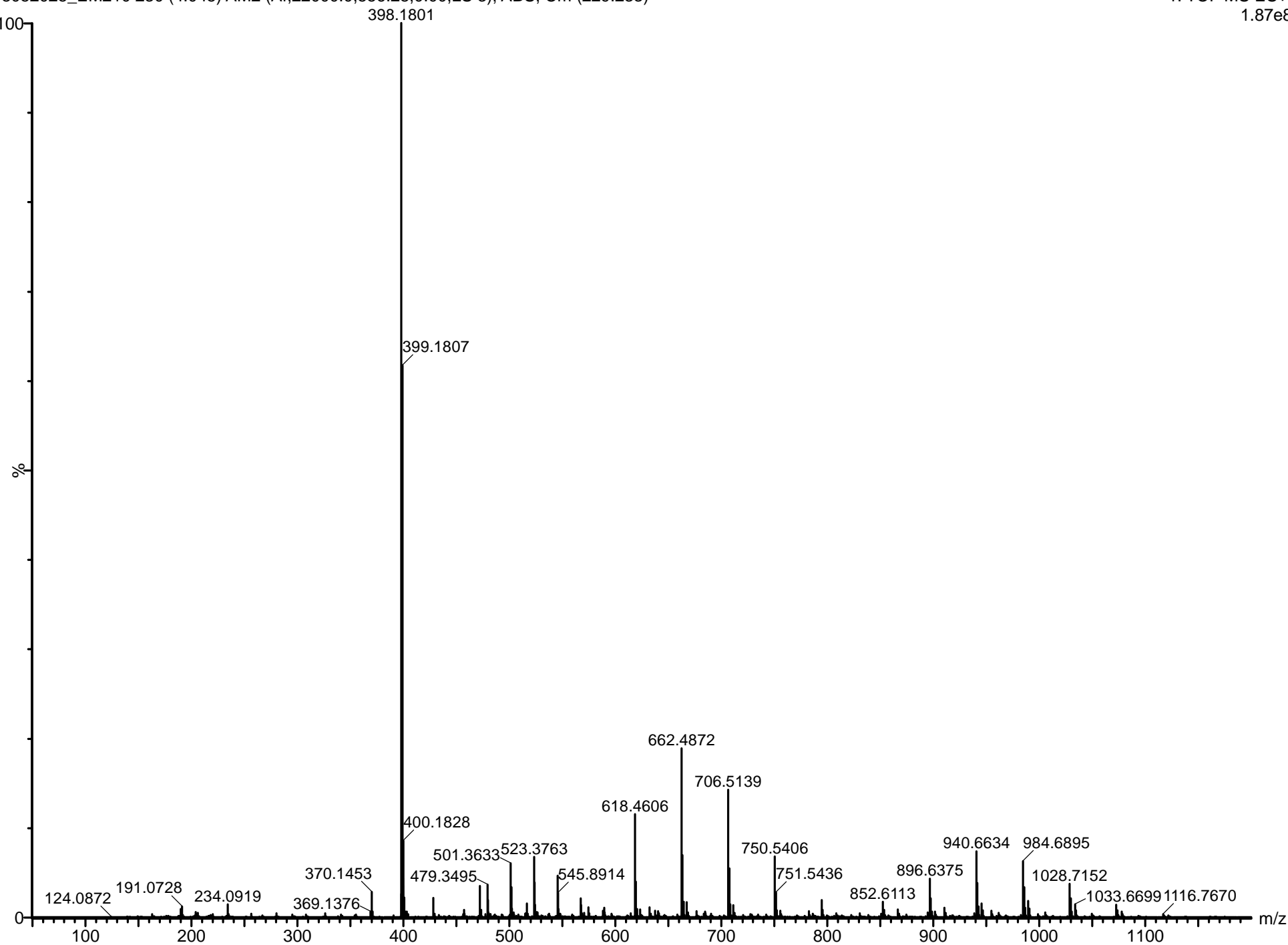

Supplement: S1 Data — Electrospray ionisation time of flight mass spectrometry (ESI-TOF MS, positive mode) spectra of the dengue cohort and ESI-TOF at different retention times. The spectra display the relative abundance (%) of detected ions across the m/z range. Prominent peaks corresponding to major ionised species are indicated. Variation in spectral profiles between retention times reflects the differences in compound composition and ionisation patterns within the sample. Data were acquired under identical instrumental conditions and are presented as representative scans. (ZIP) [file pntd.0014327.s003.zip › EM COMPLETE SAMPLES SPECTRUM/EM210 SPECTRUM RT 4.045.pdf]

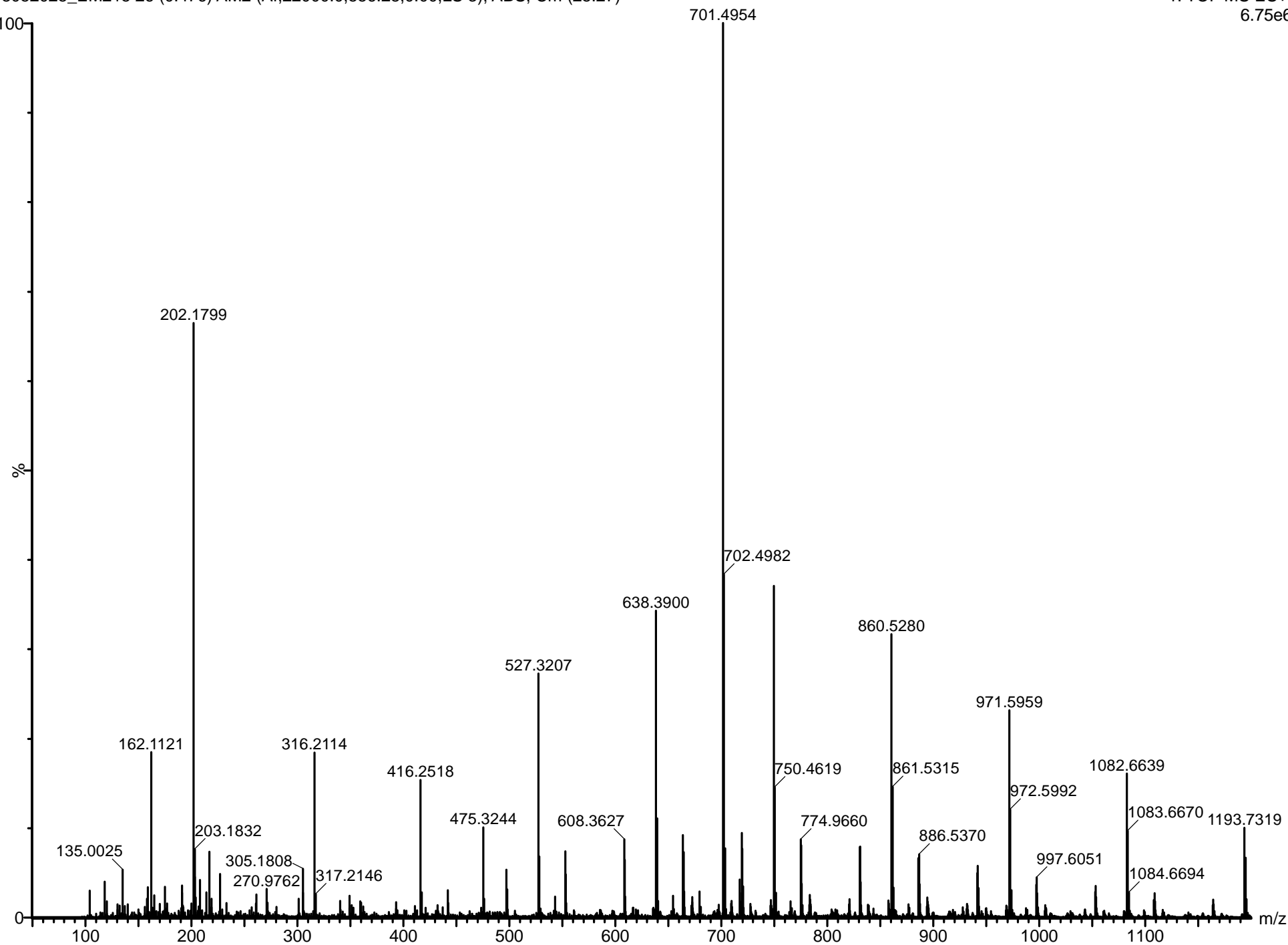

Supplement: S1 Data — Electrospray ionisation time of flight mass spectrometry (ESI-TOF MS, positive mode) spectra of the dengue cohort and ESI-TOF at different retention times. The spectra display the relative abundance (%) of detected ions across the m/z range. Prominent peaks corresponding to major ionised species are indicated. Variation in spectral profiles between retention times reflects the differences in compound composition and ionisation patterns within the sample. Data were acquired under identical instrumental conditions and are presented as representative scans. (ZIP) [file pntd.0014327.s003.zip › EM COMPLETE SAMPLES SPECTRUM/EM215 SPECTRUM RT 0.476.pdf]

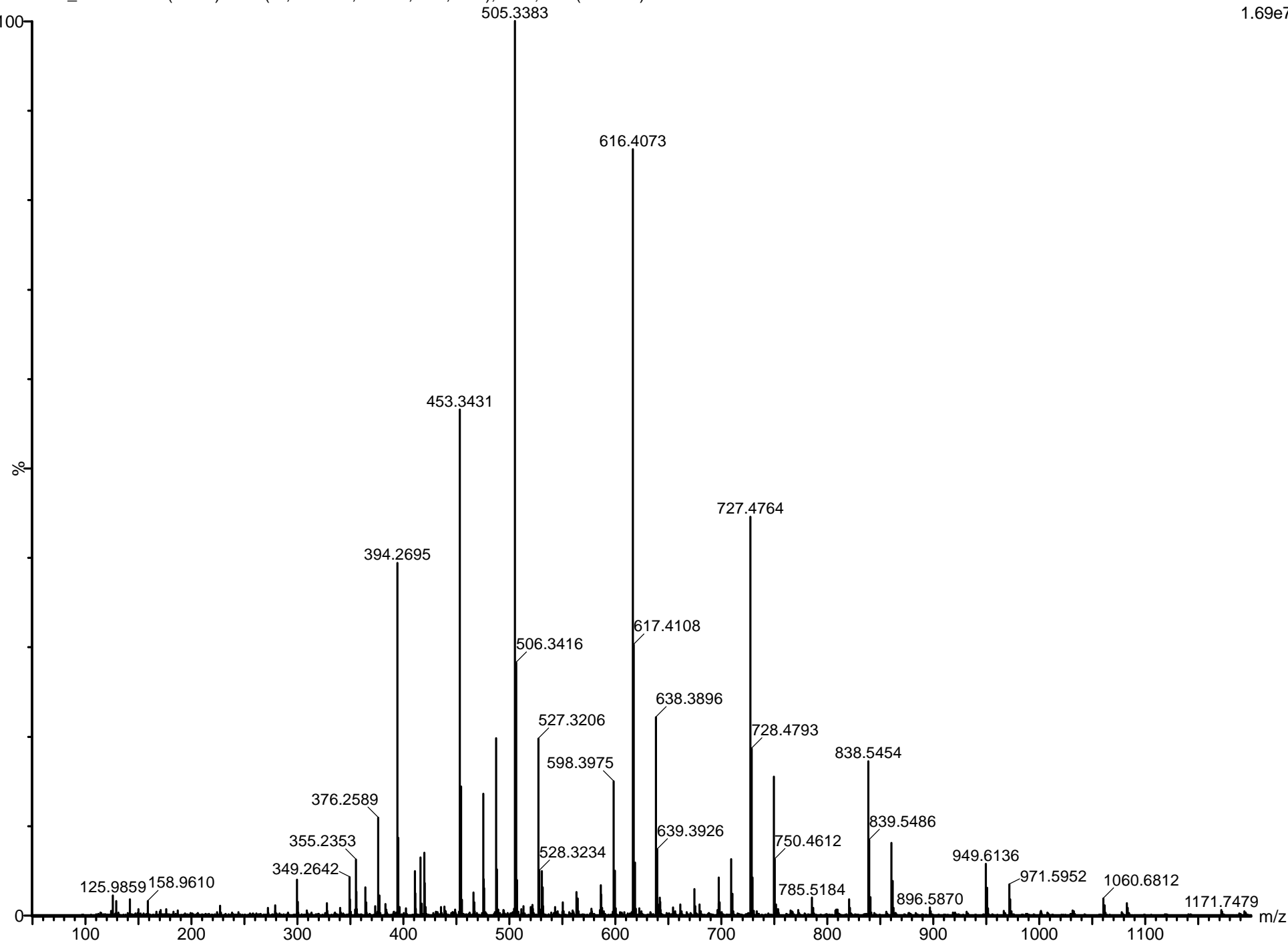

Supplement: S1 Data — Electrospray ionisation time of flight mass spectrometry (ESI-TOF MS, positive mode) spectra of the dengue cohort and ESI-TOF at different retention times. The spectra display the relative abundance (%) of detected ions across the m/z range. Prominent peaks corresponding to major ionised species are indicated. Variation in spectral profiles between retention times reflects the differences in compound composition and ionisation patterns within the sample. Data were acquired under identical instrumental conditions and are presented as representative scans. (ZIP) [file pntd.0014327.s003.zip › EM COMPLETE SAMPLES SPECTRUM/EM215 SPECTRUM RT 2.075.pdf]

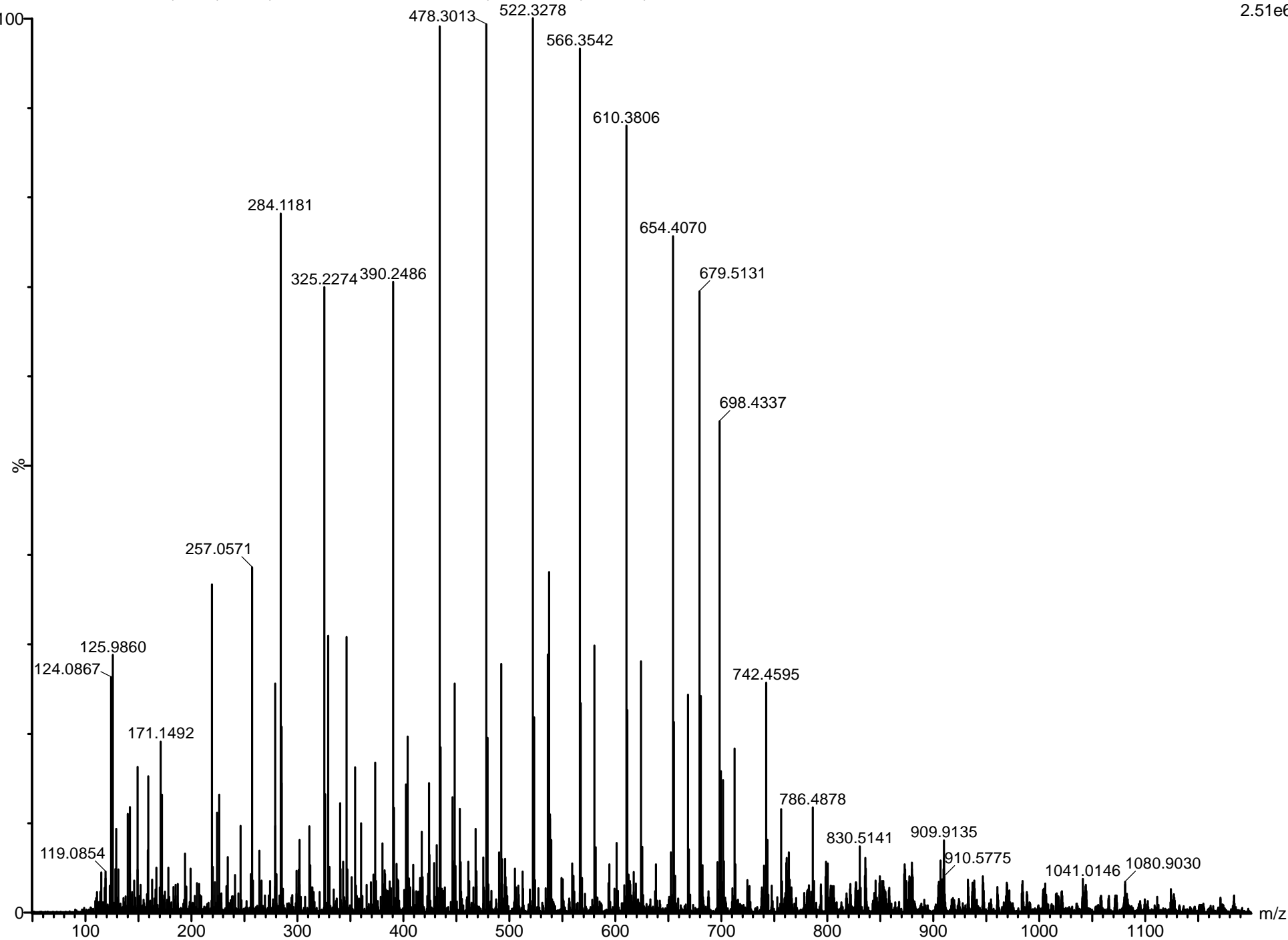

Supplement: S1 Data — Electrospray ionisation time of flight mass spectrometry (ESI-TOF MS, positive mode) spectra of the dengue cohort and ESI-TOF at different retention times. The spectra display the relative abundance (%) of detected ions across the m/z range. Prominent peaks corresponding to major ionised species are indicated. Variation in spectral profiles between retention times reflects the differences in compound composition and ionisation patterns within the sample. Data were acquired under identical instrumental conditions and are presented as representative scans. (ZIP) [file pntd.0014327.s003.zip › EM COMPLETE SAMPLES SPECTRUM/EM215 SPECTRUM RT 2.548.pdf]

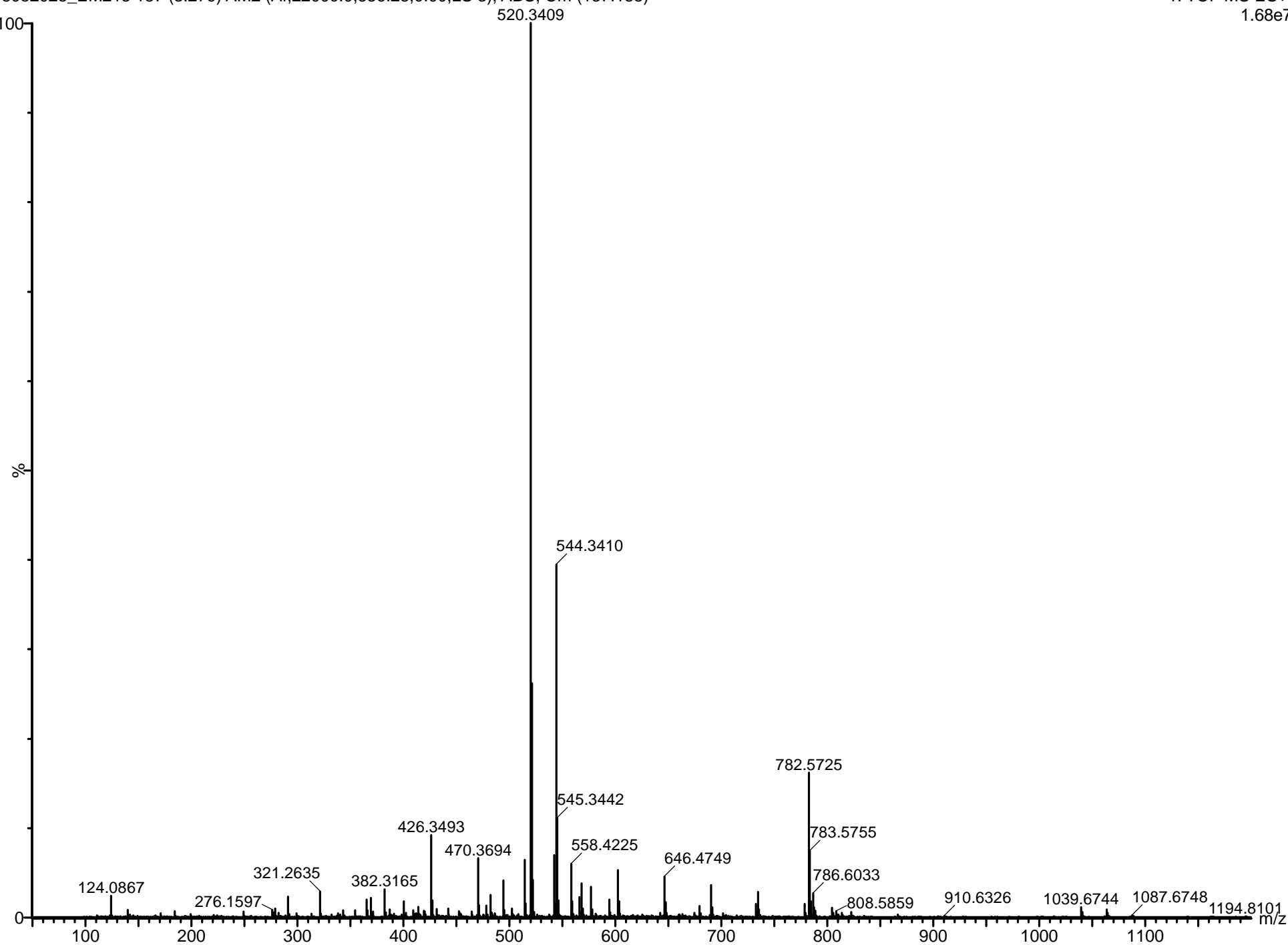

Supplement: S1 Data — Electrospray ionisation time of flight mass spectrometry (ESI-TOF MS, positive mode) spectra of the dengue cohort and ESI-TOF at different retention times. The spectra display the relative abundance (%) of detected ions across the m/z range. Prominent peaks corresponding to major ionised species are indicated. Variation in spectral profiles between retention times reflects the differences in compound composition and ionisation patterns within the sample. Data were acquired under identical instrumental conditions and are presented as representative scans. (ZIP) [file pntd.0014327.s003.zip › EM COMPLETE SAMPLES SPECTRUM/EM215 SPECTRUM RT 3.279.pdf]

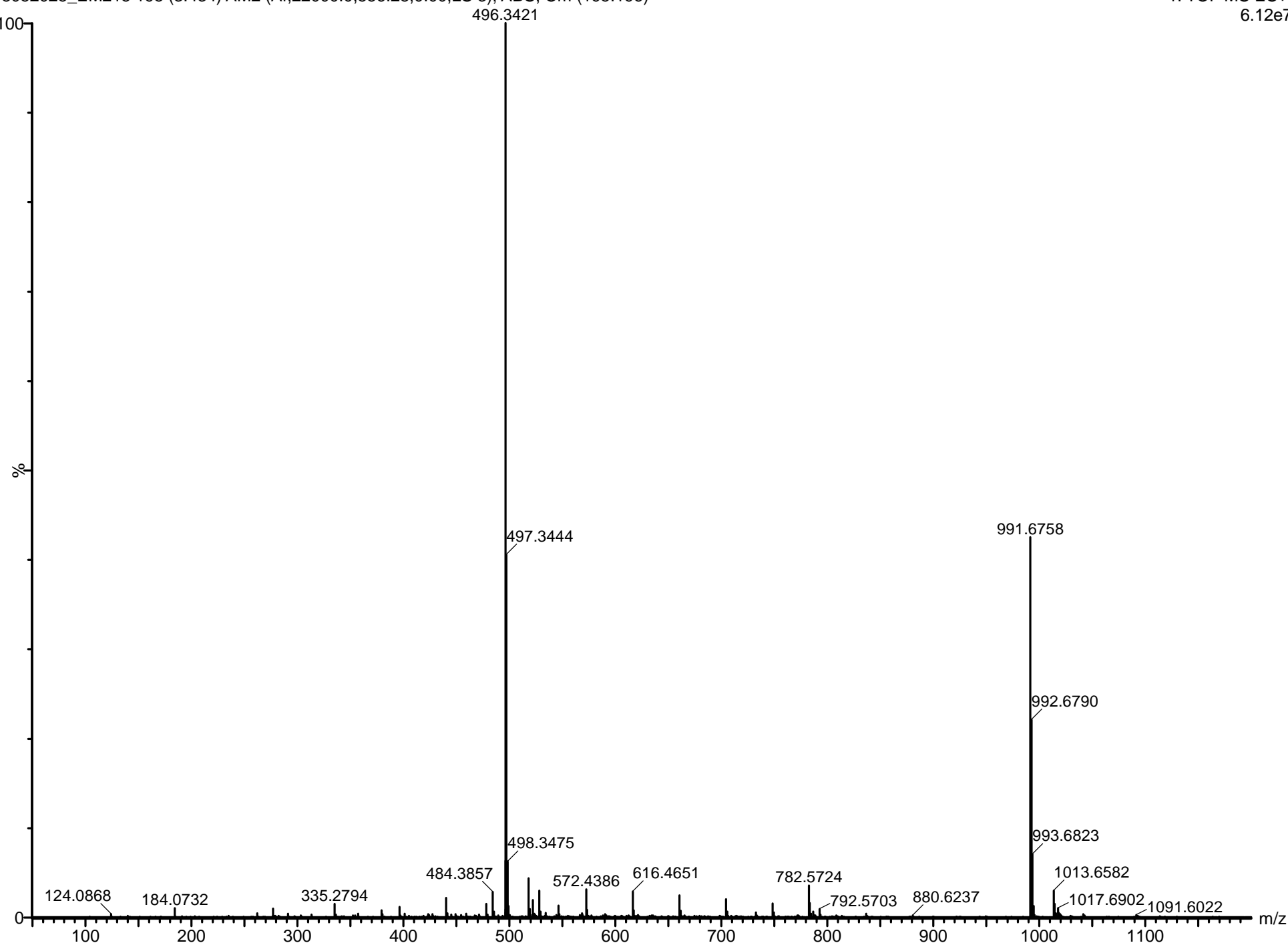

Supplement: S1 Data — Electrospray ionisation time of flight mass spectrometry (ESI-TOF MS, positive mode) spectra of the dengue cohort and ESI-TOF at different retention times. The spectra display the relative abundance (%) of detected ions across the m/z range. Prominent peaks corresponding to major ionised species are indicated. Variation in spectral profiles between retention times reflects the differences in compound composition and ionisation patterns within the sample. Data were acquired under identical instrumental conditions and are presented as representative scans. (ZIP) [file pntd.0014327.s003.zip › EM COMPLETE SAMPLES SPECTRUM/EM215 SPECTRUM RT 3.434.pdf]

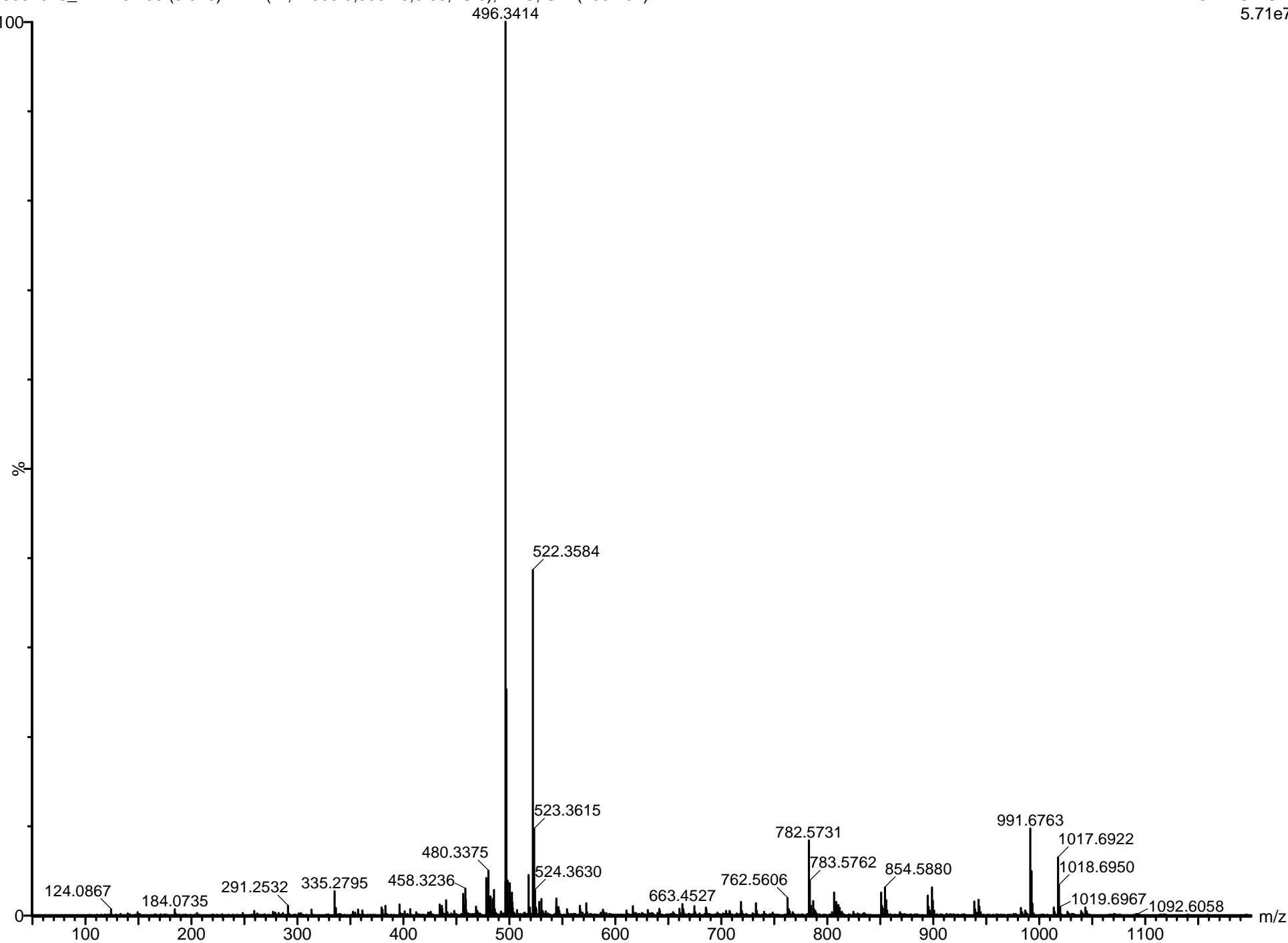

Supplement: S1 Data — Electrospray ionisation time of flight mass spectrometry (ESI-TOF MS, positive mode) spectra of the dengue cohort and ESI-TOF at different retention times. The spectra display the relative abundance (%) of detected ions across the m/z range. Prominent peaks corresponding to major ionised species are indicated. Variation in spectral profiles between retention times reflects the differences in compound composition and ionisation patterns within the sample. Data were acquired under identical instrumental conditions and are presented as representative scans. (ZIP) [file pntd.0014327.s003.zip › EM COMPLETE SAMPLES SPECTRUM/EM215 SPECTRUM RT 3.518.pdf]

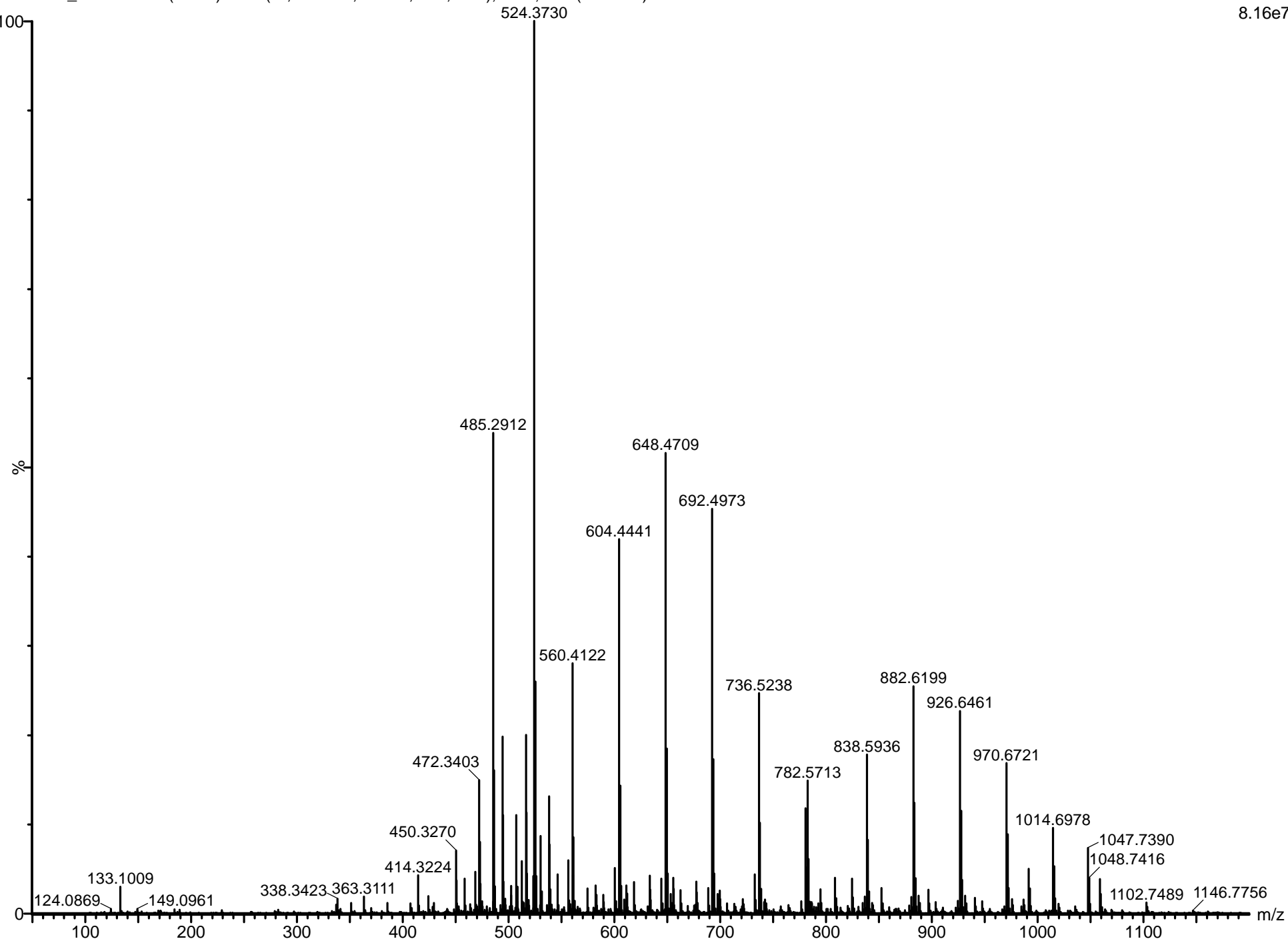

Supplement: S1 Data — Electrospray ionisation time of flight mass spectrometry (ESI-TOF MS, positive mode) spectra of the dengue cohort and ESI-TOF at different retention times. The spectra display the relative abundance (%) of detected ions across the m/z range. Prominent peaks corresponding to major ionised species are indicated. Variation in spectral profiles between retention times reflects the differences in compound composition and ionisation patterns within the sample. Data were acquired under identical instrumental conditions and are presented as representative scans. (ZIP) [file pntd.0014327.s003.zip › EM COMPLETE SAMPLES SPECTRUM/EM215 SPECTRUM RT 3.823.pdf]

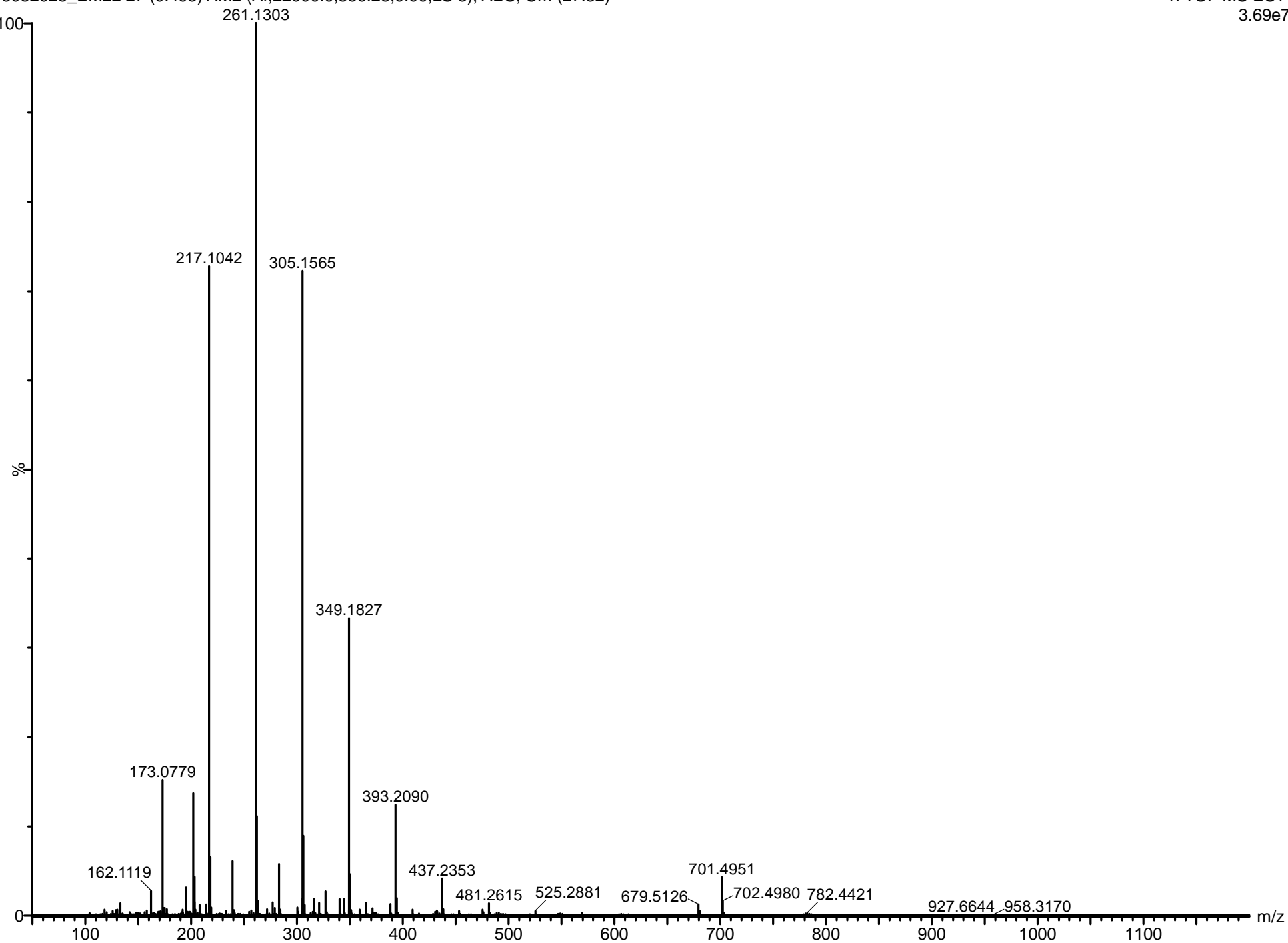

Supplement: S1 Data — Electrospray ionisation time of flight mass spectrometry (ESI-TOF MS, positive mode) spectra of the dengue cohort and ESI-TOF at different retention times. The spectra display the relative abundance (%) of detected ions across the m/z range. Prominent peaks corresponding to major ionised species are indicated. Variation in spectral profiles between retention times reflects the differences in compound composition and ionisation patterns within the sample. Data were acquired under identical instrumental conditions and are presented as representative scans. (ZIP) [file pntd.0014327.s003.zip › EM COMPLETE SAMPLES SPECTRUM/EM22 SPECTRUM RT 0.493.pdf]

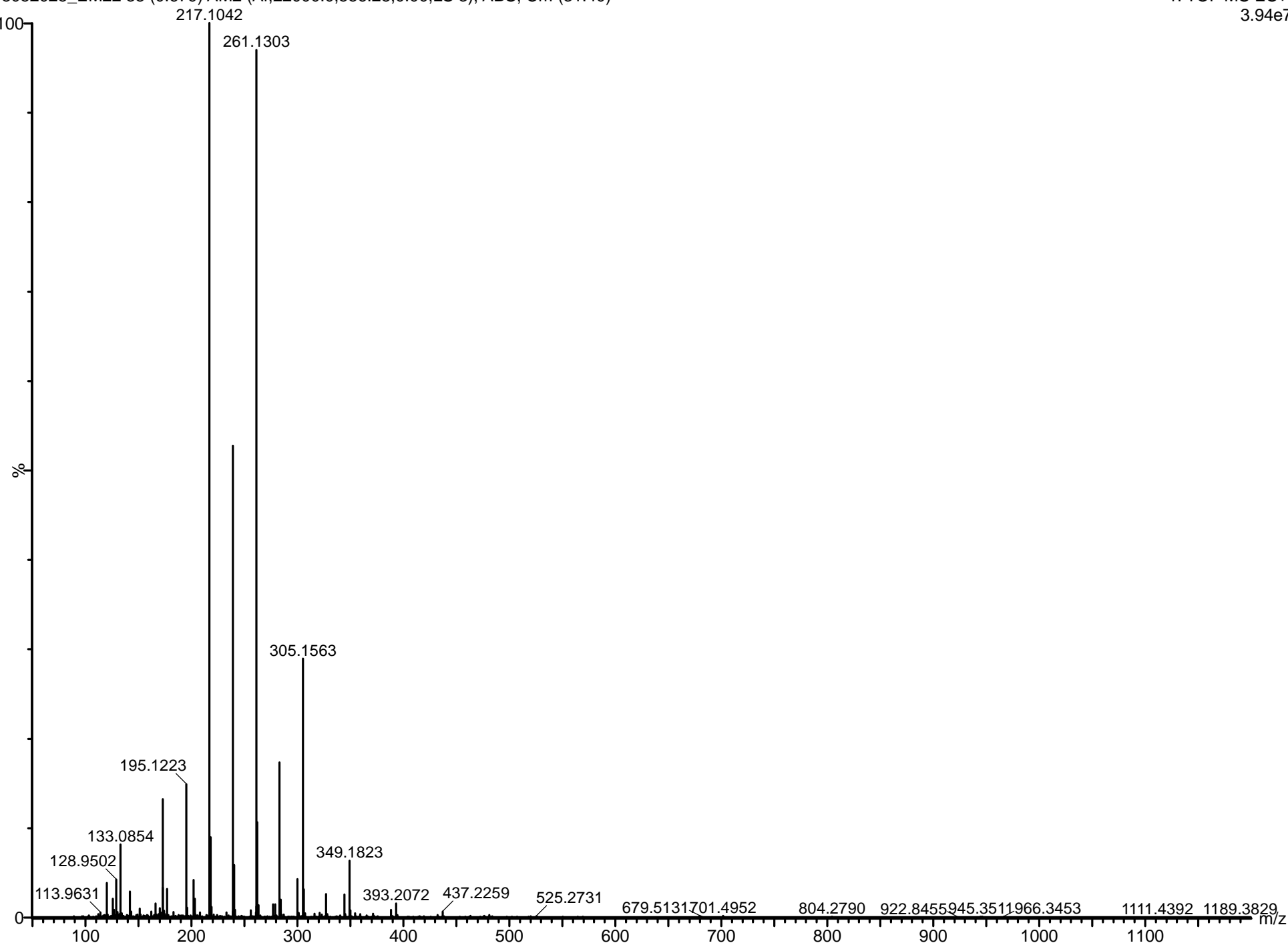

Supplement: S1 Data — Electrospray ionisation time of flight mass spectrometry (ESI-TOF MS, positive mode) spectra of the dengue cohort and ESI-TOF at different retention times. The spectra display the relative abundance (%) of detected ions across the m/z range. Prominent peaks corresponding to major ionised species are indicated. Variation in spectral profiles between retention times reflects the differences in compound composition and ionisation patterns within the sample. Data were acquired under identical instrumental conditions and are presented as representative scans. (ZIP) [file pntd.0014327.s003.zip › EM COMPLETE SAMPLES SPECTRUM/EM22 SPECTRUM RT 0.679.pdf]

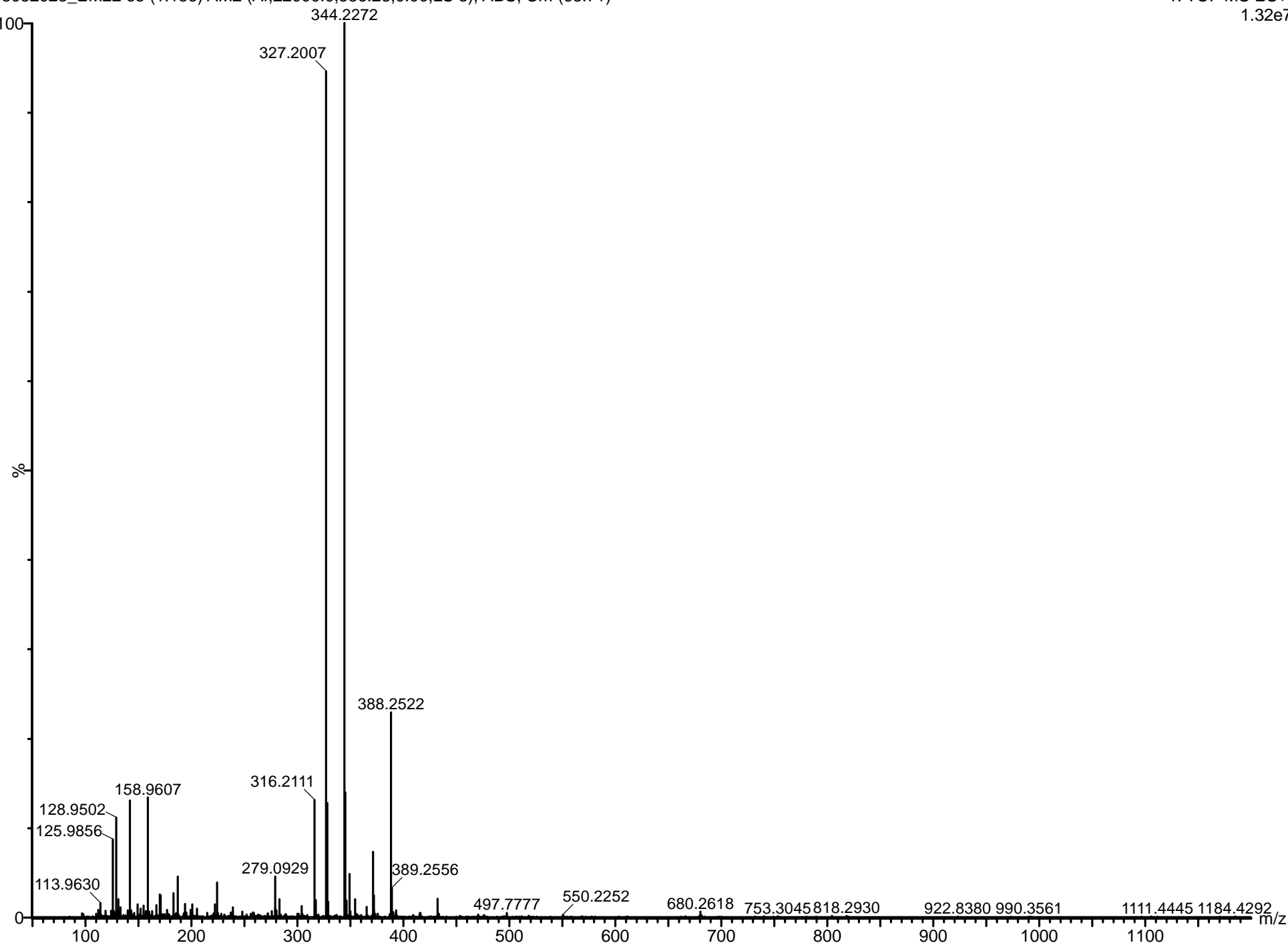

Supplement: S1 Data — Electrospray ionisation time of flight mass spectrometry (ESI-TOF MS, positive mode) spectra of the dengue cohort and ESI-TOF at different retention times. The spectra display the relative abundance (%) of detected ions across the m/z range. Prominent peaks corresponding to major ionised species are indicated. Variation in spectral profiles between retention times reflects the differences in compound composition and ionisation patterns within the sample. Data were acquired under identical instrumental conditions and are presented as representative scans. (ZIP) [file pntd.0014327.s003.zip › EM COMPLETE SAMPLES SPECTRUM/EM22 SPECTRUM RT 1.156.pdf]

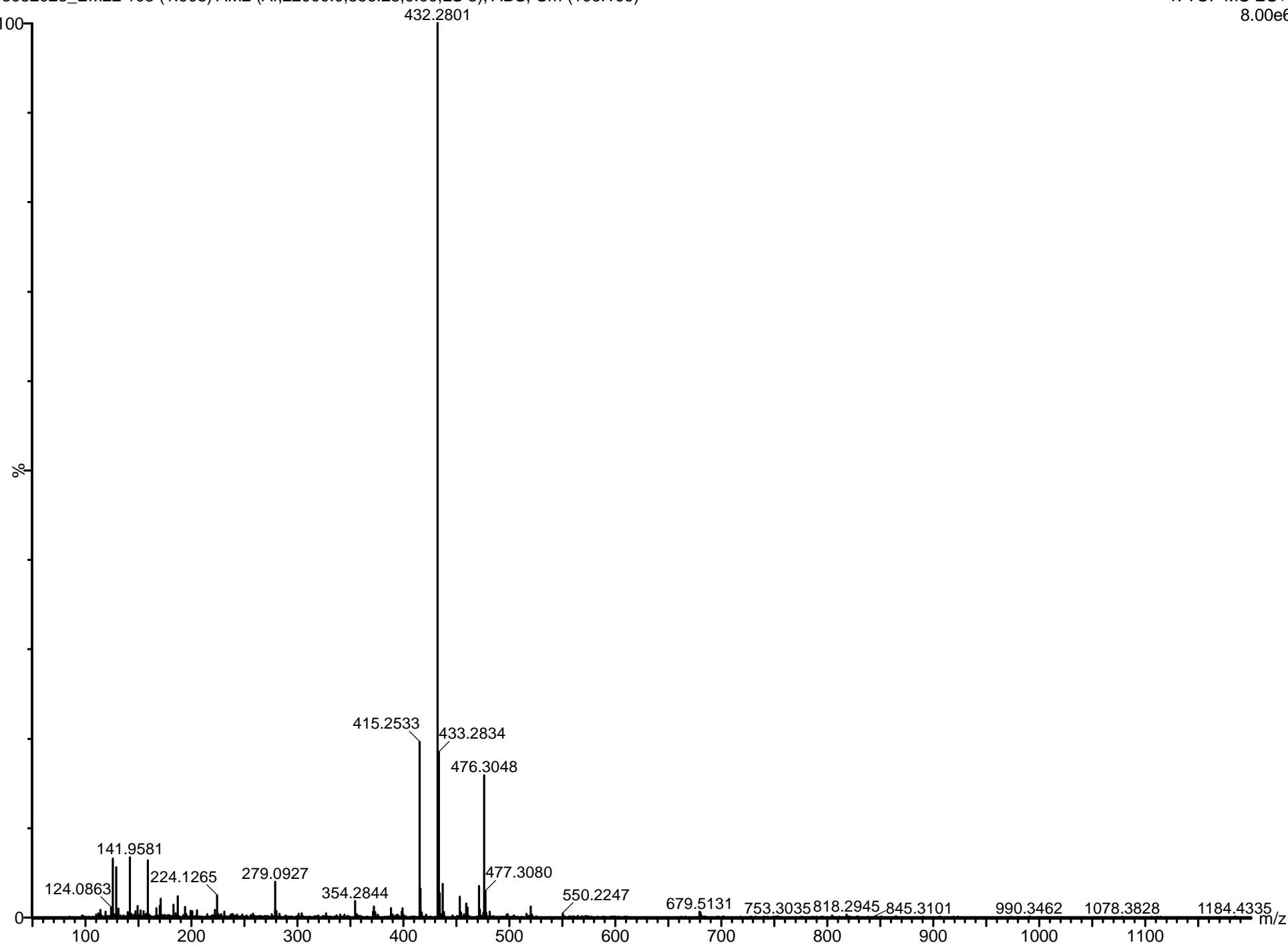

Supplement: S1 Data — Electrospray ionisation time of flight mass spectrometry (ESI-TOF MS, positive mode) spectra of the dengue cohort and ESI-TOF at different retention times. The spectra display the relative abundance (%) of detected ions across the m/z range. Prominent peaks corresponding to major ionised species are indicated. Variation in spectral profiles between retention times reflects the differences in compound composition and ionisation patterns within the sample. Data were acquired under identical instrumental conditions and are presented as representative scans. (ZIP) [file pntd.0014327.s003.zip › EM COMPLETE SAMPLES SPECTRUM/EM22 SPECTRUM RT 1.903.pdf]

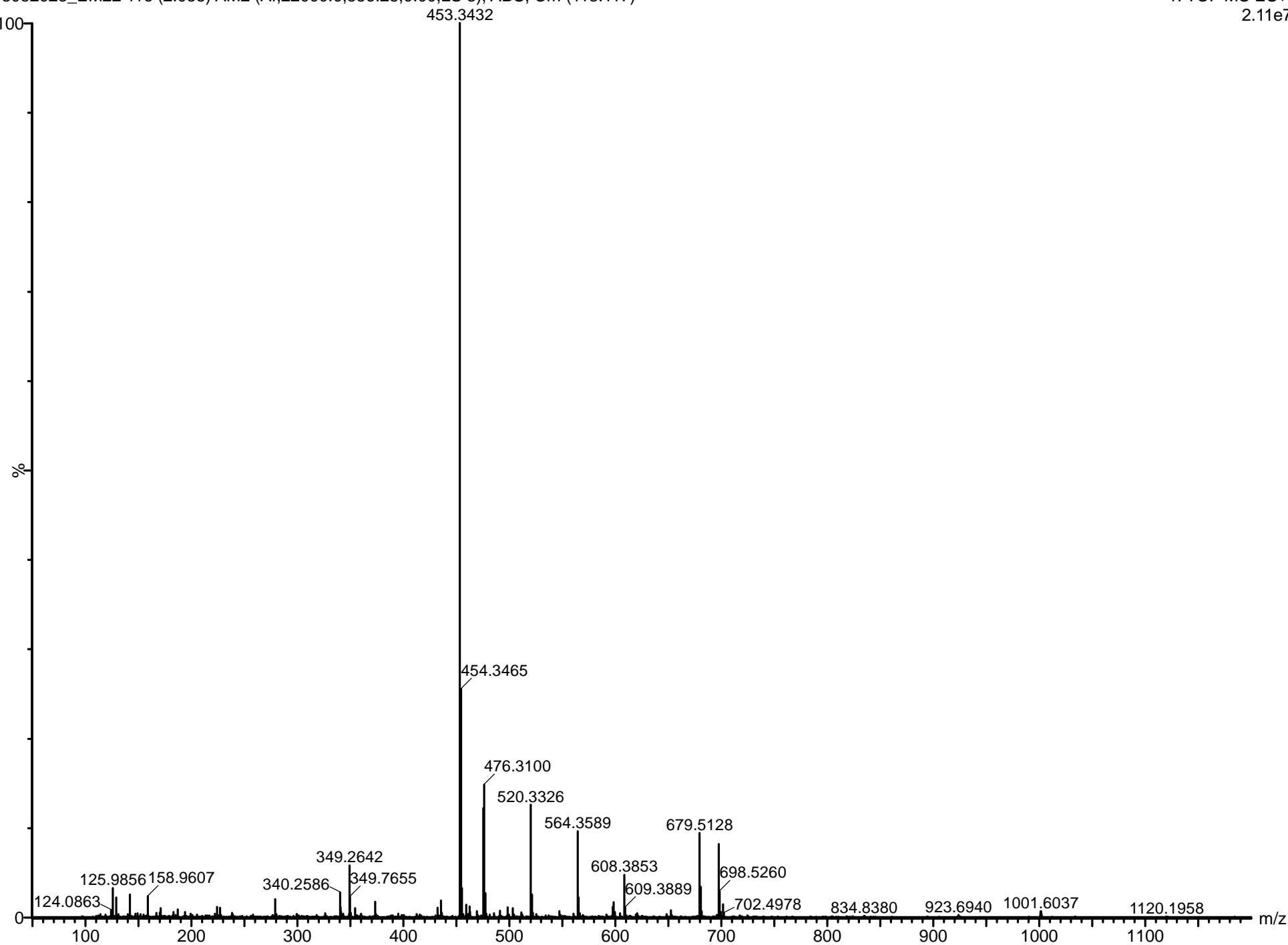

Supplement: S1 Data — Electrospray ionisation time of flight mass spectrometry (ESI-TOF MS, positive mode) spectra of the dengue cohort and ESI-TOF at different retention times. The spectra display the relative abundance (%) of detected ions across the m/z range. Prominent peaks corresponding to major ionised species are indicated. Variation in spectral profiles between retention times reflects the differences in compound composition and ionisation patterns within the sample. Data were acquired under identical instrumental conditions and are presented as representative scans. (ZIP) [file pntd.0014327.s003.zip › EM COMPLETE SAMPLES SPECTRUM/EM22 SPECTRUM RT 2.058.pdf]

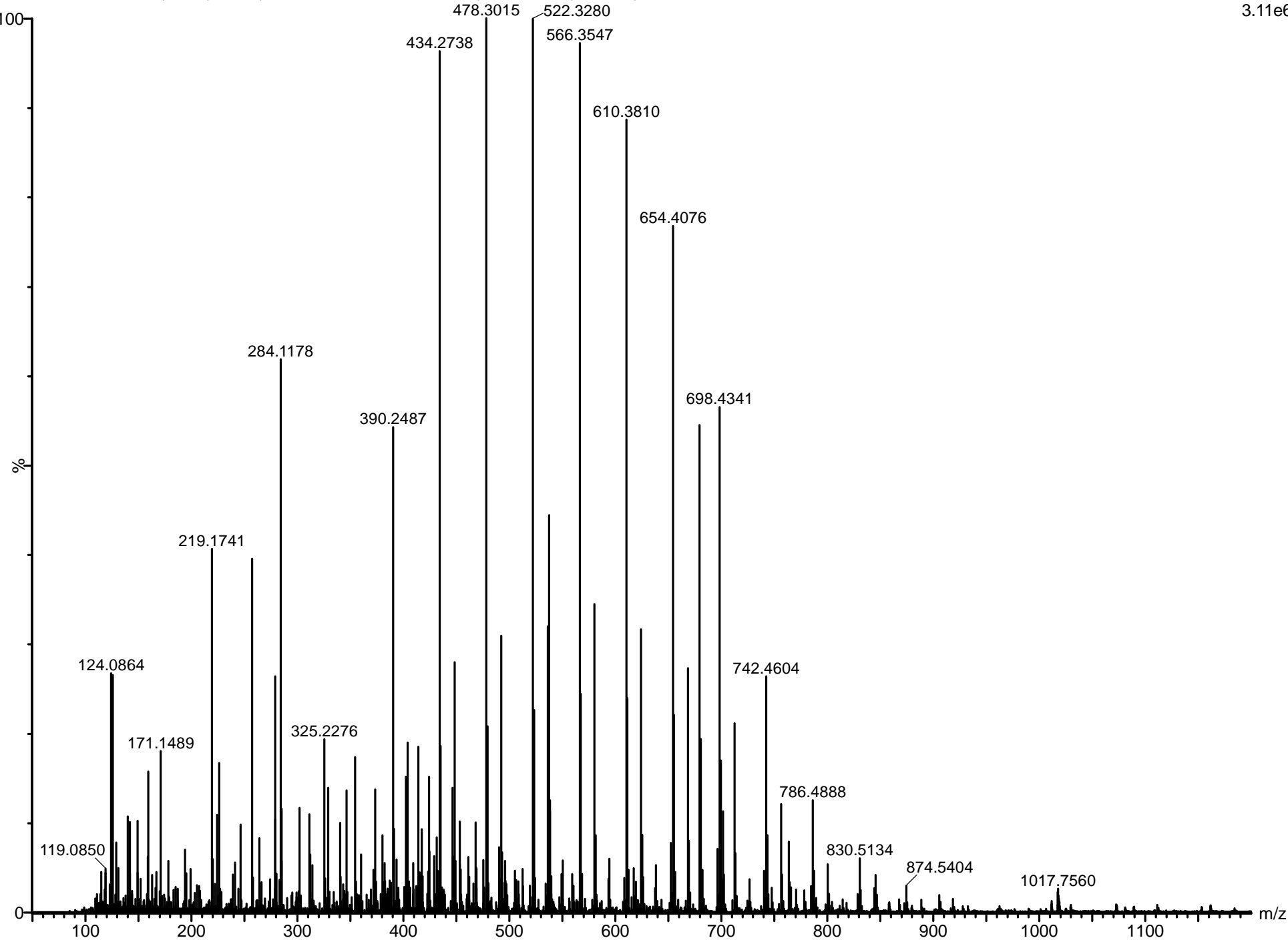

Supplement: S1 Data — Electrospray ionisation time of flight mass spectrometry (ESI-TOF MS, positive mode) spectra of the dengue cohort and ESI-TOF at different retention times. The spectra display the relative abundance (%) of detected ions across the m/z range. Prominent peaks corresponding to major ionised species are indicated. Variation in spectral profiles between retention times reflects the differences in compound composition and ionisation patterns within the sample. Data were acquired under identical instrumental conditions and are presented as representative scans. (ZIP) [file pntd.0014327.s003.zip › EM COMPLETE SAMPLES SPECTRUM/EM22 SPECTRUM RT 2.565.pdf]

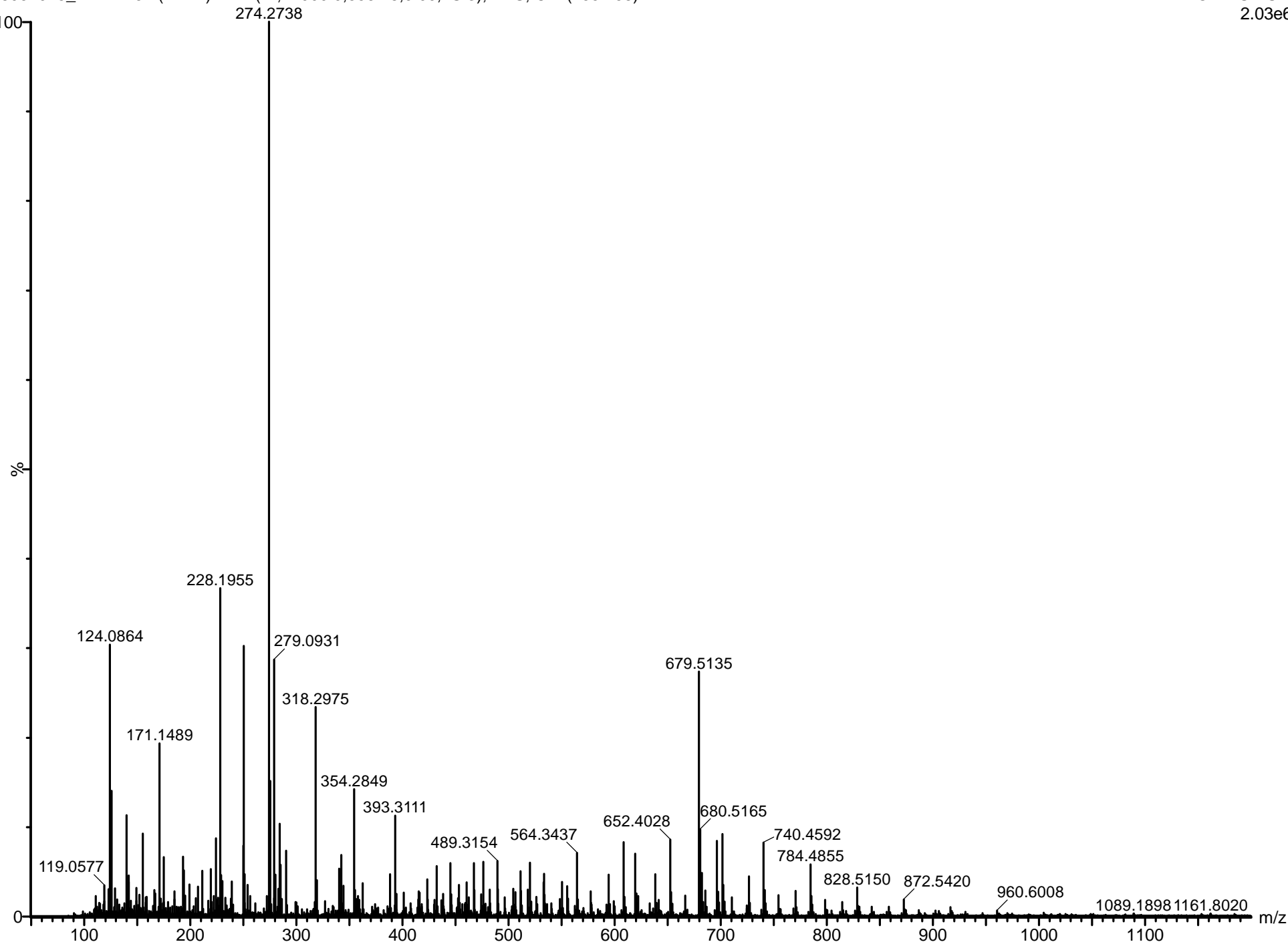

Supplement: S1 Data — Electrospray ionisation time of flight mass spectrometry (ESI-TOF MS, positive mode) spectra of the dengue cohort and ESI-TOF at different retention times. The spectra display the relative abundance (%) of detected ions across the m/z range. Prominent peaks corresponding to major ionised species are indicated. Variation in spectral profiles between retention times reflects the differences in compound composition and ionisation patterns within the sample. Data were acquired under identical instrumental conditions and are presented as representative scans. (ZIP) [file pntd.0014327.s003.zip › EM COMPLETE SAMPLES SPECTRUM/EM22 SPECTRUM RT 2.771.pdf]

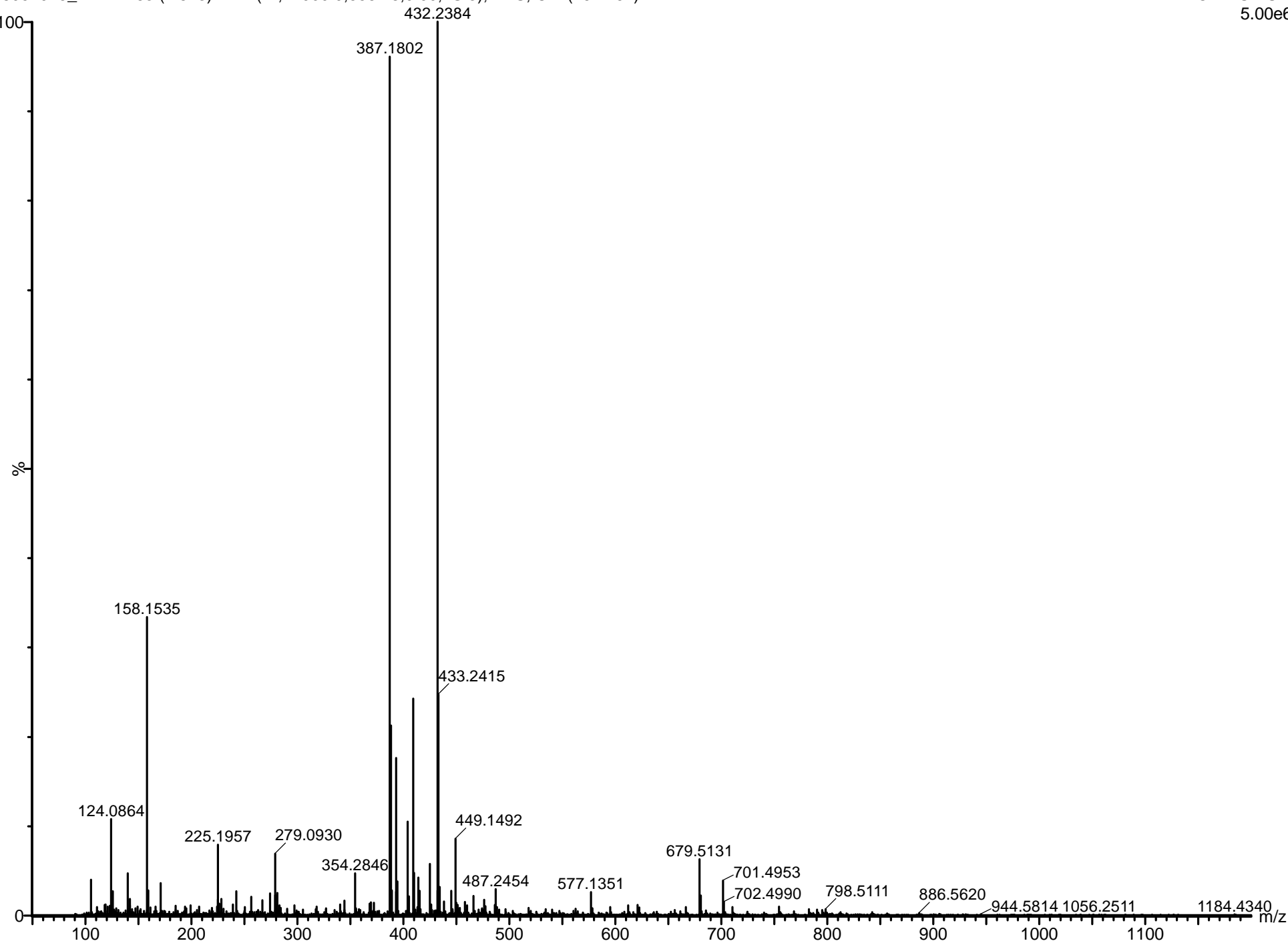

Supplement: S1 Data — Electrospray ionisation time of flight mass spectrometry (ESI-TOF MS, positive mode) spectra of the dengue cohort and ESI-TOF at different retention times. The spectra display the relative abundance (%) of detected ions across the m/z range. Prominent peaks corresponding to major ionised species are indicated. Variation in spectral profiles between retention times reflects the differences in compound composition and ionisation patterns within the sample. Data were acquired under identical instrumental conditions and are presented as representative scans. (ZIP) [file pntd.0014327.s003.zip › EM COMPLETE SAMPLES SPECTRUM/EM22 SPECTRUM RT 2.873.pdf]

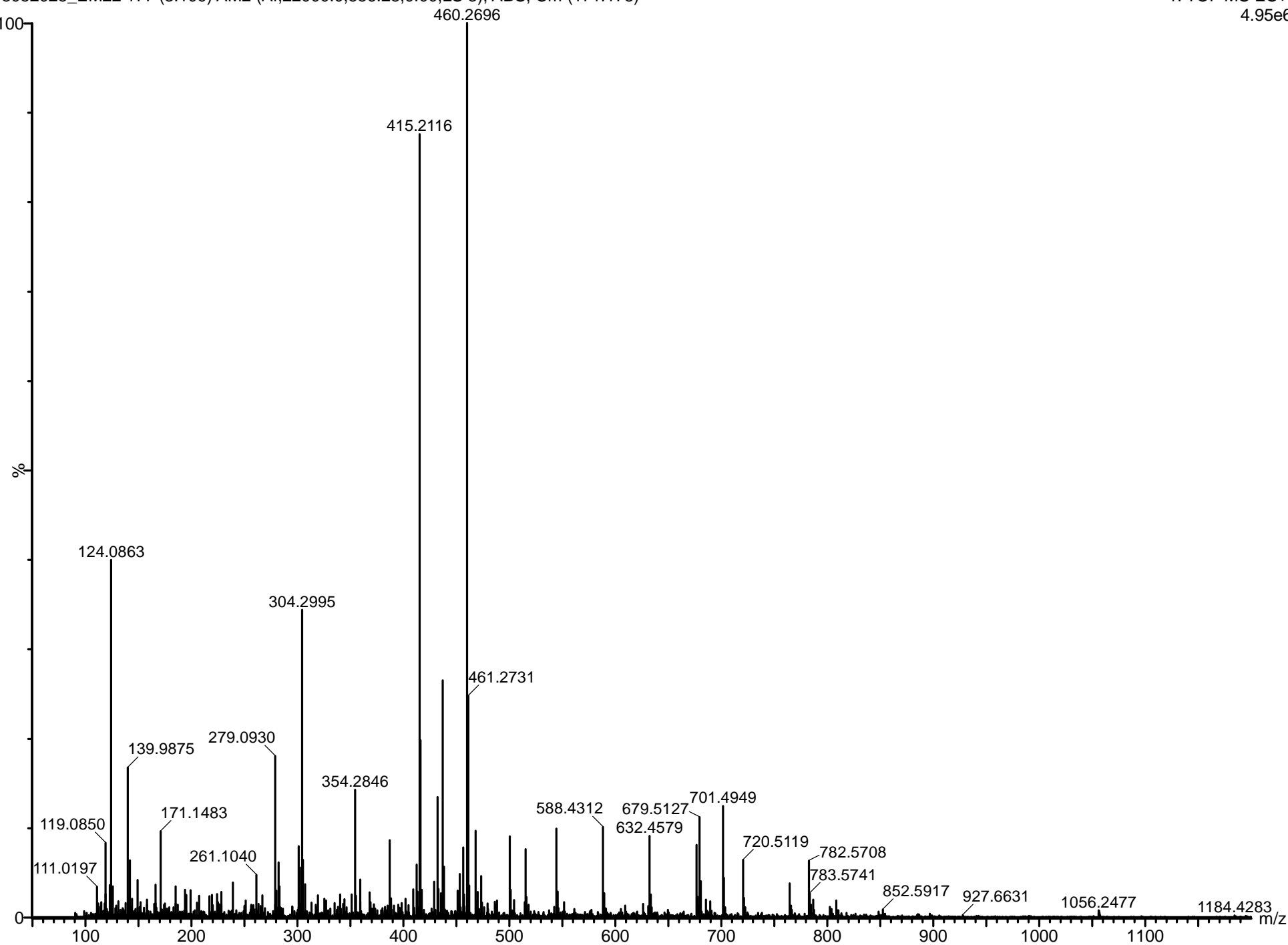

Supplement: S1 Data — Electrospray ionisation time of flight mass spectrometry (ESI-TOF MS, positive mode) spectra of the dengue cohort and ESI-TOF at different retention times. The spectra display the relative abundance (%) of detected ions across the m/z range. Prominent peaks corresponding to major ionised species are indicated. Variation in spectral profiles between retention times reflects the differences in compound composition and ionisation patterns within the sample. Data were acquired under identical instrumental conditions and are presented as representative scans. (ZIP) [file pntd.0014327.s003.zip › EM COMPLETE SAMPLES SPECTRUM/EM22 SPECTRUM RT 3.109.pdf]

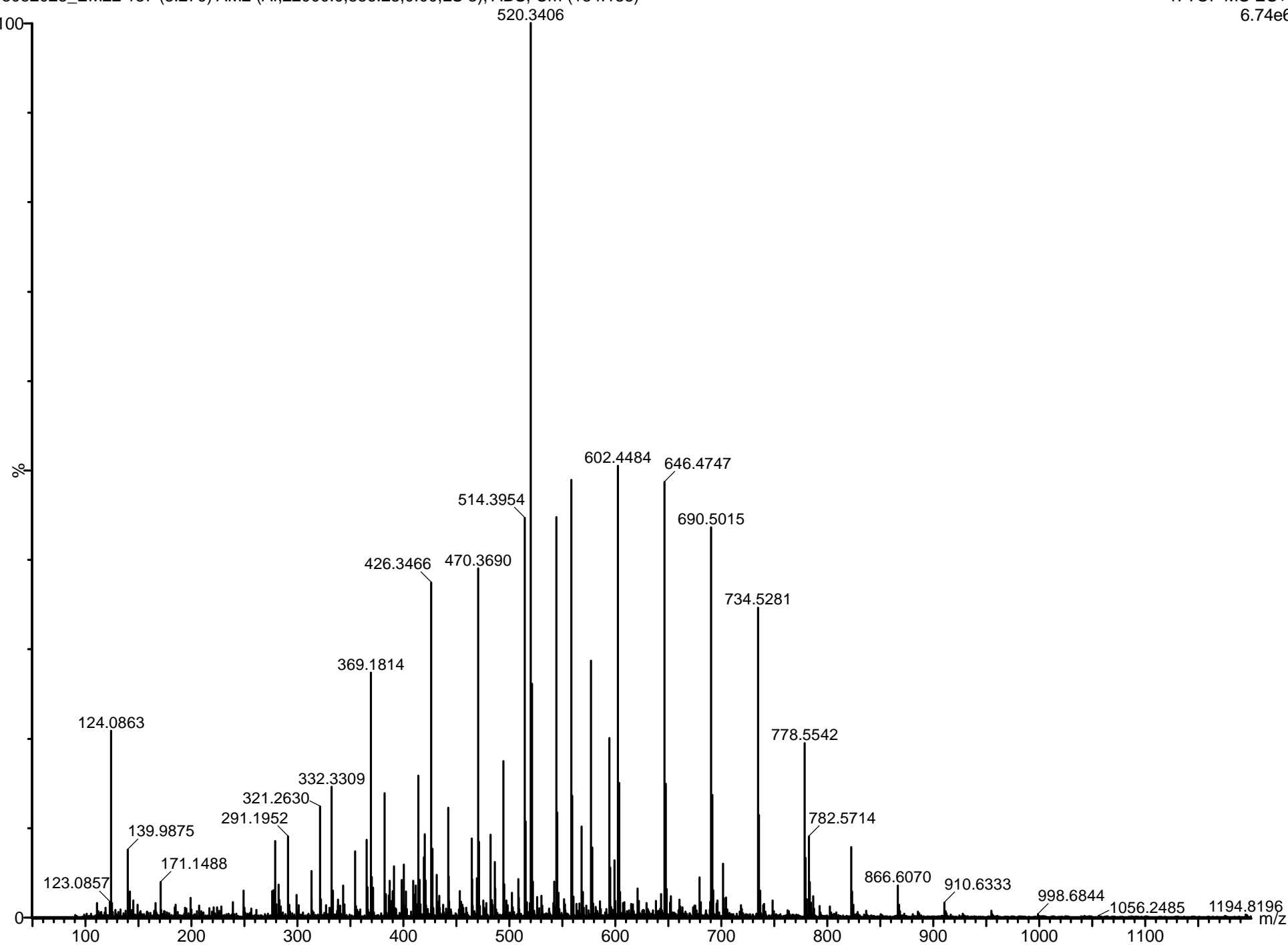

Supplement: S1 Data — Electrospray ionisation time of flight mass spectrometry (ESI-TOF MS, positive mode) spectra of the dengue cohort and ESI-TOF at different retention times. The spectra display the relative abundance (%) of detected ions across the m/z range. Prominent peaks corresponding to major ionised species are indicated. Variation in spectral profiles between retention times reflects the differences in compound composition and ionisation patterns within the sample. Data were acquired under identical instrumental conditions and are presented as representative scans. (ZIP) [file pntd.0014327.s003.zip › EM COMPLETE SAMPLES SPECTRUM/EM22 SPECTRUM RT 3.279.pdf]

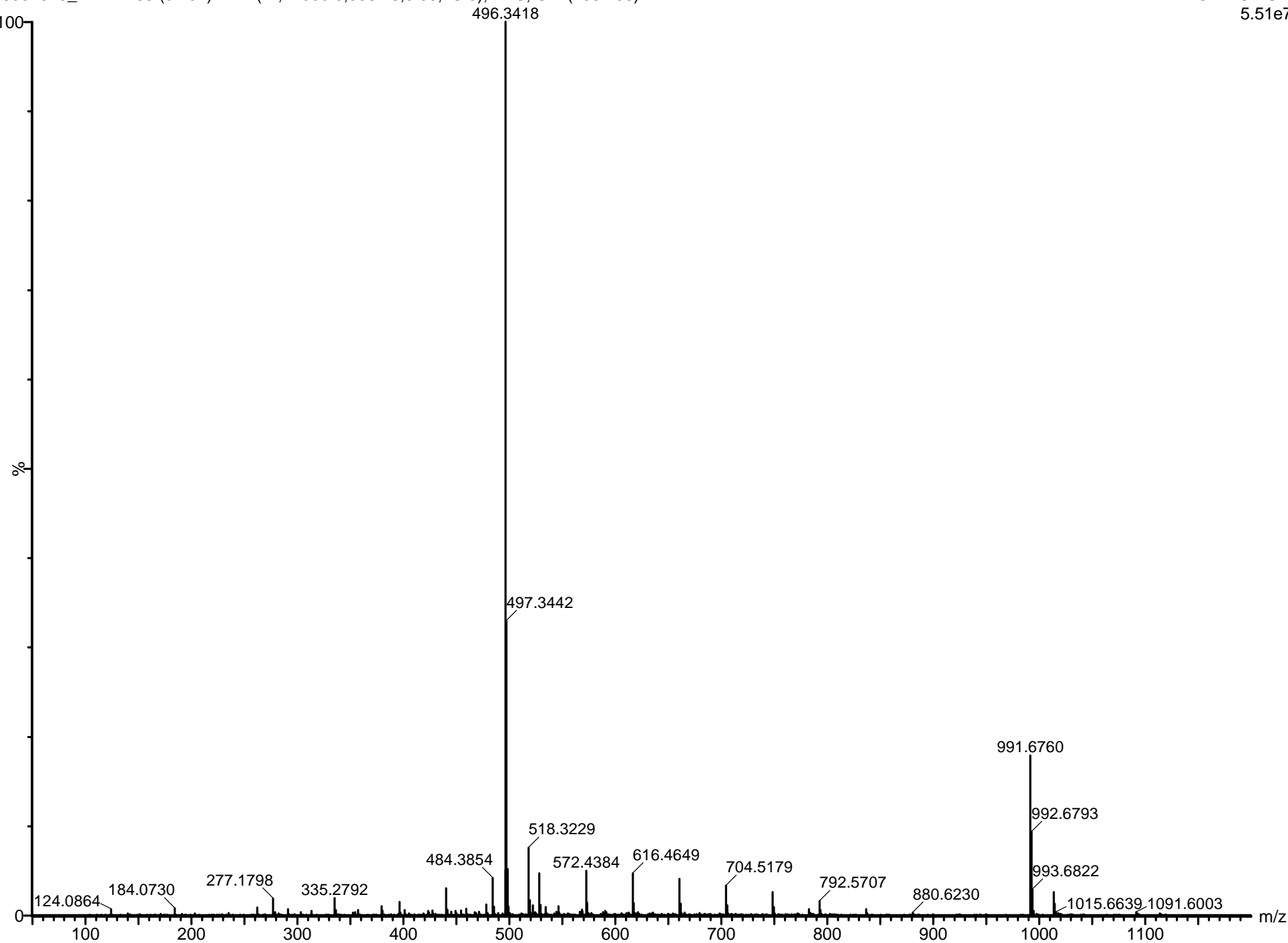

Supplement: S1 Data — Electrospray ionisation time of flight mass spectrometry (ESI-TOF MS, positive mode) spectra of the dengue cohort and ESI-TOF at different retention times. The spectra display the relative abundance (%) of detected ions across the m/z range. Prominent peaks corresponding to major ionised species are indicated. Variation in spectral profiles between retention times reflects the differences in compound composition and ionisation patterns within the sample. Data were acquired under identical instrumental conditions and are presented as representative scans. (ZIP) [file pntd.0014327.s003.zip › EM COMPLETE SAMPLES SPECTRUM/EM22 SPECTRUM RT 3.434.pdf]

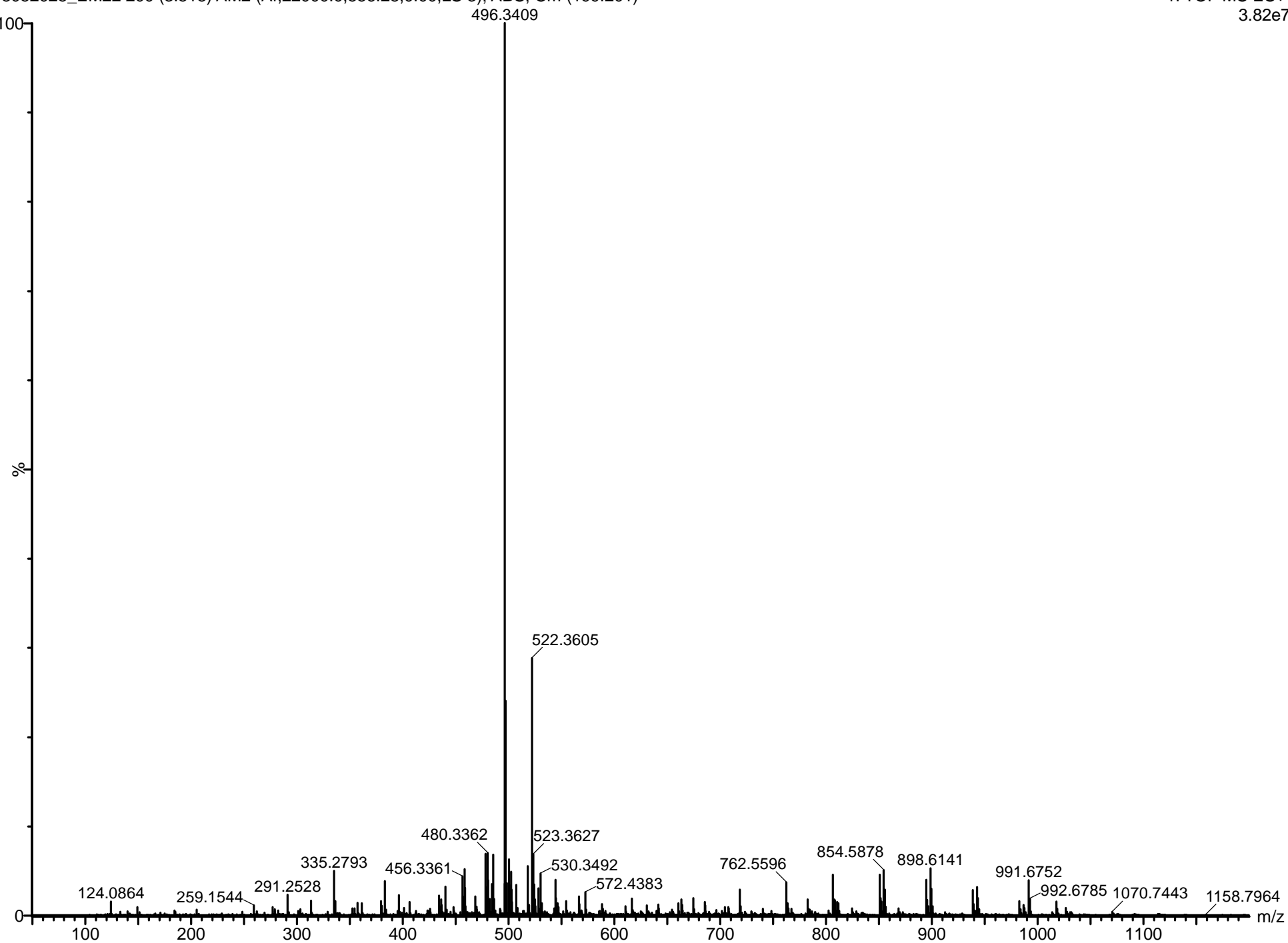

Supplement: S1 Data — Electrospray ionisation time of flight mass spectrometry (ESI-TOF MS, positive mode) spectra of the dengue cohort and ESI-TOF at different retention times. The spectra display the relative abundance (%) of detected ions across the m/z range. Prominent peaks corresponding to major ionised species are indicated. Variation in spectral profiles between retention times reflects the differences in compound composition and ionisation patterns within the sample. Data were acquired under identical instrumental conditions and are presented as representative scans. (ZIP) [file pntd.0014327.s003.zip › EM COMPLETE SAMPLES SPECTRUM/EM22 SPECTRUM RT 3.518.pdf]

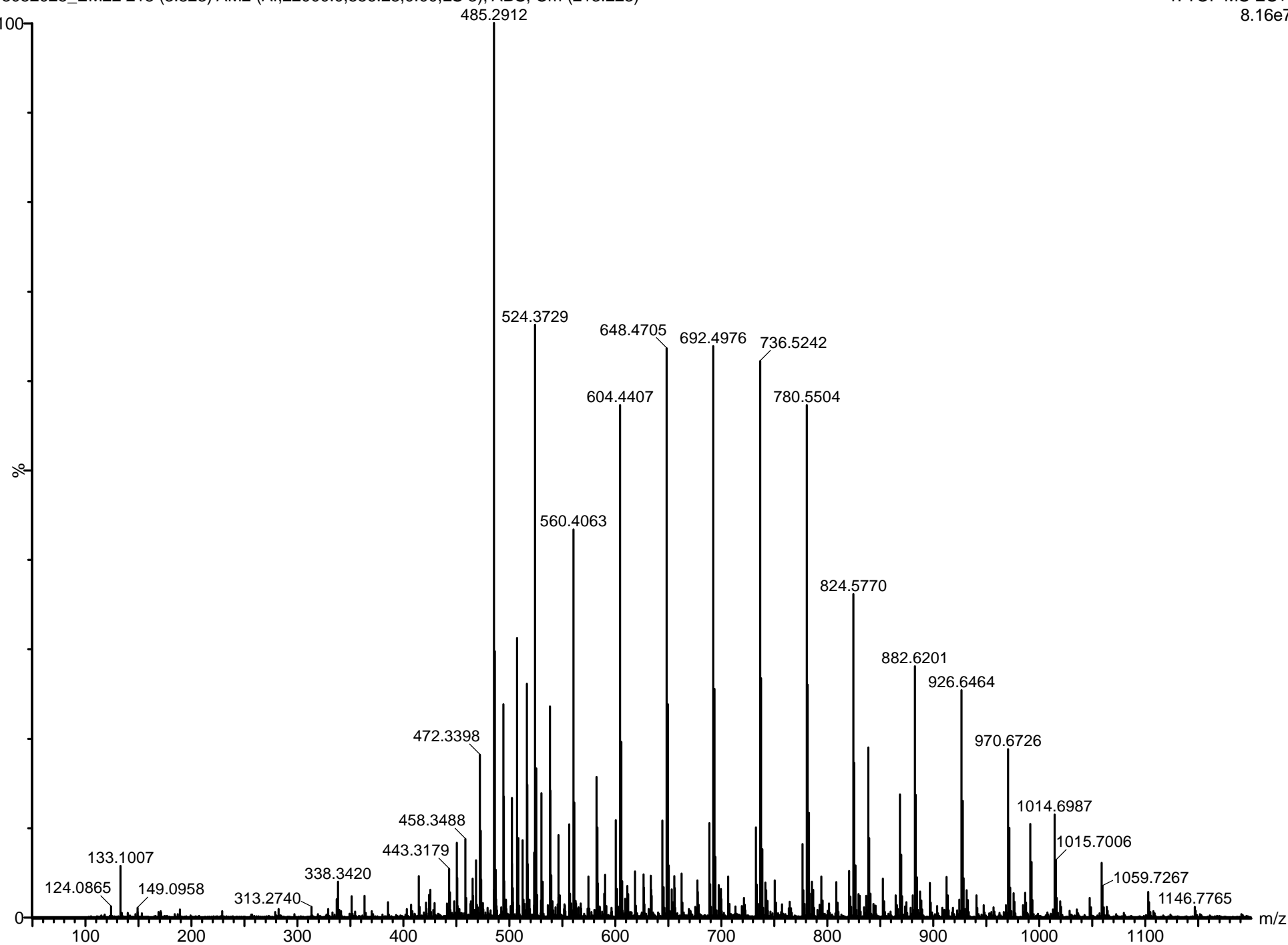

Supplement: S1 Data — Electrospray ionisation time of flight mass spectrometry (ESI-TOF MS, positive mode) spectra of the dengue cohort and ESI-TOF at different retention times. The spectra display the relative abundance (%) of detected ions across the m/z range. Prominent peaks corresponding to major ionised species are indicated. Variation in spectral profiles between retention times reflects the differences in compound composition and ionisation patterns within the sample. Data were acquired under identical instrumental conditions and are presented as representative scans. (ZIP) [file pntd.0014327.s003.zip › EM COMPLETE SAMPLES SPECTRUM/EM22 SPECTRUM RT 3.823.pdf]
