# Supplementary material for: Metabolomic atlas of dengue virus infection reveals distinct circulating bioactive lipid signatures
Source: PLoS Negl Trop Dis. 2026 May 12;20(5):e0014327. doi: 10.1371/journal.pntd.0014327 (PMC13189415; doi:10.1371/journal.pntd.0014327)
Supplement: S1 Data — Electrospray ionisation time of flight mass spectrometry (ESI-TOF MS, positive mode) spectra of the dengue cohort and ESI-TOF at different retention times. The spectra display the relative abundance (%) of detected ions across the m/z range. Prominent peaks corresponding to major ionised species are indicated. Variation in spectral profiles between retention times reflects the differences in compound composition and ionisation patterns within the sample. Data were acquired under identical instrumental conditions and are presented as representative scans. (ZIP) [file pntd.0014327.s003.zip › EM COMPLETE SAMPLES SPECTRUM/EM39 SPECTRUM RT 3.451.pdf]

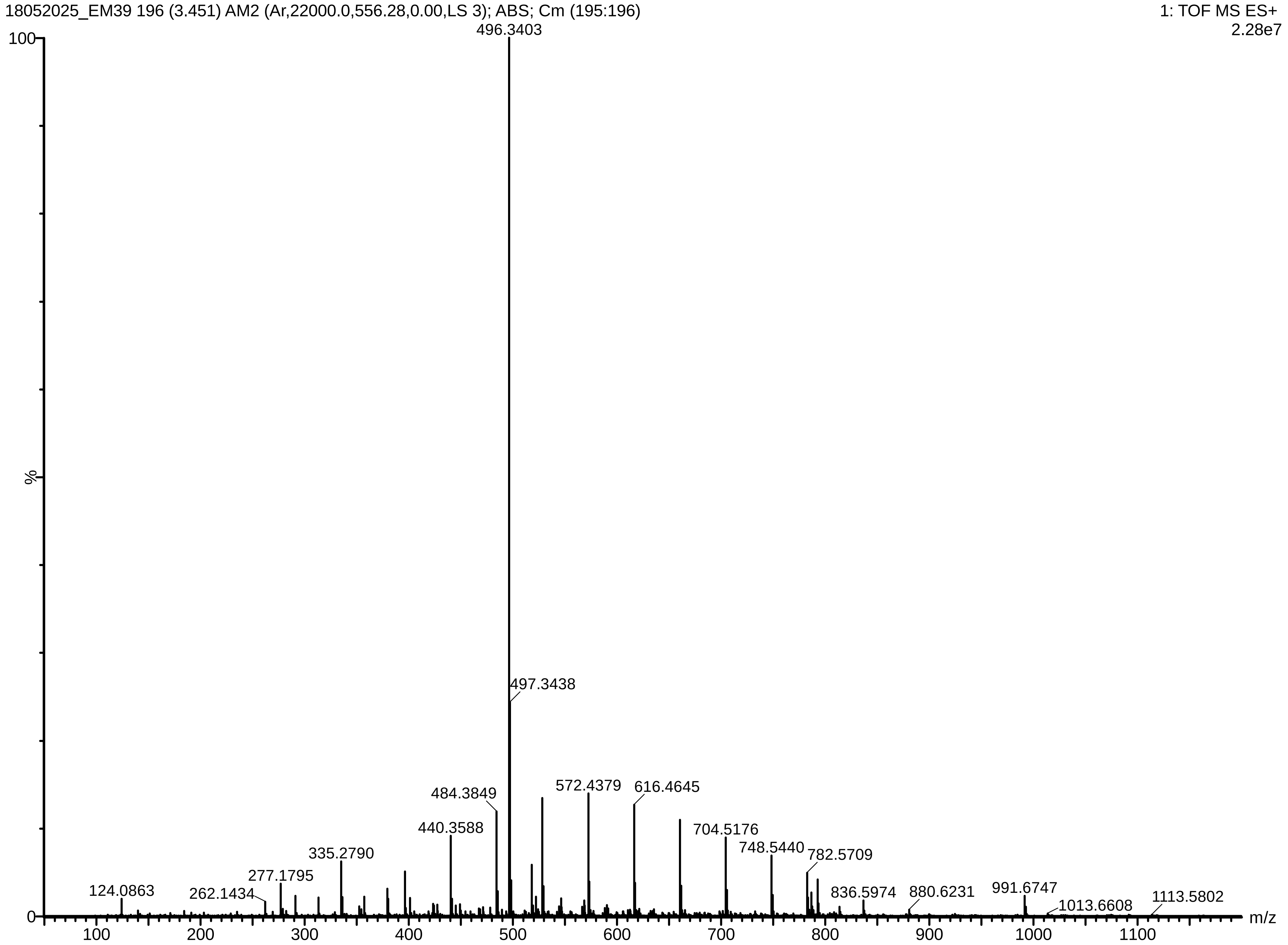
18052025_EM39 196 (3.451) AM2 (Ar,22000.0,556.28,0.00,LS 3); ABS; Cm (195:196)
1: TOF MS ES+
2.28e7
496.3403
497.3438
484.3849
572.4379
616.4645
440.3588
704.5176
748.5440
782.5709
335.2790
277.1795
124.0863
262.1434
836.5974
880.6231
991.6747
1013.6608
1113.5802
100
%
0
100
200
300
400
500
600
700
800
900
1000
1100
m/z
