# Supplementary material for: Metabolomic atlas of dengue virus infection reveals distinct circulating bioactive lipid signatures
Source: PLoS Negl Trop Dis. 2026 May 12;20(5):e0014327. doi: 10.1371/journal.pntd.0014327 (PMC13189415; doi:10.1371/journal.pntd.0014327)
Supplement: S1 Data — Electrospray ionisation time of flight mass spectrometry (ESI-TOF MS, positive mode) spectra of the dengue cohort and ESI-TOF at different retention times. The spectra display the relative abundance (%) of detected ions across the m/z range. Prominent peaks corresponding to major ionised species are indicated. Variation in spectral profiles between retention times reflects the differences in compound composition and ionisation patterns within the sample. Data were acquired under identical instrumental conditions and are presented as representative scans. (ZIP) [file pntd.0014327.s003.zip › EM COMPLETE SAMPLES SPECTRUM/EM39 SPECTRUM RT 3.823.pdf]

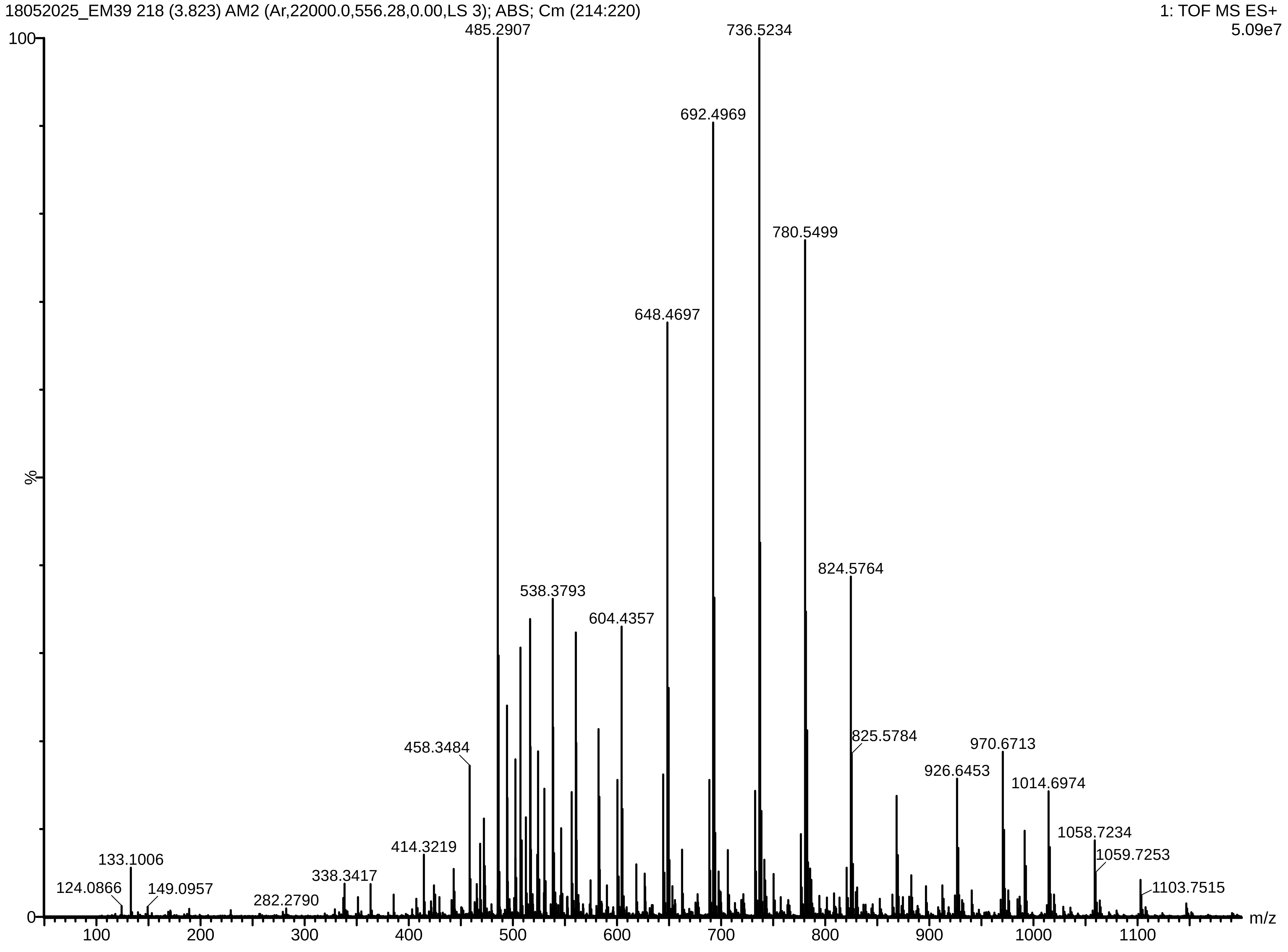

18052025_EM39 218 (3.823) AM2 (Ar,22000.0,556.28,0.00,LS 3); ABS; Cm (214:220)
1: TOF MS ES+
5.09e7
485.2907
736.5234
692.4969
780.5499
648.4697
824.5764
538.3793
604.4357
825.5784
970.6713
458.3484
926.6453
1014.6974
1058.7234
414.3219
1059.7253
133.1006
338.3417
124.0866
149.0957
1103.7515
282.2790
100
%
0
m/z
100
200
300
400
500
600
700
800
900
1000
1100
