# Supplementary material for: Metabolomic atlas of dengue virus infection reveals distinct circulating bioactive lipid signatures
Source: PLoS Negl Trop Dis. 2026 May 12;20(5):e0014327. doi: 10.1371/journal.pntd.0014327 (PMC13189415; doi:10.1371/journal.pntd.0014327)
Supplement: S1 Data — Electrospray ionisation time of flight mass spectrometry (ESI-TOF MS, positive mode) spectra of the dengue cohort and ESI-TOF at different retention times. The spectra display the relative abundance (%) of detected ions across the m/z range. Prominent peaks corresponding to major ionised species are indicated. Variation in spectral profiles between retention times reflects the differences in compound composition and ionisation patterns within the sample. Data were acquired under identical instrumental conditions and are presented as representative scans. (ZIP) [file pntd.0014327.s003.zip › EM COMPLETE SAMPLES SPECTRUM/EM44 SPECTRUM RT 0.459.pdf]

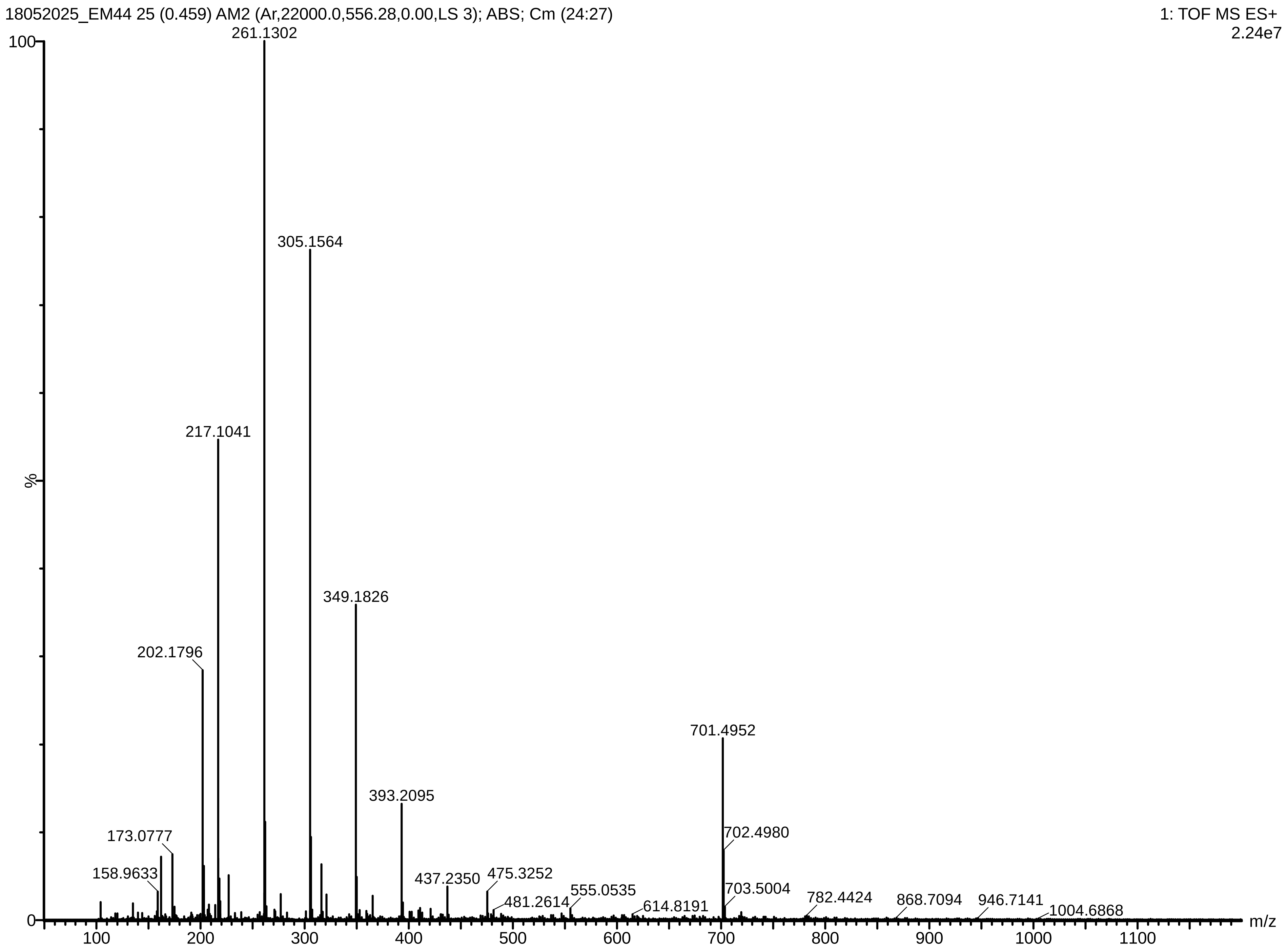
18052025_EM44 25 (0.459) AM2 (Ar,22000.0,556.28,0.00,LS 3); ABS; Cm (24:27)
1: TOF MS ES+
2.24e7
261.1302
305.1564
217.1041
349.1826
202.1796
701.4952
393.2095
702.4980
173.0777
158.9633
437.2350
475.3252
481.2614
555.0535
703.5004
614.8191
782.4424
868.7094
946.7141
1004.6868
%
m/z
100
0
100
200
300
400
500
600
700
800
900
1000
1100
