# Supplementary material for: Metabolomic atlas of dengue virus infection reveals distinct circulating bioactive lipid signatures
Source: PLoS Negl Trop Dis. 2026 May 12;20(5):e0014327. doi: 10.1371/journal.pntd.0014327 (PMC13189415; doi:10.1371/journal.pntd.0014327)
Supplement: S1 Data — Electrospray ionisation time of flight mass spectrometry (ESI-TOF MS, positive mode) spectra of the dengue cohort and ESI-TOF at different retention times. The spectra display the relative abundance (%) of detected ions across the m/z range. Prominent peaks corresponding to major ionised species are indicated. Variation in spectral profiles between retention times reflects the differences in compound composition and ionisation patterns within the sample. Data were acquired under identical instrumental conditions and are presented as representative scans. (ZIP) [file pntd.0014327.s003.zip › EM COMPLETE SAMPLES SPECTRUM/EM44 SPECTRUM RT 0.679.pdf]

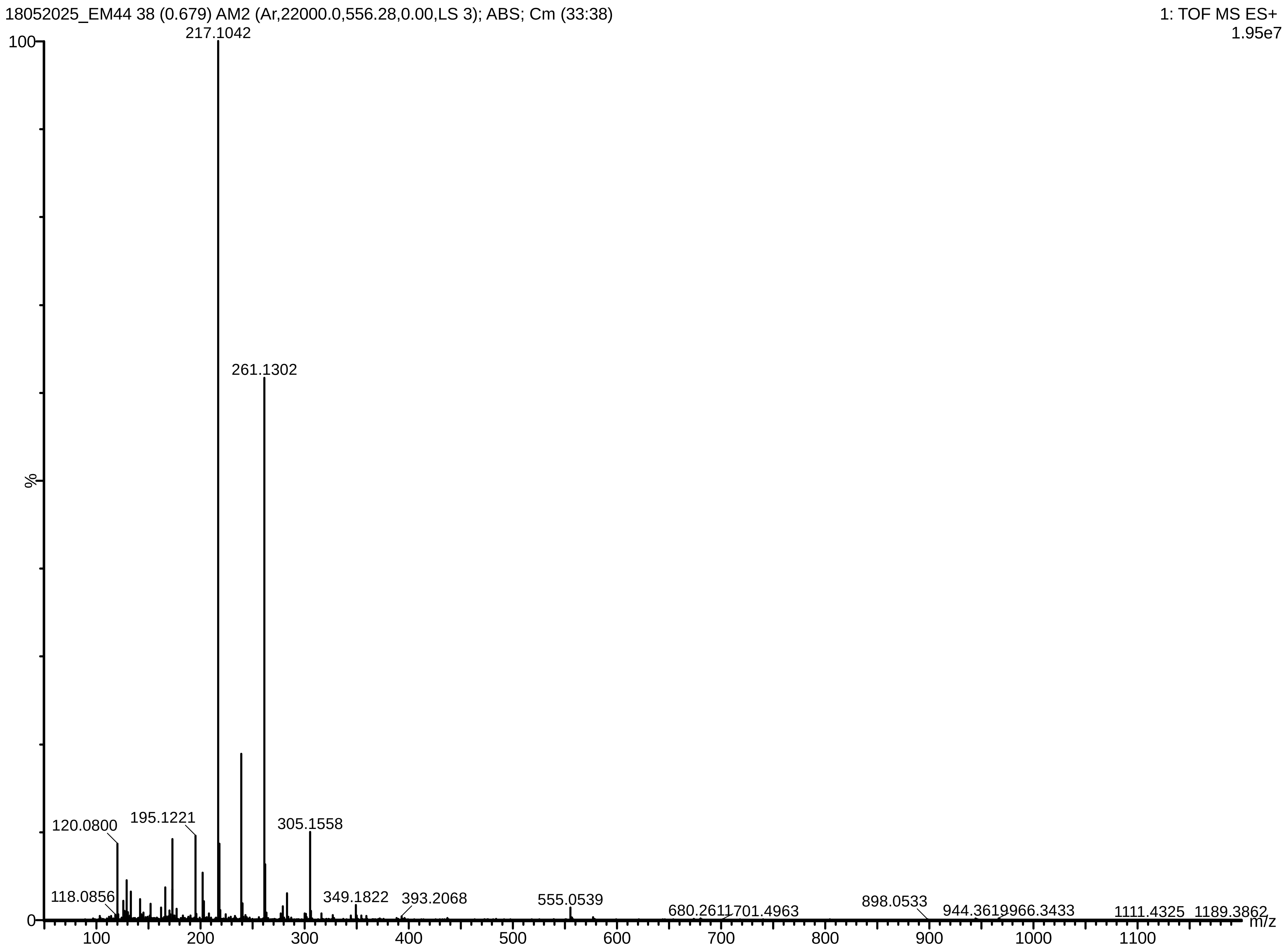
18052025_EM44 38 (0.679) AM2 (Ar,22000.0,556.28,0.00,LS 3); ABS; Cm (33:38)
1: TOF MS ES+
1.95e7
100
%
0
217.1042
261.1302
120.0800
195.1221
305.1558
118.0856
349.1822
393.2068
555.0539
680.2611701.4963
898.0533
944.3619966.3433
1111.4325
1189.3862
m/z
100
200
300
400
500
600
700
800
900
1000
1100
