# Supplementary material for: Metabolomic atlas of dengue virus infection reveals distinct circulating bioactive lipid signatures
Source: PLoS Negl Trop Dis. 2026 May 12;20(5):e0014327. doi: 10.1371/journal.pntd.0014327 (PMC13189415; doi:10.1371/journal.pntd.0014327)
Supplement: S1 Data — Electrospray ionisation time of flight mass spectrometry (ESI-TOF MS, positive mode) spectra of the dengue cohort and ESI-TOF at different retention times. The spectra display the relative abundance (%) of detected ions across the m/z range. Prominent peaks corresponding to major ionised species are indicated. Variation in spectral profiles between retention times reflects the differences in compound composition and ionisation patterns within the sample. Data were acquired under identical instrumental conditions and are presented as representative scans. (ZIP) [file pntd.0014327.s003.zip › EM COMPLETE SAMPLES SPECTRUM/EM44 SPECTRUM RT 2.058.pdf]

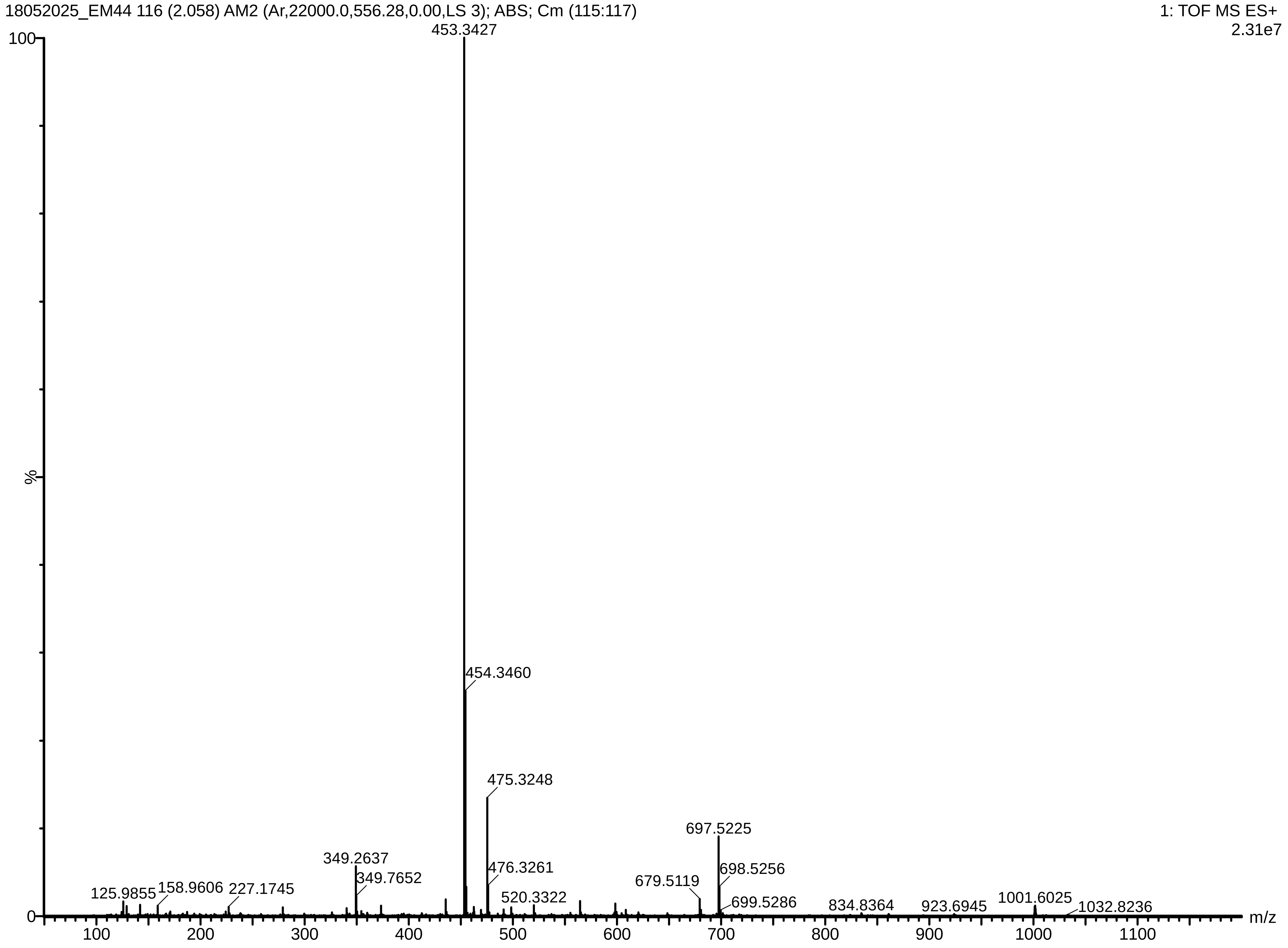
18052025_EM44 116 (2.058) AM2 (Ar,22000.0,556.28,0.00,LS 3); ABS; Cm (115:117)
1: TOF MS ES+
2.31e7
453.3427
454.3460
475.3248
697.5225
349.2637
349.7652
476.3261
698.5256
679.5119
125.9855
158.9606
227.1745
520.3322
699.5286
834.8364
923.6945
1001.6025
1032.8236
%
m/z
100
0
100
200
300
400
500
600
700
800
900
1000
1100
