# Supplementary material for: Metabolomic atlas of dengue virus infection reveals distinct circulating bioactive lipid signatures
Source: PLoS Negl Trop Dis. 2026 May 12;20(5):e0014327. doi: 10.1371/journal.pntd.0014327 (PMC13189415; doi:10.1371/journal.pntd.0014327)
Supplement: S1 Data — Electrospray ionisation time of flight mass spectrometry (ESI-TOF MS, positive mode) spectra of the dengue cohort and ESI-TOF at different retention times. The spectra display the relative abundance (%) of detected ions across the m/z range. Prominent peaks corresponding to major ionised species are indicated. Variation in spectral profiles between retention times reflects the differences in compound composition and ionisation patterns within the sample. Data were acquired under identical instrumental conditions and are presented as representative scans. (ZIP) [file pntd.0014327.s003.zip › EM COMPLETE SAMPLES SPECTRUM/EM44 SPECTRUM RT 2.565.pdf]

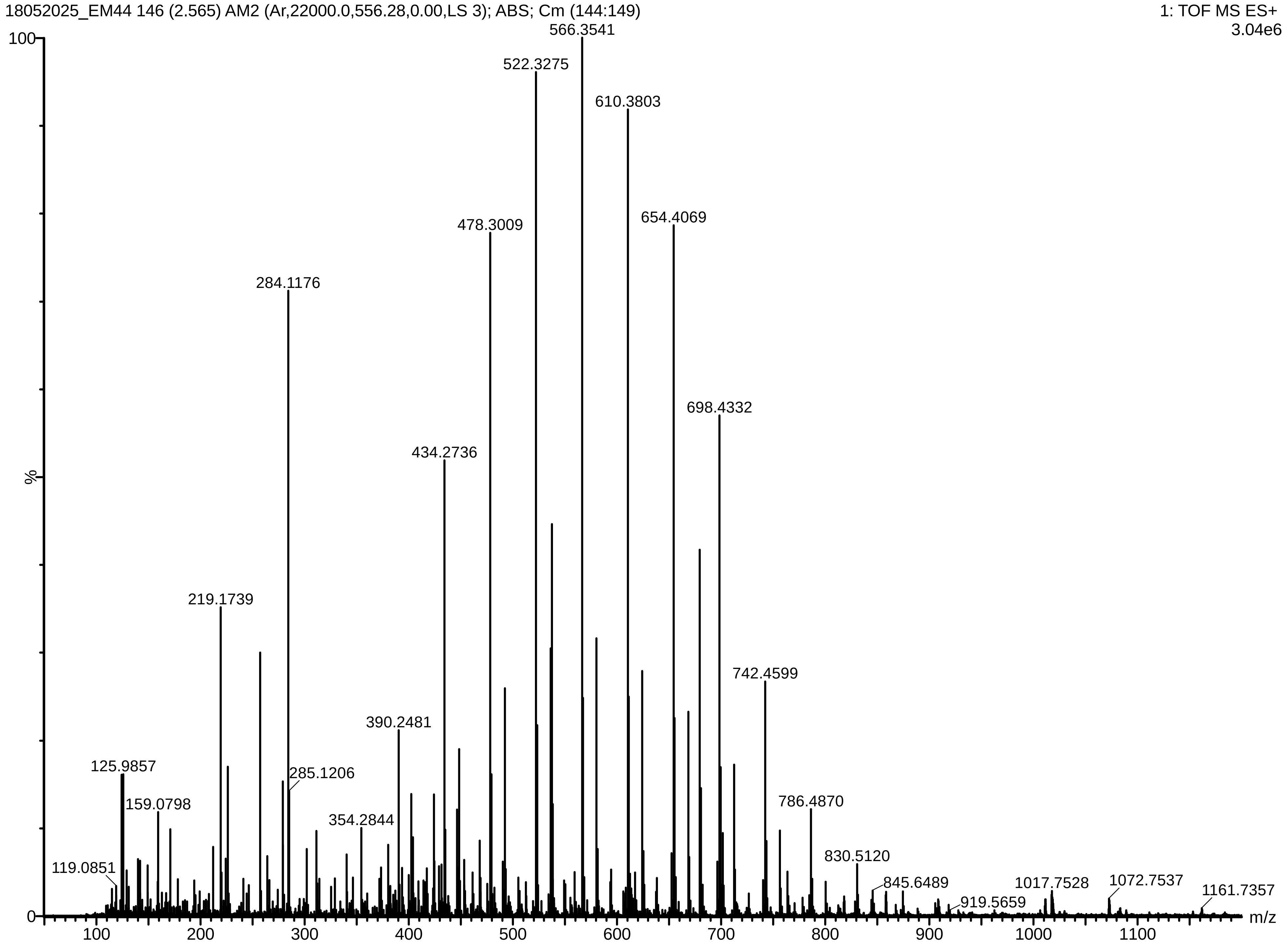
18052025_EM44 146 (2.565) AM2 (Ar,22000.0,556.28,0.00,LS 3); ABS; Cm (144:149)
1: TOF MS ES+
3.04e6
566.3541
522.3275
610.3803
654.4069
478.3009
284.1176
434.2736
698.4332
219.1739
742.4599
390.2481
125.9857
285.1206
159.0798
354.2844
786.4870
830.5120
119.0851
845.6489
1017.7528
1072.7537
1161.7357
919.5659
%
100
0
m/z
100
200
300
400
500
600
700
800
900
1000
1100
