# Supplementary material for: Metabolomic atlas of dengue virus infection reveals distinct circulating bioactive lipid signatures
Source: PLoS Negl Trop Dis. 2026 May 12;20(5):e0014327. doi: 10.1371/journal.pntd.0014327 (PMC13189415; doi:10.1371/journal.pntd.0014327)
Supplement: S1 Data — Electrospray ionisation time of flight mass spectrometry (ESI-TOF MS, positive mode) spectra of the dengue cohort and ESI-TOF at different retention times. The spectra display the relative abundance (%) of detected ions across the m/z range. Prominent peaks corresponding to major ionised species are indicated. Variation in spectral profiles between retention times reflects the differences in compound composition and ionisation patterns within the sample. Data were acquired under identical instrumental conditions and are presented as representative scans. (ZIP) [file pntd.0014327.s003.zip › EM COMPLETE SAMPLES SPECTRUM/EM44 SPECTRUM RT 2.822.pdf]

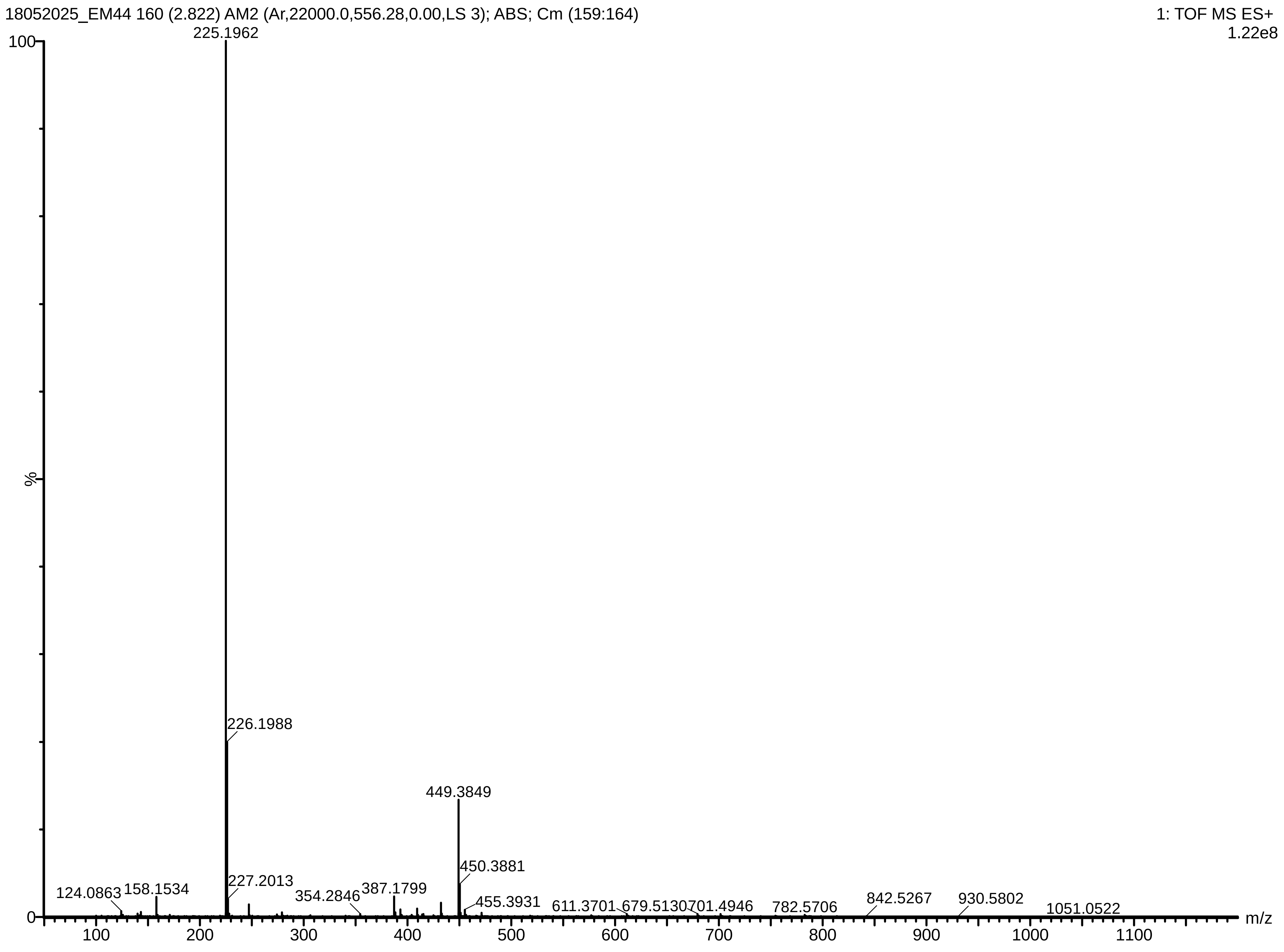

18052025_EM44 160 (2.822) AM2 (Ar,22000.0,556.28,0.00,LS 3); ABS; Cm (159:164)
1: TOF MS ES+
1.22e8
225.1962
226.1988
449.3849
450.3881
227.2013
124.0863
158.1534
354.2846
387.1799
455.3931
611.3701
679.5130
701.4946
782.5706
842.5267
930.5802
1051.0522
%
m/z
