# Supplementary material for: Metabolomic atlas of dengue virus infection reveals distinct circulating bioactive lipid signatures
Source: PLoS Negl Trop Dis. 2026 May 12;20(5):e0014327. doi: 10.1371/journal.pntd.0014327 (PMC13189415; doi:10.1371/journal.pntd.0014327)
Supplement: S1 Data — Electrospray ionisation time of flight mass spectrometry (ESI-TOF MS, positive mode) spectra of the dengue cohort and ESI-TOF at different retention times. The spectra display the relative abundance (%) of detected ions across the m/z range. Prominent peaks corresponding to major ionised species are indicated. Variation in spectral profiles between retention times reflects the differences in compound composition and ionisation patterns within the sample. Data were acquired under identical instrumental conditions and are presented as representative scans. (ZIP) [file pntd.0014327.s003.zip › EM COMPLETE SAMPLES SPECTRUM/EM44 SPECTRUM RT 3.279.pdf]

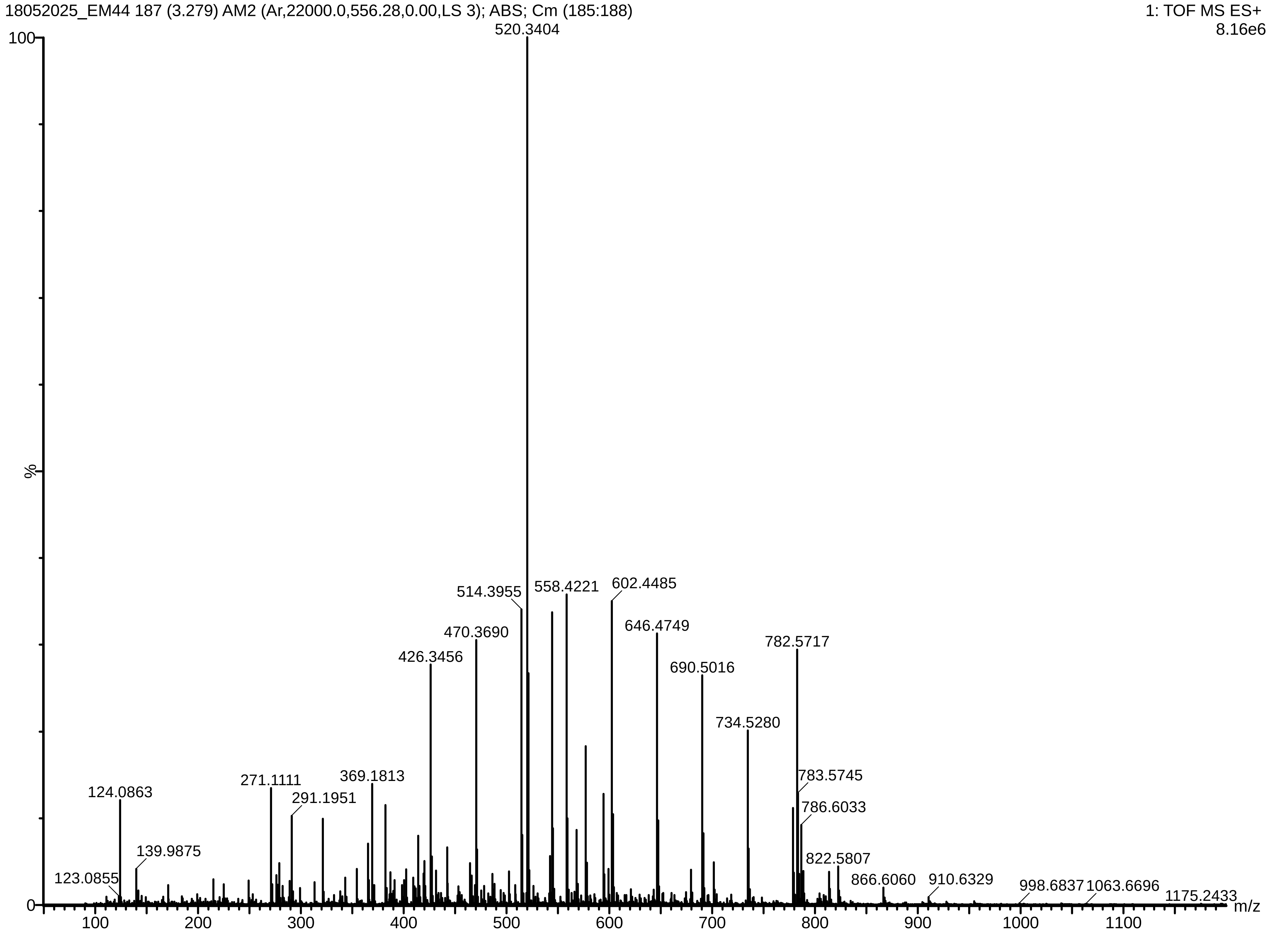

18052025_EM44 187 (3.279) AM2 (Ar,22000.0,556.28,0.00,LS 3); ABS; Cm (185:188)
1: TOF MS ES+
8.16e6
520.3404
514.3955
558.4221
602.4485
470.3690
646.4749
426.3456
782.5717
690.5016
734.5280
271.1111
369.1813
783.5745
124.0863
291.1951
786.6033
139.9875
822.5807
123.0855
866.6060
910.6329
998.6837
1063.6696
1175.2433
100
%
0
100
200
300
400
500
600
700
800
900
1000
1100
m/z
