# Supplementary material for: Metabolomic atlas of dengue virus infection reveals distinct circulating bioactive lipid signatures
Source: PLoS Negl Trop Dis. 2026 May 12;20(5):e0014327. doi: 10.1371/journal.pntd.0014327 (PMC13189415; doi:10.1371/journal.pntd.0014327)
Supplement: S1 Data — Electrospray ionisation time of flight mass spectrometry (ESI-TOF MS, positive mode) spectra of the dengue cohort and ESI-TOF at different retention times. The spectra display the relative abundance (%) of detected ions across the m/z range. Prominent peaks corresponding to major ionised species are indicated. Variation in spectral profiles between retention times reflects the differences in compound composition and ionisation patterns within the sample. Data were acquired under identical instrumental conditions and are presented as representative scans. (ZIP) [file pntd.0014327.s003.zip › EM COMPLETE SAMPLES SPECTRUM/EM44 SPECTRUM RT 3.434.pdf]

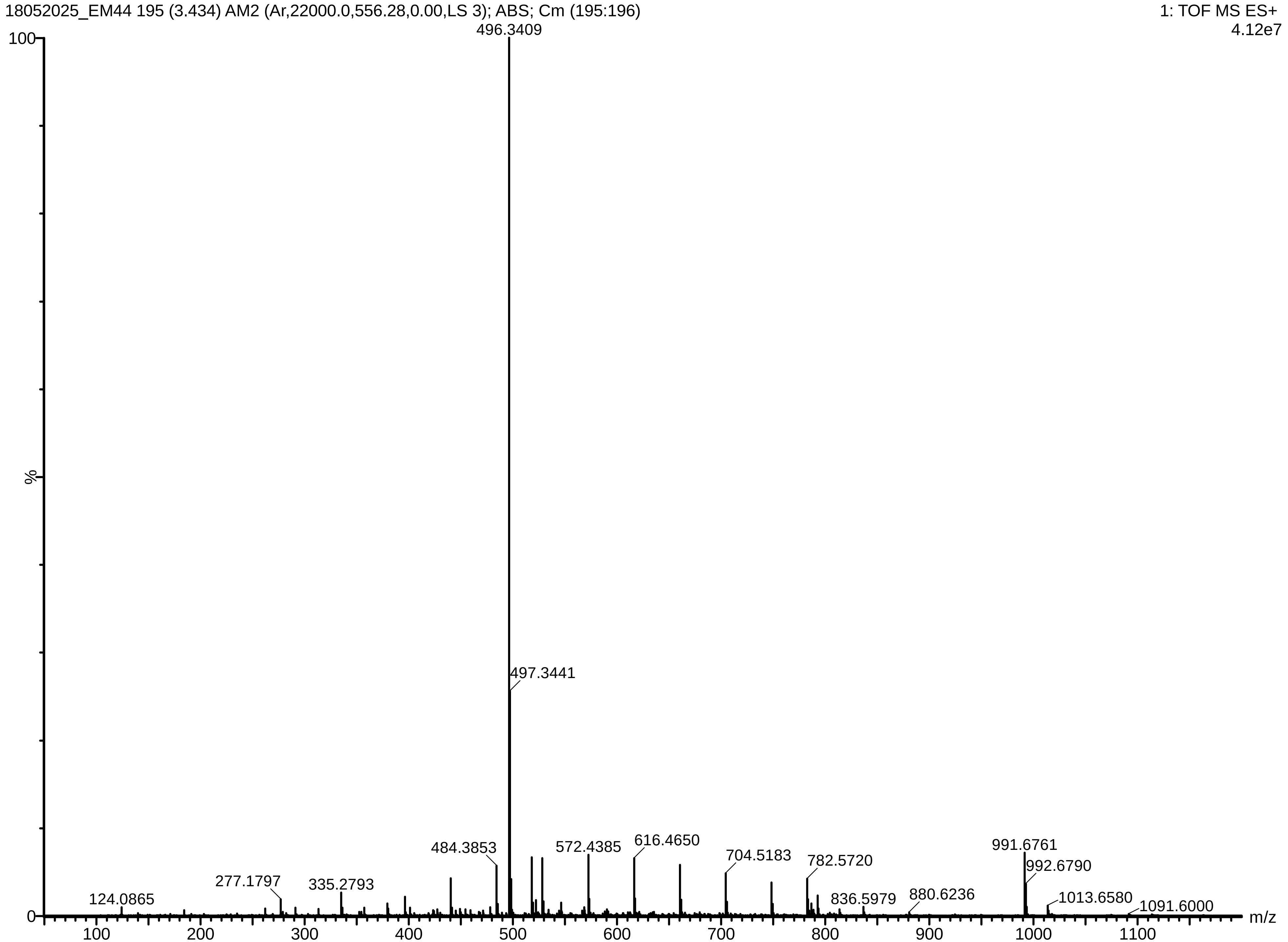

18052025_EM44 195 (3.434) AM2 (Ar,22000.0,556.28,0.00,LS 3); ABS; Cm (195:196)
1: TOF MS ES+
4.12e7
496.3409
497.3441
124.0865
277.1797
335.2793
484.3853
572.4385
616.4650
704.5183
782.5720
836.5979
880.6236
991.6761
992.6790
1013.6580
1091.6000
100
0
%
100
200
300
400
500
600
700
800
900
1000
1100
m/z
