# Supplementary material for: Metabolomic atlas of dengue virus infection reveals distinct circulating bioactive lipid signatures
Source: PLoS Negl Trop Dis. 2026 May 12;20(5):e0014327. doi: 10.1371/journal.pntd.0014327 (PMC13189415; doi:10.1371/journal.pntd.0014327)
Supplement: S1 Data — Electrospray ionisation time of flight mass spectrometry (ESI-TOF MS, positive mode) spectra of the dengue cohort and ESI-TOF at different retention times. The spectra display the relative abundance (%) of detected ions across the m/z range. Prominent peaks corresponding to major ionised species are indicated. Variation in spectral profiles between retention times reflects the differences in compound composition and ionisation patterns within the sample. Data were acquired under identical instrumental conditions and are presented as representative scans. (ZIP) [file pntd.0014327.s003.zip › EM COMPLETE SAMPLES SPECTRUM/EM44 SPECTRUM RT 3.823.pdf]

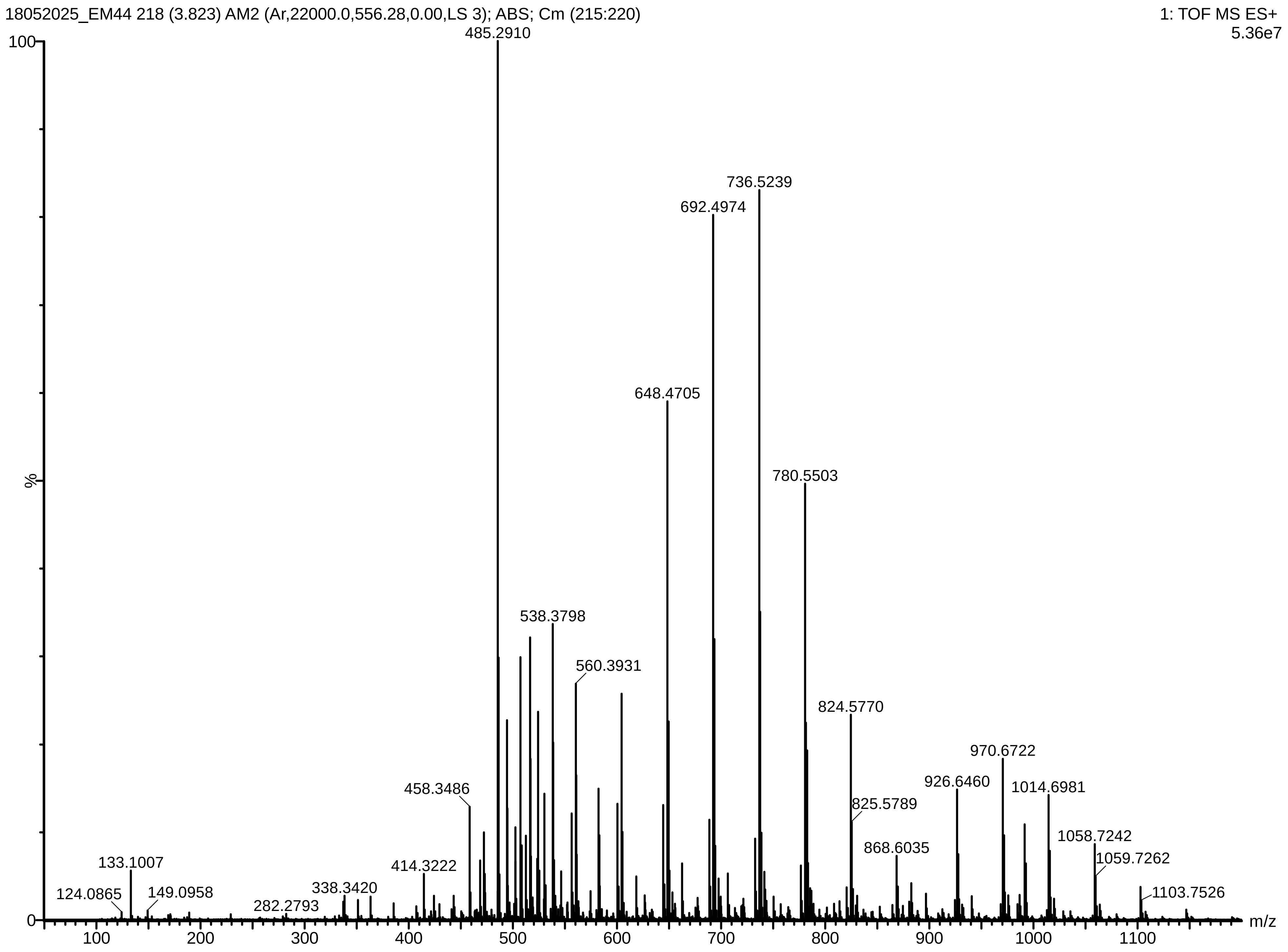
18052025_EM44 218 (3.823) AM2 (Ar,22000.0,556.28,0.00,LS 3); ABS; Cm (215:220)
1: TOF MS ES+
5.36e7
485.2910
736.5239
692.4974
648.4705
780.5503
538.3798
560.3931
824.5770
970.6722
926.6460
1014.6981
458.3486
825.5789
1058.7242
868.6035
1059.7262
133.1007
414.3222
338.3420
124.0865
149.0958
1103.7526
282.2793
100
%
0
m/z
100
200
300
400
500
600
700
800
900
1000
1100
