# Supplementary material for: Metabolomic atlas of dengue virus infection reveals distinct circulating bioactive lipid signatures
Source: PLoS Negl Trop Dis. 2026 May 12;20(5):e0014327. doi: 10.1371/journal.pntd.0014327 (PMC13189415; doi:10.1371/journal.pntd.0014327)
Supplement: S1 Data — Electrospray ionisation time of flight mass spectrometry (ESI-TOF MS, positive mode) spectra of the dengue cohort and ESI-TOF at different retention times. The spectra display the relative abundance (%) of detected ions across the m/z range. Prominent peaks corresponding to major ionised species are indicated. Variation in spectral profiles between retention times reflects the differences in compound composition and ionisation patterns within the sample. Data were acquired under identical instrumental conditions and are presented as representative scans. (ZIP) [file pntd.0014327.s003.zip › EM COMPLETE SAMPLES SPECTRUM/EM56 SPECTRUM RT 0.459.pdf]

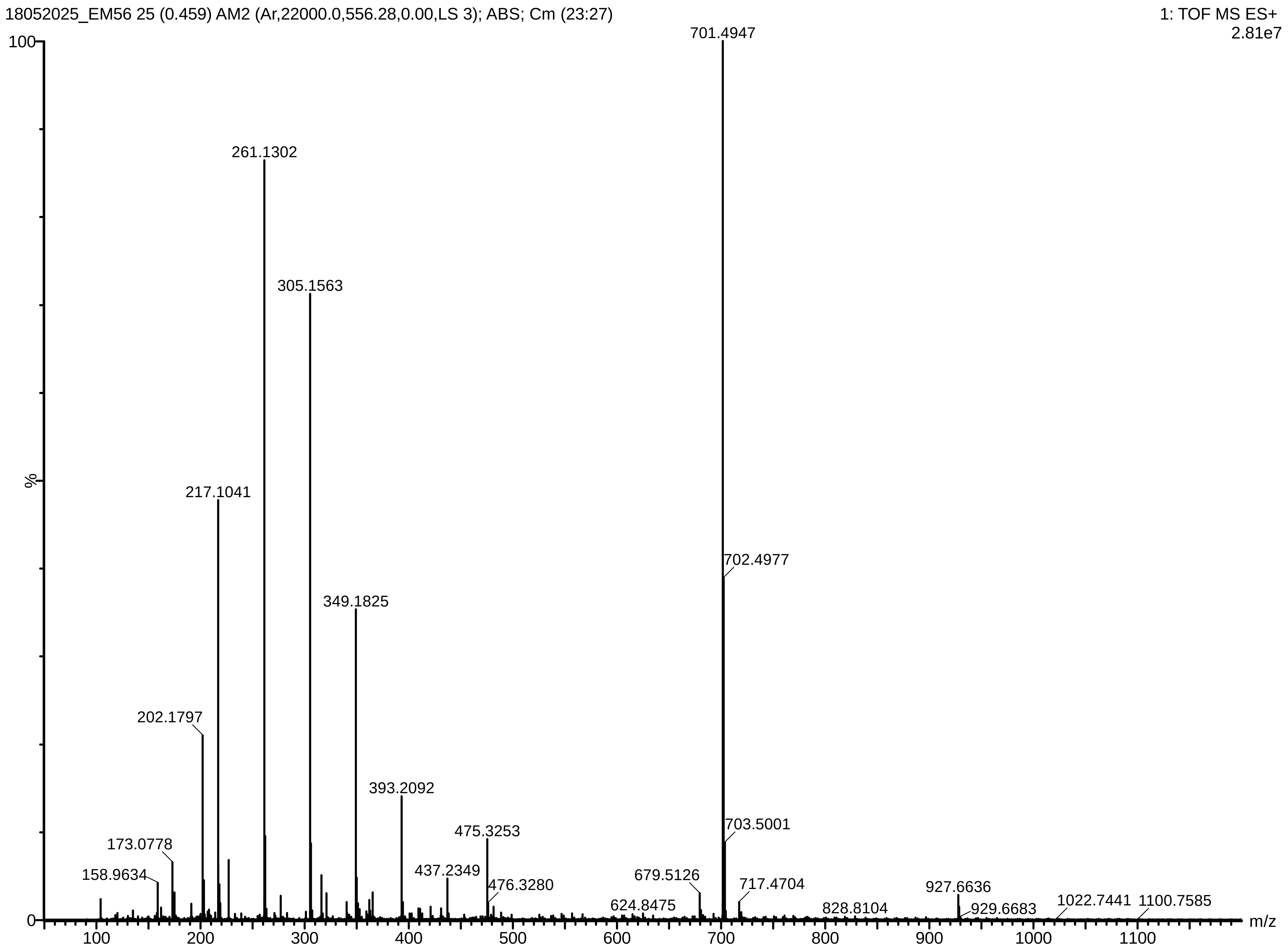
18052025_EM56 25 (0.459) AM2 (Ar,22000.0,556.28,0.00,LS 3); ABS; Cm (23:27)
1: TOF MS ES+
2.81e7
701.4947
261.1302
305.1563
217.1041
702.4977
349.1825
202.1797
393.2092
475.3253
703.5001
173.0778
437.2349
158.9634
679.5126
476.3280
717.4704
927.6636
624.8475
828.8104
929.6683
1022.7441
1100.7585
%
m/z
100
0
100
200
300
400
500
600
700
800
900
1000
1100
