# Supplementary material for: Metabolomic atlas of dengue virus infection reveals distinct circulating bioactive lipid signatures
Source: PLoS Negl Trop Dis. 2026 May 12;20(5):e0014327. doi: 10.1371/journal.pntd.0014327 (PMC13189415; doi:10.1371/journal.pntd.0014327)
Supplement: S1 Data — Electrospray ionisation time of flight mass spectrometry (ESI-TOF MS, positive mode) spectra of the dengue cohort and ESI-TOF at different retention times. The spectra display the relative abundance (%) of detected ions across the m/z range. Prominent peaks corresponding to major ionised species are indicated. Variation in spectral profiles between retention times reflects the differences in compound composition and ionisation patterns within the sample. Data were acquired under identical instrumental conditions and are presented as representative scans. (ZIP) [file pntd.0014327.s003.zip › EM COMPLETE SAMPLES SPECTRUM/EM56 SPECTRUM RT 0.679.pdf]

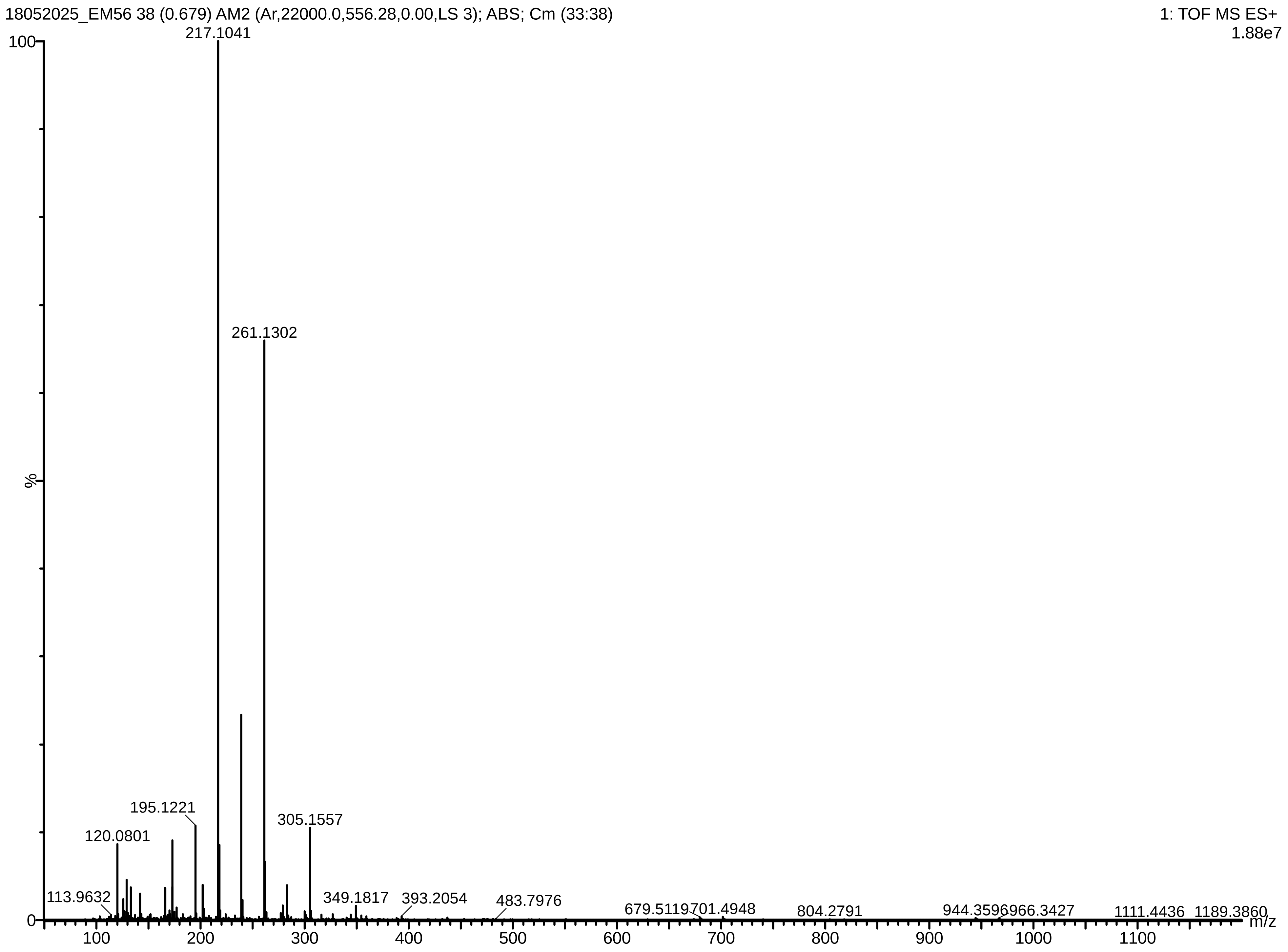

18052025_EM56 38 (0.679) AM2 (Ar,22000.0,556.28,0.00,LS 3); ABS; Cm (33:38)
1: TOF MS ES+
1.88e7
217.1041
261.1302
195.1221
305.1557
120.0801
113.9632
349.1817
393.2054
483.7976
679.5119701.4948
804.2791
944.3596966.3427
1111.4436
1189.3860
100
%
0
m/z
100
200
300
400
500
600
700
800
900
1000
1100
