# Supplementary material for: Metabolomic atlas of dengue virus infection reveals distinct circulating bioactive lipid signatures
Source: PLoS Negl Trop Dis. 2026 May 12;20(5):e0014327. doi: 10.1371/journal.pntd.0014327 (PMC13189415; doi:10.1371/journal.pntd.0014327)
Supplement: S1 Data — Electrospray ionisation time of flight mass spectrometry (ESI-TOF MS, positive mode) spectra of the dengue cohort and ESI-TOF at different retention times. The spectra display the relative abundance (%) of detected ions across the m/z range. Prominent peaks corresponding to major ionised species are indicated. Variation in spectral profiles between retention times reflects the differences in compound composition and ionisation patterns within the sample. Data were acquired under identical instrumental conditions and are presented as representative scans. (ZIP) [file pntd.0014327.s003.zip › EM COMPLETE SAMPLES SPECTRUM/EM56 SPECTRUM RT 2.566.pdf]

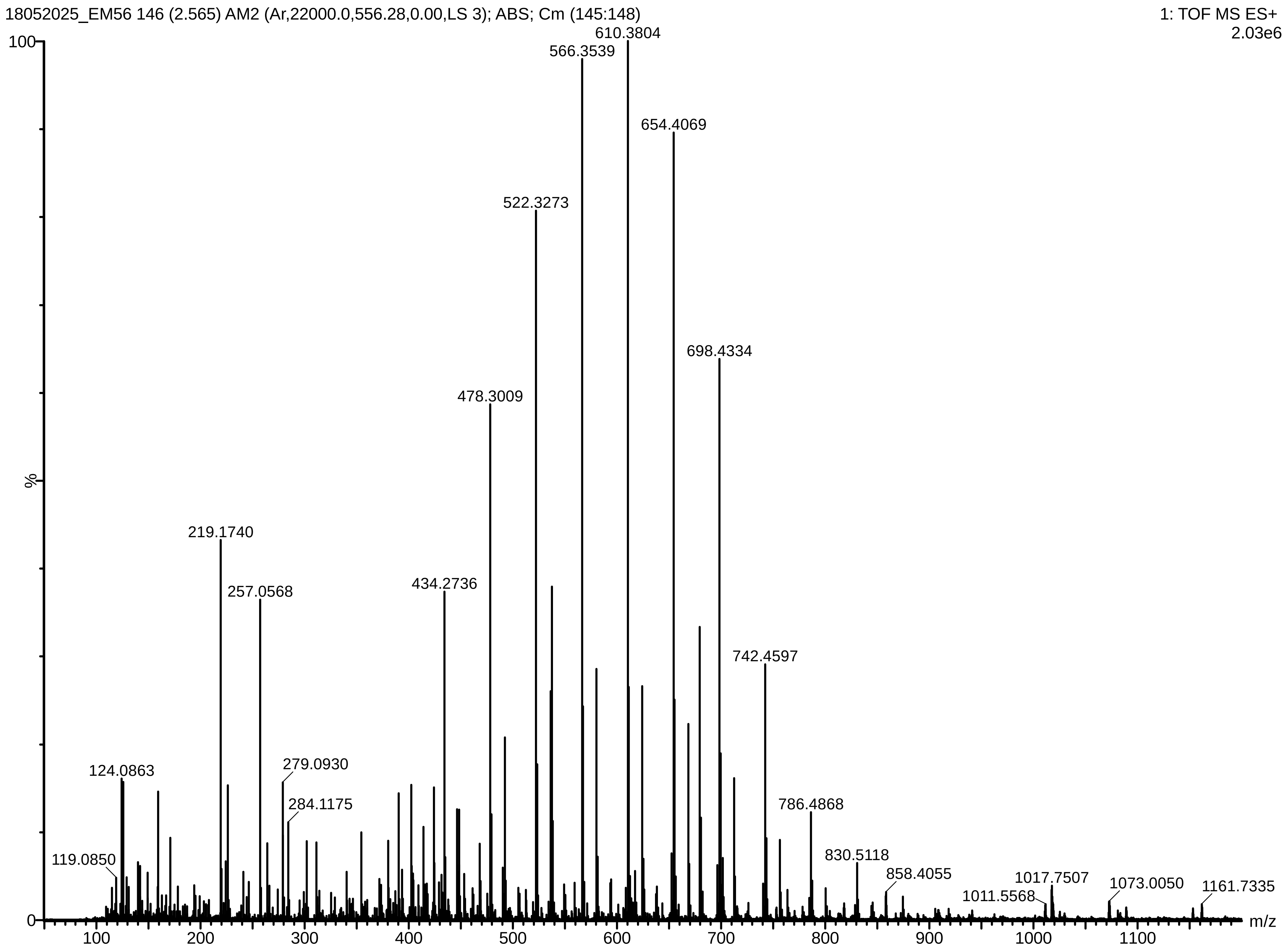
18052025_EM56 146 (2.565) AM2 (Ar,22000.0,556.28,0.00,LS 3); ABS; Cm (145:148)
1: TOF MS ES+
2.03e6
610.3804
566.3539
654.4069
522.3273
698.4334
478.3009
219.1740
257.0568
434.2736
742.4597
124.0863
279.0930
284.1175
786.4868
119.0850
830.5118
858.4055
1017.7507
1073.0050
1161.7335
1011.5568
100
%
0
100
200
300
400
500
600
700
800
900
1000
1100
m/z
