# Supplementary material for: Metabolomic atlas of dengue virus infection reveals distinct circulating bioactive lipid signatures
Source: PLoS Negl Trop Dis. 2026 May 12;20(5):e0014327. doi: 10.1371/journal.pntd.0014327 (PMC13189415; doi:10.1371/journal.pntd.0014327)
Supplement: S1 Data — Electrospray ionisation time of flight mass spectrometry (ESI-TOF MS, positive mode) spectra of the dengue cohort and ESI-TOF at different retention times. The spectra display the relative abundance (%) of detected ions across the m/z range. Prominent peaks corresponding to major ionised species are indicated. Variation in spectral profiles between retention times reflects the differences in compound composition and ionisation patterns within the sample. Data were acquired under identical instrumental conditions and are presented as representative scans. (ZIP) [file pntd.0014327.s003.zip › EM COMPLETE SAMPLES SPECTRUM/EM56 SPECTRUM RT 2.873.pdf]

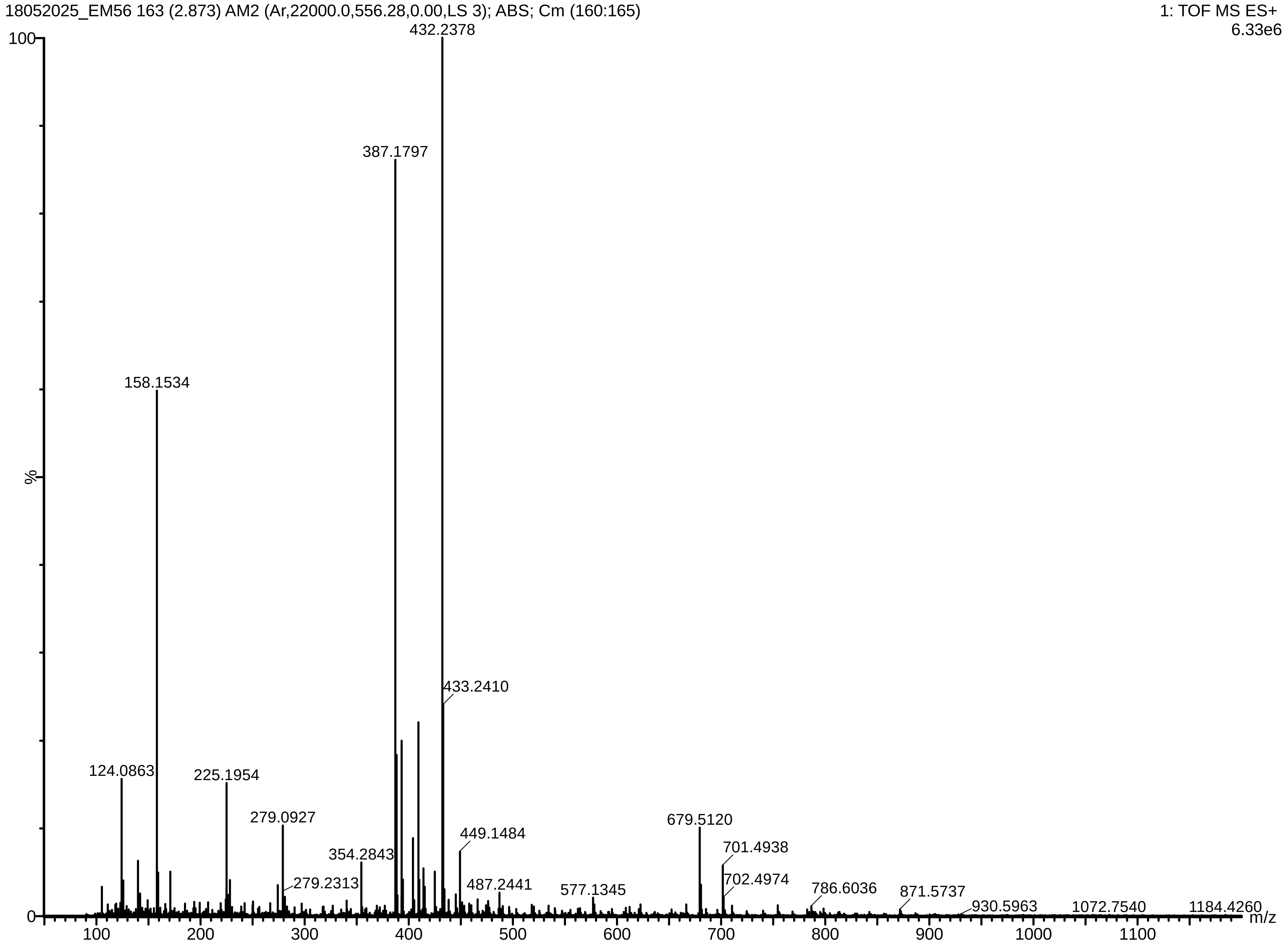
18052025_EM56 163 (2.873) AM2 (Ar,22000.0,556.28,0.00,LS 3); ABS; Cm (160:165)
1: TOF MS ES+
6.33e6
432.2378
387.1797
158.1534
433.2410
124.0863
225.1954
279.0927
279.2313
354.2843
449.1484
487.2441
577.1345
679.5120
701.4938
702.4974
786.6036
871.5737
930.5963
1072.7540
1184.4260
%
100
0
m/z
100
200
300
400
500
600
700
800
900
1000
1100
