# Supplementary material for: Metabolomic atlas of dengue virus infection reveals distinct circulating bioactive lipid signatures
Source: PLoS Negl Trop Dis. 2026 May 12;20(5):e0014327. doi: 10.1371/journal.pntd.0014327 (PMC13189415; doi:10.1371/journal.pntd.0014327)
Supplement: S1 Data — Electrospray ionisation time of flight mass spectrometry (ESI-TOF MS, positive mode) spectra of the dengue cohort and ESI-TOF at different retention times. The spectra display the relative abundance (%) of detected ions across the m/z range. Prominent peaks corresponding to major ionised species are indicated. Variation in spectral profiles between retention times reflects the differences in compound composition and ionisation patterns within the sample. Data were acquired under identical instrumental conditions and are presented as representative scans. (ZIP) [file pntd.0014327.s003.zip › EM COMPLETE SAMPLES SPECTRUM/EM56 SPECTRUM RT 3.279.pdf]

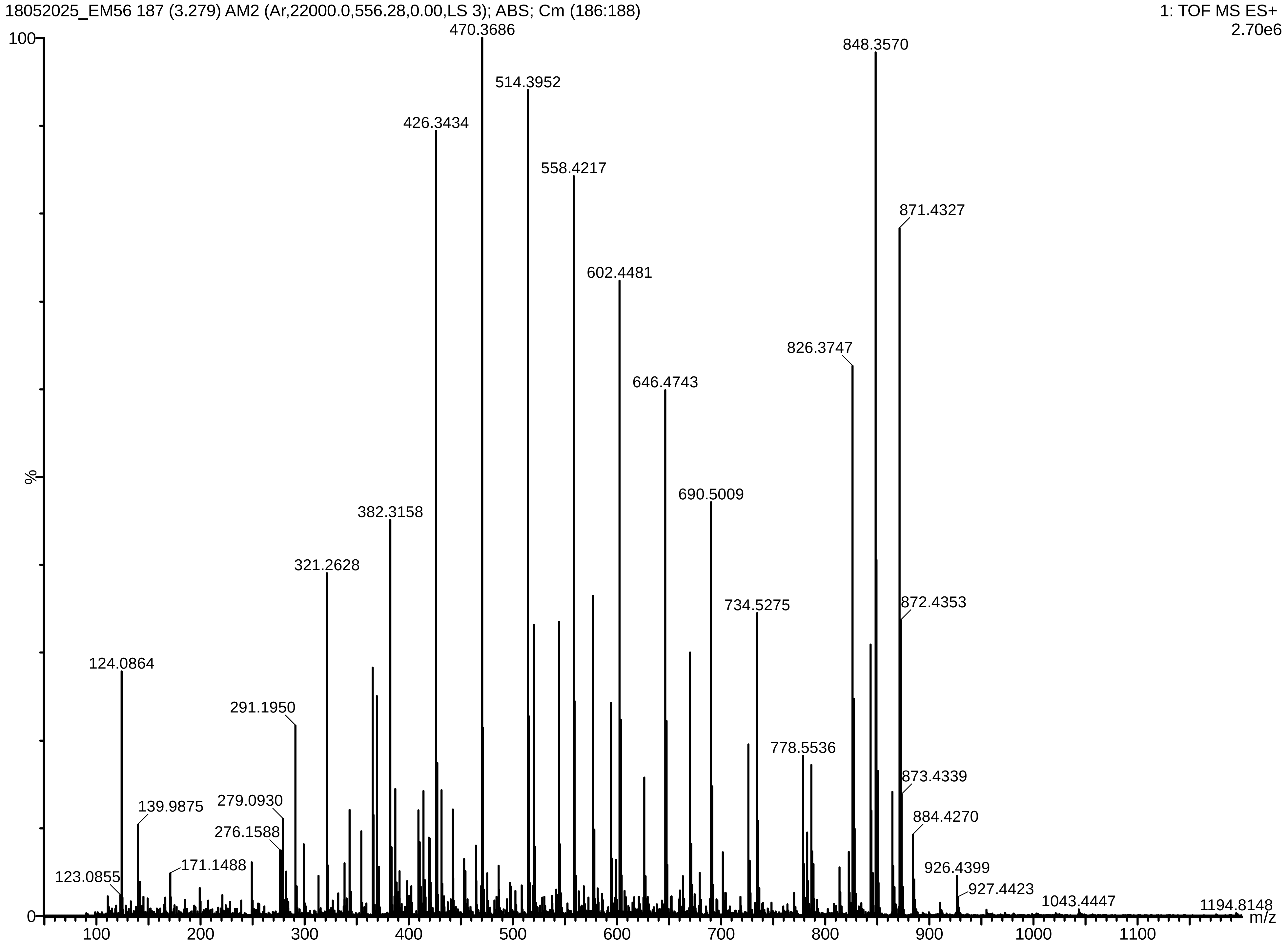
18052025_EM56 187 (3.279) AM2 (Ar,22000.0,556.28,0.00,LS 3); ABS; Cm (186:188)
1: TOF MS ES+
2.70e6
100
%
0
470.3686
848.3570
514.3952
426.3434
558.4217
871.4327
602.4481
826.3747
646.4743
690.5009
382.3158
321.2628
734.5275
872.4353
124.0864
291.1950
778.5536
873.4339
139.9875
279.0930
884.4270
276.1588
171.1488
123.0855
926.4399
927.4423
1043.4447
1194.8148
m/z
100
200
300
400
500
600
700
800
900
1000
1100
