# Supplementary material for: Metabolomic atlas of dengue virus infection reveals distinct circulating bioactive lipid signatures
Source: PLoS Negl Trop Dis. 2026 May 12;20(5):e0014327. doi: 10.1371/journal.pntd.0014327 (PMC13189415; doi:10.1371/journal.pntd.0014327)
Supplement: S1 Data — Electrospray ionisation time of flight mass spectrometry (ESI-TOF MS, positive mode) spectra of the dengue cohort and ESI-TOF at different retention times. The spectra display the relative abundance (%) of detected ions across the m/z range. Prominent peaks corresponding to major ionised species are indicated. Variation in spectral profiles between retention times reflects the differences in compound composition and ionisation patterns within the sample. Data were acquired under identical instrumental conditions and are presented as representative scans. (ZIP) [file pntd.0014327.s003.zip › EM COMPLETE SAMPLES SPECTRUM/EM56 SPECTRUM RT 3.434.pdf]

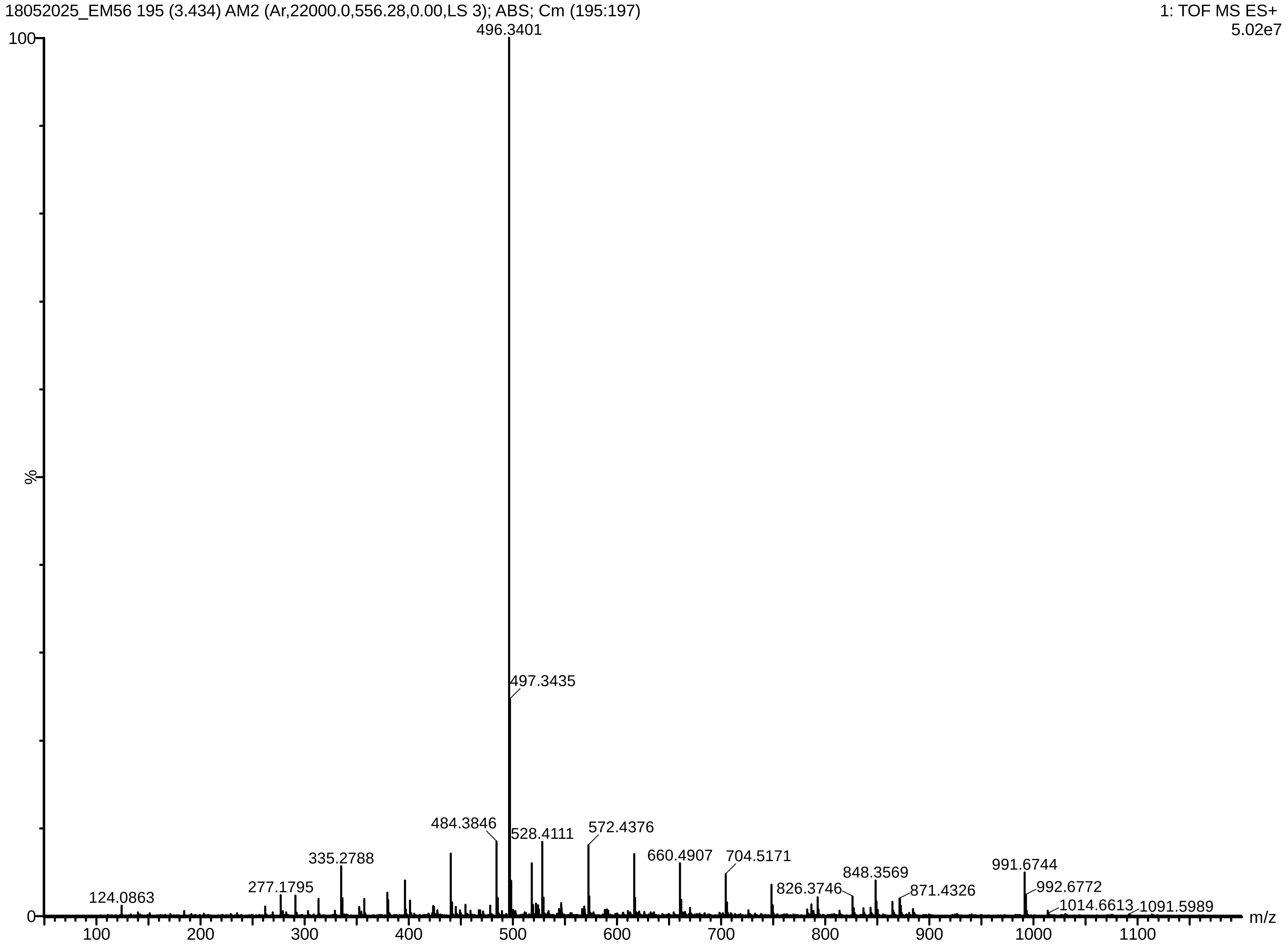
18052025_EM56 195 (3.434) AM2 (Ar,22000.0,556.28,0.00,LS 3); ABS; Cm (195:197)
1: TOF MS ES+
5.02e7
496.3401
497.3435
484.3846
528.4111
572.4376
335.2788
660.4907
704.5171
848.3569
991.6744
277.1795
826.3746
871.4326
992.6772
124.0863
1014.6613
1091.5989
%
100
0
m/z
100
200
300
400
500
600
700
800
900
1000
1100
