# Supplementary material for: Metabolomic atlas of dengue virus infection reveals distinct circulating bioactive lipid signatures
Source: PLoS Negl Trop Dis. 2026 May 12;20(5):e0014327. doi: 10.1371/journal.pntd.0014327 (PMC13189415; doi:10.1371/journal.pntd.0014327)
Supplement: S1 Data — Electrospray ionisation time of flight mass spectrometry (ESI-TOF MS, positive mode) spectra of the dengue cohort and ESI-TOF at different retention times. The spectra display the relative abundance (%) of detected ions across the m/z range. Prominent peaks corresponding to major ionised species are indicated. Variation in spectral profiles between retention times reflects the differences in compound composition and ionisation patterns within the sample. Data were acquired under identical instrumental conditions and are presented as representative scans. (ZIP) [file pntd.0014327.s003.zip › EM COMPLETE SAMPLES SPECTRUM/EM56 SPECTRUM RT 3.823.pdf]

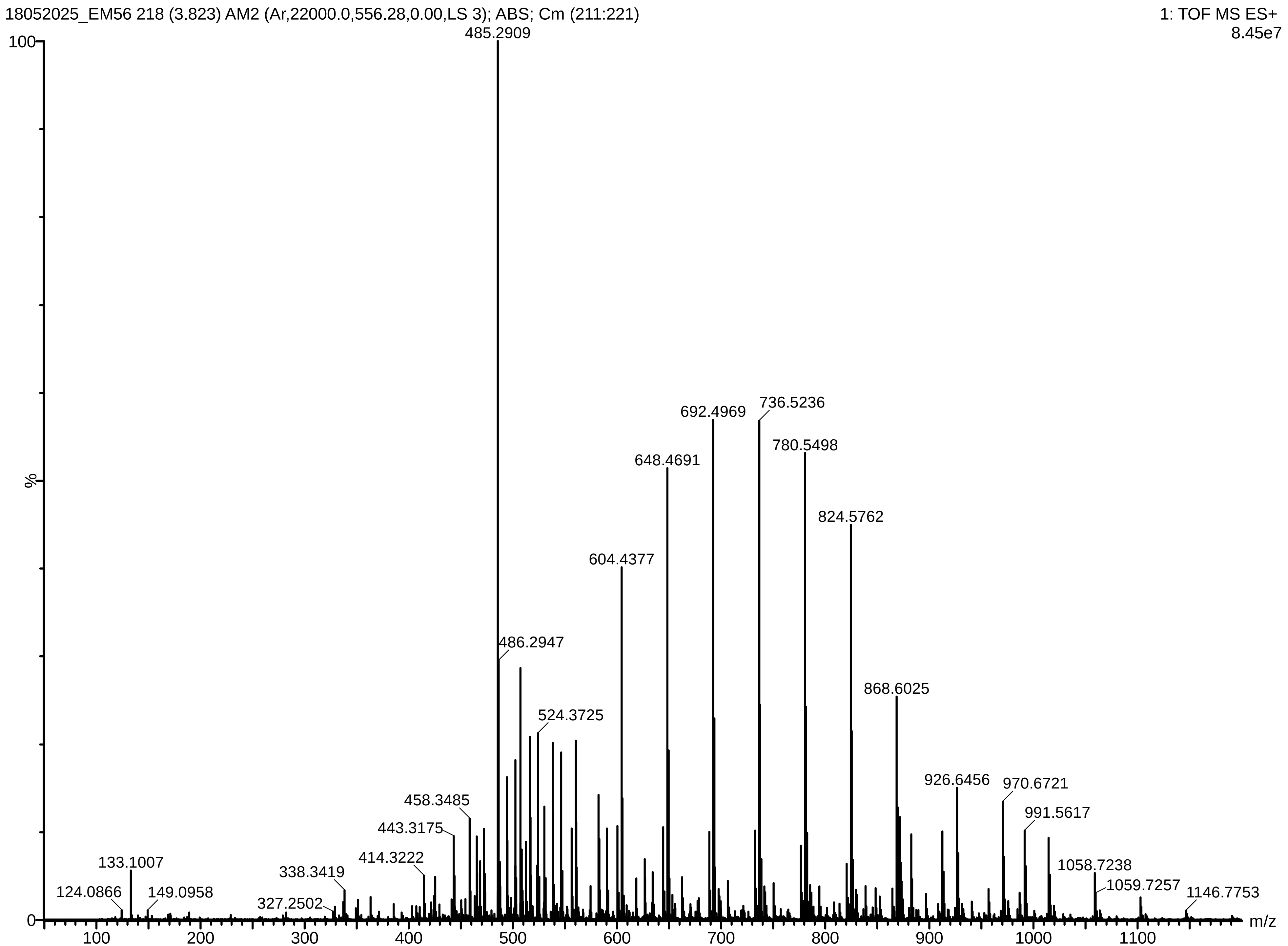

18052025_EM56 218 (3.823) AM2 (Ar,22000.0,556.28,0.00,LS 3); ABS; Cm (211:221)
1: TOF MS ES+
8.45e7
485.2909
486.2947
524.3725
604.4377
648.4691
692.4969
736.5236
780.5498
824.5762
868.6025
926.6456
970.6721
991.5617
1058.7238
1059.7257
1146.7753
458.3485
443.3175
414.3222
338.3419
327.2502
133.1007
124.0866
149.0958
100
%
0
m/z
100
200
300
400
500
600
700
800
900
1000
1100
