# Supplementary material for: Metabolomic atlas of dengue virus infection reveals distinct circulating bioactive lipid signatures
Source: PLoS Negl Trop Dis. 2026 May 12;20(5):e0014327. doi: 10.1371/journal.pntd.0014327 (PMC13189415; doi:10.1371/journal.pntd.0014327)
Supplement: S1 Data — Electrospray ionisation time of flight mass spectrometry (ESI-TOF MS, positive mode) spectra of the dengue cohort and ESI-TOF at different retention times. The spectra display the relative abundance (%) of detected ions across the m/z range. Prominent peaks corresponding to major ionised species are indicated. Variation in spectral profiles between retention times reflects the differences in compound composition and ionisation patterns within the sample. Data were acquired under identical instrumental conditions and are presented as representative scans. (ZIP) [file pntd.0014327.s003.zip › EM COMPLETE SAMPLES SPECTRUM/EM58 SPECTRUM RT 0.459.pdf]

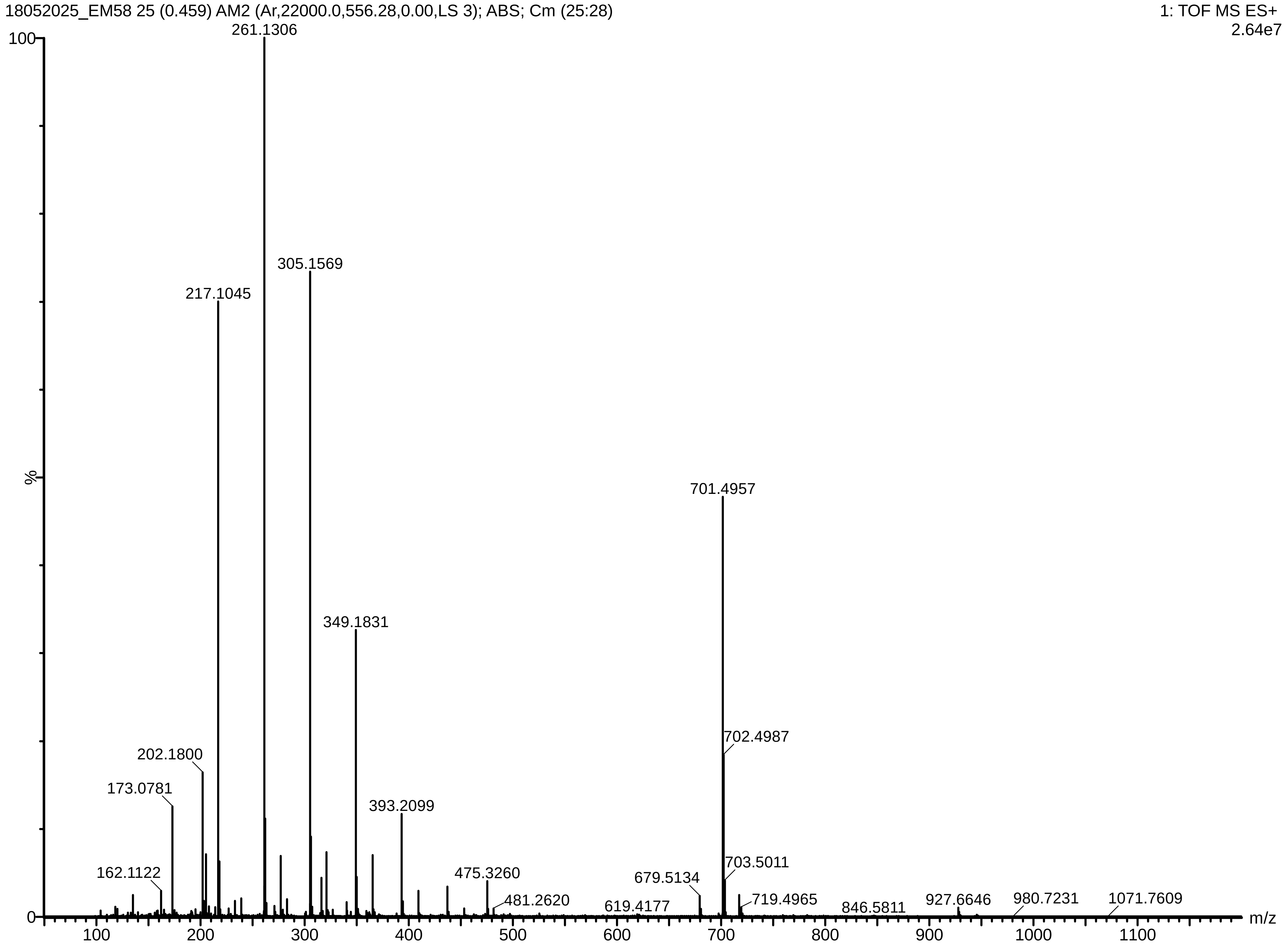
18052025_EM58 25 (0.459) AM2 (Ar,22000.0,556.28,0.00,LS 3); ABS; Cm (25:28)
1: TOF MS ES+
2.64e7
100
%
0
261.1306
305.1569
217.1045
701.4957
349.1831
702.4987
202.1800
173.0781
393.2099
703.5011
162.1122
475.3260
679.5134
481.2620
719.4965
619.4177
846.5811
927.6646
980.7231
1071.7609
100
200
300
400
500
600
700
800
900
1000
1100
m/z
