# Supplementary material for: Metabolomic atlas of dengue virus infection reveals distinct circulating bioactive lipid signatures
Source: PLoS Negl Trop Dis. 2026 May 12;20(5):e0014327. doi: 10.1371/journal.pntd.0014327 (PMC13189415; doi:10.1371/journal.pntd.0014327)
Supplement: S1 Data — Electrospray ionisation time of flight mass spectrometry (ESI-TOF MS, positive mode) spectra of the dengue cohort and ESI-TOF at different retention times. The spectra display the relative abundance (%) of detected ions across the m/z range. Prominent peaks corresponding to major ionised species are indicated. Variation in spectral profiles between retention times reflects the differences in compound composition and ionisation patterns within the sample. Data were acquired under identical instrumental conditions and are presented as representative scans. (ZIP) [file pntd.0014327.s003.zip › EM COMPLETE SAMPLES SPECTRUM/EM58 SPECTRUM RT 0.612.pdf]

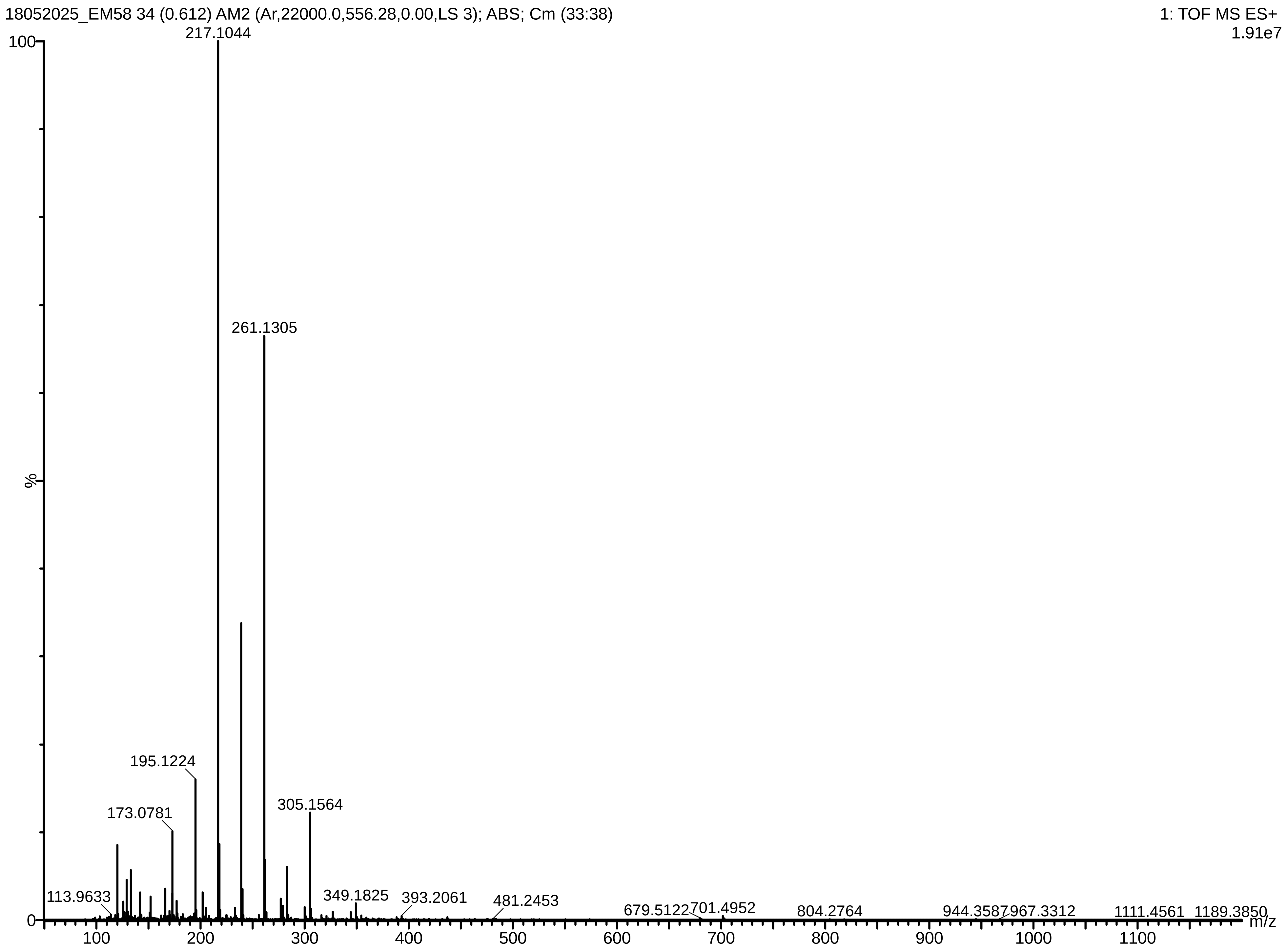
18052025_EM58 34 (0.612) AM2 (Ar,22000.0,556.28,0.00,LS 3); ABS; Cm (33:38)
1: TOF MS ES+
1.91e7
217.1044
261.1305
195.1224
173.0781
305.1564
113.9633
349.1825
393.2061
481.2453
679.5122
701.4952
804.2764
944.3587
967.3312
1111.4561
1189.3850
%
m/z
100
0
100
200
300
400
500
600
700
800
900
1000
1100
