# Supplementary material for: Metabolomic atlas of dengue virus infection reveals distinct circulating bioactive lipid signatures
Source: PLoS Negl Trop Dis. 2026 May 12;20(5):e0014327. doi: 10.1371/journal.pntd.0014327 (PMC13189415; doi:10.1371/journal.pntd.0014327)
Supplement: S1 Data — Electrospray ionisation time of flight mass spectrometry (ESI-TOF MS, positive mode) spectra of the dengue cohort and ESI-TOF at different retention times. The spectra display the relative abundance (%) of detected ions across the m/z range. Prominent peaks corresponding to major ionised species are indicated. Variation in spectral profiles between retention times reflects the differences in compound composition and ionisation patterns within the sample. Data were acquired under identical instrumental conditions and are presented as representative scans. (ZIP) [file pntd.0014327.s003.zip › EM COMPLETE SAMPLES SPECTRUM/EM58 SPECTRUM RT 2.058.pdf]

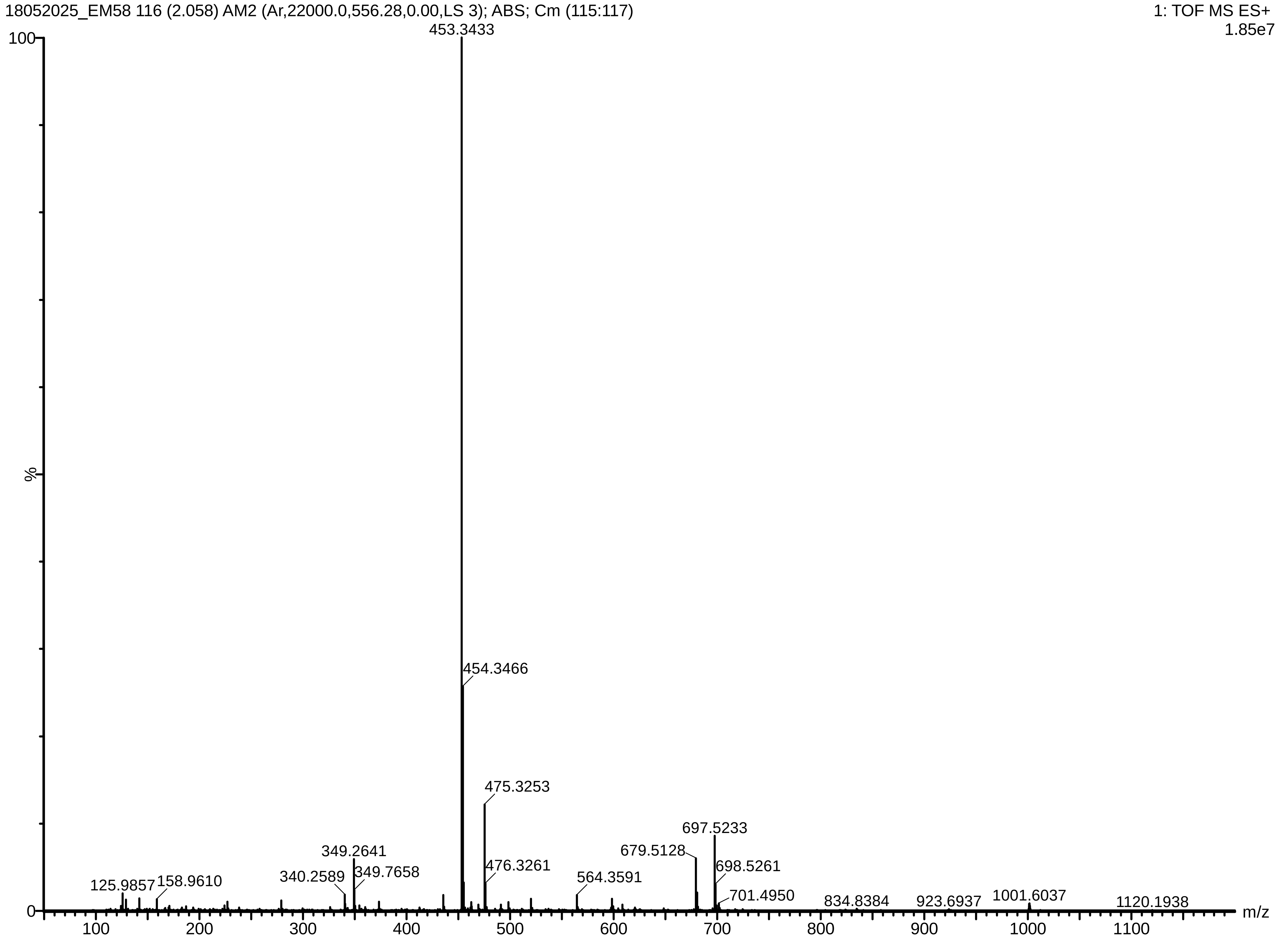
18052025_EM58 116 (2.058) AM2 (Ar,22000.0,556.28,0.00,LS 3); ABS; Cm (115:117)
1: TOF MS ES+
1.85e7
453.3433
454.3466
475.3253
697.5233
679.5128
349.2641
476.3261
698.5261
349.7658
564.3591
340.2589
158.9610
125.9857
701.4950
834.8384
923.6937
1001.6037
1120.1938
100
%
0
m/z
100
200
300
400
500
600
700
800
900
1000
1100
