# Supplementary material for: Metabolomic atlas of dengue virus infection reveals distinct circulating bioactive lipid signatures
Source: PLoS Negl Trop Dis. 2026 May 12;20(5):e0014327. doi: 10.1371/journal.pntd.0014327 (PMC13189415; doi:10.1371/journal.pntd.0014327)
Supplement: S1 Data — Electrospray ionisation time of flight mass spectrometry (ESI-TOF MS, positive mode) spectra of the dengue cohort and ESI-TOF at different retention times. The spectra display the relative abundance (%) of detected ions across the m/z range. Prominent peaks corresponding to major ionised species are indicated. Variation in spectral profiles between retention times reflects the differences in compound composition and ionisation patterns within the sample. Data were acquired under identical instrumental conditions and are presented as representative scans. (ZIP) [file pntd.0014327.s003.zip › EM COMPLETE SAMPLES SPECTRUM/EM58 SPECTRUM RT 2.565.pdf]

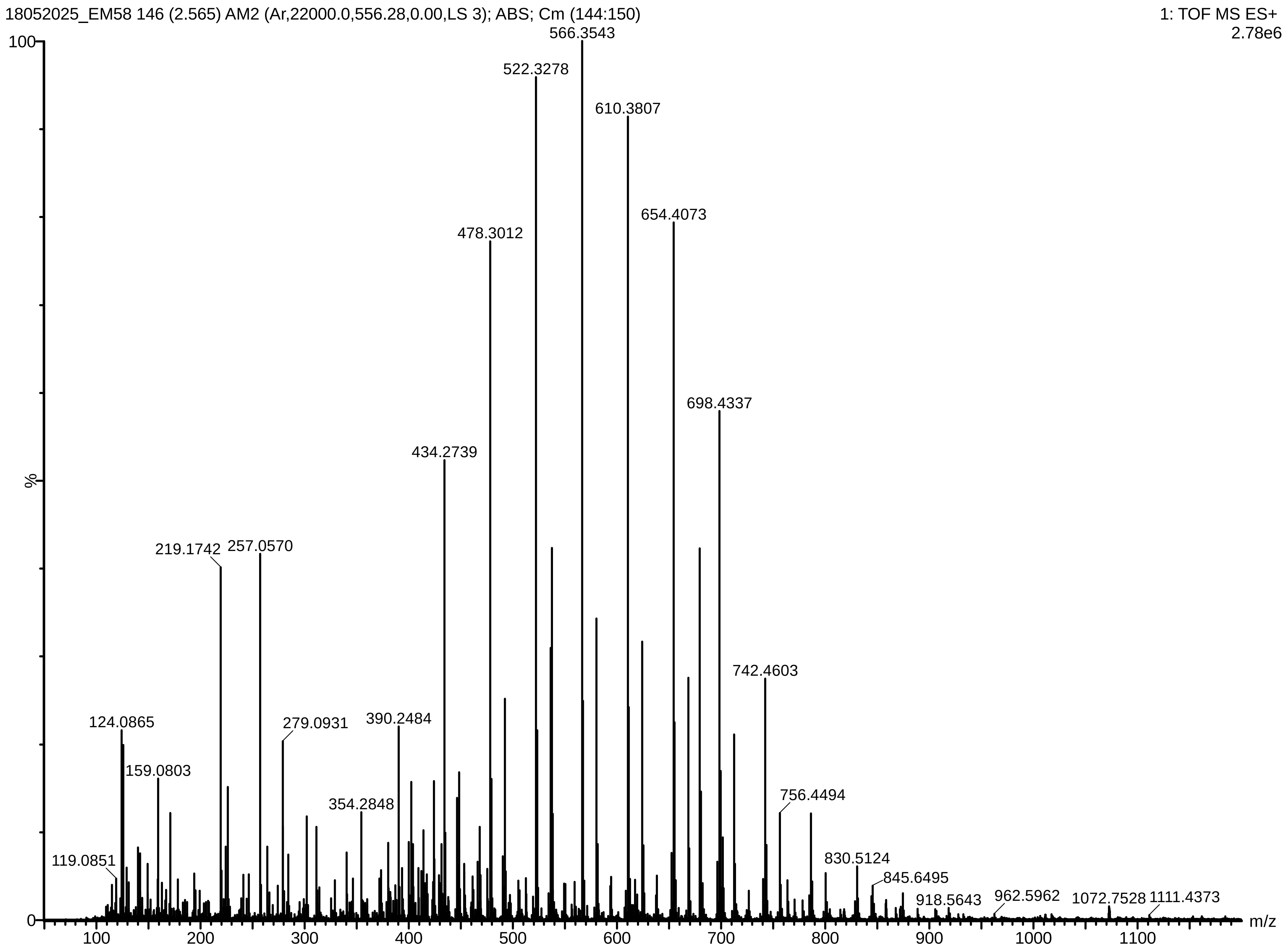
18052025_EM58 146 (2.565) AM2 (Ar,22000.0,556.28,0.00,LS 3); ABS; Cm (144:150)
1: TOF MS ES+
2.78e6
100
%
0
566.3543
522.3278
610.3807
654.4073
478.3012
698.4337
434.2739
219.1742
257.0570
742.4603
124.0865
279.0931
390.2484
159.0803
756.4494
354.2848
119.0851
830.5124
845.6495
918.5643
962.5962
1072.7528
1111.4373
100
200
300
400
500
600
700
800
900
1000
1100
m/z
