# Supplementary material for: Metabolomic atlas of dengue virus infection reveals distinct circulating bioactive lipid signatures
Source: PLoS Negl Trop Dis. 2026 May 12;20(5):e0014327. doi: 10.1371/journal.pntd.0014327 (PMC13189415; doi:10.1371/journal.pntd.0014327)
Supplement: S1 Data — Electrospray ionisation time of flight mass spectrometry (ESI-TOF MS, positive mode) spectra of the dengue cohort and ESI-TOF at different retention times. The spectra display the relative abundance (%) of detected ions across the m/z range. Prominent peaks corresponding to major ionised species are indicated. Variation in spectral profiles between retention times reflects the differences in compound composition and ionisation patterns within the sample. Data were acquired under identical instrumental conditions and are presented as representative scans. (ZIP) [file pntd.0014327.s003.zip › EM COMPLETE SAMPLES SPECTRUM/EM58 SPECTRUM RT 2.873.pdf]

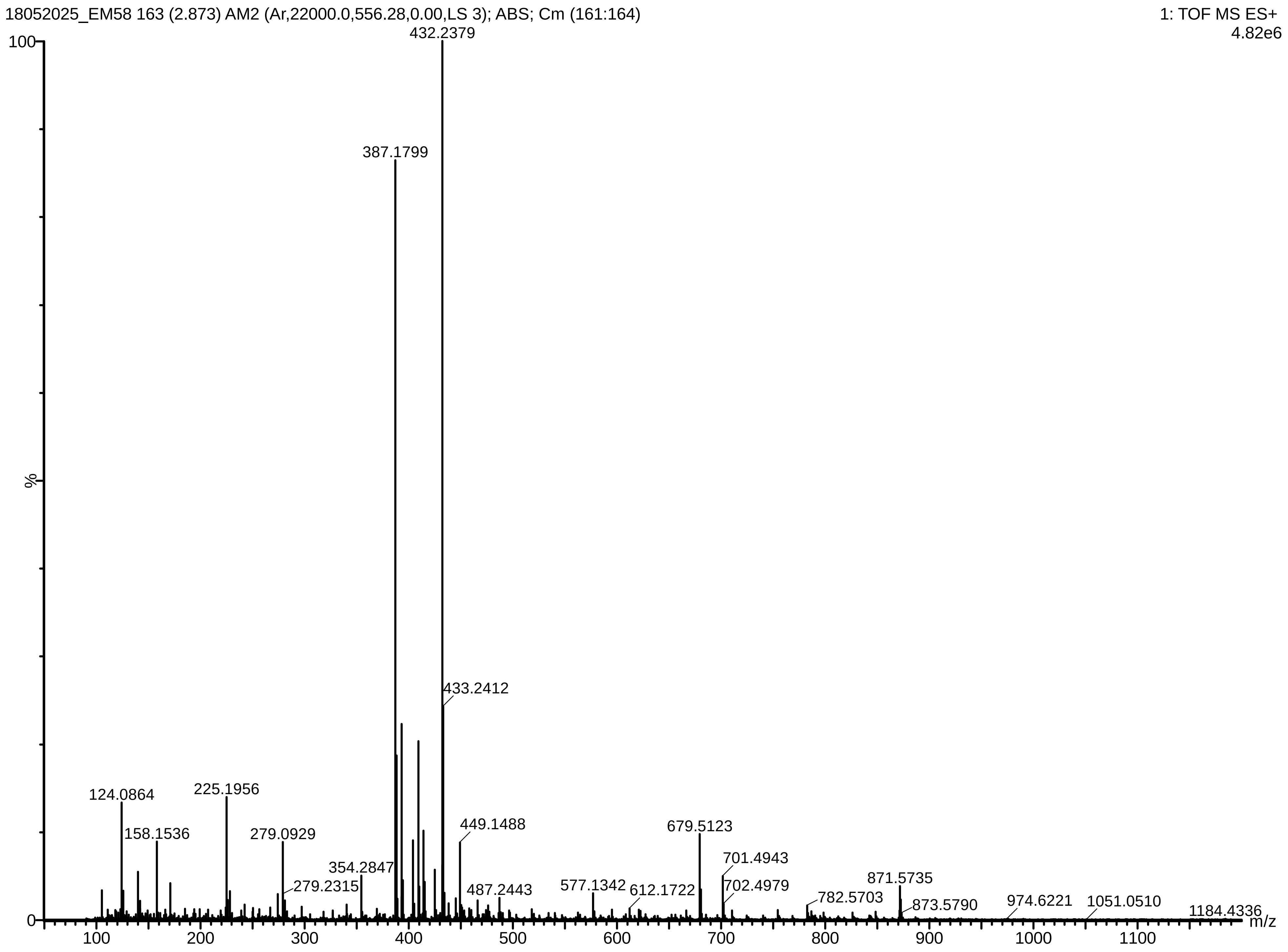
18052025_EM58 163 (2.873) AM2 (Ar,22000.0,556.28,0.00,LS 3); ABS; Cm (161:164)
1: TOF MS ES+
4.82e6
432.2379
387.1799
433.2412
124.0864
225.1956
158.1536
279.0929
279.2315
354.2847
449.1488
487.2443
577.1342
612.1722
679.5123
701.4943
702.4979
782.5703
871.5735
873.5790
974.6221
1051.0510
1184.4336
100
%
0
m/z
100
200
300
400
500
600
700
800
900
1000
1100
