# Supplementary material for: Metabolomic atlas of dengue virus infection reveals distinct circulating bioactive lipid signatures
Source: PLoS Negl Trop Dis. 2026 May 12;20(5):e0014327. doi: 10.1371/journal.pntd.0014327 (PMC13189415; doi:10.1371/journal.pntd.0014327)
Supplement: S1 Data — Electrospray ionisation time of flight mass spectrometry (ESI-TOF MS, positive mode) spectra of the dengue cohort and ESI-TOF at different retention times. The spectra display the relative abundance (%) of detected ions across the m/z range. Prominent peaks corresponding to major ionised species are indicated. Variation in spectral profiles between retention times reflects the differences in compound composition and ionisation patterns within the sample. Data were acquired under identical instrumental conditions and are presented as representative scans. (ZIP) [file pntd.0014327.s003.zip › EM COMPLETE SAMPLES SPECTRUM/EM58 SPECTRUM RT 3.279.pdf]

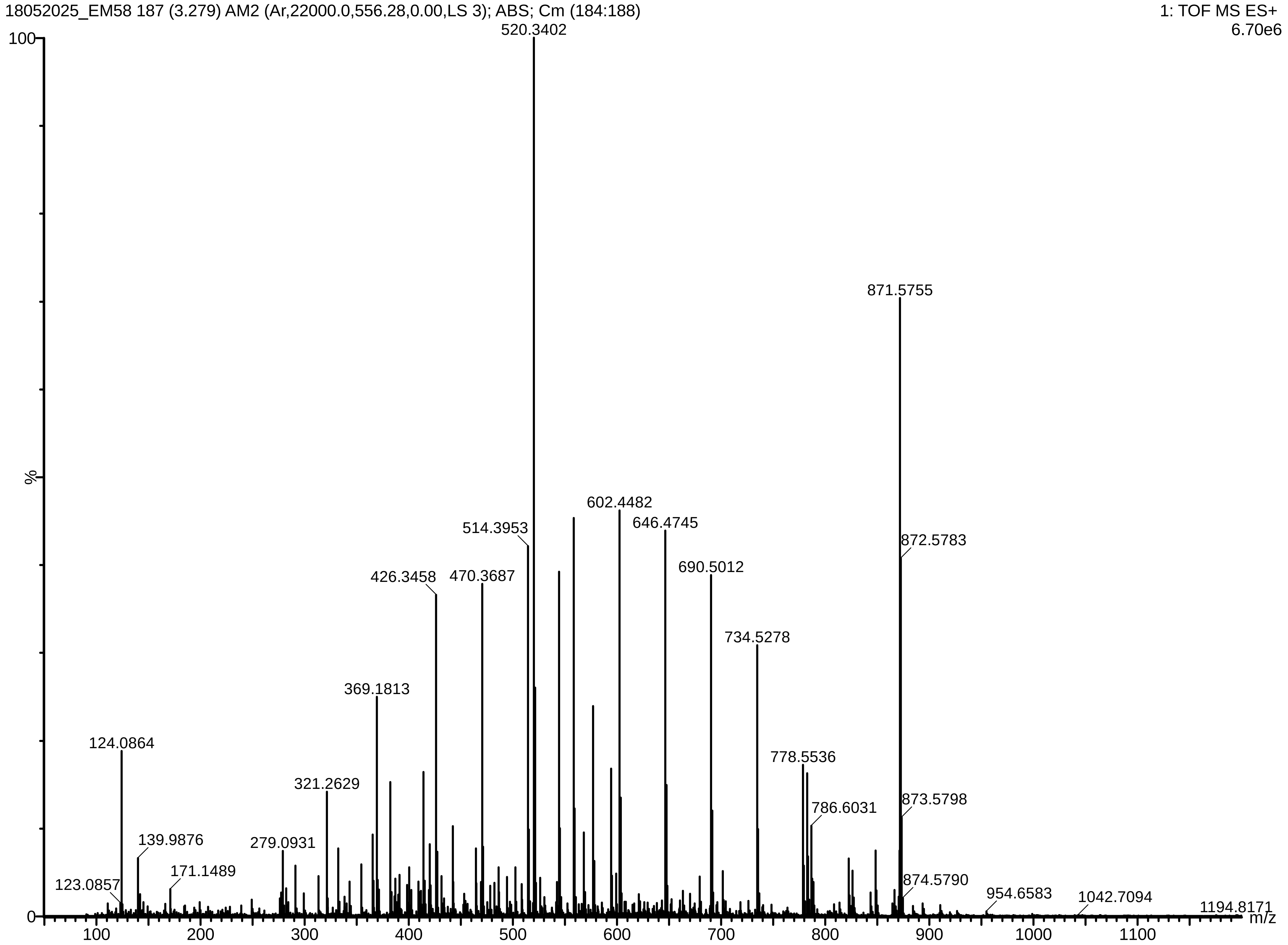

18052025_EM58 187 (3.279) AM2 (Ar,22000.0,556.28,0.00,LS 3); ABS; Cm (184:188)
1: TOF MS ES+
6.70e6
100
%
0
520.3402
871.5755
602.4482
646.4745
514.3953
872.5783
426.3458
470.3687
690.5012
734.5278
369.1813
124.0864
778.5536
321.2629
786.6031
873.5798
139.9876
279.0931
171.1489
874.5790
123.0857
954.6583
1042.7094
1194.8171
m/z
100
200
300
400
500
600
700
800
900
1000
1100
