# Supplementary material for: Metabolomic atlas of dengue virus infection reveals distinct circulating bioactive lipid signatures
Source: PLoS Negl Trop Dis. 2026 May 12;20(5):e0014327. doi: 10.1371/journal.pntd.0014327 (PMC13189415; doi:10.1371/journal.pntd.0014327)
Supplement: S1 Data — Electrospray ionisation time of flight mass spectrometry (ESI-TOF MS, positive mode) spectra of the dengue cohort and ESI-TOF at different retention times. The spectra display the relative abundance (%) of detected ions across the m/z range. Prominent peaks corresponding to major ionised species are indicated. Variation in spectral profiles between retention times reflects the differences in compound composition and ionisation patterns within the sample. Data were acquired under identical instrumental conditions and are presented as representative scans. (ZIP) [file pntd.0014327.s003.zip › EM COMPLETE SAMPLES SPECTRUM/EM58 SPECTRUM RT 3.434.pdf]

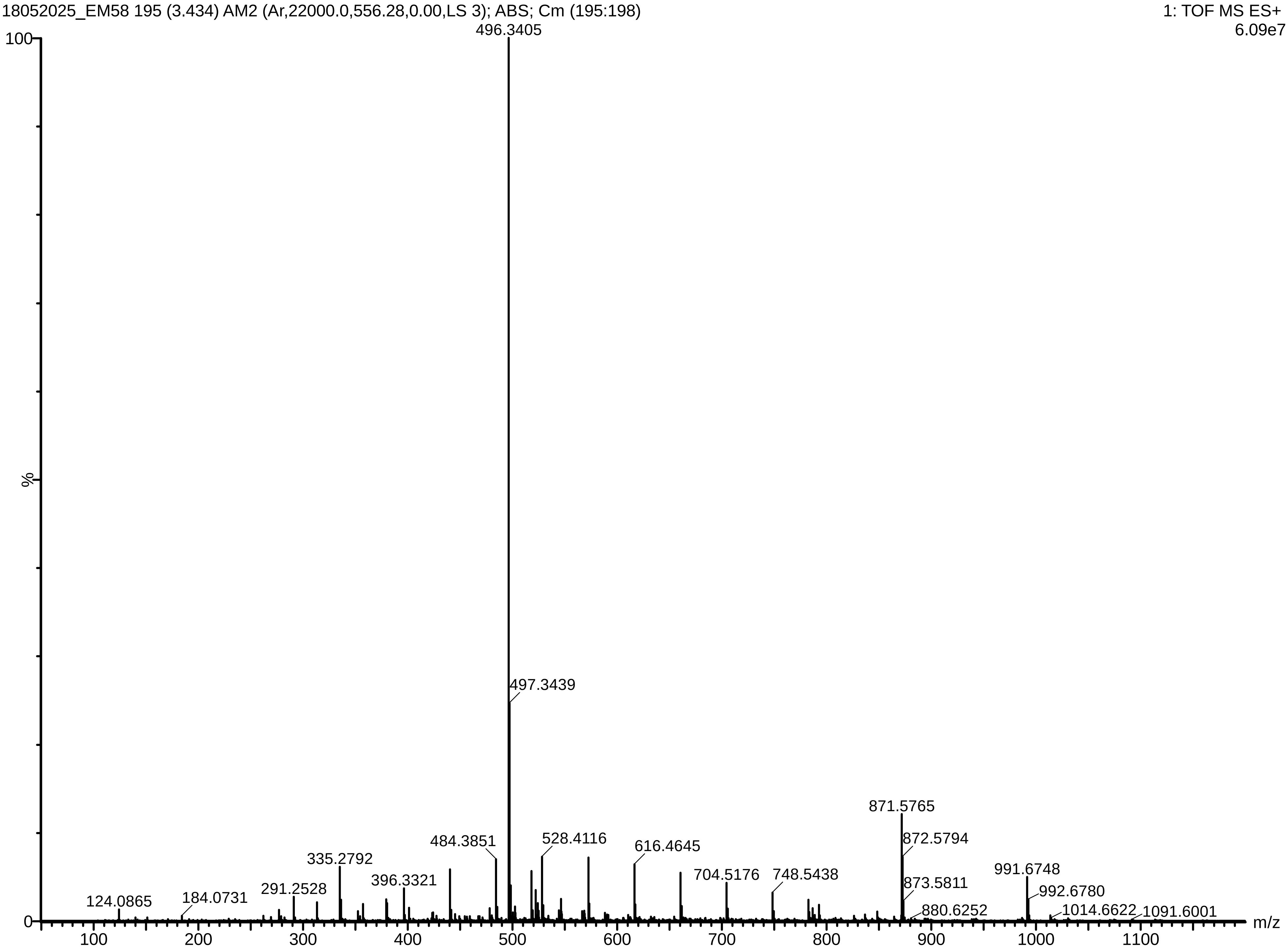
18052025_EM58 195 (3.434) AM2 (Ar,22000.0,556.28,0.00,LS 3); ABS; Cm (195:198)
1: TOF MS ES+
6.09e7
496.3405
497.3439
871.5765
872.5794
873.5811
880.6252
484.3851
528.4116
616.4645
335.2792
396.3321
291.2528
704.5176
748.5438
991.6748
992.6780
1014.6622
1091.6001
124.0865
184.0731
100
%
0
m/z
100
200
300
400
500
600
700
800
900
1000
1100
