# Supplementary material for: Metabolomic atlas of dengue virus infection reveals distinct circulating bioactive lipid signatures
Source: PLoS Negl Trop Dis. 2026 May 12;20(5):e0014327. doi: 10.1371/journal.pntd.0014327 (PMC13189415; doi:10.1371/journal.pntd.0014327)
Supplement: S1 Data — Electrospray ionisation time of flight mass spectrometry (ESI-TOF MS, positive mode) spectra of the dengue cohort and ESI-TOF at different retention times. The spectra display the relative abundance (%) of detected ions across the m/z range. Prominent peaks corresponding to major ionised species are indicated. Variation in spectral profiles between retention times reflects the differences in compound composition and ionisation patterns within the sample. Data were acquired under identical instrumental conditions and are presented as representative scans. (ZIP) [file pntd.0014327.s003.zip › EM COMPLETE SAMPLES SPECTRUM/EM58 SPECTRUM RT 3.840.pdf]

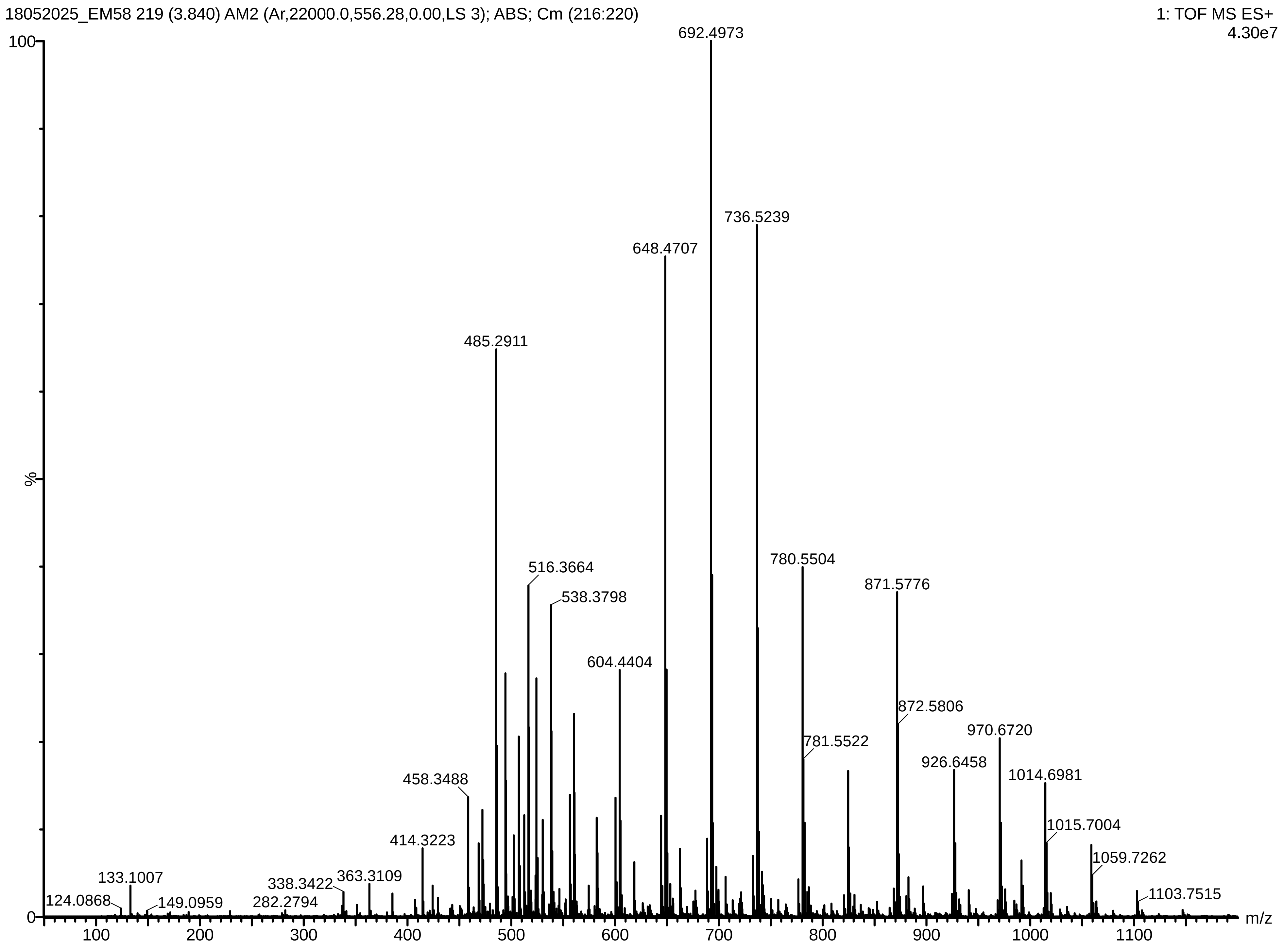
18052025_EM58 219 (3.840) AM2 (Ar,22000.0,556.28,0.00,LS 3); ABS; Cm (216:220)
1: TOF MS ES+
4.30e7
100
%
0
692.4973
736.5239
648.4707
485.2911
516.3664
538.3798
604.4404
780.5504
871.5776
872.5806
781.5522
970.6720
926.6458
1014.6981
458.3488
1015.7004
414.3223
1059.7262
133.1007
338.3422
363.3109
124.0868
149.0959
282.2794
1103.7515
100
200
300
400
500
600
700
800
900
1000
1100
m/z
