# Supplementary material for: Metabolomic atlas of dengue virus infection reveals distinct circulating bioactive lipid signatures
Source: PLoS Negl Trop Dis. 2026 May 12;20(5):e0014327. doi: 10.1371/journal.pntd.0014327 (PMC13189415; doi:10.1371/journal.pntd.0014327)
Supplement: S1 Data — Electrospray ionisation time of flight mass spectrometry (ESI-TOF MS, positive mode) spectra of the dengue cohort and ESI-TOF at different retention times. The spectra display the relative abundance (%) of detected ions across the m/z range. Prominent peaks corresponding to major ionised species are indicated. Variation in spectral profiles between retention times reflects the differences in compound composition and ionisation patterns within the sample. Data were acquired under identical instrumental conditions and are presented as representative scans. (ZIP) [file pntd.0014327.s003.zip › EM COMPLETE SAMPLES SPECTRUM/EM62 SPECTRUM RT 0.459.pdf]

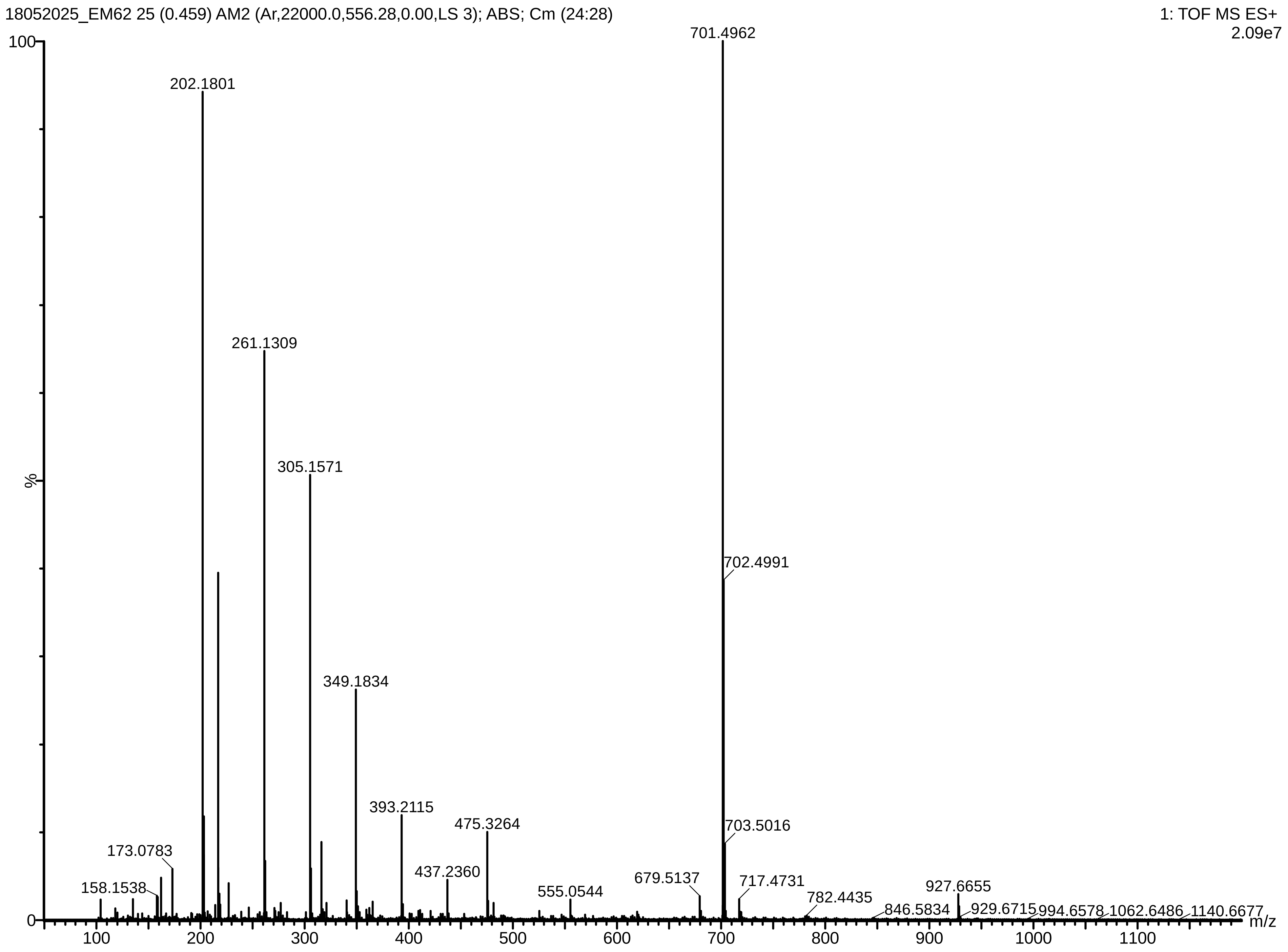
18052025_EM62 25 (0.459) AM2 (Ar,22000.0,556.28,0.00,LS 3); ABS; Cm (24:28)
1: TOF MS ES+
2.09e7
100
%
0
701.4962
202.1801
261.1309
305.1571
702.4991
349.1834
393.2115
475.3264
703.5016
173.0783
437.2360
158.1538
679.5137
717.4731
555.0544
927.6655
782.4435
846.5834
929.6715
994.6578
1062.6486
1140.6677
m/z
100
200
300
400
500
600
700
800
900
1000
1100
