# Supplementary material for: Metabolomic atlas of dengue virus infection reveals distinct circulating bioactive lipid signatures
Source: PLoS Negl Trop Dis. 2026 May 12;20(5):e0014327. doi: 10.1371/journal.pntd.0014327 (PMC13189415; doi:10.1371/journal.pntd.0014327)
Supplement: S1 Data — Electrospray ionisation time of flight mass spectrometry (ESI-TOF MS, positive mode) spectra of the dengue cohort and ESI-TOF at different retention times. The spectra display the relative abundance (%) of detected ions across the m/z range. Prominent peaks corresponding to major ionised species are indicated. Variation in spectral profiles between retention times reflects the differences in compound composition and ionisation patterns within the sample. Data were acquired under identical instrumental conditions and are presented as representative scans. (ZIP) [file pntd.0014327.s003.zip › EM COMPLETE SAMPLES SPECTRUM/EM62 SPECTRUM RT 0.679.pdf]

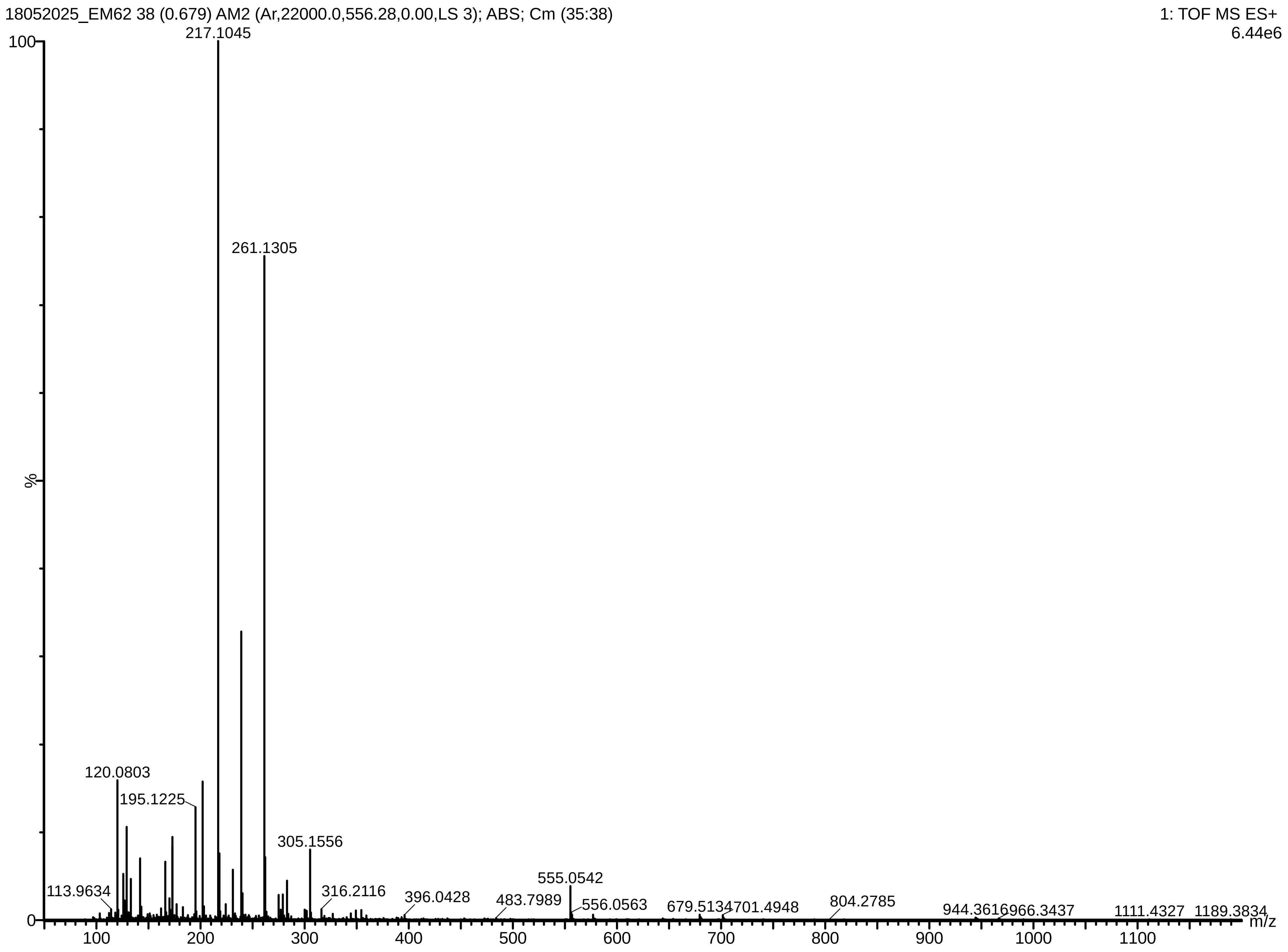
18052025_EM62 38 (0.679) AM2 (Ar,22000.0,556.28,0.00,LS 3); ABS; Cm (35:38)
1: TOF MS ES+
6.44e6
217.1045
261.1305
120.0803
195.1225
305.1556
113.9634
316.2116
396.0428
483.7989
555.0542
556.0563
679.5134
701.4948
804.2785
944.3616
966.3437
1111.4327
1189.3834
100
%
0
m/z
100
200
300
400
500
600
700
800
900
1000
1100
