# Supplementary material for: Metabolomic atlas of dengue virus infection reveals distinct circulating bioactive lipid signatures
Source: PLoS Negl Trop Dis. 2026 May 12;20(5):e0014327. doi: 10.1371/journal.pntd.0014327 (PMC13189415; doi:10.1371/journal.pntd.0014327)
Supplement: S1 Data — Electrospray ionisation time of flight mass spectrometry (ESI-TOF MS, positive mode) spectra of the dengue cohort and ESI-TOF at different retention times. The spectra display the relative abundance (%) of detected ions across the m/z range. Prominent peaks corresponding to major ionised species are indicated. Variation in spectral profiles between retention times reflects the differences in compound composition and ionisation patterns within the sample. Data were acquired under identical instrumental conditions and are presented as representative scans. (ZIP) [file pntd.0014327.s003.zip › EM COMPLETE SAMPLES SPECTRUM/EM62 SPECTRUM RT 2.058.pdf]

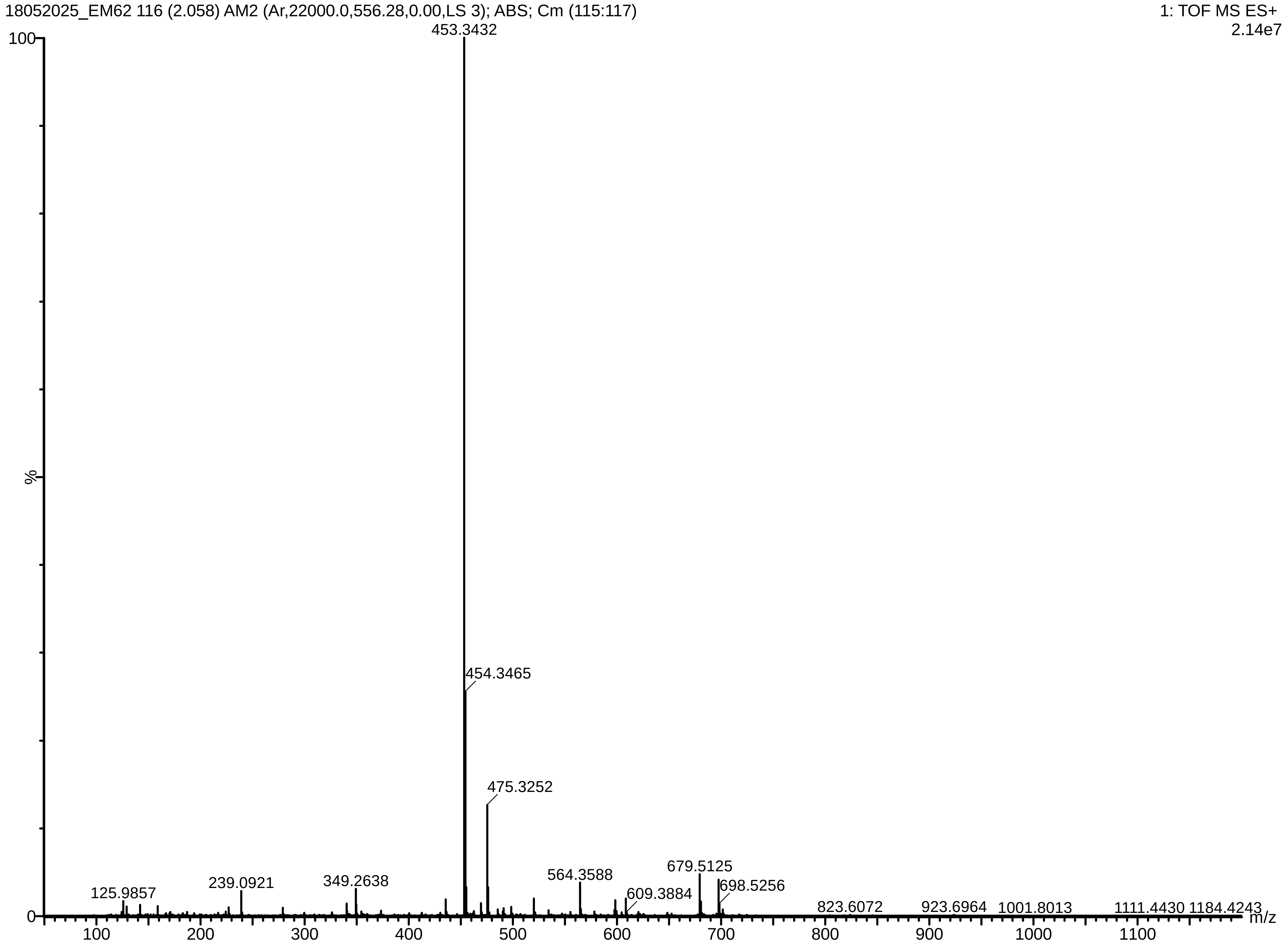
18052025_EM62 116 (2.058) AM2 (Ar,22000.0,556.28,0.00,LS 3); ABS; Cm (115:117)
1: TOF MS ES+
2.14e7
453.3432
454.3465
475.3252
125.9857
239.0921
349.2638
564.3588
609.3884
679.5125
698.5256
823.6072
923.6964
1001.8013
1111.4430
1184.4243
%
100
0
m/z
100
200
300
400
500
600
700
800
900
1000
1100
