# Supplementary material for: Metabolomic atlas of dengue virus infection reveals distinct circulating bioactive lipid signatures
Source: PLoS Negl Trop Dis. 2026 May 12;20(5):e0014327. doi: 10.1371/journal.pntd.0014327 (PMC13189415; doi:10.1371/journal.pntd.0014327)
Supplement: S1 Data — Electrospray ionisation time of flight mass spectrometry (ESI-TOF MS, positive mode) spectra of the dengue cohort and ESI-TOF at different retention times. The spectra display the relative abundance (%) of detected ions across the m/z range. Prominent peaks corresponding to major ionised species are indicated. Variation in spectral profiles between retention times reflects the differences in compound composition and ionisation patterns within the sample. Data were acquired under identical instrumental conditions and are presented as representative scans. (ZIP) [file pntd.0014327.s003.zip › EM COMPLETE SAMPLES SPECTRUM/EM62 SPECTRUM RT 2.565.pdf]

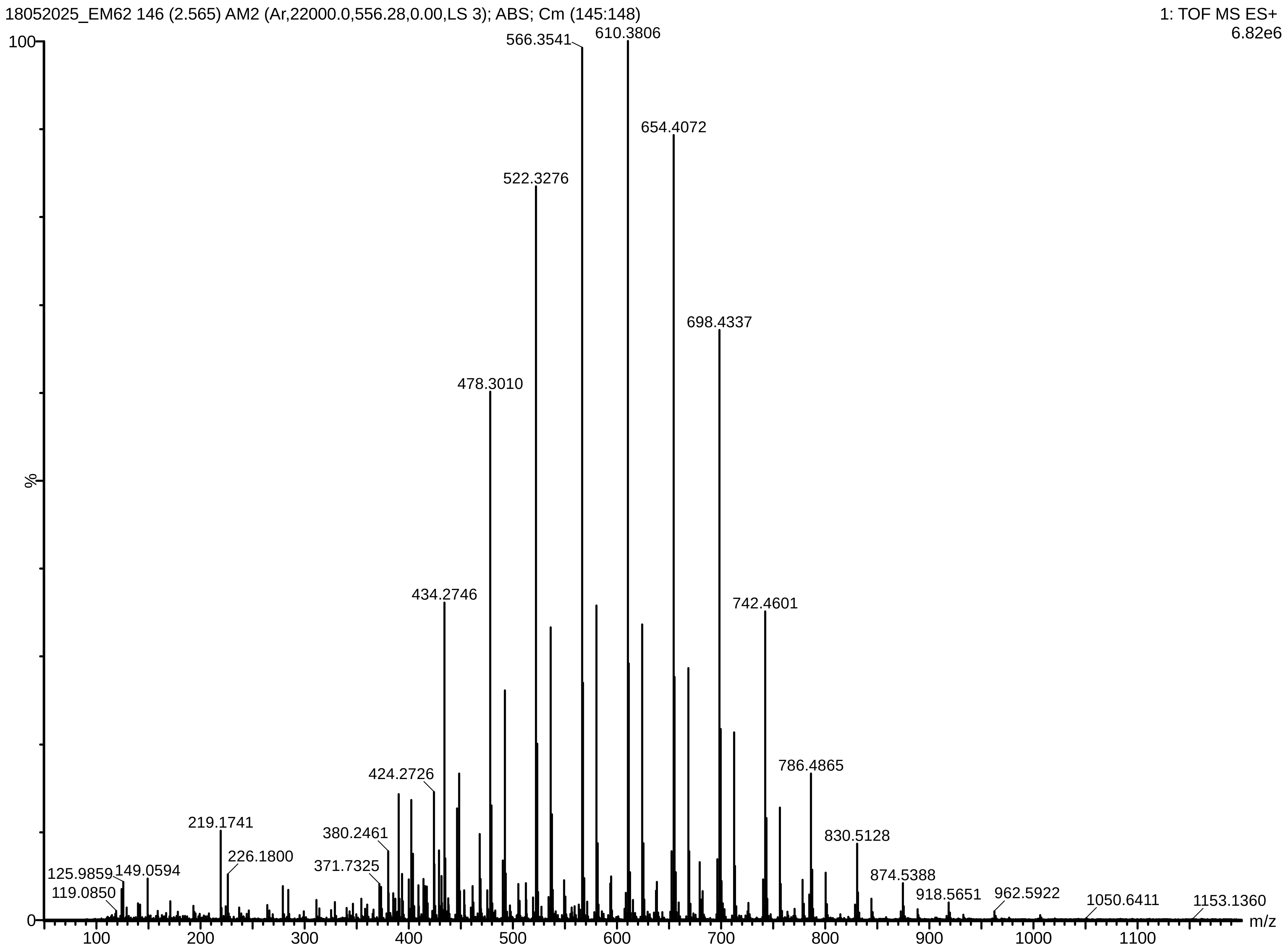
18052025_EM62 146 (2.565) AM2 (Ar,22000.0,556.28,0.00,LS 3); ABS; Cm (145:148)
1: TOF MS ES+
6.82e6
%
m/z
566.3541
610.3806
654.4072
522.3276
698.4337
478.3010
434.2746
742.4601
786.4865
424.2726
830.5128
219.1741
380.2461
226.1800
371.7325
874.5388
125.9859
149.0594
119.0850
918.5651
962.5922
1050.6411
1153.1360
100
0
100
200
300
400
500
600
700
800
900
1000
1100
