# Supplementary material for: Metabolomic atlas of dengue virus infection reveals distinct circulating bioactive lipid signatures
Source: PLoS Negl Trop Dis. 2026 May 12;20(5):e0014327. doi: 10.1371/journal.pntd.0014327 (PMC13189415; doi:10.1371/journal.pntd.0014327)
Supplement: S1 Data — Electrospray ionisation time of flight mass spectrometry (ESI-TOF MS, positive mode) spectra of the dengue cohort and ESI-TOF at different retention times. The spectra display the relative abundance (%) of detected ions across the m/z range. Prominent peaks corresponding to major ionised species are indicated. Variation in spectral profiles between retention times reflects the differences in compound composition and ionisation patterns within the sample. Data were acquired under identical instrumental conditions and are presented as representative scans. (ZIP) [file pntd.0014327.s003.zip › EM COMPLETE SAMPLES SPECTRUM/EM62 SPECTRUM RT 2.856.pdf]

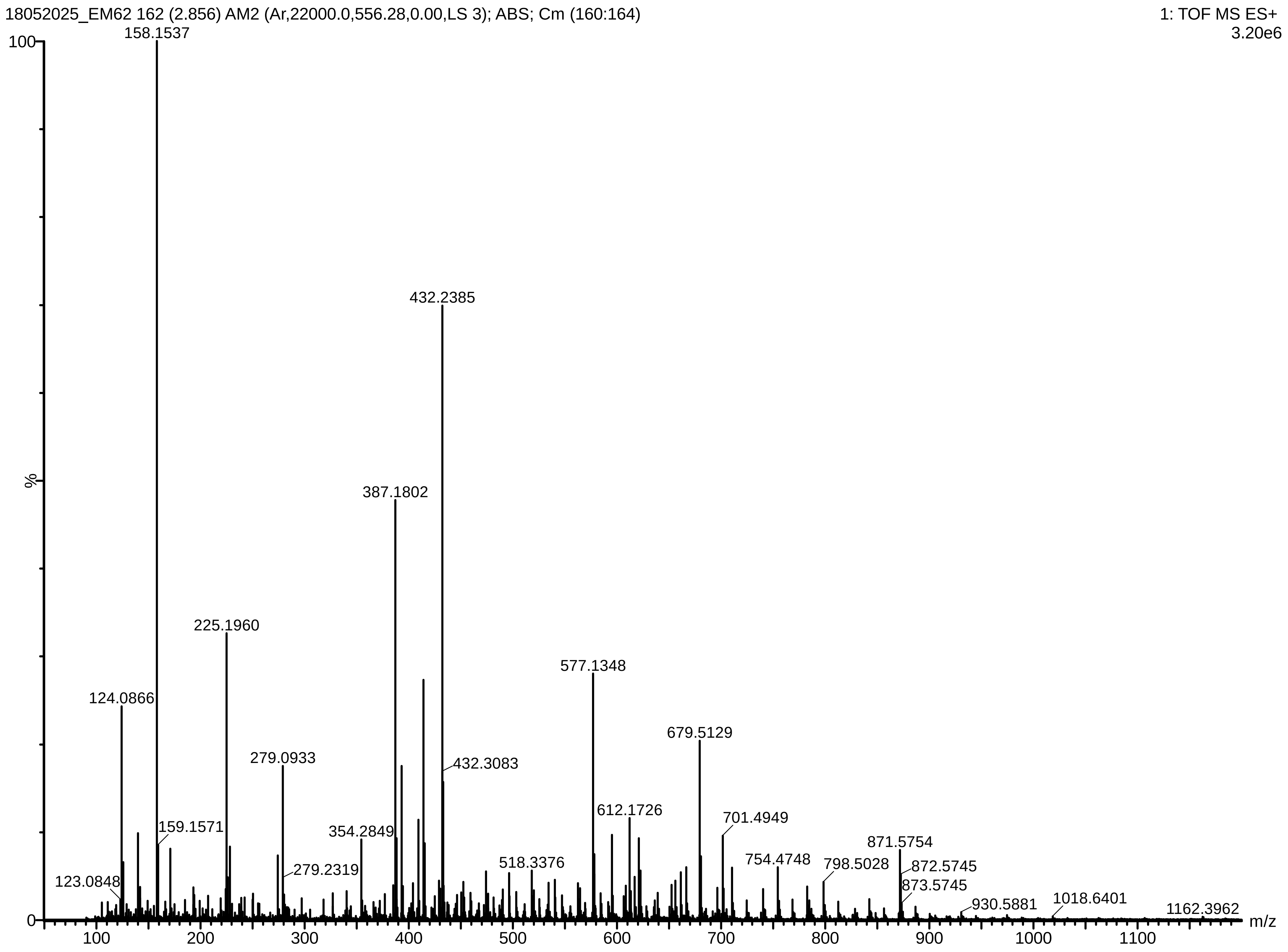

18052025_EM62 162 (2.856) AM2 (Ar,22000.0,556.28,0.00,LS 3); ABS; Cm (160:164)
1: TOF MS ES+
3.20e6
100
%
0
158.1537
432.2385
387.1802
225.1960
577.1348
124.0866
679.5129
279.0933
432.3083
612.1726
159.1571
701.4949
354.2849
871.5754
754.4748
798.5028
872.5745
518.3376
279.2319
123.0848
873.5745
930.5881
1018.6401
1162.3962
100
200
300
400
500
600
700
800
900
1000
1100
m/z
