# Supplementary material for: Metabolomic atlas of dengue virus infection reveals distinct circulating bioactive lipid signatures
Source: PLoS Negl Trop Dis. 2026 May 12;20(5):e0014327. doi: 10.1371/journal.pntd.0014327 (PMC13189415; doi:10.1371/journal.pntd.0014327)
Supplement: S1 Data — Electrospray ionisation time of flight mass spectrometry (ESI-TOF MS, positive mode) spectra of the dengue cohort and ESI-TOF at different retention times. The spectra display the relative abundance (%) of detected ions across the m/z range. Prominent peaks corresponding to major ionised species are indicated. Variation in spectral profiles between retention times reflects the differences in compound composition and ionisation patterns within the sample. Data were acquired under identical instrumental conditions and are presented as representative scans. (ZIP) [file pntd.0014327.s003.zip › EM COMPLETE SAMPLES SPECTRUM/EM62 SPECTRUM RT 3.279.pdf]

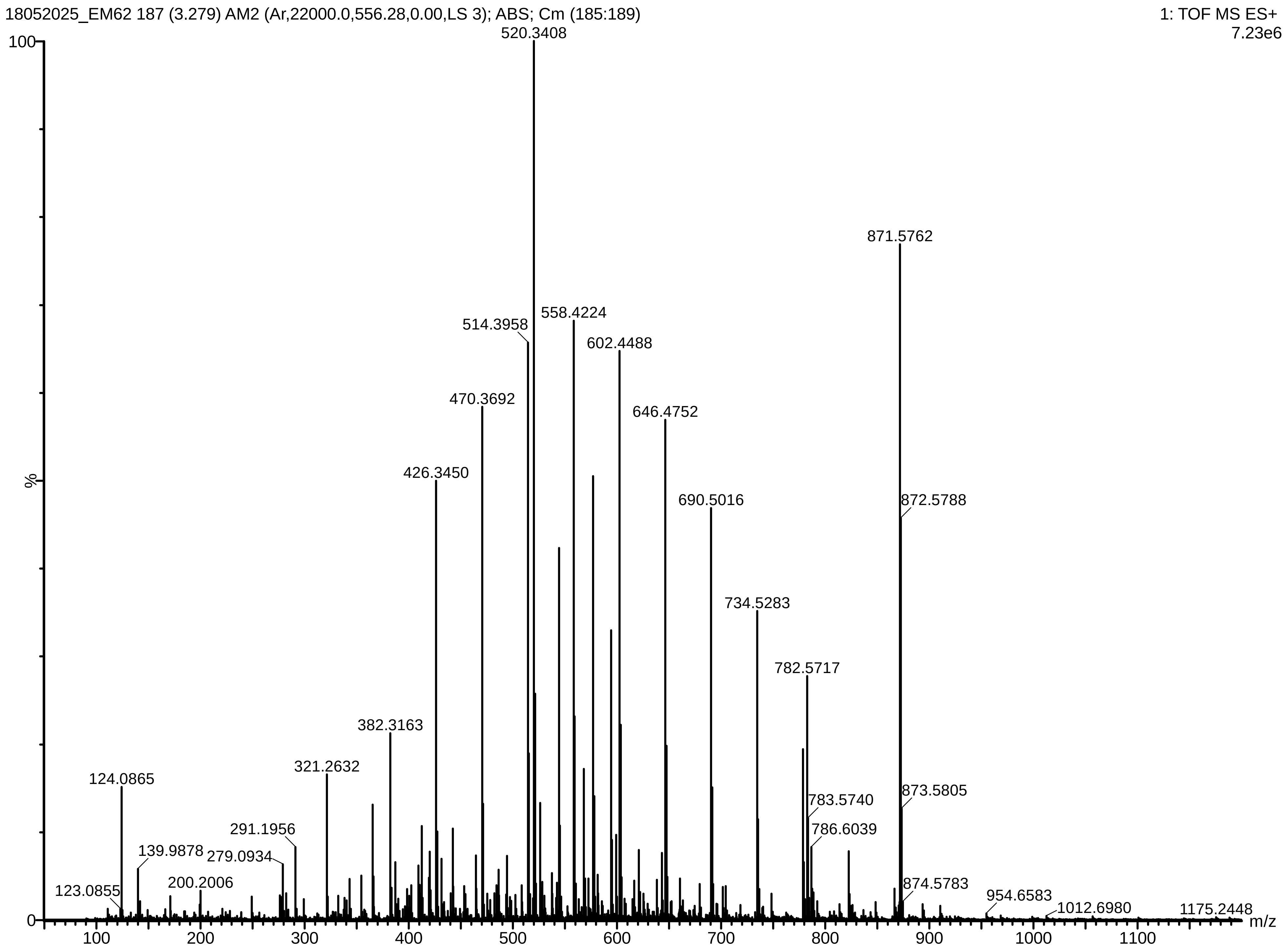
18052025_EM62 187 (3.279) AM2 (Ar,22000.0,556.28,0.00,LS 3); ABS; Cm (185:189)
1: TOF MS ES+
7.23e6
520.3408
871.5762
514.3958
558.4224
602.4488
470.3692
646.4752
426.3450
690.5016
872.5788
734.5283
782.5717
382.3163
124.0865
321.2632
873.5805
783.5740
291.1956
786.6039
139.9878
279.0934
200.2006
123.0855
874.5783
954.6583
1012.6980
1175.2448
%
m/z
100
0
100
200
300
400
500
600
700
800
900
1000
1100
