# Supplementary material for: Metabolomic atlas of dengue virus infection reveals distinct circulating bioactive lipid signatures
Source: PLoS Negl Trop Dis. 2026 May 12;20(5):e0014327. doi: 10.1371/journal.pntd.0014327 (PMC13189415; doi:10.1371/journal.pntd.0014327)
Supplement: S1 Data — Electrospray ionisation time of flight mass spectrometry (ESI-TOF MS, positive mode) spectra of the dengue cohort and ESI-TOF at different retention times. The spectra display the relative abundance (%) of detected ions across the m/z range. Prominent peaks corresponding to major ionised species are indicated. Variation in spectral profiles between retention times reflects the differences in compound composition and ionisation patterns within the sample. Data were acquired under identical instrumental conditions and are presented as representative scans. (ZIP) [file pntd.0014327.s003.zip › EM COMPLETE SAMPLES SPECTRUM/EM62 SPECTRUM RT 3.434.pdf]

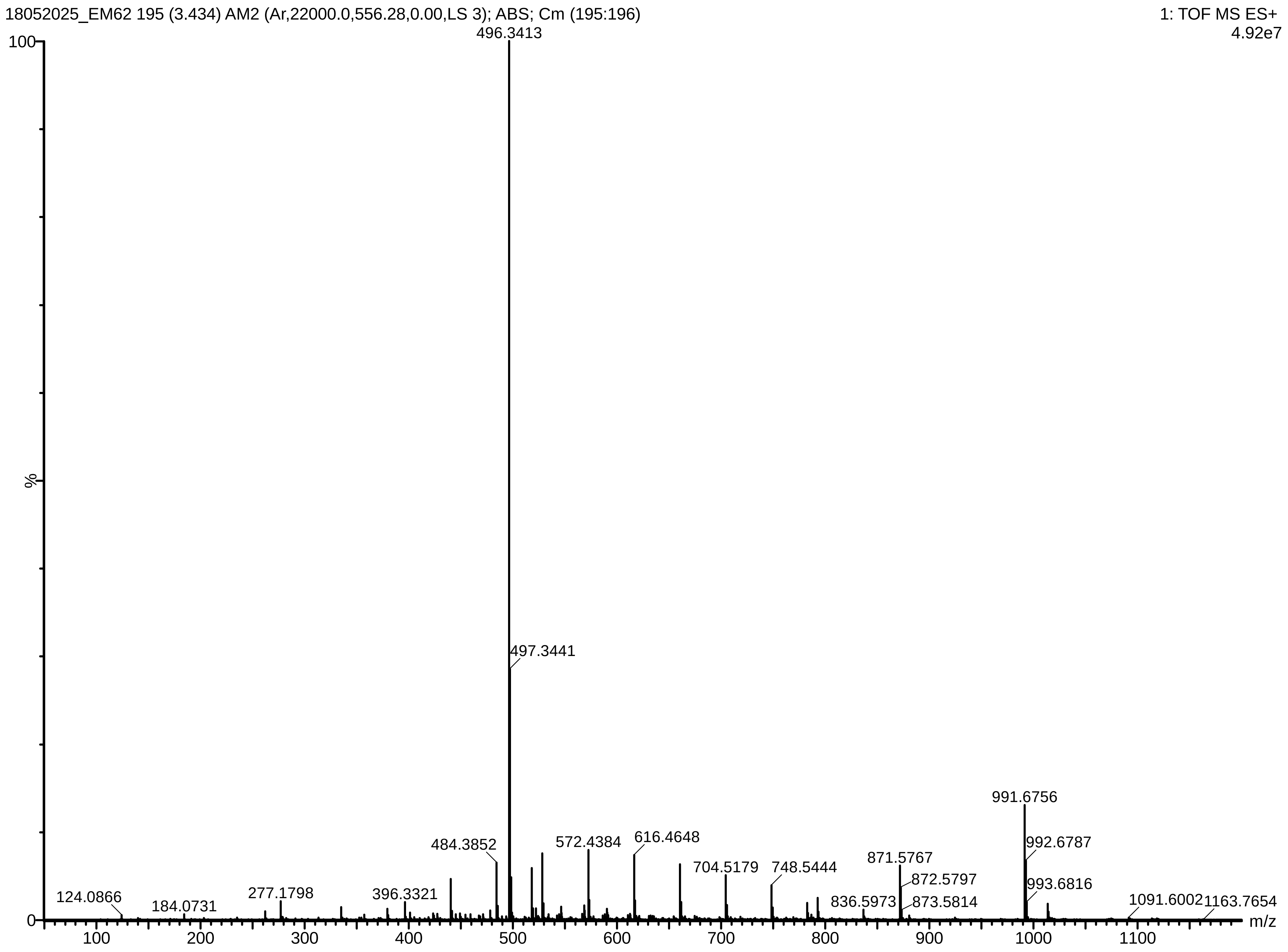
18052025_EM62 195 (3.434) AM2 (Ar,22000.0,556.28,0.00,LS 3); ABS; Cm (195:196)
1: TOF MS ES+
4.92e7
100
%
0
496.3413
497.3441
991.6756
992.6787
993.6816
484.3852
572.4384
616.4648
704.5179
748.5444
871.5767
872.5797
873.5814
836.5973
124.0866
184.0731
277.1798
396.3321
1091.6002
1163.7654
100
200
300
400
500
600
700
800
900
1000
1100
m/z
