# Supplementary material for: Metabolomic atlas of dengue virus infection reveals distinct circulating bioactive lipid signatures
Source: PLoS Negl Trop Dis. 2026 May 12;20(5):e0014327. doi: 10.1371/journal.pntd.0014327 (PMC13189415; doi:10.1371/journal.pntd.0014327)
Supplement: S1 Data — Electrospray ionisation time of flight mass spectrometry (ESI-TOF MS, positive mode) spectra of the dengue cohort and ESI-TOF at different retention times. The spectra display the relative abundance (%) of detected ions across the m/z range. Prominent peaks corresponding to major ionised species are indicated. Variation in spectral profiles between retention times reflects the differences in compound composition and ionisation patterns within the sample. Data were acquired under identical instrumental conditions and are presented as representative scans. (ZIP) [file pntd.0014327.s003.zip › EM COMPLETE SAMPLES SPECTRUM/EM62 SPECTRUM RT 3.806.pdf]

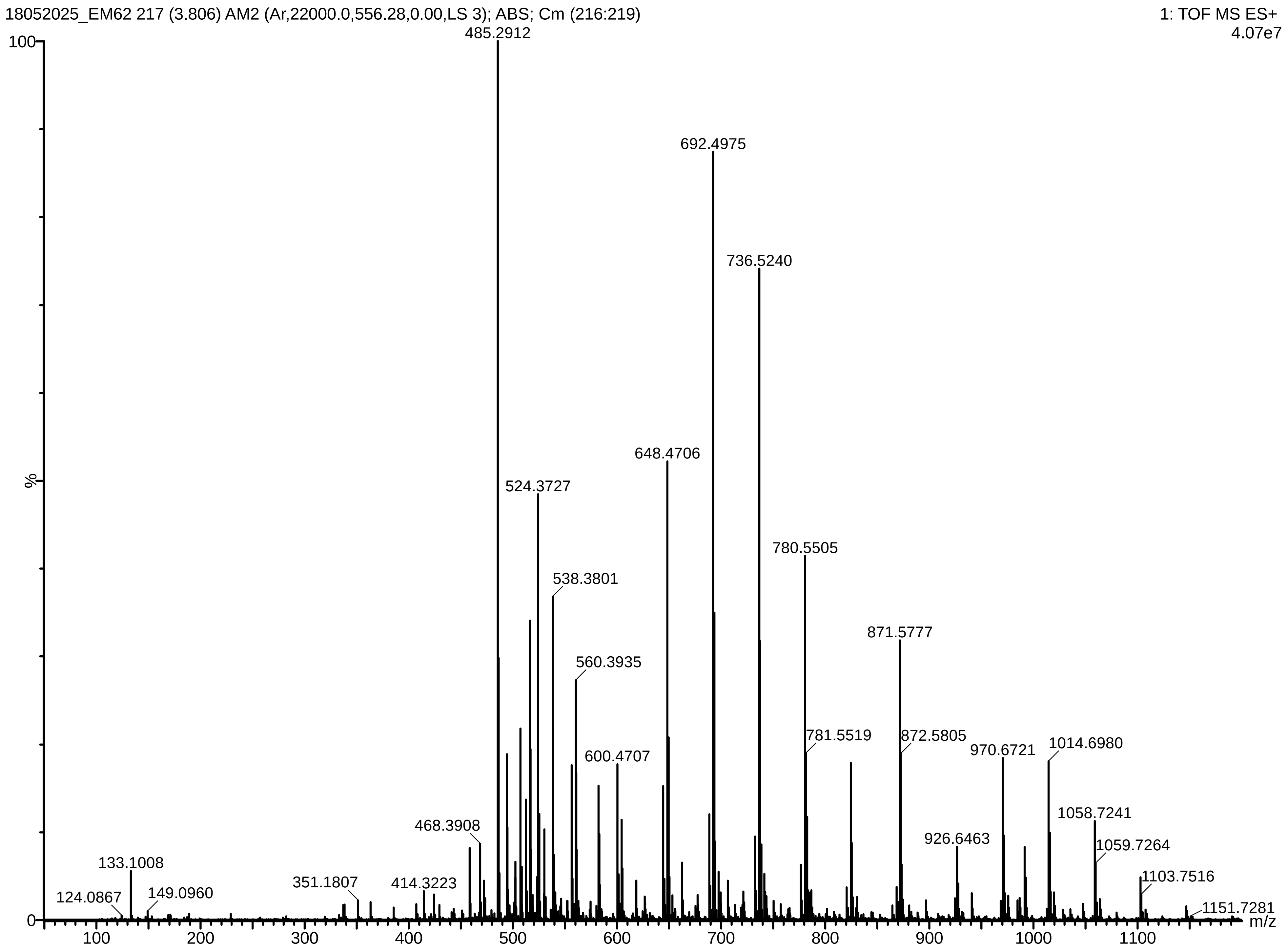

18052025_EM62 217 (3.806) AM2 (Ar,22000.0,556.28,0.00,LS 3); ABS; Cm (216:219)
1: TOF MS ES+
4.07e7
100
%
0
485.2912
692.4975
736.5240
648.4706
524.3727
780.5505
538.3801
871.5777
560.3935
781.5519
872.5805
600.4707
970.6721
1014.6980
468.3908
1058.7241
926.6463
1059.7264
133.1008
351.1807
414.3223
1103.7516
124.0867
149.0960
1151.7281
m/z
100
200
300
400
500
600
700
800
900
1000
1100
