# Supplementary material for: Metabolomic atlas of dengue virus infection reveals distinct circulating bioactive lipid signatures
Source: PLoS Negl Trop Dis. 2026 May 12;20(5):e0014327. doi: 10.1371/journal.pntd.0014327 (PMC13189415; doi:10.1371/journal.pntd.0014327)
Supplement: S1 Data — Electrospray ionisation time of flight mass spectrometry (ESI-TOF MS, positive mode) spectra of the dengue cohort and ESI-TOF at different retention times. The spectra display the relative abundance (%) of detected ions across the m/z range. Prominent peaks corresponding to major ionised species are indicated. Variation in spectral profiles between retention times reflects the differences in compound composition and ionisation patterns within the sample. Data were acquired under identical instrumental conditions and are presented as representative scans. (ZIP) [file pntd.0014327.s003.zip › EM COMPLETE SAMPLES SPECTRUM/EM7 SPECTRUM RT 0.510.pdf]

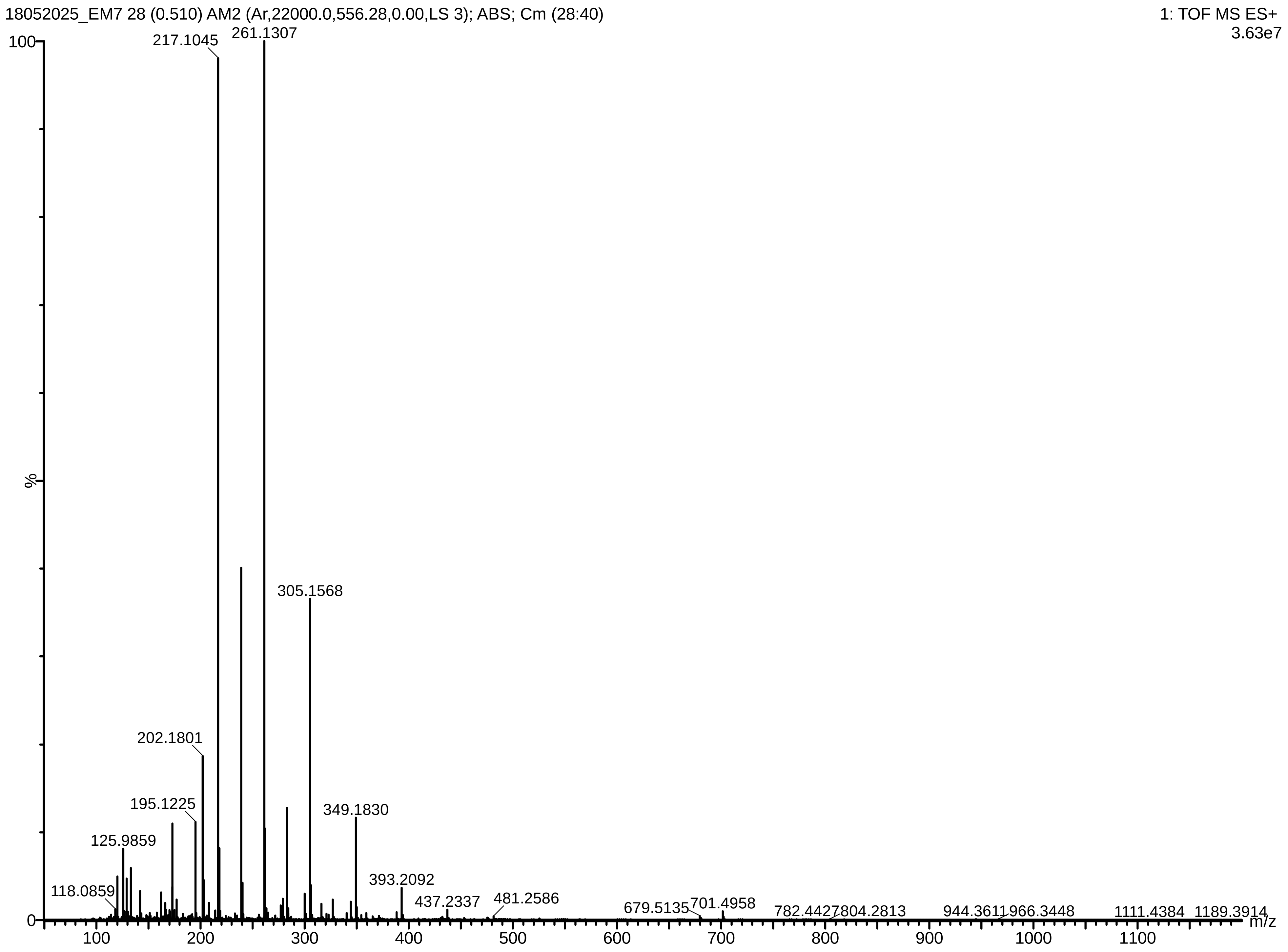

18052025_EM7 28 (0.510) AM2 (Ar,22000.0,556.28,0.00,LS 3); ABS; Cm (28:40)
1: TOF MS ES+
3.63e7
%
217.1045
261.1307
305.1568
202.1801
195.1225
349.1830
125.9859
118.0859
393.2092
437.2337
481.2586
679.5135
701.4958
782.4427
804.2813
944.3611
966.3448
1111.4384
1189.3914
m/z
