# Supplementary material for: Metabolomic atlas of dengue virus infection reveals distinct circulating bioactive lipid signatures
Source: PLoS Negl Trop Dis. 2026 May 12;20(5):e0014327. doi: 10.1371/journal.pntd.0014327 (PMC13189415; doi:10.1371/journal.pntd.0014327)
Supplement: S1 Data — Electrospray ionisation time of flight mass spectrometry (ESI-TOF MS, positive mode) spectra of the dengue cohort and ESI-TOF at different retention times. The spectra display the relative abundance (%) of detected ions across the m/z range. Prominent peaks corresponding to major ionised species are indicated. Variation in spectral profiles between retention times reflects the differences in compound composition and ionisation patterns within the sample. Data were acquired under identical instrumental conditions and are presented as representative scans. (ZIP) [file pntd.0014327.s003.zip › EM COMPLETE SAMPLES SPECTRUM/EM7 SPECTRUM RT 0.679.pdf]

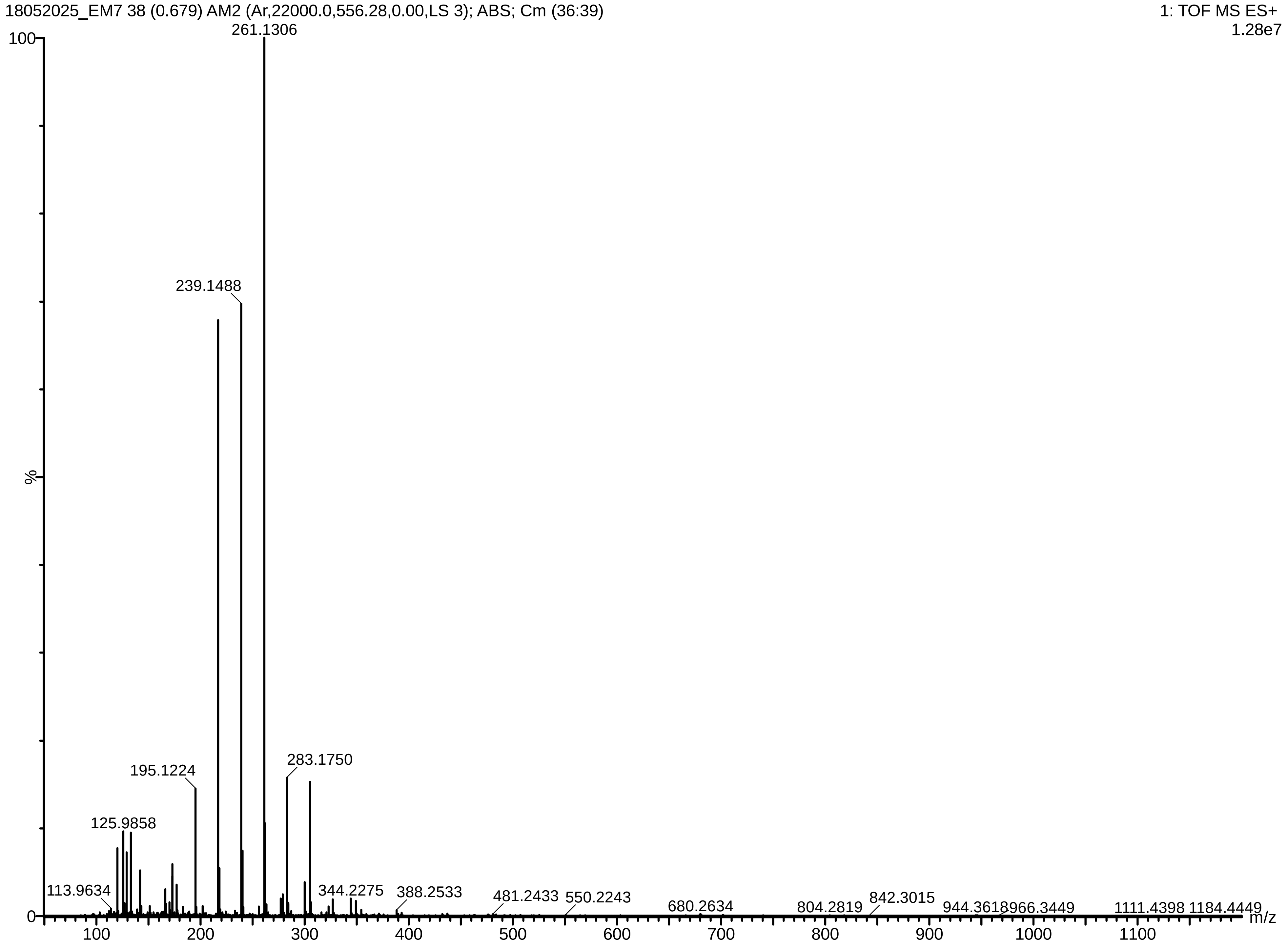

18052025_EM7 38 (0.679) AM2 (Ar,22000.0,556.28,0.00,LS 3); ABS; Cm (36:39)
1: TOF MS ES+
1.28e7
261.1306
239.1488
195.1224
283.1750
125.9858
113.9634
344.2275
388.2533
481.2433
550.2243
680.2634
804.2819
842.3015
944.3618
966.3449
1111.4398
1184.4449
%
m/z
100
0
100
200
300
400
500
600
700
800
900
1000
1100
