# Supplementary material for: Metabolomic atlas of dengue virus infection reveals distinct circulating bioactive lipid signatures
Source: PLoS Negl Trop Dis. 2026 May 12;20(5):e0014327. doi: 10.1371/journal.pntd.0014327 (PMC13189415; doi:10.1371/journal.pntd.0014327)
Supplement: S1 Data — Electrospray ionisation time of flight mass spectrometry (ESI-TOF MS, positive mode) spectra of the dengue cohort and ESI-TOF at different retention times. The spectra display the relative abundance (%) of detected ions across the m/z range. Prominent peaks corresponding to major ionised species are indicated. Variation in spectral profiles between retention times reflects the differences in compound composition and ionisation patterns within the sample. Data were acquired under identical instrumental conditions and are presented as representative scans. (ZIP) [file pntd.0014327.s003.zip › EM COMPLETE SAMPLES SPECTRUM/EM7 SPECTRUM RT 1.903.pdf]

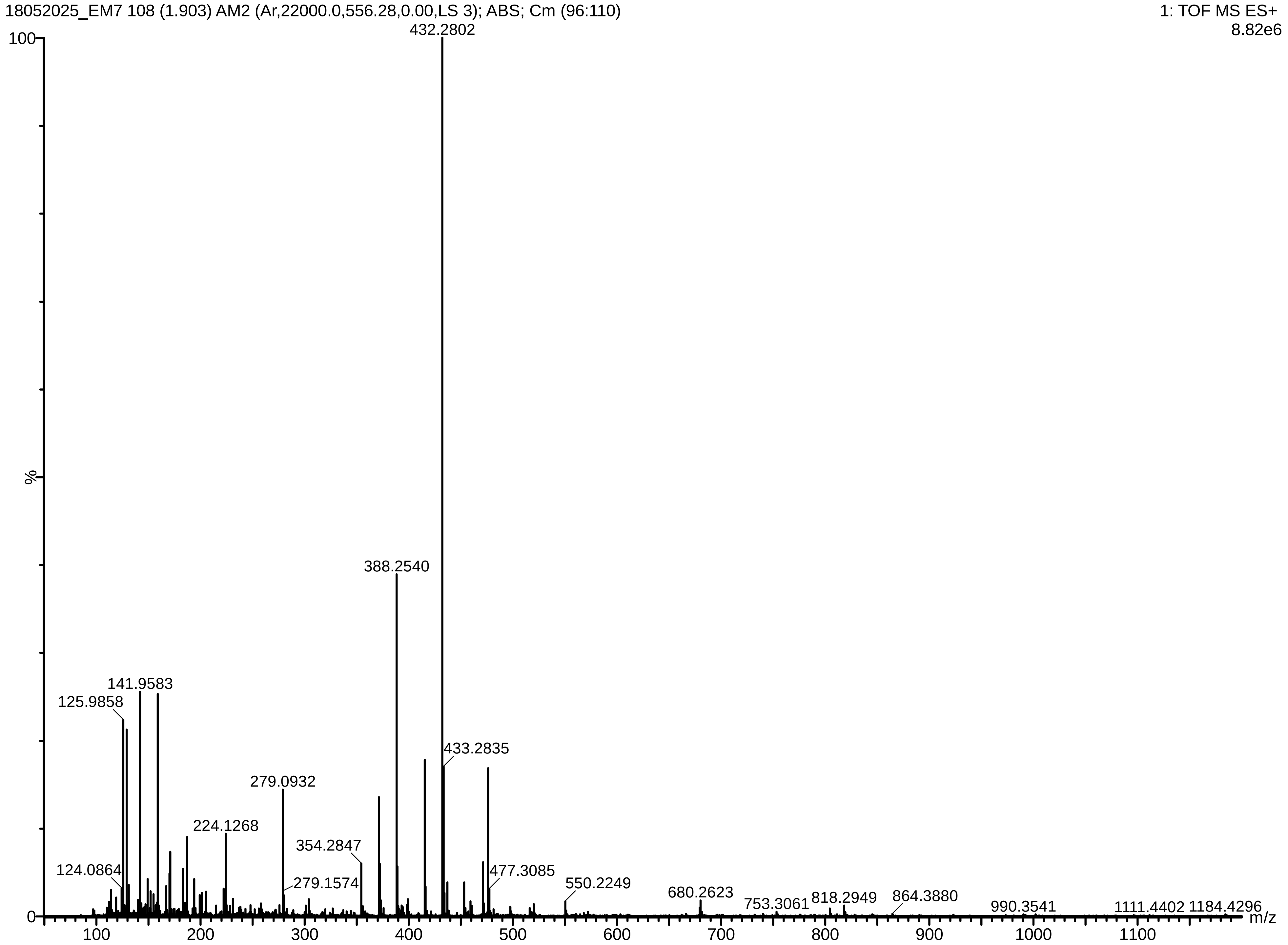

18052025_EM7 108 (1.903) AM2 (Ar,22000.0,556.28,0.00,LS 3); ABS; Cm (96:110)
1: TOF MS ES+
8.82e6
432.2802
388.2540
141.9583
125.9858
433.2835
279.0932
224.1268
354.2847
124.0864
477.3085
279.1574
550.2249
680.2623
753.3061
818.2949
864.3880
990.3541
1111.4402
1184.4296
%
100
0
100
200
300
400
500
600
700
800
900
1000
1100
m/z
