# Supplementary material for: Metabolomic atlas of dengue virus infection reveals distinct circulating bioactive lipid signatures
Source: PLoS Negl Trop Dis. 2026 May 12;20(5):e0014327. doi: 10.1371/journal.pntd.0014327 (PMC13189415; doi:10.1371/journal.pntd.0014327)
Supplement: S1 Data — Electrospray ionisation time of flight mass spectrometry (ESI-TOF MS, positive mode) spectra of the dengue cohort and ESI-TOF at different retention times. The spectra display the relative abundance (%) of detected ions across the m/z range. Prominent peaks corresponding to major ionised species are indicated. Variation in spectral profiles between retention times reflects the differences in compound composition and ionisation patterns within the sample. Data were acquired under identical instrumental conditions and are presented as representative scans. (ZIP) [file pntd.0014327.s003.zip › EM COMPLETE SAMPLES SPECTRUM/EM7 SPECTRUM RT 2.565.pdf]

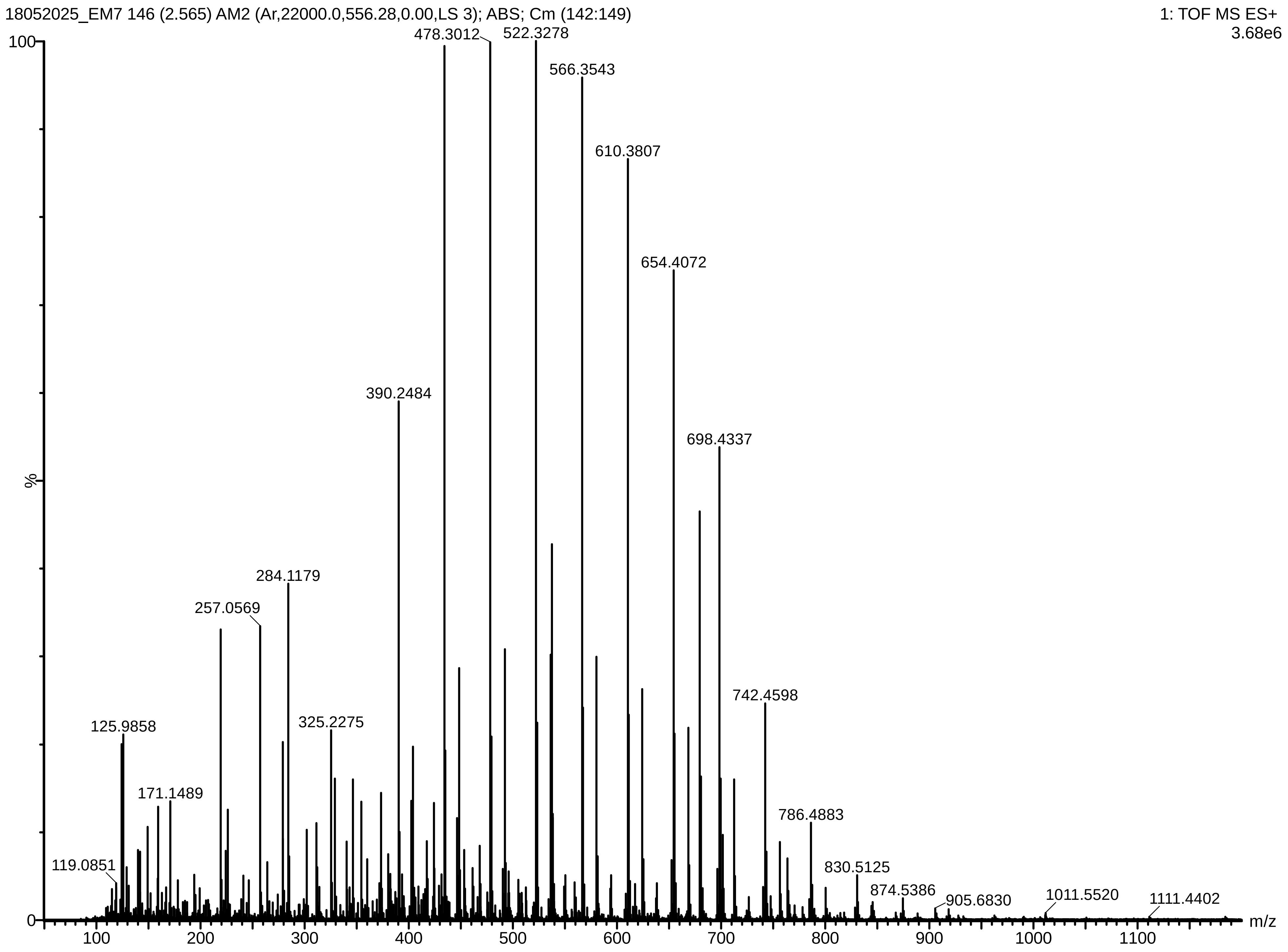
18052025_EM7 146 (2.565) AM2 (Ar,22000.0,556.28,0.00,LS 3); ABS; Cm (142:149)
1: TOF MS ES+
3.68e6
478.3012
522.3278
566.3543
610.3807
654.4072
390.2484
698.4337
284.1179
257.0569
742.4598
125.9858
325.2275
171.1489
786.4883
119.0851
830.5125
874.5386
905.6830
1011.5520
1111.4402
%
m/z
100
0
100
200
300
400
500
600
700
800
900
1000
1100
