# Supplementary material for: Metabolomic atlas of dengue virus infection reveals distinct circulating bioactive lipid signatures
Source: PLoS Negl Trop Dis. 2026 May 12;20(5):e0014327. doi: 10.1371/journal.pntd.0014327 (PMC13189415; doi:10.1371/journal.pntd.0014327)
Supplement: S1 Data — Electrospray ionisation time of flight mass spectrometry (ESI-TOF MS, positive mode) spectra of the dengue cohort and ESI-TOF at different retention times. The spectra display the relative abundance (%) of detected ions across the m/z range. Prominent peaks corresponding to major ionised species are indicated. Variation in spectral profiles between retention times reflects the differences in compound composition and ionisation patterns within the sample. Data were acquired under identical instrumental conditions and are presented as representative scans. (ZIP) [file pntd.0014327.s003.zip › EM COMPLETE SAMPLES SPECTRUM/EM7 SPECTRUM RT 2.771.pdf]

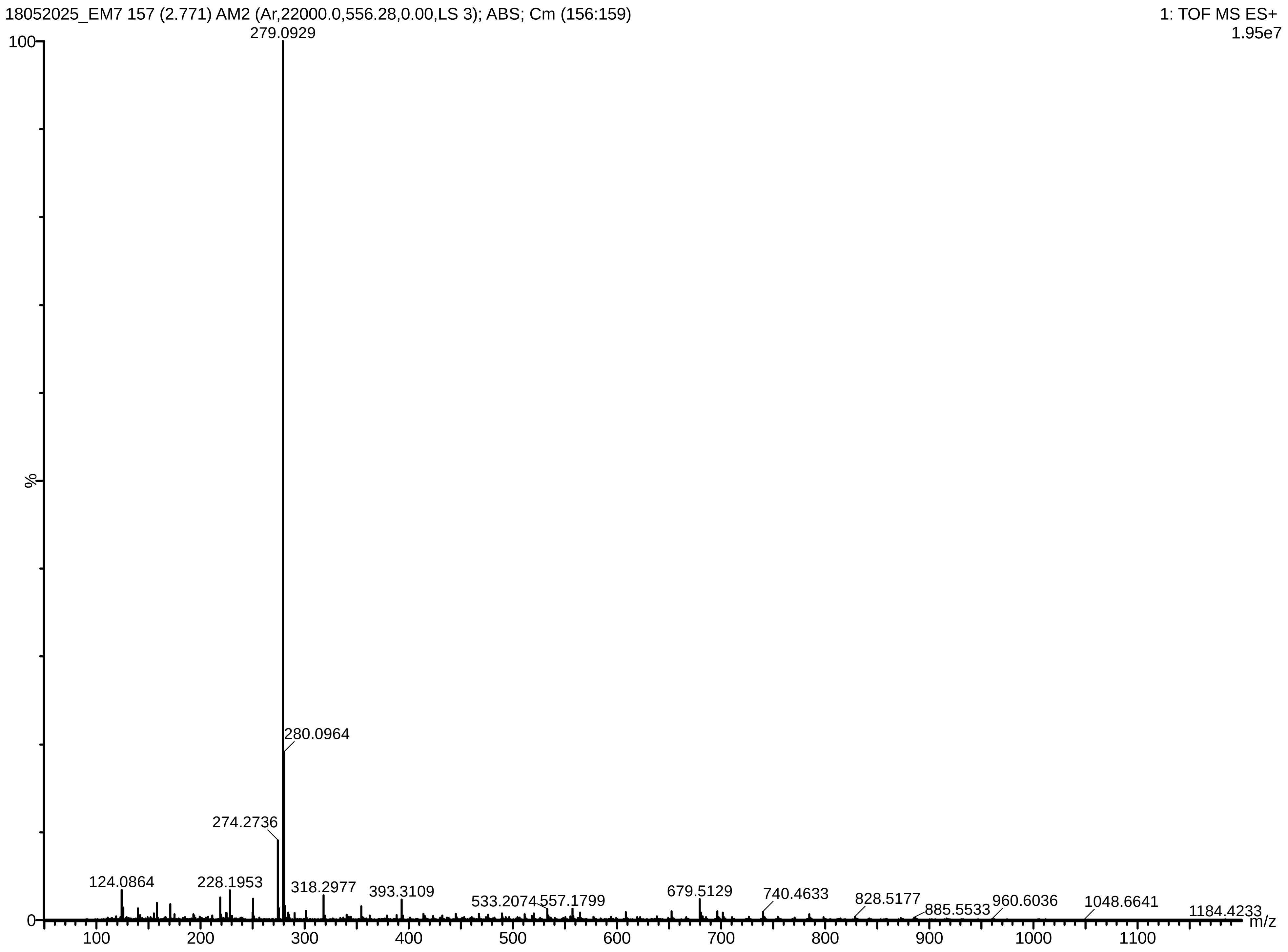

18052025_EM7 157 (2.771) AM2 (Ar,22000.0,556.28,0.00,LS 3); ABS; Cm (156:159)
1: TOF MS ES+
1.95e7
279.0929
280.0964
274.2736
124.0864
228.1953
318.2977
393.3109
533.2074
557.1799
679.5129
740.4633
828.5177
885.5533
960.6036
1048.6641
1184.4233
%
100
0
m/z
100
200
300
400
500
600
700
800
900
1000
1100
