# Supplementary material for: Metabolomic atlas of dengue virus infection reveals distinct circulating bioactive lipid signatures
Source: PLoS Negl Trop Dis. 2026 May 12;20(5):e0014327. doi: 10.1371/journal.pntd.0014327 (PMC13189415; doi:10.1371/journal.pntd.0014327)
Supplement: S1 Data — Electrospray ionisation time of flight mass spectrometry (ESI-TOF MS, positive mode) spectra of the dengue cohort and ESI-TOF at different retention times. The spectra display the relative abundance (%) of detected ions across the m/z range. Prominent peaks corresponding to major ionised species are indicated. Variation in spectral profiles between retention times reflects the differences in compound composition and ionisation patterns within the sample. Data were acquired under identical instrumental conditions and are presented as representative scans. (ZIP) [file pntd.0014327.s003.zip › EM COMPLETE SAMPLES SPECTRUM/EM7 SPECTRUM RT 2.873.pdf]

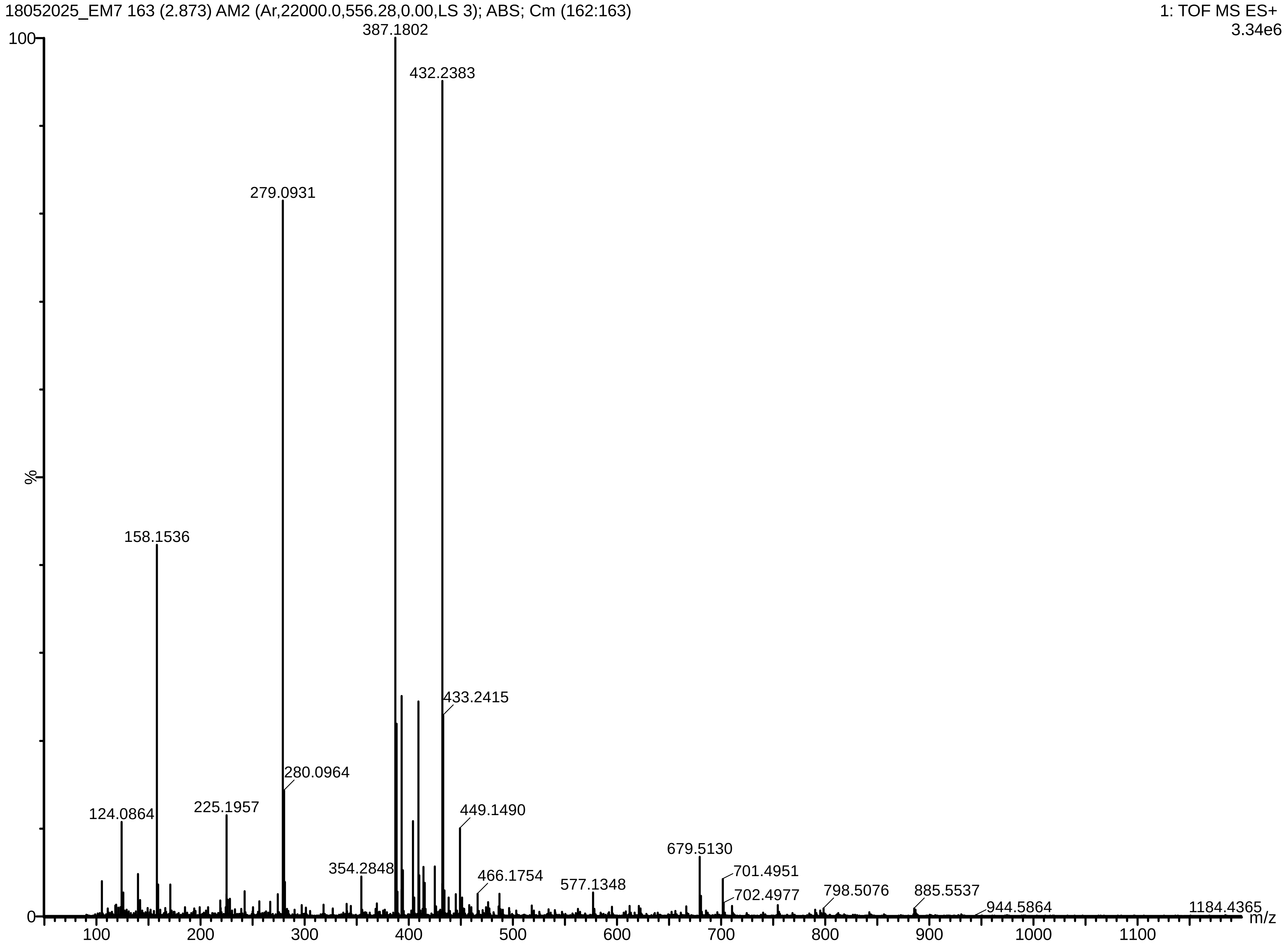

18052025_EM7 163 (2.873) AM2 (Ar,22000.0,556.28,0.00,LS 3); ABS; Cm (162:163)
1: TOF MS ES+
3.34e6
100
%
0
387.1802
432.2383
279.0931
158.1536
433.2415
280.0964
124.0864
225.1957
449.1490
679.5130
354.2848
701.4951
466.1754
577.1348
702.4977
798.5076
885.5537
944.5864
1184.4365
m/z
100
200
300
400
500
600
700
800
900
1000
1100
