# Supplementary material for: Metabolomic atlas of dengue virus infection reveals distinct circulating bioactive lipid signatures
Source: PLoS Negl Trop Dis. 2026 May 12;20(5):e0014327. doi: 10.1371/journal.pntd.0014327 (PMC13189415; doi:10.1371/journal.pntd.0014327)
Supplement: S1 Data — Electrospray ionisation time of flight mass spectrometry (ESI-TOF MS, positive mode) spectra of the dengue cohort and ESI-TOF at different retention times. The spectra display the relative abundance (%) of detected ions across the m/z range. Prominent peaks corresponding to major ionised species are indicated. Variation in spectral profiles between retention times reflects the differences in compound composition and ionisation patterns within the sample. Data were acquired under identical instrumental conditions and are presented as representative scans. (ZIP) [file pntd.0014327.s003.zip › EM COMPLETE SAMPLES SPECTRUM/EM7 SPECTRUM RT 3.092.pdf]

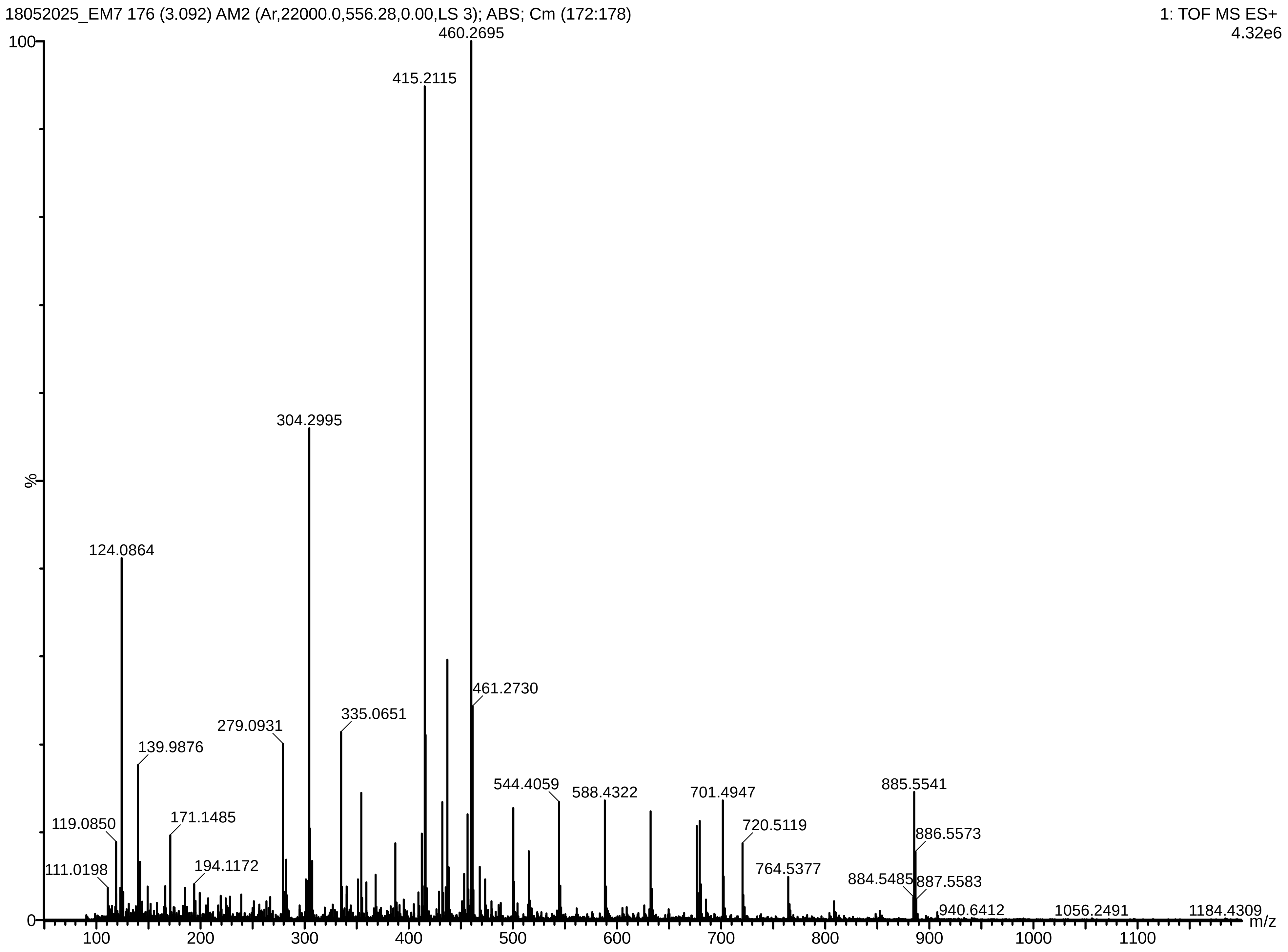

18052025_EM7 176 (3.092) AM2 (Ar,22000.0,556.28,0.00,LS 3); ABS; Cm (172:178)
1: TOF MS ES+
4.32e6
460.2695
415.2115
304.2995
124.0864
461.2730
335.0651
279.0931
139.9876
544.4059
588.4322
701.4947
885.5541
119.0850
171.1485
720.5119
886.5573
111.0198
194.1172
764.5377
884.5485
887.5583
940.6412
1056.2491
1184.4309
%
m/z
