# Supplementary material for: Metabolomic atlas of dengue virus infection reveals distinct circulating bioactive lipid signatures
Source: PLoS Negl Trop Dis. 2026 May 12;20(5):e0014327. doi: 10.1371/journal.pntd.0014327 (PMC13189415; doi:10.1371/journal.pntd.0014327)
Supplement: S1 Data — Electrospray ionisation time of flight mass spectrometry (ESI-TOF MS, positive mode) spectra of the dengue cohort and ESI-TOF at different retention times. The spectra display the relative abundance (%) of detected ions across the m/z range. Prominent peaks corresponding to major ionised species are indicated. Variation in spectral profiles between retention times reflects the differences in compound composition and ionisation patterns within the sample. Data were acquired under identical instrumental conditions and are presented as representative scans. (ZIP) [file pntd.0014327.s003.zip › EM COMPLETE SAMPLES SPECTRUM/EM7 SPECTRUM RT 3.279.pdf]

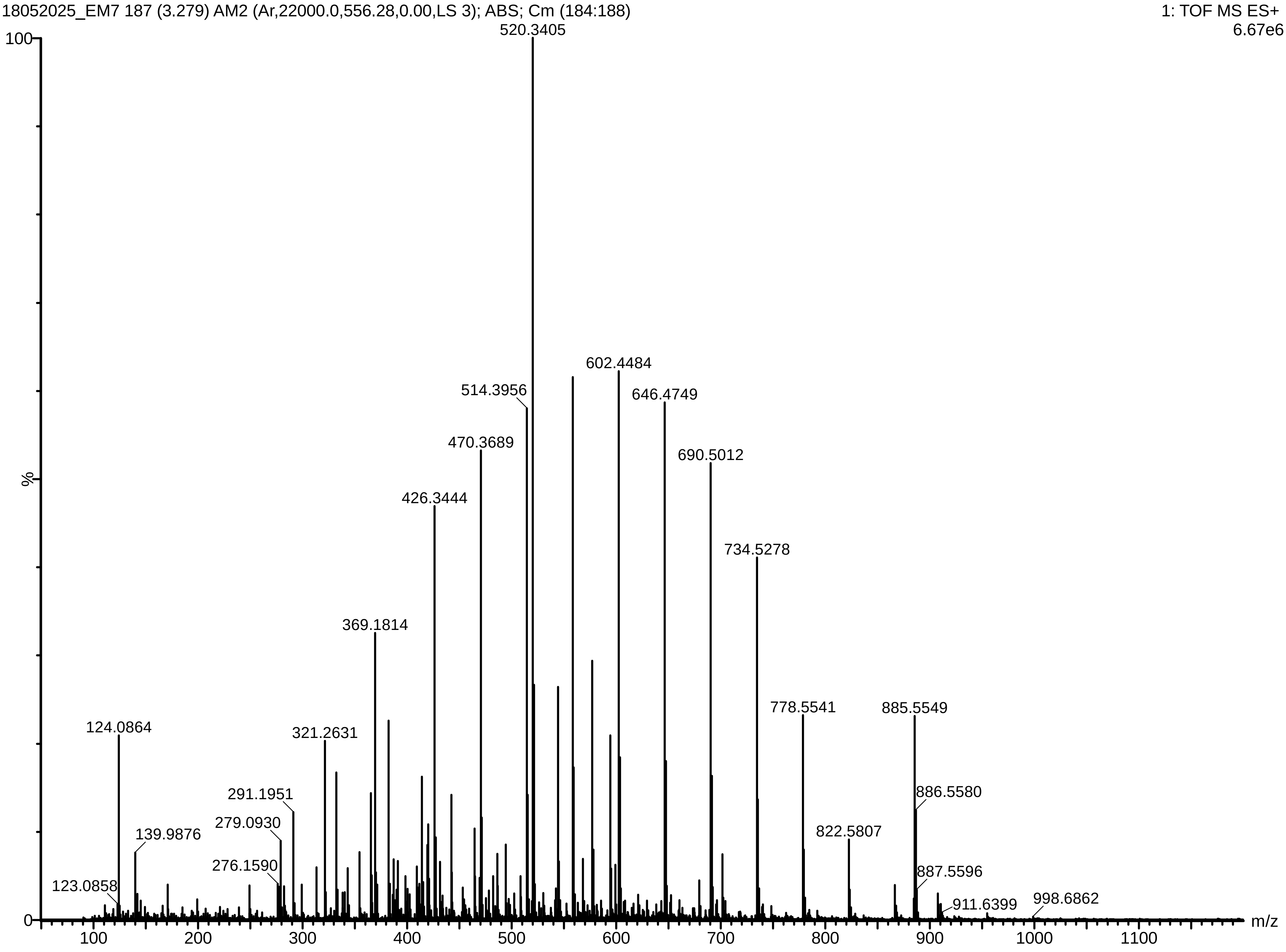
18052025_EM7 187 (3.279) AM2 (Ar,22000.0,556.28,0.00,LS 3); ABS; Cm (184:188)
1: TOF MS ES+
6.67e6
100
%
0
520.3405
602.4484
514.3956
646.4749
470.3689
690.5012
426.3444
734.5278
369.1814
778.5541
885.5549
124.0864
321.2631
886.5580
291.1951
279.0930
822.5807
139.9876
276.1590
887.5596
123.0858
998.6862
911.6399
100
200
300
400
500
600
700
800
900
1000
1100
m/z
