# Supplementary material for: Metabolomic atlas of dengue virus infection reveals distinct circulating bioactive lipid signatures
Source: PLoS Negl Trop Dis. 2026 May 12;20(5):e0014327. doi: 10.1371/journal.pntd.0014327 (PMC13189415; doi:10.1371/journal.pntd.0014327)
Supplement: S1 Data — Electrospray ionisation time of flight mass spectrometry (ESI-TOF MS, positive mode) spectra of the dengue cohort and ESI-TOF at different retention times. The spectra display the relative abundance (%) of detected ions across the m/z range. Prominent peaks corresponding to major ionised species are indicated. Variation in spectral profiles between retention times reflects the differences in compound composition and ionisation patterns within the sample. Data were acquired under identical instrumental conditions and are presented as representative scans. (ZIP) [file pntd.0014327.s003.zip › EM COMPLETE SAMPLES SPECTRUM/EM7 SPECTRUM RT 3.434.pdf]

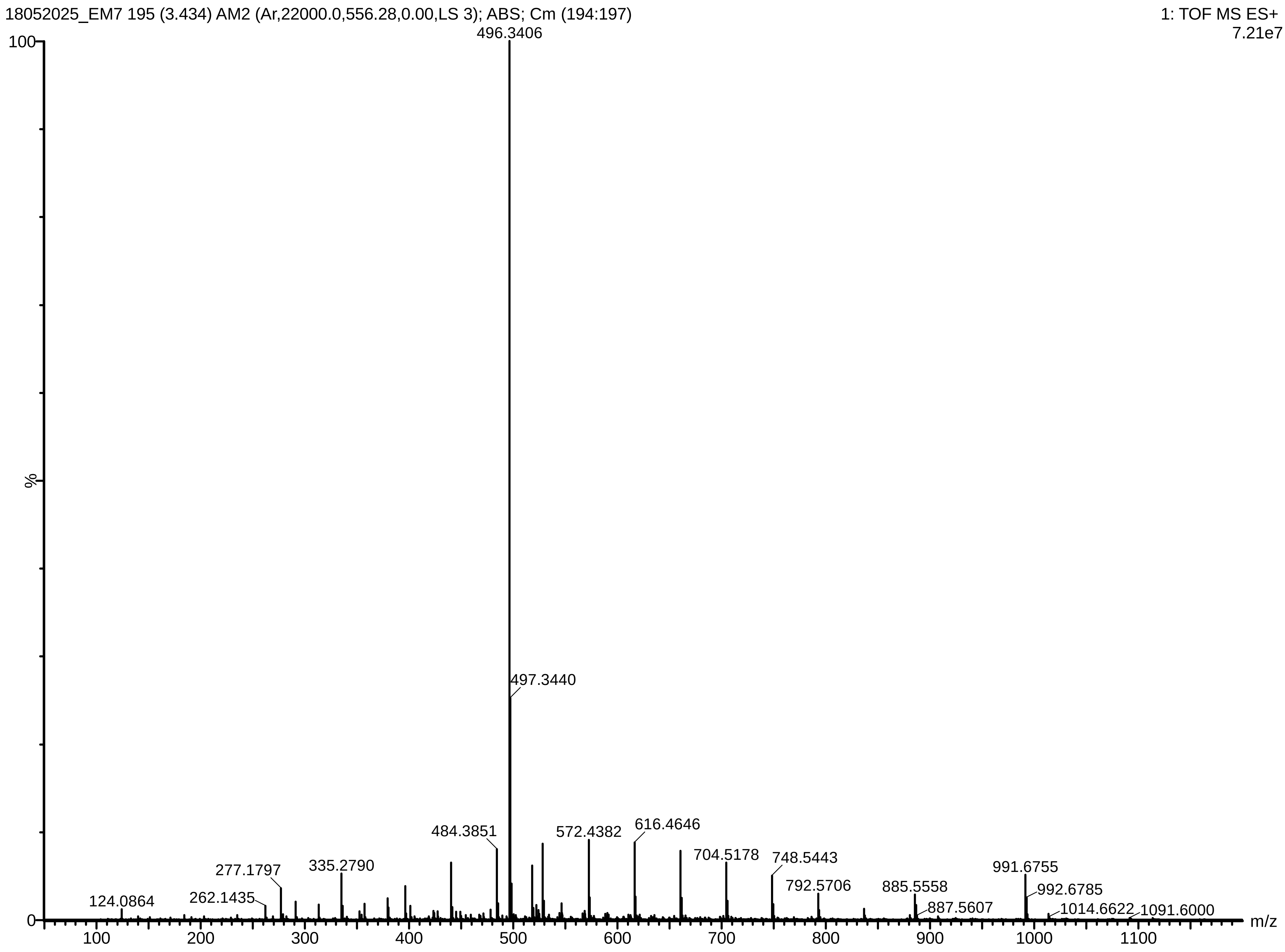

18052025_EM7 195 (3.434) AM2 (Ar,22000.0,556.28,0.00,LS 3); ABS; Cm (194:197)
1: TOF MS ES+
7.21e7
496.3406
497.3440
484.3851
572.4382
616.4646
704.5178
748.5443
792.5706
885.5558
887.5607
991.6755
992.6785
1014.6622
1091.6000
335.2790
277.1797
262.1435
124.0864
%
100
0
100
200
300
400
500
600
700
800
900
1000
1100
m/z
