# Supplementary material for: Metabolomic atlas of dengue virus infection reveals distinct circulating bioactive lipid signatures
Source: PLoS Negl Trop Dis. 2026 May 12;20(5):e0014327. doi: 10.1371/journal.pntd.0014327 (PMC13189415; doi:10.1371/journal.pntd.0014327)
Supplement: S1 Data — Electrospray ionisation time of flight mass spectrometry (ESI-TOF MS, positive mode) spectra of the dengue cohort and ESI-TOF at different retention times. The spectra display the relative abundance (%) of detected ions across the m/z range. Prominent peaks corresponding to major ionised species are indicated. Variation in spectral profiles between retention times reflects the differences in compound composition and ionisation patterns within the sample. Data were acquired under identical instrumental conditions and are presented as representative scans. (ZIP) [file pntd.0014327.s003.zip › EM COMPLETE SAMPLES SPECTRUM/EM7 SPECTRUM RT 3.823.pdf]

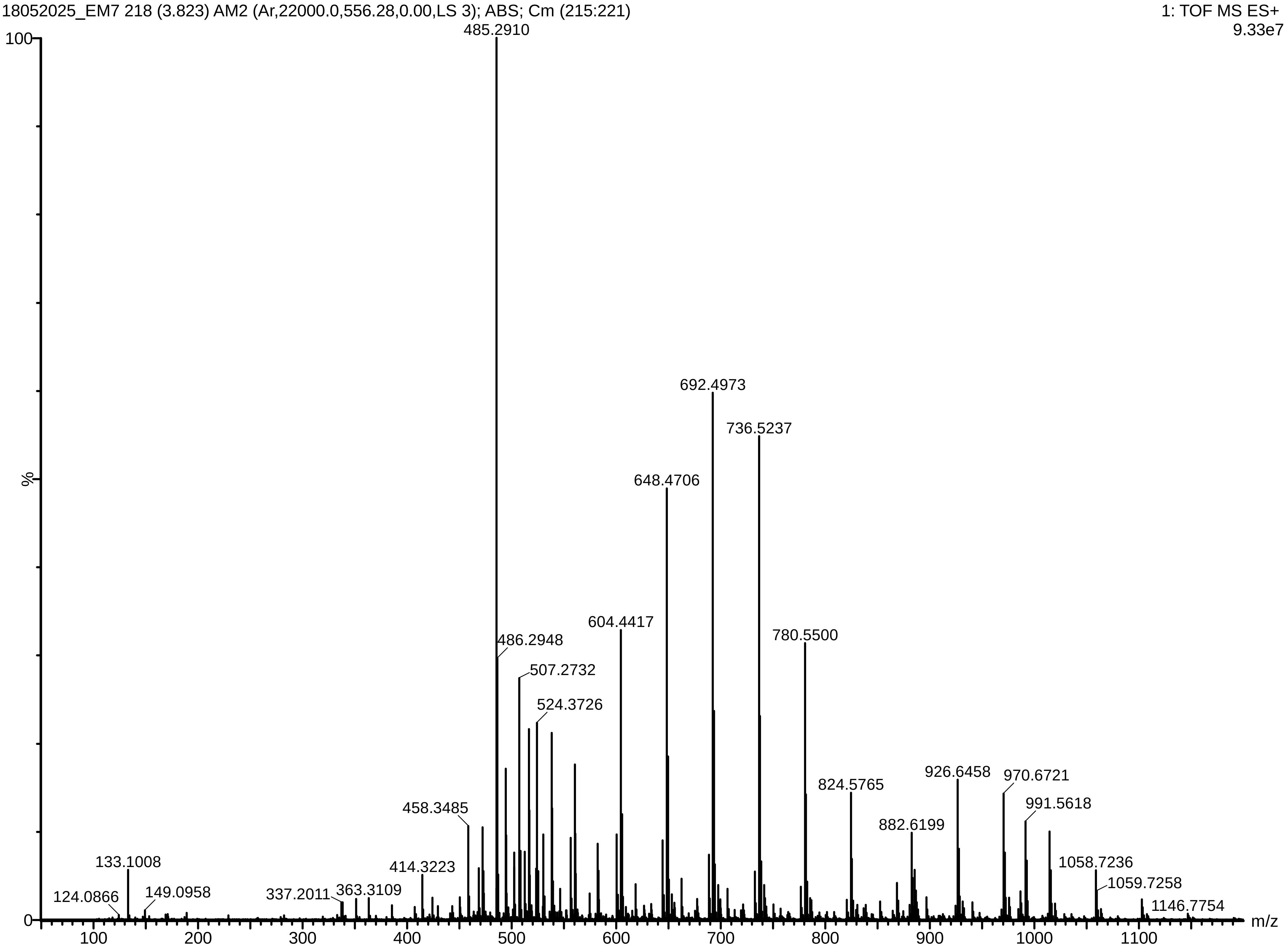
18052025_EM7 218 (3.823) AM2 (Ar,22000.0,556.28,0.00,LS 3); ABS; Cm (215:221)
1: TOF MS ES+
9.33e7
485.2910
692.4973
736.5237
648.4706
604.4417
486.2948
507.2732
524.3726
780.5500
926.6458
970.6721
824.5765
991.5618
458.3485
882.6199
414.3223
133.1008
1058.7236
1059.7258
124.0866
149.0958
337.2011
363.3109
1146.7754
100
%
0
100
200
300
400
500
600
700
800
900
1000
1100
m/z
