# Supplementary material for: Metabolomic atlas of dengue virus infection reveals distinct circulating bioactive lipid signatures
Source: PLoS Negl Trop Dis. 2026 May 12;20(5):e0014327. doi: 10.1371/journal.pntd.0014327 (PMC13189415; doi:10.1371/journal.pntd.0014327)
Supplement: S1 Data — Electrospray ionisation time of flight mass spectrometry (ESI-TOF MS, positive mode) spectra of the dengue cohort and ESI-TOF at different retention times. The spectra display the relative abundance (%) of detected ions across the m/z range. Prominent peaks corresponding to major ionised species are indicated. Variation in spectral profiles between retention times reflects the differences in compound composition and ionisation patterns within the sample. Data were acquired under identical instrumental conditions and are presented as representative scans. (ZIP) [file pntd.0014327.s003.zip › EM COMPLETE SAMPLES SPECTRUM/EM7 SPECTRUM RT 4.028.pdf]

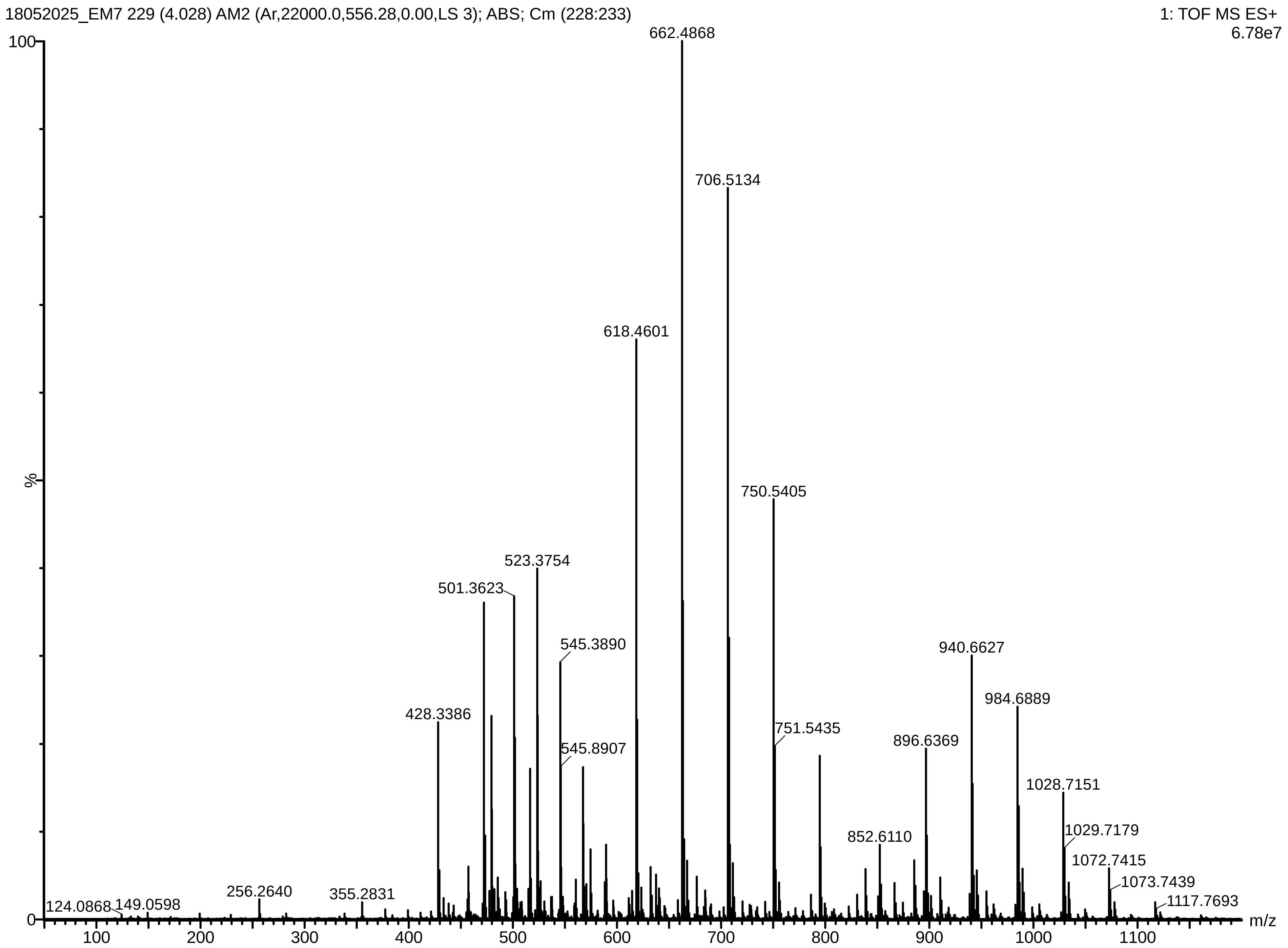

18052025_EM7 229 (4.028) AM2 (Ar,22000.0,556.28,0.00,LS 3); ABS; Cm (228:233)
1: TOF MS ES+
6.78e7
%
m/z
662.4868
706.5134
618.4601
750.5405
523.3754
501.3623
545.3890
940.6627
984.6889
428.3386
751.5435
896.6369
545.8907
1028.7151
1029.7179
852.6110
1072.7415
1073.7439
1117.7693
124.0868
149.0598
256.2640
355.2831
