# Supplementary material for: Metabolomic atlas of dengue virus infection reveals distinct circulating bioactive lipid signatures
Source: PLoS Negl Trop Dis. 2026 May 12;20(5):e0014327. doi: 10.1371/journal.pntd.0014327 (PMC13189415; doi:10.1371/journal.pntd.0014327)
Supplement: S1 Data — Electrospray ionisation time of flight mass spectrometry (ESI-TOF MS, positive mode) spectra of the dengue cohort and ESI-TOF at different retention times. The spectra display the relative abundance (%) of detected ions across the m/z range. Prominent peaks corresponding to major ionised species are indicated. Variation in spectral profiles between retention times reflects the differences in compound composition and ionisation patterns within the sample. Data were acquired under identical instrumental conditions and are presented as representative scans. (ZIP) [file pntd.0014327.s003.zip › EM COMPLETE SAMPLES SPECTRUM/EM89 SPECTRUM RT 0.476.pdf]

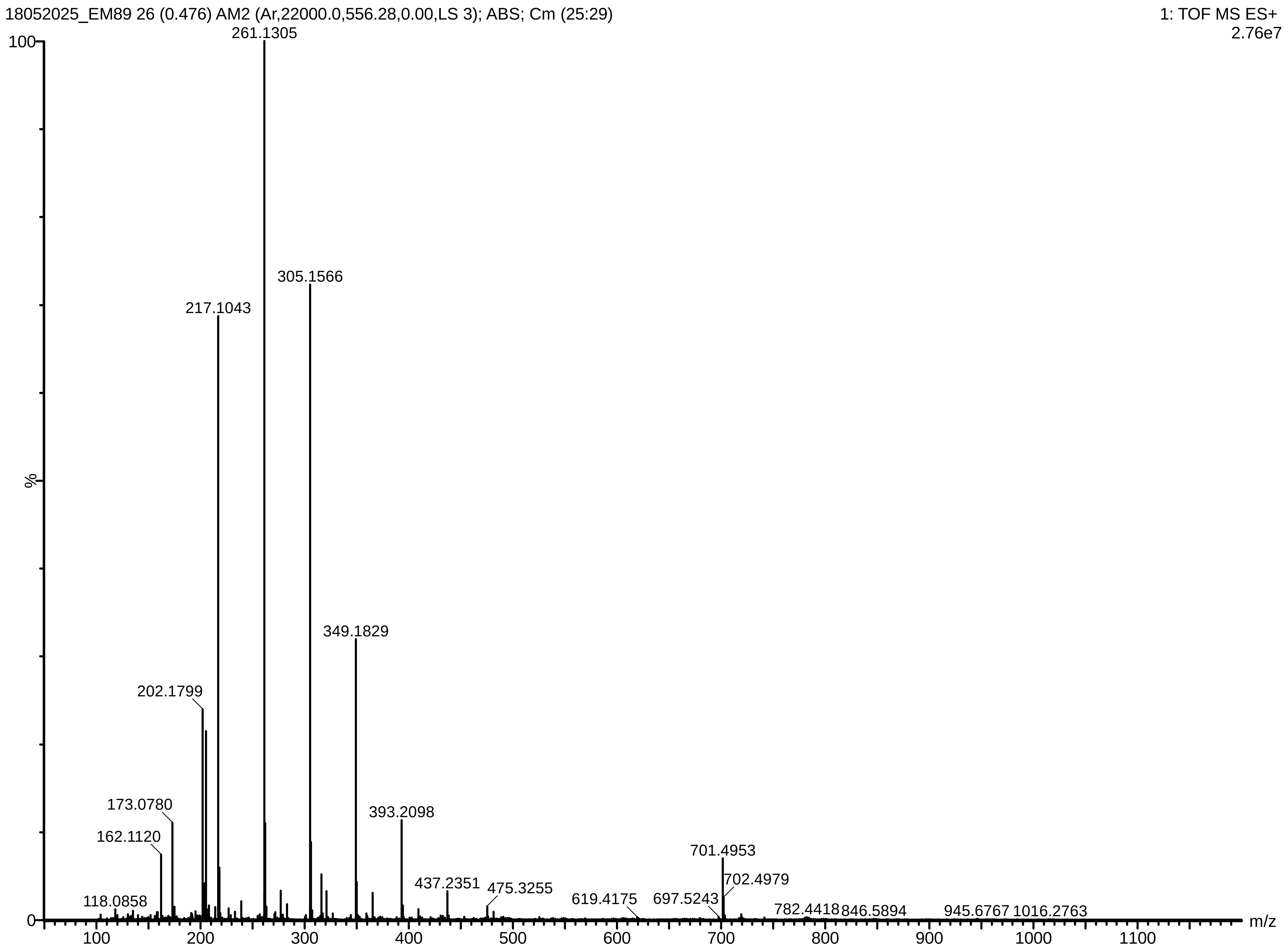
18052025_EM89 26 (0.476) AM2 (Ar,22000.0,556.28,0.00,LS 3); ABS; Cm (25:29)
1: TOF MS ES+
2.76e7
261.1305
305.1566
217.1043
349.1829
202.1799
173.0780
162.1120
393.2098
701.4953
702.4979
437.2351
475.3255
619.4175
697.5243
118.0858
782.4418
846.5894
945.6767
1016.2763
%
m/z
100
0
100
200
300
400
500
600
700
800
900
1000
1100
