# Supplementary material for: Metabolomic atlas of dengue virus infection reveals distinct circulating bioactive lipid signatures
Source: PLoS Negl Trop Dis. 2026 May 12;20(5):e0014327. doi: 10.1371/journal.pntd.0014327 (PMC13189415; doi:10.1371/journal.pntd.0014327)
Supplement: S1 Data — Electrospray ionisation time of flight mass spectrometry (ESI-TOF MS, positive mode) spectra of the dengue cohort and ESI-TOF at different retention times. The spectra display the relative abundance (%) of detected ions across the m/z range. Prominent peaks corresponding to major ionised species are indicated. Variation in spectral profiles between retention times reflects the differences in compound composition and ionisation patterns within the sample. Data were acquired under identical instrumental conditions and are presented as representative scans. (ZIP) [file pntd.0014327.s003.zip › EM COMPLETE SAMPLES SPECTRUM/EM89 SPECTRUM RT 0.629.pdf]

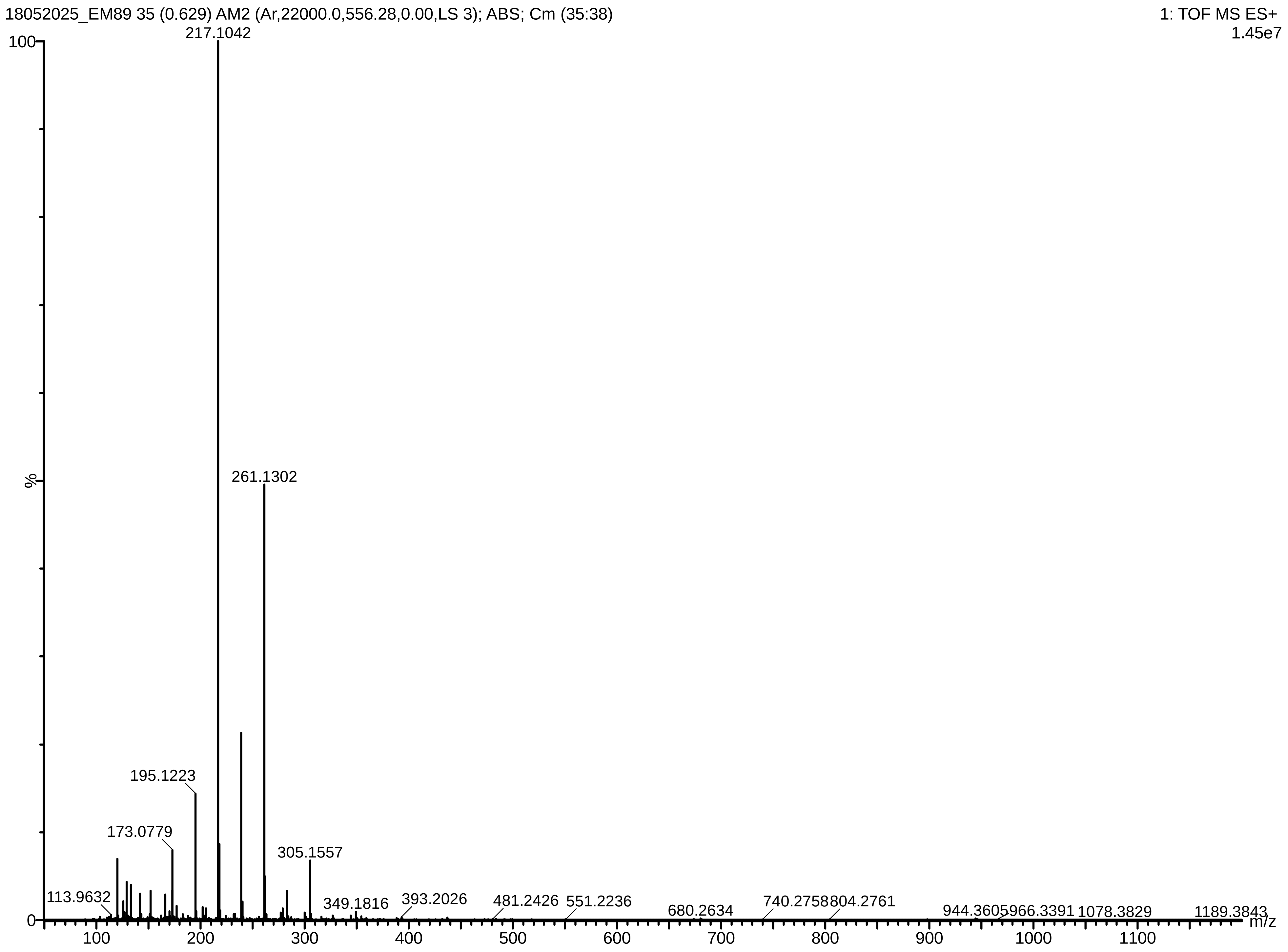

18052025_EM89 35 (0.629) AM2 (Ar,22000.0,556.28,0.00,LS 3); ABS; Cm (35:38)
1: TOF MS ES+
1.45e7
217.1042
261.1302
195.1223
173.0779
305.1557
113.9632
349.1816
393.2026
481.2426
551.2236
680.2634
740.2758
804.2761
944.3605
966.3391
1078.3829
1189.3843
100
%
0
m/z
100
200
300
400
500
600
700
800
900
1000
1100
