# Supplementary material for: Metabolomic atlas of dengue virus infection reveals distinct circulating bioactive lipid signatures
Source: PLoS Negl Trop Dis. 2026 May 12;20(5):e0014327. doi: 10.1371/journal.pntd.0014327 (PMC13189415; doi:10.1371/journal.pntd.0014327)
Supplement: S1 Data — Electrospray ionisation time of flight mass spectrometry (ESI-TOF MS, positive mode) spectra of the dengue cohort and ESI-TOF at different retention times. The spectra display the relative abundance (%) of detected ions across the m/z range. Prominent peaks corresponding to major ionised species are indicated. Variation in spectral profiles between retention times reflects the differences in compound composition and ionisation patterns within the sample. Data were acquired under identical instrumental conditions and are presented as representative scans. (ZIP) [file pntd.0014327.s003.zip › EM COMPLETE SAMPLES SPECTRUM/EM89 SPECTRUM RT 2.058.pdf]

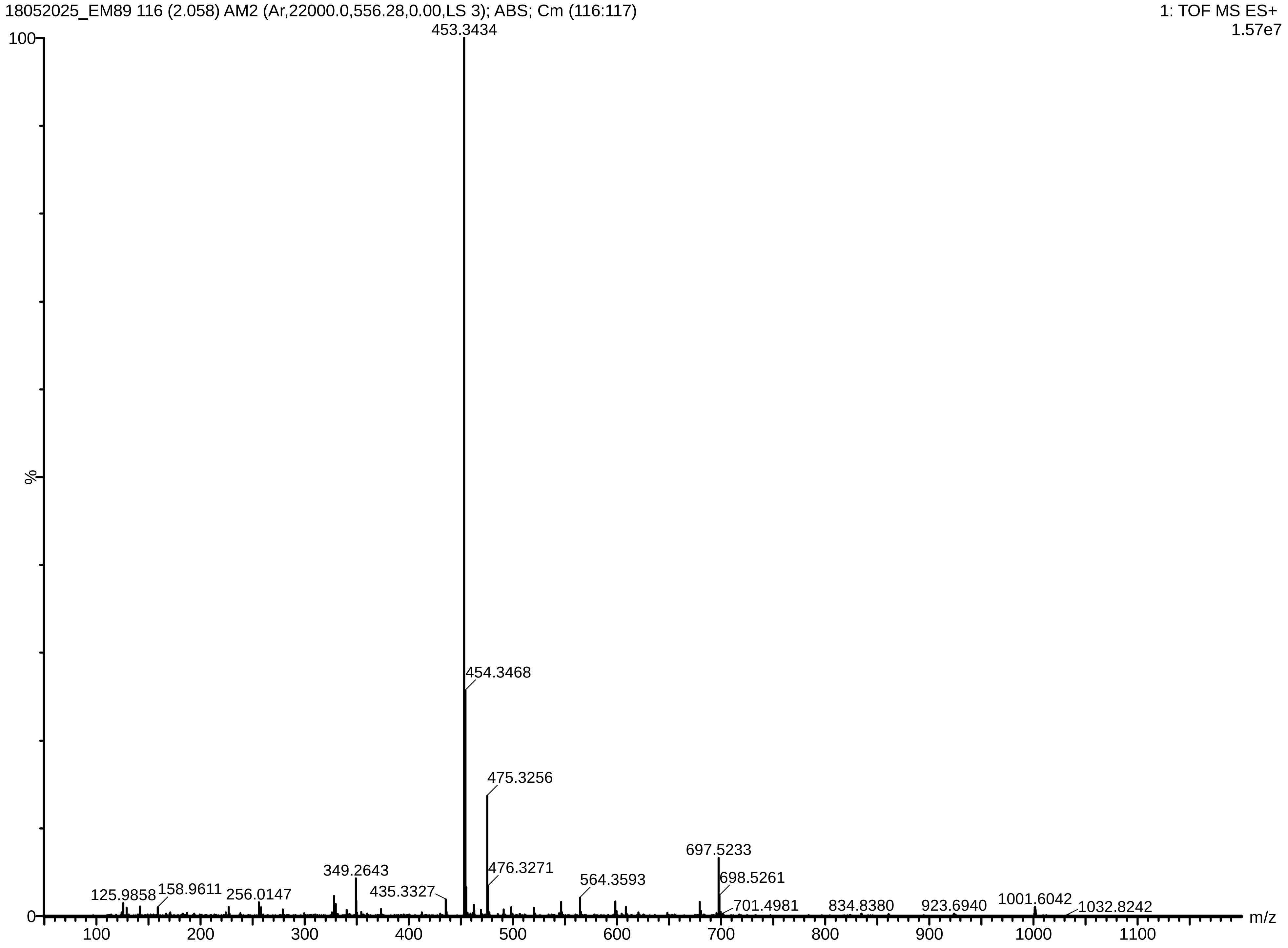
18052025_EM89 116 (2.058) AM2 (Ar,22000.0,556.28,0.00,LS 3); ABS; Cm (116:117)
1: TOF MS ES+
1.57e7
100
%
0
453.3434
454.3468
475.3256
476.3271
349.2643
435.3327
125.9858
158.9611
256.0147
564.3593
697.5233
698.5261
701.4981
834.8380
923.6940
1001.6042
1032.8242
100
200
300
400
500
600
700
800
900
1000
1100
m/z
