# Supplementary material for: Metabolomic atlas of dengue virus infection reveals distinct circulating bioactive lipid signatures
Source: PLoS Negl Trop Dis. 2026 May 12;20(5):e0014327. doi: 10.1371/journal.pntd.0014327 (PMC13189415; doi:10.1371/journal.pntd.0014327)
Supplement: S1 Data — Electrospray ionisation time of flight mass spectrometry (ESI-TOF MS, positive mode) spectra of the dengue cohort and ESI-TOF at different retention times. The spectra display the relative abundance (%) of detected ions across the m/z range. Prominent peaks corresponding to major ionised species are indicated. Variation in spectral profiles between retention times reflects the differences in compound composition and ionisation patterns within the sample. Data were acquired under identical instrumental conditions and are presented as representative scans. (ZIP) [file pntd.0014327.s003.zip › EM COMPLETE SAMPLES SPECTRUM/EM89 SPECTRUM RT 2.565.pdf]

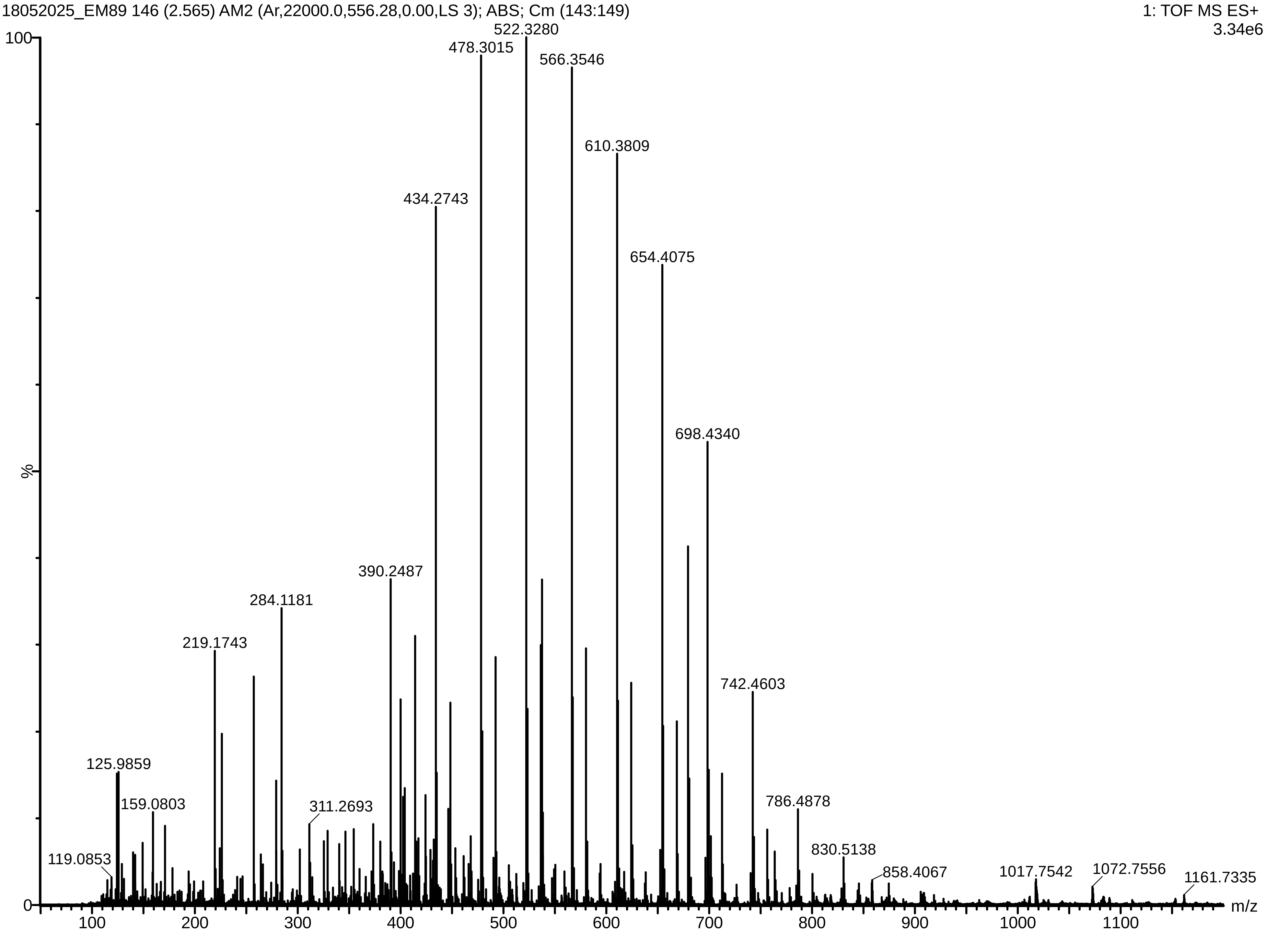

18052025_EM89 146 (2.565) AM2 (Ar,22000.0,556.28,0.00,LS 3); ABS; Cm (143:149)
1: TOF MS ES+
3.34e6
100
%
0
522.3280
478.3015
566.3546
610.3809
434.2743
654.4075
698.4340
390.2487
284.1181
219.1743
742.4603
125.9859
159.0803
311.2693
786.4878
830.5138
119.0853
858.4067
1017.7542
1072.7556
1161.7335
100
200
300
400
500
600
700
800
900
1000
1100
m/z
