# Supplementary material for: Metabolomic atlas of dengue virus infection reveals distinct circulating bioactive lipid signatures
Source: PLoS Negl Trop Dis. 2026 May 12;20(5):e0014327. doi: 10.1371/journal.pntd.0014327 (PMC13189415; doi:10.1371/journal.pntd.0014327)
Supplement: S1 Data — Electrospray ionisation time of flight mass spectrometry (ESI-TOF MS, positive mode) spectra of the dengue cohort and ESI-TOF at different retention times. The spectra display the relative abundance (%) of detected ions across the m/z range. Prominent peaks corresponding to major ionised species are indicated. Variation in spectral profiles between retention times reflects the differences in compound composition and ionisation patterns within the sample. Data were acquired under identical instrumental conditions and are presented as representative scans. (ZIP) [file pntd.0014327.s003.zip › EM COMPLETE SAMPLES SPECTRUM/EM89 SPECTRUM RT 2.873.pdf]

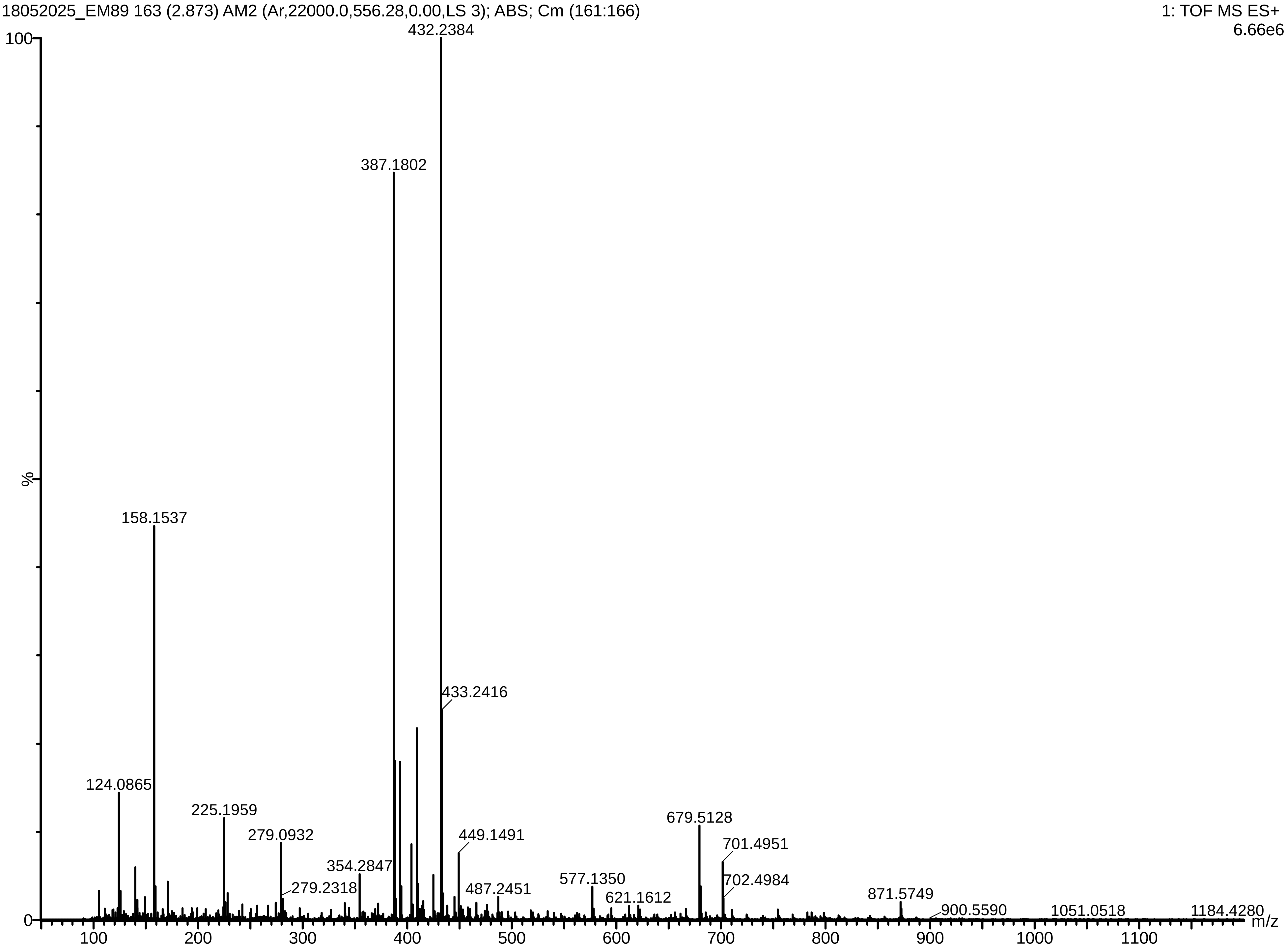
18052025_EM89 163 (2.873) AM2 (Ar,22000.0,556.28,0.00,LS 3); ABS; Cm (161:166)
1: TOF MS ES+
6.66e6
432.2384
387.1802
158.1537
433.2416
124.0865
225.1959
279.0932
279.2318
354.2847
449.1491
487.2451
577.1350
621.1612
679.5128
701.4951
702.4984
871.5749
900.5590
1051.0518
1184.4280
%
100
0
100
200
300
400
500
600
700
800
900
1000
1100
m/z
