# Supplementary material for: Metabolomic atlas of dengue virus infection reveals distinct circulating bioactive lipid signatures
Source: PLoS Negl Trop Dis. 2026 May 12;20(5):e0014327. doi: 10.1371/journal.pntd.0014327 (PMC13189415; doi:10.1371/journal.pntd.0014327)
Supplement: S1 Data — Electrospray ionisation time of flight mass spectrometry (ESI-TOF MS, positive mode) spectra of the dengue cohort and ESI-TOF at different retention times. The spectra display the relative abundance (%) of detected ions across the m/z range. Prominent peaks corresponding to major ionised species are indicated. Variation in spectral profiles between retention times reflects the differences in compound composition and ionisation patterns within the sample. Data were acquired under identical instrumental conditions and are presented as representative scans. (ZIP) [file pntd.0014327.s003.zip › EM COMPLETE SAMPLES SPECTRUM/EM89 SPECTRUM RT 3.279.pdf]

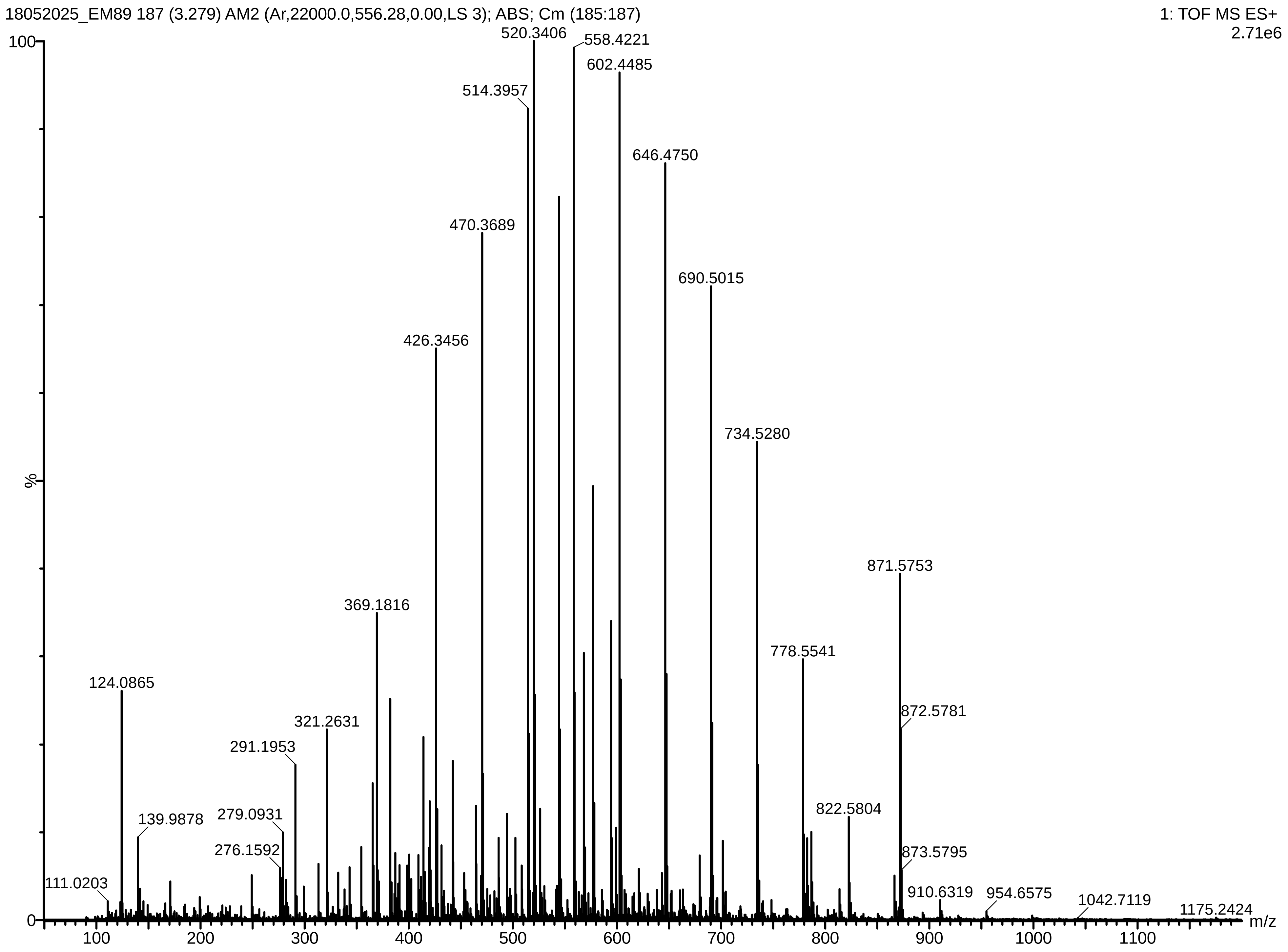
18052025_EM89 187 (3.279) AM2 (Ar,22000.0,556.28,0.00,LS 3); ABS; Cm (185:187)
1: TOF MS ES+
2.71e6
100
%
0
520.3406
558.4221
602.4485
514.3957
646.4750
470.3689
690.5015
426.3456
734.5280
871.5753
369.1816
778.5541
124.0865
872.5781
321.2631
291.1953
822.5804
139.9878
279.0931
276.1592
873.5795
111.0203
910.6319
954.6575
1042.7119
1175.2424
m/z
100
200
300
400
500
600
700
800
900
1000
1100
