# Supplementary material for: Metabolomic atlas of dengue virus infection reveals distinct circulating bioactive lipid signatures
Source: PLoS Negl Trop Dis. 2026 May 12;20(5):e0014327. doi: 10.1371/journal.pntd.0014327 (PMC13189415; doi:10.1371/journal.pntd.0014327)
Supplement: S1 Data — Electrospray ionisation time of flight mass spectrometry (ESI-TOF MS, positive mode) spectra of the dengue cohort and ESI-TOF at different retention times. The spectra display the relative abundance (%) of detected ions across the m/z range. Prominent peaks corresponding to major ionised species are indicated. Variation in spectral profiles between retention times reflects the differences in compound composition and ionisation patterns within the sample. Data were acquired under identical instrumental conditions and are presented as representative scans. (ZIP) [file pntd.0014327.s003.zip › EM COMPLETE SAMPLES SPECTRUM/EM89 SPECTRUM RT 3.434.pdf]

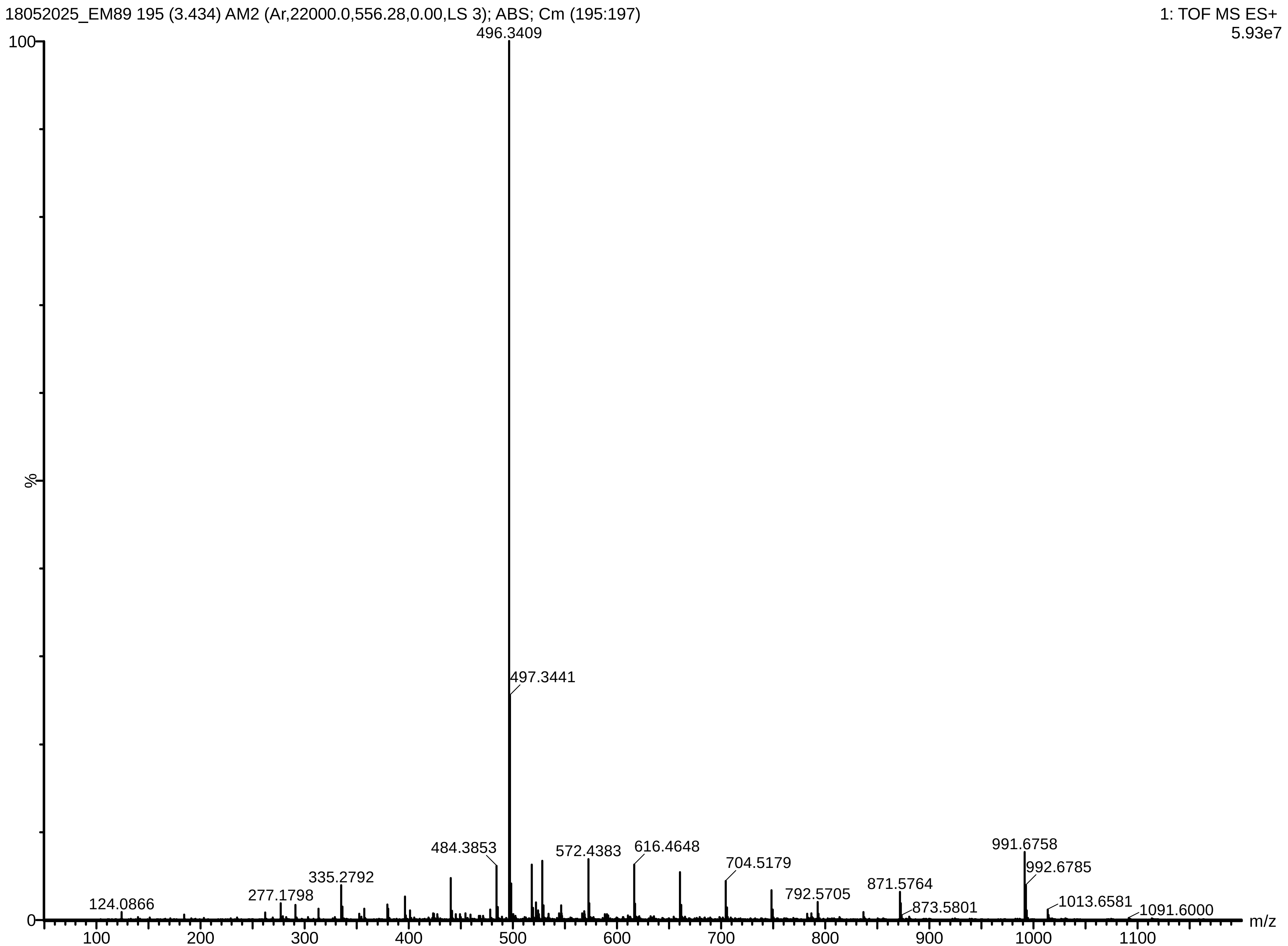
18052025_EM89 195 (3.434) AM2 (Ar,22000.0,556.28,0.00,LS 3); ABS; Cm (195:197)
1: TOF MS ES+
5.93e7
496.3409
497.3441
484.3853
572.4383
616.4648
704.5179
335.2792
277.1798
124.0866
792.5705
871.5764
873.5801
991.6758
992.6785
1013.6581
1091.6000
%
100
0
m/z
100
200
300
400
500
600
700
800
900
1000
1100
