# Supplementary material for: Metabolomic atlas of dengue virus infection reveals distinct circulating bioactive lipid signatures
Source: PLoS Negl Trop Dis. 2026 May 12;20(5):e0014327. doi: 10.1371/journal.pntd.0014327 (PMC13189415; doi:10.1371/journal.pntd.0014327)
Supplement: S1 Data — Electrospray ionisation time of flight mass spectrometry (ESI-TOF MS, positive mode) spectra of the dengue cohort and ESI-TOF at different retention times. The spectra display the relative abundance (%) of detected ions across the m/z range. Prominent peaks corresponding to major ionised species are indicated. Variation in spectral profiles between retention times reflects the differences in compound composition and ionisation patterns within the sample. Data were acquired under identical instrumental conditions and are presented as representative scans. (ZIP) [file pntd.0014327.s003.zip › EM COMPLETE SAMPLES SPECTRUM/EM89 SPECTRUM RT 3.823.pdf]

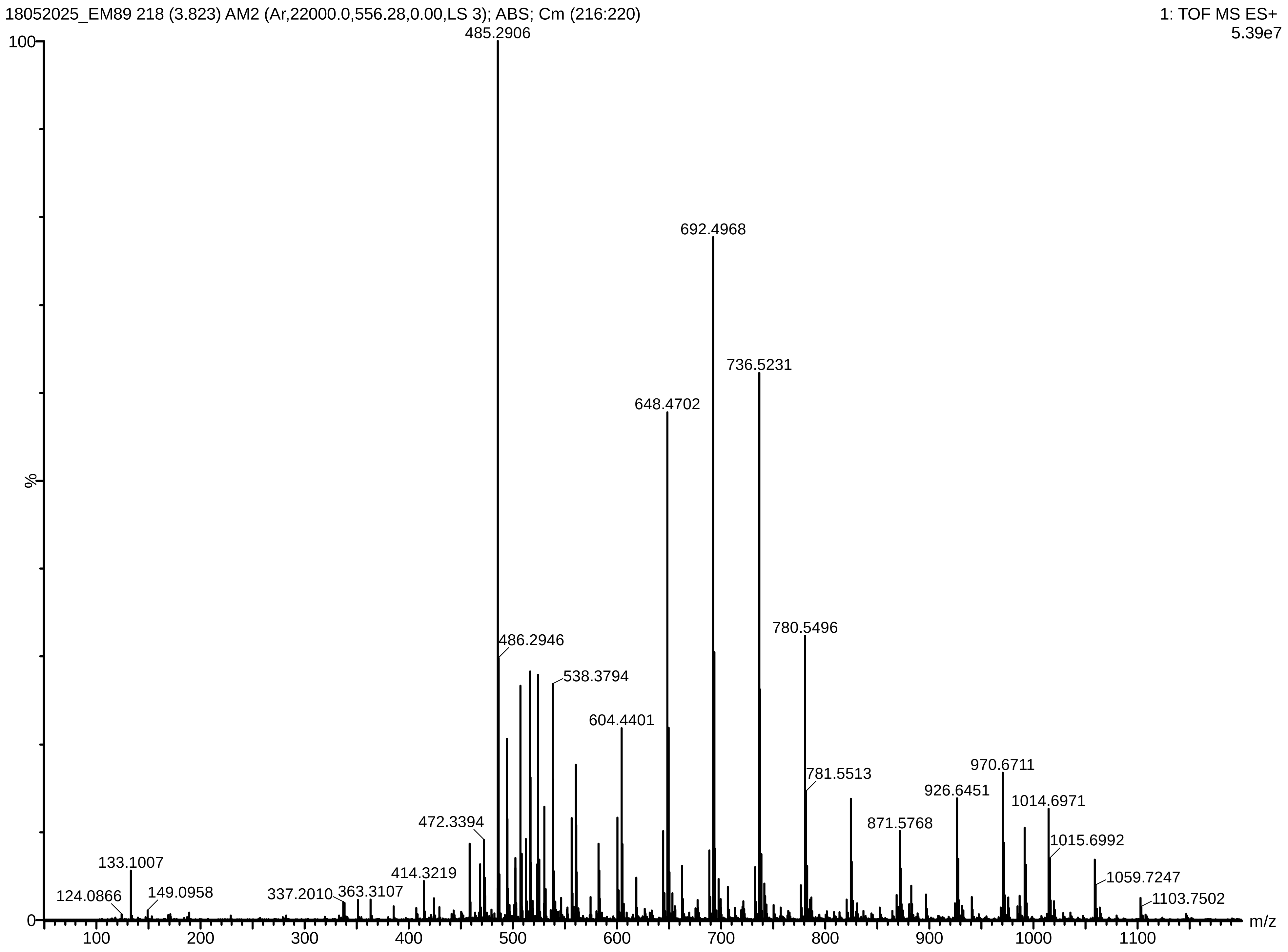
18052025_EM89 218 (3.823) AM2 (Ar,22000.0,556.28,0.00,LS 3); ABS; Cm (216:220)
1: TOF MS ES+
5.39e7
485.2906
692.4968
736.5231
648.4702
780.5496
486.2946
538.3794
604.4401
781.5513
970.6711
926.6451
1014.6971
871.5768
472.3394
1015.6992
133.1007
414.3219
1059.7247
124.0866
149.0958
337.2010
363.3107
1103.7502
%
m/z
0
100
100
200
300
400
500
600
700
800
900
1000
1100
